# Bimolecular Homolytic Substitution (S_H_2) and Radical Ligand Transfer (RLT): Emerging Paradigms in Radical Transformations

**DOI:** 10.1021/acscentsci.5c01091

**Published:** 2025-09-13

**Authors:** Anthony J. Fernandes, Dmitry Katayev

**Affiliations:** Department of Chemistry, Biochemistry and Pharmaceutical Sciences, 27210 University of Bern, 3012 Bern, Switzerland

## Abstract

Inspired by biological rebound processes, radical ligand transfer (RLT) has emerged as a powerful and versatile strategy for the selective functionalization of alkyl radicals. RLT enables direct C–X bond formation through homolytic substitution at a metal-bound ligand (M–X) and demonstrates broad functional group tolerance and high potential for catalysis. Despite growing interest and mechanistic understanding, including recent insights into asynchronous concerted ion–electron transfer (cIET), the broader application of RLT strategies remains underdeveloped. In parallel, the closely related S_H_2 (bimolecular homolytic substitution) mechanism has gained increasing utility in C–C bond formation, where low-valent metals capture transient radicals and facilitate selective coupling with persistent radical partners—a process referred to as radical sorting. Herein, we present a comprehensive perspective of the evolving landscape of RLT and S_H_2 chemistry, emphasizing recent advances. We highlight key bioinspired and computationally guided approaches that have enhanced mechanistic understanding and broadened the substrate scope, including landmark contributions by Kochi, Groves, Shaik, MacMillan, and others. To complement these studies and encourage further development, we also report DFT-based thermodynamic analyses of radical ligand transfer across first-row transition metal complexes bearing porphyrin and BOX ligands. By unifying these mechanistic frameworks, this perspective aims to provide a roadmap for designing novel, selective, and sustainable radical-based transformations.

## Graphical abstract

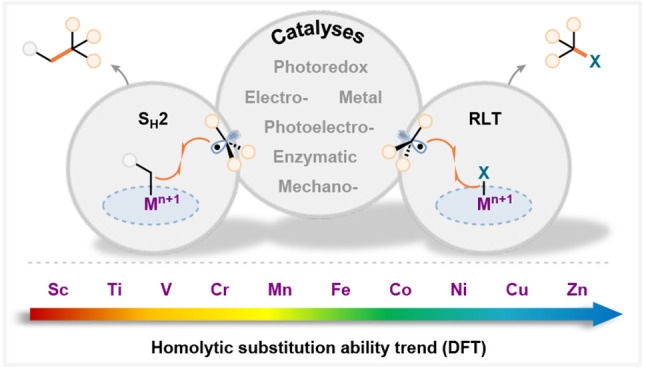

## 1 Introduction

Radical ligand transfer^1,2^ (RLT)—also referred to as oxidative ligand transfer (OLT)^3–5^ —has emerged as a versatile platform for the selective functionalization of alkyl radicals, bridging mechanistic insights from both biological “rebound” processes and synthetic radical chemistry. Although alkyl radicals have long been recognized as reactive intermediates, it is only in recent decades that they have been harnessed as selective and valuable tools for constructing C–C and C–X bonds using more sustainable and greener approaches.^6–12^ While moving away from the “tyranny of tin”,^8^ advances in radical generation via hydrogen atom transfer (HAT),^13,14^ alkene addition,^15–18^ decarboxylation,^19–21^ and in subsequent functionalization methods such as atom-transfer radical addition (ATRA)^22^ and radical-polar crossover (RPC)^23–26^ have enabled access to diverse alkyl radical intermediates. Yet the scope of these approaches remains limited by substrate class and the types of bonds that can be forged, creating an opportunity for a more generalizable strategy.

RLT addresses these challenges through an outer-sphere mechanism, wherein a heteroatomic ligand bound to a redox-active metal center M^n+1^–X is transferred directly to an alkyl radical R^•^, as first reported by Kochi.^3,27^ The corresponding transition state is characterized by a homolytic substitution at the X atom, forming a new C–X bond with the concomitant generation of the reduced metal M^*n*^ (Figure 1A).^28–30^ Subsequent reoxidation of the metal and coordination by a new X^–^ ligand reforms the M^*n*+1^–X species and completes the cycle, endowing RLT with inherent compatibility for catalytic applications.^31,32^

**Figure 1:**
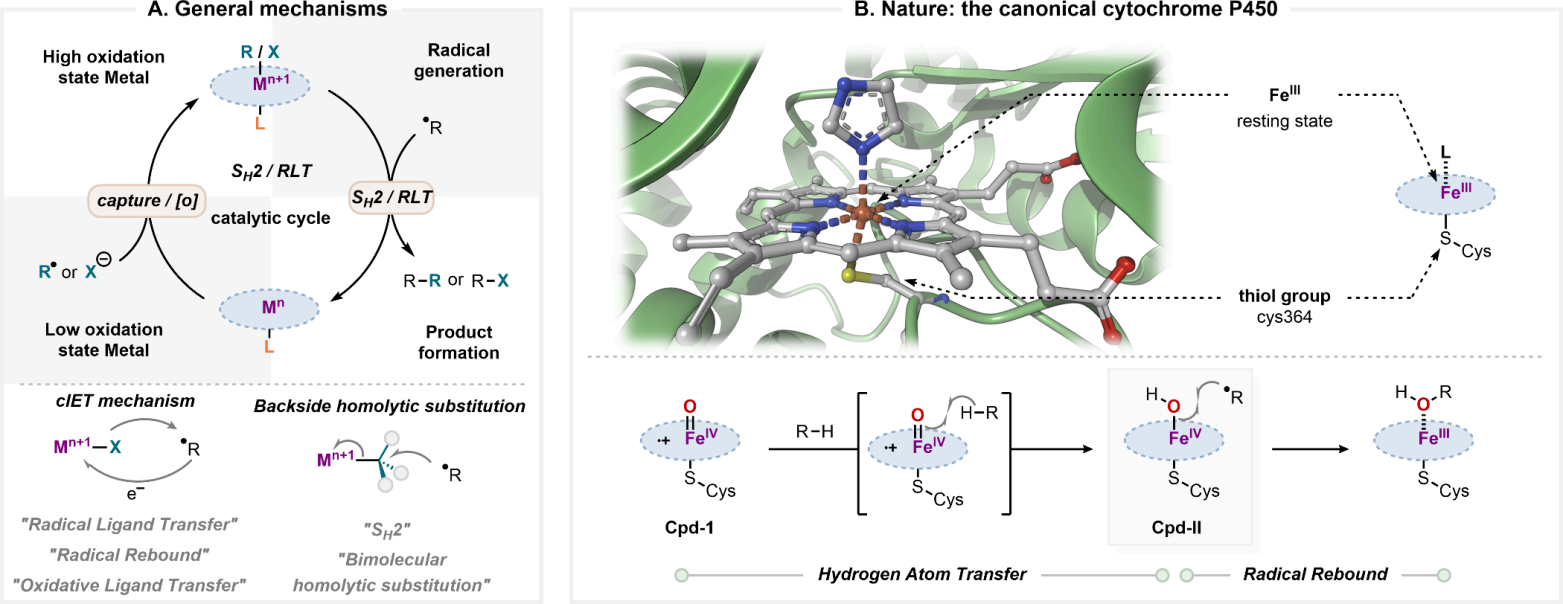
**A.** S_H_2 and RLT catalytic cycle, through radical capture or ligand exchange and oxidation, respectively. **B.** Structure of the cytochrome P450 active site (PDB: 2Z3T) and its canonical HAT-radical rebound mechanism.^54,55^

Nature’s archetypal example is the cytochrome P450 (Figure 1B). In its operating mechanism, a high-valent Fe^IV^=O species (**Cpd-I**) performs HAT from C–H bonds to generate Fe^III^–OH (**Cpd-II**) and an alkyl radical, with rapid rebound of the hydroxyl ligand yielding hydroxylated products.^30,33,34^ This “radical rebound” mechanism, initially proposed by Groves,^29^ received substantial theoretical support through the two-state reactivity (TSR) principle developed by Shaik and co-workers.^35,36^ More recently, molecular dynamics simulations by Houk^37^ and Ess^38,39^ have illuminated the diverse fates of the radical-pair intermediate prior to radical rebound. Additional computational studies by Srnec and co-workers revealed that the RLT step proceeds through an asynchronous concerted ion–electron transfer (cIET) in which electron transfer (ET) from the alkyl radical to the metal occurs in tandem with ion transfer (IT).^40^ This cIET framework resonates with earlier experimental observations by Groves and co-workers, who stated that fluorine and chlorine radical transfer “is best described as an asynchronous electron transfer from the substrate radical to iron followed by late ligand transfer to the substrate in the same single transition state, instead of a direct ligand radical transfer”.^41^ By mapping the thermodynamic cycles of ET and IT, Srnec’s group assessed the degree of synchronicity between these two coupled subevents that govern RLT reactivity and selectivity.^42^

While mechanistically related to RLT—albeit with underlying mechanistic nuances, bimolecular homolytic substitution (S_H_2)^43–46^ is a formidable radical process for constructing C–C bonds (Figure 1A). The S_H_2 designation was first introduced by Eliel^47,48^ as an analogue to Ingold’s S_N_2 classification,^49^ referring to a direct, concerted homolytic substitution reaction proceeding with Walden inversion.^46,50^ This mechanism was supported by observation of complete inversion of configuration in alkyl cobalt complexes upon reaction with radicals.^51–53^ Building on these and other mechanistic insights,^56–58^ MacMillan’s group designed a creative approach that harnesses the ability of low-oxidation state metals (e.g., Fe^II^ and Ni^II^), to capture and stabilize alkyl radicals, in a manner reminiscent of vitamin B_12_ cobalamin complex.^59^ These transformations are nowadays gaining significant importance, especially in photoredox catalysis and organometallic chemistry.^60^

Despite this rich history and recent advances in designing novel homolytic substitution reactions for C–X and C–C bond formation, the integration of RLT/S_H_2 pathways with transition-metal (TM) catalysis remains underexplored.^61^ Recent studies have demonstrated that Co, Ni, and Fe complexes can serve as competent catalysts for the RLT/S_H_2 processes. Among these, Fe-based catalysis is particularly appealing due to iron’s abundance in the Earth's crust and its high global production—accounting for 96% of the total production of first-row transition metals (1st-row TMs)—offering a more sustainable alternative to other first row TMs.^62–64^ Nevertheless, expanding the scope to include metals such as copper, manganese, and chromium may unveil new reactivity and selectivity patterns, thereby broadening the applicability of these transformations.^41,60^ Notably, the reactivity of various metal centers toward RLT/S_H_2 remains insufficiently studied and poorly understood, lacking a predictive framework. During the peer review process of this manuscript, a perspective article was published by MacMillan and co-workers on S_H_2 catalysis, the readers are advised to refer to this complementary contribution.^65^

To inspire and guide future efforts, we highlight here key contributions that have shaped the development of this emerging field and present a unified treatment of the RLT and S_H_2 mechanisms in transition-metal systems. Additionally, to encourage and facilitate exploration, we have computed the thermodynamic driving force for radical transfer involving various ligands across first row TM complexes adorned with porphyrin and BOX ligands. By delineating both the scope and the mechanistic subtleties of RLT and S_H_2, we aim to chart a path forward for the development of selective and sustainable radical-based transformations in both synthetic and biological contexts.

## 2 Implementation in Synthetic Methodology

### C–C Bond Formation by S_H_2 Catalysis

The past decade has seen a renaissance in radical homolytic substitution reactions with S_H_2 emerging as a versatile mechanistic platform for creating C–C bonds. When combined with complementary catalytic strategies, S_H_2 has facilitated the development of increasingly sophisticated cross-coupling processes (Figure 2).

**Figure 2:**
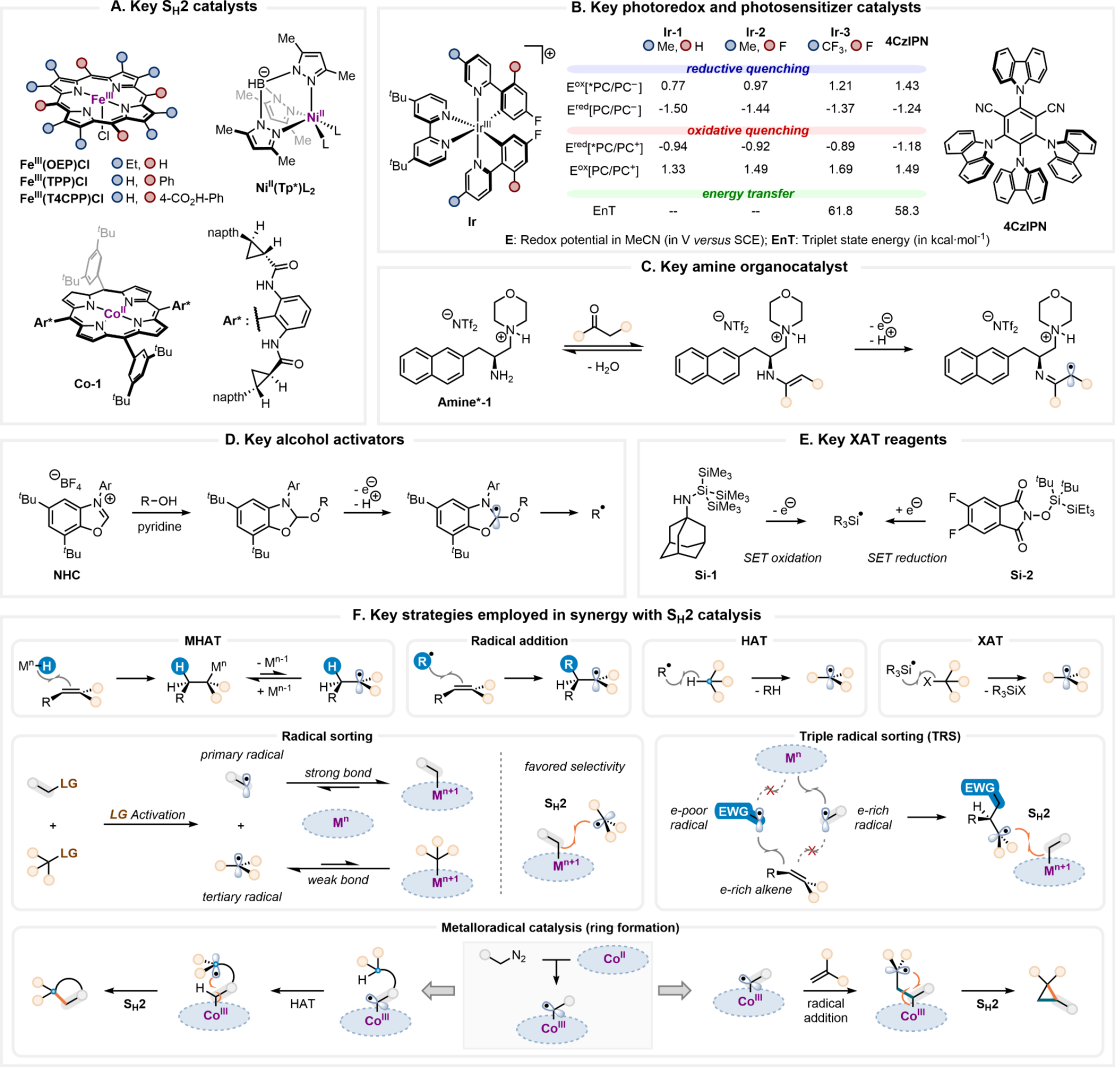
**A.** Selected S_H_2 catalysts. **B.** Selected photocatalysts and their properties.^74–76^ **C.** Selected amine organocatalyst. **D.** Selected NHC involved in alcohol activation. **E.** Selected redox-responsive silyl radical precursors involved in XAT strategy. **F.** Key strategies employed in synergy with S_H_2 catalysis.

A foundational advance originated from the MacMillan group, whose 2021 report introduced a biomimetic sp^3^-sp^3^ cross-coupling of redox-active esters (RAEs) and alkyl bromides using dual photoredox and iron-mediated S_H_2 catalysis (Figure 2A, B).^59^ This transformation involves the photoexcitation of Ir^III^(ppy)_3_ and oxidation of silane **Si-1** to generate a silyl radical that initiates XAT with an alkyl bromide, forming a primary radical (Figure 2E, F). The radical is intercepted by a Fe^II^(OEP) complex to generate an alkyl Fe^III^ intermediate, which undergoes S_H_2 with a tertiary radical—formed via reduction of the RAE by Ir^II^(ppy)_3_. This transformation enabled the construction of sterically congested quaternary carbon centers (**1**, Figure 3) via a key S_H_2 step, laying the groundwork for the development of innovative cross-couplings. The group expanded this platform with a double decarboxylative cross-coupling (**2**) mediated by a Ni^II^(scorpionate) S_H_2 catalyst (Figure 2A),^66^ and further developed analogous carboxylic acid-alcohol (**3**),^67^ alcohol-alkyl bromide (**4**),^68^ and alcohol–alcohol cross-couplings (**5**).^69^ In addition, a cross-electrophile coupling strategy involving tertiary and primary alkyl bromides or sulfonates (**9**) was designed.^70^

**Figure 3:**
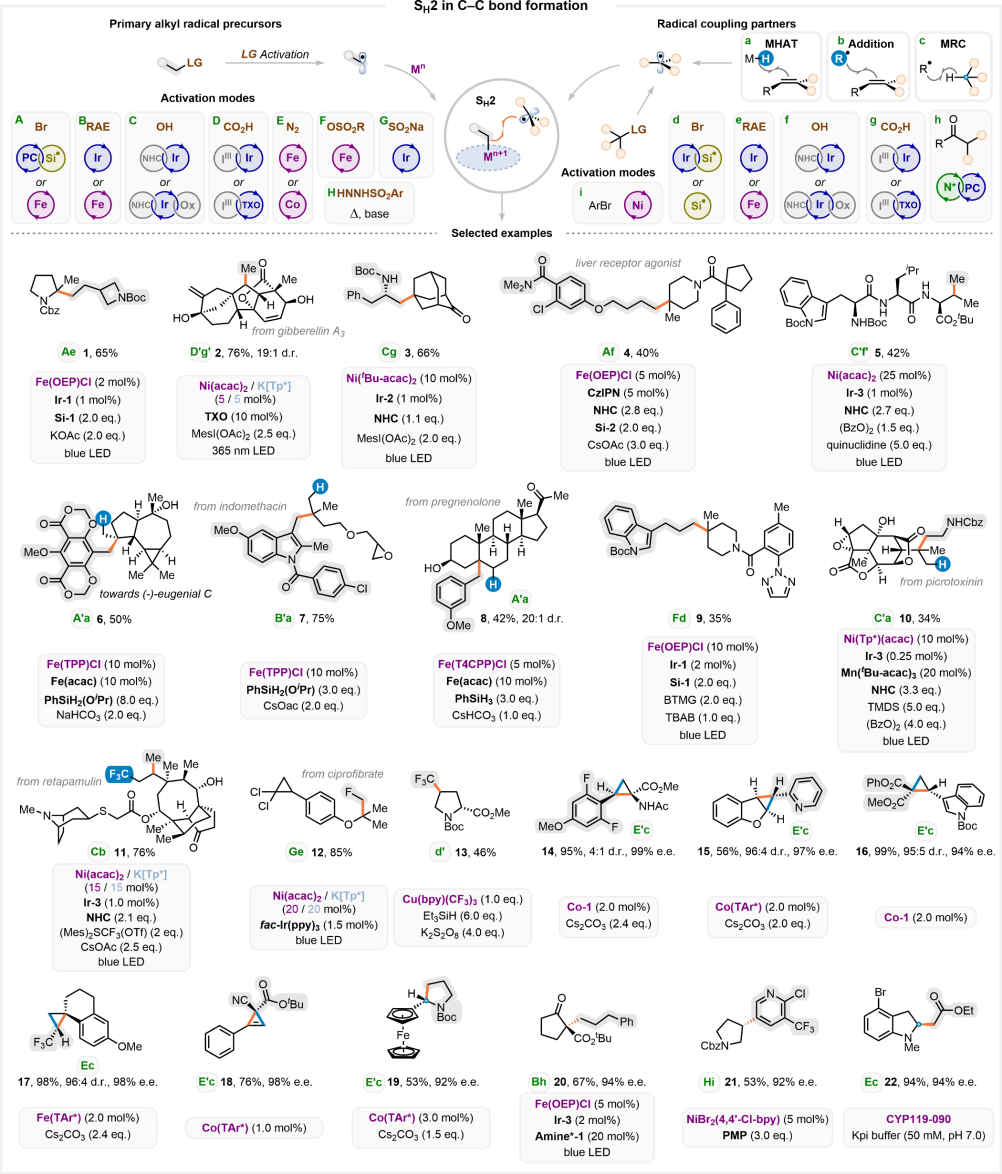
S_H_2 chemistry in C–C bond construction. Selected examples of S_H_2-mediated synthesis of complex targets are represented alongside key reaction conditions. The activation strategies used for the generation of the two radical partners (A–H and a–i) are given below the molecular structure as green letters. The alkyl fragment that is bonded to the metal in the S_H_2 step is highlighted in gray.

In a complementary approach, the Shenvi group addressed a long-standing challenge in the total synthesis of (−)-eugenial—a meroterpenoid with notable antibacterial activity. They relied on the merger of metal-hydride atom transfer (MHAT)^71^ which generates a tertiary radical after insertion of an alkene into a Mn–H bond (Figure 2F), and S_H_2-catalysis (**6**).^72^ This tandem sequence enabled high diastereoselectivity (d.r. > 10:1), attributed to the facial discrimination during the S_H_2 step. Furthermore, the synergy of MHAT-S_H_2 was applied to Fe^II^(porphyrin)-catalyzed decarboxylative coupling of RAEs and olefins (**7**),^73^ as well as to Fe-mediated reductive RAE-RAE cross-couplings that complement MacMillan’s protocol (**2**).^66^ Shenvi also independently reported Fe^II^(porphyrin)-catalyzed hydrobenzylation of alkenes via MHAT/S_H_2 catalysis (**8**).^77^ MacMillan’s group also leveraged MHAT to generate tertiary radicals for coupling them with key alkyl Ni^III^(scorpionate) complexes derived from alcohols (**10**).^78^

Pushing the boundaries of the concept, the same group introduced a sophisticated triple radical sorting method,^79,80^ where both the philicity^81–83^ and size of the radicals play critical roles in achieving the desired selectivity. While two primary radicals are concomitantly produced, the nucleophilic radical is selectively sequestered by the metal and the electrophilic radical preferentially adds onto an alkene (Figure 2F). The latter event produces a third nucleophilic and bulky tertiary radical that efficiently engages in S_H_2 with the alkyl metal. This strategically orchestrated reactivity enabled the controlled functionalization of complex molecules (**11**).^79^ The platform was also extended to include the difunctionalization of olefins, incorporating both electrophilic *N*-centered and primary alkyl radicals.^84^

Inspired by these advances, Zhang developed a Ni^II^(scorpionate)-catalyzed decarboxylative monofluoroalkylation strategy (**12**),^85^ which highlighted the strategic utility of S_H_2 in sorting fluorinated radicals. Similarly, the Li group demonstrated direct trifluoromethylation of alkyl bromides using a stoichiometric Grushin reagent bpyCu^III^(CF_3_)_3_ (**13**).^86^ Mechanistic studies revealed that the reactive species is not the parent Cu^III^ complex, but rather an *in situ*-generated bpyCu^II^(CF_3_)_2_ that undergoes S_H_2 with the alkyl radical.

Zhang and co-workers have also bridged S_H_2 and metalloradical catalysis (MRC), leveraging Co^II^- and Fe^III^(porphyrin) catalysts (e.g., **Co-1**, Figure 2A) to achieve enantioselective cyclopropanation. This strategy proceeds via Co^III^-carbene radical intermediate, which adds to various activated alkenes, followed by an intramolecular S_H_2 step (Figure 2F).^87,88^ Their strategic variation of carbene and olefins has yielded a broad scope of chiral cyclopropanes,^89,90^ including α-amino acid- (**14**),^57^ heteroaryl-(**15**),^58^ malonyl-(**16**),^91^ and fluorinated-(**17**)^92^ derivatives. Notably, S_H_2 reactivity was also extended to C­(sp^2^) centers for enantioselective synthesis of cyclopropenes (**18**).^93,94^ They also demonstrated the ability of the Co^III^-carbene radical intermediate to initiate HAT, and developed several intramolecular HAT-S_H_2 cascades forming heterocycles via stabilized radicals (**19**).^95–97^

Recently, Yang’s group developed a triple cooperative catalytic system involving photoredox, S_H_2, and chiral amine catalysis for the enantioselective construction of quaternary carbon centers α to carbonyls.^98^ In this transformation, a ketone and a chiral amine catalyst are condensed to form an enamine, which undergoes SET oxidation, leading to an α-iminyl radical (Figure 2C). Stereoselective S_H_2 with an alkyl Fe^III^(porphyrin) intermediate then forges congested quaternary centers with high enantioselectivity (**20**).

Subsequently, Baran and co-workers introduced a stereoretentive radical cross-coupling platform between enantiopure sulfonyl hydrazides and heteroaryl halides (**21**).^99^ Mechanistic studies revealed an inner-sphere rebound event involving homolytic substitution at a C­(sp^2^) center. Diao and Hoffmann have since elucidated this process in detail, showing that ligand, steric, and electronic effects significantly influence the kinetics of this process, which was described as a concerted radical capture and C–C bond formation.^100^

The S_H_2 manifold has also found interest in biocatalysis, where Fasan, Zhang, and co-workers implemented engineered Fe^II^-based cytochrome P450 “carbene transferases” to enable regio- and enantioselective alkylation at the α-C–H (**22**), β-C–H and even N–Me positions of *N*-methylindolines.^101^

Together, these studies emphasize the remarkable advancements in S_H_2 catalysis, showcasing its versatility across various radical coupling methods. The strategic combination of S_H_2 with photoredox, enzymatic, and transition metal catalysis has firmly established it as a powerful and universal platform for C–C bond formation.

### C–X Bond Formation by RLT Catalysis

Cytochrome P450 enzymes, emblematic of the HAT-RLT mechanism, have captivated many researchers and have been the subject of extensive investigation aimed at understanding and harnessing their unique reactivity.^33,34,41,102^ Engineered halogenases demonstrated promising results in the radical transfer of various functional groups (FGs); including OH, NO_2_, N_3_, Cl, Br, and NCO.^103–106^ While Kochi’s pioneering work laid the conceptual foundation for the synthetic application of RLT with many of these and other ligands,^2,27,107^ and despite recent advances in RLT chemistry, significant progress is still required to convert the strategy into a more general tool for organic synthesis.

### C–O Bond Formation

In a seminal contribution, the Goldberg group reported the first isolable and well-characterized analogue of the elusive Cpd-II species (Figure 1B), an Fe^IV^(OH)­(ttppc) corrole complex.^108^ Their studies revealed the concerted nature of the OH-transfer step as opposed to a stepwise electron-transfer/ion transfer mechanism. Paria and Moonshiram reported a Co^III^(OH) complex supported by a bis-amidate-bis-alkoxide scaffold that could transfer its OH ligand to trityl radicals.^109^

Despite these advances and key contributions by Hirobe on decarboxylative hydroxylation reactions mediated by Fe­(TPFPP)Cl ­(Figure 5, **23**),^106^ the formation of C–O bonds via the RLT remains limited mainly to hydroxylation, and a general strategy for the transfer of OR groups remains elusive.^30,41,110,111^

**Figure 4:**
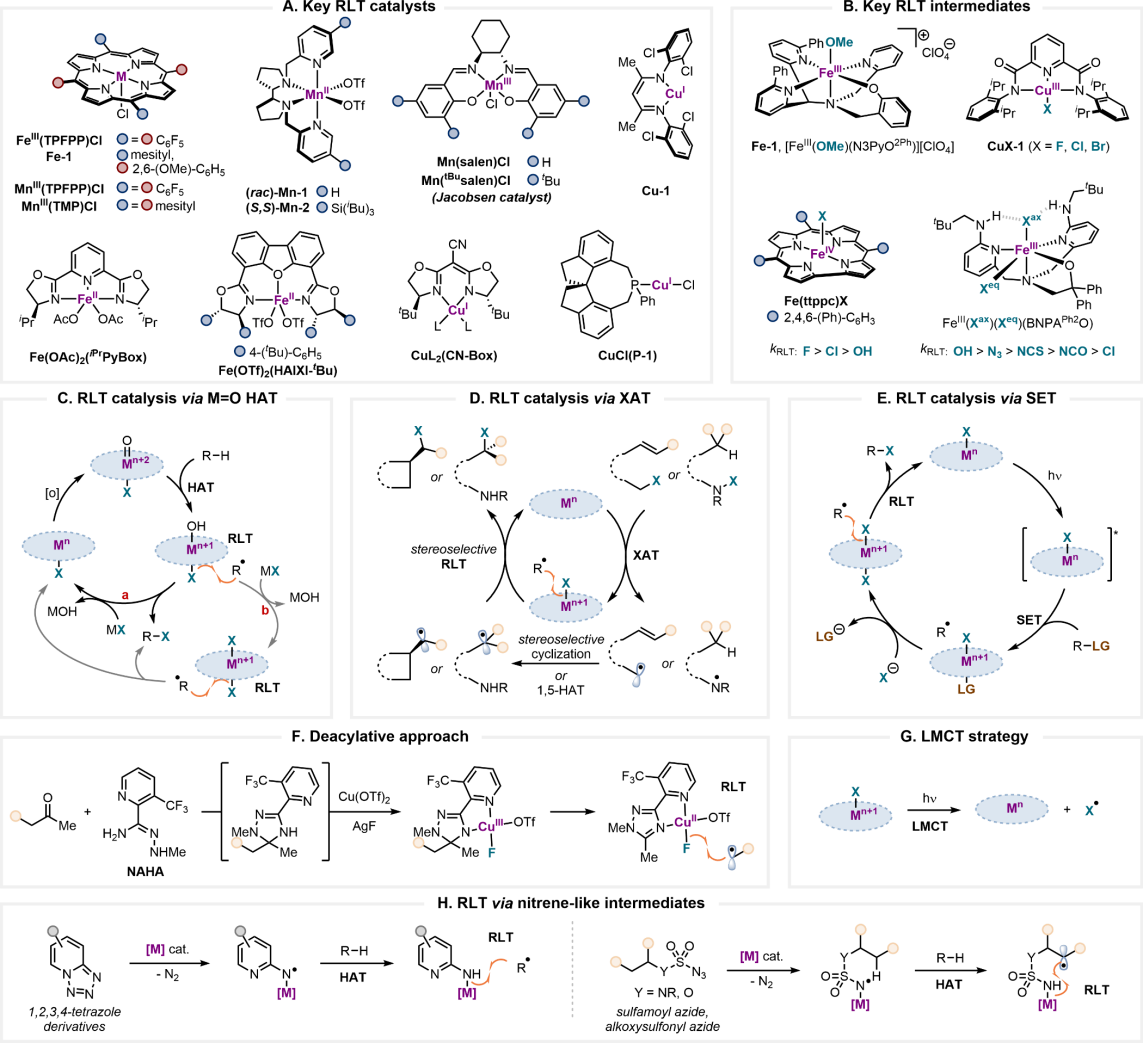
**A.** Selected RLT catalysts. **B.** Selected RLT intermediates. **C.** RLT catalytic cycle via M=O mediated HAT. **D.** RLT catalytic cycle via XAT. **E.** RLT catalytic cycle via SET. **F.** RLT in deacylative fluorination approach. **G.** Formation of radicals via LMCT approach. **H.** RLT via nitrene-like intermediates.

**Figure 5:**
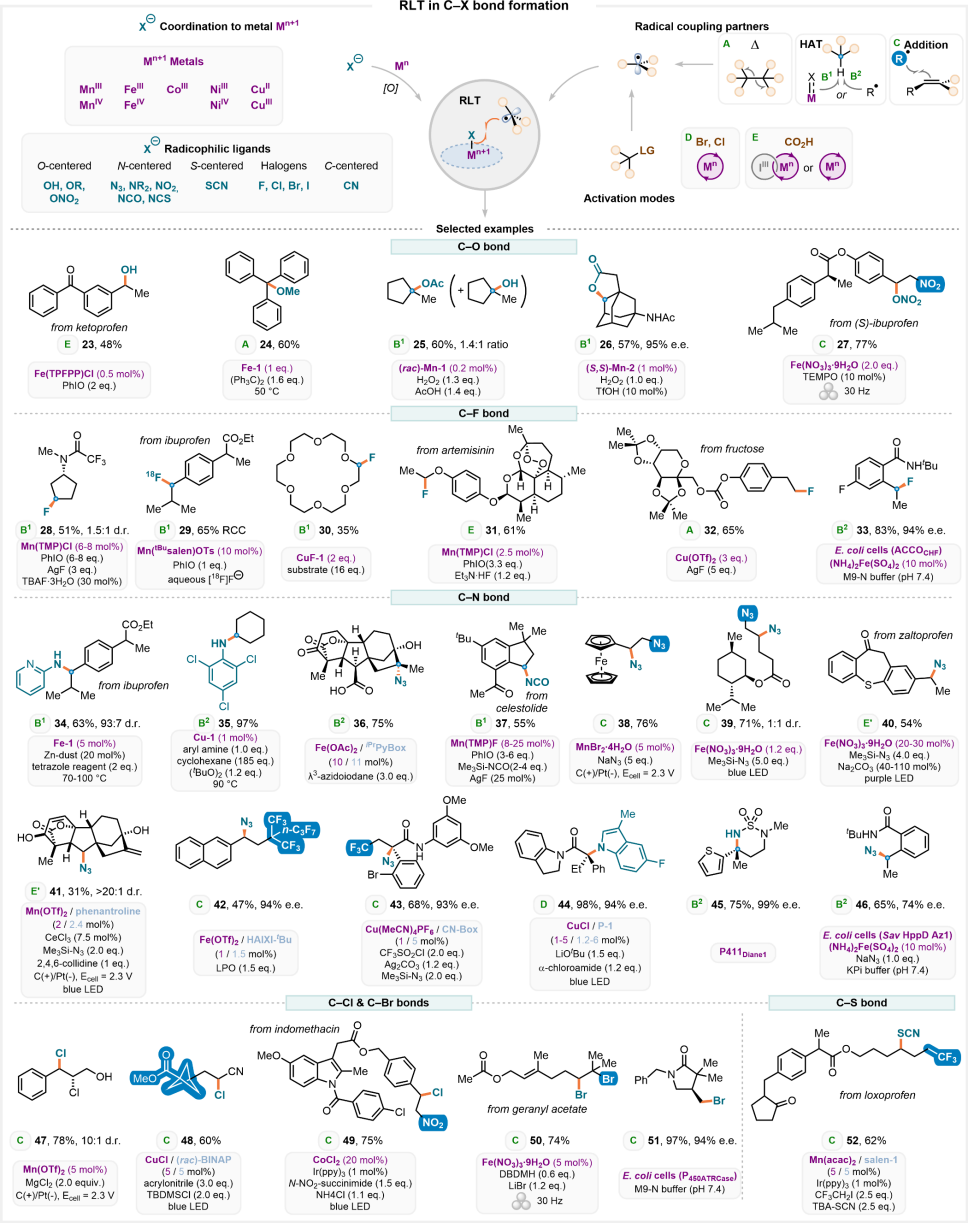
RLT chemistry in C–X bond construction. Selected examples of RLT-mediated synthesis of complex targets are represented alongside key reaction conditions. The activation strategies used for the generation of the two radical partners (A to E) are given below the molecular structure as green letters.

One rare instance of alkoxy RLT was reported by Goldberg, Jameson, and co-workers, demonstrating that complex **Fe-1** (Figure 4B) can shuttle a methoxy group to trityl radicals, resulting in the formation of methyl ethers (**24**).^112^ Addressing another challenge, Bryliakov showcased that complex **(** *rac* **)-Mn-1** promotes both hydroxylation and acetoxylation of C–H bonds (**25**) in the presence of hydrogen peroxide and acetic acid (Figure 4A, C, *path a*).^113,114^ Building on this, Costas, Bietti, and co-workers developed an enantioselective lactonization of C–H bonds of carboxylic acids (**26**) employing complex **(** *S,S* **)-Mn-2** (Figure 4A).^115^ Furthermore, this catalytic platform could mediate the transfer of various acetoxy groups including fluorinated alcohols such as trifluoroethanol. Mechanistic investigations suggested that alternative pathways, like oxidation to carbenium ion intermediates, may also be operative.^116–118^

Beyond defined ligand frameworks, Katayev and co-workers uncovered an RLT manifold using simple Fe^III^(NO_3_)_3_·9H_2_O in the mechanochemical nitrative difunctionalization of alkenes (**27**).^119^ In this transformation, TEMPO-mediated reduction of Fe^III^ to Fe^II^ triggers the release of free ^•^NO_2_ radicals, which add to alkenes to generate *C*-centered radicals. These are captured via the transfer of an ONO_2_ group from ferric nitrate, forming R–ONO_2_ alkyl nitrates.

### C–F Bond Formation

A major breakthrough in the halogenation of C–H bonds was made by the Groves group, who developed a method for the direct fluorination of alkanes, using a Mn^III^(TMP)Cl as HAT-RLT catalytic system (Figure 4A), with AgF and iodosylbenzene as the fluorine source and stoichiometric oxidant, respectively (**28**).^31,120^ The RLT step with the alkyl radical was shown to occur from a key *trans*-Mn^IV^(F)_2_(TMP) reagent (Figure 4C, *path b*). DFT studies supported the superior reactivity of the Mn^IV^F_2_ compared to the Mn^IV^(OH)­(F) intermediate. Recently, the McDonald group proposed the formation of a related Fe^IV^(F_2_) species in the fluorination of activated C–H bonds.^121^ Extension to benzylic C–H fluorination was achieved using the Jacobsen catalyst (Figure 4A), which exhibited greater selectivity for fluorine over hydroxyl transfer.^122^ Application of this concept to radiofluorination with [^18^F] source proved possible with high radiochemical conversion and applied to a range of drug derivatives (**29**).^123,124^

Following a similar concept, Stieber, Zhang, and co-workers demonstrated the high reactivity of a series of **CuX-1** (X = F, Cl, Br) complexes in the halogenation of radicals (Figure 4B).^125^ Computational studies indicated that these complexes exhibit radical character, best described as Cu^II^(X^•^)—i.e., where a halogen radical is coordinated to a Cu^II^ center. This radical nature was successfully exploited in the C–H fluorination via the HAT-RLT process (**30**).

Building on prior decarboxylative hydroxylation,^106^ Groves introduced a decarboxylative fluorination using Mn^III^(TMP)Cl (**31**).^126^ DFT investigations revealed that decarboxylation involves a synchronous F transfer from Iodine to the Mn^III^ center, which produces the transient carboxyl radical and Mn^IV^(F)_2_-porphyrin, realizing RLT fluorination in a subsequent step.

Recently, Dong and co-workers developed a deacylative fluorination strategy (**32**) employing *N*′-alkyl-hydrazonamide (NAHA) reagents, superstoichiometric Cu^II^(OTf)_2,_ and excess AgF, which serves as both the oxidant and the F source (Figure 4F).^127^ Detailed DFT studies suggest that primary radicals preferentially undergo RLT with a Cu^II^(F) species, while secondary radicals seem to form Cu^III^(R)­(F) intermediates that then react via S_N_2-type substitution with fluoride anions.

A significant milestone was achieved by the groups of Yang and Liu, who reported a biocatalytic radical relay strategy for the enantioselective fluorination of C–H bonds through a XAT-HAT-RLT cascade (Figure 4D).^128^ This remarkable feat was made possible through the engineering of an iron metalloenzyme, involving an enantioselective RLT from an Fe^III^F species, forging a highly challenging chiral C–F bond (**33**).

### C–N Bond Formation

Inspired by the HAT-RLT paradigm established by CP450 enzymes for OH-transfer, we extensively investigated the extension of this concept to *N*-based group transfer has been extensively investigated.

In early studies, Cenini and co-workers screened a broad range of porphyrin-based TM-complexes, including first row metals (Ti, V, Cr, Mn, Fe, Co, Ni, Cu, Zn) as well as Rh, Ru and Pd, for allylic amination of cyclohexene using aryl azides.^129,130^ Only Co and Ru showed notable activity, underscoring the difficulty of this transformation. de Bruin and Zhang demonstrated further the formation of a Co^III^(N^•^R) nitrene radical species, consistent with a HAT-RLT pathway.^131,132^

Chattopadhyay and co-workers reported an amination of benzylic C–H bonds (**34)** catalyzed by Fe-porphyrin complex **Fe-1** (Figure 4A).^133^ In this work, a key Fe^III^(N^•^Py) species, produced by decomposition of a tetrazole precursor, is responsible for HAT followed by RLT with the resulting radical (Figure 4H, left). Notably, Fe^III^(N^•^Ar),^134^ and Fe^V^(NTs)^135–137^ species have been reported to similarly exhibit HAT-RLT behavior. While the related Cu^II^(NHAr) complex does not engage in HAT, it was shown to readily transfer an anilide ligand to alkyl radicals (**35**).^138^

In 2015, Hartwig and co-workers reported the azidation of tertiary C–H bonds using the *in situ* generated Fe­(OAc)_2_(^*i*Pr^PyBox) complex and a λ^3^-azidoiodane reagent (Figure 4A). This strategy, was shown to involve RLT between key Fe^III^(N_3_) and alkyl radicals, proved versatile and amenable to late-stage C–H azidation (**36**).^139,140^ A similar strategy was described by Groves using Mn^III^(porphyrin) and Mn^III^(salen) catalysts in combination with sodium azide and iodosobenzene.^141,142^ The HAT-RLT reactivity was shown to be enabled by a key Mn^V^(=O)­(N_3_) species, following the general mechanism depicted in Figure 4C. Notably, DFT studies revealed that the reaction occurred preferably at the less congested terminal N^3^ atom of the azido moiety (Mn—N^1^=N^2^=N^3^). This strategy was later extended to isocyanation reactions using Me_3_Si–NCO reagent as an organic-soluble isocyanate source (**37**).^143^

In 2017, Lin and co-workers combined RLT-catalysis and electrochemistry to achieve vicinal diazidation of alkenes, and *in situ*-generated Mn^III^(N_3_)­X_2_ complex was shown to engage in RLT (**38**).^32^ Under these conditions, an azidyl radical is formed by anodic oxidation, which adds to the alkene substrate. The resulting alkyl radical subsequently engages in a RLT step with a Mn^III^(N_3_) species. Following this seminal contribution, diazidation protocols were independently developed by West^144^ and Shi^145^ using photochemical conditions, leveraging photoinduced ligand-to-metal charge transfer (LMCT) activation (Figure 4G) of Fe^III^(N_3_)­X_2_ and RLT paradigms (**39**). West’s group later applied this LMCT-RLT strategy to the decarboxylative azidation of carboxylic acids using catalytic Fe­(NO_3_)_3_·9H_2_O and Me_3_Si–N_3_ as the azide source (**40**).^146^ They also reported a C–H azidation of alkanes using Selectfluor as both an HAT agent and oxidant.^147^ A complementary decarboxylative azidation approach was developed by the Fu group, merging electrophotocatalysis and RLT catalysis. They leveraged the photoactivity of Ce^IV^ species to promote decarboxylation through LMCT mechanism (Figure 4G), with the ability of Mn^III^ species to undergo RLT. While starting from Mn^II^ and Ce^III^ catalysts, they relied on electrochemistry to anodically oxidize these species and unlock the desired reactivity (**41**).^148^

Bao’s group reported that Fe^II^(OTf)_2_ and Fe^III^(OTf)_3_ exhibit remarkable activity in catalytic carboazidation of alkenes and alkynes.^149^ West’s group reported a related process under photochemical conditions.^150^ Furthermore, by designing chiral ligands promoting weak interactions, Bao and Zhang developed asymmetric carboazidation and aminoazidation of alkenes (**42**) using Fe­(OTf)_2_(HAIXI-^*t*^Bu) complex (Figure 4A).^151,152^ Notably, Feng^153,154^ and Liu^155^ have also developed a series of enantioselective carboazidations of Michael acceptors (**43**).

A photochemical solution for the asymmetric C–N bond cross-coupling between α-chloroamides and indoles or carbazoles was reported by Fu and Peters’ groups (**44**) using generated chiral **CuCl­(P-1)** complex (Figure 4A).^156^ In this system, photoexcitation of an *in situ* generated chiral Cu^I^NR_2_(P-1) complex facilitated SET reduction of the α-chloroamides, followed by enantioselective RLT with the Cu^II^(NR_2_)_2_ species (Figure 4E).^157^ This concept was successfully extended to the cross-coupling of aniline and amides with various electrophiles.^158–160^

In 2019, Arnold, Liu, and co-workers engineered a P411 metalloenzyme that mimics the HAT-RLT reactivity of CP450. This Fe­(porphyrin)-based system enabled the enantioselective amination of C–H bonds to produce chiral 1,2-diamines (**45**) from sulfamoyl azides (Figure 4H, right).^161,162^ Very recently, the Zhang group achieved a related Co^II^-catalyzed enantioselective amination from alkoxysulfonyl azide.^163^ In a collaborative effort, the teams led by Huang, Garcia-Borràs, and Guo engineered a Fe^II^-based enzyme through directed evolution in *E. coli* to catalyze the enantioselective azidation of benzylic C–H bonds (**46**) from *N*-fluoroamides, following a XAT-HAT-RLT cascade (Figure 4D).^164^

### C–Cl and C–Br Bond Formation

Since the seminal report by Kochi,^27^ and more recently by Groves,^165–167^ several methodologies involving the ligand transfer of halogens have been developed.

For example, the Lin group reported an electrocatalytic vicinal dichlorination of alkenes catalyzed by Mn­(OTf)_2_ using MgCl_2_ as the chlorine source. After ligand exchange and anodic oxidation, key Mn^III^–Cl species were formed, which served both as Cl-atom transfer agents and as RLT partners (**47**).^168^ Additionally, building on earlier work by Wan,^169^ several LMCT-RLT systems were reported for the alkene dichlorination by West (Fe­(NO_3_)_3_·9H_2_O-NaCl),^170^ König (FeCl_3_–LiCl),^171^ and Feng (TBADT-FeCl_3_).^172^ Besides dihalogenation processes, West’s group has reported a chloro-fluoroalkylation of alkenes, using Mn^III^(salen) catalysts.^150^

More recently, Ritter and co-workers addressed a longstanding challenge in radical Kharasch additions,^173–175^ while taking careful consideration of the radical philicity^81,83,176^ of key radical intermediates (**48**).^177^ They demonstrated how electronic modulation of the ligand at the metal enables fine-tuning of the RLT kinetic reaction. Indeed, they employed CuCl equipped with an electron-rich (*rac*)-BINAP ligand to suppress the RLT event between CuX_2_(BINAP) and the nucleophilic alkyl radical (produced through SET reduction of a RAE by photoexcited *Cu^I^X-BINAP, akin to Figure 4E). This favors radical addition to electron-deficient alkenes, with the formation of a new electrophilic radical which, in turn, reacts with enhanced rate through RLT with the rich CuX_2_-BINAP.

Recently, our group has shown that ligand-free CoCl_2_ and CoBr_2_ can serve as efficient RLT catalysts in the photocatalyzed nitrative halogenation of alkenes, using NH_4_X (X = Cl^–^, Br^–^) as the halogen source (**49**).^178^ We then demonstrated that RLT paradigm remained operative under mechanochemical conditions.^179^ By changing the halogen sources, it was possible to selectively achieve bromochlorination, dibromination, and dichlorination of alkenes under ball milling conditions (**50**).

Despite these advances, enantioselective halogenation via RLT remains a challenge. A notable step forward was achieved by Yang and co-workers (**51**), who repurposed CP450 to catalyze enantioselective atom transfer radical cyclizations (ATRCs) through a XAT-cyclization-RLT cascade (Figure 4D).^180^

### C–S Bond Formation

Although radical homolytic substitutions involving sulfur atoms are well established in the literature,^181^ instances of *S*-based ligand transfer are scarce.

A representative example has been reported by the West and Wang groups, who developed a range of thiocyanato-fluoroalkylation reactions through the merger of photoredox and RLT catalysis using Mn^III^(salen) complexes (**52**).^150^

### Reactivity and Ligand Selectivity in RLT

Beyond bond construction, the identity of the transferable ligand plays a pivotal role in dictating both the rate and the selectivity of the RLT process. To elucidate this influence, several comparative studies have been carried out across the literature.

Goldberg’s group reported a series of corrole-based Fe^IV^(X) complexes, where X = F, Cl, and OH (Figure 4B);^108,182^ and kinetic analysis with trityl radicals showed that fluorine transfer is ∼86 times faster than the chlorine and ∼313 times faster than hydroxyl transfer in this system.

They also prepared a series of FeX_2_(BNPA^Ph2^O) complexes,^183^ and by varying the X (OH, N_3_, NCO, NCS, Cl), examined their reactivity with trityl radicals, revealing a clear selectivity trend with the following order: OH > N_3_ > NCS > NCO > Cl, with hydroxyl transfer being the most favored. Notably, this trend contrasts with earlier findings on high-valent Fe^IV^(X)­(ttppc) complexes, where Cl outpaces OH transfer, highlighting that oxidation state and ligand scaffold influence RLT behavior.^182^ Interestingly, the position of the X ligand within the complex (equatorial or axial) was found to have little effect on selectivity, therefore supporting the idea that the ligand identity, rather than its coordination site on the complex, preferentially dictates RLT selectivity.^183,184^ Furthermore, alteration of the secondary coordination sphere of the ligand (through the removal of the amino substituents to suppress the hydrogen bonding interaction with X^ax^) was found to also have only minimal influence. Interestingly, the selectivity outcome of the ligand transfer in Fe^III^(OH)­(N_3_)­(BNPA^Ph2^O) was found to be greatly influenced by the electronic properties of the reaction partner, as electron-rich (*p*-OMe-C_6_H_4_)_3_C^•^ radical furnished a mixture of azidation and hydroxylation products.^185^

## 3 Homolytic Substitution Ability

Recent work by Goldberg has suggested that the thermodynamic driving force of the RLT step accounts for the observed selectivity.^183^ Additionally, straightforward computational approaches (such as the calculation of bond dissociation free energies) have shown strong correlation with the homolytic substitution reactivity of metal complexes.^66^ Motivated by these insights and the growing momentum in S_H_2/RLT strategies, we examined the thermodynamic reactivity trends across a series of metal complexes (Figure 6).^186^ Our analysis focused on first row TMs,^187,188^ whose abundance, versatile reactivity, and improved sustainability relative to their heavier congeners make them a compelling platform for exploring and advancing S_H_2/RLT transformations.

**Figure 6:**
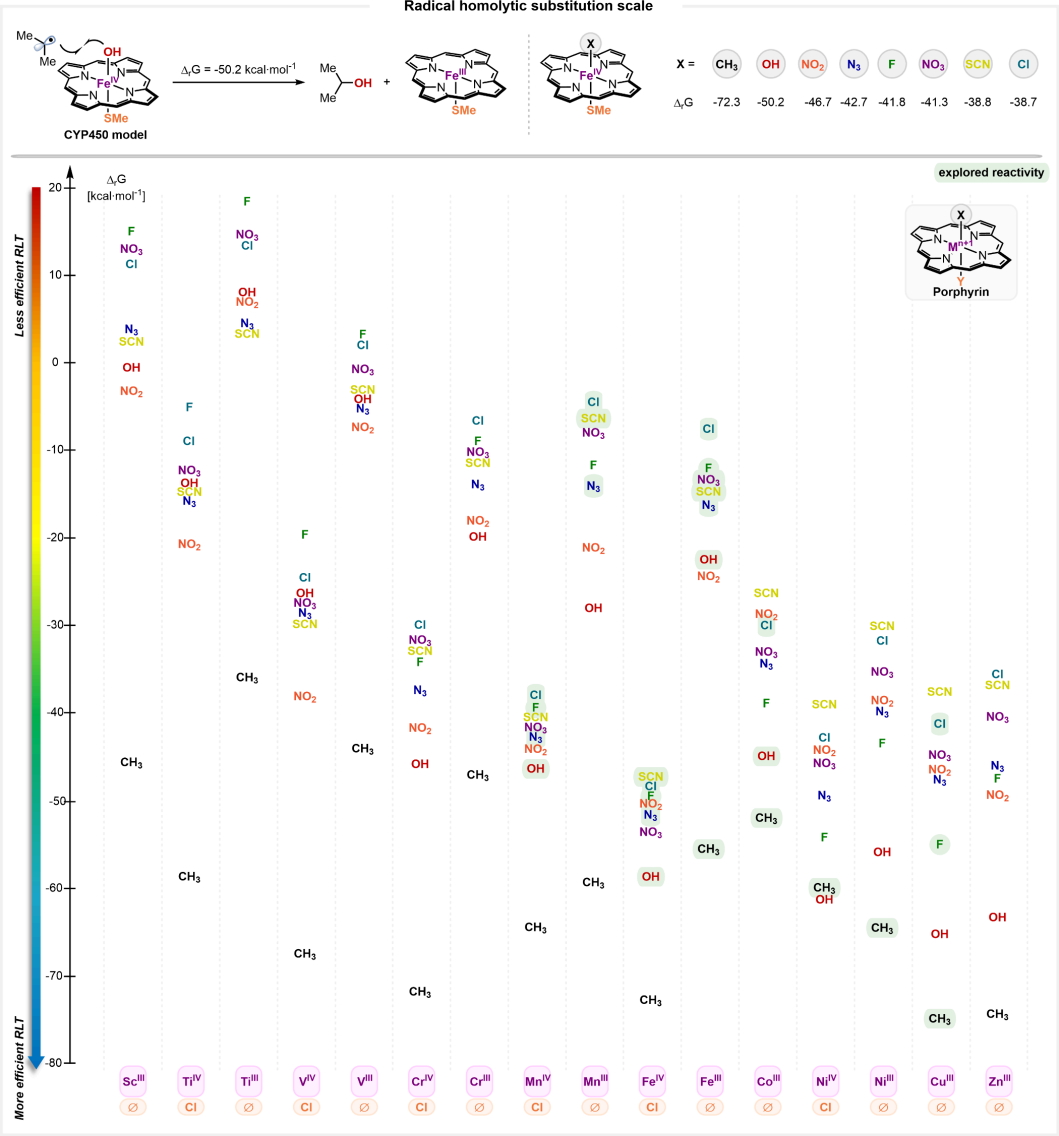
Radical homolytic substitution scale involving 1st row TM. Representative of the thermodynamic driving force associated with the S_H_2/RLT event. Computed at the (U)­M06-L-D3/Def2-TZVP,(MeCN)//(U)­M06-L-D3/Def2-SVP,(MeCN). Experimentally explored reactivity highlighted in green. (See SI for an analogous scale based on BOX-based metal complexes).

We first focused on the canonical cytochrome P450 iron porphyrin complex, **cpd-II**, and computed the Gibbs free energy for the radical transfer of the hydroxyl group to an isopropyl radical (Δ_*r*_ *G*_(OH)_ = −50.2 kcal·mol^–1^, Figure 6, see SI for details). These calculations revealed that Me-transfer is significantly more favorable (Δ_*r*_ *G*_(Me)_ = −72.3 kcal·mol^–1^), while the other heteroatomic ligands are less favored and are clustered together (from Δ_*r*_ *G*_(NO2)_ = −46.7 to Δ_*r*_ *G*_(Cl)_ = −38.7 kcal·mol^–1^). These thermodynamic profiles offer valuable insight into the relative driving force inherent to the transfer of these ligands and enable the extrapolation of the chemical reactivity of a given metal complex. The notably large exergonicity of C-transfer compared to X-transfer indicates that the M–C bonds are significantly weaker than the M–X bonds (see SI for computed BDEs).

We then assessed how the thermodynamic driving force for ligand transfer changes across the first row TM series. Interestingly, a general trend emerges: S_H_2/RLT becomes increasingly favorable when progressing from Sc to Zn across the periodic table. It is important to note at this point that Sc^III^ is not a redox-active metal and is thus expected to be inefficient in ligand transfer, as the reduced Sc^II^ species would be highly unstable. This is very well captured by the scale, which positions Sc^III^ as one of the least reactive metals of the series. Although Zn^II^ is a redox-inactive metal, its redox state of +II within the porphyrin complex prevented us from probing its RLT ability. We therefore also computed these thermodynamic values for all first row TMs equipped with the ubiquitous BOX^189^ ligand scaffold and found a similar trend to that observed in porphyrin complexes (see SI). Notably, the elusive Zn^III^ has drawn significant attention, inspiring efforts to study Zn^III^-based species.^190,191^ Calculations on hypothetical Zn^III^(X)-porphyrin complexes suggest exceptionally high reactivity, placing them among the most reactive species in the series, according to the thermodynamic scale.

Finally, these theoretical studies illustrate that the thermodynamic driving force for S_H_2/RLT increases with the oxidation state of the metal, consistent with the weaker metal–ligand bonds observed in highly oxidized species. This aligns well with the literature evidence that radical transfer of heteroatom-based groups generally involves metals in their higher oxidation state (e.g., Mn^IV^, Fe^IV^) compared to carbon-based groups (e.g., Fe^III^, Ni^II^), likely required to weaken the stronger M–X bonds and unable reactivity.

Although this scale is rooted in thermodynamics and is not intended to precisely predict reaction kinetics, it provides a valuable comparative and systematic framework for understanding radical homolytic substitution across different first row TM values in porphyrin and BOX ligands.

## 4 Future Directions

Radical ligand transfer and bimolecular homolytic substitution paradigms are redefining the landscape of radical chemistry by offering streamlined, selective approaches to C–C bond formation. These outer-sphere mechanisms—rooted in fundamental organometallic and biological paradigms—are increasingly recognized not as isolated curiosities but as foundational principles for the next generation of radical-based transformations.

While literature reports have provided qualitative and quantitative correlations between ligand features and selectivity, systematic studies dissecting the influence of radical stability, metal oxidation state, ancillary ligand architecture, and solvent effects remain lacking. Similarly, the structural and electronic features of ancillary ligands (e.g., donor type, bite angle, rigidity, and buried volume) that enable optimal radical reactivity are poorly understood, despite clear evidence that certain ligand–metal combinations outperform others.

Steric parameters are also known to influence S_H_2 and RLT mechanisms; however, studies deconvoluting the specific effects of metal–ligand combinations on catalytic efficiency are scarce. Addressing these knowledge gaps will be critical for establishing predictive design principles and guiding the rational development of new catalytic radical transformations.

Despite notable successes, particularly with iron, nickel, cobalt, manganese, and copper complexes, the broader potential of RLT and S_H_2 remains largely untapped. Challenges persist in controlling site selectivity, ligand transfer selectivity, and enantioselectivity, under either stoichiometric or catalytic conditions. This underscores the need for deeper exploration, particularly into the role of redox-active ligands, metal-centered oxidation state/electronic tuning, and radical philicity.

In this Outlook, we have outlined how modern reactivity principles and radical thermodynamic analysis are beginning to bridge these gaps. Our computational insights reveal distinct energetic preferences for radical transfer across first row TM complexes, offering a roadmap for future ligand and metal selection.

Looking forward, there is a compelling need to expand the scope of metals beyond conventional systems. Underexplored elements such as chromium^192–194^ and vanadium may enable divergent reactivity profiles and unlock orthogonal selectivity, particularly when coupled with emerging reaction manifolds, such as photochemistry, electrochemistry, biochemistry, or even mechanochemistry. Expansion of the transferable ligands and development of general, robust, and efficient synthetic protocols—targeting, for example, Csp,^2^ Csp, CN, or other heavier heteroelement moieties^46,181^ (e.g., Si, P, S, Ge, Se)—remain key objectives for advancing this field. Ultimately, as the field matures, these S_H_2/RLT principles are poised to move from conceptual novelty to central strategy driving innovation in catalysis, materials science, and even biochemistry.

We anticipate that exploration of novel reactivity modes rooted in RLT principles—such as “umpolung RLT” reactivity, where the addition of a radical to a metal-bound L-type ligand induces its transformation into an X-type ligand while concurrently oxidizing the metal center (R^•^ + M^*n*^–L → M^*n*+1^–X)—will open a new avenue for bond construction, as recently demonstrated by Zuo and Liu in the iron-catalyzed carbonylation of methane.^195^ Contemporary dehydrogenative^196^ and deconstructive approaches^197,198^ also appear attractive, as their translation to C–F bond cleavage would help address a critical societal challenge through the destruction of per- and polyfluoroalkyl substances (PFAS).^199,200^ Harnessing these principles may enable new classes of radical transformations that were previously inaccessible through classical RLT logic.

By embracing these expanded mechanistic paradigms and leveraging modern computational and synthetic tools, RLT and S_H_2 chemistry can evolve into truly transformative platforms for selective, catalytic, and sustainable radical-mediated molecular construction across diverse chemical domains.

## Supplementary Material



## Notes

Computational details and methods, calculated energies, and Cartesian geometries of optimized structures (PDF)

## Abbreviations

ttppc: 5,10,15-tris­(2,4,6-triphenylphenyl)­corrolato^3–^
BNPA^Ph2^O: 2-(bis­((6-(neopentylamino)­pyridin-2-yl) methyl)­amino)-1,1-diphenylethanolate
pdp: *N*,*N*′-bis­(2-pyridylmethyl)-2,2′-bipyrrolidine
DBFOX/Ar: (*R*,*R*)-4,6-dibenzofurandiyl-2,2′-bis­(4-aryloxazoline)
Troc: 2,2,2-trichloroethoxycarbonyl
NTB: tris­(2benzimidazoylmethyl)­amine
BOX: bis­(oxazolines)
TXO: thioxanthone
Mes: mesityl
BTMG: 2-*tert*-butyl-1,1,3,3-tetramethylguanidine
TBAB: tetrabutylammonium bromide
TMDS: tetramethyldisiloxane
PMP: pempidine
LPO: lauroyl peroxide
TBADT: tetrabutylammonium decatungstate
DBDMH: 1,3-dibromo-5,5-dimethylhydantoin.
