# Supplementary material for: Bimolecular Homolytic Substitution (SH2) and Radical Ligand Transfer (RLT): Emerging Paradigms in Radical Transformations
Source: ACS Cent Sci. 2025 Sep 13;11(10):1812–27. doi: 10.1021/acscentsci.5c01091 (PMC12550624; doi:10.1021/acscentsci.5c01091)
Supplement: Supplementary file 1 [file oc5c01091_si_001.pdf]

Supporting Information

Bimolecular Homolytic Substitution (S_H2) and Radical Ligand Transfer (RLT): Emerging Paradigms in Radical Transformations

Anthony J. Fernandes,* Dmitry Katayev*

Department of Chemistry, Biochemistry and Pharmaceutical Sciences, University of Bern, Freiestrasse 3, 3012 Bern, Switzerland

*Corresponding authors

E-mail: anthony.fernandes@unibe.ch, dmitry.katayev@unibe.ch

Table of Contents

1. Computational details -- 3

1.1. Computational methods -- 3

1.2. Computed energies --- 4

1.2.1. Computed energies of porphyrin complexes --- 5

1.2.1. Computed energies of bis(oxazoline) complexes ------------------------------------ 19

1.3. Computed structures -- 33

1.3.1. Porphyrin complexes -- 34

1.3.2. BOX complexes -- 93

1.4. References --- 150

1. Computational details

1.1. Computational methods

The DFT calculations have been performed with the Gaussian 9 program package.[1]

The conformational space of all molecules has been initially searched using meta-dynamics simulations based on tight-binding quantum chemical calculations as implemented in the software package Conformer-Rotamer Ensemble Sampling Tool CREST.[2]

The structures located with CREST have been subjected to geometry optimization using (U)M06-L functional[3] with Def2-SVP basis set,[4, 5] including D3 dispersion correction[6] and polarizable continuum model (PCM)[7] with SMD parameters[8] to consider solvent effects (SMD parameters of acetonitrile are available in the used software package). The nature of all stationary points was verified through the computation of the vibrational frequencies. Single point (SP) energies from these geometries were calculated at the (U)M06-L/Def2-TZVP level of theory, including D3 dispersion correction and SMD solvation model (SMD parameters of acetonitrile are available in the used software package). The thermal corrections to the Gibbs free energies were combined with the single-point energies to yield Gibbs free energies (ΔG) at 298.15 K. All energies are reported in kcal·mol^{-1} unless otherwise stated.

1.2. Computed energies

M06-L functional was recommended by Peverati *et al.* who conducted an extensive benchmark of 250 functional methods for the description of spin states and binding properties of first-row transition metal porphyrins.[9] M06-L ranks among the best-performing functionals, and was recommended for future studies. In our work dealing with the calculation of binding energies of first-row transition metal porphyrin and BOX ligand scaffolds, we employed this recommended method.

NO_2 is an ambivalent ligand and can coordinate through either M–NO_2 or M–ONO binding modes. Both coordination modes were considered, and the values corresponding to the less favorable complexation are shown in grey in the tables below. The associated ligand transfer reactions and bond dissociation energies (BDEs) were calculated assuming the formation of $^{i}PrNO_2$, which is more stable than ^{i}PrONO.

Table S1. Energy of small molecules computed energies at the (U)M06-L-D3/def2-TZVP,SMD(MeCN)//(U)M06-L-D3/def2-SVP,SMD(MeCN). Energies in Hartree unless otherwise stated (BDFE and BDE are given in kcal·mol^{-1}). Mult: multiplicity.

Compounds	Mult	Thermal correction		SP energy	*ΔG*	*ΔH*	BDFE	BDE
		to *H*	to *G*					
iPr-OH	1	0.114271	0.080802	-194.4078303	-194.3270283	-194.2935593	74.1	86.6
iPr-Cl	1	0.100553	0.066383	-578.7811239	-578.7147409	-578.6805709	72.9	83.6
iPr-F	1	0.10154	0.068494	-218.438818	-218.370324	-218.337278	100.3	111.2
iPr-N3	1	0.114926	0.076693	-282.8106167	-282.7339237	-282.6956907	53.5	66.4
iPr-SCN	1	0.111683	0.071346	-609.6335187	-609.5621727	-609.5218357	51.3	63.8
iPr-ONO2	1	0.119193	0.07905	-398.9487066	-398.8696566	-398.8295136	57.6	72.5
iPr-Me	1	0.137492	0.10321	-158.4895106	-158.3863006	-158.3520186	69.0	83.8
iPr-NO2	1	0.1142	0.076279	-323.7454763	-323.6691973	-323.6312763	41.8	55.9
iPr-ONO	1	0.112527	0.074062	-323.7339157	-323.6598537	-323.6213887	35.9	49.7
iPr-rad	2	0.093742	0.060601	-118.5029351	-118.4423341	-118.4091931		
OH-rad	2	0.011738	-0.008501	-75.75813453	-75.76663553	-75.74639653		
Cl-rad	2	0.00236	-0.015677	-460.140508	-460.156185	-460.138148		
F-rad	2	0.00236	-0.014813	-99.75329171	-99.76810471	-99.75093171		
N3-rad	2	0.013663	-0.01195	-164.1943096	-164.2062596	-164.1806466		
SCN-rad	2	0.012185	-0.015029	-491.023124	-491.038153	-491.010939		

Compounds	Mult	Thermal correction		SP energy	*ΔG*	*ΔH*	BDFE	BDE
		to *H*	to *G*					
ONO2-rad	2	0.016763	-0.013966	-280.3215955	-280.3355615	-280.3048325		
Me-rad	2	0.032999	0.008398	-39.84235732	-39.83395932	-39.80935832		
NO2-rad	2	0.013084	-0.014136	-205.1461025	-205.1602385	-205.1330185		

1.2.1. Computed energies of porphyrin complexes

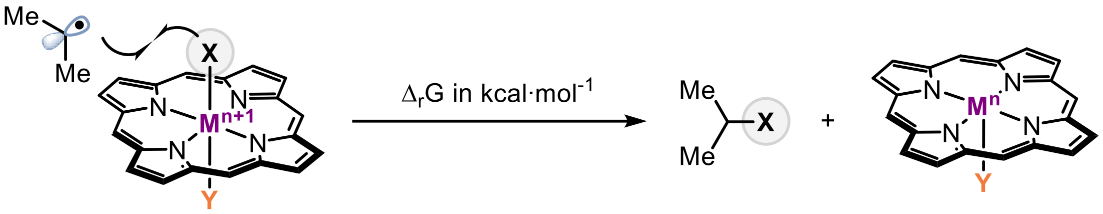

Table S2. Energy of complexes computed energies at the (U)M06-L-D3/def2-TZVP,SMD(MeCN)//(U)M06-L-D3/def2-SVP,SMD(MeCN). Energies in Hartree unless otherwise stated (BDFE and BDE are given in kcal·mol^{-1}). Mult: multiplicity; Porph: Porphyrin.

Compounds (MPorph-Y_m-X)	Mult	Thermal correction		SP energy	*ΔG*	*ΔH*	$Δ_rG$	BDFE	BDE
		to *H*	to *G*						
**FePorph-SMe**	6	0.335746	0.262396	-2690.557453	-2690.295057	-2690.221707			
	4	0.336899	0.264858	-2690.543646	-2690.278788	-2690.206747			
	2	0.336741	0.266228	-2690.544313	-2690.278085	-2690.207572			
FePorph-SMe-OH	5	0.349872	0.271298	-2766.362044	-2766.090746	-2766.012172			
	3	0.351881	0.275715	-2766.375521	-2766.099806	-2766.02364	-50.2	23.9	34.8
	1	0.352517	0.281032	-2766.356755	-2766.075723	-2766.004238			
FePorph-SMe-Cl	5	0.338188	0.260254	-3150.761742	-3150.501488	-3150.423554			
	3	0.339849	0.264643	-3150.770378	-3150.505735	-3150.430529	-38.7	34.2	44.3
	1	0.339788	0.267227	-3150.752276	-3150.485049	-3150.412488			
FePorph-SMe-F	5	0.338325	0.260202	-2790.410906	-2790.150704	-2790.072581			
	3	0.339952	0.264512	-2790.42097	-2790.156458	-2790.081018	-41.8	58.5	68.0

Compounds (MPorph-Y_m-X)	Mult	Thermal correction		SP energy	*ΔG*	*ΔH*	$\Delta_r G$	BDFE	BDE
		to *H*	to *G*						
	1	0.340278	0.268678	-2790.397823	-2790.129145	-2790.057545			
FePorph-SMe-N3	5	0.351187	0.268994	-2854.778463	-2854.509469	-2854.427276			
	3	0.352067	0.272998	-2854.791599	-2854.518601	-2854.439532	-42.7	10.8	23.3
	1	0.353236	0.276926	-2854.776276	-2854.49935	-2854.42304			
FePorph-SMe-SCN	5	0.349488	0.265235	-3181.615798	-3181.350563	-3181.26631			
	3	0.35093	0.269458	-3181.622567	-3181.353109	-3181.271637	-38.8	12.5	24.5
	1	0.351017	0.272551	-3181.608577	-3181.336026	-3181.25756			
FePorph-SMe-ONO2	5	0.357178	0.272959	-2970.910077	-2970.637118	-2970.552899			
	3	0.357477	0.274789	-2970.931407	-2970.656618	-2970.57393	-41.3	16.3	29.7
	1	0.357389	0.27838	-2970.914373	-2970.635993	-2970.556984			
FePorph-SMe-Me	5	0.372996	0.294825	-2730.413992	-2730.119167	-2730.040996			
	3	0.374532	0.297356	-2730.421171	-2730.123815	-2730.046639	-72.3	-3.3	9.8
	1	0.3749	0.301982	-2730.406562	-2730.10458	-2730.031662			
FePorph-SMe-NO2	5	0.350418	0.267866	-2895.709685	-2895.441819	-2895.359267			
	3	0.35212	0.271723	-2895.719278	-2895.447555	-2895.367158	-46.7	-4.9	7.8
	1	0.352186	0.275841	-2895.707153	-2895.431312	-2895.354967			
FePorph-SMe-ONO	5	Flips to NO_2							
	3	0.351974	0.271933	-2895.712039	-2895.440106	-2895.360065	-51.3	-9.5	3.4
	1	0.350477	0.272778	-2895.694028	-2895.42125	-2895.343551			
**ScPorph**	2	0.290171	0.226531	-1749.509851	-1749.28332	-1749.21968			
ScPorph-OH	1	0.307479	0.23768	-1825.404927	-1825.167247	-1825.097448	-0.5	73.6	82.4
ScPorph-Cl	1	0.2962	0.229429	-2209.803197	-2209.573768	-2209.506997	11.3	84.3	93.6
ScPorph-F	1	0.296397	0.230515	-1849.465678	-1849.235163	-1849.169281	15.0	115.3	124.7
ScPorph-N3	1	0.309811	0.238929	-1913.818491	-1913.579562	-1913.50868	2.9	56.5	68.0
ScPorph-SCN	1	0.307681	0.236303	-2240.644005	-2240.407702	-2240.336324	2.9	54.1	66.3
ScPorph-ONO2	1	0.314114	0.242316	-2029.973573	-2029.731257	-2029.659459	12.9	70.5	84.7

Compounds (MPorph-Y_m-X)	Mult	Thermal correction		SP energy	*ΔG*	*ΔH*	$\Delta_r G$	BDFE	BDE
		to *H*	to *G*						
ScPorph-Me	1	0.32914	0.260212	-1789.414613	-1789.154401	-1789.085473	-45.7	23.3	35.4
ScPorph-NO2	1	Flips to ONO							
ScPorph-ONO	1	0.307906	0.238214	-1954.743353	-1954.505139	-1954.435447	-3.2	38.6	51.9
**TiPorph-Cl**	2	0.296246	0.228413	-2298.50103	-2298.272617	-2298.204784			
TiPorph-Cl-OH	1	0.310611	0.239776	-2374.373657	-2374.133881	-2374.063046	-14.7	59.4	70.2
TiPorph-Cl-Cl	1	0.29855	0.226256	-2758.757118	-2758.530862	-2758.458568	-8.9	64.0	72.6
TiPorph-Cl-F	1	0.299098	0.227925	-2398.420638	-2398.192713	-2398.12154	-5.0	95.4	104.1
TiPorph-Cl-N3	1	0.312324	0.236218	-2462.776671	-2462.540453	-2462.464347	-14.9	38.6	49.5
TiPorph-Cl-SCN	1	0.310225	0.233187	-2789.602116	-2789.368929	-2789.291891	-14.8	36.5	47.8
TiPorph-Cl-ONO2	1	0.316395	0.238131	-2578.918523	-2578.680392	-2578.602128	-12.3	45.3	58.1
TiPorph-Cl-Me	1	0.332729	0.260199	-2338.383364	-2338.123165	-2338.050635	-58.6	10.4	22.9
TiPorph-Cl-NO2	1	0.311003	0.235279	-2503.692959	-2503.45768	-2503.381956	-26.2	15.6	27.7
TiPorph-Cl-ONO	1	0.309516	0.231596	-2503.698032	-2503.466436	-2503.388516	-20.7	21.1	31.8
**TiPorph**	3	0.290679	0.226322	-1838.204407	-1837.978085	-1837.913728			
	1	0.291022	0.228212	-1838.192233	-1837.964021	-1837.901211			
TiPorph-OH	2	0.306896	0.239223	-1914.113986	-1913.874763	-1913.80709	7.5	81.6	92.2
TiPorph-Cl	2	0.296246	0.228413	-2298.50103	-2298.272617	-2298.204784	13.9	86.8	96.0
TiPorph-F	2	0.296623	0.230378	-1938.165973	-1937.935595	-1937.86935	18.5	118.9	128.4
TiPorph-N3	2	0.30844	0.238099	-2002.514493	-2002.276394	-2002.206053	4.2	57.8	70.1
TiPorph-SCN	2	0.306504	0.233767	-2329.337349	-2329.103582	-2329.030845	3.6	54.8	66.6
TiPorph-ONO2	2	0.314087	0.241972	-2118.670295	-2118.428323	-2118.356208	14.4	72.0	86.4
TiPorph-Me	2	0.328905	0.260242	-1878.125138	-1877.864896	-1877.796233	-35.9	33.2	45.9
TiPorph-NO2	2	0.307572	0.236758	-2043.453229	-2043.216471	-2043.145657	7.2	49.0	62.1
TiPorph-ONO	2	0.306183	0.232868	-2043.437695	-2043.204827	-2043.131512	-0.1	41.7	53.2

Compounds (MPorph-Y_m-X)	Mult	Thermal correction to *H*	Thermal correction to *G*	SP energy	*ΔG*	*ΔH*	$\Delta_r G$	BDFE	BDE
**VPorph-Cl**	3	0.296378	0.228732	-2393.011076	-2392.782344	-2392.714698			
	1	0.296261	0.230035	-2392.985146	-2392.755111	-2392.688885			
VPorph-Cl-OH	2	0.311122	0.240544	-2468.864588	-2468.624044	-2468.553466	-27.0	47.1	58.0
VPorph-Cl-Cl	2	0.299026	0.227505	-2853.243206	-2853.015701	-2852.94418	-24.5	48.4	57.3
VPorph-Cl-F	2	0.299633	0.228386	-2492.907402	-2492.679016	-2492.607769	-19.7	80.7	89.2
VPorph-Cl-N3	2	0.312877	0.237993	-2557.266996	-2557.029003	-2556.954119	-28.2	25.4	36.9
VPorph-Cl-SCN	2	0.31042	0.233667	-2884.089199	-2883.855532	-2883.778779	-29.3	22.0	33.3
VPorph-Cl-ONO2	2	0.316801	0.24015	-2673.406065	-2673.165915	-2673.089264	-27.5	30.1	43.8
VPorph-Cl-Me	2	0.3337	0.261918	-2432.88076	-2432.618842	-2432.54706	-67.4	1.6	14.4
VPorph-Cl-NO2	2	0.311259	0.236458	-2598.184824	-2597.948366	-2597.873565	-38.2	3.6	16.2
VPorph-Cl-ONO	2	0.310011	0.23432	-2598.185911	-2597.951591	-2597.8759	-36.2	5.7	17.7
**VPorph**	4	0.291878	0.228032	-1932.734219	-1932.506187	-1932.442341			
	2	0.291577	0.228171	-1932.704685	-1932.476514	-1932.413108			
VPorph-OH	3	0.308261	0.241586	-2008.625678	-2008.384092	-2008.317417	-4.3	69.8	80.7
	1	0.307712	0.241609	-2008.60278	-2008.361171	-2008.295068			
VPorph-Cl	3	0.296378	0.228732	-2393.011076	-2392.782344	-2392.714698	2.4	75.3	84.2
	1	0.296261	0.230035	-2392.985146	-2392.755111	-2392.688885			
VPorph-F	3	0.296702	0.229879	-2032.668173	-2032.438294	-2032.371471	2.6	102.9	111.8
	1	0.296207	0.230419	-2032.643176	-2032.412757	-2032.346969			
VPorph-N3	3	0.309925	0.238627	-2097.029579	-2096.790952	-2096.719654	-4.3	49.3	60.7
	1	0.309666	0.239149	-2097.002615	-2096.763466	-2096.692949			
VPorph-SCN	3	0.307786	0.23502	-2423.855691	-2423.620671	-2423.547905	-3.4	47.9	59.4
	1	0.307298	0.235346	-2423.82896	-2423.593614	-2423.521662			
VPorph-ONO2	3	0.314033	0.240945	-2213.172925	-2212.93198	-2212.858892	-1.0	56.6	70.1
	1	0.312384	0.240811	-2213.143214	-2212.902403	-2212.83083			
VPorph-Me	3	0.330065	0.261949	-1972.642019	-1972.38007	-1972.311954	-44.0	25.1	37.8

Compounds (MPorph-Y_m-X)	Mult	Thermal correction		SP energy	*ΔG*	*ΔH*	$\Delta_r G$	BDFE	BDE
		to *H*	to *G*						
	1	0.330457	0.263069	-1972.619841	-1972.356772	-1972.289384			
VPorph-NO2	3	0.308416	0.237421	-2137.958798	-2137.721377	-2137.650382	-7.3	34.5	47.1
	1	0.307935	0.238701	-2137.939923	-2137.701222	-2137.631988			
VPorph-ONO	3	0.307261	0.234418	-2137.950743	-2137.716325	-2137.643482	-10.5	31.3	42.7
	1	Dissociates							
**CrPorph-Cl**	4	0.296737	0.229293	-2493.479696	-2493.250403	-2493.182959			
	2	0.296627	0.229939	-2493.428206	-2493.198267	-2493.131579			
CrPorph-Cl-OH	3	0.311118	0.240365	-2569.302905	-2569.06254	-2568.991787	-45.5	28.6	39.2
	1	0.311638	0.242717	-2569.246391	-2569.003674	-2568.934753			
CrPorph-Cl-Cl	3	0.298171	0.225659	-2953.700999	-2953.47534	-2953.402828	-29.8	43.1	51.3
	1	0.298311	0.227562	-2953.649159	-2953.421597	-2953.350848			
CrPorph-Cl-F	3	0.298511	0.226468	-2593.351789	-2593.125321	-2593.053278	-33.3	67.0	74.9
	1	0.299819	0.23014	-2593.284344	-2593.054204	-2592.984525			
CrPorph-Cl-N3	3	0.312119	0.236376	-2657.718613	-2657.482237	-2657.406494	-37.5	16.0	26.9
	1	0.315602	0.24234	-2657.670602	-2657.428262	-2657.355			
CrPorph-Cl-SCN	3	0.309906	0.232973	-2984.551101	-2984.318128	-2984.241195	-32.7	18.6	29.7
	1	0.309676	0.233304	-2984.499601	-2984.266297	-2984.189925			
CrPorph-Cl-ONO2	3	0.315857	0.237332	-2773.863265	-2773.625933	-2773.547408	-32.5	25.1	37.4
	1	0.315928	0.238425	-2773.811181	-2773.572756	-2773.495253			
CrPorph-Cl-Me	3	0.333225	0.259788	-2533.339462	-2533.079674	-2533.006237	-72.0	-2.9	8.7
	1	0.334344	0.263612	-2533.29832	-2533.034708	-2532.963976			
CrPorph-Cl-NO2	3	0.310463	0.234202	-2698.645175	-2698.410973	-2698.334712	-41.6	0.2	11.8
	1	0.310665	0.235693	-2698.594556	-2698.358863	-2698.283891			
CrPorph-Cl-ONO	3	0.309281	0.232342	-2698.642314	-2698.409972	-2698.333033	-42.2	-0.4	10.7
	1	0.309926	0.234785	-2698.59203	-2698.357245	-2698.282104			

Compounds (MPorph-Y_m-X)	Mult	Thermal correction		SP energy	*ΔG*	*ΔH*	$\Delta_r G$	BDFE	BDE
		to *H*	to *G*						
**CrPorph**	5	0.292949	0.229623	-2033.218205	-2032.988582	-2032.925256			
	3	0.292591	0.229699	-2033.169904	-2032.940205	-2032.877313			
	1	0.292681	0.231084	-2033.101729	-2032.870645	-2032.809048			
CrPorph-OH	4	0.308622	0.240869	-2109.082406	-2108.841537	-2108.773784	-19.9	54.2	64.1
	2	0.308701	0.242153	-2109.034882	-2108.792729	-2108.726181			
CrPorph-Cl	4	0.296737	0.229293	-2493.479696	-2493.250403	-2493.182959	-6.6	66.3	75.0
	2	0.296627	0.229939	-2493.428206	-2493.198267	-2493.131579			
CrPorph-F	4	0.297166	0.230839	-2133.13191	-2132.901071	-2132.834744	-9.7	90.6	99.5
	2	0.297021	0.231173	-2133.080168	-2132.848995	-2132.783147			
CrPorph-N3	4	0.310442	0.239408	-2197.497408	-2197.258	-2197.186966	-13.9	39.6	50.9
	2	0.310103	0.239142	-2197.447717	-2197.208575	-2197.137614			
CrPorph-SCN	4	0.308089	0.235193	-2524.326276	-2524.091083	-2524.018187	-10.9	40.4	51.5
	2	0.307813	0.234541	-2524.275607	-2524.041066	-2523.967794			
CrPorph-ONO2	4	0.314551	0.240936	-2313.640477	-2313.399541	-2313.325926	-10.3	47.3	60.1
	2	0.314287	0.240649	-2313.583588	-2313.342939	-2313.269301			
CrPorph-Me	4	0.33101	0.263547	-2073.121285	-2072.857738	-2072.790275	-46.9	22.1	34.9
	2	0.330771	0.263504	-2073.072904	-2072.8094	-2072.742133			
CrPorph-NO2	4	0.308767	0.237547	-2238.424155	-2238.186608	-2238.115388	-18.1	23.7	35.8
	2	0.308621	0.238002	-2238.36929	-2238.131288	-2238.060669			
CrPorph-ONO	4	0.30777	0.235694	-2238.420431	-2238.184737	-2238.112661	-19.3	22.5	34.1
	2	0.307457	0.235153	-2238.363877	-2238.128724	-2238.05642			
**MnPorph-Cl**	5	0.296687	0.228003	-2599.991078	-2599.763075	-2599.694391			
	3	0.29708	0.230212	-2599.944623	-2599.714411	-2599.647543			
	1	0.296791	0.230597	-2599.890848	-2599.660251	-2599.594057			
MnPorph-Cl-OH	4	0.311935	0.23969	-2675.813469	-2675.573779	-2675.501534	-46.4	27.7	38.1
	2	0.311051	0.240431	-2675.770795	-2675.530364	-2675.459744			

Compounds (MPorph-Y_m-X)	Mult	Thermal correction		SP energy	*ΔG*	*ΔH*	$\Delta_r G$	BDFE	BDE
		to *H*	to *G*						
MnPorph-Cl-Cl	4	0.299701	0.226845	-3060.20176	-3059.974915	-3059.902059	-38.0	34.9	43.6
	2	0.298508	0.226855	-3060.167978	-3059.941123	-3059.86947			
MnPorph-Cl-F	4	0.300227	0.229075	-2699.855787	-2699.626712	-2699.55556	-40.4	59.9	69.2
	2	0.298794	0.227996	-2699.820752	-2699.592756	-2699.521958			
MnPorph-Cl-N3	4	0.313293	0.237324	-2764.22378	-2763.986456	-2763.910487	-42.8	10.7	22.2
	2	0.312312	0.237943	-2764.187307	-2763.949364	-2763.874995			
MnPorph-Cl-SCN	4	0.311018	0.233559	-3091.052043	-3090.818484	-3090.741025	-40.4	10.8	22.4
	2	0.310131	0.234283	-3091.019263	-3090.78498	-3090.709132			
MnPorph-Cl-ONO2	4	0.317287	0.238611	-2880.362934	-2880.124323	-2880.045647	-41.5	16.1	29.1
	2	0.316295	0.238155	-2880.329719	-2880.091564	-2880.013424			
MnPorph-Cl-Me	4	0.334652	0.26121	-2639.865343	-2639.604133	-2639.530691	-64.6	4.5	16.9
	2	0.334578	0.262714	-2639.816925	-2639.554211	-2639.482347			
MnPorph-Cl-NO2	4	0.31208	0.235579	-2805.15521	-2804.919631	-2804.84313	-44.1	-2.3	9.9
	2	0.318281	0.243257	-2805.118609	-2804.875352	-2804.800328			
MnPorph-Cl-ONO	4	0.310747	0.234237	-2805.144424	-2804.910187	-2804.833677	-50.0	-8.2	3.9
	2	0.309658	0.233577	-2805.110166	-2804.876589	-2804.800508			
**MnPorph**	6	0.291569	0.226841	-2139.724827	-2139.497986	-2139.433258			
	4	0.292851	0.229318	-2139.721543	-2139.492225	-2139.428692			
	2	0.293029	0.230758	-2139.658644	-2139.427886	-2139.365615			
MnPorph-OH	5	0.307993	0.238003	-2215.576047	-2215.338044	-2215.268054	-28.0	46.1	55.5
	3	0.308789	0.242032	-2215.553197	-2215.311165	-2215.244408			
	1	0.309172	0.244462	-2215.512054	-2215.267592	-2215.202882			
MnPorph-Cl	5	0.296687	0.228003	-2599.991078	-2599.763075	-2599.694391	-4.6	68.3	77.2
	3	0.29708	0.230212	-2599.944623	-2599.714411	-2599.647543			
	1	0.296791	0.230597	-2599.890848	-2599.660251	-2599.594057			
MnPorph-F	5	0.296922	0.228933	-2239.636059	-2239.407126	-2239.339137	-11.8	88.5	97.2

Compounds (MPorph-Y_m-X)	Mult	Thermal correction		SP energy	*ΔG*	*ΔH*	$\Delta_r G$	BDFE	BDE
		to *H*	to *G*						
	3	0.297568	0.231474	-2239.597129	-2239.365655	-2239.299561			
	1	0.297407	0.232862	-2239.574628	-2239.341766	-2239.277221			
MnPorph-N3	5	0.310194	0.236612	-2304.003597	-2303.766985	-2303.693403	-14.2	39.4	49.9
	3	0.310563	0.23949	-2303.964568	-2303.725078	-2303.654005			
	1	0.310486	0.240281	-2303.943052	-2303.702771	-2303.632566			
MnPorph-SCN	5	0.30814	0.233367	-2630.841009	-2630.607642	-2630.532869	-6.4	44.9	55.6
	3	0.309987	0.238751	-2630.799975	-2630.561224	-2630.489988			
	1	0.306727	0.235904	-2630.749123	-2630.513219	-2630.442396			
MnPorph-ONO2	5	0.31443	0.238834	-2420.151407	-2419.912573	-2419.836977	-8.0	49.6	62.1
	3	0.314703	0.241663	-2420.106228	-2419.864565	-2419.791525			
	1	0.314494	0.24173	-2420.082238	-2419.840508	-2419.767744			
MnPorph-Me	5	0.328966	0.256613	-2179.606227	-2179.349614	-2179.277261	-59.3	11.1	21.7
	3	0.331849	0.264654	-2179.603833	-2179.339179	-2179.271984			
	1	0.331058	0.26441	-2179.566904	-2179.302494	-2179.235846			
MnPorph-NO2	5	0.308173	0.233872	-2344.922036	-2344.688164	-2344.613863	-23.0	18.8	29.9
	3	0.308751	0.238271	-2344.905125	-2344.666854	-2344.596374			
	1	0.309117	0.240931	-2344.858754	-2344.617823	-2344.549637			
MnPorph-ONO	5	0.307803	0.233472	-2344.924603	-2344.691131	-2344.6168	-21.2	20.6	31.7
	3	0.30813	0.236638	-2344.886213	-2344.649575	-2344.578083			
	1	0.307721	0.236659	-2344.855977	-2344.619318	-2344.548256			
**FePorph-Cl**	6	0.296185	0.227495	-2712.676282	-2712.448787	-2712.380097			
	4	0.297163	0.228796	-2712.664862	-2712.436066	-2712.367699			
	2	0.297301	0.230764	-2712.642523	-2712.411759	-2712.345222			
FePorph-Cl-OH	5	0.308917	0.234912	-2788.470044	-2788.235132	-2788.161127			
	3	0.311958	0.240695	-2788.482533	-2788.241838	-2788.170575	-57.5	16.6	27.7
	1	0.312294	0.243138	-2788.454911	-2788.211773	-2788.142617			

Compounds (MPorph-Y_m-X)	Mult	Thermal correction		SP energy	*ΔG*	*ΔH*	$\Delta_r G$	BDFE	BDE
		to *H*	to *G*						
FePorph-Cl-Cl	5	0.298561	0.223304	-3172.86661	-3172.643306	-3172.568049	-48.9	24.1	31.3
	3	0.299637	0.226006	-3172.867534	-3172.641528	-3172.567897			
	1	0.298584	0.227257	-3172.864646	-3172.637389	-3172.566062			
FePorph-Cl-F	5	0.297515	0.224396	-2812.522909	-2812.298513	-2812.225394	-49.1	51.2	59.2
	3	0.300515	0.230041	-2812.520552	-2812.290511	-2812.220037			
	1	0.299491	0.229523	-2812.488525	-2812.259002	-2812.189034			
FePorph-Cl-N3	5	0.311165	0.233153	-2876.891607	-2876.658454	-2876.580442	-51.4	2.1	12.4
	3	0.31306	0.23778	-2876.892643	-2876.654863	-2876.579583			
	1	0.312969	0.239286	-2876.870181	-2876.630895	-2876.557212			
FePorph-Cl-SCN	5	0.310277	0.230694	-3203.722187	-3203.491493	-3203.41191	-48.4	2.9	13.1
	3	0.310593	0.232228	-3203.720208	-3203.48798	-3203.409615			
	1	0.310501	0.23564	-3203.701279	-3203.465639	-3203.390778			
FePorph-Cl-ONO2	5	0.316111	0.236105	-2993.02679	-2992.790685	-2992.710679	-53.6	4.0	16.2
	3	0.316798	0.239303	-2993.029852	-2992.790549	-2992.713054	-53.7	3.9	17.6
	1	0.316211	0.239282	-2993.004119	-2992.764837	-2992.687908			
FePorph-Cl-Me	5	0.333301	0.25904	-2752.53398	-2752.27494	-2752.200679			
	3	0.33518	0.262621	-2752.539461	-2752.27684	-2752.204281	-72.7	-3.7	9.3
	1	0.33487	0.26488	-2752.506196	-2752.241316	-2752.171326			
FePorph-Cl-NO2	5	0.311149	0.231372	-2917.828254	-2917.596882	-2917.517105	-49.4	-7.6	2.5
	3	0.31236	0.236488	-2917.825164	-2917.588676	-2917.512804			
	1	0.311329	0.237527	-2917.80019	-2917.562663	-2917.488861			
FePorph-Cl-ONO	5	0.311806	0.229249	-2917.814443	-2917.585194	-2917.502637	-56.8	-15.0	-6.6
	3	0.310282	0.23353	-2917.812665	-2917.579135	-2917.502383			
	1	0.309795	0.233335	-2917.789251	-2917.555916	-2917.479456			
**FePorph**	5	0.292	0.227316	-2252.415651	-2252.188335	-2252.123651			
	3	0.293546	0.230638	-2252.414787	-2252.184149	-2252.121241			

Compounds (MPorph-Y$_m$-X)	Mult	Thermal correction		SP energy	*ΔG*	*ΔH*	*Δ$_r$G*	BDFE	BDE
		to *H*	to *G*						
	1	0.29357	0.231723	-2252.377528	-2252.145805	-2252.083958			
FePorph-OH	6	0.307743	0.239002	-2328.276157	-2328.037155	-2327.968414	-22.5	51.6	61.7
	4	0.308557	0.240124	-2328.258385	-2328.018261	-2327.949828			
	2	0.30939	0.242441	-2328.252952	-2328.010511	-2327.943562			
FePorph-Cl	6	0.296185	0.227495	-2712.676282	-2712.448787	-2712.380097	-7.5	65.4	74.2
	4	0.297163	0.228796	-2712.664862	-2712.436066	-2712.367699			
	2	0.297301	0.230764	-2712.642523	-2712.411759	-2712.345222			
FePorph-F	6	0.296588	0.229105	-2352.325113	-2352.096008	-2352.028525	-12.7	87.6	96.6
	4	0.297286	0.229609	-2352.312139	-2352.08253	-2352.014853			
	2	0.297434	0.231681	-2352.288226	-2352.056545	-2351.990792			
FePorph-N3	6	0.309519	0.236018	-2416.689899	-2416.453881	-2416.38038	-16.3	37.2	47.7
	4	0.310558	0.237857	-2416.679947	-2416.44209	-2416.369389			
	2	0.310831	0.240469	-2416.663587	-2416.423118	-2416.352756			
FePorph-SCN	6	0.307242	0.232606	-2743.519207	-2743.286601	-2743.211965	-13.5	37.7	48.6
	4	0.311081	0.238339	-2743.51414	-2743.275801	-2743.203059			
	2	0.308982	0.237667	-2743.500137	-2743.26247	-2743.191155			
FePorph-ONO2	6	0.313868	0.23933	-2532.833658	-2532.594328	-2532.51979	-13.4	44.2	57.3
	4	0.314857	0.240083	-2532.823678	-2532.583595	-2532.508821			
	2	0.314754	0.241732	-2532.803758	-2532.562026	-2532.489004			
FePorph-Me	6	0.329685	0.259396	-2292.303065	-2292.043669	-2291.97338	-55.6	13.4	25.3
	4	0.329821	0.260629	-2292.288884	-2292.028255	-2291.959063			
	2	0.331893	0.264532	-2292.306517	-2292.041985	-2291.974624			
FePorph-NO2	6	0.307522	0.232383	-2457.606186	-2457.373803	-2457.298664	-26.0	15.8	26.4
	4	0.308551	0.234004	-2457.599894	-2457.36589	-2457.291343			
	2	0.309235	0.235849	-2457.601643	-2457.365794	-2457.292408			
FePorph-ONO	6	0.306764	0.231849	-2457.608199	-2457.37635	-2457.301435	-24.4	17.4	28.1
	4	0.307764	0.231603	-2457.599516	-2457.367913	-2457.291752			

Compounds (MPorph-Y_m-X)	Mult	Thermal correction		SP energy	*ΔG*	*ΔH*	$\Delta_r G$	BDFE	BDE
		to *H*	to *G*						
	2	0.30796	0.236469	-2457.585269	-2457.3488	-2457.277309			
**CoPorph**	4	0.292472	0.228667	-2371.456364	-2371.227697	-2371.163892			
	2	0.294091	0.231884	-2371.483215	-2371.251331	-2371.189124			
CoPorph-OH	5	0.307554	0.238486	-2447.294899	-2447.056413	-2446.987345			
	3	0.308805	0.240342	-2447.29836	-2447.058018	-2446.989555			
	1	0.309728	0.242971	-2447.307307	-2447.064336	-2446.997579	-45.0	29.1	38.9
CoPorph-Cl	5	0.295984	0.226647	-2831.692765	-2831.466118	-2831.396781			
	3	0.296768	0.228138	-2831.701235	-2831.473097	-2831.404467			
	1	0.297462	0.230779	-2831.707393	-2831.476614	-2831.409931	-29.6	43.4	51.9
CoPorph-F	5	0.296364	0.228211	-2471.339	-2471.110789	-2471.042636			
	3	0.297125	0.229708	-2471.343891	-2471.114183	-2471.046766			
	1	0.297855	0.230681	-2471.348102	-2471.117421	-2471.050247	-38.8	61.5	69.1
CoPorph-N3	5	0.309478	0.235669	-2535.708424	-2535.472755	-2535.398946			
	3	0.310483	0.238411	-2535.720702	-2535.482291	-2535.410219			
	1	0.311026	0.241023	-2535.729779	-2535.488756	-2535.418753	-34.0	19.6	30.7
CoPorph-SCN	5	0.307241	0.232173	-2862.537552	-2862.305379	-2862.230311			
	3	0.308318	0.233846	-2862.556099	-2862.322253	-2862.247781			
	1	0.308838	0.236944	-2862.566137	-2862.329193	-2862.257299	-26.3	24.9	35.9
CoPorph-ONO2	5	0.313667	0.238764	-2651.850317	-2651.611553	-2651.53665			
	3	0.314403	0.23891	-2651.861536	-2651.622626	-2651.547133			
	1	0.315172	0.242021	-2651.867567	-2651.625546	-2651.552395	-33.3	24.3	36.7
CoPorph-Me	5	0.32949	0.259265	-2411.324987	-2411.065722	-2410.995497			
	3	0.330612	0.261793	-2411.338999	-2411.077206	-2411.008387			
	1	0.332577	0.265782	-2411.378282	-2411.1125	-2411.045705	-52.0	17.1	29.6
CoPorph-NO2	5	0.30788	0.234467	-2576.626039	-2576.391572	-2576.318159			
	3	0.308786	0.235079	-2576.645197	-2576.410118	-2576.336411			

Compounds (MPorph-Y_m-X)	Mult	Thermal correction		SP energy	*ΔG*	*ΔH*	$\Delta_r G$	BDFE	BDE
		to *H*	to *G*						
	1	0.310059	0.240544	-2576.672669	-2576.432125	-2576.36261	-28.9	12.9	25.4
CoPorph-ONO	5	0.308212	0.233539	-2576.624686	-2576.391147	-2576.316474			
	3	0.308596	0.235681	-2576.639229	-2576.403548	-2576.330633			
	1	0.308614	0.237014	-2576.649863	-2576.412849	-2576.341249	-41.0	0.8	12.0
**NiPorph-Cl**	4	0.294411	0.22476	-2957.223849	-2956.999089	-2956.929438			
	2	0.297178	0.228025	-2957.238696	-2957.010671	-2956.941518			
NiPorph-Cl-OH	5	0.309464	0.2366	-3033.020687	-3032.784087	-3032.711223			
	3	0.310045	0.237556	-3033.034347	-3032.796791	-3032.724302			
	1	0.31222	0.242111	-3033.04053	-3032.798419	-3032.72831	-60.8	13.2	25.3
NiPorph-Cl-Cl	5	0.297233	0.223349	-3417.420455	-3417.197106	-3417.123222			
	3	0.298253	0.225176	-3417.440045	-3417.214869	-3417.141792	-42.8	30.1	39.0
	1	0.299773	0.228538	-3417.433841	-3417.205303	-3417.134068			
NiPorph-Cl-F	5	0.297586	0.22491	-3057.058883	-3056.833973	-3056.761297			
	3	0.298188	0.226152	-3057.078139	-3056.851987	-3056.779951	-54.4	45.9	54.9
	1	0.300057	0.230294	-3057.077605	-3056.847311	-3056.777548			
NiPorph-Cl-N3	5	0.309592	0.234168	-3121.444419	-3121.210251	-3121.134827			
	3	0.311616	0.235354	-3121.458751	-3121.223397	-3121.147135	-49.5	4.1	15.7
	1	0.313218	0.238894	-3121.46032	-3121.221426	-3121.147102			
NiPorph-Cl-SCN	5	0.308822	0.229397	-3448.281144	-3448.051747	-3447.972322			
	3	0.310095	0.230873	-3448.299177	-3448.068304	-3447.989082	-39.0	12.2	23.0
	1	0.311147	0.235076	-3448.293806	-3448.05873	-3447.982659			
NiPorph-Cl-ONO2	5	0.314551	0.233282	-3237.584209	-3237.350927	-3237.269658			
	3	0.315863	0.23552	-3237.601643	-3237.366123	-3237.28578	-45.1	12.5	24.7
	1	0.317263	0.239795	-3237.59873	-3237.358935	-3237.281467			
NiPorph-Cl-Me	5	0.329656	0.250078	-2997.075986	-2996.825908	-2996.74633			
	3	0.333322	0.260364	-2997.109047	-2996.848683	-2996.775725			

Compounds (MPorph-Y_m-X)	Mult	Thermal correction		SP energy	*ΔG*	*ΔH*	$\Delta_r G$	BDFE	BDE
		to *H*	to *G*						
	1	0.335934	0.264951	-2997.123392	-2996.858441	-2996.787458	-60.4	8.7	23.0
NiPorph-Cl-NO2	5	0.311279	0.231566	-3162.375227	-3162.143661	-3162.063948			
	3	0.310271	0.233873	-3162.397434	-3162.163561	-3162.087163			
	1	0.312713	0.238096	-3162.404169	-3162.166073	-3162.091456	-44.8	-3.0	10.6
NiPorph-Cl-ONO	5	dissociates							
	3	dissociates							
	1	0.311091	0.234825	-3162.378495	-3162.14367	-3162.067404	-58.9	-17.1	-4.5
**NiPorph**	3	0.293066	0.229426	-2497.005024	-2496.775598	-2496.711958			
	1	0.294358	0.231521	-2497.020449	-2496.788928	-2496.726091			
NiPorph-OH	4	0.307881	0.239386	-2572.817241	-2572.577855	-2572.50936			
	2	0.308926	0.240624	-2572.825112	-2572.584488	-2572.516186	-55.9	18.2	27.4
NiPorph-Cl	4	0.294411	0.22476	-2957.223849	-2956.999089	-2956.929438			
	2	0.297178	0.228025	-2957.238696	-2957.010671	-2956.941518	-31.8	41.1	48.5
NiPorph-F	4	0.295077	0.22549	-2596.861783	-2596.636293	-2596.566706			
	2	0.297363	0.229692	-2596.877233	-2596.647541	-2596.57987	-43.5	56.8	64.5
NiPorph-N3	4	0.309435	0.237095	-2661.236381	-2660.999286	-2660.926946			
	2	0.310525	0.239288	-2661.257115	-2661.017827	-2660.94659	-39.3	14.2	25.0
NiPorph-SCN	4	0.30511	0.228575	-2988.079879	-2987.851304	-2987.774769			
	2	0.308491	0.235312	-2988.096056	-2987.860744	-2987.787565	-30.1	21.1	31.7
NiPorph-ONO2	4	0.311261	0.238017	-2777.387022	-2777.149005	-2777.075761			
	2	0.314792	0.239763	-2777.399658	-2777.159895	-2777.084866	-35.4	22.2	33.8
NiPorph-Me	4	0.329538	0.259023	-2536.855589	-2536.596566	-2536.526051			
	2	0.331261	0.263227	-2536.893243	-2536.630016	-2536.561982	-64.6	4.5	16.6
NiPorph-NO2	4	0.306068	0.231089	-2702.166412	-2701.935323	-2701.860344			
	2	0.309192	0.236251	-2702.189496	-2701.953245	-2701.880304	-39.2	2.6	13.3
NiPorph-ONO	4	0.304671	0.23121	-2702.164792	-2701.933582	-2702.164792			

Compounds (MPorph-Y_m-X)	Mult	Thermal correction		SP energy	*ΔG*	*ΔH*	$\Delta_r G$	BDFE	BDE
		to *H*	to *G*						
	2	0.308065	0.233848	-2702.176002	-2701.942154	-2701.867937	-46.2	-4.4	5.5
**CuPorph**	2	0.293165	0.22978	-2629.177267	-2628.947487	-2628.884102			
CuPorph-OH	3	0.307438	0.237695	-2704.965881	-2704.728186	-2704.658443	-65.3	8.8	17.5
	1	0.306954	0.238343	-2704.938486	-2704.700143	-2704.631532			
CuPorph-Cl	3	0.295617	0.226623	-3089.380713	-3089.15409	-3089.085096	-41.3	31.6	39.4
	1	0.295497	0.226873	-3089.366361	-3089.139488	-3089.070864			
CuPorph-F	3	0.2959	0.227359	-2729.015256	-2728.787897	-2728.719356	-55.0	45.4	52.9
	1	0.295738	0.227882	-2729.001852	-2728.77397	-2728.706114			
CuPorph-N3	3	0.309078	0.236248	-2793.399915	-2793.163667	-2793.090837	-47.3	6.2	16.4
	1	0.3091	0.236483	-2793.383682	-2793.147199	-2793.074582			
CuPorph-SCN	3	0.306996	0.232267	-3120.239668	-3120.007401	-3119.932672	-37.6	13.7	23.6
	1	0.307026	0.233118	-3120.227066	-3119.993948	-3119.92004			
CuPorph-ONO2	3	0.313003	0.237286	-2909.540613	-2909.303327	-2909.22761	-44.9	12.7	24.3
	1	0.313252	0.236027	-2909.530672	-2909.294645	-2909.21742			
CuPorph-Me	3	0.329251	0.25758	-2669.029633	-2668.772053	-2668.700382	-74.9	-5.9	4.3
	1	0.328848	0.257612	-2669.029277	-2668.771665	-2668.700429			
CuPorph-NO2	3	0.307777	0.232419	-2834.33238	-2834.099961	-2834.024603	-46.7	-4.9	4.7
	1	0.307845	0.230925	-2834.329567	-2834.098642	-2834.021722			
CuPorph-ONO	3	Flips to NO2							
	1	0.308175	0.232249	-2834.327432	-2834.095183	-2834.019257	-49.7	-7.9	1.3
**ZnPorph**	1	0.292479	0.228972	-2768.055626	-2767.826654	-2767.763147			
ZnPorph-OH	2	0.306552	0.237045	-2843.84739	-2843.610345	-2843.540838	-63.4	10.7	19.6
ZnPorph-Cl	2	0.294456	0.225153	-3228.266973	-3228.04182	-3227.972517	-35.9	37.0	44.7
ZnPorph-F	2	0.29458	0.22579	-2867.905202	-2867.679412	-2867.610622	-47.2	53.1	60.6
ZnPorph-N3	2	0.308384	0.235272	-2932.279271	-2932.043999	-2931.970887	-46.6	7.0	17.0

Compounds (MPorph-Y_m-X)	Mult	Thermal correction to *H*	Thermal correction to *G*	SP energy	*ΔG*	*ΔH*	$\Delta_r G$	BDFE	BDE
ZnPorph-SCN	2	0.306079	0.230526	-3259.118597	-3258.888071	-3258.812518	-36.7	14.6	24.1
ZnPorph-ONO2	2	0.312154	0.236226	-3048.425679	-3048.189453	-3048.113525	-40.5	17.1	28.6
ZnPorph-Me	2	0.328143	0.25618	-2807.908308	-2807.652128	-2807.580165	-74.4	-5.3	4.8
ZnPorph-NO2	2	0.306838	0.231466	-2973.206292	-2972.974826	-2972.899454	-49.4	-7.6	2.1
ZnPorph-ONO	2	dissociates							

1.2.1. Computed energies of bis(oxazoline) complexes

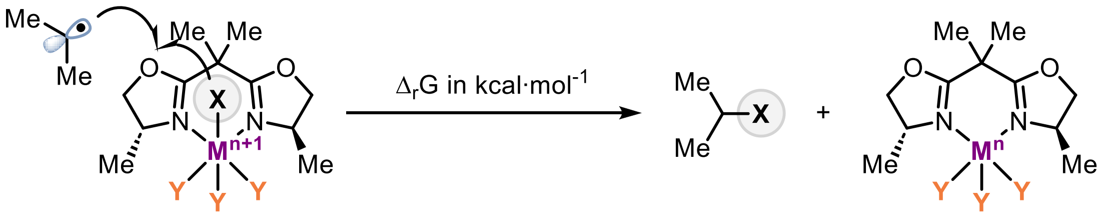

Table S3. Energy of complexes computed energies at the (U)M06-L-D3/def2-TZVP,SMD(MeCN)//(U)M06-L-D3/def2-SVP,SMD(MeCN). Energies in Hartree unless otherwise stated (BDFE and BDE are given in kcal·mol^{-1}). Mult: multiplicity; BOX: Bis(oxazoline).

Compounds (MBOX-Y_m-X)	Mult	Thermal correction to *H*	Thermal correction to *G*	SP energy	*ΔG*	*ΔH*	$\Delta_r G$	BDFE	BDE
**ScBOX-Cl2**	2	0.308751	0.238871	-2371.497232	-2371.258361	-2371.188481			
ScBOX-Cl2-OH	1	0.326449	0.253924	-2447.428955	-2447.175031	-2447.102506	20.1	94.1	105.2
ScBOX-Cl3	1	0.314629	0.240361	-2831.825623	-2831.585262	-2831.510994	34.2	107.1	115.7
ScBOX-Cl2-F	1	0.315474	0.245332	-2471.487605	-2471.242273	-2471.172131	35.1	135.4	146.0
ScBOX-Cl2-N3	1	0.328649	0.252472	-2535.840384	-2535.587912	-2535.511735	23.8	77.4	89.5
ScBOX-Cl2-SCN	1	0.326368	0.251167	-2862.672965	-2862.421798	-2862.346597	27.4	78.6	92.4
ScBOX-Cl2-ONO2	1	0.332993	0.256168	-2651.988599	-2651.732431	-2651.655606	29.3	86.9	101.8

Compounds (MBOX-Y_m-X)	Mult	Thermal correction		SP energy	*ΔG*	*ΔH*	$\Delta_r G$	BDFE	BDE
		to *H*	to *G*						
ScBOX-Cl2-Me	1	0.347929	0.273365	-2411.436166	-2411.162801	-2411.088237	-24.8	44.2	56.7
ScBOX-Cl2-NO2	1	Flips ONO							
ScBOX-Cl2-ONO	1	0.326501	0.251327	-2576.767932	-2576.516605	-2576.441431	19.7	61.5	75.3
**TiBOX-Cl3**	2	0.31506	0.243435	-2920.505051	-2920.261616	-2920.189991			
TiBOX-Cl3-OH	1	0.329869	0.254453	-2996.398226	-2996.143773	-2996.068357	-1.6	72.5	82.8
TiBOX-Cl4	1	0.31809	0.242389	-3380.777207	-3380.534818	-3380.459117	0.5	73.4	82.2
TiBOX-Cl3-F	1	0.318636	0.244318	-3020.443628	-3020.19931	-3020.124992	6.1	106.4	115.5
TiBOX-Cl3-N3	1	0.331677	0.25287	-3084.802661	-3084.549791	-3084.470984	-2.1	51.4	63.0
TiBOX-Cl3-SCN	1	0.329558	0.249671	-3411.620971	-3411.3713	-3411.291413	-6.4	44.9	56.8
TiBOX-Cl3-ONO2	1	0.335759	0.255631	-3200.940149	-3200.684518	-3200.60439	-2.8	54.8	68.8
TiBOX-Cl3-Me	1	0.351888	0.275083	-2960.40989	-2960.134807	-2960.058002	-44.4	24.6	36.8
TiBOX-Cl3-NO2	1	0.329917	0.249686	-3125.718252	-3125.468566	-3125.388335	-12.5	29.3	41.0
TiBOX-Cl3-ONO	1	0.328629	0.248256	-3125.722834	-3125.474578	-3125.394205	-8.7	33.1	44.7
**TiBOX-Cl2**	3	0.309915	0.238438	-2460.209495	-2459.971057	-2459.89958			
	1	0.309298	0.240953	-2460.213062	-2459.972109	-2459.903764			
TiBOX-Cl2-OH	2	0.326768	0.254106	-2536.125705	-2535.871599	-2535.798937	9.3	83.4	93.4
TiBOX-Cl3	2	0.31506	0.243435	-2920.505051	-2920.261616	-2920.189991	10.7	83.7	92.9
TiBOX-Cl2-F	2	0.315439	0.24327	-2560.179097	-2559.935827	-2559.863658	22.4	122.7	131.1
TiBOX-Cl2-N3	2	0.328215	0.250469	-2624.529659	-2624.27919	-2624.201444	9.7	63.3	73.4
TiBOX-Cl2-SCN	2	0.326268	0.248683	-2951.35387	-2951.105187	-2951.027602	8.3	59.6	70.8
TiBOX-Cl2-ONO2	2	0.332453	0.252561	-2740.674055	-2740.421494	-2740.341602	13.8	71.4	83.5
TiBOX-Cl2-Me	2	0.348538	0.275137	-2500.136496	-2499.861359	-2499.787958	-34.3	34.7	47.0
TiBOX-Cl2-NO2	2	0.326091	0.249812	-2665.464216	-2665.214404	-2665.138125	9.7	51.5	63.6
TiBOX-Cl2-ONO	2	0.326065	0.249172	-2665.450989	-2665.201817	-2665.124924	1.8	43.6	55.3

Compounds (MBOX-Y_m-X)	Mult	Thermal correction		SP energy	*ΔG*	*ΔH*	*$Δ_rG$*	BDFE	BDE
		to *H*	to *G*						
**VBOX-Cl3**	3	0.314793	0.241541	-3015.016615	-3014.775074	-3014.701822			
	1	0.314713	0.241911	-3014.988866	-3014.746955	-3014.674153			
VBOX-Cl3-OH	2	0.329813	0.254018	-3090.86663	-3090.612612	-3090.536817	-29.6	44.5	55.6
VBOX-Cl4	2	0.31922	0.244553	-3475.240226	-3474.995673	-3474.921006	-32.5	40.4	50.9
VBOX-Cl3-F	2	0.318677	0.244387	-3114.906503	-3114.662116	-3114.587826	-25.7	74.6	84.8
VBOX-Cl3-N3	2	0.330531	0.253113	-3179.270048	-3179.016935	-3178.939517	-31.2	22.3	35.8
VBOX-Cl3-SCN	2	0.329425	0.247288	-3506.085719	-3505.838431	-3505.756294	-35.4	15.8	27.3
VBOX-Cl3-ONO2	2	0.334386	0.253636	-3295.406486	-3295.15285	-3295.0721	-31.1	26.5	41.1
VBOX-Cl3-Me	2	0.352324	0.275235	-3054.879538	-3054.604303	-3054.527214	-72.0	-3.0	10.1
VBOX-Cl3-NO2	2	0.330118	0.251309	-3220.188483	-3219.937174	-3219.858365	-40.6	1.2	14.8
VBOX-Cl3-ONO	2	0.327387	0.248373	-3220.189488	-3219.941115	-3219.862101	-38.2	3.6	17.1
**VBOX-Cl2**	4	0.310915	0.239891	-2554.750784	-2554.510893	-2554.439869			
	2	0.309558	0.238283	-2554.708822	-2554.470539	-2554.399264			
VBOX-Cl2-OH	3	0.326828	0.254746	-2630.625478	-2630.370732	-2630.29865	-15.6	58.5	70.5
	1	0.326899	0.254922	-2630.606451	-2630.351529	-2630.279552			
VBOX-Cl3	3	0.314793	0.241541	-3015.016615	-3014.775074	-3014.701822	-5.2	67.8	77.7
	1	0.314713	0.241911	-3014.988866	-3014.746955	-3014.674153			
VBOX-Cl2-F	3	0.315595	0.244529	-2654.675923	-2654.431394	-2654.360328	-4.7	95.6	106.4
	1	0.314523	0.242985	-2654.649728	-2654.406743	-2654.335205			
VBOX-Cl2-N3	3	0.328729	0.252495	-2719.037515	-2718.78502	-2718.708786	-11.0	42.6	55.4
	1	0.327602	0.253887	-2719.007047	-2718.75316	-2718.679445			
VBOX-Cl2-SCN	3	0.326504	0.249278	-3045.861851	-3045.612573	-3045.535347	-11.4	39.9	53.0
	1	0.326323	0.249666	-3045.834375	-3045.584709	-3045.508052			
VBOX-Cl2-ONO2	3	0.33313	0.255718	-2835.180706	-2834.924988	-2834.847576	-8.3	49.3	64.6
	1	0.331617	0.25625	-2835.151869	-2834.895619	-2834.820252			
VBOX-Cl2-Me	3	0.348396	0.272954	-2594.645041	-2594.372087	-2594.296645	-51.9	17.1	29.8

Compounds (MBOX-Y_m-X)	Mult	Thermal correction		SP energy	*ΔG*	*ΔH*	$\Delta_r G$	BDFE	BDE
		to *H*	to *G*						
	1	0.348365	0.274751	-2594.622786	-2594.348035	-2594.274421			
VBOX-Cl2-NO2	3	0.326821	0.248369	-2759.954421	-2759.706052	-2759.6276	-19.9	21.9	34.3
	1	0.326912	0.250732	-2759.924906	-2759.674174	-2759.597994			
VBOX-Cl2-ONO	3	0.326384	0.249387	-2759.958308	-2759.708921	-2759.631924	-18.1	23.7	37.0
	1	0.325165	0.250522	-2759.930858	-2759.680336	-2759.605693			
**CrBOX-Cl3**	4	0.315018	0.241729	-3115.477437	-3115.235708	-3115.162419			
	2	0.315083	0.243398	-3115.425178	-3115.18178	-3115.110095			
CrBOX-Cl3-OH	3	0.329947	0.253987	-3191.294978	-3191.040991	-3190.965031	-49.8	24.3	35.3
	1	0.329856	0.255007	-3191.261952	-3191.006945	-3190.932096			
CrBOX-Cl4	3	0.317356	0.240084	-3575.666718	-3575.426634	-3575.349362	-51.1	21.8	30.6
	1	0.317776	0.243519	-3575.635273	-3575.391754	-3575.317497			
CrBOX-Cl3-F	3	0.318686	0.243397	-3215.325656	-3215.082259	-3215.00697	-51.1	49.2	58.7
	1	0.318389	0.244231	-3215.29527	-3215.051039	-3214.976881			
CrBOX-Cl3-N3	3	0.331362	0.250483	-3279.699983	-3279.4495	-3279.368621	-48.8	4.7	16.0
	1	0.330732	0.251711	-3279.665079	-3279.413368	-3279.334347			
CrBOX-Cl3-SCN	3	0.328667	0.245685	-3606.52149	-3606.275805	-3606.192823	-50.0	1.2	12.2
	1	0.329553	0.248793	-3606.485741	-3606.236948	-3606.156188			
CrBOX-Cl3-ONO2	3	0.334853	0.252272	-3395.83532	-3395.583048	-3395.500467	-50.2	7.4	20.8
	1	0.335753	0.25603	-3395.80138	-3395.54535	-3395.465627			
CrBOX-Cl3-Me	3	0.351354	0.272029	-3155.331296	-3155.059267	-3154.979942	-75.6	-6.5	5.1
	1	0.352524	0.276203	-3155.293738	-3155.017535	-3154.941214			
CrBOX-Cl3-NO2	3	0.329941	0.247978	-3320.624547	-3320.376569	-3320.294606	-54.0	-12.2	-0.5
	1	0.33034	0.251535	-3320.589913	-3320.338378	-3320.259573			
CrBOX-Cl3-ONO	3	0.329088	0.246541	-3320.629934	-3320.383393	-3320.300846	-49.7	-7.9	3.4
	1	0.328177	0.248959	-3320.586835	-3320.337876	-3320.258658			

Compounds (MBOX-Y_m-X)	Mult	Thermal correction		SP energy	*ΔG*	*ΔH*	$\Delta_r G$	BDFE	BDE
		to *H*	to *G*						
**CrBOX-Cl2**	5	0.310973	0.240129	-2655.222876	-2654.982747	-2654.911903			
	3	0.310242	0.238757	-2655.162327	-2654.92357	-2654.852085			
	1	0.309739	0.238242	-2655.115223	-2654.876981	-2654.805484			
CrBOX-Cl2-OH	4	0.327176	0.254218	-2731.080964	-2730.826746	-2730.753788	-25.5	48.5	59.9
	2	0.326963	0.255385	-2731.028526	-2730.773141	-2730.701563			
CrBOX-Cl3	4	0.315018	0.241729	-3115.477437	-3115.235708	-3115.162419	-12.2	60.7	70.5
	2	0.315083	0.243398	-3115.425178	-3115.18178	-3115.110095			
CrBOX-Cl2-F	4	0.315351	0.242902	-2755.133311	-2754.890409	-2754.81796	-12.8	87.6	97.3
	2	0.315518	0.244674	-2755.080155	-2754.835481	-2754.764637			
CrBOX-Cl2-N3	4	0.32862	0.252288	-2819.498204	-2819.245916	-2819.169584	-17.8	35.7	48.3
	2	0.328676	0.253164	-2819.445578	-2819.192414	-2819.116902			
CrBOX-Cl2-SCN	4	0.32621	0.247005	-3146.323098	-3146.076093	-3145.996888	-16.6	34.6	46.5
	2	0.326303	0.248783	-3146.270286	-3146.021503	-3145.943983			
CrBOX-Cl2-ONO2	4	0.333177	0.254379	-2935.64164	-2935.387261	-2935.308463	-14.3	43.3	57.6
	2	0.332891	0.254625	-2935.588796	-2935.334171	-2935.255905			
CrBOX-Cl2-Me	4	0.348921	0.274664	-2695.115094	-2694.84043	-2694.766173	-54.1	14.9	28.2
	2	0.34876	0.27417	-2695.061503	-2694.787333	-2694.712743			
CrBOX-Cl2-NO2	4	0.327076	0.249916	-2860.42132	-2860.171404	-2860.094244	-24.0	17.8	31.0
	2	0.326958	0.250035	-2860.369168	-2860.119133	-2860.04221			
CrBOX-Cl2-ONO	4	0.326489	0.248836	-2860.418598	-2860.169762	-2860.092109	-25.0	16.8	29.6
	2	0.326314	0.249712	-2860.365791	-2860.116079	-2860.039477			
**MnBOX-Cl3**	5	0.314489	0.239882	-3221.991934	-3221.752052	-3221.677445			
	3	0.3153	0.243176	-3221.940529	-3221.697353	-3221.625229			
	1	0.313949	0.243946	-3221.917782	-3221.673836	-3221.603833			
MnBOX-Cl3-OH	4	0.330057	0.253997	-3297.808117	-3297.55412	-3297.47806	-51.8	22.2	34.0
	2	0.330488	0.256067	-3297.757396	-3297.501329	-3297.426908			

Compounds (MBOX-Y_m-X)	Mult	Thermal correction		SP energy	*ΔG*	*ΔH*	$\Delta_r G$	BDFE	BDE
		to *H*	to *G*						
MnBOX-Cl4	4	0.318354	0.24198	-3682.193176	-3681.951196	-3681.874822	-46.0	27.0	37.2
	2	0.317797	0.242965	-3682.139052	-3681.896087	-3681.821255			
MnBOX-Cl3-F	4	0.318459	0.243316	-3321.848641	-3321.605325	-3321.530182	-46.9	53.4	63.9
	2	0.318408	0.243615	-3321.794969	-3321.551354	-3321.476561			
MnBOX-Cl3-N3	4	0.331779	0.252456	-3386.218233	-3385.965777	-3385.886454	-48.9	4.7	17.8
	2	0.331828	0.253446	-3386.166227	-3385.912781	-3385.834399			
MnBOX-Cl3-SCN	4	0.329604	0.24868	-3713.045655	-3712.796975	-3712.716051	-47.0	4.2	17.4
	2	0.329735	0.249374	-3712.9888	-3712.739426	-3712.659065			
MnBOX-Cl3-ONO2	4	0.335766	0.252582	-3502.355869	-3502.103287	-3502.020103	-47.7	9.8	23.7
	2	0.335582	0.254852	-3502.304099	-3502.049247	-3501.968517			
MnBOX-Cl3-Me	4	0.352968	0.275873	-3261.860324	-3261.584451	-3261.507356	-70.0	-1.0	12.9
	2	0.353278	0.277462	-3261.803014	-3261.525552	-3261.449736			
MnBOX-Cl3-NO2	4	0.330704	0.250928	-3427.147894	-3426.896966	-3426.81719	-51.4	-9.6	4.2
	2	0.330733	0.252032	-3427.090939	-3426.838907	-3426.760206			
MnBOX-Cl3-ONO	4	0.329183	0.246999	-3427.144463	-3426.897464	-3426.81528	-51.1	-9.3	3.0
	2	0.328905	0.249558	-3427.088911	-3426.839353	-3426.760006			
**MnBOX-Cl2**	6	0.311152	0.238185	-2761.766868	-2761.528683	-2761.455716			
	4	0.309672	0.240993	-2761.700563	-2761.45957	-2761.390891			
	2	0.310978	0.240152	-2761.660182	-2761.42003	-2761.349204			
MnBOX-Cl2-OH	5	0.326525	0.25207	-2837.597554	-2837.345484	-2837.271029	-42.6	31.5	43.2
	3	0.326006	0.255383	-2837.550807	-2837.295424	-2837.224801			
	1	0.32686	0.255175	-2837.521979	-2837.266804	-2837.195119			
MnBOX-Cl3	5	0.314489	0.239882	-3221.991934	-3221.752052	-3221.677445	-30.8	42.2	52.4
	3	0.3153	0.243176	-3221.940529	-3221.697353	-3221.625229			
	1	0.313949	0.243946	-3221.917782	-3221.673836	-3221.603833			
MnBOX-Cl2-F	5	0.314719	0.240523	-2861.64194	-2861.401417	-2861.327221	-34.7	65.7	75.7

Compounds (MBOX-Y$_m$-X)	Mult	Thermal correction		SP energy	*ΔG*	*ΔH*	$\Delta_r G$	BDFE	BDE
		to *H*	to *G*						
	3	0.315356	0.243059	-2861.597482	-2861.354423	-2861.282126			
	1	0.31498	0.241653	-2861.574408	-2861.332755	-2861.259428			
MnBOX-Cl2-N3	5	0.328002	0.250735	-2926.010563	-2925.759828	-2925.682561	-37.9	15.6	29.0
	3	0.32867	0.252481	-2925.963733	-2925.711252	-2925.635063			
	1	0.327623	0.251196	-2925.93229	-2925.681094	-2925.604667			
MnBOX-Cl2-SCN	5	0.325527	0.244982	-3252.839541	-3252.594559	-3252.514014	-33.9	17.4	29.7
	3	0.326318	0.247274	-3252.791013	-3252.543739	-3252.464695			
	1	0.326563	0.250413	-3252.768791	-3252.518378	-3252.442228			
MnBOX-Cl2-ONO2	5	0.332689	0.253792	-3042.144946	-3041.891154	-3041.812257	-40.7	16.9	32.4
	3	0.332877	0.254228	-3042.104221	-3041.849993	-3041.771344			
	1	0.331374	0.254315	-3042.081795	-3041.82748	-3041.750421			
MnBOX-Cl2-Me	5	0.348589	0.272522	-2801.64234	-2801.369818	-2801.293751	-64.5	4.5	18.0
	3	0.349008	0.274824	-2801.58738	-2801.312556	-2801.238372			
	1	0.348996	0.275593	-2801.556335	-2801.280742	-2801.207339			
MnBOX-Cl2-NO2	5	0.326726	0.249448	-2966.937697	-2966.688249	-2966.610971	-42.2	-0.4	14.0
	3	0.327457	0.251675	-2966.886995	-2966.63532	-2966.559538			
	1	0.327539	0.252499	-2966.866123	-2966.613624	-2966.538584			
MnBOX-Cl2-ONO	5	0.325546	0.246726	-2966.928879	-2966.682153	-2966.603333	-46.1	-4.2	9.2
	3	0.326244	0.24911	-2966.883388	-2966.634278	-2966.557144			
	1	0.325481	0.249145	-2966.861275	-2966.61213	-2966.535794			
**FeBOX-Cl3**	6	0.31444	0.240154	-3334.68374	-3334.443586	-3334.3693			
	4	0.31448	0.240079	-3334.656445	-3334.416366	-3334.341965			
	2	0.314952	0.241959	-3334.635195	-3334.393236	-3334.320243			
FeBOX-Cl3-OH	5	0.329563	0.251866	-3410.477073	-3410.225207	-3410.14751	-64.7	9.4	20.0
	3	0.330625	0.255454	-3410.467782	-3410.212328	-3410.137157			
	1	0.329964	0.253863	-3410.447461	-3410.193598	-3410.117497			

Compounds (MBOX-Y_m-X)	Mult	Thermal correction		SP energy	*ΔG*	*ΔH*	$\Delta_r G$	BDFE	BDE
		to *H*	to *G*						
FeBOX-Cl4	5	0.317792	0.238966	-3794.854404	-3794.615438	-3794.536612	-63.1	9.8	18.3
	3	0.317896	0.242594	-3794.849483	-3794.606889	-3794.531587			
	1	0.318022	0.244368	-3794.828659	-3794.584291	-3794.510637			
FeBOX-Cl3-F	5	0.317812	0.239989	-3434.5092	-3434.269211	-3434.191388	-64.2	36.1	44.7
	3	0.319094	0.245737	-3434.503397	-3434.25766	-3434.184303			
	1	0.319011	0.246263	-3434.481364	-3434.235101	-3434.162353			
FeBOX-Cl3-N3	5	0.33046	0.248296	-3498.889628	-3498.641332	-3498.559168	-58.9	-5.3	5.8
	3	0.331921	0.253676	-3498.877413	-3498.623737	-3498.545492			
	1	0.331971	0.253817	-3498.857756	-3498.603939	-3498.525785			
FeBOX-Cl3-SCN	5	0.328577	0.244954	-3825.72385	-3825.478896	-3825.395273	-53.0	-1.8	9.4
	3	0.329845	0.249324	-3825.704637	-3825.455313	-3825.374792			
	1	0.329812	0.250643	-3825.683714	-3825.433071	-3825.353902			
FeBOX-Cl3-ONO2	5	0.334302	0.250019	-3615.022874	-3614.772855	-3614.688572	-61.5	-3.9	9.1
	3	0.336072	0.255837	-3615.012255	-3614.756418	-3614.676183			
	1	0.33595	0.255787	-3614.983258	-3614.727471	-3614.647308			
FeBOX-Cl3-Me	5	0.352046	0.272599	-3374.548786	-3374.276187	-3374.19674	-69.9	-0.9	11.3
	3	0.353333	0.277066	-3374.526113	-3374.249047	-3374.17278			
	1	0.353476	0.278354	-3374.504022	-3374.225668	-3374.150546			
FeBOX-Cl3-NO2	5	0.330096	0.246287	-3539.836835	-3539.590548	-3539.506739	-50.1	-8.3	2.8
	3	0.330401	0.250766	-3539.80924	-3539.558474	-3539.478839			
	1	0.330809	0.252717	-3539.785965	-3539.533248	-3539.455156			
FeBOX-Cl3-ONO	5	0.3296	0.244004	-3539.836694	-3539.59269	-3539.507094	-48.8	-7.0	3.0
	3	0.329942	0.244874	-3539.804983	-3539.560109	-3539.475041			
	1	0.329019	0.248564	-3539.777921	-3539.529357	-3539.448902			
**FeBOX-Cl2**	5	0.31084	0.236864	-2874.443367	-2874.206503	-2874.132527			
	3	0.310554	0.240777	-2874.394843	-2874.154066	-2874.084289			

Compounds (MBOX-Y_m-X)	Mult	Thermal correction		SP energy	*ΔG*	*ΔH*	$\Delta_r G$	BDFE	BDE
		to *H*	to *G*						
	1	0.311022	0.24171	-2874.382708	-2874.140998	-2874.071686			
FeBOX-Cl2-OH	6	0.326611	0.252143	-2950.292732	-2950.040589	-2949.966121	-31.8	42.3	54.7
	4	0.327244	0.25461	-2950.262648	-2950.008038	-2949.935404			
	2	0.327298	0.255731	-2950.245152	-2949.989421	-2949.917854			
FeBOX-Cl3	6	0.31444	0.240154	-3334.68374	-3334.443586	-3334.3693	-22.2	50.8	61.9
	4	0.31448	0.240079	-3334.656445	-3334.416366	-3334.341965			
	2	0.314952	0.241959	-3334.635195	-3334.393236	-3334.320243			
FeBOX-Cl2-F	6	0.315339	0.242087	-2974.33961	-2974.097523	-2974.024271	-23.2	77.1	88.4
	4	0.315533	0.243475	-2974.308411	-2974.064936	-2973.992878			
	2	0.315665	0.244047	-2974.285941	-2974.041894	-2973.970276			
FeBOX-Cl2-N3	6	0.328246	0.249179	-3038.704302	-3038.455123	-3038.376056	-27.0	26.6	39.5
	4	0.32842	0.251031	-3038.676235	-3038.425204	-3038.347815			
	2	0.328081	0.252385	-3038.659527	-3038.407142	-3038.331446			
FeBOX-Cl2-SCN	6	0.325871	0.245553	-3365.529823	-3365.28427	-3365.203952	-26.4	24.9	38.0
	4	0.326214	0.248298	-3365.504939	-3365.256641	-3365.178725			
	2	0.32642	0.249199	-3365.486331	-3365.237132	-3365.159911			
FeBOX-Cl2-ONO2	6	0.332791	0.253316	-3154.854722	-3154.601406	-3154.521931	-20.3	37.2	53.1
	4	0.332157	0.252863	-3154.821391	-3154.568528	-3154.489234			
	2	0.333693	0.25757	-3154.799489	-3154.541919	-3154.465796			
FeBOX-Cl2-Me	6	0.348131	0.271243	-2914.322313	-2914.05107	-2913.974182	-62.4	6.7	20.3
	4	0.349135	0.273975	-2914.311027	-2914.037052	-2913.961892			
	2	0.349836	0.277781	-2914.289987	-2914.012206	-2913.940151			
FeBOX-Cl2-NO2	6	0.326102	0.245705	-3079.62482	-3079.379115	-3079.298718	-34.0	7.8	20.8
	4	0.326547	0.248754	-3079.605233	-3079.356479	-3079.278686			
	2	0.327485	0.252566	-3079.589144	-3079.336578	-3079.261659			
FeBOX-Cl2-ONO	6	0.325441	0.244493	-3079.61902	-3079.374527	-3079.293579	-36.9	4.9	17.6
	4	0.325868	0.247754	-3079.594088	-3079.346334	-3079.26822			

Compounds (MBOX-Y_m-X)	Mult	Thermal correction		SP energy	*ΔG*	*ΔH*	$Δ_rG$	BDFE	BDE
		to *H*	to *G*						
	2	0.326691	0.250821	-3079.579003	-3079.328182	-3079.252312			
**CoBOX-Cl2**	4	0.311522	0.239642	-2993.490438	-2993.250796	-2993.178916			
	2	0.311339	0.242641	-2993.467988	-2993.225347	-2993.156649			
CoBOX-Cl2-OH	5	0.326305	0.250716	-3069.303968	-3069.053252	-3068.977663			
	3	0.327574	0.254627	-3069.308421	-3069.053794	-3068.980847	-51.3	22.8	34.8
	1	0.327782	0.257583	-3069.298521	-3069.040938	-3068.970739			
CoBOX-Cl3	5	0.31434	0.23958	-3453.692279	-3453.452699	-3453.377939			
	3	0.314967	0.242576	-3453.695197	-3453.452621	-3453.38023	-44.3	28.6	39.6
	1	0.314898	0.242875	-3453.69431	-3453.451435	-3453.379412			
CoBOX-Cl2-F	5	0.314979	0.240883	-3093.335031	-3093.094148	-3093.020052			
	3	0.31567	0.244221	-3093.343295	-3093.099074	-3093.027625	-50.0	50.3	61.4
	1	0.315557	0.245641	-3093.342185	-3093.096544	-3093.026628			
CoBOX-Cl2-N3	5	0.327861	0.250421	-3157.711247	-3157.460826	-3157.383386			
	3	0.328424	0.25146	-3157.719523	-3157.468063	-3157.391099	-46.6	6.9	19.8
	1	0.328793	0.254308	-3157.716322	-3157.462014	-3157.387529			
CoBOX-Cl2-SCN	5	0.326055	0.247553	-3484.54444	-3484.296887	-3484.218385			
	3	0.326416	0.247561	-3484.549652	-3484.302091	-3484.223236	-43.0	8.2	20.9
	1	0.326896	0.250465	-3484.551203	-3484.300738	-3484.224307			
CoBOX-Cl2-ONO2	5	0.332156	0.253357	-3273.851699	-3273.598342	-3273.519543			
	3	0.332969	0.255313	-3273.855001	-3273.599688	-3273.522032			
	1	0.333328	0.25774	-3273.859742	-3273.602002	-3273.526414	-47.8	9.8	26.8
CoBOX-Cl2-Me	5	0.347296	0.269603	-3033.338957	-3033.069354	-3032.991661			
	3	0.349247	0.274252	-3033.355998	-3033.081746	-3033.006751	-70.9	-1.9	11.6
	1	0.350034	0.278367	-3033.353873	-3033.075506	-3033.003839			
CoBOX-Cl2-NO2	5	dissociates							
	3	0.327026	0.250364	-3198.648053	-3198.397689	-3198.321027			

Compounds (MBOX-Y$_m$-X)	Mult	Thermal correction		SP energy	*ΔG*	*ΔH*	*Δ$_r$G*	BDFE	BDE
		to *H*	to *G*						
	1	0.328135	0.254546	-3198.654712	-3198.400166	-3198.326577	-48.6	-6.8	9.2
CoBOX-Cl2-ONO	5	dissociates							
	3	dissociates							
	1	0.326538	0.251773	-3198.638941	-3198.387168	-3198.312403	-56.8	-15.0	0.3
**NiBOX-Cl3**	4	0.314355	0.237161	-3579.212632	-3578.975471	-3578.898277			
	2	0.315042	0.242337	-3579.233278	-3578.990941	-3578.918236			
NiBOX-Cl3-OH	5	0.329234	0.249787	-3654.994493	-3654.744706	-3654.665259			
	3	0.32953	0.251738	-3655.017576	-3654.765838	-3654.688046			
	1	0.330415	0.256395	-3655.028918	-3654.772523	-3654.698503	-64.7	9.4	21.3
NiBOX-Cl4	5	0.31743	0.237533	-4039.383291	-4039.145758	-4039.065861			
	3	0.316651	0.237847	-4039.393689	-4039.155842	-4039.077038			
	1	0.318028	0.244139	-4039.420021	-4039.175882	-4039.101993	-54.9	18.0	28.6
NiBOX-Cl3-F	5	0.316978	0.240808	-3679.026609	-3678.785801	-3678.709631			
	3	0.317709	0.241122	-3679.029788	-3678.788666	-3678.712079			
	1	0.318505	0.245288	-3679.063209	-3678.817921	-3678.744704	-63.4	36.9	47.4
NiBOX-Cl3-N3	5	0.330315	0.245748	-3743.41704	-3743.171292	-3743.086725			
	3	0.330992	0.248614	-3743.431425	-3743.182811	-3743.100433			
	1	0.331983	0.254639	-3743.447871	-3743.193232	-3743.115888	-56.0	-2.5	10.7
NiBOX-Cl3-SCN	5	0.328361	0.242658	-4070.250811	-4070.008153	-4069.92245			
	3	0.328838	0.244997	-4070.266848	-4070.021851	-4069.93801			
	1	0.329303	0.24992	-4070.280405	-4070.030485	-4069.951102	-50.4	0.9	13.8
NiBOX-Cl3-ONO2	5	0.334459	0.249598	-3859.54719	-3859.297592	-3859.212731			
	3	0.33445	0.250767	-3859.563692	-3859.312925	-3859.229242			
	1	0.336271	0.257204	-3859.582776	-3859.325572	-3859.246505	-58.2	-0.6	14.7
NiBOX-Cl3-Me	5	dissociates							
	3	0.352775	0.275006	-3619.07935	-3618.804344	-3618.726575			

Compounds (MBOX-Y_m-X)	Mult	Thermal correction		SP energy	*ΔG*	*ΔH*	$\Delta_r G$	BDFE	BDE
		to *H*	to *G*						
	1	0.353752	0.279331	-3619.110181	-3618.83085	-3618.756429	-65.3	3.7	18.1
NiBOX-Cl3-NO2	5	dissociates							
	3	0.330295	0.248466	-3784.367643	-3784.119177	-3784.037348			
	1	0.330869	0.25302	-3784.389204	-3784.136184	-3784.058335	-51.2	-9.4	4.4
NiBOX-Cl3-ONO	5	dissociates							
	3	dissociates							
	1	0.329904	0.250829	-3784.379581	-3784.128752	-3784.049677	-55.9	-14.1	-1.0
**NiBOX-Cl2**	3	0.31179	0.240848	-3119.029321	-3118.788473	-3118.717531			
	1	0.311482	0.240663	-3118.998443	-3118.75778	-3118.686961			
NiBOX-Cl2-OH	4	0.325362	0.25276	-3194.82845	-3194.57569	-3194.503088			
	2	0.32734	0.254476	-3194.83989	-3194.585414	-3194.51255	-55.1	19.0	30.5
NiBOX-Cl3	4	0.314355	0.237161	-3579.212632	-3578.975471	-3578.898277			
	2	0.315042	0.242337	-3579.233278	-3578.990941	-3578.918236	-43.9	29.0	39.3
NiBOX-Cl2-F	4	0.31507	0.241052	-3218.857654	-3218.616602	-3218.542584			
	2	0.315035	0.24181	-3218.876659	-3218.634849	-3218.561624	-51.2	49.1	58.5
NiBOX-Cl2-N3	4	0.327911	0.246889	-3283.240841	-3282.993952	-3282.91293			
	2	0.327886	0.249656	-3283.255546	-3283.00589	-3282.92766	-46.5	7.0	18.5
NiBOX-Cl2-SCN	4	dissociates							
	2	0.326409	0.248816	-3610.092215	-3609.843399	-3609.765806	-40.7	10.5	23.4
NiBOX-Cl2-ONO2	4	0.331599	0.249546	-3399.374048	-3399.124502	-3399.042449			
	2	0.33258	0.253418	-3399.395494	-3399.142076	-3399.062914	-46.3	11.3	25.4
NiBOX-Cl2-Me	4	0.346428	0.270676	-3158.883754	-3158.613078	-3158.537326			
	2	0.349674	0.274878	-3158.906339	-3158.631461	-3158.556665	-63.4	5.7	18.7
NiBOX-Cl2-NO2	4	dissociates							
	2	0.327394	0.250799	-3324.196586	-3323.945787	-3323.869192	-43.6	-1.8	11.7
NiBOX-Cl2-ONO	4	dissociates							

Compounds (MBOX-Y_m-X)	Mult	Thermal correction		SP energy	*ΔG*	*ΔH*	$\Delta_r G$	BDFE	BDE
		to *H*	to *G*						
	2	dissociates							
**CuBOX-Cl2**	2	0.310878	0.239488	-3251.166013	-3250.926525	-3250.855135			
CuBOX-Cl2-OH	3	0.32647	0.252261	-3326.962919	-3326.710658	-3326.636449			
	1	0.326514	0.252834	-3326.971267	-3326.718433	-3326.644753	-58.2	15.9	27.1
CuBOX-Cl3	3	0.31417	0.239065	-3711.358316	-3711.119251	-3711.044146			
	1	0.314368	0.240969	-3711.364812	-3711.123843	-3711.050444	-47.1	25.8	35.9
CuBOX-Cl2-F	3	0.314843	0.241696	-3351.000151	-3350.758455	-3350.685308			
	1	0.314814	0.241557	-3351.004593	-3350.763036	-3350.689779	-57.4	42.9	52.5
CuBOX-Cl2-N3	3	0.327555	0.247317	-3415.387303	-3415.139986	-3415.059748			
	1	0.327617	0.250006	-3415.391254	-3415.141248	-3415.063637	-48.2	5.3	17.5
CuBOX-Cl2-SCN	3	0.32526	0.242768	-3742.216896	-3741.974128	-3741.891636			
	1	0.325474	0.24615	-3742.22365	-3741.9775	-3741.898176	-43.2	8.0	20.1
CuBOX-Cl2-ONO2	3	0.331158	0.249975	-3531.522674	-3531.272699	-3531.191516			
	1	0.3321	0.253135	-3531.524597	-3531.271462	-3531.192497	-51.7	5.9	20.4
CuBOX-Cl2-Me	3	dissociates							
	1	0.349171	0.273876	-3291.049948	-3290.776072	-3290.700777	-59.2	9.8	22.8
CuBOX-Cl2-NO2	3	dissociates							
	1	0.326565	0.248867	-3456.333745	-3456.084878	-3456.00718	-43.0	-1.2	11.9
CuBOX-Cl2-ONO	3	dissociates							
	1	dissociates							
**CuBOX-Cl**	3	0.303711	0.236255	-2790.849813	-2790.613558	-2790.546102			
	1	0.307213	0.238628	-2790.936429	-2790.697801	-2790.629216			
CuBOX-Cl -OH	2	0.323032	0.251697	-2866.76735	-2866.515653	-2866.444318	-41.9	85.0	95.3
CuBOX-Cl2	2	0.310878	0.239488	-3251.166013	-3250.926525	-3250.855135	-27.4	98.4	107.2
CuBOX-Cl -F	2	0.311516	0.242749	-2890.824742	-2890.581993	-2890.513226	-27.5	125.7	135.7

Compounds (MBOX-Y_m-X)	Mult	Thermal correction		SP energy	*ΔG*	*ΔH*	$\Delta_r G$	BDFE	BDE
		to *H*	to *G*						
CuBOX-Cl -N3	2	0.324399	0.247226	-2955.198921	-2954.951695	-2954.874522	-23.7	82.8	92.7
CuBOX-Cl -SCN	2	0.322452	0.247256	-3282.02789	-3281.780634	-3281.705438	-23.2	80.9	93.1
CuBOX-Cl -ONO2	2	0.328985	0.251858	-3071.346526	-3071.094668	-3071.017541	-19.1	91.3	104.5
CuBOX-Cl -Me	2	0.345282	0.27269	-2830.816732	-2830.544042	-2830.47145	-61.3	60.6	72.8
CuBOX-Cl -NO2	2	0.32281	0.248064	-2996.125198	-2995.877134	-2995.802388	-29.8	64.8	77.4
CuBOX-Cl -ONO	2	0.322153	0.244881	-2996.116723	-2995.871842	-2995.79457	-33.1	85.0	95.3
**ZnBOX-Cl**	2	0.306747	0.23889	-2929.749852	-2929.510962	-2929.443105			
ZnBOX-Cl -OH	1	0.322948	0.25132	-3005.670875	-3005.419555	-3005.347927	15.0	89.1	99.4
ZnBOX-Cl2	1	0.311389	0.239939	-3390.08809	-3389.848151	-3389.776701	40.7	113.6	122.6
ZnBOX-Cl -F	1	0.311215	0.239173	-3029.729722	-3029.490549	-3029.418507	32.4	132.7	140.9
ZnBOX-Cl -N3	1	0.32496	0.249549	-3094.097353	-3093.847804	-3093.772393	28.4	81.9	93.3
ZnBOX-Cl -SCN	1	0.322801	0.246925	-3420.93368	-3420.686755	-3420.610879	35.1	86.4	98.4
ZnBOX-Cl -ONO2	1	0.328954	0.251253	-3210.24643	-3209.995177	-3209.917476	35.7	93.3	106.4
ZnBOX-Cl -Me	1	0.345499	0.273553	-2969.717086	-2969.443533	-2969.371587	-7.2	61.9	74.8
ZnBOX-Cl -NO2	1	0.323191	0.248511	-3135.018421	-3134.76991	-3134.69523	20.1	61.9	74.7
ZnBOX-Cl -ONO	1	0.322327	0.245934	-3135.018459	-3134.772525	-3134.696132	21.8	89.1	99.4

1.3. Computed structures

iPr-OH (m1)

C	2.122574000	-0.780974000	0.957122000
H	3.145343000	-0.371871000	0.880746000
C	1.409059000	-0.090916000	2.101052000
H	1.920665000	-0.257532000	3.060196000
H	1.351038000	0.993459000	1.932176000
H	0.380012000	-0.471338000	2.205756000
C	2.213798000	-2.280115000	1.152577000
H	1.208762000	-2.726582000	1.225860000
H	2.732485000	-2.757973000	0.309690000
H	2.755810000	-2.538783000	2.073833000
O	1.520215000	-0.461221000	-0.291410000
H	0.616370000	-0.803356000	-0.258724000

iPr-Cl (m1)

C	2.132946000	-0.792828000	0.992404000
H	3.135117000	-0.363048000	0.850801000
C	1.407747000	-0.087722000	2.105312000
H	1.933498000	-0.269439000	3.054689000
H	1.366715000	0.997611000	1.947089000
H	0.380236000	-0.462669000	2.213839000
C	2.215623000	-2.285252000	1.157353000
H	1.215826000	-2.735295000	1.235239000
H	2.745514000	-2.761925000	0.323089000
H	2.763008000	-2.521443000	2.082035000
Cl	1.279896000	-0.421329000	-0.602895000

iPr-F (m1)

C	2.147486000	-0.787409000	0.998398000
H	3.164834000	-0.370208000	0.896368000
C	1.409872000	-0.085222000	2.103272000
H	1.920173000	-0.234192000	3.064419000
H	1.354770000	0.995264000	1.915473000
H	0.385253000	-0.473038000	2.199215000
C	2.214708000	-2.281115000	1.148778000
H	1.206118000	-2.713890000	1.220807000
H	2.728326000	-2.742143000	0.294584000
H	2.765672000	-2.553282000	2.059121000
F	1.490538000	-0.499152000	-0.216472000

iPr-N3 (m1)

C	2.219347000	-0.737196000	0.898484000
H	3.252995000	-0.382748000	1.019046000
C	1.333579000	-0.070355000	1.928006000
H	1.653988000	-0.345966000	2.942022000
H	1.371845000	1.024203000	1.846875000
H	0.284740000	-0.386102000	1.815818000
C	2.191385000	-2.248245000	0.975035000
H	1.171329000	-2.635103000	0.826531000
H	2.844255000	-2.702661000	0.217821000
H	2.533077000	-2.586763000	1.962794000
N	1.865408000	-0.276506000	-0.478266000
N	0.743873000	-0.521935000	-0.886867000
N	-0.277188000	-0.691727000	-1.381474000

iPr-SCN (m1)

C	1.992062000	-1.080504000	0.818864000
H	2.864741000	-0.608876000	0.341672000
C	1.303752000	-0.121179000	1.753021000
H	1.993897000	0.137356000	2.569741000
H	1.019322000	0.816218000	1.255958000
H	0.404213000	-0.565134000	2.202656000
C	2.383358000	-2.391169000	1.458683000
H	1.514496000	-2.911718000	1.887153000
H	2.885667000	-3.069838000	0.756632000
H	3.082515000	-2.188168000	2.281780000
S	0.871680000	-1.549375000	-0.625958000
C	0.557160000	-0.036636000	-1.270760000
N	0.315770000	1.007920000	-1.743618000

iPr-ONO2 (m1)

C	2.051969000	-0.815442000	1.057403000
H	3.013832000	-0.308414000	0.885481000
C	1.396264000	-0.208508000	2.272114000
H	2.091164000	-0.282419000	3.119448000
H	1.182484000	0.857097000	2.114503000
H	0.468952000	-0.719713000	2.557110000
C	2.293292000	-2.302650000	1.120079000
H	1.387345000	-2.873206000	1.358318000
H	2.709939000	-2.676551000	0.175364000
H	3.030928000	-2.505707000	1.908103000
O	1.391895000	-0.407509000	-0.194806000
N	0.087451000	-0.836690000	-0.415045000
O	-0.446359000	-1.526277000	0.417604000
O	-0.357092000	-0.443258000	-1.460276000

iPr-Me (m1)

C	2.111082000	-0.754789000	0.957274000
H	3.117310000	-0.295373000	0.925392000
C	1.359270000	-0.142431000	2.128538000
H	1.872080000	-0.323478000	3.085366000
H	1.245063000	0.946526000	2.018198000
H	0.345977000	-0.567909000	2.216901000
C	2.292146000	-2.252858000	1.143395000
H	1.318566000	-2.768748000	1.182478000
H	2.864790000	-2.702991000	0.318373000
H	2.822279000	-2.489425000	2.078457000
C	1.419749000	-0.445227000	-0.361158000
H	1.970713000	-0.857329000	-1.219994000
H	0.405168000	-0.875792000	-0.388515000
H	1.317297000	0.638074000	-0.525641000

iPr-NO2 (m1)

C	2.129218000	-0.800380000	1.054378000
H	3.082210000	-0.274533000	0.886001000
C	1.424559000	-0.207033000	2.253776000
H	2.014796000	-0.407353000	3.156846000
H	1.308730000	0.878950000	2.156933000
H	0.431284000	-0.657768000	2.391976000
C	2.337121000	-2.286527000	1.126340000
H	1.389378000	-2.821526000	1.272614000
H	2.819986000	-2.678051000	0.222707000
H	2.987749000	-2.513648000	1.980951000
N	1.323074000	-0.425518000	-0.190263000

O	0.845062000	-1.315290000	-0.869844000
O	1.200524000	0.764686000	-0.422931000

iPr-ONO (m1)

C	2.105026000	-0.778763000	1.033675000
H	3.081243000	-0.288390000	0.902260000
C	1.400484000	-0.193499000	2.231770000
H	2.003035000	-0.355946000	3.135402000
H	1.251744000	0.888646000	2.117022000
H	0.420280000	-0.663477000	2.394356000
C	2.291160000	-2.274508000	1.085870000
H	1.331226000	-2.795967000	1.207823000
H	2.775575000	-2.647165000	0.173330000
H	2.929288000	-2.542958000	1.938373000
O	1.425457000	-0.390226000	-0.205139000
N	0.137784000	-0.895812000	-0.256214000
O	-0.403878000	-0.584906000	-1.260733000

iPr-rad (m2)

C	2.215964000	-0.803603000	1.102634000
H	3.007437000	-0.276022000	0.555777000
C	1.411888000	-0.043324000	2.085404000
H	1.837553000	-0.088008000	3.108744000
H	1.333341000	1.023980000	1.833314000
H	0.388287000	-0.444180000	2.180126000
C	2.230548000	-2.283436000	1.105357000
H	1.218283000	-2.706299000	1.221926000
H	2.667510000	-2.704306000	0.188141000
H	2.820736000	-2.701493000	1.946421000

OH-rad (m2)

O	0.278308000	0.000000000	-3.979455000
H	0.278308000	0.000000000	-4.958717000

Cl-rad (m2)

Cl	0.278308000	0.000000000	-2.289086000

F-rad (m2)

F	0.278308000	0.000000000	-3.989086000

N3-rad (m2)

N	0.321978000	0.000000000	-6.631076000
N	0.321978000	0.000000000	-7.810734000
N	0.321978000	0.000000000	-5.451417000

SCN-rad (m2)

S	-0.536298000	0.000000000	-3.786551000
C	-0.536298000	0.000000000	-5.401790000
N	-0.536298000	0.000000000	-6.592599000

ONO2-rad (m2)

O	-0.648296000	0.044527000	-2.058848000
N	-0.656243000	0.046175000	-0.829957000
O	0.358072000	-0.263433000	-0.207839000
O	-1.677886000	0.357248000	-0.221375000

Me-rad (m2)

C	-0.131008000	-0.014325000	-1.624192000
H	-1.182980000	-0.168856000	-1.380241000
H	0.415411000	-0.774242000	-2.184521000
H	0.374377000	0.899446000	-1.308391000

NO2-rad (m2)

N	-3.200546000	0.000000000	-2.354014000
O	-3.200546000	-1.098481000	-1.890592000
O	-3.200546000	1.098481000	-1.890592000

1.3.1. Porphyrin complexes

FePorph-SMe (m6)

C	2.774435000	-1.372371000	0.028926000
C	4.179578000	-1.184571000	-0.223740000
C	4.358030000	0.141417000	-0.499364000
C	3.062199000	0.764218000	-0.411048000
N	2.117983000	-0.175267000	-0.091987000
C	2.170203000	-2.595588000	0.329378000
C	2.805483000	2.121756000	-0.614350000
C	1.560212000	2.752066000	-0.545652000
C	1.324315000	4.153531000	-0.772670000
C	-0.017633000	4.360633000	-0.615096000
C	-0.602403000	3.083238000	-0.299679000
N	0.376739000	2.124745000	-0.257985000
C	-1.965316000	2.848190000	-0.100964000
C	-2.576180000	1.614682000	0.139038000
C	-3.992208000	1.409152000	0.292841000
C	-4.179408000	0.070420000	0.492251000
C	-2.876360000	-0.541960000	0.466413000
N	-1.919931000	0.414786000	0.244920000
C	-2.621637000	-1.901707000	0.658670000
C	-1.369574000	-2.521456000	0.654684000
C	-1.136790000	-3.928333000	0.846184000
C	0.214063000	-4.121520000	0.766024000
C	0.806605000	-2.832391000	0.521285000
N	-0.174669000	-1.876384000	0.467471000
H	4.926369000	-1.977271000	-0.198312000
H	5.281736000	0.664079000	-0.745124000
H	2.830716000	-3.463156000	0.393902000
H	3.662618000	2.752905000	-0.859302000
H	2.095830000	4.880296000	-1.024082000
H	-0.574696000	5.291600000	-0.713672000
Fe	0.139233000	0.189160000	0.493053000
H	-2.625710000	3.716127000	-0.161186000
H	-4.742357000	2.197672000	0.246241000
H	-5.114608000	-0.466647000	0.646389000
H	-3.487980000	-2.545016000	0.828279000
H	-1.916817000	-4.670088000	1.012786000
H	0.770001000	-5.054414000	0.851913000
S	0.417720000	0.583008000	2.771940000
C	-0.812306000	-0.526674000	3.506227000
H	-1.827240000	-0.309831000	3.140875000
H	-0.582279000	-1.582653000	3.298629000
H	-0.812695000	-0.390192000	4.596548000

FePorph-SMe-OH (m3)

C	2.730662000	-1.370107000	0.092486000
C	4.136003000	-1.175976000	-0.140233000

C	4.296310000	0.125102000	-0.515564000
C	2.990279000	0.728240000	-0.492426000
N	2.054282000	-0.198881000	-0.126304000
C	2.149908000	-2.583069000	0.440546000
C	2.737937000	2.069758000	-0.753309000
C	1.500880000	2.689912000	-0.627821000
C	1.282318000	4.101779000	-0.790983000
C	-0.032342000	4.332094000	-0.509869000
C	-0.618411000	3.056837000	-0.196637000
N	0.330593000	2.072648000	-0.274088000
C	-1.961375000	2.859231000	0.097167000
C	-2.557671000	1.619698000	0.294925000
C	-3.969231000	1.416655000	0.472605000
C	-4.166866000	0.069449000	0.550253000
C	-2.872584000	-0.547264000	0.443932000
N	-1.907719000	0.414319000	0.290098000
C	-2.644951000	-1.913026000	0.548688000
C	-1.391799000	-2.514901000	0.564538000
C	-1.161688000	-3.912732000	0.805379000
C	0.190590000	-4.092538000	0.821374000
C	0.784045000	-2.807668000	0.568459000
N	-0.196551000	-1.862387000	0.421152000
H	4.890217000	-1.954338000	-0.034267000
H	5.211821000	0.652765000	-0.779484000
H	2.815126000	-3.436621000	0.582008000
H	3.586763000	2.694958000	-1.035712000
H	2.055089000	4.814361000	-1.075995000
H	-0.579016000	5.273999000	-0.519097000
Fe	0.058101000	0.106004000	0.058023000
H	-2.606886000	3.738725000	0.124728000
H	-4.704907000	2.218579000	0.515163000
H	-5.099716000	-0.477704000	0.679126000
H	-3.514957000	-2.559901000	0.674663000
H	-1.949822000	-4.649769000	0.951799000
H	0.755351000	-5.010574000	0.977948000
O	-0.158071000	-0.209481000	-1.730055000
S	0.429105000	0.466528000	2.300056000
H	0.321093000	-1.024218000	-1.952243000
C	-0.843292000	-0.427179000	3.201800000
H	-1.854914000	-0.077632000	2.944805000
H	-0.784866000	-1.511996000	3.024431000
H	-0.683717000	-0.252731000	4.275481000

FePorph-SMe-Cl (m3)

C	2.750706000	-1.366632000	0.099314000
C	4.157191000	-1.169715000	-0.108843000
C	4.325097000	0.140342000	-0.446743000
C	3.021449000	0.744828000	-0.429154000
N	2.073610000	-0.189684000	-0.103969000
C	2.173709000	-2.582359000	0.432885000
C	2.781149000	2.090136000	-0.665226000
C	1.538937000	2.701384000	-0.562022000
C	1.311790000	4.107895000	-0.737028000
C	-0.017442000	4.323723000	-0.516590000
C	-0.601229000	3.046200000	-0.221458000
N	0.363472000	2.069418000	-0.251842000
C	-1.951387000	2.841838000	0.021465000
C	-2.543310000	1.604950000	0.237878000
C	-3.954843000	1.402900000	0.401368000
C	-4.147859000	0.058942000	0.531491000
C	-2.850964000	-0.554772000	0.463208000
N	-1.886932000	0.402167000	0.285129000
C	-2.620183000	-1.916421000	0.601277000
C	-1.364990000	-2.507577000	0.617115000
C	-1.130338000	-3.903298000	0.851248000
C	0.222162000	-4.084706000	0.837812000
C	0.810504000	-2.801871000	0.577992000
N	-0.170318000	-1.850950000	0.454870000
H	4.907639000	-1.952219000	-0.008632000
H	5.244632000	0.674451000	-0.681538000
H	2.838677000	-3.437876000	0.560880000
H	3.635470000	2.720173000	-0.917345000
H	2.088451000	4.827387000	-0.991837000
H	-0.574581000	5.258534000	-0.554369000
Fe	0.091090000	0.102165000	0.083628000
H	-2.603613000	3.716435000	0.009248000
H	-4.692903000	2.203560000	0.402999000
H	-5.079125000	-0.488428000	0.669294000
H	-3.486259000	-2.565202000	0.741167000
H	-1.916797000	-4.639590000	1.008872000
H	0.789943000	-5.003173000	0.978833000
Cl	-0.228243000	-0.270466000	-2.178358000
S	0.386202000	0.531895000	2.298792000
C	-0.795197000	-0.463023000	3.208546000
H	-1.835287000	-0.218354000	2.946145000
H	-0.623162000	-1.540232000	3.070779000
H	-0.651706000	-0.229447000	4.273956000

FePorph-SMe-F (m3)

C	2.769016000	-1.350357000	0.206481000
C	4.169178000	-1.155586000	-0.041704000
C	4.323566000	0.143055000	-0.430013000
C	3.019659000	0.744139000	-0.399130000
N	2.085453000	-0.182291000	-0.018343000
C	2.200673000	-2.559496000	0.581712000
C	2.767887000	2.082923000	-0.666935000
C	1.528991000	2.696492000	-0.541262000
C	1.300570000	4.103674000	-0.716536000
C	-0.015795000	4.327811000	-0.437944000
C	-0.592969000	3.054373000	-0.112622000
N	0.363307000	2.073330000	-0.183062000
C	-1.931826000	2.855819000	0.193110000
C	-2.519560000	1.618635000	0.421432000
C	-3.925721000	1.416049000	0.622716000
C	-4.118252000	0.070100000	0.738942000
C	-2.826369000	-0.545630000	0.630238000
N	-1.865294000	0.413041000	0.435861000
C	-2.593769000	-1.907637000	0.763613000
C	-1.337606000	-2.498812000	0.766768000
C	-1.097026000	-3.891215000	1.019616000
C	0.256229000	-4.065798000	1.013041000
C	0.838971000	-2.784252000	0.734171000
N	-0.147176000	-1.841610000	0.593209000
H	4.925128000	-1.932182000	0.062787000
H	5.235086000	0.669622000	-0.708319000
H	2.870546000	-3.408641000	0.724977000

H	3.613889000	2.707523000	-0.957586000
H	2.068708000	4.817911000	-1.008729000
H	-0.568457000	5.265717000	-0.456139000
Fe	0.103009000	0.109939000	0.191998000
H	-2.580779000	3.732659000	0.211960000
H	-4.662897000	2.216790000	0.653284000
H	-5.047205000	-0.476561000	0.892948000
H	-3.458036000	-2.555499000	0.917226000
H	-1.880313000	-4.628613000	1.187418000
H	0.827798000	-4.979433000	1.168369000
F	-0.154500000	-0.212726000	-1.578680000
S	0.488236000	0.546013000	2.392277000
C	-0.779789000	-0.307409000	3.334618000
H	-1.788787000	0.063648000	3.102704000
H	-0.745059000	-1.396191000	3.182685000
H	-0.579626000	-0.106756000	4.397099000

FePorph-SMe-N3 (m3)

C	2.746461000	-1.360643000	0.146290000
C	4.147394000	-1.165163000	-0.098416000
C	4.301022000	0.131099000	-0.494300000
C	2.996588000	0.731509000	-0.467450000
N	2.060648000	-0.195354000	-0.085984000
C	2.176138000	-2.569064000	0.520764000
C	2.747233000	2.071115000	-0.730680000
C	1.512428000	2.691712000	-0.587625000
C	1.290133000	4.101746000	-0.741692000
C	-0.024606000	4.328283000	-0.454840000
C	-0.607680000	3.053455000	-0.148753000
N	0.345729000	2.068086000	-0.233634000
C	-1.951554000	2.852523000	0.134938000
C	-2.546549000	1.611488000	0.320808000
C	-3.957490000	1.403846000	0.478742000
C	-4.152718000	0.055032000	0.550130000
C	-2.857677000	-0.557288000	0.461154000
N	-1.892114000	0.404667000	0.316481000
C	-2.622975000	-1.922371000	0.573229000
C	-1.366178000	-2.508901000	0.618117000
C	-1.131892000	-3.902587000	0.866322000
C	0.221291000	-4.075924000	0.903479000
C	0.811658000	-2.792840000	0.650944000
N	-0.169295000	-1.847385000	0.489892000
H	4.904279000	-1.939837000	0.013331000
H	5.212853000	0.657331000	-0.772134000
H	2.844046000	-3.418022000	0.673729000
H	3.594609000	2.694425000	-1.020207000
H	2.060058000	4.816637000	-1.027383000
H	-0.572857000	5.268884000	-0.459497000
Fe	0.090068000	0.103008000	0.139264000
H	-2.598170000	3.730813000	0.162861000
H	-4.695466000	2.203616000	0.513423000
H	-5.085297000	-0.495141000	0.663953000
H	-3.489152000	-2.575955000	0.686977000
H	-1.919090000	-4.642552000	1.001220000
H	0.787981000	-4.990899000	1.068482000
S	0.387500000	0.555083000	2.340171000
C	-0.803656000	-0.435910000	3.245847000
H	-1.838201000	-0.197259000	2.957360000
H	-0.627632000	-1.513601000	3.115839000
H	-0.685611000	-0.195378000	4.312169000
N	-0.176066000	-0.215185000	-1.816237000
N	-1.005428000	0.425722000	-2.403329000
N	-1.800070000	1.023547000	-2.998361000

FePorph-SMe-SCN (m3)

C	2.771354000	-1.340385000	0.158762000
C	4.175073000	-1.146469000	-0.062640000
C	4.333933000	0.141244000	-0.483002000
C	3.029391000	0.740756000	-0.488874000
N	2.085990000	-0.176955000	-0.101656000
C	2.194394000	-2.549323000	0.514805000
C	2.779296000	2.070380000	-0.793389000
C	1.544450000	2.688683000	-0.657375000
C	1.323376000	4.095658000	-0.821215000
C	0.017968000	4.332120000	-0.499060000
C	-0.565484000	3.065276000	-0.171735000
N	0.379008000	2.067749000	-0.277163000
C	-1.904784000	2.877585000	0.128556000
C	-2.506861000	1.636380000	0.281035000
C	-3.917491000	1.431634000	0.431486000
C	-4.120054000	0.082123000	0.455081000
C	-2.828555000	-0.534070000	0.352044000
N	-1.855460000	0.428010000	0.236422000
C	-2.604326000	-1.900435000	0.437701000
C	-1.349563000	-2.490933000	0.499695000
C	-1.122519000	-3.882694000	0.755011000
C	0.229674000	-4.055701000	0.833685000
C	0.826788000	-2.774668000	0.596512000
N	-0.149376000	-1.828241000	0.399268000
H	4.930224000	-1.918509000	0.075026000
H	5.248985000	0.663573000	-0.757833000
H	2.858934000	-3.396344000	0.691080000
H	3.624783000	2.689454000	-1.096630000
H	2.089808000	4.804090000	-1.131391000
H	-0.524693000	5.276021000	-0.493938000
Fe	0.119383000	0.133875000	0.122293000
H	-2.542734000	3.761023000	0.178227000
H	-4.651043000	2.233873000	0.492108000
H	-5.055557000	-0.467112000	0.548662000
H	-3.474675000	-2.551987000	0.528552000
H	-1.913105000	-4.622132000	0.871339000
H	0.791563000	-4.969245000	1.021696000
S	0.379828000	0.505501000	2.330939000
C	-0.861051000	-0.469931000	3.180209000
H	-1.882891000	-0.186133000	2.887588000
H	-0.714137000	-1.548140000	3.021975000
H	-0.746054000	-0.263650000	4.254523000
S	-0.132590000	-0.389141000	-2.330394000
C	-0.887950000	0.980105000	-2.868225000
N	-1.422258000	1.952259000	-3.267474000

FePorph-SMe-ONO2 (m3)

C	2.748393000	-1.396803000	0.041817000
C	4.154479000	-1.188183000	-0.148999000
C	4.319981000	0.138692000	-0.417408000
C	3.013517000	0.734774000	-0.386072000

N	2.065881000	-0.217106000	-0.112470000
C	2.173506000	-2.623036000	0.340075000
C	2.767657000	2.082850000	-0.595042000
C	1.514413000	2.676157000	-0.542077000
C	1.277139000	4.075664000	-0.750361000
C	-0.066399000	4.273109000	-0.613535000
C	-0.643534000	2.992121000	-0.326638000
N	0.333522000	2.028823000	-0.286570000
C	-1.999408000	2.770696000	-0.134335000
C	-2.571687000	1.535933000	0.125853000
C	-3.975401000	1.329824000	0.325906000
C	-4.149386000	-0.001815000	0.567165000
C	-2.851594000	-0.607587000	0.511537000
N	-1.896352000	0.340121000	0.232977000
C	-2.608507000	-1.952897000	0.732959000
C	-1.353455000	-2.536632000	0.698010000
C	-1.104904000	-3.925013000	0.943226000
C	0.242914000	-4.112755000	0.835124000
C	0.817247000	-2.836926000	0.527193000
N	-0.171340000	-1.880246000	0.441534000
H	4.906289000	-1.972860000	-0.082043000
H	5.238597000	0.687025000	-0.620767000
H	2.837478000	-3.483456000	0.435027000
H	3.622523000	2.725904000	-0.809542000
H	2.056079000	4.802862000	-0.974095000
H	-0.635353000	5.197276000	-0.701595000
Fe	0.073232000	0.060820000	0.107019000
H	-2.666421000	3.631938000	-0.193971000
H	-4.722046000	2.121206000	0.283677000
H	-5.070619000	-0.545933000	0.769452000
H	-3.461913000	-2.594234000	0.957826000
H	-1.880684000	-4.655621000	1.166290000
H	0.817103000	-5.030704000	0.950358000
O	0.045576000	-0.119135000	-1.922765000
S	0.389763000	0.539125000	2.288785000
C	-0.762797000	-0.460837000	3.229009000
H	-1.809984000	-0.225210000	2.987933000
H	-0.584283000	-1.537171000	3.092673000
H	-0.595570000	-0.218712000	4.289003000
N	-0.817020000	-0.750737000	-2.669714000
O	-1.669334000	-1.459339000	-2.155176000
O	-0.696497000	-0.583332000	-3.877441000

FePorph-SMe-Me (m3)

C	2.772872000	-1.343799000	0.076008000
C	4.177984000	-1.150883000	-0.148200000
C	4.346764000	0.156896000	-0.493962000
C	3.045518000	0.765617000	-0.464231000
N	2.094920000	-0.160513000	-0.117118000
C	2.200761000	-2.562717000	0.404058000
C	2.802722000	2.109010000	-0.712612000
C	1.558884000	2.717799000	-0.604365000
C	1.324449000	4.121633000	-0.788560000
C	-0.003722000	4.333689000	-0.557702000
C	-0.581570000	3.056462000	-0.249056000
N	0.390335000	2.079655000	-0.274920000
C	-1.931857000	2.853853000	-0.006856000
C	-2.530925000	1.616741000	0.211416000
C	-3.945536000	1.411365000	0.370354000
C	-4.132621000	0.066702000	0.498689000
C	-2.828901000	-0.538965000	0.434120000
N	-1.870362000	0.423197000	0.263717000
C	-2.588327000	-1.901255000	0.568750000
C	-1.332764000	-2.496756000	0.588357000
C	-1.103649000	-3.897307000	0.816047000
C	0.248141000	-4.080575000	0.803439000
C	0.835737000	-2.792745000	0.550066000
N	-0.138004000	-1.842876000	0.433262000
H	4.927760000	-1.935035000	-0.054372000
H	5.266018000	0.685673000	-0.742090000
H	2.871696000	-3.415316000	0.522819000
H	3.653469000	2.737765000	-0.980086000
H	2.094888000	4.843581000	-1.055575000
H	-0.563800000	5.266819000	-0.597866000
Fe	0.137407000	0.140119000	0.110280000
H	-2.580895000	3.731148000	-0.024244000
H	-4.687764000	2.208402000	0.370882000
H	-5.061948000	-0.484752000	0.634503000
H	-3.453720000	-2.552921000	0.703632000
H	-1.891315000	-4.633007000	0.971418000
H	0.814670000	-5.000493000	0.941420000
S	0.330653000	0.493374000	2.443240000
C	-0.884959000	-0.528343000	3.287253000
H	-1.910471000	-0.292566000	2.960904000
H	-0.704722000	-1.601121000	3.114483000
H	-0.830768000	-0.347462000	4.370477000
C	-0.282263000	-0.283313000	-1.832397000
H	0.655812000	-0.642751000	-2.278879000
H	-1.066607000	-1.047329000	-1.898392000
H	-0.612300000	0.654957000	-2.299241000

FePorph-SMe-NO2 (m3)

C	2.724755000	-1.354968000	0.070337000
C	4.126529000	-1.172521000	-0.173005000
C	4.295257000	0.128691000	-0.544333000
C	2.998284000	0.743693000	-0.499972000
N	2.049212000	-0.175670000	-0.134386000
C	2.146568000	-2.563614000	0.422579000
C	2.760802000	2.086778000	-0.748745000
C	1.526008000	2.703834000	-0.606978000
C	1.301686000	4.108870000	-0.785648000
C	-0.017789000	4.334834000	-0.518959000
C	-0.599452000	3.063931000	-0.200633000
N	0.357809000	2.076838000	-0.251788000
C	-1.946780000	2.871460000	0.060744000
C	-2.546173000	1.634592000	0.250036000
C	-3.959287000	1.438269000	0.394621000
C	-4.162453000	0.092161000	0.485573000
C	-2.868886000	-0.526502000	0.422647000
N	-1.893599000	0.427599000	0.276903000
C	-2.646612000	-1.891036000	0.538794000
C	-1.391350000	-2.481675000	0.570560000
C	-1.161955000	-3.879094000	0.794735000
C	0.190991000	-4.059905000	0.804619000
C	0.782108000	-2.776638000	0.560802000
N	-0.195736000	-1.821132000	0.430412000

H	4.873649000	-1.958723000	-0.076948000
H	5.211727000	0.650445000	-0.815869000
H	2.807731000	-3.421330000	0.555180000
H	3.609176000	2.709086000	-1.037100000
H	2.072553000	4.821252000	-1.075513000
H	-0.570131000	5.272892000	-0.544787000
Fe	0.089701000	0.141850000	0.174608000
H	-2.591253000	3.751768000	0.066949000
H	-4.692609000	2.243133000	0.409124000
H	-5.098557000	-0.452715000	0.597929000
H	-3.516014000	-2.540152000	0.653852000
H	-1.950896000	-4.616777000	0.932090000
H	0.756352000	-4.979629000	0.947160000
S	0.394180000	0.521636000	2.414581000
C	-0.792148000	-0.527959000	3.265416000
H	-1.816795000	-0.353660000	2.904458000
H	-0.549718000	-1.594837000	3.150277000
H	-0.762431000	-0.285738000	4.337219000
N	-0.193566000	-0.211509000	-1.909395000
O	0.349242000	-1.183213000	-2.402383000
O	-0.876265000	0.585105000	-2.527494000

FePorph-SMe-ONO (m3)

C	2.740042000	-1.379713000	0.053737000
C	4.146612000	-1.184950000	-0.154906000
C	4.319080000	0.131757000	-0.463489000
C	3.017529000	0.739482000	-0.431770000
N	2.064956000	-0.197488000	-0.125212000
C	2.160434000	-2.597533000	0.374324000
C	2.780212000	2.087724000	-0.652746000
C	1.535549000	2.695237000	-0.563923000
C	1.308298000	4.100799000	-0.744328000
C	-0.025172000	4.313166000	-0.548449000
C	-0.609681000	3.034755000	-0.260025000
N	0.356282000	2.057528000	-0.275277000
C	-1.961222000	2.829732000	-0.026916000
C	-2.550931000	1.594408000	0.202019000
C	-3.959379000	1.396430000	0.385231000
C	-4.151534000	0.055241000	0.546687000
C	-2.857808000	-0.560668000	0.471935000
N	-1.892086000	0.390631000	0.258217000
C	-2.628501000	-1.919442000	0.629364000
C	-1.374202000	-2.511514000	0.626671000
C	-1.138618000	-3.905734000	0.859370000
C	0.212999000	-4.091905000	0.811095000
C	0.798854000	-2.811906000	0.538275000
N	-0.182165000	-1.855536000	0.432889000
H	4.893964000	-1.972712000	-0.074381000
H	5.239707000	0.667082000	-0.691127000
H	2.822686000	-3.457522000	0.485469000
H	3.636505000	2.720568000	-0.890792000
H	2.087127000	4.821739000	-0.988085000
H	-0.584317000	5.246239000	-0.598016000
Fe	0.087018000	0.100181000	0.112501000
H	-2.614879000	3.703122000	-0.046458000
H	-4.696929000	2.197539000	0.380452000
H	-5.081205000	-0.488313000	0.708169000
H	-3.492677000	-2.563845000	0.797951000
H	-1.923277000	-4.639363000	1.036812000
H	0.781062000	-5.012213000	0.938280000
O	-0.125032000	-0.199381000	-1.821132000
S	0.393559000	0.526763000	2.294228000
C	-0.783531000	-0.465595000	3.215367000
H	-1.823681000	-0.218570000	2.955430000
H	-0.614241000	-1.542251000	3.069557000
H	-0.635100000	-0.237249000	4.280716000
N	-1.046493000	-0.990041000	-2.287140000
O	-1.078753000	-1.087483000	-3.487363000

ScPorph (m2)

C	2.688388000	-1.463119000	0.031484000
C	4.109785000	-1.219368000	0.068159000
C	4.293820000	0.122661000	-0.085929000
C	2.987366000	0.721312000	-0.221644000
N	2.020726000	-0.267286000	-0.162623000
C	2.087646000	-2.699386000	0.205032000
C	2.746478000	2.078646000	-0.354849000
C	1.483286000	2.714948000	-0.395929000
C	1.265464000	4.117918000	-0.425648000
C	-0.106988000	4.312873000	-0.404585000
C	-0.705736000	3.024675000	-0.363215000
N	0.272666000	2.052316000	-0.364880000
C	-2.092965000	2.761661000	-0.282089000
C	-2.691214000	1.520007000	-0.141226000
C	-4.105901000	1.302473000	0.041328000
C	-4.292066000	-0.038629000	0.196867000
C	-2.993590000	-0.663179000	0.114601000
N	-2.029947000	0.304165000	-0.113226000
C	-2.754193000	-2.016957000	0.279999000
C	-1.490819000	-2.652758000	0.310325000
C	-1.264588000	-4.033861000	0.549477000
C	0.108084000	-4.226680000	0.527412000
C	0.698970000	-2.959655000	0.275515000
N	-0.285566000	-2.000288000	0.142740000
H	4.865336000	-1.991105000	0.214397000
H	5.230841000	0.679562000	-0.092025000
H	2.752725000	-3.555266000	0.342805000
H	3.621401000	2.732837000	-0.380243000
H	2.052394000	4.872106000	-0.442591000
H	-0.654131000	5.255680000	-0.401081000
Sc	-0.025055000	-0.068945000	-0.847956000
H	-2.755960000	3.630259000	-0.280720000
H	-4.853941000	2.094919000	0.065912000
H	-5.224174000	-0.574835000	0.375381000
H	-3.625643000	-2.654562000	0.447780000
H	-2.045866000	-4.773759000	0.722918000
H	0.660672000	-5.153679000	0.681201000

ScPorph-OH (m1)

C	2.676144000	-1.436601000	0.052592000
C	4.097717000	-1.203721000	0.082171000
C	4.282770000	0.143456000	-0.063269000
C	2.973651000	0.735784000	-0.182684000
N	2.019958000	-0.245813000	-0.114842000
C	2.052933000	-2.681728000	0.212072000
C	2.712910000	2.106833000	-0.308530000

C	1.461268000	2.736748000	-0.340386000
C	1.242065000	4.160839000	-0.354557000
C	-0.111246000	4.354990000	-0.313497000
C	-0.720622000	3.048722000	-0.274691000
N	0.255033000	2.088197000	-0.306149000
C	-2.093915000	2.793427000	-0.165425000
C	-2.715830000	1.546073000	-0.018837000
C	-4.128380000	1.335900000	0.172455000
C	-4.314765000	-0.011676000	0.310991000
C	-3.015665000	-0.627128000	0.206739000
N	-2.065690000	0.339218000	-0.000008000
C	-2.756075000	-1.997757000	0.337161000
C	-1.505309000	-2.630199000	0.332620000
C	-1.275598000	-4.032724000	0.570216000
C	0.078067000	-4.224968000	0.536466000
C	0.677235000	-2.939801000	0.276850000
N	-0.305946000	-1.994413000	0.147438000
H	4.853930000	-1.977013000	0.212608000
H	5.220527000	0.698102000	-0.075951000
H	2.714936000	-3.541775000	0.338091000
H	3.585351000	2.764021000	-0.339391000
H	2.029869000	4.913345000	-0.373726000
H	-0.656963000	5.297816000	-0.292152000
Sc	-0.053396000	-0.029800000	-0.776724000
H	-2.750675000	3.666603000	-0.151381000
H	-4.877098000	2.126620000	0.208904000
H	-5.246727000	-0.549062000	0.483904000
H	-3.623541000	-2.641297000	0.502765000
H	-2.056015000	-4.770321000	0.753851000
H	0.630903000	-5.151529000	0.688550000
O	-0.046947000	-0.266212000	-2.626986000
H	-0.076725000	-0.347100000	-3.582049000

ScPorph-Cl (m1)

C	2.691479000	-1.442211000	0.090270000
C	4.110675000	-1.208558000	0.122668000
C	4.294338000	0.137539000	-0.029485000
C	2.986614000	0.726082000	-0.156606000
N	2.028197000	-0.253459000	-0.079337000
C	2.074056000	-2.687564000	0.241544000
C	2.730025000	2.092254000	-0.307077000
C	1.476917000	2.710250000	-0.364014000
C	1.253690000	4.130858000	-0.400225000
C	-0.100089000	4.321478000	-0.376435000
C	-0.704662000	3.016188000	-0.327693000
N	0.271967000	2.052783000	-0.335478000
C	-2.075173000	2.761611000	-0.226116000
C	-2.685555000	1.513973000	-0.066601000
C	-4.096397000	1.306359000	0.123634000
C	-4.283028000	-0.038483000	0.276998000
C	-2.985823000	-0.653835000	0.182296000
N	-2.028821000	0.307415000	-0.032688000
C	-2.733876000	-2.020620000	0.325516000
C	-1.481619000	-2.641440000	0.331519000
C	-1.247937000	-4.039664000	0.574163000
C	0.105943000	-4.225785000	0.548395000
C	0.700238000	-2.940458000	0.291498000
N	-0.284851000	-1.993338000	0.152165000
H	4.865777000	-1.982185000	0.255943000
H	5.230263000	0.694817000	-0.046372000
H	2.734515000	-3.547867000	0.370757000
H	3.600280000	2.751608000	-0.340859000
H	2.040995000	4.883595000	-0.419967000
H	-0.650692000	5.261434000	-0.372583000
Sc	-0.032493000	-0.056522000	-0.828620000
H	-2.736189000	3.631201000	-0.225446000
H	-4.842540000	2.099620000	0.150008000
H	-5.213391000	-0.576461000	0.455120000
H	-3.600779000	-2.663842000	0.492368000
H	-2.027999000	-4.778059000	0.754658000
H	0.664374000	-5.147953000	0.705219000
Cl	-0.584845000	-0.519247000	-3.114628000

ScPorph-F (m1)

C	2.686630000	-1.455213000	0.055887000
C	4.107575000	-1.222615000	0.080221000
C	4.291350000	0.122761000	-0.078588000
C	2.981771000	0.710700000	-0.202355000
N	2.025468000	-0.267860000	-0.121294000
C	2.068585000	-2.698655000	0.230474000
C	2.720879000	2.078173000	-0.343590000
C	1.465581000	2.695136000	-0.385318000
C	1.240303000	4.117034000	-0.409739000
C	-0.113487000	4.304866000	-0.379942000
C	-0.715663000	2.996589000	-0.339209000
N	0.263007000	2.036748000	-0.355423000
C	-2.087814000	2.741934000	-0.240829000
C	-2.703372000	1.494705000	-0.086090000
C	-4.113665000	1.286807000	0.115933000
C	-4.297232000	-0.058141000	0.275770000
C	-2.998606000	-0.672262000	0.174014000
N	-2.048027000	0.289727000	-0.056703000
C	-2.740393000	-2.037454000	0.334698000
C	-1.485697000	-2.656993000	0.350012000
C	-1.250155000	-4.054750000	0.603920000
C	0.103945000	-4.239172000	0.573541000
C	0.694327000	-2.953090000	0.302378000
N	-0.291724000	-2.009731000	0.166306000
H	4.863370000	-1.995256000	0.216382000
H	5.227713000	0.679438000	-0.099138000
H	2.731179000	-3.557328000	0.361659000
H	3.589542000	2.739813000	-0.378627000
H	2.025982000	4.871712000	-0.426490000
H	-0.665626000	5.243997000	-0.366877000
Sc	-0.047222000	-0.069987000	-0.865544000
H	-2.746509000	3.613521000	-0.234761000
H	-4.861229000	2.078681000	0.148150000
H	-5.225713000	-0.595775000	0.465359000
H	-3.605863000	-2.680779000	0.509948000
H	-2.027987000	-4.793822000	0.792196000
H	0.663878000	-5.160057000	0.733794000
F	-0.261516000	0.058110000	-2.725618000

ScPorph-N3 (m1)

C	2.668938000	-1.403827000	0.047323000
C	4.089744000	-1.184349000	0.100962000

C	4.286308000	0.164595000	-0.002967000
C	2.985081000	0.769194000	-0.122108000
N	2.017651000	-0.204644000	-0.090803000
C	2.038036000	-2.647195000	0.156277000
C	2.744484000	2.143178000	-0.221668000
C	1.498767000	2.778168000	-0.257857000
C	1.290361000	4.202396000	-0.251972000
C	-0.061470000	4.405532000	-0.226868000
C	-0.678268000	3.104291000	-0.216369000
N	0.288430000	2.132906000	-0.249454000
C	-2.051695000	2.858492000	-0.128294000
C	-2.674019000	1.612019000	-0.005091000
C	-4.090103000	1.412979000	0.157343000
C	-4.291223000	0.066427000	0.272167000
C	-2.997711000	-0.558002000	0.181793000
N	-2.028667000	0.399320000	0.010090000
C	-2.760922000	-1.931258000	0.286326000
C	-1.515109000	-2.564865000	0.278575000
C	-1.296638000	-3.975199000	0.459494000
C	0.054520000	-4.175359000	0.415058000
C	0.661761000	-2.886295000	0.208545000
N	-0.312774000	-1.922829000	0.121380000
H	4.836988000	-1.968261000	0.217782000
H	5.226849000	0.714225000	0.011156000
H	2.689568000	-3.518659000	0.252018000
H	3.622867000	2.792589000	-0.229127000
H	2.084879000	4.947829000	-0.248334000
H	-0.603003000	5.350408000	-0.198141000
Sc	-0.040097000	0.034320000	-0.818134000
H	-2.706373000	3.732839000	-0.112978000
H	-4.829917000	2.211952000	0.190058000
H	-5.229234000	-0.467669000	0.419430000
H	-3.635773000	-2.570963000	0.422865000
H	-2.083962000	-4.712002000	0.612986000
H	0.603620000	-5.109625000	0.526809000
N	-0.754390000	-0.706159000	-2.695888000
N	-1.134027000	-1.780753000	-3.061210000
N	-1.509304000	-2.809712000	-3.442298000

ScPorph-SCN (m1)

C	2.621616000	-1.479206000	0.038200000
C	4.039048000	-1.241382000	0.013014000
C	4.211867000	0.104578000	-0.148627000
C	2.899703000	0.687467000	-0.227098000
N	1.944186000	-0.294408000	-0.120035000
C	2.021731000	-2.723216000	0.244636000
C	2.635285000	2.050066000	-0.367700000
C	1.376369000	2.650787000	-0.391460000
C	1.140500000	4.066623000	-0.465643000
C	-0.213213000	4.245649000	-0.421890000
C	-0.803280000	2.937467000	-0.322233000
N	0.180179000	1.978327000	-0.319041000
C	-2.171883000	2.687145000	-0.209304000
C	-2.775460000	1.441867000	-0.025152000
C	-4.176647000	1.232975000	0.220745000
C	-4.348093000	-0.107784000	0.419668000
C	-3.051545000	-0.717296000	0.293685000
N	-2.109228000	0.240546000	0.007562000
C	-2.782063000	-2.072341000	0.490451000
C	-1.523585000	-2.677182000	0.484217000
C	-1.275515000	-4.063001000	0.774618000
C	0.076224000	-4.248529000	0.699602000
C	0.653835000	-2.973575000	0.368951000
N	-0.336669000	-2.029650000	0.237024000
H	4.799650000	-2.013131000	0.123542000
H	5.143575000	0.666566000	-0.199859000
H	2.691651000	-3.577045000	0.366691000
H	3.499068000	2.715119000	-0.433998000
H	1.921902000	4.823034000	-0.527203000
H	-0.774942000	5.178697000	-0.440596000
Sc	-0.116488000	-0.129704000	-0.828751000
H	-2.832155000	3.557066000	-0.215047000
H	-4.924610000	2.024040000	0.257262000
H	-5.265788000	-0.646309000	0.652927000
H	-3.635242000	-2.715462000	0.717073000
H	-2.046699000	-4.792982000	1.017219000
H	0.644910000	-5.161880000	0.870149000
S	1.003676000	0.507715000	-3.247788000
C	-0.249919000	1.560413000	-3.513414000
N	-1.140144000	2.313279000	-3.695565000

ScPorph-ONO2 (m1)

C	2.601221000	-1.516566000	0.049865000
C	4.021276000	-1.289932000	0.009624000
C	4.204357000	0.052641000	-0.171770000
C	2.895795000	0.647698000	-0.244333000
N	1.937297000	-0.326624000	-0.113869000
C	1.986569000	-2.754497000	0.263883000
C	2.637672000	2.013669000	-0.393411000
C	1.385398000	2.636738000	-0.397871000
C	1.162637000	4.056049000	-0.472982000
C	-0.189266000	4.250035000	-0.409900000
C	-0.793862000	2.948103000	-0.297330000
N	0.182344000	1.984028000	-0.297461000
C	-2.164405000	2.700839000	-0.168941000
C	-2.780639000	1.459444000	0.020812000
C	-4.188459000	1.256495000	0.236920000
C	-4.372752000	-0.084927000	0.425605000
C	-3.077431000	-0.703604000	0.326095000
N	-2.126784000	0.252832000	0.068728000
C	-2.815633000	-2.064929000	0.506777000
C	-1.562554000	-2.686112000	0.503146000
C	-1.326935000	-4.081035000	0.764751000
C	0.024103000	-4.276175000	0.693790000
C	0.615588000	-2.999188000	0.390202000
N	-0.367881000	-2.048781000	0.275797000
H	4.777783000	-2.065534000	0.121713000
H	5.141554000	0.603808000	-0.239404000
H	2.649667000	-3.614623000	0.380238000
H	3.508017000	2.668069000	-0.479611000
H	1.949265000	4.805644000	-0.550092000
H	-0.738485000	5.190697000	-0.424882000
Sc	-0.140131000	-0.129214000	-0.726210000
H	-2.821030000	3.573704000	-0.180020000
H	-4.934800000	2.049835000	0.255352000
H	-5.300790000	-0.617232000	0.630785000

H	-3.677295000	-2.705006000	0.709376000
H	-2.104378000	-4.811564000	0.984655000
H	0.582078000	-5.199455000	0.845549000
O	0.583105000	0.276702000	-2.801423000
N	-0.236986000	-0.538133000	-3.343520000
O	-1.011268000	-1.132440000	-2.520697000
O	-0.275078000	-0.730950000	-4.529371000

ScPorph-Me (m1)

C	2.709301000	-1.461074000	0.092151000
C	4.130473000	-1.227768000	0.128180000
C	4.315495000	0.115279000	-0.049162000
C	3.006332000	0.700742000	-0.193786000
N	2.050542000	-0.276958000	-0.109213000
C	2.087829000	-2.702212000	0.275322000
C	2.743575000	2.066988000	-0.349635000
C	1.488255000	2.684914000	-0.394257000
C	1.265349000	4.107506000	-0.424616000
C	-0.088055000	4.297792000	-0.388477000
C	-0.692198000	2.990349000	-0.340497000
N	0.284521000	2.028950000	-0.357294000
C	-2.064972000	2.736776000	-0.243594000
C	-2.682312000	1.488796000	-0.099779000
C	-4.095834000	1.279172000	0.079892000
C	-4.281622000	-0.067283000	0.223738000
C	-2.980495000	-0.679971000	0.137768000
N	-2.027181000	0.283921000	-0.070945000
C	-2.723711000	-2.046221000	0.295808000
C	-1.468842000	-2.665800000	0.334460000
C	-1.235601000	-4.062523000	0.598689000
C	0.119347000	-4.243932000	0.596610000
C	0.712481000	-2.957812000	0.329597000
N	-0.272992000	-2.017526000	0.171003000
H	4.885440000	-1.997675000	0.283463000
H	5.252166000	0.671621000	-0.068055000
H	2.748488000	-3.559397000	0.425400000
H	3.611943000	2.728984000	-0.388367000
H	2.051935000	4.861110000	-0.448611000
H	-0.638285000	5.238117000	-0.377317000
Sc	-0.019448000	-0.083090000	-0.870808000
H	-2.723148000	3.608831000	-0.237709000
H	-4.844353000	2.070404000	0.106775000
H	-5.213094000	-0.606483000	0.393503000
H	-3.591886000	-2.690597000	0.453482000
H	-2.015096000	-4.802854000	0.775010000
H	0.677663000	-5.163030000	0.772343000
C	-0.466581000	0.144541000	-3.059502000
H	0.457753000	0.272721000	-3.656614000
H	-0.985870000	-0.744624000	-3.468606000
H	-1.110868000	1.016038000	-3.285189000

ScPorph-ONO (m1)

C	2.633019000	-1.579237000	0.141068000
C	4.050906000	-1.334398000	0.134374000
C	4.220970000	-0.000064000	-0.108224000
C	2.906334000	0.569021000	-0.249204000
N	1.956109000	-0.410561000	-0.101352000
C	2.030420000	-2.813005000	0.403001000
C	2.636054000	1.924492000	-0.455633000
C	1.376893000	2.531518000	-0.494689000
C	1.148020000	3.948871000	-0.600561000
C	-0.204558000	4.136303000	-0.546640000
C	-0.799443000	2.832123000	-0.409688000
N	0.178821000	1.871803000	-0.388889000
C	-2.166380000	2.578540000	-0.271371000
C	-2.763686000	1.337484000	-0.034790000
C	-4.171023000	1.129602000	0.181852000
C	-4.343149000	-0.206426000	0.409261000
C	-3.040001000	-0.813714000	0.333261000
N	-2.094546000	0.141423000	0.055801000
C	-2.776524000	-2.169158000	0.547700000
C	-1.520277000	-2.780784000	0.571603000
C	-1.272034000	-4.156609000	0.913606000
C	0.082267000	-4.334976000	0.874894000
C	0.660046000	-3.067654000	0.508513000
N	-0.334058000	-2.139886000	0.320337000
H	4.813750000	-2.091978000	0.309454000
H	5.151747000	0.562581000	-0.171800000
H	2.700543000	-3.656271000	0.584758000
H	3.499846000	2.586914000	-0.547845000
H	1.931663000	4.700748000	-0.686424000
H	-0.760177000	5.072837000	-0.577977000
Sc	-0.122069000	-0.271009000	-0.814938000
H	-2.833137000	3.443055000	-0.307159000
H	-4.923588000	1.917220000	0.172444000
H	-5.266072000	-0.743146000	0.626082000
H	-3.636579000	-2.805395000	0.768790000
H	-2.043022000	-4.883322000	1.166894000
H	0.652215000	-5.237995000	1.090890000
N	-1.230535000	0.107683000	-2.718167000
O	-1.181331000	-1.162808000	-2.519848000
O	-1.833255000	0.547691000	-3.663993000

TiPorph-Cl (m2)

C	2.686693000	-1.470550000	-0.008501000
C	4.105801000	-1.244527000	-0.024205000
C	4.292022000	0.103875000	-0.151689000
C	2.985745000	0.699920000	-0.220594000
N	2.021316000	-0.275189000	-0.142456000
C	2.076512000	-2.714799000	0.161012000
C	2.727996000	2.065957000	-0.329895000
C	1.470501000	2.672427000	-0.338074000
C	1.247567000	4.090432000	-0.391533000
C	-0.104985000	4.281662000	-0.344242000
C	-0.707583000	2.979326000	-0.263398000
N	0.267312000	2.009487000	-0.265128000
C	-2.079542000	2.740866000	-0.173264000
C	-2.690662000	1.494103000	-0.039917000
C	-4.103821000	1.273806000	0.096354000
C	-4.284086000	-0.075245000	0.221807000
C	-2.979641000	-0.677892000	0.169607000
N	-2.022016000	0.294147000	-0.001872000
C	-2.726817000	-2.043100000	0.312291000
C	-1.473978000	-2.658535000	0.329769000
C	-1.242326000	-4.062290000	0.524650000
C	0.111669000	-4.251261000	0.480143000

C	0.706052000	-2.962049000	0.258128000
N	-0.275905000	-2.004162000	0.169093000
H	4.859819000	-2.025500000	0.065254000
H	5.229383000	0.657608000	-0.188867000
H	2.739897000	-3.577374000	0.254582000
H	3.593530000	2.729602000	-0.386447000
H	2.035410000	4.840725000	-0.446274000
H	-0.657659000	5.220343000	-0.352126000
Ti	-0.027894000	-0.065929000	-0.622034000
H	-2.734844000	3.614375000	-0.186930000
H	-4.857512000	2.060316000	0.100731000
H	-5.216077000	-0.624328000	0.350883000
H	-3.595003000	-2.691800000	0.448195000
H	-2.023663000	-4.806153000	0.674543000
H	0.668442000	-5.181463000	0.587107000
Cl	-0.128694000	-0.415223000	-2.865696000

TiPorph-Cl-OH (m1)

C	2.735230000	-1.468103000	0.124253000
C	4.146139000	-1.243823000	-0.018556000
C	4.330505000	0.103234000	-0.176184000
C	3.033792000	0.719491000	-0.132521000
N	2.082988000	-0.256313000	0.064591000
C	2.120367000	-2.713987000	0.278537000
C	2.775290000	2.084559000	-0.284718000
C	1.524366000	2.713411000	-0.275788000
C	1.281836000	4.120428000	-0.449990000
C	-0.071453000	4.306592000	-0.377520000
C	-0.668024000	3.014848000	-0.158104000
N	0.328163000	2.071702000	-0.099273000
C	-2.039167000	2.762772000	-0.040270000
C	-2.668676000	1.524921000	0.148354000
C	-4.090182000	1.297029000	0.212907000
C	-4.272965000	-0.050002000	0.361653000
C	-2.963437000	-0.653113000	0.389969000
N	-2.021078000	0.328917000	0.269330000
C	-2.690994000	-2.023055000	0.498704000
C	-1.440161000	-2.651654000	0.489309000
C	-1.214963000	-4.070740000	0.579229000
C	0.136822000	-4.269707000	0.517368000
C	0.748575000	-2.972406000	0.388532000
N	-0.229447000	-2.017233000	0.378747000
H	4.900693000	-2.029034000	-0.002690000
H	5.265939000	0.643197000	-0.316181000
H	2.783703000	-3.581042000	0.300199000
H	3.643677000	2.728669000	-0.437365000
H	2.057541000	4.868480000	-0.606349000
H	-0.629409000	5.237822000	-0.465800000
Ti	0.060740000	0.043254000	0.278531000
H	-2.696203000	3.631781000	-0.117390000
H	-4.847641000	2.076353000	0.141952000
H	-5.210150000	-0.599575000	0.438868000
H	-3.557627000	-2.681981000	0.587201000
H	-2.001928000	-4.817561000	0.674283000
H	0.683271000	-5.211122000	0.552973000
Cl	-0.275839000	-0.231551000	-2.108096000
O	-0.001529000	0.231893000	2.059908000
H	-0.698273000	0.318545000	2.724115000

TiPorph-Cl-Cl (m1)

C	2.699088000	-1.471967000	0.034089000
C	4.114041000	-1.241953000	-0.042525000
C	4.296933000	0.106649000	-0.190001000
C	2.994282000	0.711605000	-0.209877000
N	2.042241000	-0.270024000	-0.074113000
C	2.081215000	-2.714907000	0.198275000
C	2.731823000	2.077840000	-0.340996000
C	1.479225000	2.697966000	-0.361612000
C	1.245148000	4.110571000	-0.461927000
C	-0.110228000	4.299541000	-0.422053000
C	-0.715762000	3.003280000	-0.296358000
N	0.271906000	2.047982000	-0.270745000
C	-2.086644000	2.750408000	-0.197354000
C	-2.704588000	1.507479000	-0.033178000
C	-4.119584000	1.277508000	0.043423000
C	-4.302475000	-0.071083000	0.190905000
C	-2.999771000	-0.676008000	0.210790000
N	-2.047798000	0.305589000	0.075041000
C	-2.737249000	-2.042255000	0.341911000
C	-1.484659000	-2.662373000	0.362538000
C	-1.250629000	-4.074983000	0.462819000
C	0.104742000	-4.264018000	0.422975000
C	0.710328000	-2.967789000	0.297293000
N	-0.277272000	-2.012441000	0.271707000
H	4.871272000	-2.022274000	0.015932000
H	5.233688000	0.654849000	-0.278397000
H	2.741289000	-3.583049000	0.248094000
H	3.599921000	2.734323000	-0.426892000
H	2.027980000	4.862768000	-0.545656000
H	-0.662503000	5.237015000	-0.465854000
Ti	-0.002634000	0.017732000	0.000517000
H	-2.746681000	3.618579000	-0.247184000
H	-4.876803000	2.057840000	-0.015047000
H	-5.239221000	-0.619299000	0.279299000
H	-3.605321000	-2.698776000	0.427797000
H	-2.033493000	-4.827151000	0.546519000
H	0.656971000	-5.201519000	0.466771000
Cl	-0.160979000	-0.316900000	-2.302452000
Cl	0.154116000	0.352748000	2.303486000

TiPorph-Cl-F (m1)

C	2.705846000	-1.473114000	0.048030000
C	4.120622000	-1.245489000	-0.048236000
C	4.304444000	0.102714000	-0.195125000
C	3.002581000	0.711022000	-0.192829000
N	2.051452000	-0.268011000	-0.041007000
C	2.087542000	-2.717839000	0.200658000
C	2.739700000	2.077293000	-0.327992000
C	1.487631000	2.699667000	-0.333845000
C	1.252415000	4.109775000	-0.468004000
C	-0.102192000	4.300064000	-0.420105000
C	-0.707131000	3.007434000	-0.255669000
N	0.281595000	2.053845000	-0.206676000
C	-2.078701000	2.754759000	-0.161998000
C	-2.698549000	1.512026000	0.001978000
C	-4.114824000	1.278775000	0.054605000

C	-4.297006000	-0.070916000	0.189852000
C	-2.992823000	-0.673961000	0.225157000
N	-2.042459000	0.312068000	0.117567000
C	-2.730122000	-2.042629000	0.336428000
C	-1.478589000	-2.666609000	0.356704000
C	-1.246547000	-4.081720000	0.438403000
C	0.108563000	-4.271764000	0.403208000
C	0.716342000	-2.973591000	0.298085000
N	-0.269956000	-2.019082000	0.283375000
H	4.876829000	-2.027867000	-0.004507000
H	5.241112000	0.648711000	-0.297488000
H	2.748077000	-3.586449000	0.236098000
H	3.607052000	2.731211000	-0.438337000
H	2.034231000	4.859124000	-0.581299000
H	-0.654726000	5.236131000	-0.486203000
Ti	0.003953000	0.021549000	0.075267000
H	-2.738786000	3.621806000	-0.228862000
H	-4.872824000	2.057736000	-0.011878000
H	-5.233853000	-0.621879000	0.258313000
H	-3.599673000	-2.699605000	0.403014000
H	-2.030583000	-4.834116000	0.509112000
H	0.659508000	-5.210439000	0.438303000
Cl	-0.173670000	-0.293992000	-2.266033000
F	0.104164000	0.230197000	1.864042000

TiPorph-Cl-N3 (m1)

C	2.656743000	-1.418784000	0.184984000
C	4.075417000	-1.190669000	0.129558000
C	4.260784000	0.156637000	-0.020923000
C	2.955498000	0.760347000	-0.063606000
N	2.005973000	-0.218037000	0.062470000
C	2.031356000	-2.659616000	0.352263000
C	2.687522000	2.125417000	-0.223836000
C	1.436086000	2.742064000	-0.294386000
C	1.205317000	4.146810000	-0.470580000
C	-0.150532000	4.337954000	-0.494206000
C	-0.765038000	3.052077000	-0.328298000
N	0.218973000	2.097149000	-0.212928000
C	-2.138411000	2.799997000	-0.271962000
C	-2.754710000	1.561242000	-0.075548000
C	-4.167356000	1.326509000	0.005522000
C	-4.345218000	-0.014557000	0.217651000
C	-3.043279000	-0.616347000	0.266071000
N	-2.091286000	0.365776000	0.084887000
C	-2.784095000	-1.974994000	0.456403000
C	-1.533231000	-2.603938000	0.494549000
C	-1.301666000	-4.012404000	0.666513000
C	0.052662000	-4.204134000	0.628210000
C	0.657898000	-2.913420000	0.435757000
N	-0.330415000	-1.964487000	0.357946000
H	4.831213000	-1.971110000	0.202907000
H	5.198599000	0.704679000	-0.098676000
H	2.690365000	-3.526281000	0.435077000
H	3.554310000	2.783927000	-0.309241000
H	1.991405000	4.894346000	-0.564800000
H	-0.696673000	5.272945000	-0.609819000
Ti	-0.060058000	0.082953000	0.030950000
H	-2.798998000	3.662714000	-0.376549000
H	-4.928250000	2.099681000	-0.088370000
H	-5.280901000	-0.559884000	0.332004000
H	-3.656071000	-2.620503000	0.579695000
H	-2.084480000	-4.757776000	0.797345000
H	0.604812000	-5.137968000	0.723012000
Cl	-0.013266000	-0.342083000	-2.258111000
N	0.091975000	0.245455000	2.019293000
N	0.692002000	-0.313019000	2.892331000
N	1.261082000	-0.837158000	3.750340000

TiPorph-Cl-SCN (m1)

C	2.749153000	-1.367956000	0.115635000
C	4.157561000	-1.164172000	-0.052634000
C	4.333992000	0.131148000	-0.458423000
C	3.035680000	0.736802000	-0.514865000
N	2.081607000	-0.199036000	-0.183367000
C	2.142352000	-2.570087000	0.478354000
C	2.777724000	2.085265000	-0.761414000
C	1.540664000	2.721937000	-0.646199000
C	1.313601000	4.132324000	-0.750041000
C	-0.006779000	4.351732000	-0.458569000
C	-0.602112000	3.074917000	-0.198570000
N	0.358272000	2.092558000	-0.323979000
C	-1.952318000	2.852610000	0.073863000
C	-2.581152000	1.616358000	0.223467000
C	-3.989910000	1.413383000	0.389795000
C	-4.203632000	0.061445000	0.418993000
C	-2.923377000	-0.571582000	0.298106000
N	-1.949536000	0.393220000	0.166804000
C	-2.679724000	-1.941639000	0.393720000
C	-1.426461000	-2.551964000	0.447671000
C	-1.182690000	-3.932785000	0.737202000
C	0.174337000	-4.094711000	0.826530000
C	0.772417000	-2.820557000	0.561850000
N	-0.221226000	-1.891638000	0.332969000
H	4.915012000	-1.928314000	0.112852000
H	5.265474000	0.647997000	-0.683741000
H	2.806434000	-3.413306000	0.676413000
H	3.636215000	2.716282000	-0.997846000
H	2.079673000	4.863786000	-1.001781000
H	-0.544007000	5.298329000	-0.430958000
Ti	0.037195000	0.058877000	-0.239337000
H	-2.591597000	3.735666000	0.127751000
H	-4.723811000	2.214108000	0.463925000
H	-5.146711000	-0.470827000	0.531349000
H	-3.548933000	-2.590040000	0.517257000
H	-1.961432000	-4.681404000	0.871993000
H	0.732820000	-5.004384000	1.040982000
Cl	-0.242914000	-0.275025000	-2.458057000
S	0.381727000	0.540100000	2.378533000
C	-0.672096000	-0.534933000	3.063883000
N	-1.416823000	-1.297212000	3.569567000

TiPorph-Cl-ONO2 (m1)

C	2.634120000	-1.523939000	0.144119000
C	4.054637000	-1.340636000	0.071229000
C	4.279807000	-0.016869000	-0.198318000
C	2.998704000	0.623883000	-0.273625000

N	2.012610000	-0.313855000	-0.075896000
C	1.982508000	-2.741410000	0.355488000
C	2.781178000	1.993221000	-0.445224000
C	1.552573000	2.656034000	-0.391533000
C	1.367871000	4.078616000	-0.409356000
C	0.032746000	4.315124000	-0.219306000
C	-0.612462000	3.038953000	-0.105005000
N	0.332073000	2.043621000	-0.224294000
C	-1.983121000	2.835210000	0.067225000
C	-2.647158000	1.606760000	0.121522000
C	-4.068014000	1.419197000	0.171350000
C	-4.299431000	0.069738000	0.120974000
C	-3.021780000	-0.579579000	0.069033000
N	-2.033477000	0.378393000	0.065190000
C	-2.808019000	-1.959513000	0.085037000
C	-1.577772000	-2.616665000	0.167576000
C	-1.386979000	-4.031853000	0.309538000
C	-0.040829000	-4.247493000	0.439674000
C	0.603085000	-2.967007000	0.353409000
N	-0.352884000	-1.995068000	0.192562000
H	4.787648000	-2.134446000	0.205278000
H	5.233716000	0.493169000	-0.323779000
H	2.620415000	-3.616681000	0.492413000
H	3.668206000	2.617180000	-0.570084000
H	2.169562000	4.805035000	-0.532172000
H	-0.481222000	5.273317000	-0.160525000
Ti	-0.024457000	0.011060000	-0.128464000
H	-2.606078000	3.729848000	0.123958000
H	-4.797122000	2.226272000	0.222585000
H	-5.255983000	-0.450503000	0.130632000
H	-3.698948000	-2.590553000	0.088153000
H	-2.190713000	-4.766506000	0.319111000
H	0.481065000	-5.194281000	0.569835000
Cl	-0.222132000	-0.293978000	-2.394011000
O	0.140563000	0.234560000	1.867933000
N	-0.632416000	-0.053859000	2.896303000
O	-1.552131000	-0.838409000	2.739625000
O	-0.345918000	0.492193000	3.946804000

TiPorph-Cl-Me (m1)

C	2.722964000	-1.449631000	0.045431000
C	4.137923000	-1.224871000	-0.027840000
C	4.332014000	0.123203000	-0.170985000
C	3.036253000	0.739044000	-0.188954000
N	2.072344000	-0.233834000	-0.060630000
C	2.113421000	-2.695896000	0.208477000
C	2.781009000	2.107915000	-0.314933000
C	1.528273000	2.727317000	-0.326621000
C	1.290644000	4.135568000	-0.452708000
C	-0.064863000	4.324804000	-0.408546000
C	-0.674113000	3.034956000	-0.253198000
N	0.320596000	2.073804000	-0.211722000
C	-2.046113000	2.798133000	-0.153937000
C	-2.681474000	1.558005000	0.011652000
C	-4.099288000	1.324397000	0.105022000
C	-4.271199000	-0.024549000	0.258688000
C	-2.959750000	-0.621445000	0.260925000
N	-2.024182000	0.369704000	0.109949000
C	-2.687895000	-1.989583000	0.393058000
C	-1.442724000	-2.634381000	0.390073000
C	-1.225774000	-4.051829000	0.534982000
C	0.125816000	-4.257600000	0.482388000
C	0.739511000	-2.964972000	0.303553000
N	-0.231440000	-2.013679000	0.249819000
H	4.890740000	-2.009848000	0.028348000
H	5.274230000	0.662588000	-0.256278000
H	2.785813000	-3.554063000	0.271558000
H	3.649911000	2.761887000	-0.409900000
H	2.071165000	4.887207000	-0.560087000
H	-0.615583000	5.262156000	-0.473220000
Ti	0.039056000	0.053203000	-0.046786000
H	-2.692746000	3.676213000	-0.209226000
H	-4.862582000	2.099611000	0.054978000
H	-5.203867000	-0.577723000	0.360449000
H	-3.559355000	-2.638054000	0.510078000
H	-2.014993000	-4.791473000	0.661273000
H	0.668215000	-5.198985000	0.558187000
Cl	-0.350255000	-0.414678000	-2.350165000
C	-0.079519000	0.040333000	2.085204000
H	-0.928946000	-0.528211000	2.491847000
H	0.856042000	-0.384656000	2.487593000
H	-0.166006000	1.091582000	2.413421000

TiPorph-Cl-NO2 (m1)

C	2.630415000	-1.541345000	-0.066952000
C	4.051270000	-1.362269000	-0.036999000
C	4.290226000	-0.014210000	-0.038281000
C	3.017272000	0.640753000	-0.088444000
N	2.017074000	-0.306223000	-0.121730000
C	1.974478000	-2.767857000	0.023074000
C	2.814171000	2.019005000	-0.115659000
C	1.581147000	2.666204000	-0.189343000
C	1.387602000	4.084247000	-0.243702000
C	0.038735000	4.304848000	-0.319894000
C	-0.602454000	3.023704000	-0.293606000
N	0.357937000	2.034520000	-0.237465000
C	-1.980079000	2.818244000	-0.255637000
C	-2.635401000	1.597940000	-0.083500000
C	-4.052389000	1.432454000	0.067679000
C	-4.278900000	0.102423000	0.295128000
C	-3.002273000	-0.552157000	0.269378000
N	-2.018106000	0.376520000	0.027786000
C	-2.792424000	-1.921012000	0.438889000
C	-1.567073000	-2.584990000	0.373133000
C	-1.375067000	-3.999464000	0.509392000
C	-0.035826000	-4.239551000	0.359502000
C	0.598807000	-2.971353000	0.145771000
N	-0.353188000	-1.980449000	0.143258000
H	4.774327000	-2.175593000	-0.001116000
H	5.248200000	0.502388000	-0.007392000
H	2.606414000	-3.657040000	0.059945000
H	3.703105000	2.651432000	-0.088068000
H	2.192070000	4.817788000	-0.225329000
H	-0.488533000	5.256011000	-0.373458000
Ti	-0.007479000	0.006346000	-0.319369000
H	-2.608974000	3.708841000	-0.307033000

H	-4.777924000	2.242532000	0.013940000
H	-5.228166000	-0.404259000	0.462109000
H	-3.675847000	-2.535083000	0.623090000
H	-2.173722000	-4.716668000	0.691185000
H	0.488898000	-5.192832000	0.398902000
Cl	-0.278898000	-0.261220000	-2.574141000
N	0.050879000	0.180819000	1.920979000
O	0.717332000	-0.636530000	2.552492000
O	-0.550821000	1.095324000	2.478569000

TiPorph-Cl-ONO (m1)

C	2.656385000	-1.523285000	0.069236000
C	4.077367000	-1.339742000	-0.005154000
C	4.305822000	-0.000211000	-0.172305000
C	3.026347000	0.648774000	-0.198518000
N	2.038225000	-0.298363000	-0.054507000
C	2.001758000	-2.745996000	0.232342000
C	2.811439000	2.022806000	-0.328722000
C	1.580124000	2.683401000	-0.319909000
C	1.388215000	4.102566000	-0.391596000
C	0.040944000	4.334483000	-0.307698000
C	-0.604904000	3.059084000	-0.191760000
N	0.353607000	2.068604000	-0.207942000
C	-1.981628000	2.852183000	-0.081743000
C	-2.645269000	1.625932000	0.029382000
C	-4.067590000	1.443316000	0.116002000
C	-4.296031000	0.096293000	0.198488000
C	-3.014365000	-0.552706000	0.172949000
N	-2.032613000	0.399510000	0.061007000
C	-2.794655000	-1.929838000	0.273820000
C	-1.562265000	-2.590077000	0.300959000
C	-1.374232000	-4.009724000	0.413826000
C	-0.024809000	-4.239045000	0.415745000
C	0.621202000	-2.961119000	0.299600000
N	-0.334017000	-1.979017000	0.236134000
H	4.808392000	-2.143749000	0.064470000
H	5.260752000	0.514460000	-0.267207000
H	2.637165000	-3.631051000	0.300978000
H	3.700328000	2.649429000	-0.423100000
H	2.191901000	4.831193000	-0.485138000
H	-0.480514000	5.290367000	-0.321235000
Ti	0.005738000	0.046881000	-0.075906000
H	-2.608576000	3.745893000	-0.088759000
H	-4.796822000	2.252009000	0.110713000
H	-5.249937000	-0.422649000	0.279337000
H	-3.683612000	-2.559524000	0.347543000
H	-2.181553000	-4.737195000	0.482598000
H	0.497738000	-5.191897000	0.484835000
Cl	-0.216792000	-0.286924000	-2.356569000
O	0.031434000	0.233131000	1.880389000
N	-0.865426000	-0.221115000	2.754971000
O	-0.573099000	-0.030398000	3.897783000

TiPorph (m3)

C	2.721134000	-1.485865000	0.041347000
C	4.136041000	-1.255985000	-0.049193000
C	4.322140000	0.095882000	-0.189495000
C	3.022168000	0.708703000	-0.188545000
N	2.063704000	-0.274493000	-0.045033000
C	2.091289000	-2.728689000	0.189982000
C	2.750934000	2.076974000	-0.316267000
C	1.496454000	2.700411000	-0.329977000
C	1.254985000	4.111002000	-0.449865000
C	-0.103041000	4.298066000	-0.410227000
C	-0.710087000	3.003594000	-0.267129000
N	0.284616000	2.044068000	-0.223323000
C	-2.083928000	2.744464000	-0.180585000
C	-2.713869000	1.501327000	-0.034231000
C	-4.128846000	1.271500000	0.055108000
C	-4.315018000	-0.080395000	0.195402000
C	-3.015044000	-0.693148000	0.196075000
N	-2.056499000	0.290116000	0.052932000
C	-2.743447000	-2.061086000	0.325960000
C	-1.488899000	-2.684473000	0.341191000
C	-1.247455000	-4.094988000	0.460911000
C	0.110634000	-4.282015000	0.421522000
C	0.717537000	-2.987575000	0.278125000
N	-0.277191000	-2.028031000	0.234325000
H	4.895610000	-2.036536000	-0.009471000
H	5.263031000	0.636982000	-0.288278000
H	2.749418000	-3.599863000	0.238132000
H	3.617632000	2.735366000	-0.414860000
H	2.033423000	4.867135000	-0.549040000
H	-0.653509000	5.236733000	-0.470807000
Ti	0.003790000	0.007820000	0.003664000
H	-2.742213000	3.615538000	-0.228589000
H	-4.888372000	2.052042000	0.014468000
H	-5.255949000	-0.621524000	0.293658000
H	-3.610043000	-2.719625000	0.424732000
H	-2.025766000	-4.851206000	0.560335000
H	0.661031000	-5.220740000	0.482108000

TiPorph-OH (m2)

C	2.713241000	-1.434128000	0.135489000
C	4.125197000	-1.229256000	-0.032938000
C	4.315252000	0.112110000	-0.217173000
C	3.017952000	0.729538000	-0.153786000
N	2.056193000	-0.224231000	0.073053000
C	2.104217000	-2.684070000	0.273060000
C	2.769268000	2.093800000	-0.313247000
C	1.511765000	2.702908000	-0.300852000
C	1.273559000	4.105604000	-0.499715000
C	-0.078594000	4.294236000	-0.415864000
C	-0.668150000	3.007001000	-0.174466000
N	0.320230000	2.047979000	-0.101619000
C	-2.043251000	2.772252000	-0.084597000
C	-2.668554000	1.537820000	0.088318000
C	-4.083838000	1.306763000	0.126507000
C	-4.264339000	-0.037560000	0.313918000
C	-2.956034000	-0.631215000	0.380670000
N	-1.998843000	0.347002000	0.251867000
C	-2.697098000	-1.998944000	0.510261000
C	-1.444669000	-2.622824000	0.495539000
C	-1.223708000	-4.041333000	0.545353000
C	0.129314000	-4.237981000	0.458399000
C	0.735964000	-2.941210000	0.366587000

N	-0.239300000	-1.970118000	0.391951000
H	4.870979000	-2.023584000	-0.030919000
H	5.247664000	0.645717000	-0.398270000
H	2.768626000	-3.551283000	0.279031000
H	3.633122000	2.742203000	-0.473753000
H	2.049316000	4.845908000	-0.691089000
H	-0.640193000	5.221534000	-0.525299000
Ti	0.039170000	0.090690000	0.685785000
H	-2.694235000	3.643903000	-0.183583000
H	-4.843268000	2.081091000	0.024001000
H	-5.200458000	-0.588940000	0.395097000
H	-3.566959000	-2.655384000	0.585400000
H	-2.010074000	-4.790140000	0.632478000
H	0.676622000	-5.180086000	0.461585000
O	0.056133000	0.221191000	2.480986000
H	-0.543520000	-0.144372000	3.143361000

TiPorph-F (m2)

C	2.695320000	-1.475038000	0.041522000
C	4.108231000	-1.255367000	-0.106817000
C	4.288328000	0.093878000	-0.224507000
C	2.984061000	0.697234000	-0.157165000
N	2.027236000	-0.274880000	0.019549000
C	2.086029000	-2.723100000	0.168046000
C	2.734057000	2.062112000	-0.310018000
C	1.482574000	2.680735000	-0.331182000
C	1.248748000	4.080679000	-0.546699000
C	-0.105436000	4.270325000	-0.491157000
C	-0.697113000	2.983886000	-0.245193000
N	0.286473000	2.029336000	-0.150343000
C	-2.068129000	2.734802000	-0.143697000
C	-2.680636000	1.490782000	0.023578000
C	-4.100896000	1.265641000	0.022206000
C	-4.289201000	-0.083729000	0.136161000
C	-2.983821000	-0.681054000	0.216472000
N	-2.018874000	0.294069000	0.156952000
C	-2.722964000	-2.045908000	0.324412000
C	-1.462824000	-2.649384000	0.337233000
C	-1.242778000	-4.068365000	0.385270000
C	0.109238000	-4.261981000	0.333503000
C	0.713042000	-2.959396000	0.257874000
N	-0.258829000	-1.988155000	0.263730000
H	4.860232000	-2.043285000	-0.129047000
H	5.218963000	0.642974000	-0.363788000
H	2.741004000	-3.596928000	0.176393000
H	3.603767000	2.705990000	-0.459421000
H	2.028360000	4.822498000	-0.715263000
H	-0.663314000	5.198869000	-0.607075000
Ti	0.028828000	0.101023000	0.669387000
H	-2.731756000	3.596499000	-0.244790000
H	-4.853519000	2.047791000	-0.069590000
H	-5.227407000	-0.637003000	0.158657000
H	-3.586560000	-2.712820000	0.373884000
H	-2.032431000	-4.817206000	0.436155000
H	0.660512000	-5.201609000	0.332705000
F	0.016770000	0.490044000	2.423303000

TiPorph-N3 (m2)

C	2.636035000	-1.390197000	0.220895000
C	4.052302000	-1.181306000	0.091912000
C	4.241065000	0.157175000	-0.123028000
C	2.942051000	0.769601000	-0.117772000
N	1.980229000	-0.187718000	0.099627000
C	2.013945000	-2.629208000	0.400090000
C	2.674118000	2.125541000	-0.305744000
C	1.414436000	2.730001000	-0.348775000
C	1.189452000	4.131131000	-0.571991000
C	-0.164116000	4.324436000	-0.573598000
C	-0.769038000	3.041298000	-0.344954000
N	0.206617000	2.081738000	-0.211120000
C	-2.142878000	2.803348000	-0.272258000
C	-2.752188000	1.563598000	-0.072138000
C	-4.169500000	1.329554000	-0.054832000
C	-4.347745000	-0.012226000	0.137919000
C	-3.040284000	-0.601135000	0.235607000
N	-2.079946000	0.379775000	0.117658000
C	-2.796232000	-1.968741000	0.381622000
C	-1.548926000	-2.592691000	0.431466000
C	-1.315728000	-4.005095000	0.528222000
C	0.041239000	-4.185499000	0.550812000
C	0.640325000	-2.882218000	0.457483000
N	-0.347308000	-1.925992000	0.392928000
H	4.803897000	-1.967136000	0.155068000
H	5.178927000	0.691197000	-0.271824000
H	2.671639000	-3.499376000	0.457879000
H	3.534571000	2.782625000	-0.451000000
H	1.976977000	4.869659000	-0.716590000
H	-0.714936000	5.252328000	-0.723022000
Ti	-0.030595000	0.130228000	0.596021000
H	-2.802299000	3.665360000	-0.392333000
H	-4.927781000	2.099642000	-0.189706000
H	-5.282380000	-0.569013000	0.195924000
H	-3.672983000	-2.617966000	0.435561000
H	-2.097069000	-4.762788000	0.574687000
H	0.597420000	-5.119676000	0.618298000
N	0.220933000	0.189236000	2.551609000
N	0.847513000	-0.402628000	3.387537000
N	1.433920000	-0.947975000	4.216786000

TiPorph-SCN (m2)

C	2.767924000	-1.413324000	-0.007382000
C	4.171566000	-1.201948000	-0.212962000
C	4.353252000	0.135505000	-0.443164000
C	3.059999000	0.754595000	-0.378121000
N	2.107221000	-0.206995000	-0.110751000
C	2.159167000	-2.644462000	0.254139000
C	2.801459000	2.116297000	-0.556390000
C	1.558415000	2.751685000	-0.499353000
C	1.323789000	4.151826000	-0.714466000
C	-0.023012000	4.358641000	-0.585402000
C	-0.624655000	3.087580000	-0.293330000
N	0.358967000	2.123223000	-0.236671000
C	-1.990563000	2.852463000	-0.116208000
C	-2.608941000	1.620608000	0.116678000
C	-4.018731000	1.395566000	0.252264000
C	-4.199957000	0.052523000	0.451039000

C	-2.900290000	-0.556810000	0.437213000
N	-1.944882000	0.419190000	0.237269000
C	-2.639456000	-1.919889000	0.602443000
C	-1.393251000	-2.551837000	0.586314000
C	-1.166613000	-3.959455000	0.754893000
C	0.183642000	-4.165191000	0.660121000
C	0.795403000	-2.886176000	0.433806000
N	-0.182446000	-1.916064000	0.401759000
H	4.925096000	-1.988043000	-0.183295000
H	5.282723000	0.665865000	-0.645121000
H	2.820508000	-3.512003000	0.306010000
H	3.663649000	2.751129000	-0.770663000
H	2.099476000	4.880630000	-0.944419000
H	-0.572930000	5.293570000	-0.686318000
Ti	0.102229000	0.132429000	0.289351000
H	-2.648268000	3.721263000	-0.189022000
H	-4.777214000	2.175575000	0.199233000
H	-5.134551000	-0.488398000	0.592135000
H	-3.507590000	-2.563991000	0.757373000
H	-1.950841000	-4.696850000	0.918624000
H	0.727493000	-5.106100000	0.734433000
S	0.424785000	0.538027000	2.671606000
C	-0.795888000	-0.414461000	3.289643000
N	-1.652535000	-1.077010000	3.748032000

TiPorph-ONO2 (m2)

C	2.563160000	-1.560409000	0.090649000
C	3.986704000	-1.391638000	-0.015338000
C	4.222119000	-0.048252000	-0.107781000
C	2.942561000	0.601822000	-0.056265000
N	1.944290000	-0.331938000	0.075843000
C	1.907912000	-2.790176000	0.158442000
C	2.723208000	1.973229000	-0.161201000
C	1.482650000	2.609981000	-0.205764000
C	1.312367000	4.027314000	-0.367933000
C	-0.031902000	4.266139000	-0.389788000
C	-0.681652000	2.994432000	-0.234942000
N	0.253101000	1.991856000	-0.127929000
C	-2.062506000	2.809060000	-0.200728000
C	-2.710423000	1.583007000	-0.070868000
C	-4.133922000	1.391874000	-0.090973000
C	-4.352807000	0.049722000	0.037272000
C	-3.063134000	-0.578793000	0.128582000
N	-2.071355000	0.375889000	0.074782000
C	-2.865990000	-1.956835000	0.208800000
C	-1.640015000	-2.620994000	0.219405000
C	-1.463985000	-4.045763000	0.214926000
C	-0.116475000	-4.278010000	0.202414000
C	0.528447000	-2.993509000	0.195774000
N	-0.416667000	-1.995647000	0.209897000
H	4.708049000	-2.207763000	-0.026366000
H	5.176245000	0.466398000	-0.214097000
H	2.537166000	-3.682778000	0.149637000
H	3.604769000	2.611940000	-0.249095000
H	2.129638000	4.741075000	-0.463089000
H	-0.551111000	5.216450000	-0.507267000
Ti	-0.056560000	0.036268000	0.671131000
H	-2.688244000	3.698630000	-0.295974000
H	-4.865933000	2.190506000	-0.203071000
H	-5.302067000	-0.484446000	0.052845000
H	-3.763461000	-2.579062000	0.223936000
H	-2.275915000	-4.771947000	0.215749000
H	0.406572000	-5.233456000	0.192720000
O	0.725704000	0.882292000	2.486915000
N	-0.049533000	0.208861000	3.251014000
O	-0.885328000	-0.507301000	2.598371000
O	0.008625000	0.238129000	4.449563000

TiPorph-Me (m2)

C	2.706158000	-1.460951000	0.081736000
C	4.117214000	-1.227305000	-0.079199000
C	4.289083000	0.122444000	-0.175111000
C	2.982760000	0.724903000	-0.090360000
N	2.030661000	-0.259504000	0.097902000
C	2.098570000	-2.703782000	0.191531000
C	2.728331000	2.081478000	-0.257563000
C	1.471669000	2.702037000	-0.301823000
C	1.230484000	4.092002000	-0.515556000
C	-0.133086000	4.275360000	-0.478099000
C	-0.720706000	2.995361000	-0.242992000
N	0.271419000	2.042745000	-0.144260000
C	-2.093624000	2.731141000	-0.132269000
C	-2.694134000	1.489679000	0.051317000
C	-4.115744000	1.258824000	0.064491000
C	-4.304476000	-0.088616000	0.169186000
C	-2.999783000	-0.692756000	0.236397000
N	-2.029567000	0.285206000	0.182105000
C	-2.735360000	-2.050288000	0.339099000
C	-1.465532000	-2.645652000	0.345681000
C	-1.232723000	-4.052605000	0.437306000
C	0.126326000	-4.237550000	0.391563000
C	0.719565000	-2.941391000	0.275664000
N	-0.263735000	-1.975277000	0.241773000
H	4.872721000	-2.010562000	-0.128325000
H	5.215364000	0.677815000	-0.318653000
H	2.750502000	-3.579727000	0.200172000
H	3.596504000	2.726085000	-0.412360000
H	2.006545000	4.841857000	-0.663573000
H	-0.691392000	5.204050000	-0.590550000
Ti	0.014931000	0.109927000	0.579291000
H	-2.764972000	3.586435000	-0.235846000
H	-4.868401000	2.042025000	-0.017886000
H	-5.243210000	-0.640986000	0.191111000
H	-3.592811000	-2.723286000	0.405236000
H	-2.015565000	-4.806205000	0.514722000
H	0.684448000	-5.172588000	0.422767000
C	0.064688000	0.524900000	2.624369000
H	-0.724679000	1.233875000	2.925941000
H	-0.110262000	-0.444998000	3.125693000
H	1.046671000	0.919224000	2.937859000

TiPorph-NO2 (m2)

C	2.662187000	-1.552047000	-0.003652000
C	4.082560000	-1.390821000	-0.162511000
C	4.317483000	-0.050032000	-0.282572000
C	3.039902000	0.605283000	-0.199114000

N	2.046783000	-0.326405000	-0.035940000
C	1.999221000	-2.769531000	0.152516000
C	2.828396000	1.981629000	-0.279406000
C	1.595620000	2.635948000	-0.249042000
C	1.419703000	4.052824000	-0.402900000
C	0.075032000	4.295982000	-0.366454000
C	-0.573663000	3.029090000	-0.184823000
N	0.367359000	2.027543000	-0.104897000
C	-1.951876000	2.831319000	-0.128633000
C	-2.603457000	1.602514000	-0.017947000
C	-4.031403000	1.428977000	0.001760000
C	-4.264472000	0.089429000	0.122956000
C	-2.978182000	-0.553283000	0.178640000
N	-1.980176000	0.383665000	0.079858000
C	-2.781257000	-1.928537000	0.305652000
C	-1.552039000	-2.585043000	0.333414000
C	-1.371706000	-4.008166000	0.373026000
C	-0.024904000	-4.239578000	0.334662000
C	0.620959000	-2.958388000	0.267255000
N	-0.327436000	-1.959230000	0.284429000
H	4.802002000	-2.208439000	-0.180417000
H	5.269328000	0.461413000	-0.419974000
H	2.617038000	-3.670004000	0.154079000
H	3.712064000	2.610913000	-0.405411000
H	2.232347000	4.766066000	-0.533487000
H	-0.443429000	5.248895000	-0.463443000
Ti	0.071364000	0.077750000	0.624661000
H	-2.582181000	3.720186000	-0.198528000
H	-4.755221000	2.238982000	-0.077077000
H	-5.219334000	-0.432951000	0.165050000
H	-3.675508000	-2.552805000	0.360349000
H	-2.182195000	-4.734808000	0.409787000
H	0.498340000	-5.194893000	0.335576000
N	-0.472492000	0.363564000	2.493063000
O	0.844173000	0.127310000	2.452154000
O	-1.051983000	0.571888000	3.528437000

TiPorph-ONO (m2)

C	2.665054000	-1.545743000	-0.002606000
C	4.082728000	-1.366658000	-0.121652000
C	4.308359000	-0.020539000	-0.226647000
C	3.028555000	0.624733000	-0.172124000
N	2.037555000	-0.320126000	-0.027615000
C	2.023480000	-2.785806000	0.093329000
C	2.827088000	2.005049000	-0.280492000
C	1.599323000	2.662459000	-0.262796000
C	1.396595000	4.077265000	-0.419720000
C	0.050677000	4.297633000	-0.339929000
C	-0.578170000	3.016692000	-0.149386000
N	0.387913000	2.035729000	-0.091650000
C	-1.956725000	2.809319000	-0.079788000
C	-2.623743000	1.583184000	0.022065000
C	-4.043595000	1.398683000	0.069023000
C	-4.271019000	0.049469000	0.171715000
C	-2.990007000	-0.592428000	0.187537000
N	-2.000613000	0.358617000	0.093136000
C	-2.759623000	-1.968802000	0.285502000
C	-1.523063000	-2.616609000	0.281508000
C	-1.342745000	-4.044091000	0.314066000
C	0.000326000	-4.282022000	0.238126000
C	0.650262000	-3.000644000	0.175318000
N	-0.292212000	-1.999926000	0.208892000
H	4.810264000	-2.177178000	-0.137014000
H	5.258451000	0.498602000	-0.346713000
H	2.664598000	-3.669805000	0.090913000
H	3.719036000	2.622642000	-0.405395000
H	2.191549000	4.805094000	-0.576241000
H	-0.484927000	5.243099000	-0.416548000
Ti	0.012824000	0.052262000	0.474912000
H	-2.584924000	3.700864000	-0.138234000
H	-4.776503000	2.203850000	0.036602000
H	-5.226316000	-0.469231000	0.241492000
H	-3.642690000	-2.608752000	0.345509000
H	-2.154064000	-4.768344000	0.375195000
H	0.517331000	-5.240633000	0.221941000
O	-0.098152000	0.218180000	2.333623000
N	-1.162451000	0.469980000	3.163623000
O	-0.852122000	0.544599000	4.309874000

VPorph-Cl (m3)

C	2.711292000	-1.476941000	-0.031677000
C	4.134158000	-1.251931000	-0.049464000
C	4.317911000	0.093007000	-0.187349000
C	3.006101000	0.686632000	-0.255433000
N	2.044960000	-0.288149000	-0.180123000
C	2.090389000	-2.708292000	0.162843000
C	2.740758000	2.050984000	-0.335027000
C	1.479963000	2.649254000	-0.307815000
C	1.248007000	4.058868000	-0.396225000
C	-0.107441000	4.246818000	-0.352100000
C	-0.708418000	2.952237000	-0.234639000
N	0.275632000	1.983681000	-0.197992000
C	-2.083770000	2.722370000	-0.180285000
C	-2.709215000	1.482907000	-0.065885000
C	-4.129470000	1.267994000	0.049184000
C	-4.313341000	-0.076818000	0.187813000
C	-3.004079000	-0.679751000	0.159929000
N	-2.046344000	0.285273000	-0.009920000
C	-2.734266000	-2.036526000	0.318958000
C	-1.470371000	-2.627944000	0.357793000
C	-1.235964000	-4.032227000	0.506788000
C	0.119394000	-4.220586000	0.462951000
C	0.717779000	-2.931822000	0.284353000
N	-0.265802000	-1.964160000	0.237137000
H	4.887456000	-2.031586000	0.055126000
H	5.252834000	0.650999000	-0.220272000
H	2.737601000	-3.583911000	0.245866000
H	3.598907000	2.722989000	-0.402317000
H	2.031563000	4.809767000	-0.487459000
H	-0.663024000	5.182427000	-0.400230000
V	-0.017100000	-0.031726000	-0.381124000
H	-2.728176000	3.603281000	-0.215655000
H	-4.880245000	2.057100000	0.035754000
H	-5.246323000	-0.624917000	0.313256000
H	-3.589760000	-2.705308000	0.435106000
H	-2.018729000	-4.780822000	0.620529000

H	0.675399000	-5.154514000	0.533761000
Cl	-0.225306000	-0.297051000	-2.615148000

VPorph-Cl-OH (m2)

C	2.722577000	-1.454675000	0.126774000
C	4.132939000	-1.234924000	-0.009230000
C	4.321091000	0.111201000	-0.167549000
C	3.026619000	0.728088000	-0.130457000
N	2.065577000	-0.241108000	0.065583000
C	2.117384000	-2.700153000	0.281700000
C	2.779244000	2.090578000	-0.285972000
C	1.526272000	2.709045000	-0.279617000
C	1.283431000	4.114785000	-0.451275000
C	-0.070154000	4.296696000	-0.380658000
C	-0.660864000	3.003005000	-0.166369000
N	0.335636000	2.056853000	-0.106567000
C	-2.029234000	2.760625000	-0.043995000
C	-2.654122000	1.523012000	0.150215000
C	-4.076116000	1.302774000	0.228624000
C	-4.259126000	-0.042966000	0.381836000
C	-2.948028000	-0.643439000	0.397184000
N	-2.002802000	0.331826000	0.265358000
C	-2.682968000	-2.012464000	0.507484000
C	-1.431920000	-2.634138000	0.492263000
C	-1.211035000	-4.053180000	0.595760000
C	0.139740000	-4.252643000	0.531566000
C	0.745973000	-2.954000000	0.390341000
N	-0.225095000	-1.997015000	0.371225000
H	4.884582000	-2.022822000	0.012124000
H	5.257587000	0.650440000	-0.303259000
H	2.783067000	-3.565197000	0.308839000
H	3.648014000	2.734553000	-0.435771000
H	2.059058000	4.863929000	-0.602854000
H	-0.632352000	5.225878000	-0.463851000
V	0.082013000	0.052668000	0.242969000
H	-2.683986000	3.631662000	-0.117215000
H	-4.830839000	2.085460000	0.165012000
H	-5.194786000	-0.593471000	0.470947000
H	-3.549301000	-2.670325000	0.606329000
H	-1.999683000	-4.796765000	0.702066000
H	0.689066000	-5.192106000	0.574969000
Cl	-0.278947000	-0.242128000	-2.071751000
O	-0.015375000	0.201016000	1.996352000
H	-0.812648000	0.115324000	2.543586000

VPorph-Cl-Cl (m2)

C	2.676532000	-1.458811000	0.040010000
C	4.095885000	-1.239580000	-0.044024000
C	4.278458000	0.107293000	-0.195679000
C	2.968967000	0.704842000	-0.207483000
N	2.020941000	-0.266560000	-0.063004000
C	2.057851000	-2.702248000	0.202792000
C	2.705790000	2.071209000	-0.341961000
C	1.453216000	2.682068000	-0.356936000
C	1.229079000	4.094781000	-0.464385000
C	-0.125171000	4.291279000	-0.420327000
C	-0.730854000	2.997359000	-0.286400000
N	0.242203000	2.034302000	-0.255195000
C	-2.102844000	2.751178000	-0.190083000
C	-2.705736000	1.507476000	-0.032201000
C	-4.117425000	1.276821000	0.046491000
C	-4.300836000	-0.071735000	0.188539000
C	-3.001922000	-0.677736000	0.204073000
N	-2.037120000	0.302233000	0.068958000
C	-2.754148000	-2.040008000	0.333677000
C	-1.498482000	-2.652855000	0.354549000
C	-1.263099000	-4.063283000	0.463435000
C	0.093489000	-4.244803000	0.427998000
C	0.689319000	-2.945893000	0.295890000
N	-0.303009000	-1.991163000	0.259137000
H	4.849759000	-2.023505000	0.010930000
H	5.212784000	0.658487000	-0.291835000
H	2.713549000	-3.573752000	0.254625000
H	3.570405000	2.731412000	-0.436155000
H	2.016887000	4.840977000	-0.555415000
H	-0.674990000	5.230025000	-0.467677000
V	-0.046966000	0.024156000	0.006023000
H	-2.765974000	3.616786000	-0.238944000
H	-4.873629000	2.058547000	-0.006412000
H	-5.237351000	-0.620398000	0.276519000
H	-3.624216000	-2.693395000	0.420454000
H	-2.043884000	-4.817253000	0.550150000
H	0.652550000	-5.177938000	0.479092000
Cl	-0.015222000	-0.302525000	-2.265918000
Cl	0.236356000	0.315347000	2.262578000

VPorph-Cl-F (m2)

C	2.689921000	-1.464427000	0.044040000
C	4.104011000	-1.242340000	-0.049458000
C	4.291195000	0.106110000	-0.189894000
C	2.990873000	0.713259000	-0.186008000
N	2.031921000	-0.258253000	-0.037626000
C	2.082072000	-2.710533000	0.188816000
C	2.738307000	2.078536000	-0.319754000
C	1.485232000	2.688787000	-0.326354000
C	1.251002000	4.096284000	-0.463524000
C	-0.104101000	4.285038000	-0.421996000
C	-0.705965000	2.993631000	-0.258240000
N	0.280977000	2.033905000	-0.199907000
C	-2.074906000	2.751383000	-0.171990000
C	-2.685769000	1.506389000	-0.008402000
C	-4.101378000	1.277495000	0.055515000
C	-4.281048000	-0.071589000	0.197452000
C	-2.975050000	-0.669170000	0.223121000
N	-2.022361000	0.312982000	0.102765000
C	-2.722411000	-2.035966000	0.338293000
C	-1.470299000	-2.653846000	0.352125000
C	-1.244613000	-4.069561000	0.444398000
C	0.109645000	-4.260340000	0.402391000
C	0.711346000	-2.960220000	0.284267000
N	-0.267642000	-2.004066000	0.263685000
H	4.856907000	-2.028095000	-0.008390000
H	5.228073000	0.652436000	-0.288556000
H	2.744647000	-3.577453000	0.224557000
H	3.605018000	2.733152000	-0.428632000
H	2.033679000	4.845101000	-0.574420000

H	-0.658801000	5.219557000	-0.491878000
V	0.011816000	0.036784000	0.060587000
H	-2.733871000	3.618990000	-0.239529000
H	-4.858401000	2.057866000	-0.005822000
H	-5.215469000	-0.625237000	0.277222000
H	-3.592903000	-2.690394000	0.415766000
H	-2.030693000	-4.818600000	0.527338000
H	0.663455000	-5.197141000	0.443495000
Cl	-0.164368000	-0.299780000	-2.225050000
F	0.070596000	0.176158000	1.821702000

VPorph-Cl-N3 (m2)

C	2.643318000	-1.393889000	0.226675000
C	4.063791000	-1.175144000	0.161944000
C	4.250619000	0.167468000	-0.018782000
C	2.942526000	0.766428000	-0.064348000
N	1.991275000	-0.198245000	0.086281000
C	2.024579000	-2.634460000	0.407730000
C	2.676814000	2.129225000	-0.245202000
C	1.425247000	2.735854000	-0.306465000
C	1.198520000	4.138217000	-0.496845000
C	-0.156250000	4.334353000	-0.499951000
C	-0.769544000	3.052834000	-0.310033000
N	0.207345000	2.089368000	-0.194947000
C	-2.141380000	2.811181000	-0.248764000
C	-2.748352000	1.570463000	-0.058695000
C	-4.159875000	1.333312000	0.010880000
C	-4.334559000	-0.010374000	0.204245000
C	-3.031321000	-0.606847000	0.253942000
N	-2.074240000	0.378921000	0.091511000
C	-2.781384000	-1.963326000	0.437806000
C	-1.528474000	-2.587413000	0.490743000
C	-1.301404000	-3.995754000	0.675240000
C	0.053598000	-4.181916000	0.664656000
C	0.651718000	-2.887311000	0.473129000
N	-0.334148000	-1.941245000	0.370669000
H	4.816254000	-1.957616000	0.247312000
H	5.187548000	0.714419000	-0.113987000
H	2.685366000	-3.498216000	0.506732000
H	3.540946000	2.788204000	-0.351810000
H	1.986636000	4.880440000	-0.613566000
H	-0.702334000	5.268859000	-0.619870000
V	-0.077177000	0.113066000	0.054802000
H	-2.801757000	3.673819000	-0.354087000
H	-4.921506000	2.106247000	-0.079154000
H	-5.268476000	-0.561289000	0.305913000
H	-3.655550000	-2.608067000	0.549388000
H	-2.085592000	-4.741278000	0.797317000
H	0.610067000	-5.111306000	0.776903000
Cl	0.017254000	-0.340164000	-2.186614000
N	0.093988000	0.221782000	2.000886000
N	0.723272000	-0.446267000	2.774324000
N	1.318698000	-1.072823000	3.537997000

VPorph-Cl-SCN (m2)

C	2.743298000	-1.396068000	0.034899000
C	4.151040000	-1.187032000	-0.115929000
C	4.334466000	0.136784000	-0.405722000
C	3.040373000	0.750219000	-0.423209000
N	2.069897000	-0.204845000	-0.176283000
C	2.145206000	-2.609870000	0.344029000
C	2.801427000	2.104365000	-0.611085000
C	1.554393000	2.727955000	-0.539867000
C	1.321519000	4.131202000	-0.694773000
C	-0.023021000	4.334371000	-0.527579000
C	-0.614214000	3.055170000	-0.276815000
N	0.368465000	2.082389000	-0.292749000
C	-1.973470000	2.837415000	-0.073469000
C	-2.595527000	1.605119000	0.132549000
C	-4.007815000	1.412724000	0.306292000
C	-4.206468000	0.068576000	0.462967000
C	-2.912484000	-0.551448000	0.392433000
N	-1.951493000	0.398700000	0.186083000
C	-2.662901000	-1.916535000	0.541174000
C	-1.412124000	-2.522905000	0.538637000
C	-1.180102000	-3.913358000	0.773396000
C	0.176274000	-4.107174000	0.748532000
C	0.775943000	-2.837015000	0.482171000
N	-0.202177000	-1.876401000	0.353742000
H	4.903341000	-1.966614000	-0.009177000
H	5.268323000	0.667728000	-0.583543000
H	2.806328000	-3.467440000	0.479265000
H	3.666605000	2.741043000	-0.803062000
H	2.095922000	4.866553000	-0.906263000
H	-0.577392000	5.270270000	-0.574959000
V	0.076499000	0.059967000	-0.172735000
H	-2.618279000	3.717968000	-0.092398000
H	-4.746677000	2.212507000	0.304525000
H	-5.141827000	-0.466403000	0.619402000
H	-3.524745000	-2.565464000	0.707115000
H	-1.964715000	-4.649390000	0.940001000
H	0.730027000	-5.034111000	0.888464000
Cl	-0.320951000	-0.226682000	-2.352139000
S	0.302604000	0.611815000	2.349578000
C	-0.648919000	-0.528401000	3.081217000
N	-1.321975000	-1.328415000	3.624965000

VPorph-Cl-ONO2 (m2)

C	2.632196000	-1.445902000	0.149619000
C	4.054053000	-1.260511000	0.095739000
C	4.278185000	0.073887000	-0.105746000
C	2.992719000	0.708994000	-0.170055000
N	2.005421000	-0.229733000	-0.023732000
C	1.992467000	-2.666599000	0.342125000
C	2.771570000	2.078516000	-0.323790000
C	1.532400000	2.710872000	-0.322251000
C	1.323074000	4.122560000	-0.428797000
C	-0.026959000	4.340013000	-0.360265000
C	-0.658147000	3.062890000	-0.220780000
N	0.311418000	2.072633000	-0.199820000
C	-2.029967000	2.854588000	-0.139876000
C	-2.679057000	1.619999000	-0.038903000
C	-4.100549000	1.429930000	0.026174000
C	-4.316257000	0.081360000	0.111915000
C	-3.026169000	-0.549811000	0.111257000
N	-2.048463000	0.408729000	0.006748000

C	-2.805096000	-1.919277000	0.244440000
C	-1.564063000	-2.555367000	0.329772000
C	-1.364256000	-3.968324000	0.476744000
C	-0.010922000	-4.176676000	0.516895000
C	0.612169000	-2.890740000	0.389058000
N	-0.348520000	-1.921669000	0.289869000
H	4.785848000	-2.059395000	0.204373000
H	5.230270000	0.594720000	-0.195521000
H	2.635014000	-3.542808000	0.448597000
H	3.651120000	2.716498000	-0.427034000
H	2.119001000	4.857306000	-0.538680000
H	-0.560736000	5.288052000	-0.405167000
V	-0.012230000	0.103328000	-0.055074000
H	-2.662667000	3.743631000	-0.167587000
H	-4.835440000	2.233268000	0.007557000
H	-5.264369000	-0.449747000	0.181834000
H	-3.689005000	-2.557212000	0.305485000
H	-2.163876000	-4.705055000	0.536028000
H	0.526018000	-5.118918000	0.615199000
Cl	-0.157250000	-0.309987000	-2.276933000
O	-0.007044000	0.282668000	1.873170000
N	-0.535872000	-0.573087000	2.758848000
O	-1.719346000	-0.454792000	2.991000000
O	0.227357000	-1.360551000	3.272529000

VPorph-Cl-Me (m2)

C	2.689165000	-1.433395000	0.058628000
C	4.104739000	-1.216958000	-0.006344000
C	4.302861000	0.130843000	-0.148747000
C	3.007283000	0.744627000	-0.172707000
N	2.037921000	-0.218005000	-0.047014000
C	2.083073000	-2.680396000	0.208226000
C	2.758992000	2.113302000	-0.310207000
C	1.507171000	2.721167000	-0.336725000
C	1.272277000	4.129328000	-0.464590000
C	-0.081833000	4.319245000	-0.425634000
C	-0.689213000	3.028961000	-0.271886000
N	0.298398000	2.059168000	-0.230006000
C	-2.056247000	2.796085000	-0.166432000
C	-2.674272000	1.551105000	0.008094000
C	-4.089680000	1.320866000	0.097731000
C	-4.262707000	-0.028319000	0.249804000
C	-2.954038000	-0.622869000	0.253042000
N	-2.007387000	0.365816000	0.107679000
C	-2.702683000	-1.991027000	0.375230000
C	-1.458953000	-2.633818000	0.364458000
C	-1.242176000	-4.053003000	0.513607000
C	0.108772000	-4.250287000	0.466026000
C	0.710455000	-2.949343000	0.286758000
N	-0.260787000	-2.002625000	0.222773000
H	4.853816000	-2.005504000	0.050681000
H	5.245503000	0.669783000	-0.233014000
H	2.756679000	-3.537573000	0.275266000
H	3.627717000	2.768065000	-0.401172000
H	2.055151000	4.879293000	-0.567084000
H	-0.633645000	5.256037000	-0.489825000
V	0.026030000	0.076366000	-0.075462000
H	-2.709416000	3.669111000	-0.222102000
H	-4.850848000	2.097883000	0.042918000
H	-5.194941000	-0.583279000	0.346108000
H	-3.579918000	-2.632024000	0.489588000
H	-2.030152000	-4.793854000	0.641169000
H	0.660467000	-5.185954000	0.546971000
Cl	-0.268914000	-0.467484000	-2.320631000
C	-0.002933000	-0.045809000	2.020524000
H	-0.920464000	-0.497494000	2.420670000
H	0.876230000	-0.595970000	2.386422000
H	0.058952000	1.011934000	2.331373000

VPorph-Cl-NO2 (m2)

C	2.611941000	-1.410134000	0.226350000
C	4.027805000	-1.212089000	0.190254000
C	4.247258000	0.133376000	0.038281000
C	2.965786000	0.767639000	-0.020639000
N	1.979350000	-0.183363000	0.126231000
C	1.984326000	-2.655883000	0.287551000
C	2.752531000	2.128364000	-0.254551000
C	1.519087000	2.762956000	-0.394202000
C	1.320414000	4.178547000	-0.571940000
C	-0.027438000	4.395162000	-0.562672000
C	-0.657386000	3.112724000	-0.378256000
N	0.303039000	2.138348000	-0.313907000
C	-2.025071000	2.895619000	-0.220145000
C	-2.648488000	1.669883000	0.026448000
C	-4.062832000	1.467689000	0.102007000
C	-4.272018000	0.121960000	0.263317000
C	-2.986984000	-0.507319000	0.286772000
N	-2.005704000	0.460915000	0.173214000
C	-2.781183000	-1.886707000	0.344201000
C	-1.557173000	-2.557304000	0.279095000
C	-1.364446000	-3.980703000	0.422363000
C	-0.017249000	-4.198009000	0.405689000
C	0.610488000	-2.906568000	0.252385000
N	-0.348312000	-1.947139000	0.138206000
H	4.764944000	-2.011338000	0.250961000
H	5.199198000	0.654789000	-0.051111000
H	2.642339000	-3.523048000	0.373309000
H	3.642944000	2.754348000	-0.340129000
H	2.122465000	4.908715000	-0.669117000
H	-0.562275000	5.339757000	-0.650818000
V	-0.014143000	0.138506000	0.033609000
H	-2.677531000	3.767317000	-0.299276000
H	-4.805935000	2.258861000	0.014624000
H	-5.220215000	-0.408805000	0.335164000
H	-3.676118000	-2.503890000	0.446882000
H	-2.166926000	-4.707769000	0.537695000
H	0.519164000	-5.140563000	0.505579000
Cl	-0.123312000	-0.227569000	-2.217141000
N	-0.061203000	-0.250573000	2.050263000
O	-0.186690000	-1.032210000	2.947295000
O	0.147821000	0.971635000	2.151728000

VPorph-Cl-ONO (m2)

C	2.600172000	-1.484146000	0.150313000
C	4.018252000	-1.275479000	0.116703000
C	4.219768000	0.076977000	0.050168000

C	2.924376000	0.695077000	0.026353000
N	1.953073000	-0.279769000	0.098354000
C	1.961349000	-2.723994000	0.232970000
C	2.701460000	2.065359000	-0.094313000
C	1.464275000	2.708928000	-0.202197000
C	1.273679000	4.130681000	-0.317191000
C	-0.075720000	4.341520000	-0.384173000
C	-0.701020000	3.046412000	-0.307624000
N	0.253620000	2.079433000	-0.209365000
C	-2.079542000	2.810789000	-0.294812000
C	-2.711739000	1.577356000	-0.152464000
C	-4.131023000	1.390481000	-0.075910000
C	-4.352818000	0.052220000	0.111767000
C	-3.068294000	-0.582376000	0.145607000
N	-2.082460000	0.355453000	-0.028446000
C	-2.842656000	-1.947446000	0.333210000
C	-1.602218000	-2.574948000	0.371609000
C	-1.394592000	-3.984231000	0.503988000
C	-0.044126000	-4.204084000	0.450531000
C	0.590362000	-2.931019000	0.303069000
N	-0.378211000	-1.937575000	0.250906000
H	4.762759000	-2.069782000	0.142103000
H	5.163147000	0.619262000	0.006699000
H	2.597444000	-3.610343000	0.266063000
H	3.588687000	2.701416000	-0.120641000
H	2.076574000	4.866164000	-0.337777000
H	-0.611012000	5.285943000	-0.471260000
V	-0.069258000	0.000791000	-0.087178000
H	-2.730122000	3.684244000	-0.372991000
H	-4.864295000	2.192152000	-0.150083000
H	-5.303444000	-0.467171000	0.222762000
H	-3.721176000	-2.584815000	0.448945000
H	-2.192680000	-4.716991000	0.611872000
H	0.487064000	-5.152819000	0.508975000
Cl	0.099984000	-0.113133000	-2.349027000
O	-0.088665000	0.491482000	1.844201000
N	0.271686000	-0.184869000	2.905216000
O	0.850729000	-1.224075000	2.747027000

Vporph (m4)

C	2.711737000	-1.478560000	0.037959000
C	4.132024000	-1.254148000	-0.048147000
C	4.316691000	0.095095000	-0.186865000
C	3.009202000	0.701513000	-0.188585000
N	2.054318000	-0.275880000	-0.049995000
C	2.086167000	-2.723352000	0.186439000
C	2.741293000	2.070364000	-0.315347000
C	1.486297000	2.692894000	-0.327340000
C	1.252945000	4.108630000	-0.451402000
C	-0.102170000	4.296493000	-0.410836000
C	-0.704462000	2.995781000	-0.262717000
N	0.282138000	2.039441000	-0.216367000
C	-2.078740000	2.739201000	-0.176649000
C	-2.704499000	1.494312000	-0.029554000
C	-4.124869000	1.269663000	0.054839000
C	-4.309438000	-0.079582000	0.193561000
C	-3.001816000	-0.685758000	0.196837000
N	-2.047000000	0.291792000	0.059073000
C	-2.733774000	-2.054463000	0.324776000
C	-1.478762000	-2.676950000	0.338028000
C	-1.245564000	-4.092789000	0.461263000
C	0.109549000	-4.280734000	0.420856000
C	0.711922000	-2.979912000	0.273675000
N	-0.274533000	-2.023506000	0.228297000
H	4.890557000	-2.035603000	-0.005617000
H	5.256130000	0.639474000	-0.282039000
H	2.745513000	-3.593741000	0.236940000
H	3.608751000	2.728127000	-0.414267000
H	2.033341000	4.862325000	-0.554266000
H	-0.653979000	5.234293000	-0.473742000
V	0.003755000	0.007941000	0.004515000
H	-2.737990000	3.609642000	-0.227573000
H	-4.883491000	2.050980000	0.011382000
H	-5.248847000	-0.624170000	0.287840000
H	-3.601284000	-2.712259000	0.423160000
H	-2.026035000	-4.846482000	0.563604000
H	0.661322000	-5.218583000	0.483324000

VPorph-OH (m3)

C	2.740127000	-1.454848000	0.157755000
C	4.156543000	-1.243839000	-0.012715000
C	4.339630000	0.099484000	-0.165338000
C	3.034453000	0.709571000	-0.090179000
N	2.082007000	-0.251409000	0.135866000
C	2.115184000	-2.694620000	0.271537000
C	2.764817000	2.063178000	-0.275243000
C	1.501007000	2.662595000	-0.307470000
C	1.267686000	4.065840000	-0.484655000
C	-0.086520000	4.257937000	-0.419119000
C	-0.681721000	2.971550000	-0.201501000
N	0.300344000	2.006956000	-0.156785000
C	-2.052636000	2.745897000	-0.043281000
C	-2.670484000	1.517664000	0.182307000
C	-4.093662000	1.293762000	0.227638000
C	-4.277555000	-0.050217000	0.375826000
C	-2.966009000	-0.648096000	0.423247000
N	-2.004114000	0.327021000	0.331713000
C	-2.703461000	-2.014062000	0.493289000
C	-1.445613000	-2.622530000	0.439619000
C	-1.217743000	-4.035175000	0.519907000
C	0.136898000	-4.225563000	0.457506000
C	0.737959000	-2.928849000	0.339212000
N	-0.243215000	-1.963331000	0.313088000
H	4.904775000	-2.035498000	-0.032305000
H	5.269043000	0.641575000	-0.336733000
H	2.761232000	-3.575282000	0.285621000
H	3.621297000	2.722949000	-0.431482000
H	2.049163000	4.810421000	-0.630841000
H	-0.642214000	5.191441000	-0.500381000
V	0.053854000	0.066821000	0.508659000
H	-2.703937000	3.618881000	-0.126151000
H	-4.848330000	2.073398000	0.131182000
H	-5.213867000	-0.604817000	0.427340000
H	-3.564635000	-2.681193000	0.573846000
H	-2.001674000	-4.785062000	0.618160000
H	0.690977000	-5.162874000	0.493273000

O	0.150440000	0.268797000	2.295030000
H	-0.711347000	0.424116000	2.714087000

VPorph-F (m3)

C	2.713507000	-1.458996000	0.073961000
C	4.132423000	-1.245417000	-0.061904000
C	4.316669000	0.099255000	-0.200101000
C	3.009487000	0.705897000	-0.149401000
N	2.052764000	-0.258006000	0.038553000
C	2.088150000	-2.698975000	0.179624000
C	2.741394000	2.062130000	-0.313733000
C	1.477756000	2.657007000	-0.347567000
C	1.242801000	4.058866000	-0.518936000
C	-0.112896000	4.247537000	-0.474278000
C	-0.709962000	2.961157000	-0.274219000
N	0.274516000	1.996816000	-0.211570000
C	-2.083507000	2.734651000	-0.157388000
C	-2.703805000	1.503010000	0.036017000
C	-4.127050000	1.277273000	0.046477000
C	-4.311727000	-0.067573000	0.182425000
C	-3.000326000	-0.662477000	0.254815000
N	-2.038520000	0.313339000	0.188070000
C	-2.737080000	-2.027300000	0.330090000
C	-1.476582000	-2.628680000	0.301290000
C	-1.244711000	-4.038142000	0.392999000
C	0.111249000	-4.225936000	0.349724000
C	0.711848000	-2.931174000	0.230949000
N	-0.272577000	-1.964877000	0.189463000
H	4.881864000	-2.035977000	-0.065712000
H	5.248599000	0.645189000	-0.342265000
H	2.733303000	-3.579702000	0.207566000
H	3.597532000	2.726825000	-0.447819000
H	2.024695000	4.805602000	-0.649959000
H	-0.669350000	5.179868000	-0.561780000
V	0.018499000	0.050866000	0.387729000
H	-2.732465000	3.608099000	-0.250046000
H	-4.880026000	2.056986000	-0.060525000
H	-5.247139000	-0.625086000	0.211225000
H	-3.596645000	-2.697754000	0.395851000
H	-2.027892000	-4.789079000	0.487706000
H	0.666842000	-5.161402000	0.401881000
F	0.123647000	0.220142000	2.162026000

VPorph-N3 (m3)

C	2.673141000	-1.381410000	0.101177000
C	4.083526000	-1.156698000	-0.017952000
C	4.262022000	0.187724000	-0.206215000
C	2.960764000	0.789894000	-0.201664000
N	2.001917000	-0.184086000	-0.028771000
C	2.083086000	-2.626783000	0.335080000
C	2.715236000	2.158690000	-0.339197000
C	1.468773000	2.783326000	-0.325065000
C	1.231159000	4.184991000	-0.561537000
C	-0.121163000	4.367387000	-0.529610000
C	-0.710174000	3.076417000	-0.273345000
N	0.276265000	2.135027000	-0.130791000
C	-2.076449000	2.804528000	-0.223016000
C	-2.670498000	1.549501000	-0.061433000
C	-4.082033000	1.318938000	0.024887000
C	-4.258867000	-0.024132000	0.224923000
C	-2.955500000	-0.620223000	0.258914000
N	-1.997951000	0.353039000	0.064290000
C	-2.706575000	-1.976701000	0.483926000
C	-1.457415000	-2.594086000	0.544479000
C	-1.223418000	-4.006230000	0.709319000
C	0.128779000	-4.190499000	0.663393000
C	0.720833000	-2.889902000	0.472546000
N	-0.261932000	-1.936151000	0.418800000
H	4.841527000	-1.936282000	0.045769000
H	5.196143000	0.734117000	-0.330508000
H	2.761446000	-3.479886000	0.405417000
H	3.587792000	2.798020000	-0.490426000
H	2.009547000	4.924365000	-0.745238000
H	-0.683878000	5.287425000	-0.683292000
V	0.014207000	0.130008000	0.371708000
H	-2.755097000	3.653580000	-0.329781000
H	-4.841472000	2.096287000	-0.048163000
H	-5.192474000	-0.570918000	0.351397000
H	-3.581694000	-2.617488000	0.612611000
H	-2.004451000	-4.755823000	0.829253000
H	0.687830000	-5.122422000	0.737940000
N	0.103988000	0.331045000	2.304841000
N	0.054227000	-0.561543000	3.116960000
N	0.014514000	-1.364160000	3.943299000

VPorph-SCN (m3)

C	2.652556000	-1.396027000	0.045670000
C	4.063451000	-1.198073000	-0.096219000
C	4.262320000	0.138190000	-0.320970000
C	2.973388000	0.762891000	-0.315279000
N	1.997922000	-0.190184000	-0.103608000
C	2.045529000	-2.624783000	0.313911000
C	2.752740000	2.132081000	-0.477201000
C	1.519830000	2.782391000	-0.446278000
C	1.304469000	4.183615000	-0.698299000
C	-0.042657000	4.395373000	-0.631089000
C	-0.650786000	3.122871000	-0.338307000
N	0.319280000	2.161255000	-0.210412000
C	-2.020328000	2.881115000	-0.241992000
C	-2.632403000	1.642584000	-0.038267000
C	-4.044361000	1.440597000	0.083836000
C	-4.242479000	0.104554000	0.311265000
C	-2.952093000	-0.516679000	0.327341000
N	-1.978061000	0.434616000	0.099466000
C	-2.725970000	-1.874756000	0.560976000
C	-1.487806000	-2.514437000	0.597336000
C	-1.277600000	-3.926077000	0.786879000
C	0.069252000	-4.138317000	0.714407000
C	0.681373000	-2.855664000	0.481037000
N	-0.282723000	-1.881589000	0.431095000
H	4.808889000	-1.988437000	-0.020639000
H	5.203852000	0.665678000	-0.468052000
H	2.708835000	-3.488677000	0.392241000
H	3.634614000	2.749949000	-0.659450000
H	2.092927000	4.903033000	-0.914809000
H	-0.590552000	5.324443000	-0.782973000

V	0.029922000	0.169604000	0.310172000
H	-2.684856000	3.741215000	-0.347279000
H	-4.790074000	2.231079000	0.012338000
H	-5.183377000	-0.422079000	0.465389000
H	-3.609938000	-2.495871000	0.720569000
H	-2.070339000	-4.656708000	0.941046000
H	0.612141000	-5.078878000	0.797827000
S	0.225835000	0.681340000	2.621557000
C	0.151810000	-0.826401000	3.335842000
N	0.109619000	-1.865656000	3.882267000

VPorph-ONO2 (m3)

C	2.688844000	-1.561342000	0.005286000
C	4.112034000	-1.378679000	-0.121746000
C	4.330345000	-0.034737000	-0.220767000
C	3.040177000	0.603534000	-0.154181000
N	2.060366000	-0.342657000	0.001559000
C	2.031088000	-2.788059000	0.081988000
C	2.805919000	1.972583000	-0.269336000
C	1.559716000	2.604307000	-0.271536000
C	1.361144000	4.019054000	-0.381226000
C	0.011913000	4.242156000	-0.316347000
C	-0.619390000	2.964452000	-0.166767000
N	0.339510000	1.973615000	-0.154465000
C	-1.996521000	2.767503000	-0.041740000
C	-2.648296000	1.545613000	0.114001000
C	-4.075031000	1.357671000	0.154741000
C	-4.294425000	0.013348000	0.249292000
C	-3.001134000	-0.619930000	0.266678000
N	-2.014297000	0.332266000	0.209310000
C	-2.772061000	-1.993820000	0.299213000
C	-1.529763000	-2.629359000	0.236921000
C	-1.333389000	-4.046615000	0.305045000
C	0.016112000	-4.269032000	0.243558000
C	0.650075000	-2.988298000	0.137376000
N	-0.309385000	-1.997629000	0.119514000
H	4.842217000	-2.186596000	-0.145694000
H	5.276559000	0.490636000	-0.344117000
H	2.654731000	-3.684482000	0.090662000
H	3.679855000	2.618045000	-0.380464000
H	2.161641000	4.749898000	-0.487222000
H	-0.519177000	5.192167000	-0.358553000
V	0.038453000	0.005421000	0.325567000
H	-2.623560000	3.660350000	-0.088090000
H	-4.808607000	2.160701000	0.097057000
H	-5.244692000	-0.517684000	0.286789000
H	-3.648498000	-2.641906000	0.366135000
H	-2.134379000	-4.778437000	0.399795000
H	0.546979000	-5.219512000	0.278010000
O	0.157470000	0.120100000	2.201240000
N	-0.945935000	0.339954000	2.968423000
O	-1.528580000	-0.642817000	3.364760000
O	-1.203725000	1.494370000	3.219526000

VPorph-Me (m3)

C	2.665575000	-1.438934000	0.049477000
C	4.075665000	-1.215145000	-0.058217000
C	4.258318000	0.135851000	-0.203486000
C	2.959241000	0.739363000	-0.186650000
N	1.997286000	-0.237073000	-0.042927000
C	2.066202000	-2.690548000	0.231743000
C	2.714260000	2.112715000	-0.294076000
C	1.469005000	2.737812000	-0.278633000
C	1.236644000	4.147369000	-0.477908000
C	-0.114594000	4.333201000	-0.446083000
C	-0.709482000	3.036771000	-0.227365000
N	0.272954000	2.086167000	-0.109165000
C	-2.076033000	2.770838000	-0.182671000
C	-2.676622000	1.513018000	-0.056138000
C	-4.088558000	1.284779000	0.012205000
C	-4.270268000	-0.065345000	0.164151000
C	-2.968642000	-0.663776000	0.189559000
N	-2.006986000	0.312113000	0.039454000
C	-2.722899000	-2.030869000	0.358337000
C	-1.475601000	-2.649452000	0.410444000
C	-1.247908000	-4.071207000	0.495278000
C	0.103177000	-4.257575000	0.457084000
C	0.702503000	-2.948813000	0.351454000
N	-0.276142000	-1.986779000	0.345763000
H	4.832543000	-1.997695000	-0.019020000
H	5.194577000	0.682839000	-0.308793000
H	2.737907000	-3.551390000	0.263962000
H	3.588465000	2.754997000	-0.422828000
H	2.017979000	4.889493000	-0.637091000
H	-0.673715000	5.259462000	-0.574377000
V	0.010674000	0.077180000	0.357999000
H	-2.750694000	3.625685000	-0.267898000
H	-4.846264000	2.065678000	-0.041086000
H	-5.206498000	-0.613893000	0.261507000
H	-3.599715000	-2.677265000	0.439718000
H	-2.033128000	-4.823816000	0.557713000
H	0.658973000	-5.194294000	0.481141000
C	0.197645000	0.306157000	2.393368000
H	-0.802175000	0.605463000	2.758378000
H	0.496888000	-0.622968000	2.907056000
H	0.908252000	1.112400000	2.642508000

VPorph-NO2 (m3)

C	2.656866000	-1.416068000	0.198800000
C	4.080449000	-1.231143000	0.096392000
C	4.294586000	0.108964000	-0.054848000
C	3.000891000	0.741014000	-0.044892000
N	2.020996000	-0.201261000	0.140499000
C	2.005248000	-2.646017000	0.273308000
C	2.762951000	2.095911000	-0.266543000
C	1.514065000	2.709926000	-0.375966000
C	1.314399000	4.116092000	-0.589760000
C	-0.034367000	4.333017000	-0.572365000
C	-0.659840000	3.059434000	-0.348509000
N	0.298406000	2.076348000	-0.263527000
C	-2.036044000	2.869262000	-0.211759000
C	-2.683982000	1.656119000	0.011795000
C	-4.110058000	1.462934000	0.035542000
C	-4.324214000	0.122395000	0.182670000
C	-3.028229000	-0.501332000	0.248457000
N	-2.045071000	0.452870000	0.170873000

C	-2.795707000	-1.873333000	0.317499000
C	-1.551728000	-2.504714000	0.280068000
C	-1.354222000	-3.922832000	0.373134000
C	-0.004748000	-4.139645000	0.359736000
C	0.625926000	-2.854022000	0.260027000
N	-0.333750000	-1.869042000	0.179152000
H	4.813611000	-2.036523000	0.114447000
H	5.239746000	0.633664000	-0.188926000
H	2.634482000	-3.537182000	0.323948000
H	3.637129000	2.738895000	-0.390475000
H	2.115701000	4.841007000	-0.726104000
H	-0.571986000	5.272762000	-0.692274000
V	-0.005428000	0.149657000	0.458369000
H	-2.665536000	3.755896000	-0.313186000
H	-4.846275000	2.258172000	-0.073319000
H	-5.271994000	-0.413056000	0.221777000
H	-3.671600000	-2.522083000	0.385772000
H	-2.156896000	-4.655129000	0.448672000
H	0.530393000	-5.086261000	0.422512000
N	-0.071822000	-0.183879000	2.390553000
O	-0.198248000	-0.843770000	3.385365000
O	0.145419000	1.069069000	2.375590000

VPorph-ONO (m3)

C	2.627183000	-1.474216000	0.043048000
C	4.042332000	-1.275510000	-0.046856000
C	4.251999000	0.071346000	-0.180463000
C	2.964974000	0.700683000	-0.172796000
N	1.982111000	-0.258422000	-0.047590000
C	2.007153000	-2.715495000	0.207652000
C	2.753436000	2.078447000	-0.265294000
C	1.521371000	2.730023000	-0.248070000
C	1.320321000	4.147447000	-0.413042000
C	-0.027509000	4.360310000	-0.383549000
C	-0.648698000	3.072125000	-0.202422000
N	0.312132000	2.100422000	-0.105183000
C	-2.020884000	2.830661000	-0.173273000
C	-2.642400000	1.582638000	-0.084246000
C	-4.059066000	1.378882000	-0.045039000
C	-4.269720000	0.030597000	0.070363000
C	-2.981690000	-0.595869000	0.104187000
N	-1.997237000	0.365308000	-0.004726000
C	-2.768965000	-1.968671000	0.247016000
C	-1.534545000	-2.612655000	0.310874000
C	-1.335354000	-4.037624000	0.385213000
C	0.013218000	-4.248120000	0.378829000
C	0.636900000	-2.950832000	0.300652000
N	-0.323319000	-1.972114000	0.281240000
H	4.782793000	-2.073124000	-0.003490000
H	5.198870000	0.602152000	-0.270592000
H	2.664222000	-3.586989000	0.245166000
H	3.643186000	2.702427000	-0.373684000
H	2.117992000	4.876694000	-0.547982000
H	-0.567428000	5.300392000	-0.490000000
V	-0.001306000	0.086166000	0.330924000
H	-2.679841000	3.698770000	-0.242334000
H	-4.799763000	2.176105000	-0.091858000
H	-5.217429000	-0.501972000	0.138448000
H	-3.660187000	-2.597695000	0.298910000
H	-2.135856000	-4.775538000	0.419879000
H	0.552108000	-5.194325000	0.407215000
O	0.052429000	0.339256000	2.202334000
N	-0.517315000	-0.581251000	3.019876000
O	-0.285581000	-0.377370000	4.171565000

CrPorph-Cl (m4)

C	2.672734000	-1.445013000	0.077715000
C	4.091413000	-1.221229000	0.020855000
C	4.274693000	0.122823000	-0.135741000
C	2.967200000	0.720743000	-0.177251000
N	2.007593000	-0.250652000	-0.041189000
C	2.064961000	-2.688341000	0.242794000
C	2.717637000	2.083910000	-0.322014000
C	1.464878000	2.694571000	-0.337750000
C	1.239098000	4.107446000	-0.476165000
C	-0.112646000	4.298133000	-0.441092000
C	-0.712667000	3.001490000	-0.277845000
N	0.264866000	2.041055000	-0.211319000
C	-2.081836000	2.758145000	-0.193808000
C	-2.686072000	1.515224000	-0.013955000
C	-4.105122000	1.292315000	0.037649000
C	-4.289988000	-0.051672000	0.191225000
C	-2.983031000	-0.649906000	0.236431000
N	-2.020898000	0.322922000	0.126442000
C	-2.736170000	-2.015175000	0.362411000
C	-1.483358000	-2.624756000	0.390221000
C	-1.256364000	-4.038109000	0.517340000
C	0.095808000	-4.226690000	0.485855000
C	0.695465000	-2.928296000	0.335667000
N	-0.283027000	-1.966619000	0.294008000
H	4.844592000	-2.003652000	0.102414000
H	5.209532000	0.676730000	-0.210609000
H	2.724581000	-3.556101000	0.306418000
H	3.586502000	2.737678000	-0.421972000
H	2.026562000	4.852310000	-0.581745000
H	-0.668992000	5.231775000	-0.512345000
Cr	-0.020633000	0.008957000	-0.144444000
H	-2.744818000	3.621637000	-0.277637000
H	-4.857067000	2.076055000	-0.042673000
H	-5.225225000	-0.605100000	0.264538000
H	-3.606268000	-2.670552000	0.437721000
H	-2.043214000	-4.784426000	0.616951000
H	0.653161000	-5.159934000	0.554469000
Cl	-0.220289000	-0.296182000	-2.387070000

CrPorph-Cl-OH (m3)

C	2.703217000	-1.441287000	0.148868000
C	4.116790000	-1.225968000	0.008900000
C	4.300341000	0.113747000	-0.176631000
C	3.000559000	0.725290000	-0.148478000
N	2.041007000	-0.240029000	0.052471000
C	2.097685000	-2.679659000	0.346207000
C	2.755543000	2.088490000	-0.293676000
C	1.505382000	2.715313000	-0.270181000
C	1.273204000	4.127002000	-0.428524000
C	-0.077567000	4.311397000	-0.351662000

C	-0.665231000	3.011994000	-0.149530000
N	0.321472000	2.065369000	-0.098342000
C	-2.035131000	2.764237000	-0.048175000
C	-2.659878000	1.523380000	0.107988000
C	-4.083210000	1.315508000	0.209097000
C	-4.274088000	-0.028822000	0.349471000
C	-2.965843000	-0.634528000	0.336284000
N	-2.014121000	0.326088000	0.180487000
C	-2.701600000	-2.000483000	0.475406000
C	-1.447494000	-2.611531000	0.504587000
C	-1.227857000	-4.027358000	0.633121000
C	0.123342000	-4.223441000	0.605048000
C	0.726019000	-2.926235000	0.457058000
N	-0.241583000	-1.964695000	0.409914000
H	4.866505000	-2.014461000	0.048664000
H	5.232728000	0.657102000	-0.320669000
H	2.760419000	-3.545623000	0.400827000
H	3.627004000	2.729191000	-0.442994000
H	2.053602000	4.870913000	-0.580115000
H	-0.641301000	5.239801000	-0.428467000
Cr	0.065789000	0.047815000	0.166298000
H	-2.691034000	3.635881000	-0.103481000
H	-4.829473000	2.107825000	0.176039000
H	-5.209680000	-0.575040000	0.458296000
H	-3.566151000	-2.660112000	0.576942000
H	-2.019694000	-4.768793000	0.727040000
H	0.675893000	-5.159264000	0.670186000
Cl	-0.236305000	-0.172932000	-2.170162000
O	-0.072911000	0.360334000	1.968069000
H	-0.454390000	-0.413943000	2.413790000

CrPorph-Cl-Cl (m3)

C	2.689735000	-1.456782000	0.031009000
C	4.110952000	-1.238959000	-0.037962000
C	4.293310000	0.105643000	-0.186133000
C	2.981339000	0.699313000	-0.210924000
N	2.028120000	-0.268800000	-0.079992000
C	2.069718000	-2.701012000	0.194443000
C	2.717128000	2.067090000	-0.339881000
C	1.464179000	2.691055000	-0.357732000
C	1.243027000	4.109400000	-0.461932000
C	-0.108458000	4.298046000	-0.421086000
C	-0.703174000	2.992927000	-0.290854000
N	0.270676000	2.037041000	-0.260971000
C	-2.075164000	2.736477000	-0.193947000
C	-2.695236000	1.492271000	-0.030391000
C	-4.116477000	1.274480000	0.038616000
C	-4.298819000	-0.070087000	0.187127000
C	-2.986808000	-0.663711000	0.212055000
N	-2.033649000	0.304352000	0.080874000
C	-2.722528000	-2.031472000	0.341242000
C	-1.469588000	-2.655427000	0.358937000
C	-1.248490000	-4.073785000	0.463114000
C	0.102968000	-4.262536000	0.421919000
C	0.697727000	-2.957455000	0.291548000
N	-0.276032000	-2.001500000	0.261875000
H	4.862341000	-2.024066000	0.026276000
H	5.225839000	0.661123000	-0.270366000
H	2.731356000	-3.568864000	0.247409000
H	3.586297000	2.723687000	-0.425156000
H	2.031045000	4.855363000	-0.549161000
H	-0.667515000	5.231066000	-0.467173000
Cr	-0.002572000	0.017760000	0.000472000
H	-2.736777000	3.604339000	-0.247099000
H	-4.867872000	2.059566000	-0.025816000
H	-5.231340000	-0.625562000	0.271498000
H	-3.591662000	-2.688087000	0.426741000
H	-2.036538000	-4.819695000	0.550540000
H	0.661975000	-5.195593000	0.467868000
Cl	-0.199449000	-0.317384000	-2.324251000
Cl	0.192332000	0.353283000	2.325224000

CrPorph-Cl-F (m3)

C	2.695106000	-1.454165000	0.039298000
C	4.115675000	-1.240452000	-0.048766000
C	4.303287000	0.104693000	-0.184423000
C	2.995103000	0.706115000	-0.180923000
N	2.037832000	-0.256993000	-0.042512000
C	2.076908000	-2.700091000	0.181629000
C	2.733225000	2.074600000	-0.308876000
C	1.479810000	2.695832000	-0.318564000
C	1.252712000	4.109063000	-0.462860000
C	-0.098956000	4.295265000	-0.424821000
C	-0.692650000	2.994896000	-0.256040000
N	0.287215000	2.040243000	-0.193800000
C	-2.063843000	2.741099000	-0.172036000
C	-2.687785000	1.495899000	-0.012032000
C	-4.109678000	1.277584000	0.051929000
C	-4.290019000	-0.067884000	0.198849000
C	-2.976406000	-0.659831000	0.223697000
N	-2.026840000	0.311754000	0.095906000
C	-2.713139000	-2.028740000	0.344413000
C	-1.461438000	-2.657247000	0.348334000
C	-1.242852000	-4.077089000	0.450645000
C	0.108702000	-4.267930000	0.402387000
C	0.703030000	-2.961685000	0.271817000
N	-0.267781000	-2.008861000	0.242113000
H	4.864236000	-2.029983000	-0.008446000
H	5.237654000	0.655213000	-0.280376000
H	2.740932000	-3.566754000	0.223303000
H	3.601100000	2.729744000	-0.414238000
H	2.037859000	4.854246000	-0.577814000
H	-0.659584000	5.225361000	-0.501808000
Cr	0.017112000	0.035668000	0.046492000
H	-2.723204000	3.609530000	-0.240391000
H	-4.862440000	2.061414000	-0.012789000
H	-5.222216000	-0.624441000	0.280907000
H	-3.583382000	-2.683263000	0.435270000
H	-2.031011000	-4.822260000	0.544292000
H	0.666415000	-5.201872000	0.448066000
Cl	-0.219553000	-0.311051000	-2.296298000
F	0.049511000	0.149207000	1.880570000

CrPorph-Cl-N3 (m3)

C	2.641631000	-1.384506000	0.238791000
C	4.062720000	-1.166645000	0.198315000

C	4.248654000	0.171593000	-0.000041000
C	2.939197000	0.762813000	-0.077509000
N	1.981939000	-0.199865000	0.066172000
C	2.021724000	-2.622337000	0.443010000
C	2.676434000	2.124489000	-0.260685000
C	1.423319000	2.739567000	-0.320236000
C	1.200761000	4.148539000	-0.500523000
C	-0.150973000	4.337996000	-0.492484000
C	-0.750664000	3.043949000	-0.306398000
N	0.224169000	2.087631000	-0.205082000
C	-2.123651000	2.795941000	-0.234529000
C	-2.744520000	1.555983000	-0.049071000
C	-4.164867000	1.334641000	0.007699000
C	-4.344627000	-0.006432000	0.189393000
C	-3.032863000	-0.596788000	0.244504000
N	-2.079817000	0.373830000	0.100220000
C	-2.773676000	-1.959148000	0.419931000
C	-1.522729000	-2.582805000	0.480775000
C	-1.302495000	-3.991525000	0.677482000
C	0.050144000	-4.178556000	0.687803000
C	0.646275000	-2.882670000	0.492402000
N	-0.326681000	-1.935339000	0.369527000
H	4.812459000	-1.947471000	0.312538000
H	5.183066000	0.724028000	-0.083501000
H	2.684899000	-3.481328000	0.568879000
H	3.544418000	2.780176000	-0.360641000
H	1.989774000	4.889646000	-0.615020000
H	-0.707978000	5.267319000	-0.599187000
Cr	-0.052703000	0.091777000	0.038174000
H	-2.782072000	3.661813000	-0.333628000
H	-4.918019000	2.115238000	-0.084187000
H	-5.276691000	-0.561847000	0.279719000
H	-3.645631000	-2.609426000	0.521936000
H	-2.091421000	-4.732346000	0.794782000
H	0.608110000	-5.104648000	0.815402000
Cl	-0.164241000	-0.323754000	-2.257462000
N	0.021354000	0.234075000	2.098070000
N	0.762787000	-0.471646000	2.721728000
N	1.468537000	-1.140453000	3.356830000

CrPorph-Cl-SCN (m3)

C	2.759873000	-1.389242000	0.010557000
C	4.173470000	-1.191134000	-0.159739000
C	4.355327000	0.137865000	-0.414770000
C	3.051423000	0.744290000	-0.394296000
N	2.098409000	-0.201247000	-0.134361000
C	2.145961000	-2.617209000	0.279228000
C	2.790641000	2.103898000	-0.587664000
C	1.544531000	2.736888000	-0.533634000
C	1.320803000	4.146188000	-0.704901000
C	-0.021010000	4.351485000	-0.553903000
C	-0.609396000	3.064976000	-0.295699000
N	0.359845000	2.100081000	-0.286687000
C	-1.972991000	2.828689000	-0.092509000
C	-2.590634000	1.597296000	0.141559000
C	-4.003728000	1.399176000	0.318367000
C	-4.192004000	0.059152000	0.499036000
C	-2.891657000	-0.552164000	0.435667000
N	-1.935506000	0.398918000	0.221346000
C	-2.635081000	-1.918442000	0.592072000
C	-1.385470000	-2.548584000	0.563460000
C	-1.159470000	-3.952951000	0.769992000
C	0.190136000	-4.147329000	0.696348000
C	0.779521000	-2.860450000	0.439958000
N	-0.197289000	-1.904887000	0.367647000
H	4.921384000	-1.978852000	-0.089026000
H	5.284308000	0.675233000	-0.597470000
H	2.808112000	-3.481682000	0.365648000
H	3.653538000	2.742399000	-0.790111000
H	2.101683000	4.876631000	-0.908405000
H	-0.577231000	5.285564000	-0.609874000
Cr	0.071266000	0.086599000	-0.034290000
H	-2.628705000	3.701561000	-0.129385000
H	-4.746640000	2.194689000	0.301708000
H	-5.122097000	-0.481600000	0.665019000
H	-3.501325000	-2.560973000	0.766022000
H	-1.943215000	-4.686109000	0.951128000
H	0.750897000	-5.074240000	0.803490000
Cl	-0.225623000	-0.265042000	-2.306896000
S	0.412190000	0.496632000	2.463540000
C	-0.818577000	-0.417148000	3.084368000
N	-1.690998000	-1.061657000	3.547146000

CrPorph-Cl-ONO2 (m3)

C	2.620041000	-1.523310000	0.091585000
C	4.046679000	-1.351348000	0.017475000
C	4.270457000	-0.013601000	-0.141594000
C	2.978306000	0.620377000	-0.161760000
N	1.995829000	-0.316099000	-0.020763000
C	1.959427000	-2.749217000	0.241966000
C	2.757260000	1.996166000	-0.293255000
C	1.524714000	2.661133000	-0.298917000
C	1.352194000	4.087006000	-0.386891000
C	0.008419000	4.322500000	-0.321614000
C	-0.628483000	3.038196000	-0.199701000
N	0.310317000	2.048298000	-0.193919000
C	-2.009240000	2.827552000	-0.098703000
C	-2.669665000	1.599472000	0.015745000
C	-4.097252000	1.424241000	0.057846000
C	-4.323559000	0.079273000	0.126854000
C	-3.032814000	-0.556718000	0.139207000
N	-2.048164000	0.385470000	0.075600000
C	-2.813900000	-1.936895000	0.209481000
C	-1.581265000	-2.597480000	0.255932000
C	-1.405789000	-4.023012000	0.337798000
C	-0.059843000	-4.251266000	0.366915000
C	0.577928000	-2.962942000	0.293268000
N	-0.365724000	-1.977740000	0.238151000
H	4.773354000	-2.159408000	0.080572000
H	5.219440000	0.511361000	-0.236865000
H	2.592126000	-3.637684000	0.303681000
H	3.646764000	2.623689000	-0.387031000
H	2.164333000	4.806057000	-0.478499000
H	-0.518226000	5.274844000	-0.350097000
Cr	-0.037379000	0.027329000	0.017749000
H	-2.640889000	3.718582000	-0.122932000

H	-4.822539000	2.235534000	0.028976000
H	-5.274340000	-0.449269000	0.169444000
H	-3.704358000	-2.569660000	0.233416000
H	-2.218390000	-4.746842000	0.367311000
H	0.468803000	-5.201186000	0.424053000
Cl	-0.165077000	-0.271603000	-2.306217000
O	0.219599000	0.401986000	1.980810000
N	-0.465109000	-0.101809000	2.972985000
O	-1.409008000	-0.846269000	2.742523000
O	-0.100936000	0.222573000	4.094378000

CrPorph-Cl-Me (m3)

C	2.693865000	-1.453732000	0.048181000
C	4.114537000	-1.242868000	-0.034425000
C	4.308812000	0.100764000	-0.174218000
C	3.005060000	0.710289000	-0.179040000
N	2.039059000	-0.248044000	-0.038585000
C	2.079946000	-2.696659000	0.197503000
C	2.752463000	2.077184000	-0.308487000
C	1.500886000	2.691004000	-0.314429000
C	1.272897000	4.103804000	-0.445705000
C	-0.077816000	4.294333000	-0.399325000
C	-0.679212000	2.998308000	-0.238747000
N	0.301423000	2.032510000	-0.187930000
C	-2.048230000	2.760029000	-0.145597000
C	-2.671937000	1.517987000	0.012736000
C	-4.093016000	1.298747000	0.091213000
C	-4.273605000	-0.046865000	0.231709000
C	-2.962259000	-0.644196000	0.240502000
N	-2.009043000	0.331351000	0.111417000
C	-2.707282000	-2.011169000	0.360730000
C	-1.459807000	-2.642148000	0.362231000
C	-1.242916000	-4.061964000	0.478401000
C	0.107542000	-4.257008000	0.431856000
C	0.707736000	-2.954029000	0.287096000
N	-0.261596000	-1.997598000	0.251933000
H	4.860503000	-2.034773000	0.012979000
H	5.246866000	0.645974000	-0.267332000
H	2.744317000	-3.562527000	0.244626000
H	3.620253000	2.731762000	-0.413936000
H	2.059117000	4.848714000	-0.557390000
H	-0.634370000	5.228002000	-0.464934000
Cr	0.021857000	0.041814000	-0.032472000
H	-2.701220000	3.633486000	-0.204849000
H	-4.846112000	2.083576000	0.039439000
H	-5.206529000	-0.601582000	0.320921000
H	-3.580420000	-2.660986000	0.455724000
H	-2.032655000	-4.804643000	0.580782000
H	0.661996000	-5.192624000	0.488063000
Cl	-0.260692000	-0.403059000	-2.336825000
C	-0.009690000	0.015532000	2.129303000
H	-0.916028000	-0.488831000	2.485943000
H	0.897061000	-0.499939000	2.472521000
H	-0.004947000	1.079090000	2.408863000

CrPorph-Cl-NO2 (m3)

C	2.619695000	-1.497732000	0.083526000
C	4.047933000	-1.334547000	0.028643000
C	4.284101000	-0.001609000	-0.149503000
C	2.998325000	0.641689000	-0.197333000
N	2.004347000	-0.285384000	-0.056217000
C	1.958045000	-2.717744000	0.248626000
C	2.788270000	2.016324000	-0.333829000
C	1.556207000	2.674491000	-0.327242000
C	1.377226000	4.099257000	-0.400395000
C	0.034194000	4.330711000	-0.319422000
C	-0.601118000	3.045776000	-0.204491000
N	0.341283000	2.053929000	-0.212290000
C	-1.978706000	2.839390000	-0.101734000
C	-2.642587000	1.613369000	0.002715000
C	-4.070016000	1.449841000	0.072541000
C	-4.307255000	0.107754000	0.152453000
C	-3.022906000	-0.540163000	0.140309000
N	-2.028048000	0.394753000	0.047333000
C	-2.816526000	-1.918186000	0.234666000
C	-1.583938000	-2.576717000	0.270896000
C	-1.402312000	-3.997984000	0.398704000
C	-0.055002000	-4.219804000	0.415847000
C	0.577162000	-2.933094000	0.292173000
N	-0.369970000	-1.953117000	0.210518000
H	4.768302000	-2.145940000	0.117686000
H	5.238324000	0.515483000	-0.235803000
H	2.590870000	-3.603794000	0.337255000
H	3.678106000	2.642525000	-0.428917000
H	2.186843000	4.821261000	-0.491938000
H	-0.494629000	5.282286000	-0.334061000
Cr	-0.019242000	0.044482000	-0.106081000
H	-2.607057000	3.732838000	-0.113823000
H	-4.788596000	2.267589000	0.057525000
H	-5.261419000	-0.412057000	0.221141000
H	-3.709089000	-2.544547000	0.298352000
H	-2.211517000	-4.722912000	0.468631000
H	0.477504000	-5.165654000	0.500845000
Cl	-0.161651000	-0.285295000	-2.411654000
N	0.084901000	0.218658000	2.055659000
O	0.742957000	-0.601072000	2.676755000
O	-0.524859000	1.139505000	2.578967000

CrPorph-Cl-ONO (m3)

C	2.636332000	-1.505307000	0.093828000
C	4.062987000	-1.338628000	0.003189000
C	4.290005000	-0.003894000	-0.176970000
C	3.000575000	0.635878000	-0.192083000
N	2.017045000	-0.296810000	-0.026464000
C	1.974027000	-2.727662000	0.262551000
C	2.782494000	2.009748000	-0.336463000
C	1.550115000	2.672097000	-0.321574000
C	1.371636000	4.097509000	-0.405090000
C	0.028749000	4.330500000	-0.314653000
C	-0.604779000	3.045114000	-0.183860000
N	0.336835000	2.057930000	-0.193649000
C	-1.983474000	2.832198000	-0.066930000
C	-2.642362000	1.605676000	0.052887000
C	-4.069770000	1.435159000	0.111936000
C	-4.303456000	0.091803000	0.176596000
C	-3.017995000	-0.554228000	0.171670000

N	-2.025029000	0.385116000	0.102046000
C	-2.805941000	-1.932833000	0.240224000
C	-1.571724000	-2.586116000	0.295225000
C	-1.385863000	-4.009015000	0.393635000
C	-0.038906000	-4.229319000	0.420633000
C	0.594696000	-2.940450000	0.328763000
N	-0.356862000	-1.957802000	0.268468000
H	4.788007000	-2.147986000	0.070357000
H	5.240391000	0.515624000	-0.288068000
H	2.605515000	-3.616681000	0.326609000
H	3.670621000	2.635352000	-0.451442000
H	2.180214000	4.819014000	-0.509165000
H	-0.499449000	5.282393000	-0.331274000
Cr	-0.015565000	0.037679000	-0.016195000
H	-2.616294000	3.722447000	-0.082714000
H	-4.791288000	2.250316000	0.097007000
H	-5.257140000	-0.430635000	0.229940000
H	-3.695958000	-2.565162000	0.269438000
H	-2.194029000	-4.737285000	0.435638000
H	0.493920000	-5.176435000	0.487104000
Cl	-0.128355000	-0.268486000	-2.333404000
O	0.183336000	0.348764000	1.954173000
N	-0.703044000	-0.230873000	2.698190000
O	-0.597168000	-0.040160000	3.882331000

CrPorph (m5)

C	2.697139000	-1.441867000	-0.093238000
C	4.116556000	-1.222686000	-0.196532000
C	4.298979000	0.120503000	-0.369439000
C	2.990376000	0.722921000	-0.375583000
N	2.034377000	-0.246020000	-0.203527000
C	2.082919000	-2.683604000	0.088392000
C	2.731857000	2.085973000	-0.538311000
C	1.477031000	2.700251000	-0.562616000
C	1.247889000	4.113550000	-0.712518000
C	-0.104859000	4.302939000	-0.670366000
C	-0.703999000	3.004903000	-0.495720000
N	0.276937000	2.046344000	-0.437092000
C	-2.075641000	2.759913000	-0.394012000
C	-2.689861000	1.518175000	-0.212383000
C	-4.109281000	1.298991000	-0.109128000
C	-4.291700000	-0.044191000	0.063846000
C	-2.983098000	-0.646613000	0.069960000
N	-2.027099000	0.322328000	-0.102097000
C	-2.724579000	-2.009664000	0.232693000
C	-1.469753000	-2.623941000	0.256998000
C	-1.240609000	-4.037234000	0.406958000
C	0.112135000	-4.226633000	0.364718000
C	0.711277000	-2.928593000	0.190106000
N	-0.269658000	-1.970035000	0.131475000
H	4.872180000	-2.005681000	-0.141540000
H	5.234603000	0.666545000	-0.486694000
H	2.742539000	-3.552253000	0.151438000
H	3.599343000	2.739687000	-0.656245000
H	2.031261000	4.861504000	-0.829932000
H	-0.659770000	5.237612000	-0.745855000
Cr	0.003639000	0.038154000	-0.152808000
H	-2.735262000	3.628560000	-0.457073000
H	-4.864906000	2.081982000	-0.164148000
H	-5.227323000	-0.590230000	0.181125000
H	-3.592064000	-2.663376000	0.350645000
H	-2.023979000	-4.785184000	0.524413000
H	0.667045000	-5.161310000	0.440179000

CrPorph-OH (m4)

C	2.683454000	-1.424815000	0.052882000
C	4.105174000	-1.207441000	0.005492000
C	4.295533000	0.138653000	-0.126222000
C	2.989296000	0.743667000	-0.163344000
N	2.027648000	-0.226164000	-0.051823000
C	2.065801000	-2.667535000	0.200623000
C	2.742886000	2.111281000	-0.288230000
C	1.491053000	2.727568000	-0.311393000
C	1.268687000	4.144705000	-0.434402000
C	-0.083349000	4.335357000	-0.421591000
C	-0.686410000	3.034190000	-0.288289000
N	0.289771000	2.075531000	-0.217965000
C	-2.058341000	2.789169000	-0.231953000
C	-2.672548000	1.545574000	-0.078813000
C	-4.093474000	1.330263000	-0.018154000
C	-4.285397000	-0.013292000	0.135346000
C	-2.980982000	-0.620004000	0.170100000
N	-2.015962000	0.347335000	0.044591000
C	-2.736235000	-1.986435000	0.309113000
C	-1.484503000	-2.601600000	0.344163000
C	-1.259126000	-4.016844000	0.474742000
C	0.092787000	-4.206978000	0.440699000
C	0.694299000	-2.907590000	0.289197000
N	-0.283953000	-1.947529000	0.245502000
H	4.855540000	-1.994010000	0.074886000
H	5.234037000	0.688216000	-0.189327000
H	2.722207000	-3.538470000	0.259204000
H	3.614832000	2.764232000	-0.367521000
H	2.057336000	4.891530000	-0.517358000
H	-0.637560000	5.270622000	-0.491355000
Cr	-0.000836000	0.035623000	-0.170522000
H	-2.718099000	3.656279000	-0.307276000
H	-4.842640000	2.118025000	-0.086665000
H	-5.224198000	-0.559451000	0.219507000
H	-3.608629000	-2.638079000	0.393356000
H	-2.045861000	-4.763208000	0.576762000
H	0.648263000	-5.141517000	0.510011000
O	-0.077478000	-0.186085000	-1.993861000
H	-0.948643000	-0.534917000	-2.229161000

CrPorph-F (m4)

C	2.668201000	-1.459165000	0.068638000
C	4.087551000	-1.234723000	0.016590000
C	4.271354000	0.110587000	-0.129654000
C	2.963618000	0.709486000	-0.168872000
N	2.005293000	-0.263981000	-0.045232000
C	2.058859000	-2.703666000	0.226245000
C	2.713473000	2.074634000	-0.301203000
C	1.460657000	2.687225000	-0.316252000
C	1.235339000	4.101703000	-0.444743000

C	-0.116666000	4.292250000	-0.413282000
C	-0.718048000	2.993880000	-0.263228000
N	0.259867000	2.034677000	-0.201389000
C	-2.088490000	2.749901000	-0.187012000
C	-2.695789000	1.505104000	-0.025350000
C	-4.115156000	1.282236000	0.033005000
C	-4.299915000	-0.062978000	0.177465000
C	-2.992603000	-0.662958000	0.209641000
N	-2.032630000	0.310379000	0.096103000
C	-2.744283000	-2.028897000	0.336768000
C	-1.491410000	-2.640570000	0.363224000
C	-1.264452000	-4.053529000	0.501310000
C	0.087828000	-4.242938000	0.471358000
C	0.688500000	-2.945563000	0.312248000
N	-0.290581000	-1.985813000	0.259042000
H	4.841027000	-2.017521000	0.092539000
H	5.206771000	0.664231000	-0.199954000
H	2.718815000	-3.571031000	0.292769000
H	3.582866000	2.729061000	-0.392598000
H	2.022766000	4.847639000	-0.543560000
H	-0.672247000	5.226684000	-0.481123000
Cr	-0.021965000	0.001538000	-0.136188000
H	-2.750069000	3.615495000	-0.260291000
H	-4.867714000	2.066678000	-0.034871000
H	-5.235465000	-0.615521000	0.254367000
H	-3.614401000	-2.683508000	0.419268000
H	-2.051032000	-4.799314000	0.607403000
H	0.644125000	-5.176268000	0.547980000
F	-0.125338000	-0.190856000	-1.920365000

CrPorph-N3 (m4)

C	2.530639000	-1.485704000	-0.021851000
C	3.947506000	-1.289127000	-0.161687000
C	4.147610000	0.051313000	-0.330284000
C	2.852778000	0.675937000	-0.293772000
N	1.884496000	-0.277267000	-0.099152000
C	1.904870000	-2.719281000	0.154349000
C	2.621109000	2.043950000	-0.427894000
C	1.382358000	2.682317000	-0.376136000
C	1.178533000	4.099319000	-0.508876000
C	-0.165685000	4.318188000	-0.406028000
C	-0.784470000	3.034586000	-0.211413000
N	0.176202000	2.055539000	-0.187200000
C	-2.154774000	2.817021000	-0.077089000
C	-2.777913000	1.582854000	0.102021000
C	-4.197420000	1.385386000	0.214167000
C	-4.402110000	0.042139000	0.350636000
C	-3.106932000	-0.582353000	0.325843000
N	-2.132753000	0.374376000	0.184190000
C	-2.879136000	-1.953346000	0.430778000
C	-1.637642000	-2.587778000	0.411439000
C	-1.433616000	-4.005367000	0.530851000
C	-0.086875000	-4.220823000	0.457765000
C	0.534194000	-2.934894000	0.291348000
N	-0.427117000	-1.954056000	0.276661000
H	4.688181000	-2.086946000	-0.129673000
H	5.087172000	0.585257000	-0.466134000
H	2.549508000	-3.599890000	0.186676000
H	3.496113000	2.678592000	-0.582718000
H	1.974111000	4.827712000	-0.658947000
H	-0.705373000	5.262969000	-0.455647000
Cr	-0.138514000	0.031944000	-0.099542000
H	-2.802433000	3.694895000	-0.122019000
H	-4.936699000	2.184729000	0.185683000
H	-5.344328000	-0.493282000	0.459697000
H	-3.757565000	-2.593040000	0.537940000
H	-2.231093000	-4.736606000	0.654610000
H	0.453379000	-5.165141000	0.508620000
N	-0.289086000	-0.198780000	-2.045975000
N	0.384503000	-1.020134000	-2.613792000
N	1.002440000	-1.789172000	-3.218566000

CrPorph-SCN (m4)

C	2.643857000	-1.450647000	0.105703000
C	4.060722000	-1.228919000	0.012383000
C	4.241914000	0.113538000	-0.159149000
C	2.935392000	0.713954000	-0.168931000
N	1.977929000	-0.254535000	0.002241000
C	2.036733000	-2.695090000	0.263081000
C	2.683636000	2.075346000	-0.325944000
C	1.432513000	2.688941000	-0.316286000
C	1.207856000	4.100555000	-0.453104000
C	-0.142007000	4.297167000	-0.380239000
C	-0.743789000	3.004945000	-0.203054000
N	0.231358000	2.039062000	-0.161147000
C	-2.113554000	2.767155000	-0.119975000
C	-2.720836000	1.520583000	0.015927000
C	-4.138616000	1.294584000	0.067377000
C	-4.322293000	-0.053904000	0.179446000
C	-3.016038000	-0.653437000	0.202589000
N	-2.054306000	0.323261000	0.108891000
C	-2.768476000	-2.020036000	0.312032000
C	-1.516459000	-2.630895000	0.353152000
C	-1.290979000	-4.043416000	0.475616000
C	0.062189000	-4.232878000	0.468696000
C	0.666284000	-2.936130000	0.338541000
N	-0.312050000	-1.972349000	0.283047000
H	4.814034000	-2.012986000	0.073614000
H	5.175046000	0.664497000	-0.268199000
H	2.695890000	-3.563491000	0.320345000
H	3.550319000	2.726266000	-0.457275000
H	1.994947000	4.841838000	-0.582436000
H	-0.696288000	5.232810000	-0.438855000
Cr	-0.046328000	0.017853000	-0.062783000
H	-2.772681000	3.635576000	-0.176672000
H	-4.891249000	2.080123000	0.017895000
H	-5.256914000	-0.609327000	0.243505000
H	-3.638925000	-2.676204000	0.374109000
H	-2.079347000	-4.790325000	0.556296000
H	0.616952000	-5.167244000	0.541430000
S	-0.111820000	-0.388871000	-2.431272000
C	-0.527049000	1.114601000	-3.008310000
N	-0.821137000	2.158272000	-3.465295000

CrPorph-ONO2 (m4)

C	2.640767000	-1.487953000	0.049397000

C	4.058309000	-1.265041000	-0.028352000
C	4.241493000	0.082488000	-0.153778000
C	2.934829000	0.683568000	-0.158779000
N	1.977402000	-0.289580000	-0.024399000
C	2.031865000	-2.733499000	0.193245000
C	2.685037000	2.048059000	-0.290928000
C	1.432553000	2.659598000	-0.312480000
C	1.208440000	4.069755000	-0.477740000
C	-0.144036000	4.259472000	-0.475180000
C	-0.746437000	2.965631000	-0.302165000
N	0.230623000	2.008900000	-0.193974000
C	-2.117005000	2.721488000	-0.246446000
C	-2.719597000	1.480175000	-0.052890000
C	-4.135930000	1.259247000	0.041432000
C	-4.315622000	-0.077806000	0.254770000
C	-3.009851000	-0.676991000	0.282175000
N	-2.051537000	0.289636000	0.099116000
C	-2.761848000	-2.035488000	0.457605000
C	-1.511985000	-2.649134000	0.448274000
C	-1.285771000	-4.059258000	0.597360000
C	0.062721000	-4.258441000	0.505920000
C	0.663462000	-2.967866000	0.309582000
N	-0.312805000	-2.001507000	0.280960000
H	4.811024000	-2.050933000	0.015993000
H	5.175846000	0.636078000	-0.236307000
H	2.689061000	-3.604296000	0.234367000
H	3.554006000	2.701228000	-0.393519000
H	1.997285000	4.813008000	-0.584155000
H	-0.699300000	5.190775000	-0.578249000
Cr	-0.050887000	-0.025649000	-0.125874000
H	-2.779995000	3.582704000	-0.349917000
H	-4.889773000	2.040636000	-0.043168000
H	-5.248602000	-0.625290000	0.382048000
H	-3.629530000	-2.682924000	0.599925000
H	-2.071022000	-4.798199000	0.749885000
H	0.617078000	-5.193738000	0.570067000
O	0.079005000	-0.077037000	-2.055056000
N	-0.708653000	-0.770606000	-2.859603000
O	-1.627462000	-1.414266000	-2.384871000
O	-0.434277000	-0.696437000	-4.043723000

CrPorph-Me (m4)

C	2.667506000	-1.468143000	0.057665000
C	4.088285000	-1.247591000	-0.005256000
C	4.273910000	0.097215000	-0.155115000
C	2.965749000	0.698527000	-0.185485000
N	2.006005000	-0.272402000	-0.054061000
C	2.054297000	-2.711559000	0.219361000
C	2.715224000	2.064279000	-0.322506000
C	1.463037000	2.681282000	-0.333587000
C	1.239228000	4.097337000	-0.458196000
C	-0.112906000	4.288919000	-0.424828000
C	-0.715227000	2.989347000	-0.279478000
N	0.262128000	2.028839000	-0.221729000
C	-2.087213000	2.743985000	-0.209113000
C	-2.699734000	1.499379000	-0.054399000
C	-4.120359000	1.279708000	0.012364000
C	-4.306896000	-0.065726000	0.155392000
C	-2.999309000	-0.668085000	0.179185000
N	-2.038371000	0.302640000	0.054699000
C	-2.749201000	-2.034146000	0.315765000
C	-1.496040000	-2.647308000	0.354163000
C	-1.270489000	-4.060486000	0.504452000
C	0.081898000	-4.250583000	0.478001000
C	0.682891000	-2.953221000	0.309850000
N	-0.295107000	-1.993276000	0.246606000
H	4.841355000	-2.031496000	0.065449000
H	5.210554000	0.647917000	-0.234152000
H	2.713211000	-3.579987000	0.287056000
H	3.585553000	2.717324000	-0.418204000
H	2.026473000	4.843883000	-0.555522000
H	-0.666959000	5.224685000	-0.489243000
Cr	-0.022910000	-0.001071000	-0.138949000
H	-2.747200000	3.611769000	-0.275242000
H	-4.872670000	2.065379000	-0.046007000
H	-5.243662000	-0.615343000	0.240397000
H	-3.619651000	-2.687955000	0.404865000
H	-2.056927000	-4.805886000	0.615697000
H	0.636984000	-5.184112000	0.562988000
C	-0.132029000	-0.181192000	-2.172888000
H	0.862975000	-0.012533000	-2.616392000
H	-0.482560000	-1.190345000	-2.445003000
H	-0.836934000	0.564547000	-2.574993000

CrPorph-NO2 (m4)

C	2.617893000	-1.514102000	0.050667000
C	4.044378000	-1.341290000	0.006681000
C	4.278873000	-0.001652000	-0.120435000
C	2.995069000	0.644903000	-0.156633000
N	1.999641000	-0.293816000	-0.055270000
C	1.966564000	-2.740093000	0.176492000
C	2.793906000	2.019174000	-0.276642000
C	1.560206000	2.667916000	-0.310441000
C	1.377180000	4.090354000	-0.404984000
C	0.030963000	4.320189000	-0.370730000
C	-0.609984000	3.038612000	-0.255975000
N	0.338582000	2.046590000	-0.236903000
C	-1.986666000	2.841012000	-0.163010000
C	-2.639303000	1.616701000	-0.025756000
C	-4.064480000	1.447211000	0.059307000
C	-4.298733000	0.107037000	0.181752000
C	-3.016030000	-0.543356000	0.174205000
N	-2.022861000	0.393489000	0.043646000
C	-2.813700000	-1.918385000	0.283033000
C	-1.580965000	-2.570457000	0.278850000
C	-1.398375000	-3.991983000	0.392229000
C	-0.052291000	-4.222468000	0.361060000
C	0.588488000	-2.942066000	0.229715000
N	-0.360260000	-1.952994000	0.172482000
H	4.767860000	-2.153234000	0.066798000
H	5.234237000	0.517311000	-0.186231000
H	2.598149000	-3.628624000	0.239284000
H	3.685070000	2.646863000	-0.340237000
H	2.186890000	4.814783000	-0.481085000
H	-0.495846000	5.272510000	-0.415054000
Cr	-0.006208000	0.061715000	0.076889000

H	-2.615654000	3.732960000	-0.196411000
H	-4.787390000	2.261143000	0.023830000
H	-5.253375000	-0.409877000	0.270232000
H	-3.703654000	-2.543782000	0.378771000
H	-2.207901000	-4.714857000	0.483515000
H	0.474495000	-5.173908000	0.421570000
N	0.064708000	0.253840000	2.091611000
O	0.705607000	-0.578059000	2.717612000
O	-0.531857000	1.186854000	2.608821000

CrPorph-ONO (m4)

C	2.630599000	-1.516128000	0.044240000
C	4.055447000	-1.338010000	-0.021135000
C	4.282080000	0.001670000	-0.157953000
C	2.995294000	0.643046000	-0.178145000
N	2.004566000	-0.299557000	-0.062863000
C	1.985473000	-2.742800000	0.190802000
C	2.790223000	2.016756000	-0.293879000
C	1.556691000	2.665875000	-0.310453000
C	1.373714000	4.088478000	-0.404346000
C	0.028604000	4.319974000	-0.353188000
C	-0.611772000	3.038903000	-0.229549000
N	0.335778000	2.045703000	-0.220739000
C	-1.987471000	2.840217000	-0.124686000
C	-2.635122000	1.612862000	0.007878000
C	-4.059449000	1.435747000	0.084776000
C	-4.287206000	0.093679000	0.196532000
C	-3.001866000	-0.550655000	0.194151000
N	-2.012096000	0.392006000	0.074471000
C	-2.795561000	-1.924789000	0.299159000
C	-1.561876000	-2.573755000	0.301960000
C	-1.378578000	-3.995528000	0.404936000
C	-0.032484000	-4.225476000	0.378949000
C	0.608147000	-2.944375000	0.257777000
N	-0.340602000	-1.953932000	0.208117000
H	4.782612000	-2.146946000	0.033863000
H	5.234004000	0.524646000	-0.239591000
H	2.619079000	-3.629816000	0.252057000
H	3.680106000	2.644953000	-0.369249000
H	2.183217000	4.811724000	-0.492380000
H	-0.498286000	5.272443000	-0.391686000
Cr	0.001294000	0.063898000	0.142277000
H	-2.618708000	3.730672000	-0.151009000
H	-4.785890000	2.246460000	0.049777000
H	-5.239593000	-0.429026000	0.274048000
H	-3.684495000	-2.553491000	0.381277000
H	-2.188549000	-4.719319000	0.483666000
H	0.494964000	-5.176892000	0.432425000
O	0.154197000	0.322067000	2.027167000
N	-0.776609000	-0.263468000	2.778554000
O	-0.631748000	-0.041665000	3.950648000

MnPorph-Cl (m5)

C	2.676013000	-1.468364000	0.028709000
C	4.092276000	-1.242941000	-0.028180000
C	4.274716000	0.102103000	-0.171549000
C	2.969149000	0.699561000	-0.206213000
N	2.008883000	-0.273320000	-0.079161000
C	2.077927000	-2.714567000	0.181691000
C	2.727400000	2.062349000	-0.338555000
C	1.473720000	2.664185000	-0.348200000
C	1.246561000	4.077977000	-0.447901000
C	-0.104510000	4.267098000	-0.401411000
C	-0.703688000	2.968453000	-0.271807000
N	0.274334000	2.005526000	-0.241988000
C	-2.070467000	2.733042000	-0.174964000
C	-2.666729000	1.487425000	-0.008838000
C	-4.082765000	1.265437000	0.068919000
C	-4.266570000	-0.080136000	0.204221000
C	-2.961937000	-0.680415000	0.214496000
N	-2.000233000	0.292164000	0.096640000
C	-2.721559000	-2.045363000	0.323788000
C	-1.467687000	-2.646478000	0.338141000
C	-1.239580000	-4.058851000	0.450810000
C	0.112038000	-4.246610000	0.413602000
C	0.710368000	-2.948590000	0.274968000
N	-0.267931000	-1.985920000	0.244164000
H	4.843307000	-2.028122000	0.043392000
H	5.207193000	0.659957000	-0.243281000
H	2.738219000	-3.581817000	0.234091000
H	3.595776000	2.717514000	-0.426056000
H	2.035631000	4.823430000	-0.533533000
H	-0.664321000	5.200299000	-0.440823000
Mn	-0.010792000	-0.018871000	-0.212479000
H	-2.732041000	3.599349000	-0.227501000
H	-4.832381000	2.053692000	0.019021000
H	-5.199115000	-0.635869000	0.290166000
H	-3.590202000	-2.701418000	0.401404000
H	-2.028022000	-4.803884000	0.545103000
H	0.672895000	-5.178286000	0.470506000
Cl	-0.232916000	-0.338133000	-2.580389000

MnPorph-Cl-OH (m4)

C	2.693761000	-1.448737000	0.132823000
C	4.107397000	-1.231868000	0.007145000
C	4.284681000	0.095715000	-0.253862000
C	2.980375000	0.697114000	-0.272943000
N	2.029167000	-0.261119000	-0.036831000
C	2.100018000	-2.688153000	0.340342000
C	2.736590000	2.051583000	-0.465017000
C	1.488044000	2.659111000	-0.405874000
C	1.258740000	4.074506000	-0.501090000
C	-0.079156000	4.272637000	-0.323724000
C	-0.672491000	2.976863000	-0.135428000
N	0.299024000	2.013310000	-0.196302000
C	-2.030773000	2.746332000	0.043445000
C	-2.629636000	1.497708000	0.164265000
C	-4.045702000	1.275619000	0.226117000
C	-4.237301000	-0.075628000	0.248168000
C	-2.937636000	-0.684783000	0.220381000
N	-1.974259000	0.292133000	0.178912000
C	-2.703011000	-2.053659000	0.258218000
C	-1.449495000	-2.652378000	0.296590000
C	-1.214392000	-4.063754000	0.426647000
C	0.137264000	-4.237065000	0.487847000

C	0.732543000	-2.933168000	0.377527000
N	-0.252156000	-1.988359000	0.264558000
H	4.858251000	-2.014629000	0.101365000
H	5.213016000	0.641718000	-0.414750000
H	2.764577000	-3.547612000	0.441728000
H	3.599813000	2.696221000	-0.637459000
H	2.041310000	4.812842000	-0.667786000
H	-0.637320000	5.207580000	-0.320448000
Mn	0.028026000	0.014196000	0.070856000
H	-2.688838000	3.616580000	0.053645000
H	-4.790472000	2.069894000	0.234124000
H	-5.172478000	-0.632101000	0.285085000
H	-3.573763000	-2.710211000	0.291429000
H	-2.001362000	-4.814824000	0.473581000
H	0.703000000	-5.161960000	0.588821000
Cl	-0.179459000	-0.267208000	-2.236788000
O	0.158581000	0.203300000	1.883999000
H	-0.714713000	0.030794000	2.272155000

MnPorph-Cl-Cl (m4)

C	2.669395000	-1.464371000	0.020959000
C	4.083976000	-1.239278000	-0.055099000
C	4.266536000	0.105221000	-0.196957000
C	2.963108000	0.705933000	-0.212985000
N	2.005355000	-0.268088000	-0.082003000
C	2.076538000	-2.709410000	0.180574000
C	2.725714000	2.067455000	-0.338606000
C	1.472712000	2.664528000	-0.353491000
C	1.243317000	4.076197000	-0.462072000
C	-0.107645000	4.264450000	-0.422652000
C	-0.707812000	2.967848000	-0.288372000
N	0.272015000	2.007971000	-0.256376000
C	-2.073081000	2.737417000	-0.193392000
C	-2.665834000	1.492478000	-0.032895000
C	-4.080390000	1.267406000	0.044161000
C	-4.262787000	-0.076999000	0.187137000
C	-2.959410000	-0.677699000	0.202695000
N	-2.001700000	0.296281000	0.070073000
C	-2.721947000	-2.039193000	0.328636000
C	-1.469210000	-2.636448000	0.342560000
C	-1.239694000	-4.048246000	0.449469000
C	0.111068000	-4.236586000	0.409034000
C	0.711497000	-2.939979000	0.275704000
N	-0.268386000	-1.979908000	0.245040000
H	4.832340000	-2.028034000	0.000429000
H	5.197151000	0.663552000	-0.284238000
H	2.736961000	-3.576096000	0.230494000
H	3.593639000	2.722558000	-0.424871000
H	2.033753000	4.819290000	-0.552342000
H	-0.670277000	5.195160000	-0.472828000
Mn	0.001721000	0.013910000	-0.006145000
H	-2.733331000	3.604178000	-0.244211000
H	-4.828821000	2.056082000	-0.011511000
H	-5.193346000	-0.635282000	0.275339000
H	-3.590025000	-2.694055000	0.415227000
H	-2.029967000	-4.791564000	0.539497000
H	0.673417000	-5.167549000	0.458004000
Cl	-0.161675000	-0.298474000	-2.270294000
Cl	0.165774000	0.326183000	2.258414000

MnPorph-Cl-F (m4)

C	2.672726000	-1.435353000	0.181320000
C	4.085171000	-1.217208000	0.049836000
C	4.257248000	0.097550000	-0.271825000
C	2.951998000	0.693999000	-0.313172000
N	2.003757000	-0.257546000	-0.035102000
C	2.080243000	-2.670175000	0.413584000
C	2.704762000	2.041649000	-0.540160000
C	1.458651000	2.650880000	-0.462443000
C	1.229087000	4.064108000	-0.557454000
C	-0.102880000	4.267233000	-0.338738000
C	-0.696681000	2.976042000	-0.136238000
N	0.272642000	2.008969000	-0.212552000
C	-2.054820000	2.747816000	0.042844000
C	-2.654271000	1.496732000	0.115291000
C	-4.070470000	1.269834000	0.156960000
C	-4.259352000	-0.081318000	0.119526000
C	-2.957824000	-0.686397000	0.085005000
N	-1.997583000	0.293368000	0.081014000
C	-2.718647000	-2.054101000	0.112862000
C	-1.465184000	-2.644595000	0.212958000
C	-1.229591000	-4.052980000	0.354884000
C	0.117586000	-4.219952000	0.494694000
C	0.713211000	-2.916071000	0.412067000
N	-0.269786000	-1.973229000	0.252926000
H	4.838576000	-1.992584000	0.177339000
H	5.183235000	0.640065000	-0.455756000
H	2.743469000	-3.525812000	0.548511000
H	3.564452000	2.682362000	-0.741437000
H	2.008398000	4.799846000	-0.748471000
H	-0.657582000	5.204005000	-0.322295000
Mn	0.000562000	0.017497000	0.027617000
H	-2.710282000	3.619263000	0.074702000
H	-4.816542000	2.061959000	0.194285000
H	-5.193096000	-0.641193000	0.129379000
H	-3.586029000	-2.715725000	0.108154000
H	-2.014349000	-4.807771000	0.357276000
H	0.680834000	-5.142306000	0.627168000
Cl	-0.115788000	-0.274681000	-2.237771000
F	0.074796000	0.224655000	1.815322000

MnPorph-Cl-N3 (m4)

C	2.740721000	-1.358528000	0.236701000
C	4.149159000	-1.139061000	0.072181000
C	4.307416000	0.157581000	-0.323029000
C	2.998165000	0.744361000	-0.372387000
N	2.059828000	-0.197917000	-0.034305000
C	2.160050000	-2.581185000	0.548460000
C	2.738139000	2.082272000	-0.640731000
C	1.491741000	2.688068000	-0.538136000
C	1.251756000	4.098111000	-0.652912000
C	-0.070981000	4.300969000	-0.382066000
C	-0.650227000	3.012087000	-0.130900000
N	0.320040000	2.046158000	-0.227638000
C	-1.999764000	2.782326000	0.104549000
C	-2.594775000	1.531247000	0.213568000

C	-4.008040000	1.301935000	0.293951000
C	-4.195218000	-0.050357000	0.278609000
C	-2.894635000	-0.653758000	0.222110000
N	-1.934890000	0.327642000	0.182366000
C	-2.648788000	-2.019666000	0.279916000
C	-1.389156000	-2.597036000	0.381683000
C	-1.138027000	-3.993586000	0.589909000
C	0.212196000	-4.140548000	0.728233000
C	0.794971000	-2.838854000	0.573880000
N	-0.198573000	-1.911720000	0.377018000
H	4.910178000	-1.901479000	0.228917000
H	5.227130000	0.694914000	-0.548721000
H	2.831349000	-3.423156000	0.723446000
H	3.588376000	2.720011000	-0.886462000
H	2.019009000	4.832806000	-0.891217000
H	-0.628223000	5.236115000	-0.361623000
Mn	0.059206000	0.061930000	0.037127000
H	-2.656554000	3.652533000	0.143386000
H	-4.754946000	2.092733000	0.339694000
H	-5.127575000	-0.611010000	0.319385000
H	-3.511983000	-2.685890000	0.309907000
H	-1.914993000	-4.754697000	0.638310000
H	0.784541000	-5.049902000	0.903938000
Cl	-0.138917000	-0.282568000	-2.224517000
N	0.198995000	0.353723000	2.007183000
N	-0.594004000	-0.214697000	2.715872000
N	-1.331149000	-0.735968000	3.436903000

MnPorph-Cl-SCN (m4)

C	2.753161000	-1.327814000	0.205723000
C	4.154246000	-1.126252000	-0.019503000
C	4.304277000	0.144135000	-0.497377000
C	2.999115000	0.737439000	-0.529909000
N	2.067314000	-0.177114000	-0.103699000
C	2.177083000	-2.529777000	0.596451000
C	2.735764000	2.064618000	-0.843620000
C	1.501588000	2.683788000	-0.690715000
C	1.265041000	4.090773000	-0.829684000
C	-0.035895000	4.314232000	-0.478482000
C	-0.609981000	3.039725000	-0.160244000
N	0.345938000	2.060104000	-0.286453000
C	-1.948331000	2.826457000	0.143545000
C	-2.548178000	1.580258000	0.273806000
C	-3.959339000	1.360274000	0.390253000
C	-4.158072000	0.009613000	0.353017000
C	-2.865773000	-0.603139000	0.254767000
N	-1.897416000	0.371384000	0.210743000
C	-2.628786000	-1.969980000	0.307757000
C	-1.371099000	-2.548511000	0.418251000
C	-1.123324000	-3.932235000	0.697359000
C	0.225342000	-4.072341000	0.861330000
C	0.811955000	-2.783126000	0.641412000
N	-0.179188000	-1.864775000	0.389048000
H	4.917175000	-1.883172000	0.153406000
H	5.217920000	0.660473000	-0.787191000
H	2.849077000	-3.362397000	0.808803000
H	3.576160000	2.685457000	-1.156731000
H	2.020101000	4.810561000	-1.141057000
H	-0.583134000	5.254957000	-0.450847000
Mn	0.072737000	0.082863000	-0.029639000
H	-2.595376000	3.702681000	0.202838000
H	-4.698404000	2.155651000	0.468957000
H	-5.094075000	-0.543626000	0.407812000
H	-3.494983000	-2.631235000	0.353995000
H	-1.901343000	-4.689299000	0.778634000
H	0.794163000	-4.971541000	1.091654000
Cl	-0.184290000	-0.278579000	-2.264344000
S	0.389296000	0.512080000	2.399319000
C	-0.851273000	-0.390111000	3.025842000
N	-1.724520000	-1.025234000	3.494460000

MnPorph-Cl-ONO2 (m4)

C	2.642223000	-1.390141000	0.286082000
C	4.054056000	-1.174685000	0.151562000
C	4.222635000	0.113644000	-0.267044000
C	2.916314000	0.701323000	-0.352633000
N	1.968888000	-0.233032000	-0.015956000
C	2.049830000	-2.611205000	0.582648000
C	2.664985000	2.037792000	-0.635738000
C	1.422000000	2.650884000	-0.537524000
C	1.193838000	4.063572000	-0.634737000
C	-0.124500000	4.275394000	-0.347936000
C	-0.714437000	2.989123000	-0.113643000
N	0.246252000	2.015011000	-0.226093000
C	-2.067612000	2.765232000	0.106048000
C	-2.669526000	1.513996000	0.145381000
C	-4.084215000	1.283737000	0.165864000
C	-4.271605000	-0.064937000	0.056624000
C	-2.971485000	-0.668398000	0.013805000
N	-2.011294000	0.311858000	0.067508000
C	-2.729192000	-2.035240000	0.020065000
C	-1.479604000	-2.618437000	0.182904000
C	-1.243360000	-4.018587000	0.377373000
C	0.092654000	-4.169833000	0.617499000
C	0.685576000	-2.866862000	0.529163000
N	-0.292616000	-1.935300000	0.282733000
H	4.810445000	-1.934956000	0.338091000
H	5.147827000	0.645345000	-0.483516000
H	2.710954000	-3.453998000	0.789072000
H	3.520210000	2.670519000	-0.877044000
H	1.965838000	4.794448000	-0.869280000
H	-0.672581000	5.215404000	-0.310355000
Mn	-0.036193000	0.025189000	-0.017627000
H	-2.719253000	3.637821000	0.167910000
H	-4.832007000	2.072098000	0.233315000
H	-5.205841000	-0.623262000	0.028037000
H	-3.592755000	-2.700127000	-0.027202000
H	-2.020498000	-4.780535000	0.348150000
H	0.650922000	-5.083221000	0.815994000
Cl	-0.118505000	-0.280056000	-2.263053000
O	0.170606000	0.370467000	1.932305000
N	-0.539229000	-0.145764000	2.918342000
O	-1.482763000	-0.872519000	2.675404000
O	-0.162941000	0.176516000	4.032466000

MnPorph-Cl-Me (m4)

C	2.673270000	-1.448166000	0.064001000
C	4.088980000	-1.224979000	-0.016883000
C	4.272081000	0.120411000	-0.153598000
C	2.967416000	0.721235000	-0.161167000
N	2.010115000	-0.250674000	-0.028065000
C	2.075109000	-2.693511000	0.217690000
C	2.724560000	2.083715000	-0.290268000
C	1.469782000	2.680357000	-0.310759000
C	1.239854000	4.091490000	-0.438621000
C	-0.111362000	4.278276000	-0.405108000
C	-0.709198000	2.981109000	-0.255963000
N	0.270450000	2.021760000	-0.205270000
C	-2.076059000	2.748188000	-0.168557000
C	-2.671925000	1.502471000	-0.009070000
C	-4.087998000	1.278622000	0.070202000
C	-4.269249000	-0.065281000	0.219101000
C	-2.963479000	-0.664132000	0.233556000
N	-2.006250000	0.309046000	0.094456000
C	-2.723661000	-2.026023000	0.368180000
C	-1.470540000	-2.628158000	0.379766000
C	-1.240773000	-4.040707000	0.494785000
C	0.110407000	-4.227726000	0.450478000
C	0.707979000	-2.929026000	0.309188000
N	-0.272760000	-1.971872000	0.272906000
H	4.838292000	-2.013604000	0.029757000
H	5.203618000	0.676880000	-0.244692000
H	2.735449000	-3.560855000	0.265465000
H	3.591160000	2.740109000	-0.383716000
H	2.028523000	4.835379000	-0.538785000
H	-0.674084000	5.208168000	-0.470737000
Mn	0.000020000	0.017597000	-0.006761000
H	-2.736628000	3.614595000	-0.227242000
H	-4.837900000	2.066003000	0.013376000
H	-5.199985000	-0.622860000	0.311632000
H	-3.592282000	-2.679598000	0.462988000
H	-2.029000000	-4.785418000	0.592302000
H	0.672999000	-5.158517000	0.503045000
Cl	-0.157939000	-0.311329000	-2.378487000
C	0.135920000	0.185970000	2.050722000
H	-0.875479000	0.074324000	2.462010000
H	0.799522000	-0.615942000	2.397731000
H	0.555159000	1.176449000	2.269127000

MnPorph-Cl-NO2 (m4)

C	2.651716000	-1.430220000	0.021523000
C	4.066887000	-1.206626000	-0.022408000
C	4.255119000	0.143129000	-0.097567000
C	2.953675000	0.745945000	-0.116844000
N	1.988936000	-0.228177000	-0.037811000
C	2.055656000	-2.677501000	0.147817000
C	2.718266000	2.110036000	-0.220489000
C	1.463096000	2.698799000	-0.292659000
C	1.229764000	4.103971000	-0.452906000
C	-0.122613000	4.278567000	-0.511641000
C	-0.718968000	2.982194000	-0.371963000
N	0.263645000	2.030311000	-0.240577000
C	-2.085845000	2.746128000	-0.334602000
C	-2.677668000	1.510147000	-0.113797000
C	-4.090913000	1.291307000	-0.012940000
C	-4.271143000	-0.037081000	0.242704000
C	-2.968218000	-0.635038000	0.285790000
N	-2.008860000	0.325803000	0.076288000
C	-2.729883000	-1.988358000	0.482312000
C	-1.479002000	-2.590825000	0.459396000
C	-1.247983000	-3.999216000	0.595410000
C	0.097623000	-4.194555000	0.474736000
C	0.692428000	-2.904219000	0.277902000
N	-0.284646000	-1.939224000	0.276924000
H	4.813419000	-1.998347000	0.013580000
H	5.188408000	0.702133000	-0.140846000
H	2.714135000	-3.546755000	0.175615000
H	3.586331000	2.768656000	-0.271617000
H	2.017764000	4.852616000	-0.515055000
H	-0.687262000	5.202053000	-0.629152000
Mn	-0.019731000	0.031014000	-0.106307000
H	-2.747971000	3.604283000	-0.457604000
H	-4.840584000	2.073065000	-0.122127000
H	-5.200791000	-0.585405000	0.386284000
H	-3.595549000	-2.634191000	0.635839000
H	-2.032688000	-4.736591000	0.755100000
H	0.659465000	-5.126160000	0.518129000
Cl	-0.172906000	-0.295786000	-2.390032000
N	0.079283000	0.282885000	2.057181000
O	0.758902000	-0.512828000	2.667122000
O	-0.551582000	1.202245000	2.530448000

MnPorph-Cl-ONO (m4)

C	2.666219000	-1.393818000	0.232750000
C	4.077719000	-1.176015000	0.098814000
C	4.246750000	0.128729000	-0.263919000
C	2.940569000	0.719905000	-0.324559000
N	1.992834000	-0.225344000	-0.020595000
C	2.076000000	-2.621807000	0.502073000
C	2.691164000	2.061942000	-0.579066000
C	1.444443000	2.669532000	-0.503478000
C	1.213999000	4.080966000	-0.612601000
C	-0.115483000	4.286927000	-0.379962000
C	-0.706343000	2.998627000	-0.158010000
N	0.261636000	2.028626000	-0.231919000
C	-2.062878000	2.772865000	0.034658000
C	-2.660392000	1.521959000	0.113474000
C	-4.074778000	1.294160000	0.160566000
C	-4.263132000	-0.057781000	0.132702000
C	-2.962873000	-0.662435000	0.101584000
N	-2.000569000	0.317275000	0.087488000
C	-2.723408000	-2.028618000	0.155920000
C	-1.468719000	-2.612070000	0.271499000
C	-1.228810000	-4.013830000	0.452932000
C	0.118119000	-4.171775000	0.609293000
C	0.710056000	-2.870494000	0.494337000
N	-0.275528000	-1.933065000	0.301319000
H	4.833102000	-1.944726000	0.251746000
H	5.171559000	0.668192000	-0.462022000
H	2.739565000	-3.471458000	0.668684000
H	3.548656000	2.700134000	-0.796730000
H	1.991212000	4.814312000	-0.820487000

H	-0.669963000	5.223824000	-0.366767000
Mn	-0.012861000	0.036794000	-0.006059000
H	-2.718647000	3.644041000	0.064684000
H	-4.821170000	2.086035000	0.194809000
H	-5.196592000	-0.617841000	0.149053000
H	-3.589380000	-2.691849000	0.161349000
H	-2.010725000	-4.771261000	0.469991000
H	0.683380000	-5.087835000	0.772657000
Cl	-0.115949000	-0.279617000	-2.259784000
O	0.128092000	0.333939000	1.905645000
N	-0.757047000	-0.270191000	2.700269000
O	-0.580105000	-0.008072000	3.856362000

MnPorph (m6)

C	2.777674000	-1.410515000	-0.080789000
C	4.204268000	-1.191525000	-0.152345000
C	4.390289000	0.146659000	-0.370478000
C	3.076977000	0.748174000	-0.432107000
N	2.130889000	-0.223068000	-0.273310000
C	2.149053000	-2.638275000	0.191619000
C	2.806599000	2.118829000	-0.586626000
C	1.558228000	2.764877000	-0.546484000
C	1.337079000	4.186785000	-0.676501000
C	-0.011451000	4.391143000	-0.558322000
C	-0.615626000	3.094828000	-0.347415000
N	0.359520000	2.139195000	-0.353789000
C	-1.984495000	2.845671000	-0.142724000
C	-2.613228000	1.614512000	0.111375000
C	-4.031531000	1.415787000	0.310457000
C	-4.217609000	0.079175000	0.535243000
C	-2.912823000	-0.542117000	0.472615000
N	-1.971005000	0.413448000	0.218950000
C	-2.640818000	-1.908401000	0.652306000
C	-1.393790000	-2.556881000	0.592681000
C	-1.156188000	-3.957227000	0.856262000
C	0.191444000	-4.162788000	0.730037000
C	0.778838000	-2.888174000	0.382960000
N	-0.207183000	-1.946870000	0.301017000
H	4.964562000	-1.963259000	-0.032338000
H	5.332938000	0.686238000	-0.463110000
H	2.813357000	-3.498696000	0.309984000
H	3.676862000	2.766790000	-0.722371000
H	2.116375000	4.931748000	-0.836923000
H	-0.551613000	5.337365000	-0.593792000
Mn	0.036636000	0.045453000	-0.371804000
H	-2.640998000	3.719309000	-0.180449000
H	-4.785728000	2.202434000	0.282466000
H	-5.154252000	-0.444990000	0.725794000
H	-3.501568000	-2.543939000	0.877840000
H	-1.923228000	-4.684502000	1.122716000
H	0.742985000	-5.093361000	0.864747000

MnPorph-OH (m5)

C	2.713520000	-1.444304000	0.080038000
C	4.126743000	-1.239614000	-0.091390000
C	4.311938000	0.100972000	-0.269521000
C	3.010833000	0.711730000	-0.206343000
N	2.054530000	-0.247225000	-0.005906000
C	2.105359000	-2.676632000	0.304890000
C	2.760525000	2.077015000	-0.313100000
C	1.505627000	2.679150000	-0.260480000
C	1.274083000	4.090435000	-0.391057000
C	-0.075107000	4.281253000	-0.309491000
C	-0.672054000	2.986083000	-0.133543000
N	0.308859000	2.024192000	-0.103798000
C	-2.043612000	2.758743000	-0.054409000
C	-2.661267000	1.515481000	0.061185000
C	-4.082166000	1.300063000	0.130212000
C	-4.269198000	-0.044984000	0.268974000
C	-2.960559000	-0.644934000	0.289660000
N	-2.002027000	0.319544000	0.141320000
C	-2.703068000	-2.001770000	0.469011000
C	-1.442759000	-2.593220000	0.517904000
C	-1.212890000	-4.004847000	0.650614000
C	0.138313000	-4.194144000	0.608763000
C	0.738021000	-2.898168000	0.449603000
N	-0.241489000	-1.936262000	0.407342000
H	4.872759000	-2.032994000	-0.074580000
H	5.242374000	0.644230000	-0.429460000
H	2.755984000	-3.551926000	0.351360000
H	3.619986000	2.734789000	-0.455344000
H	2.058217000	4.832453000	-0.533730000
H	-0.636041000	5.212657000	-0.372610000
Mn	0.055140000	0.076656000	0.394585000
H	-2.692477000	3.635607000	-0.097037000
H	-4.831555000	2.088869000	0.079564000
H	-5.204274000	-0.596466000	0.358512000
H	-3.563806000	-2.665116000	0.573797000
H	-2.000925000	-4.750248000	0.747219000
H	0.697148000	-5.127427000	0.661721000
O	0.096688000	0.318920000	2.306681000
H	-0.602673000	-0.175971000	2.750371000

MnPorph-F (m5)

C	2.673256000	-1.461259000	0.021071000
C	4.088492000	-1.243002000	-0.085451000
C	4.273032000	0.101937000	-0.225475000
C	2.969680000	0.705785000	-0.207613000
N	2.008396000	-0.263482000	-0.065350000
C	2.075670000	-2.707404000	0.182137000
C	2.728982000	2.070520000	-0.325601000
C	1.475473000	2.673652000	-0.328168000
C	1.246615000	4.084239000	-0.464866000
C	-0.104637000	4.272868000	-0.421173000
C	-0.702128000	2.977054000	-0.257651000
N	0.276804000	2.015630000	-0.209526000
C	-2.070406000	2.741611000	-0.174453000
C	-2.670178000	1.494384000	-0.032025000
C	-4.087123000	1.269127000	0.021938000
C	-4.269591000	-0.076419000	0.159893000
C	-2.963343000	-0.673810000	0.193656000
N	-2.003407000	0.299214000	0.068210000
C	-2.721846000	-2.037071000	0.327757000
C	-1.468415000	-2.640629000	0.346076000
C	-1.241957000	-4.055286000	0.445193000
C	0.109287000	-4.243996000	0.403572000

C	0.708427000	-2.943950000	0.279157000
N	-0.269214000	-1.982221000	0.247742000
H	4.836985000	-2.033391000	-0.054533000
H	5.205131000	0.654358000	-0.335131000
H	2.736875000	-3.574653000	0.224825000
H	3.596963000	2.724169000	-0.428100000
H	2.033917000	4.827406000	-0.581004000
H	-0.665578000	5.203473000	-0.493696000
Mn	0.014170000	0.046199000	0.247016000
H	-2.730733000	3.608612000	-0.231991000
H	-4.838350000	2.054604000	-0.044970000
H	-5.202264000	-0.634114000	0.231203000
H	-3.591157000	-2.691491000	0.412839000
H	-2.030952000	-4.801302000	0.527274000
H	0.668936000	-5.177337000	0.443772000
F	0.099474000	0.229935000	2.144685000

MnPorph-N3 (m5)

C	2.625202000	-1.414196000	0.171058000
C	4.041281000	-1.189246000	0.117331000
C	4.225111000	0.155786000	-0.025895000
C	2.920650000	0.754007000	-0.067732000
N	1.958305000	-0.219057000	0.052346000
C	2.024726000	-2.658624000	0.329827000
C	2.679876000	2.113897000	-0.231060000
C	1.426530000	2.710456000	-0.317629000
C	1.200376000	4.114954000	-0.505147000
C	-0.151962000	4.300379000	-0.528764000
C	-0.754684000	3.009874000	-0.350420000
N	0.223426000	2.052006000	-0.237230000
C	-2.124274000	2.778359000	-0.282354000
C	-2.724881000	1.540112000	-0.081093000
C	-4.140647000	1.316917000	-0.009891000
C	-4.322545000	-0.019501000	0.201441000
C	-3.017719000	-0.615715000	0.258098000
N	-2.057951000	0.351124000	0.082547000
C	-2.774843000	-1.971846000	0.445943000
C	-1.522239000	-2.576312000	0.470418000
C	-1.298416000	-3.985790000	0.619945000
C	0.052070000	-4.180745000	0.576504000
C	0.655770000	-2.889665000	0.405270000
N	-0.320210000	-1.924990000	0.338138000
H	4.791460000	-1.975437000	0.185651000
H	5.158138000	0.711834000	-0.103521000
H	2.683670000	-3.525165000	0.404463000
H	3.549520000	2.768610000	-0.308526000
H	1.990707000	4.857194000	-0.604727000
H	-0.710135000	5.227442000	-0.650545000
Mn	-0.047620000	0.092911000	0.219435000
H	-2.783319000	3.641736000	-0.387339000
H	-4.892114000	2.097905000	-0.113845000
H	-5.255073000	-0.571731000	0.307585000
H	-3.643162000	-2.621723000	0.567188000
H	-2.089020000	-4.725156000	0.737079000
H	0.608897000	-5.113426000	0.652345000
N	0.035833000	0.397188000	2.316516000
N	0.711016000	-0.258057000	3.054381000
N	1.363930000	-0.887421000	3.787132000

MnPorph-SCN (m5)

C	2.715784000	-1.378468000	0.047067000
C	4.125550000	-1.180595000	-0.125009000
C	4.309180000	0.139820000	-0.418316000
C	3.012461000	0.753515000	-0.414401000
N	2.050397000	-0.189976000	-0.141732000
C	2.119255000	-2.596083000	0.350142000
C	2.778053000	2.107647000	-0.617706000
C	1.534472000	2.725408000	-0.555415000
C	1.309662000	4.129040000	-0.737138000
C	-0.029500000	4.342720000	-0.574654000
C	-0.627212000	3.068133000	-0.302307000
N	0.341992000	2.091468000	-0.294283000
C	-1.987613000	2.860220000	-0.105887000
C	-2.594249000	1.626959000	0.101046000
C	-4.007635000	1.423634000	0.233796000
C	-4.204361000	0.081319000	0.385496000
C	-2.910071000	-0.537168000	0.356762000
N	-1.940185000	0.420834000	0.179912000
C	-2.678021000	-1.897999000	0.511064000
C	-1.426845000	-2.502139000	0.523466000
C	-1.199174000	-3.896556000	0.762388000
C	0.152403000	-4.089162000	0.743265000
C	0.752780000	-2.814439000	0.478713000
N	-0.225595000	-1.855706000	0.348594000
H	4.871643000	-1.967790000	-0.030501000
H	5.238553000	0.672281000	-0.614298000
H	2.777736000	-3.456380000	0.479715000
H	3.641829000	2.740550000	-0.826996000
H	2.092732000	4.852326000	-0.958310000
H	-0.583109000	5.278148000	-0.638157000
Mn	0.068054000	0.132745000	0.130903000
H	-2.637191000	3.736086000	-0.142988000
H	-4.747945000	2.221453000	0.203423000
H	-5.140229000	-0.460651000	0.511999000
H	-3.546519000	-2.541812000	0.658663000
H	-1.988583000	-4.627557000	0.928594000
H	0.711488000	-5.012200000	0.888212000
S	0.386027000	0.595117000	2.728400000
C	-0.475766000	-0.686492000	3.315984000
N	-1.086097000	-1.599069000	3.751834000

MnPorph-ONO2 (m5)

C	2.593851000	-1.529714000	0.061433000
C	4.015439000	-1.349764000	-0.006269000
C	4.239620000	-0.013135000	-0.169096000
C	2.954958000	0.625761000	-0.197509000
N	1.962768000	-0.315712000	-0.065136000
C	1.959222000	-2.756649000	0.217550000
C	2.759065000	1.995981000	-0.325676000
C	1.525985000	2.638201000	-0.330132000
C	1.344839000	4.058847000	-0.417113000
C	0.001980000	4.293579000	-0.342289000
C	-0.639181000	3.016286000	-0.215960000
N	0.305260000	2.018068000	-0.224336000
C	-2.012254000	2.828617000	-0.104667000
C	-2.651652000	1.598477000	0.000723000

C	-4.074044000	1.416647000	0.044897000
C	-4.300818000	0.071436000	0.103362000
C	-3.017339000	-0.570163000	0.109935000
N	-2.025330000	0.377186000	0.046500000
C	-2.820618000	-1.943663000	0.178593000
C	-1.586649000	-2.581448000	0.217638000
C	-1.405439000	-4.001118000	0.314876000
C	-0.059765000	-4.230061000	0.345625000
C	0.583382000	-2.950441000	0.255608000
N	-0.363059000	-1.958411000	0.180651000
H	4.741080000	-2.158564000	0.063177000
H	5.188546000	0.513052000	-0.261157000
H	2.593122000	-3.641373000	0.294954000
H	3.648674000	2.621908000	-0.413060000
H	2.156924000	4.777538000	-0.514826000
H	-0.526390000	5.245366000	-0.368202000
Mn	-0.031294000	0.044166000	0.135031000
H	-2.641926000	3.719655000	-0.118315000
H	-4.798483000	2.229221000	0.021282000
H	-5.251494000	-0.458278000	0.140675000
H	-3.709958000	-2.575221000	0.212118000
H	-2.219565000	-4.723183000	0.353722000
H	0.469200000	-5.179369000	0.414249000
O	0.248770000	0.452348000	2.203630000
N	-0.467232000	-0.076696000	3.141250000
O	-1.402582000	-0.817189000	2.834953000
O	-0.173307000	0.193966000	4.301506000

MnPorph-Me (m5)

C	2.702695000	-1.469163000	-0.007158000
C	4.127031000	-1.249673000	-0.098283000
C	4.311241000	0.098938000	-0.235345000
C	2.999322000	0.701955000	-0.233330000
N	2.050393000	-0.272553000	-0.108342000
C	2.070973000	-2.708295000	0.174115000
C	2.723760000	2.074222000	-0.331929000
C	1.468632000	2.699561000	-0.326516000
C	1.240307000	4.119687000	-0.432499000
C	-0.113793000	4.309790000	-0.393270000
C	-0.717685000	3.006338000	-0.263344000
N	0.264778000	2.052341000	-0.229245000
C	-2.094555000	2.749258000	-0.195895000
C	-2.727757000	1.503737000	-0.070896000
C	-4.152511000	1.282475000	0.007291000
C	-4.336144000	-0.065476000	0.150463000
C	-3.023356000	-0.666397000	0.164160000
N	-2.075048000	0.306691000	0.013676000
C	-2.746562000	-2.032265000	0.322296000
C	-1.490023000	-2.655332000	0.359305000
C	-1.262094000	-4.076244000	0.455520000
C	0.092055000	-4.266531000	0.412249000
C	0.696046000	-2.962390000	0.290347000
N	-0.286647000	-2.009055000	0.270336000
H	4.885841000	-2.030550000	-0.055702000
H	5.250824000	0.642528000	-0.330206000
H	2.725888000	-3.582117000	0.220672000
H	3.589046000	2.736232000	-0.419643000
H	2.022355000	4.872419000	-0.527319000
H	-0.662648000	5.249307000	-0.450047000
Mn	0.006570000	0.058540000	0.325672000
H	-2.749711000	3.622907000	-0.241122000
H	-4.911657000	2.062732000	-0.041071000
H	-5.275457000	-0.609509000	0.245179000
H	-3.612182000	-2.692887000	0.417030000
H	-2.044880000	-4.830523000	0.529599000
H	0.640074000	-5.207680000	0.444405000
C	0.158175000	0.223046000	2.336658000
H	-0.848135000	0.059952000	2.749903000
H	0.859758000	-0.551690000	2.677449000
H	0.531049000	1.231186000	2.567771000

MnPorph-NO2 (m5)

C	2.658150000	-1.427971000	0.075538000
C	4.072323000	-1.195952000	0.021875000
C	4.252105000	0.149659000	-0.126579000
C	2.947665000	0.746093000	-0.166933000
N	1.988592000	-0.231451000	-0.034786000
C	2.068850000	-2.682521000	0.203943000
C	2.709045000	2.106240000	-0.340898000
C	1.457205000	2.709985000	-0.417225000
C	1.227413000	4.120573000	-0.564991000
C	-0.125527000	4.304243000	-0.546311000
C	-0.719773000	3.005454000	-0.385180000
N	0.260380000	2.049726000	-0.331158000
C	-2.084135000	2.761183000	-0.263096000
C	-2.672708000	1.516496000	-0.056962000
C	-4.084312000	1.292035000	0.062790000
C	-4.262721000	-0.050315000	0.240525000
C	-2.960353000	-0.652919000	0.228094000
N	-2.004519000	0.319562000	0.050707000
C	-2.720476000	-2.017486000	0.360988000
C	-1.472506000	-2.633191000	0.326748000
C	-1.243021000	-4.041047000	0.498953000
C	0.108269000	-4.230014000	0.448040000
C	0.701718000	-2.936241000	0.248227000
N	-0.278278000	-1.983794000	0.154710000
H	4.825836000	-1.979336000	0.086615000
H	5.184002000	0.706669000	-0.211103000
H	2.740964000	-3.538398000	0.286596000
H	3.582572000	2.755305000	-0.424028000
H	2.013459000	4.867879000	-0.661912000
H	-0.686993000	5.234200000	-0.623512000
Mn	-0.007649000	0.051189000	0.031323000
H	-2.753858000	3.620920000	-0.322095000
H	-4.837469000	2.076635000	0.009410000
H	-5.193084000	-0.602327000	0.365395000
H	-3.590275000	-2.660739000	0.505849000
H	-2.027595000	-4.781329000	0.647548000
H	0.669796000	-5.157818000	0.547510000
N	0.036680000	0.324206000	2.249469000
O	0.145853000	-0.657722000	2.988308000
O	-0.067616000	1.463940000	2.711057000

MnPorph-ONO (m5)

C	2.675855000	-1.423644000	0.069318000
C	4.089614000	-1.200083000	-0.028638000

C	4.268137000	0.139733000	-0.219404000
C	2.963074000	0.737079000	-0.236553000
N	2.004659000	-0.232421000	-0.065678000
C	2.083133000	-2.666444000	0.263848000
C	2.717992000	2.095871000	-0.397949000
C	1.462579000	2.693623000	-0.407389000
C	1.227915000	4.100366000	-0.561532000
C	-0.122753000	4.287831000	-0.494250000
C	-0.715644000	2.995043000	-0.301658000
N	0.266445000	2.034620000	-0.258549000
C	-2.081574000	2.761799000	-0.186318000
C	-2.678401000	1.516244000	-0.022657000
C	-4.093372000	1.289718000	0.051421000
C	-4.273507000	-0.056619000	0.188291000
C	-2.967900000	-0.653752000	0.205179000
N	-2.009926000	0.320951000	0.073030000
C	-2.721612000	-2.014905000	0.344515000
C	-1.465390000	-2.610889000	0.369116000
C	-1.232244000	-4.018705000	0.517452000
C	0.120707000	-4.201425000	0.504117000
C	0.715766000	-2.905412000	0.342027000
N	-0.266648000	-1.949023000	0.260820000
H	4.841924000	-1.983995000	0.042468000
H	5.198252000	0.693295000	-0.338870000
H	2.747463000	-3.527954000	0.348793000
H	3.583228000	2.749283000	-0.521450000
H	2.011957000	4.842933000	-0.700204000
H	-0.686340000	5.216671000	-0.567421000
Mn	0.005837000	0.064996000	0.157189000
H	-2.742441000	3.628438000	-0.240363000
H	-4.845544000	2.075053000	-0.004385000
H	-5.204864000	-0.614623000	0.271739000
H	-3.587316000	-2.672424000	0.439898000
H	-2.018619000	-4.764994000	0.618136000
H	0.684005000	-5.129358000	0.589924000
O	0.130910000	0.383873000	2.217661000
N	-0.755503000	-0.224877000	2.949418000
O	-0.671838000	0.006441000	4.139898000

FePorph-Cl (m6)

C	2.676689000	-1.459919000	0.016587000
C	4.094153000	-1.240766000	-0.081596000
C	4.277806000	0.105587000	-0.221800000
C	2.971547000	0.707969000	-0.210582000
N	2.011887000	-0.262733000	-0.070037000
C	2.068651000	-2.705750000	0.173612000
C	2.720281000	2.074309000	-0.333100000
C	1.467283000	2.687666000	-0.338129000
C	1.237487000	4.096282000	-0.510180000
C	-0.115203000	4.284494000	-0.471312000
C	-0.710554000	2.990523000	-0.271361000
N	0.269535000	2.033812000	-0.194727000
C	-2.080375000	2.742779000	-0.186707000
C	-2.686800000	1.496952000	-0.025091000
C	-4.105819000	1.270154000	-0.000460000
C	-4.289782000	-0.075869000	0.143569000
C	-2.982131000	-0.670973000	0.206910000
N	-2.020842000	0.303746000	0.106557000
C	-2.731794000	-2.037227000	0.329677000
C	-1.478828000	-2.649769000	0.337157000
C	-1.251951000	-4.065733000	0.434236000
C	0.100679000	-4.253939000	0.390480000
C	0.699047000	-2.952211000	0.264556000
N	-0.279967000	-1.990742000	0.233051000
H	4.846722000	-2.027310000	-0.044998000
H	5.212287000	0.655442000	-0.325890000
H	2.729870000	-3.573548000	0.220037000
H	3.588400000	2.727188000	-0.445733000
H	2.021015000	4.839780000	-0.649205000
H	-0.673435000	5.214362000	-0.571971000
Fe	0.020636000	0.065386000	0.461817000
H	-2.744410000	3.605325000	-0.272879000
H	-4.859502000	2.050746000	-0.094181000
H	-5.225229000	-0.631556000	0.193541000
H	-3.601405000	-2.693221000	0.406254000
H	-2.037638000	-4.815040000	0.520376000
H	0.657219000	-5.189254000	0.432741000
Cl	0.238946000	0.321408000	2.695749000

FePorph-Cl-OH (m3)

C	2.682252000	-1.444703000	0.144630000
C	4.095287000	-1.228765000	0.030440000
C	4.278349000	0.111203000	-0.151055000
C	2.977431000	0.714369000	-0.144064000
N	2.015151000	-0.247564000	0.042720000
C	2.097894000	-2.691045000	0.312020000
C	2.747503000	2.071937000	-0.309988000
C	1.495283000	2.670397000	-0.297514000
C	1.267050000	4.081756000	-0.442369000
C	-0.079637000	4.273024000	-0.352260000
C	-0.670487000	2.977782000	-0.155279000
N	0.305951000	2.017257000	-0.131140000
C	-2.032142000	2.753572000	-0.016228000
C	-2.621804000	1.509738000	0.168985000
C	-4.036339000	1.293055000	0.264112000
C	-4.221995000	-0.052952000	0.396043000
C	-2.920408000	-0.655179000	0.385044000
N	-1.958078000	0.313738000	0.259566000
C	-2.689039000	-2.020992000	0.481782000
C	-1.437324000	-2.619166000	0.465480000
C	-1.212658000	-4.031994000	0.603854000
C	0.136840000	-4.221461000	0.563552000
C	0.731735000	-2.924051000	0.396266000
N	-0.241339000	-1.966433000	0.333540000
H	4.841673000	-2.019886000	0.080538000
H	5.208128000	0.662339000	-0.282035000
H	2.762888000	-3.553572000	0.375223000
H	3.615796000	2.716798000	-0.452587000
H	2.055137000	4.818708000	-0.588163000
H	-0.644405000	5.202164000	-0.409042000
Fe	0.044768000	0.031500000	0.140215000
H	-2.693157000	3.620166000	-0.064614000
H	-4.782148000	2.085349000	0.225163000
H	-5.153592000	-0.608387000	0.490757000
H	-3.558107000	-2.672335000	0.585537000
H	-2.004474000	-4.770030000	0.721482000

H	0.700501000	-5.149833000	0.639907000
Cl	-0.167535000	-0.205914000	-2.119383000
O	0.145600000	0.212650000	1.922976000
H	-0.759728000	0.222314000	2.279596000

FePorph-Cl-Cl (m5)

C	2.646752000	-1.401582000	0.267674000
C	4.059759000	-1.192008000	0.123724000
C	4.224842000	0.079933000	-0.343802000
C	2.914541000	0.659980000	-0.439927000
N	1.970289000	-0.261792000	-0.075067000
C	2.052888000	-2.621680000	0.580014000
C	2.659104000	1.999622000	-0.719698000
C	1.424346000	2.627878000	-0.578452000
C	1.211433000	4.047595000	-0.623498000
C	-0.087727000	4.262077000	-0.262122000
C	-0.675649000	2.970058000	-0.042638000
N	0.262061000	1.993633000	-0.236386000
C	-2.025991000	2.738086000	0.202860000
C	-2.637652000	1.487222000	0.209735000
C	-4.055873000	1.267647000	0.201786000
C	-4.246665000	-0.072829000	0.022988000
C	-2.944164000	-0.675250000	-0.027361000
N	-1.981692000	0.292672000	0.081733000
C	-2.700701000	-2.045469000	-0.058262000
C	-1.453843000	-2.641918000	0.109887000
C	-1.227177000	-4.047447000	0.296441000
C	0.100149000	-4.198278000	0.579370000
C	0.688471000	-2.889241000	0.516420000
N	-0.276003000	-1.958638000	0.242713000
H	4.817589000	-1.944567000	0.334025000
H	5.148304000	0.605919000	-0.579981000
H	2.718025000	-3.459408000	0.796993000
H	3.515148000	2.628984000	-0.969228000
H	1.979165000	4.778180000	-0.872155000
H	-0.623003000	5.205555000	-0.169026000
Fe	-0.007077000	0.015758000	0.003361000
H	-2.674618000	3.610502000	0.299650000
H	-4.801285000	2.055733000	0.292720000
H	-5.182088000	-0.625381000	-0.047060000
H	-3.566870000	-2.705966000	-0.127179000
H	-2.002477000	-4.809321000	0.236456000
H	0.653801000	-5.112687000	0.785541000
Cl	-0.184267000	-0.298366000	-2.399492000
Cl	0.200068000	0.328297000	2.405201000

FePorph-Cl-F (m5)

C	2.717177000	-1.462335000	0.056164000
C	4.137272000	-1.248045000	-0.067905000
C	4.320704000	0.099497000	-0.210812000
C	3.010810000	0.702109000	-0.176166000
N	2.068166000	-0.266804000	-0.008786000
C	2.082188000	-2.708391000	0.204592000
C	2.732813000	2.073369000	-0.311089000
C	1.480185000	2.712526000	-0.313116000
C	1.252258000	4.123734000	-0.498713000
C	-0.101471000	4.312305000	-0.454783000
C	-0.694263000	3.015252000	-0.239529000
N	0.287418000	2.074646000	-0.153613000
C	-2.071042000	2.744338000	-0.151891000
C	-2.705832000	1.502055000	0.020676000
C	-4.130058000	1.274675000	0.021351000
C	-4.312648000	-0.072397000	0.165537000
C	-2.998518000	-0.662071000	0.253865000
N	-2.054642000	0.316759000	0.176108000
C	-2.719949000	-2.035051000	0.363776000
C	-1.467257000	-2.673871000	0.380875000
C	-1.246914000	-4.098164000	0.425407000
C	0.106342000	-4.288262000	0.381269000
C	0.707079000	-2.978287000	0.308554000
N	-0.269571000	-2.029017000	0.322734000
H	4.890993000	-2.033695000	-0.048185000
H	5.254994000	0.645121000	-0.333846000
H	2.742015000	-3.579713000	0.219960000
H	3.600132000	2.725213000	-0.444159000
H	2.032894000	4.867935000	-0.647264000
H	-0.657846000	5.242378000	-0.560420000
Fe	0.012716000	0.031953000	0.171757000
H	-2.734157000	3.607322000	-0.253118000
H	-4.886105000	2.050623000	-0.086975000
H	-5.248305000	-0.628330000	0.200249000
H	-3.589946000	-2.695235000	0.410349000
H	-2.032883000	-4.850128000	0.475550000
H	0.658154000	-5.226957000	0.387025000
Cl	-0.223563000	-0.271474000	-2.237839000
F	0.121309000	0.233254000	2.024519000

FePorph-Cl-N3 (m5)

C	2.639938000	-1.521810000	0.138146000
C	4.062015000	-1.300207000	0.197291000
C	4.255236000	0.049506000	0.103344000
C	2.949507000	0.647255000	-0.019551000
N	1.995060000	-0.325853000	-0.007279000
C	2.000738000	-2.763731000	0.248563000
C	2.682811000	2.023883000	-0.094408000
C	1.430129000	2.658156000	-0.171197000
C	1.213030000	4.081570000	-0.178797000
C	-0.140229000	4.272559000	-0.194394000
C	-0.745157000	2.964692000	-0.191634000
N	0.233383000	2.007773000	-0.194476000
C	-2.119208000	2.703161000	-0.148702000
C	-2.757393000	1.454803000	-0.051358000
C	-4.178864000	1.245182000	0.024152000
C	-4.372736000	-0.104568000	0.134949000
C	-3.067851000	-0.713091000	0.130013000
N	-2.111794000	0.255061000	0.010729000
C	-2.805709000	-2.085179000	0.250419000
C	-1.554548000	-2.720578000	0.286104000
C	-1.334381000	-4.135813000	0.426614000
C	0.020371000	-4.325924000	0.423201000
C	0.621465000	-3.025366000	0.281216000
N	-0.355071000	-2.075712000	0.198601000
H	4.809673000	-2.083480000	0.310485000
H	5.192458000	0.603238000	0.123539000
H	2.655944000	-3.633115000	0.342544000
H	3.553239000	2.684203000	-0.074731000

H	2.002035000	4.831696000	-0.160782000
H	-0.691825000	5.211320000	-0.192233000
Fe	-0.072808000	-0.060452000	-0.192836000
H	-2.775862000	3.576398000	-0.163949000
H	-4.927310000	2.035615000	-0.000937000
H	-5.312155000	-0.648456000	0.220535000
H	-3.678552000	-2.737157000	0.334390000
H	-2.121104000	-4.882833000	0.518391000
H	0.572654000	-5.260053000	0.512445000
Cl	-0.079936000	-0.332796000	-2.494957000
N	0.091515000	0.225928000	2.028262000
N	0.941083000	0.951582000	2.446529000
N	1.770514000	1.661380000	2.861161000

FePorph-Cl-SCN (m5)

C	2.731817000	-1.341041000	0.174097000
C	4.135614000	-1.155912000	-0.047351000
C	4.291770000	0.099721000	-0.561224000
C	2.987100000	0.692401000	-0.606683000
N	2.045792000	-0.200983000	-0.162543000
C	2.145310000	-2.537518000	0.572512000
C	2.732761000	2.023402000	-0.919652000
C	1.513728000	2.664764000	-0.726343000
C	1.314973000	4.082146000	-0.803701000
C	0.037500000	4.323505000	-0.384888000
C	-0.551995000	3.048683000	-0.099040000
N	0.363468000	2.047869000	-0.303975000
C	-1.886758000	2.850089000	0.237280000
C	-2.505343000	1.610379000	0.336657000
C	-3.915294000	1.413550000	0.502843000
C	-4.138800000	0.069823000	0.412369000
C	-2.858173000	-0.550660000	0.237455000
N	-1.873522000	0.399992000	0.187205000
C	-2.637007000	-1.923810000	0.269903000
C	-1.385024000	-2.524969000	0.357832000
C	-1.164677000	-3.908117000	0.663563000
C	0.179420000	-4.059882000	0.848393000
C	0.777953000	-2.779357000	0.607956000
N	-0.192702000	-1.851924000	0.319464000
H	4.894013000	-1.911049000	0.150677000
H	5.207596000	0.606440000	-0.860405000
H	2.810137000	-3.371839000	0.801926000
H	3.577536000	2.634774000	-1.241315000
H	2.077515000	4.793940000	-1.114671000
H	-0.481684000	5.275575000	-0.290412000
Fe	0.063066000	0.070369000	-0.177543000
H	-2.512960000	3.735801000	0.356741000
H	-4.634914000	2.216808000	0.649518000
H	-5.079697000	-0.472587000	0.485270000
H	-3.512016000	-2.572684000	0.334116000
H	-1.954453000	-4.651553000	0.754522000
H	0.736276000	-4.958905000	1.106330000
Cl	-0.223423000	-0.273255000	-2.465601000
S	0.366959000	0.447209000	2.614816000
C	-0.917001000	-0.479201000	3.060153000
N	-1.840189000	-1.144723000	3.383435000

FePorph-Cl-ONO2 (m5)

C	2.647243000	-1.503942000	-0.287265000
C	4.061429000	-1.268577000	-0.257686000
C	4.236169000	0.056713000	0.028855000
C	2.928509000	0.640033000	0.112871000
N	1.977743000	-0.328241000	-0.072846000
C	2.041890000	-2.756686000	-0.352301000
C	2.664577000	2.003209000	0.223126000
C	1.416856000	2.592052000	0.038626000
C	1.179223000	4.002852000	-0.073574000
C	-0.135783000	4.156673000	-0.408361000
C	-0.711353000	2.841670000	-0.440346000
N	0.254944000	1.906171000	-0.193685000
C	-2.075644000	2.572472000	-0.510211000
C	-2.656876000	1.346886000	-0.196049000
C	-4.059946000	1.125616000	-0.000154000
C	-4.197319000	-0.148865000	0.473554000
C	-2.882712000	-0.720753000	0.515304000
N	-1.958933000	0.210944000	0.120949000
C	-2.606422000	-2.060569000	0.774353000
C	-1.377104000	-2.678989000	0.563611000
C	-1.155132000	-4.097061000	0.571661000
C	0.123875000	-4.299322000	0.136658000
C	0.697698000	-3.001914000	-0.082666000
N	-0.234584000	-2.033695000	0.173176000
H	4.817206000	-2.038094000	-0.403902000
H	5.165662000	0.610738000	0.148311000
H	2.690254000	-3.620757000	-0.506885000
H	3.517428000	2.669412000	0.364048000
H	1.940683000	4.768005000	0.066089000
H	-0.692004000	5.076296000	-0.581707000
Fe	0.004521000	-0.069234000	-0.080160000
H	-2.746015000	3.412103000	-0.701486000
H	-4.831417000	1.872444000	-0.178312000
H	-5.107377000	-0.680283000	0.746315000
H	-3.445877000	-2.697980000	1.057271000
H	-1.904269000	-4.835643000	0.851221000
H	0.657388000	-5.238757000	0.002185000
Cl	-0.215673000	-0.325217000	-2.435005000
O	0.131889000	0.125074000	2.046328000
N	-0.757442000	0.820508000	2.683463000
O	-1.642892000	0.217224000	3.283190000
O	-0.666372000	2.045764000	2.666400000

FePorph-Cl-Me (m3)

C	2.634200000	-1.440913000	0.084459000
C	4.050965000	-1.225760000	0.019799000
C	4.240169000	0.118185000	-0.118693000
C	2.936975000	0.719626000	-0.141605000
N	1.969102000	-0.242994000	-0.018567000
C	2.042384000	-2.686836000	0.233390000
C	2.707418000	2.081469000	-0.277616000
C	1.453428000	2.672609000	-0.306691000
C	1.231594000	4.083633000	-0.436854000
C	-0.118687000	4.274872000	-0.408907000
C	-0.718467000	2.979688000	-0.261701000
N	0.253918000	2.010176000	-0.206620000
C	-2.084463000	2.759478000	-0.179008000
C	-2.677276000	1.513543000	-0.023740000

C	-4.093709000	1.297537000	0.055364000
C	-4.279043000	-0.046286000	0.198137000
C	-2.974507000	-0.645930000	0.208368000
N	-2.009637000	0.321215000	0.070457000
C	-2.746040000	-2.007466000	0.341842000
C	-1.494029000	-2.608148000	0.359019000
C	-1.274468000	-4.021193000	0.485348000
C	0.076038000	-4.213088000	0.454009000
C	0.674853000	-2.915572000	0.308983000
N	-0.296556000	-1.952764000	0.253256000
H	4.795355000	-2.018469000	0.076717000
H	5.173514000	0.673046000	-0.201424000
H	2.703109000	-3.553148000	0.292676000
H	3.576431000	2.735300000	-0.365351000
H	2.024691000	4.823333000	-0.534054000
H	-0.679493000	5.205758000	-0.477766000
Fe	-0.021887000	0.030357000	-0.031149000
H	-2.741700000	3.628398000	-0.236528000
H	-4.839548000	2.089168000	0.002504000
H	-5.210641000	-0.602803000	0.289162000
H	-3.617534000	-2.657333000	0.436097000
H	-2.067845000	-4.760696000	0.581913000
H	0.636688000	-5.144369000	0.518859000
Cl	-0.163067000	-0.318029000	-2.356746000
C	0.038091000	0.133288000	1.998974000
H	-0.940337000	-0.192837000	2.369243000
H	0.843028000	-0.532658000	2.329684000
H	0.245250000	1.182822000	2.240268000

FePorph-Cl-NO2 (m5)

C	2.642269000	-1.524348000	0.149343000
C	4.066809000	-1.339880000	0.164354000
C	4.293462000	0.003402000	0.063679000
C	3.006957000	0.638227000	-0.017658000
N	2.013115000	-0.308393000	0.036947000
C	1.996726000	-2.754569000	0.259239000
C	2.802739000	2.012298000	-0.124594000
C	1.569379000	2.658918000	-0.179223000
C	1.390238000	4.084274000	-0.225265000
C	0.043686000	4.313146000	-0.224236000
C	-0.596248000	3.026985000	-0.173060000
N	0.348966000	2.032597000	-0.159666000
C	-1.972373000	2.824780000	-0.087218000
C	-2.614406000	1.598111000	0.071119000
C	-4.034675000	1.427150000	0.205655000
C	-4.259867000	0.088631000	0.356513000
C	-2.976770000	-0.557235000	0.311994000
N	-1.986153000	0.378853000	0.141655000
C	-2.773785000	-1.930957000	0.427949000
C	-1.543671000	-2.585848000	0.399921000
C	-1.359968000	-4.002251000	0.561497000
C	-0.015115000	-4.234351000	0.506538000
C	0.619230000	-2.958754000	0.314543000
N	-0.328012000	-1.969601000	0.242605000
H	4.794319000	-2.145712000	0.250324000
H	5.245187000	0.532761000	0.048713000
H	2.631793000	-3.639283000	0.336747000
H	3.693162000	2.643735000	-0.147302000
H	2.202099000	4.810045000	-0.243295000
H	-0.483729000	5.265927000	-0.240509000
Fe	-0.008138000	-0.012726000	-0.404552000
H	-2.606329000	3.713293000	-0.113711000
H	-4.759446000	2.239900000	0.188675000
H	-5.208269000	-0.430044000	0.489841000
H	-3.661976000	-2.551727000	0.562235000
H	-2.166700000	-4.719401000	0.705771000
H	0.514892000	-5.181446000	0.597783000
Cl	-0.185788000	-0.270050000	-2.632677000
N	0.073051000	0.259712000	2.449110000
O	0.225514000	-0.777174000	3.018090000
O	-0.053883000	1.386080000	2.819817000

FePorph-Cl-ONO (m5)

C	2.612276000	-1.454696000	0.249278000
C	4.034905000	-1.284896000	0.198852000
C	4.264860000	0.041300000	-0.022323000
C	2.981318000	0.675979000	-0.097072000
N	1.977722000	-0.247485000	0.073449000
C	1.984282000	-2.676027000	0.443245000
C	2.798840000	2.037702000	-0.284917000
C	1.566734000	2.673801000	-0.286111000
C	1.393866000	4.092149000	-0.405240000
C	0.053804000	4.332840000	-0.321355000
C	-0.585234000	3.059750000	-0.158556000
N	0.349739000	2.052633000	-0.136334000
C	-1.955481000	2.887446000	-0.036067000
C	-2.584594000	1.662200000	0.123925000
C	-4.005028000	1.499313000	0.230278000
C	-4.239581000	0.160501000	0.342849000
C	-2.960380000	-0.486874000	0.313766000
N	-1.954048000	0.442717000	0.189226000
C	-2.783143000	-1.858563000	0.413434000
C	-1.550788000	-2.493533000	0.441378000
C	-1.373963000	-3.902980000	0.624888000
C	-0.028381000	-4.130328000	0.657502000
C	0.610552000	-2.860269000	0.480412000
N	-0.329787000	-1.863288000	0.358298000
H	4.754688000	-2.092729000	0.320638000
H	5.214872000	0.564498000	-0.120154000
H	2.617115000	-3.556536000	0.564040000
H	3.689156000	2.656133000	-0.409220000
H	2.209811000	4.803083000	-0.525188000
H	-0.474696000	5.284121000	-0.360101000
Fe	-0.002719000	0.064274000	-0.168473000
H	-2.584217000	3.778373000	-0.070622000
H	-4.719772000	2.320636000	0.214623000
H	-5.189628000	-0.362166000	0.442804000
H	-3.675707000	-2.480472000	0.496980000
H	-2.189765000	-4.616978000	0.725749000
H	0.502580000	-5.072080000	0.787719000
Cl	-0.176642000	-0.245715000	-2.447329000
O	0.166832000	0.244035000	2.727930000
N	-0.654144000	-0.608087000	2.902789000
O	-0.739408000	-1.533515000	3.646108000

FePorph (m5)

C	2.710911000	-1.480267000	0.030264000
C	4.131174000	-1.252889000	-0.044863000
C	4.314388000	0.096132000	-0.183669000
C	3.002993000	0.696976000	-0.194354000
N	2.052411000	-0.281805000	-0.065464000
C	2.086628000	-2.725454000	0.178833000
C	2.735795000	2.066454000	-0.317062000
C	1.480945000	2.689428000	-0.324717000
C	1.253022000	4.106895000	-0.453937000
C	-0.101298000	4.296585000	-0.410101000
C	-0.705103000	2.997494000	-0.254453000
N	0.277856000	2.041718000	-0.206718000
C	-2.079224000	2.741247000	-0.169384000
C	-2.703570000	1.495937000	-0.021900000
C	-4.123796000	1.268652000	0.053976000
C	-4.307057000	-0.080436000	0.192150000
C	-2.995717000	-0.681395000	0.201855000
N	-2.045128000	0.297337000	0.072681000
C	-2.728469000	-2.050818000	0.324938000
C	-1.473565000	-2.673645000	0.333419000
C	-1.245622000	-4.091066000	0.463207000
C	0.108714000	-4.280693000	0.420096000
C	0.712519000	-2.981629000	0.264215000
N	-0.270439000	-2.025875000	0.215876000
H	4.891156000	-2.032537000	0.005676000
H	5.252258000	0.644137000	-0.270401000
H	2.746554000	-3.594958000	0.235506000
H	3.603302000	2.723856000	-0.414991000
H	2.035385000	4.857238000	-0.563560000
H	-0.651722000	5.234956000	-0.477399000
Fe	0.003658000	0.007738000	0.003384000
H	-2.739113000	3.610831000	-0.225240000
H	-4.883751000	2.048387000	0.004326000
H	-5.244928000	-0.628358000	0.279389000
H	-3.595952000	-2.708262000	0.422788000
H	-2.027985000	-4.841404000	0.572869000
H	0.659165000	-5.219017000	0.487826000

FePorph-OH (m6)

C	2.708234000	-1.439293000	0.108761000
C	4.124372000	-1.231269000	-0.043442000
C	4.310834000	0.111578000	-0.211493000
C	3.007982000	0.723072000	-0.159522000
N	2.052052000	-0.238584000	0.035553000
C	2.094483000	-2.682300000	0.281879000
C	2.755870000	2.088991000	-0.302745000
C	1.503779000	2.707999000	-0.284514000
C	1.271263000	4.114240000	-0.486265000
C	-0.079411000	4.306136000	-0.412750000
C	-0.670528000	3.015959000	-0.166852000
N	0.310869000	2.063521000	-0.090677000
C	-2.040647000	2.768646000	-0.056339000
C	-2.652871000	1.524968000	0.114764000
C	-4.074353000	1.306559000	0.167645000
C	-4.263172000	-0.038939000	0.309973000
C	-2.955908000	-0.641889000	0.344793000
N	-1.994430000	0.327653000	0.226780000
C	-2.706398000	-2.010143000	0.467064000
C	-1.453611000	-2.627325000	0.464518000
C	-1.225962000	-4.044312000	0.571863000
C	0.126539000	-4.232146000	0.523285000
C	0.723940000	-2.929214000	0.384799000
N	-0.256316000	-1.971539000	0.349245000
H	4.873257000	-2.022292000	-0.029083000
H	5.244417000	0.652055000	-0.363695000
H	2.753796000	-3.552217000	0.321813000
H	3.621800000	2.735310000	-0.462378000
H	2.050813000	4.852925000	-0.668179000
H	-0.638555000	5.234454000	-0.524192000
Fe	0.057233000	0.091287000	0.594361000
H	-2.703568000	3.633045000	-0.136044000
H	-4.826207000	2.091267000	0.093056000
H	-5.201480000	-0.588205000	0.378427000
H	-3.576771000	-2.663511000	0.559242000
H	-2.010129000	-4.794253000	0.668053000
H	0.682730000	-5.167680000	0.570183000
O	0.149685000	0.264582000	2.428135000
H	-0.544931000	-0.176549000	2.935505000

FePorph-F (m6)

C	2.684201000	-1.457960000	0.025068000
C	4.101523000	-1.240886000	-0.086387000
C	4.285881000	0.105441000	-0.224930000
C	2.980268000	0.710031000	-0.197311000
N	2.021828000	-0.259396000	-0.049559000
C	2.074774000	-2.704243000	0.175796000
C	2.728548000	2.076662000	-0.317968000
C	1.475337000	2.690194000	-0.312659000
C	1.243875000	4.095778000	-0.507890000
C	-0.108716000	4.284004000	-0.466231000
C	-0.702454000	2.992976000	-0.242140000
N	0.278852000	2.039656000	-0.148620000
C	-2.072546000	2.743669000	-0.164142000
C	-2.679238000	1.497054000	-0.008178000
C	-4.098584000	1.270025000	0.000935000
C	-4.283946000	-0.076450000	0.138679000
C	-2.976751000	-0.671826000	0.212229000
N	-2.014970000	0.303378000	0.126561000
C	-2.727107000	-2.038978000	0.327187000
C	-1.473420000	-2.650481000	0.334075000
C	-1.245747000	-4.066954000	0.426064000
C	0.107014000	-4.253917000	0.384367000
C	0.704751000	-2.950818000	0.264850000
N	-0.275000000	-1.990678000	0.234793000
H	4.853022000	-2.029015000	-0.061798000
H	5.220165000	0.653740000	-0.339044000
H	2.735170000	-3.573034000	0.216047000
H	3.595370000	2.728554000	-0.445702000
H	2.026217000	4.836600000	-0.666823000
H	-0.667866000	5.211306000	-0.584541000
Fe	0.026662000	0.061548000	0.506913000
H	-2.737142000	3.604695000	-0.261168000
H	-4.851373000	2.050864000	-0.098288000
H	-5.219860000	-0.632365000	0.176694000
H	-3.596746000	-2.695793000	0.396258000
H	-2.031131000	-4.817062000	0.508705000

H	0.664150000	-5.189016000	0.424763000
F	0.249202000	0.161009000	2.313342000

FePorph-N3 (m6)

C	2.632501000	-1.417028000	0.163372000
C	4.050821000	-1.195421000	0.079427000
C	4.233955000	0.148392000	-0.088543000
C	2.927003000	0.749318000	-0.112409000
N	1.968905000	-0.222797000	0.036399000
C	2.022282000	-2.658575000	0.353459000
C	2.676763000	2.114010000	-0.270956000
C	1.426362000	2.734216000	-0.320949000
C	1.201310000	4.141631000	-0.511839000
C	-0.152659000	4.330982000	-0.512291000
C	-0.755978000	3.039418000	-0.318455000
N	0.223178000	2.086289000	-0.206339000
C	-2.128038000	2.788247000	-0.258401000
C	-2.738244000	1.545022000	-0.077862000
C	-4.157423000	1.316090000	-0.051242000
C	-4.340509000	-0.025143000	0.137571000
C	-3.032781000	-0.617286000	0.228873000
N	-2.074820000	0.356221000	0.093985000
C	-2.780674000	-1.977904000	0.414707000
C	-1.529248000	-2.594628000	0.475565000
C	-1.306606000	-4.009001000	0.612912000
C	0.046481000	-4.201408000	0.593873000
C	0.651023000	-2.904079000	0.444564000
N	-0.326054000	-1.944328000	0.382243000
H	4.805518000	-1.977827000	0.146676000
H	5.169620000	0.696828000	-0.189920000
H	2.683877000	-3.524285000	0.426577000
H	3.549450000	2.762534000	-0.374246000
H	1.987537000	4.885582000	-0.632307000
H	-0.705221000	5.261961000	-0.632700000
Fe	-0.039892000	0.128056000	0.428807000
H	-2.791580000	3.648441000	-0.369412000
H	-4.912912000	2.091697000	-0.168754000
H	-5.276651000	-0.577675000	0.207092000
H	-3.652514000	-2.630138000	0.500010000
H	-2.094351000	-4.755718000	0.702982000
H	0.598367000	-5.137783000	0.665094000
N	0.107572000	0.363031000	2.385769000
N	0.756848000	-0.275705000	3.166473000
N	1.372463000	-0.872349000	3.945119000

FePorph-SCN (m6)

C	2.756984000	-1.400405000	0.008753000
C	4.165589000	-1.198821000	-0.187654000
C	4.345342000	0.131550000	-0.447735000
C	3.046615000	0.746047000	-0.406719000
N	2.094848000	-0.204485000	-0.129890000
C	2.150274000	-2.627823000	0.282080000
C	2.791456000	2.104360000	-0.601626000
C	1.546025000	2.733647000	-0.546168000
C	1.317259000	4.140241000	-0.727967000
C	-0.025491000	4.349521000	-0.571931000
C	-0.619919000	3.069506000	-0.300276000
N	0.354035000	2.101292000	-0.292434000
C	-1.980987000	2.838548000	-0.092082000
C	-2.591801000	1.604499000	0.138105000
C	-4.006137000	1.394356000	0.275516000
C	-4.195430000	0.050301000	0.440576000
C	-2.895614000	-0.562710000	0.413713000
N	-1.934334000	0.401441000	0.233526000
C	-2.645613000	-1.927330000	0.561852000
C	-1.396005000	-2.549154000	0.553066000
C	-1.167778000	-3.956772000	0.725099000
C	0.184428000	-4.152530000	0.662680000
C	0.784431000	-2.865171000	0.444200000
N	-0.195308000	-1.903268000	0.388870000
H	4.917390000	-1.984843000	-0.132473000
H	5.275084000	0.662024000	-0.648907000
H	2.810848000	-3.494207000	0.355553000
H	3.653959000	2.740615000	-0.810652000
H	2.094714000	4.871914000	-0.942950000
H	-0.575661000	5.287304000	-0.635566000
Fe	0.113194000	0.140316000	0.331559000
H	-2.638506000	3.709302000	-0.134777000
H	-4.755850000	2.183494000	0.238951000
H	-5.132177000	-0.489823000	0.570770000
H	-3.514499000	-2.573517000	0.703204000
H	-1.951568000	-4.698100000	0.873151000
H	0.736888000	-5.087250000	0.747816000
S	0.462398000	0.559880000	2.736790000
C	-0.776678000	-0.385723000	3.306571000
N	-1.648258000	-1.049043000	3.738748000

FePorph-ONO2 (m6)

C	2.553122000	-1.583531000	0.127799000
C	3.979165000	-1.416547000	0.055096000
C	4.214338000	-0.075589000	-0.051510000
C	2.931591000	0.573920000	-0.043183000
N	1.932207000	-0.361416000	0.064572000
C	1.896903000	-2.809589000	0.223480000
C	2.737042000	1.949732000	-0.152596000
C	1.510851000	2.610998000	-0.199409000
C	1.348570000	4.030208000	-0.367316000
C	0.005248000	4.273807000	-0.384753000
C	-0.649009000	3.002385000	-0.228680000
N	0.283323000	2.003607000	-0.116868000
C	-2.029290000	2.810746000	-0.218946000
C	-2.685309000	1.584241000	-0.124409000
C	-4.111880000	1.409294000	-0.163865000
C	-4.347698000	0.068320000	-0.058294000
C	-3.064894000	-0.572524000	0.044179000
N	-2.064768000	0.367106000	0.000385000
C	-2.870361000	-1.948176000	0.153701000
C	-1.644029000	-2.608221000	0.207643000
C	-1.481725000	-4.035258000	0.275862000
C	-0.138366000	-4.279126000	0.296137000
C	0.516591000	-2.999819000	0.241283000
N	-0.415784000	-1.995579000	0.186277000
H	4.700013000	-2.232762000	0.075888000
H	5.168818000	0.441768000	-0.138930000
H	2.522064000	-3.703720000	0.267148000
H	3.633364000	2.568366000	-0.233487000

H	2.168287000	4.740140000	-0.468409000
H	-0.511129000	5.225511000	-0.502947000
Fe	-0.066230000	0.039050000	0.576858000
H	-2.654697000	3.700594000	-0.315438000
H	-4.833058000	2.219008000	-0.265919000
H	-5.302461000	-0.455870000	-0.056582000
H	-3.766377000	-2.572032000	0.175844000
H	-2.302127000	-4.751325000	0.296861000
H	0.377826000	-5.237253000	0.339157000
O	0.740542000	0.837198000	2.460641000
N	-0.107825000	0.233758000	3.193710000
O	-0.939589000	-0.481902000	2.553574000
O	-0.118954000	0.336635000	4.395637000

FePorph-Me (m6)

C	2.682488000	-1.455795000	0.029303000
C	4.099899000	-1.239012000	-0.092746000
C	4.283278000	0.107836000	-0.229772000
C	2.976904000	0.712145000	-0.192343000
N	2.020338000	-0.256932000	-0.034597000
C	2.073664000	-2.704666000	0.173754000
C	2.724848000	2.079426000	-0.321250000
C	1.470665000	2.693668000	-0.336410000
C	1.239145000	4.100641000	-0.533227000
C	-0.114258000	4.285692000	-0.503676000
C	-0.706577000	2.990818000	-0.288304000
N	0.275478000	2.041249000	-0.187536000
C	-2.078026000	2.737841000	-0.218388000
C	-2.686265000	1.489206000	-0.071377000
C	-4.107244000	1.263465000	-0.043467000
C	-4.290878000	-0.082661000	0.100324000
C	-2.980904000	-0.677439000	0.159304000
N	-2.022288000	0.296370000	0.050640000
C	-2.729222000	-2.044267000	0.292864000
C	-1.475211000	-2.658414000	0.309799000
C	-1.247895000	-4.076676000	0.395895000
C	0.105451000	-4.262696000	0.359165000
C	0.702594000	-2.956767000	0.252809000
N	-0.277299000	-1.999120000	0.224237000
H	4.852093000	-2.026950000	-0.080055000
H	5.216908000	0.655308000	-0.354194000
H	2.736174000	-3.572383000	0.207385000
H	3.593129000	2.730137000	-0.446696000
H	2.020733000	4.844366000	-0.683013000
H	-0.673920000	5.212499000	-0.624998000
Fe	0.015822000	0.063097000	0.525028000
H	-2.743092000	3.599538000	-0.308285000
H	-4.861990000	2.044014000	-0.131216000
H	-5.226868000	-0.637334000	0.155759000
H	-3.599323000	-2.700083000	0.368615000
H	-2.032562000	-4.828918000	0.467361000
H	0.662186000	-5.198431000	0.394149000
C	0.152225000	0.278944000	2.596808000
H	-0.845076000	0.448361000	3.036596000
H	0.585768000	-0.640110000	3.026882000
H	0.807012000	1.132745000	2.840104000

FePorph-NO2 (m6)

C	2.642510000	-1.535893000	0.138196000
C	4.066729000	-1.364076000	0.045047000
C	4.298757000	-0.022642000	-0.080979000
C	3.015768000	0.626513000	-0.069490000
N	2.024045000	-0.313158000	0.059952000
C	1.989842000	-2.761550000	0.285537000
C	2.810925000	2.003970000	-0.172321000
C	1.580035000	2.663713000	-0.176988000
C	1.398420000	4.085452000	-0.281762000
C	0.050965000	4.318159000	-0.248639000
C	-0.592535000	3.039020000	-0.125868000
N	0.356957000	2.047849000	-0.083207000
C	-1.972343000	2.833923000	-0.064939000
C	-2.628724000	1.606727000	0.052242000
C	-4.053766000	1.431436000	0.122213000
C	-4.284411000	0.089536000	0.248746000
C	-2.999766000	-0.556819000	0.260151000
N	-2.010062000	0.384518000	0.129022000
C	-2.791587000	-1.931244000	0.395720000
C	-1.560160000	-2.590086000	0.426820000
C	-1.380447000	-4.013863000	0.512803000
C	-0.033154000	-4.247551000	0.480943000
C	0.611744000	-2.966809000	0.378199000
N	-0.335714000	-1.975090000	0.357313000
H	4.792657000	-2.175512000	0.075444000
H	5.253417000	0.493083000	-0.176151000
H	2.622095000	-3.651272000	0.319453000
H	3.702830000	2.628268000	-0.257863000
H	2.206786000	4.810311000	-0.367502000
H	-0.471729000	5.272204000	-0.303325000
Fe	0.017301000	0.065947000	0.372603000
H	-2.602975000	3.724339000	-0.108852000
H	-4.781417000	2.240840000	0.080592000
H	-5.239253000	-0.427576000	0.334249000
H	-3.683641000	-2.556257000	0.475027000
H	-2.190112000	-4.739418000	0.578512000
H	0.488223000	-5.203269000	0.515397000
N	0.073848000	0.280166000	2.513421000
O	0.822398000	-0.454732000	3.147738000
O	-0.640523000	1.128500000	3.035258000

FePorph-ONO (m6)

C	2.649649000	-1.512936000	0.171329000
C	4.071831000	-1.339447000	0.057454000
C	4.298801000	0.000571000	-0.087867000
C	3.015081000	0.647022000	-0.063405000
N	2.026521000	-0.292093000	0.096994000
C	2.000690000	-2.740210000	0.315342000
C	2.805948000	2.020447000	-0.194932000
C	1.573318000	2.675182000	-0.199904000
C	1.388550000	4.092770000	-0.349686000
C	0.042370000	4.325141000	-0.300820000
C	-0.595783000	3.049475000	-0.123557000
N	0.353499000	2.060270000	-0.065919000
C	-1.973430000	2.844831000	-0.037469000
C	-2.621377000	1.616774000	0.099391000
C	-4.046434000	1.435759000	0.141538000
C	-4.272913000	0.093104000	0.259202000

C	-2.985985000	-0.546863000	0.293348000
N	-1.994842000	0.398699000	0.196056000
C	-2.780212000	-1.922370000	0.405514000
C	-1.548301000	-2.577501000	0.426188000
C	-1.365563000	-4.001163000	0.505279000
C	-0.018140000	-4.230475000	0.483681000
C	0.622937000	-2.946885000	0.390925000
N	-0.326792000	-1.956357000	0.362523000
H	4.798835000	-2.150043000	0.083068000
H	5.250025000	0.517651000	-0.206941000
H	2.634510000	-3.628637000	0.351699000
H	3.694612000	2.644130000	-0.312698000
H	2.194938000	4.813729000	-0.476346000
H	-0.484135000	5.275300000	-0.380330000
Fe	0.034484000	0.091296000	0.516455000
H	-2.608176000	3.731243000	-0.097772000
H	-4.775716000	2.242439000	0.081366000
H	-5.225952000	-0.431026000	0.317247000
H	-3.672133000	-2.549386000	0.466822000
H	-2.174520000	-4.728100000	0.563466000
H	0.507418000	-5.183777000	0.520674000
O	0.102861000	0.282516000	2.458911000
N	-0.844355000	-0.285947000	3.196442000
O	-0.729635000	-0.086281000	4.372849000

CoPorph (m2)

C	2.657045000	-1.427647000	-0.107579000
C	4.075099000	-1.214643000	-0.194811000
C	4.257756000	0.124507000	-0.365066000
C	2.948972000	0.718465000	-0.382436000
N	1.974773000	-0.239463000	-0.225584000
C	2.071191000	-2.671125000	0.075812000
C	2.717837000	2.076414000	-0.537985000
C	1.462491000	2.665110000	-0.551011000
C	1.240540000	4.075284000	-0.710750000
C	-0.107861000	4.264660000	-0.665799000
C	-0.698648000	2.968224000	-0.477640000
N	0.270092000	1.993929000	-0.410141000
C	-2.063871000	2.747550000	-0.380479000
C	-2.649769000	1.503990000	-0.197803000
C	-4.067848000	1.290867000	-0.111413000
C	-4.250528000	-0.048367000	0.058174000
C	-2.941715000	-0.642240000	0.076081000
N	-1.967486000	0.315788000	-0.079924000
C	-2.710588000	-2.000191000	0.231636000
C	-1.455214000	-2.588809000	0.245282000
C	-1.233242000	-3.998901000	0.405689000
C	0.115173000	-4.188250000	0.360973000
C	0.705968000	-2.891803000	0.172951000
N	-0.262798000	-1.917572000	0.104855000
H	4.821389000	-2.005183000	-0.127023000
H	5.187633000	0.682219000	-0.470049000
H	2.732139000	-3.536891000	0.149208000
H	3.585100000	2.729197000	-0.654870000
H	2.031994000	4.812584000	-0.838651000
H	-0.673480000	5.191957000	-0.748664000
Co	0.003659000	0.038192000	-0.152672000
H	-2.724803000	3.613351000	-0.453639000
H	-4.814140000	2.081412000	-0.179143000
H	-5.180428000	-0.606150000	0.162575000
H	-3.577877000	-2.653028000	0.348017000
H	-2.024686000	-4.736196000	0.533680000
H	0.680771000	-5.115520000	0.444289000

CoPorph-OH (m1)

C	2.650743000	-1.427054000	0.143792000
C	4.068826000	-1.215666000	0.047582000
C	4.248519000	0.114060000	-0.185039000
C	2.938594000	0.705514000	-0.219320000
N	1.971441000	-0.243986000	-0.005502000
C	2.064320000	-2.668574000	0.335929000
C	2.707385000	2.057890000	-0.415215000
C	1.457140000	2.656353000	-0.396900000
C	1.242869000	4.069257000	-0.539197000
C	-0.100203000	4.272933000	-0.434953000
C	-0.697473000	2.980892000	-0.240914000
N	0.265117000	2.001669000	-0.206784000
C	-2.061891000	2.763266000	-0.132935000
C	-2.648974000	1.513899000	-0.005291000
C	-4.067478000	1.294114000	0.021266000
C	-4.252053000	-0.053794000	0.090227000
C	-2.944087000	-0.647400000	0.120666000
N	-1.970851000	0.321004000	0.074700000
C	-2.716998000	-2.011178000	0.213267000
C	-1.463900000	-2.598141000	0.295208000
C	-1.247917000	-4.011618000	0.434960000
C	0.100395000	-4.194779000	0.494128000
C	0.697155000	-2.892195000	0.376990000
N	-0.269247000	-1.923056000	0.276686000
H	4.815691000	-2.001995000	0.147055000
H	5.176775000	0.668408000	-0.317045000
H	2.724351000	-3.532687000	0.430222000
H	3.573973000	2.702341000	-0.573706000
H	2.036722000	4.799579000	-0.688812000
H	-0.659093000	5.206395000	-0.484532000
Co	-0.007061000	0.024045000	-0.067874000
H	-2.721542000	3.631340000	-0.180771000
H	-4.812731000	2.087255000	-0.019602000
H	-5.182776000	-0.618420000	0.123853000
H	-3.587574000	-2.669038000	0.237575000
H	-2.043697000	-4.753597000	0.484089000
H	0.663236000	-5.121336000	0.599072000
O	-0.084785000	-0.210876000	-1.885925000
H	-0.694782000	0.481232000	-2.181743000

CoPorph-Cl (m1)

C	2.639635000	-1.428871000	0.100493000
C	4.056768000	-1.214408000	0.023679000
C	4.239407000	0.125168000	-0.137382000
C	2.931546000	0.718500000	-0.159531000
N	1.959169000	-0.238672000	0.004786000
C	2.054339000	-2.675168000	0.251861000
C	2.703868000	2.074813000	-0.321623000
C	1.451149000	2.665342000	-0.337729000
C	1.230950000	4.074363000	-0.502235000
C	-0.117052000	4.264597000	-0.464861000

C	-0.708807000	2.969833000	-0.275916000
N	0.259161000	1.999953000	-0.184991000
C	-2.072404000	2.745689000	-0.189958000
C	-2.653945000	1.502194000	-0.005130000
C	-4.072067000	1.286635000	0.046173000
C	-4.257305000	-0.053984000	0.194703000
C	-2.949926000	-0.646521000	0.236765000
N	-1.973848000	0.316227000	0.139039000
C	-2.724728000	-2.007663000	0.351926000
C	-1.471569000	-2.596282000	0.376670000
C	-1.252410000	-4.010662000	0.482412000
C	0.096185000	-4.198432000	0.457241000
C	0.690041000	-2.896725000	0.333478000
N	-0.277021000	-1.920962000	0.306408000
H	4.801629000	-2.005717000	0.092575000
H	5.168306000	0.685754000	-0.231290000
H	2.713996000	-3.543158000	0.299413000
H	3.572457000	2.724170000	-0.443746000
H	2.024547000	4.809835000	-0.624324000
H	-0.682712000	5.191239000	-0.549870000
Co	-0.017476000	0.018844000	-0.063539000
H	-2.736677000	3.606464000	-0.283049000
H	-4.815685000	2.078284000	-0.031470000
H	-5.187344000	-0.615745000	0.267633000
H	-3.594911000	-2.663308000	0.413534000
H	-2.047090000	-4.750603000	0.564117000
H	0.661988000	-5.127170000	0.513314000
Cl	-0.178580000	-0.282340000	-2.255068000

CoPorph-F (m1)

C	2.634310000	-1.442347000	0.099027000
C	4.051344000	-1.228145000	0.016953000
C	4.234792000	0.112650000	-0.134773000
C	2.927697000	0.708253000	-0.145344000
N	1.957350000	-0.250271000	0.016324000
C	2.048431000	-2.689813000	0.241844000
C	2.700461000	2.066079000	-0.297856000
C	1.447555000	2.657239000	-0.311389000
C	1.226858000	4.066577000	-0.472846000
C	-0.121451000	4.256468000	-0.437159000
C	-0.714225000	2.961185000	-0.253656000
N	0.254921000	1.993239000	-0.163684000
C	-2.078970000	2.737545000	-0.176638000
C	-2.662984000	1.492242000	-0.009756000
C	-4.081147000	1.275846000	0.038875000
C	-4.266686000	-0.066240000	0.176243000
C	-2.959492000	-0.660034000	0.213632000
N	-1.985494000	0.304026000	0.121005000
C	-2.733411000	-2.021606000	0.328190000
C	-1.479582000	-2.609998000	0.354770000
C	-1.260343000	-4.024098000	0.465755000
C	0.088417000	-4.212594000	0.443312000
C	0.683539000	-2.911717000	0.317308000
N	-0.284078000	-1.937484000	0.282737000
H	4.795796000	-2.020858000	0.073740000
H	5.163985000	0.672300000	-0.231597000
H	2.707943000	-3.557981000	0.286675000
H	3.569103000	2.715416000	-0.419263000
H	2.020186000	4.802486000	-0.594276000
H	-0.686970000	5.183151000	-0.523115000
Co	-0.018665000	0.011392000	-0.048381000
H	-2.741614000	3.600079000	-0.264317000
H	-4.825038000	2.067986000	-0.031357000
H	-5.197097000	-0.627876000	0.245694000
H	-3.603187000	-2.677665000	0.390263000
H	-2.055182000	-4.763701000	0.549275000
H	0.653360000	-5.141603000	0.503859000
F	-0.081781000	-0.175537000	-1.829282000

CoPorph-N3 (m1)

C	2.631682000	-1.432260000	0.067925000
C	4.049675000	-1.220151000	-0.005086000
C	4.235940000	0.122096000	-0.139299000
C	2.929306000	0.719446000	-0.151702000
N	1.955925000	-0.239167000	-0.008754000
C	2.041126000	-2.679077000	0.200423000
C	2.703100000	2.078646000	-0.295006000
C	1.450649000	2.671024000	-0.313749000
C	1.232235000	4.081451000	-0.471178000
C	-0.116362000	4.271076000	-0.453665000
C	-0.710731000	2.974757000	-0.282029000
N	0.256289000	2.005466000	-0.184386000
C	-2.076270000	2.749737000	-0.218932000
C	-2.662361000	1.507166000	-0.042271000
C	-4.080558000	1.293030000	0.001667000
C	-4.269488000	-0.046675000	0.156931000
C	-2.964275000	-0.642070000	0.210097000
N	-1.986165000	0.318053000	0.106443000
C	-2.739635000	-2.004238000	0.324656000
C	-1.486272000	-2.594359000	0.354134000
C	-1.267443000	-4.008949000	0.451231000
C	0.080775000	-4.198589000	0.411027000
C	0.676792000	-2.898283000	0.288311000
N	-0.290992000	-1.920700000	0.271813000
H	4.792718000	-2.014468000	0.047366000
H	5.166484000	0.681531000	-0.223296000
H	2.697665000	-3.550030000	0.234723000
H	3.572913000	2.728966000	-0.402414000
H	2.027073000	4.817903000	-0.578662000
H	-0.680716000	5.198106000	-0.543143000
Co	-0.023776000	0.023643000	-0.060576000
H	-2.738692000	3.611297000	-0.317649000
H	-4.822741000	2.085152000	-0.083985000
H	-5.201152000	-0.605844000	0.228088000
H	-3.610190000	-2.659067000	0.388190000
H	-2.062285000	-4.748220000	0.536397000
H	0.645123000	-5.128769000	0.456274000
N	-0.110384000	-0.194057000	-1.954523000
N	-1.006650000	-0.885029000	-2.374339000
N	-1.834158000	-1.532508000	-2.862173000

CoPorph-SCN (m1)

C	2.633045000	-1.421866000	0.185314000
C	4.048954000	-1.212403000	0.084738000
C	4.230124000	0.113280000	-0.168523000
C	2.924111000	0.707758000	-0.204750000

N	1.952072000	-0.236997000	0.028138000
C	2.046108000	-2.662820000	0.371511000
C	2.696100000	2.057484000	-0.416201000
C	1.449507000	2.658831000	-0.377132000
C	1.232396000	4.070332000	-0.513620000
C	-0.106093000	4.275245000	-0.364673000
C	-0.699129000	2.984851000	-0.156960000
N	0.261344000	2.002659000	-0.153148000
C	-2.061518000	2.768575000	-0.035509000
C	-2.650672000	1.518563000	0.059326000
C	-4.068952000	1.303770000	0.074846000
C	-4.259768000	-0.044613000	0.089288000
C	-2.955728000	-0.644243000	0.102524000
N	-1.974964000	0.321396000	0.094674000
C	-2.732547000	-2.009292000	0.157816000
C	-1.481250000	-2.596740000	0.250391000
C	-1.264901000	-4.008602000	0.383614000
C	0.081714000	-4.190218000	0.477005000
C	0.679943000	-2.888846000	0.383054000
N	-0.285779000	-1.916492000	0.265739000
H	4.793904000	-1.999260000	0.191103000
H	5.158203000	0.664321000	-0.312940000
H	2.705803000	-3.525954000	0.474013000
H	3.562265000	2.696060000	-0.597333000
H	2.022620000	4.799013000	-0.686691000
H	-0.666089000	5.208624000	-0.394116000
Co	-0.015395000	0.029490000	-0.052989000
H	-2.719172000	3.638956000	-0.056770000
H	-4.809694000	2.101836000	0.065481000
H	-5.192104000	-0.607003000	0.100105000
H	-3.603220000	-2.667070000	0.156130000
H	-2.060852000	-4.751255000	0.408840000
H	0.643507000	-5.115972000	0.591552000
S	-0.004428000	-0.275477000	-2.289938000
C	-0.948066000	0.989276000	-2.811715000
N	-1.609591000	1.870145000	-3.225361000

CoPorph-ONO2 (m1)

C	2.599890000	-1.475287000	-0.048046000
C	4.017243000	-1.256971000	-0.099108000
C	4.205193000	0.092018000	-0.068829000
C	2.899221000	0.689439000	-0.031037000
N	1.927047000	-0.279649000	0.011790000
C	2.005595000	-2.726888000	-0.007612000
C	2.671992000	2.054669000	-0.100183000
C	1.419192000	2.638221000	-0.208110000
C	1.200719000	4.041195000	-0.422214000
C	-0.142608000	4.208266000	-0.575665000
C	-0.738089000	2.910576000	-0.423962000
N	0.224758000	1.961841000	-0.185284000
C	-2.102661000	2.669875000	-0.451081000
C	-2.683146000	1.444404000	-0.166019000
C	-4.097761000	1.231814000	-0.049145000
C	-4.267638000	-0.068382000	0.319838000
C	-2.957901000	-0.651884000	0.391369000
N	-1.994968000	0.288236000	0.116212000
C	-2.722944000	-1.994914000	0.631824000
C	-1.480527000	-2.599939000	0.538592000
C	-1.268375000	-4.016464000	0.625653000
C	0.055118000	-4.230175000	0.381157000
C	0.649543000	-2.939026000	0.180653000
N	-0.297762000	-1.949342000	0.285676000
H	4.760139000	-2.052219000	-0.137824000
H	5.136297000	0.656428000	-0.085523000
H	2.655321000	-3.601332000	-0.069597000
H	3.542224000	2.712390000	-0.130444000
H	1.993465000	4.786818000	-0.460359000
H	-0.703505000	5.123323000	-0.759786000
Co	-0.046111000	-0.018457000	-0.085011000
H	-2.765386000	3.511855000	-0.657410000
H	-4.849551000	2.002127000	-0.213095000
H	-5.191457000	-0.610395000	0.516625000
H	-3.583795000	-2.631735000	0.842183000
H	-2.052578000	-4.742623000	0.833785000
H	0.605721000	-5.169282000	0.353905000
O	0.085012000	-0.092992000	-1.958409000
N	-0.738643000	-0.773185000	-2.740505000
O	-1.668289000	-1.395363000	-2.268088000
O	-0.461111000	-0.696483000	-3.926348000

CoPorph-Me (m1)

C	2.637249000	-1.443898000	0.059368000
C	4.055949000	-1.230207000	-0.011351000
C	4.239843000	0.110701000	-0.161373000
C	2.930956000	0.704386000	-0.183151000
N	1.955872000	-0.254015000	-0.038641000
C	2.051121000	-2.690541000	0.211726000
C	2.703495000	2.062603000	-0.336248000
C	1.449783000	2.653211000	-0.353201000
C	1.229797000	4.064114000	-0.509743000
C	-0.119069000	4.252230000	-0.484657000
C	-0.711062000	2.954312000	-0.310844000
N	0.257213000	1.983006000	-0.222641000
C	-2.076765000	2.729914000	-0.240883000
C	-2.662494000	1.486559000	-0.060784000
C	-4.081282000	1.273255000	0.008745000
C	-4.265851000	-0.066641000	0.168176000
C	-2.957294000	-0.660343000	0.196348000
N	-1.981477000	0.299125000	0.068302000
C	-2.728581000	-2.019829000	0.334609000
C	-1.473653000	-2.607194000	0.362557000
C	-1.254245000	-4.019093000	0.509223000
C	0.093929000	-4.208752000	0.471748000
C	0.686293000	-2.910354000	0.303188000
N	-0.280043000	-1.934167000	0.249195000
H	4.800983000	-2.021998000	0.054049000
H	5.169933000	0.670897000	-0.248397000
H	2.710998000	-3.558040000	0.269988000
H	3.572653000	2.713888000	-0.445752000
H	2.023011000	4.802028000	-0.621405000
H	-0.684468000	5.179256000	-0.570523000
Co	-0.018917000	0.012165000	-0.079662000
H	-2.738676000	3.593542000	-0.327882000
H	-4.826022000	2.064970000	-0.060588000
H	-5.196223000	-0.625485000	0.260677000
H	-3.597230000	-2.674388000	0.427277000

H	-2.047555000	-4.756155000	0.625591000
H	0.659282000	-5.136426000	0.550533000
C	-0.145917000	-0.170380000	-2.000021000
H	0.865420000	-0.160085000	-2.433173000
H	-0.650713000	-1.117499000	-2.242465000
H	-0.729783000	0.675066000	-2.393639000

CoPorph-NO2 (m1)

C	2.635594000	-1.468330000	0.069573000
C	4.054585000	-1.259336000	0.014660000
C	4.247409000	0.084413000	-0.095221000
C	2.943436000	0.686208000	-0.117046000
N	1.965097000	-0.271898000	-0.002473000
C	2.043994000	-2.714116000	0.199981000
C	2.723019000	2.046577000	-0.255094000
C	1.468658000	2.632155000	-0.308040000
C	1.245650000	4.038996000	-0.486397000
C	-0.104253000	4.218231000	-0.517872000
C	-0.695839000	2.920614000	-0.349000000
N	0.274908000	1.959221000	-0.212720000
C	-2.061381000	2.693652000	-0.310359000
C	-2.649246000	1.456861000	-0.101635000
C	-4.068112000	1.250111000	-0.032945000
C	-4.258763000	-0.080859000	0.183696000
C	-2.954204000	-0.678390000	0.239550000
N	-1.975975000	0.273267000	0.080164000
C	-2.732460000	-2.034669000	0.410663000
C	-1.478961000	-2.623855000	0.423201000
C	-1.257434000	-4.035089000	0.561104000
C	0.088924000	-4.227292000	0.485082000
C	0.680653000	-2.930882000	0.308530000
N	-0.286182000	-1.955377000	0.284858000
H	4.794568000	-2.056645000	0.066356000
H	5.180569000	0.642204000	-0.159018000
H	2.700765000	-3.584736000	0.239574000
H	3.594032000	2.698429000	-0.339742000
H	2.038641000	4.779818000	-0.576569000
H	-0.671555000	5.140323000	-0.636263000
Co	-0.009117000	-0.015202000	-0.073745000
H	-2.722532000	3.552911000	-0.435234000
H	-4.808948000	2.041239000	-0.138077000
H	-5.191113000	-0.632747000	0.294234000
H	-3.602097000	-2.684192000	0.522962000
H	-2.049295000	-4.770618000	0.693824000
H	0.654649000	-5.155899000	0.545473000
N	-0.079347000	-0.241108000	-1.944350000
O	0.557579000	-1.158330000	-2.422681000
O	-0.756889000	0.546349000	-2.574390000

CoPorph-ONO (m1)

C	2.634010000	-1.453393000	0.048957000
C	4.051493000	-1.236973000	-0.009502000
C	4.236791000	0.109513000	-0.094734000
C	2.929779000	0.705396000	-0.100939000
N	1.957329000	-0.258951000	0.006375000
C	2.045319000	-2.702810000	0.156746000
C	2.703566000	2.066310000	-0.222940000
C	1.449672000	2.652959000	-0.276927000
C	1.230529000	4.060572000	-0.454721000
C	-0.118447000	4.242463000	-0.500207000
C	-0.713267000	2.945728000	-0.337610000
N	0.254118000	1.983028000	-0.188455000
C	-2.078817000	2.716826000	-0.309518000
C	-2.661481000	1.477689000	-0.100091000
C	-4.078850000	1.262158000	-0.038702000
C	-4.262514000	-0.069367000	0.180367000
C	-2.955820000	-0.660236000	0.242540000
N	-1.981935000	0.297689000	0.091109000
C	-2.729080000	-2.016347000	0.403331000
C	-1.476488000	-2.606369000	0.404123000
C	-1.258436000	-4.020418000	0.509288000
C	0.086522000	-4.216175000	0.419474000
C	0.682354000	-2.918728000	0.270214000
N	-0.281826000	-1.938704000	0.278038000
H	4.795198000	-2.031807000	0.021770000
H	5.166787000	0.673028000	-0.152990000
H	2.701246000	-3.574664000	0.169711000
H	3.573559000	2.720417000	-0.300760000
H	2.025830000	4.799888000	-0.536162000
H	-0.683091000	5.165289000	-0.624486000
Co	-0.021856000	0.000516000	-0.066311000
H	-2.742168000	3.572528000	-0.445156000
H	-4.823455000	2.048672000	-0.150519000
H	-5.192272000	-0.626178000	0.287077000
H	-3.597553000	-2.668338000	0.509833000
H	-2.052176000	-4.755918000	0.629923000
H	0.649289000	-5.147814000	0.452687000
O	-0.017438000	-0.155374000	-1.916508000
N	-0.959786000	-0.941133000	-2.420277000
O	-0.893552000	-0.989138000	-3.622653000

NiPorph-Cl (m2)

C	2.644780000	-1.424625000	0.094093000
C	4.059477000	-1.210802000	-0.001746000
C	4.239728000	0.121132000	-0.218286000
C	2.933823000	0.714475000	-0.245883000
N	1.965862000	-0.237251000	-0.036265000
C	2.068623000	-2.668925000	0.275537000
C	2.710426000	2.065658000	-0.436476000
C	1.458261000	2.652349000	-0.418854000
C	1.236273000	4.062005000	-0.559921000
C	-0.107740000	4.257838000	-0.462083000
C	-0.699365000	2.965004000	-0.270972000
N	0.268891000	1.991980000	-0.231779000
C	-2.060279000	2.751644000	-0.153041000
C	-2.637175000	1.504095000	0.002475000
C	-4.053057000	1.287763000	0.070766000
C	-4.238734000	-0.057402000	0.168715000
C	-2.933964000	-0.654115000	0.172492000
N	-1.961017000	0.312019000	0.090516000
C	-2.715070000	-2.016043000	0.266003000
C	-1.460570000	-2.596126000	0.311623000
C	-1.237696000	-4.006135000	0.447148000
C	0.111660000	-4.188139000	0.468054000
C	0.704700000	-2.888467000	0.334963000
N	-0.266094000	-1.920793000	0.261351000

H	4.803421000	-2.000525000	0.088607000
H	5.165987000	0.679504000	-0.344275000
H	2.730678000	-3.532194000	0.352715000
H	3.575830000	2.710927000	-0.592370000
H	2.029374000	4.793977000	-0.702765000
H	-0.674256000	5.186415000	-0.509629000
Ni	-0.008790000	0.015833000	-0.140017000
H	-2.721377000	3.617592000	-0.203378000
H	-4.794219000	2.084705000	0.038601000
H	-5.167202000	-0.621706000	0.239457000
H	-3.584788000	-2.672198000	0.316965000
H	-2.032983000	-4.745923000	0.522062000
H	0.680771000	-5.111765000	0.560544000
Cl	-0.192437000	-0.308448000	-2.479094000

NiPorph-Cl-OH (m1)

C	2.639086000	-1.368288000	0.293274000
C	4.052381000	-1.165133000	0.130858000
C	4.215346000	0.086593000	-0.382649000
C	2.905440000	0.669559000	-0.482828000
N	1.966685000	-0.237392000	-0.077635000
C	2.056735000	-2.575847000	0.643293000
C	2.665073000	1.992963000	-0.815521000
C	1.433297000	2.611219000	-0.670830000
C	1.223112000	4.029465000	-0.748916000
C	-0.066588000	4.260680000	-0.371588000
C	-0.663653000	2.980146000	-0.116846000
N	0.268445000	1.994933000	-0.302419000
C	-2.007679000	2.778595000	0.151370000
C	-2.605319000	1.528685000	0.144309000
C	-4.022437000	1.302942000	0.144286000
C	-4.211002000	-0.034018000	-0.046190000
C	-2.908595000	-0.636417000	-0.104640000
N	-1.949886000	0.335240000	0.000069000
C	-2.679345000	-2.002185000	-0.134596000
C	-1.434679000	-2.575756000	0.071359000
C	-1.213496000	-3.979682000	0.281983000
C	0.101051000	-4.127951000	0.609012000
C	0.696872000	-2.821628000	0.542672000
N	-0.259683000	-1.895814000	0.231701000
H	4.806271000	-1.917908000	0.355307000
H	5.134648000	0.605778000	-0.649167000
H	2.711998000	-3.407340000	0.904866000
H	3.515124000	2.609557000	-1.109260000
H	1.994600000	4.745166000	-1.028230000
H	-0.600219000	5.206755000	-0.294953000
Ni	0.008628000	0.052805000	-0.026573000
H	-2.646557000	3.654041000	0.270908000
H	-4.765464000	2.092296000	0.245439000
H	-5.143148000	-0.592967000	-0.111979000
H	-3.540437000	-2.664858000	-0.226914000
H	-1.992797000	-4.736737000	0.210660000
H	0.652436000	-5.037374000	0.842510000
O	-0.066680000	-0.209781000	-1.871337000
H	-0.660814000	0.498372000	-2.169213000
Cl	0.146220000	0.364244000	2.255160000

NiPorph-Cl-Cl (m3)

C	2.608355000	-1.430139000	0.032476000
C	4.025060000	-1.211986000	-0.044699000
C	4.207962000	0.128717000	-0.205419000
C	2.901528000	0.725016000	-0.228972000
N	1.940712000	-0.240166000	-0.085174000
C	2.043222000	-2.695176000	0.206172000
C	2.696824000	2.097763000	-0.376194000
C	1.465547000	2.746136000	-0.399834000
C	1.254045000	4.166538000	-0.526089000
C	-0.095468000	4.356209000	-0.484836000
C	-0.682278000	3.047317000	-0.333097000
N	0.281045000	2.090626000	-0.289037000
C	-2.040148000	2.761942000	-0.231987000
C	-2.605298000	1.496904000	-0.058407000
C	-4.021984000	1.278733000	0.018939000
C	-4.204865000	-0.062022000	0.179230000
C	-2.898422000	-0.658327000	0.202471000
N	-1.937629000	0.306883000	0.058754000
C	-2.693777000	-2.031115000	0.349417000
C	-1.462518000	-2.679525000	0.372993000
C	-1.250977000	-4.099866000	0.499878000
C	0.098548000	-4.289497000	0.458901000
C	0.685343000	-2.980647000	0.306820000
N	-0.278037000	-2.024021000	0.261999000
H	4.769468000	-2.003256000	0.022543000
H	5.136344000	0.688992000	-0.300906000
H	2.740834000	-3.533409000	0.264206000
H	3.593157000	2.714305000	-0.471601000
H	2.045050000	4.907660000	-0.627361000
H	-0.657479000	5.286757000	-0.544794000
Ni	0.001541000	0.033352000	-0.013319000
H	-2.737690000	3.600263000	-0.289581000
H	-4.766382000	2.070048000	-0.047904000
H	-5.133233000	-0.622321000	0.274718000
H	-3.590142000	-2.647608000	0.444822000
H	-2.041964000	-4.840991000	0.601263000
H	0.660566000	-5.220014000	0.519278000
Cl	-0.186328000	-0.303925000	-2.330224000
Cl	0.189276000	0.370550000	2.303547000

NiPorph-Cl-F (m3)

C	2.609177000	-1.444319000	0.034259000
C	4.027258000	-1.226144000	-0.050052000
C	4.210564000	0.117074000	-0.198455000
C	2.903057000	0.715662000	-0.208049000
N	1.947519000	-0.251942000	-0.065550000
C	2.036254000	-2.711474000	0.200365000
C	2.691478000	2.092752000	-0.344340000
C	1.461608000	2.751671000	-0.370778000
C	1.249918000	4.175419000	-0.481393000
C	-0.100840000	4.364389000	-0.445454000
C	-0.689260000	3.051660000	-0.314172000
N	0.276125000	2.099702000	-0.276661000
C	-2.048903000	2.754634000	-0.219698000
C	-2.622573000	1.486523000	-0.062727000
C	-4.040613000	1.268719000	0.021984000
C	-4.223838000	-0.074063000	0.174854000
C	-2.916208000	-0.672262000	0.186188000

N	-1.960191000	0.294297000	0.038201000
C	-2.703799000	-2.047768000	0.336787000
C	-1.473616000	-2.706546000	0.360836000
C	-1.261369000	-4.127839000	0.497841000
C	0.089173000	-4.317728000	0.457577000
C	0.676635000	-3.008130000	0.297070000
N	-0.288880000	-2.056693000	0.245467000
H	4.773787000	-2.016787000	0.002126000
H	5.140657000	0.674298000	-0.296830000
H	2.735569000	-3.549130000	0.254465000
H	3.589503000	2.708573000	-0.433227000
H	2.038765000	4.920729000	-0.569856000
H	-0.659637000	5.297513000	-0.497858000
Ni	-0.003449000	0.019968000	-0.017971000
H	-2.747680000	3.593236000	-0.266088000
H	-4.787377000	2.058882000	-0.033985000
H	-5.153972000	-0.631164000	0.273594000
H	-3.601126000	-2.662547000	0.439154000
H	-2.049713000	-4.871095000	0.605532000
H	0.648179000	-5.249735000	0.525267000
F	-0.075601000	-0.220049000	-1.844705000
Cl	0.129572000	0.340446000	2.251166000

NiPorph-Cl-N3 (m3)

C	2.753998000	-1.285761000	0.153360000
C	4.176001000	-1.121049000	-0.028023000
C	4.367113000	0.181201000	-0.385169000
C	3.058323000	0.789570000	-0.412841000
N	2.102917000	-0.118634000	-0.087749000
C	2.098115000	-2.458770000	0.523502000
C	2.771052000	2.118843000	-0.719534000
C	1.507475000	2.718897000	-0.726458000
C	1.291459000	4.100060000	-1.059964000
C	-0.050784000	4.321585000	-0.967169000
C	-0.650428000	3.074550000	-0.578082000
N	0.314477000	2.114717000	-0.436135000
C	-2.027468000	2.909589000	-0.395383000
C	-2.683900000	1.735954000	-0.027887000
C	-4.104743000	1.576254000	0.164144000
C	-4.298566000	0.275059000	0.524823000
C	-2.992218000	-0.336992000	0.544500000
N	-2.035428000	0.565027000	0.206623000
C	-2.703971000	-1.667275000	0.848435000
C	-1.437550000	-2.263463000	0.865281000
C	-1.221203000	-3.644025000	1.195452000
C	0.121441000	-3.865589000	1.102860000
C	0.722467000	-2.620256000	0.714349000
N	-0.243550000	-1.659886000	0.571652000
H	4.917667000	-1.907144000	0.103523000
H	5.299466000	0.697556000	-0.608342000
H	2.713443000	-3.345520000	0.691392000
H	3.612858000	2.765625000	-0.977241000
H	2.083485000	4.795565000	-1.331979000
H	-0.607591000	5.239664000	-1.146730000
Ni	0.041914000	0.226132000	0.087104000
H	-2.643723000	3.796107000	-0.561359000
H	-4.844452000	2.364999000	0.037477000
H	-5.231091000	-0.237580000	0.755331000
H	-3.544303000	-2.315842000	1.106346000
H	-2.014023000	-4.340435000	1.462381000
H	0.677234000	-4.785337000	1.276663000
N	-0.182169000	-0.274284000	-1.874728000
N	-1.121592000	-0.983489000	-2.110562000
N	-2.019917000	-1.669193000	-2.383288000
Cl	0.326961000	0.763851000	2.324572000

NiPorph-Cl-SCN (m3)

C	2.767798000	-1.199229000	0.300756000
C	4.180251000	-1.032829000	0.093395000
C	4.350490000	0.224891000	-0.402847000
C	3.041018000	0.814070000	-0.471590000
N	2.089761000	-0.069782000	-0.053081000
C	2.158425000	-2.357203000	0.764491000
C	2.773360000	2.121766000	-0.853508000
C	1.524024000	2.729170000	-0.808652000
C	1.308023000	4.110510000	-1.125561000
C	-0.014427000	4.359541000	-0.911709000
C	-0.602033000	3.123424000	-0.485201000
N	0.346038000	2.136039000	-0.428068000
C	-1.963927000	2.966993000	-0.249428000
C	-2.588136000	1.761817000	0.047757000
C	-4.008599000	1.576635000	0.162555000
C	-4.206441000	0.244966000	0.372343000
C	-2.901892000	-0.358270000	0.405524000
N	-1.930176000	0.578740000	0.196650000
C	-2.646875000	-1.695408000	0.669121000
C	-1.380340000	-2.258431000	0.791875000
C	-1.159560000	-3.619342000	1.185089000
C	0.189812000	-3.792312000	1.257510000
C	0.786186000	-2.543003000	0.883913000
N	-0.184592000	-1.614976000	0.603728000
H	4.927759000	-1.796935000	0.298311000
H	5.269948000	0.732593000	-0.688744000
H	2.799166000	-3.201516000	1.024616000
H	3.614049000	2.739323000	-1.174639000
H	2.090685000	4.788034000	-1.461477000
H	-0.570109000	5.286371000	-1.040884000
Ni	0.085091000	0.270411000	0.134604000
H	-2.591799000	3.854253000	-0.347055000
H	-4.743260000	2.374801000	0.073992000
H	-5.139990000	-0.298360000	0.507265000
H	-3.501500000	-2.353593000	0.834573000
H	-1.956985000	-4.332812000	1.383560000
H	0.757026000	-4.683274000	1.520007000
S	-0.161232000	-0.412275000	-2.381843000
C	-1.210859000	0.766255000	-2.852264000
N	-1.964149000	1.609524000	-3.195582000
Cl	0.372317000	0.803915000	2.440939000

NiPorph-Cl-ONO2 (m3)

C	2.574677000	-1.477716000	-0.017221000
C	3.990777000	-1.257347000	-0.076174000
C	4.174114000	0.090942000	-0.160066000
C	2.867805000	0.686352000	-0.166828000
N	1.906725000	-0.284585000	-0.077611000
C	2.004645000	-2.745690000	0.106159000

C	2.661194000	2.060972000	-0.286608000
C	1.426408000	2.698913000	-0.350776000
C	1.213561000	4.114448000	-0.513012000
C	-0.137219000	4.294985000	-0.554065000
C	-0.723495000	2.986798000	-0.405331000
N	0.240972000	2.037749000	-0.286105000
C	-2.084787000	2.702615000	-0.353327000
C	-2.648865000	1.446091000	-0.130879000
C	-4.062566000	1.234148000	-0.006930000
C	-4.238003000	-0.091103000	0.260582000
C	-2.931847000	-0.684909000	0.282294000
N	-1.977067000	0.264509000	0.039169000
C	-2.719455000	-2.047758000	0.488696000
C	-1.489127000	-2.694705000	0.451001000
C	-1.278805000	-4.112004000	0.591558000
C	0.061927000	-4.315958000	0.450183000
C	0.647254000	-3.015914000	0.241777000
N	-0.310891000	-2.051215000	0.239895000
H	4.734576000	-2.051543000	-0.048280000
H	5.102278000	0.656084000	-0.222379000
H	2.693989000	-3.592182000	0.125628000
H	3.555330000	2.684770000	-0.346945000
H	2.005521000	4.857658000	-0.587497000
H	-0.701233000	5.219411000	-0.666084000
Ni	-0.037662000	-0.015201000	-0.017870000
H	-2.780994000	3.536473000	-0.463853000
H	-4.809044000	2.019563000	-0.108953000
H	-5.162303000	-0.642640000	0.422984000
H	-3.605788000	-2.658150000	0.673905000
H	-2.066882000	-4.842231000	0.766374000
H	0.621594000	-5.248902000	0.488802000
O	0.035502000	-0.051286000	-2.070266000
N	-0.736042000	-0.753042000	-2.840178000
O	-1.581186000	-1.495904000	-2.357240000
O	-0.555557000	-0.615027000	-4.046496000
Cl	0.124986000	0.318644000	2.316797000

NiPorph-Cl-Me (m1)

C	2.637880000	-1.424381000	0.154356000
C	4.053590000	-1.215985000	0.034281000
C	4.230042000	0.079995000	-0.344391000
C	2.923072000	0.670592000	-0.419024000
N	1.966134000	-0.264152000	-0.122462000
C	2.057982000	-2.649426000	0.435126000
C	2.693364000	2.011808000	-0.672503000
C	1.450298000	2.613805000	-0.571591000
C	1.231566000	4.030088000	-0.655903000
C	-0.088640000	4.239619000	-0.392693000
C	-0.680591000	2.948142000	-0.183019000
N	0.273196000	1.972587000	-0.292726000
C	-2.033526000	2.740095000	0.023490000
C	-2.625055000	1.489357000	0.067950000
C	-4.043559000	1.272646000	0.094428000
C	-4.237794000	-0.072998000	0.010017000
C	-2.935702000	-0.677952000	-0.027849000
N	-1.966988000	0.289738000	0.004682000
C	-2.714676000	-2.044350000	-0.017091000
C	-1.462170000	-2.618751000	0.118539000
C	-1.237909000	-4.024323000	0.305881000
C	0.099895000	-4.184952000	0.503999000
C	0.694290000	-2.881952000	0.397939000
N	-0.273367000	-1.939541000	0.172240000
H	4.799796000	-1.990136000	0.204836000
H	5.154874000	0.620423000	-0.539904000
H	2.717461000	-3.495660000	0.630689000
H	3.552697000	2.645810000	-0.893470000
H	2.014072000	4.757015000	-0.866839000
H	-0.642470000	5.176294000	-0.353438000
Ni	0.002477000	0.023744000	-0.044980000
H	-2.682342000	3.614493000	0.084745000
H	-4.781685000	2.071161000	0.149783000
H	-5.170507000	-0.634537000	-0.005741000
H	-3.581925000	-2.704875000	-0.044910000
H	-2.027907000	-4.773650000	0.302273000
H	0.663573000	-5.098524000	0.686412000
C	-0.112427000	-0.133572000	-2.022718000
H	0.880521000	0.126146000	-2.404713000
H	-0.385932000	-1.172914000	-2.233729000
H	-0.881026000	0.579229000	-2.339202000
Cl	0.184109000	0.319915000	2.343758000

NiPorph-Cl-NO2 (m1)

C	2.627337000	-1.413293000	0.222424000
C	4.040936000	-1.205903000	0.088198000
C	4.213356000	0.069456000	-0.358631000
C	2.906888000	0.656388000	-0.453435000
N	1.954379000	-0.269358000	-0.118143000
C	2.047430000	-2.626844000	0.547257000
C	2.674018000	1.989292000	-0.746908000
C	1.436720000	2.598071000	-0.618034000
C	1.221103000	4.015018000	-0.690996000
C	-0.085517000	4.231529000	-0.370176000
C	-0.679176000	2.944030000	-0.146866000
N	0.263218000	1.963357000	-0.309523000
C	-2.026743000	2.740827000	0.093505000
C	-2.621540000	1.491397000	0.118587000
C	-4.039359000	1.274711000	0.151051000
C	-4.237113000	-0.067809000	0.027125000
C	-2.937624000	-0.675398000	-0.031042000
N	-1.968896000	0.292256000	0.007256000
C	-2.716642000	-2.042033000	-0.031208000
C	-1.466342000	-2.614205000	0.135222000
C	-1.242074000	-4.013579000	0.359955000
C	0.089117000	-4.164458000	0.608392000
C	0.685449000	-2.864751000	0.487724000
N	-0.278543000	-1.934656000	0.199984000
H	4.789962000	-1.969248000	0.291603000
H	5.136266000	0.600022000	-0.587042000
H	2.704752000	-3.462515000	0.789092000
H	3.530123000	2.615897000	-0.998466000
H	1.998280000	4.737904000	-0.933038000
H	-0.631310000	5.171560000	-0.306935000
Ni	-0.000585000	0.020674000	-0.004972000
H	-2.668938000	3.616566000	0.191349000
H	-4.775991000	2.072325000	0.231710000
H	-5.171142000	-0.626633000	0.000589000

H	-3.582985000	-2.703014000	-0.068521000
H	-2.028968000	-4.765959000	0.347049000
H	0.648551000	-5.071977000	0.829399000
N	-0.100831000	-0.237333000	-2.049934000
O	0.535677000	-1.155894000	-2.497510000
O	-0.787812000	0.551903000	-2.645394000
Cl	0.156734000	0.336227000	2.281244000

NiPorph-Cl-ONO (m1)

C	2.614530000	-1.450643000	-0.193757000
C	4.033275000	-1.235705000	-0.167044000
C	4.225060000	0.106198000	-0.028570000
C	2.923462000	0.711616000	-0.018557000
N	1.955093000	-0.251964000	-0.132984000
C	2.019462000	-2.702321000	-0.177256000
C	2.696757000	2.077775000	-0.000691000
C	1.448363000	2.651511000	-0.177570000
C	1.216110000	4.057742000	-0.343114000
C	-0.112735000	4.208096000	-0.603998000
C	-0.696177000	2.896920000	-0.551550000
N	0.274060000	1.958756000	-0.321602000
C	-2.057926000	2.649825000	-0.624586000
C	-2.641859000	1.429986000	-0.326338000
C	-4.053326000	1.232361000	-0.158755000
C	-4.221563000	-0.041263000	0.294977000
C	-2.917173000	-0.635951000	0.356679000
N	-1.966558000	0.269705000	-0.044741000
C	-2.677127000	-1.962857000	0.669539000
C	-1.441717000	-2.572526000	0.527231000
C	-1.215086000	-3.984472000	0.641126000
C	0.089306000	-4.201836000	0.311183000
C	0.669559000	-2.916074000	0.045529000
N	-0.286070000	-1.938389000	0.145948000
H	4.772387000	-2.033139000	-0.222471000
H	5.157764000	0.663792000	0.038401000
H	2.670659000	-3.575260000	-0.235908000
H	3.558160000	2.740458000	0.090469000
H	1.994696000	4.816400000	-0.284166000
H	-0.677386000	5.119600000	-0.792749000
Ni	-0.003421000	0.019115000	-0.004092000
H	-2.718298000	3.494203000	-0.826003000
H	-4.802923000	1.999414000	-0.344860000
H	-5.142450000	-0.564827000	0.546460000
H	-3.523617000	-2.581204000	0.970007000
H	-1.983243000	-4.706135000	0.913764000
H	0.640915000	-5.139552000	0.269515000
O	-0.023227000	-0.173446000	-2.144914000
N	-0.974141000	-0.880162000	-2.517810000
O	-1.183786000	-1.118183000	-3.687039000
Cl	0.106854000	0.322473000	2.266985000

NiPorph (m1)

C	2.633216000	-1.429400000	0.021587000
C	4.049581000	-1.217323000	-0.058096000
C	4.233028000	0.122391000	-0.211251000
C	2.925986000	0.714962000	-0.225290000
N	1.945738000	-0.241137000	-0.082485000
C	2.057547000	-2.675960000	0.184291000
C	2.706545000	2.072053000	-0.368367000
C	1.450769000	2.649909000	-0.384290000
C	1.231302000	4.059506000	-0.532732000
C	-0.116218000	4.247741000	-0.498705000
C	-0.706086000	2.950661000	-0.329987000
N	0.259151000	1.971160000	-0.260812000
C	-2.069577000	2.738573000	-0.250548000
C	-2.645250000	1.492050000	-0.087534000
C	-4.061651000	1.279839000	-0.008899000
C	-4.244950000	-0.059508000	0.147636000
C	-2.937808000	-0.651765000	0.164283000
N	-1.957619000	0.304169000	0.019905000
C	-2.718225000	-2.008486000	0.310669000
C	-1.462465000	-2.586391000	0.326249000
C	-1.242983000	-3.995947000	0.474966000
C	0.104413000	-4.184531000	0.437879000
C	0.694188000	-2.887718000	0.266914000
N	-0.270967000	-1.908000000	0.199523000
H	4.791784000	-2.012047000	0.001281000
H	5.160634000	0.684810000	-0.308141000
H	2.717953000	-3.541883000	0.250832000
H	3.574079000	2.725427000	-0.473313000
H	2.026564000	4.794688000	-0.646361000
H	-0.685409000	5.172877000	-0.577674000
Ni	-0.005921000	0.031547000	-0.030801000
H	-2.730068000	3.604273000	-0.319146000
H	-4.803953000	2.074271000	-0.070812000
H	-5.172538000	-0.621827000	0.245304000
H	-3.585697000	-2.661676000	0.417278000
H	-2.038140000	-4.730897000	0.590770000
H	0.673537000	-5.109773000	0.516082000

NiPorph-OH (m2)

C	2.639241000	-1.403024000	0.142120000
C	4.051631000	-1.200059000	-0.013385000
C	4.224254000	0.104715000	-0.362411000
C	2.918610000	0.701511000	-0.389228000
N	1.958447000	-0.229671000	-0.075642000
C	2.069875000	-2.631613000	0.434292000
C	2.699659000	2.051274000	-0.609293000
C	1.463496000	2.666689000	-0.498370000
C	1.255604000	4.085237000	-0.581697000
C	-0.073410000	4.297744000	-0.367666000
C	-0.670361000	3.004139000	-0.183973000
N	0.281779000	2.020669000	-0.245991000
C	-2.030612000	2.782026000	-0.049108000
C	-2.609659000	1.524287000	-0.006627000
C	-4.026580000	1.302055000	-0.003250000
C	-4.213358000	-0.047075000	-0.028131000
C	-2.909213000	-0.646335000	-0.014568000
N	-1.936739000	0.324883000	-0.000351000
C	-2.695597000	-2.011704000	0.078495000
C	-1.449421000	-2.593402000	0.245332000
C	-1.234134000	-3.994636000	0.477452000
C	0.109105000	-4.161578000	0.631902000
C	0.705091000	-2.864844000	0.464321000
N	-0.259281000	-1.915200000	0.253440000
H	4.799577000	-1.979336000	0.123936000

H	5.147318000	0.645465000	-0.566170000
H	2.740051000	-3.476869000	0.597520000
H	3.565310000	2.677262000	-0.830803000
H	2.045139000	4.812126000	-0.766104000
H	-0.625026000	5.236525000	-0.347021000
Ni	0.000685000	0.030329000	-0.170922000
H	-2.693998000	3.647857000	-0.030995000
H	-4.769526000	2.098146000	0.009003000
H	-5.144236000	-0.612100000	-0.030109000
H	-3.569728000	-2.664446000	0.075078000
H	-2.028079000	-4.738583000	0.524206000
H	0.670175000	-5.074988000	0.824091000
O	-0.104048000	-0.190311000	-2.126219000
H	-0.689359000	0.530591000	-2.396956000

NiPorph-Cl (m2)

C	2.644780000	-1.424625000	0.094093000
C	4.059477000	-1.210802000	-0.001746000
C	4.239728000	0.121132000	-0.218286000
C	2.933823000	0.714475000	-0.245883000
N	1.965862000	-0.237251000	-0.036265000
C	2.068623000	-2.668925000	0.275537000
C	2.710426000	2.065658000	-0.436476000
C	1.458261000	2.652349000	-0.418854000
C	1.236273000	4.062005000	-0.559921000
C	-0.107740000	4.257838000	-0.462083000
C	-0.699365000	2.965004000	-0.270972000
N	0.268891000	1.991980000	-0.231779000
C	-2.060279000	2.751644000	-0.153041000
C	-2.637175000	1.504095000	0.002475000
C	-4.053057000	1.287763000	0.070766000
C	-4.238734000	-0.057402000	0.168715000
C	-2.933964000	-0.654115000	0.172492000
N	-1.961017000	0.312019000	0.090516000
C	-2.715070000	-2.016043000	0.266003000
C	-1.460570000	-2.596126000	0.311623000
C	-1.237696000	-4.006135000	0.447148000
C	0.111660000	-4.188139000	0.468054000
C	0.704700000	-2.888467000	0.334963000
N	-0.266094000	-1.920793000	0.261351000
H	4.803421000	-2.000525000	0.088607000
H	5.165987000	0.679504000	-0.344275000
H	2.730678000	-3.532194000	0.352715000
H	3.575830000	2.710927000	-0.592370000
H	2.029374000	4.793977000	-0.702765000
H	-0.674256000	5.186415000	-0.509629000
Ni	-0.008790000	0.015833000	-0.140017000
H	-2.721377000	3.617592000	-0.203378000
H	-4.794219000	2.084705000	0.038601000
H	-5.167202000	-0.621706000	0.239457000
H	-3.584788000	-2.672198000	0.316965000
H	-2.032983000	-4.745923000	0.522062000
H	0.680771000	-5.111765000	0.560544000
Cl	-0.192437000	-0.308448000	-2.479094000

NiPorph-F (m2)

C	2.638250000	-1.409024000	0.227386000
C	4.051332000	-1.199723000	0.092411000
C	4.220514000	0.094485000	-0.297564000
C	2.912461000	0.682030000	-0.359577000
N	1.957199000	-0.244701000	-0.024873000
C	2.059792000	-2.638828000	0.491991000
C	2.676669000	2.018302000	-0.633204000
C	1.434831000	2.620616000	-0.520521000
C	1.214867000	4.034544000	-0.625767000
C	-0.102371000	4.247985000	-0.349353000
C	-0.689334000	2.959216000	-0.115776000
N	0.265634000	1.978424000	-0.200618000
C	-2.042497000	2.749781000	0.088522000
C	-2.629447000	1.496834000	0.136338000
C	-4.046488000	1.277262000	0.173965000
C	-4.237667000	-0.068629000	0.082836000
C	-2.934689000	-0.668670000	0.030132000
N	-1.962495000	0.298833000	0.072946000
C	-2.711184000	-2.034839000	0.028297000
C	-1.458177000	-2.609993000	0.159179000
C	-1.235148000	-4.014548000	0.348791000
C	0.102300000	-4.175348000	0.552521000
C	0.696279000	-2.873208000	0.447482000
N	-0.271084000	-1.924789000	0.226603000
H	4.801926000	-1.970475000	0.259187000
H	5.142683000	0.633725000	-0.508673000
H	2.720307000	-3.487072000	0.675909000
H	3.530194000	2.647350000	-0.888857000
H	1.993689000	4.758965000	-0.858394000
H	-0.655659000	5.185318000	-0.318279000
Ni	-0.009113000	0.010827000	-0.138939000
H	-2.694724000	3.622452000	0.139809000
H	-4.786730000	2.072799000	0.242600000
H	-5.169559000	-0.631752000	0.073964000
H	-3.577668000	-2.696298000	-0.006987000
H	-2.024685000	-4.764432000	0.347300000
H	0.663563000	-5.088843000	0.742755000
F	-0.058229000	-0.142156000	-2.032729000

NiPorph-N3 (m2)

C	2.634487000	-1.295407000	0.021237000
C	4.051387000	-1.112635000	-0.105069000
C	4.266217000	0.228732000	-0.205411000
C	2.975867000	0.856375000	-0.181739000
N	1.988396000	-0.084119000	-0.020850000
C	2.029014000	-2.521070000	0.239391000
C	2.770245000	2.210066000	-0.383846000
C	1.518254000	2.782369000	-0.531860000
C	1.301058000	4.146936000	-0.917572000
C	-0.043011000	4.300600000	-1.076458000
C	-0.644596000	3.040374000	-0.747272000
N	0.320335000	2.121533000	-0.412856000
C	-2.011235000	2.827139000	-0.690897000
C	-2.588597000	1.673659000	-0.188741000
C	-3.994917000	1.514107000	0.044132000
C	-4.159891000	0.294633000	0.628967000
C	-2.858334000	-0.302596000	0.711511000
N	-1.903406000	0.559539000	0.231047000
C	-2.630445000	-1.604091000	1.123684000
C	-1.403857000	-2.238676000	1.024691000

C	-1.203294000	-3.636244000	1.276953000
C	0.092408000	-3.910471000	0.956849000
C	0.687769000	-2.671661000	0.546790000
N	-0.236623000	-1.656310000	0.595080000
H	4.775436000	-1.925721000	-0.092533000
H	5.206104000	0.768608000	-0.309085000
H	2.659381000	-3.411090000	0.241761000
H	3.644937000	2.850512000	-0.503623000
H	2.097389000	4.875439000	-1.060636000
H	-0.604132000	5.187695000	-1.365611000
Ni	0.030161000	0.204860000	-0.021308000
H	-2.670167000	3.643754000	-0.988518000
H	-4.743678000	2.266919000	-0.196674000
H	-5.076099000	-0.189605000	0.962996000
H	-3.481763000	-2.187402000	1.476671000
H	-1.979201000	-4.309113000	1.638161000
H	0.627173000	-4.857476000	1.007772000
N	-0.227831000	-0.330851000	-2.048692000
N	-1.178810000	-1.031953000	-2.242152000
N	-2.100410000	-1.715221000	-2.455182000

NiPorph-SCN (m2)

C	2.720767000	-1.155140000	0.416175000
C	4.125931000	-0.992133000	0.180502000
C	4.283735000	0.215109000	-0.431180000
C	2.979733000	0.803330000	-0.525820000
N	2.032067000	-0.041435000	0.000413000
C	2.147668000	-2.313512000	0.911229000
C	2.737874000	2.082113000	-0.996294000
C	1.511069000	2.715149000	-0.898021000
C	1.296999000	4.100693000	-1.197419000
C	0.005976000	4.379327000	-0.862744000
C	-0.575167000	3.152250000	-0.401535000
N	0.358823000	2.145018000	-0.410673000
C	-1.915975000	3.000170000	-0.096281000
C	-2.511441000	1.775281000	0.147936000
C	-3.928913000	1.582552000	0.243653000
C	-4.137350000	0.238983000	0.326803000
C	-2.843505000	-0.379930000	0.329354000
N	-1.856408000	0.571142000	0.222136000
C	-2.631331000	-1.734420000	0.514657000
C	-1.378963000	-2.293644000	0.703405000
C	-1.157509000	-3.653629000	1.099428000
C	0.184409000	-3.794228000	1.287393000
C	0.782059000	-2.531389000	0.962898000
N	-0.186131000	-1.615127000	0.628157000
H	4.878867000	-1.734515000	0.439855000
H	5.197458000	0.697400000	-0.774581000
H	2.808835000	-3.130065000	1.203804000
H	3.578730000	2.651127000	-1.394742000
H	2.063328000	4.766369000	-1.590857000
H	-0.534347000	5.321826000	-0.933202000
Ni	0.075606000	0.234280000	-0.006517000
H	-2.554834000	3.883279000	-0.131615000
H	-4.657365000	2.391412000	0.222284000
H	-5.075583000	-0.307609000	0.406685000
H	-3.502385000	-2.386214000	0.591084000
H	-1.950262000	-4.387549000	1.233917000
H	0.746958000	-4.673682000	1.596249000
S	-0.165432000	-0.440039000	-2.414996000
C	-1.250232000	0.722101000	-2.860537000
N	-2.026852000	1.546779000	-3.192415000

NiPorph-ONO2 (m2)

C	2.601921000	-1.463973000	-0.156001000
C	4.018316000	-1.243294000	-0.186474000
C	4.209235000	0.100258000	-0.060842000
C	2.906600000	0.700166000	-0.004302000
N	1.935654000	-0.269840000	-0.035161000
C	2.008571000	-2.715261000	-0.147950000
C	2.681643000	2.065967000	-0.021557000
C	1.429110000	2.636306000	-0.177406000
C	1.206063000	4.035476000	-0.402547000
C	-0.125351000	4.186202000	-0.650672000
C	-0.718396000	2.885708000	-0.529121000
N	0.241731000	1.950102000	-0.234214000
C	-2.079764000	2.641694000	-0.597339000
C	-2.654949000	1.429304000	-0.256305000
C	-4.066157000	1.218515000	-0.108230000
C	-4.226297000	-0.051726000	0.358221000
C	-2.915792000	-0.629327000	0.445413000
N	-1.965451000	0.295396000	0.094216000
C	-2.676989000	-1.960213000	0.739321000
C	-1.439996000	-2.566733000	0.606528000
C	-1.228454000	-3.983507000	0.682516000
C	0.071655000	-4.205881000	0.339179000
C	0.662804000	-2.919666000	0.105368000
N	-0.270163000	-1.929208000	0.281064000
H	4.759331000	-2.036886000	-0.267278000
H	5.141215000	0.662509000	-0.032243000
H	2.650569000	-3.589647000	-0.260741000
H	3.547371000	2.728853000	0.003316000
H	1.991966000	4.788997000	-0.388949000
H	-0.684541000	5.094074000	-0.871011000
Ni	-0.029215000	-0.012387000	-0.122978000
H	-2.739563000	3.472483000	-0.850246000
H	-4.821947000	1.975236000	-0.311652000
H	-5.144457000	-0.581984000	0.606061000
H	-3.529481000	-2.587257000	1.003277000
H	-2.001836000	-4.703668000	0.944585000
H	0.613412000	-5.148064000	0.272781000
O	0.031850000	-0.118220000	-2.169226000
N	-0.811186000	-0.802875000	-2.881412000
O	-1.682308000	-1.467225000	-2.335113000
O	-0.664814000	-0.729832000	-4.097358000

NiPorph-Me (m2)

C	2.681192000	-1.452635000	0.023410000
C	4.103097000	-1.239152000	-0.069329000
C	4.287606000	0.103913000	-0.223094000
C	2.976494000	0.703396000	-0.224329000
N	2.014253000	-0.258480000	-0.059946000
C	2.073275000	-2.698606000	0.173026000
C	2.727399000	2.066293000	-0.377808000
C	1.475215000	2.679872000	-0.388655000
C	1.252255000	4.093844000	-0.551556000

C	-0.098689000	4.283897000	-0.512617000
C	-0.693740000	2.984379000	-0.326071000
N	0.281021000	2.025269000	-0.240781000
C	-2.064061000	2.739897000	-0.245983000
C	-2.672670000	1.496609000	-0.077113000
C	-4.095551000	1.279322000	-0.016250000
C	-4.280482000	-0.064960000	0.128178000
C	-2.968655000	-0.661881000	0.157044000
N	-2.005317000	0.306010000	0.046157000
C	-2.718656000	-2.027615000	0.282252000
C	-1.465212000	-2.638035000	0.309510000
C	-1.244500000	-4.056346000	0.433846000
C	0.106571000	-4.245773000	0.403094000
C	0.703363000	-2.941382000	0.259809000
N	-0.268487000	-1.976663000	0.218546000
H	4.854330000	-2.026674000	-0.018729000
H	5.222273000	0.654266000	-0.326718000
H	2.733672000	-3.567671000	0.219362000
H	3.596483000	2.716354000	-0.503619000
H	2.038852000	4.837195000	-0.676330000
H	-0.656357000	5.215981000	-0.598630000
Ni	-0.005698000	0.009566000	-0.170549000
H	-2.725954000	3.605150000	-0.328724000
H	-4.847421000	2.065258000	-0.079533000
H	-5.215999000	-0.617499000	0.210018000
H	-3.588652000	-2.683863000	0.360561000
H	-2.032513000	-4.802202000	0.532279000
H	0.663702000	-5.179726000	0.470364000
C	-0.135131000	-0.140520000	-2.100864000
H	0.885587000	-0.040924000	-2.489896000
H	-0.567480000	-1.126941000	-2.308473000
H	-0.786781000	0.676081000	-2.433914000

NiPorph-NO2 (m2)

C	2.623108000	-1.439961000	0.074155000
C	4.034079000	-1.234997000	-0.077668000
C	4.212873000	0.087739000	-0.346738000
C	2.911307000	0.690126000	-0.342433000
N	1.943906000	-0.254104000	-0.085154000
C	2.056959000	-2.674850000	0.339508000
C	2.697291000	2.046657000	-0.513459000
C	1.456095000	2.652424000	-0.420231000
C	1.240239000	4.068010000	-0.520332000
C	-0.099251000	4.267923000	-0.374552000
C	-0.690860000	2.970296000	-0.204925000
N	0.271269000	1.994423000	-0.213527000
C	-2.052747000	2.744458000	-0.112727000
C	-2.627806000	1.487577000	-0.035524000
C	-4.044046000	1.267388000	-0.008627000
C	-4.232170000	-0.080227000	0.047309000
C	-2.928951000	-0.678733000	0.074740000
N	-1.952792000	0.289817000	0.020836000
C	-2.716196000	-2.039965000	0.207302000
C	-1.465550000	-2.619279000	0.337198000
C	-1.244295000	-4.023215000	0.540634000
C	0.104660000	-4.197675000	0.609949000
C	0.694904000	-2.900813000	0.430351000
N	-0.274404000	-1.942443000	0.291362000
H	4.778058000	-2.024330000	0.016542000
H	5.137566000	0.635927000	-0.520425000
H	2.728502000	-3.527160000	0.451603000
H	3.565703000	2.679977000	-0.699660000
H	2.031131000	4.799958000	-0.676038000
H	-0.661699000	5.200254000	-0.388895000
Ni	-0.008160000	0.009320000	-0.099374000
H	-2.719435000	3.607621000	-0.132168000
H	-4.785517000	2.064501000	-0.031644000
H	-5.162554000	-0.644570000	0.086056000
H	-3.590560000	-2.691099000	0.244641000
H	-2.038162000	-4.764228000	0.620298000
H	0.672707000	-5.115565000	0.753893000
N	-0.103538000	-0.269288000	-2.217392000
O	0.203769000	-1.364818000	-2.650081000
O	-0.459324000	0.675612000	-2.898197000

NiPorph-ONO (m2)

C	2.637019000	-1.445906000	0.044445000
C	4.052263000	-1.229642000	-0.033287000
C	4.235760000	0.111716000	-0.178249000
C	2.930522000	0.707318000	-0.192366000
N	1.961263000	-0.253027000	-0.036442000
C	2.057283000	-2.694007000	0.183676000
C	2.707665000	2.062335000	-0.353951000
C	1.452809000	2.643434000	-0.375553000
C	1.230843000	4.050756000	-0.537314000
C	-0.117479000	4.239670000	-0.513176000
C	-0.711814000	2.946462000	-0.335281000
N	0.258293000	1.978892000	-0.241439000
C	-2.075576000	2.732191000	-0.257811000
C	-2.652299000	1.489495000	-0.067976000
C	-4.066758000	1.274930000	0.025320000
C	-4.249355000	-0.063412000	0.196903000
C	-2.944924000	-0.659972000	0.207172000
N	-1.974698000	0.301254000	0.060320000
C	-2.723828000	-2.017769000	0.344110000
C	-1.469495000	-2.599634000	0.355958000
C	-1.248034000	-4.009650000	0.489393000
C	0.099215000	-4.201308000	0.437142000
C	0.694153000	-2.906237000	0.275912000
N	-0.274530000	-1.932672000	0.241535000
H	4.794750000	-2.024014000	0.022364000
H	5.163383000	0.674256000	-0.270991000
H	2.715409000	-3.562534000	0.225982000
H	3.574685000	2.713512000	-0.471314000
H	2.026586000	4.785122000	-0.650189000
H	-0.685189000	5.164630000	-0.600475000
Ni	-0.016645000	0.004235000	-0.134111000
H	-2.736671000	3.595123000	-0.344984000
H	-4.809045000	2.069071000	-0.035488000
H	-5.176323000	-0.623236000	0.310287000
H	-3.591531000	-2.670372000	0.447230000
H	-2.043188000	-4.743762000	0.607707000
H	0.666521000	-5.128256000	0.503165000
O	-0.008094000	-0.168021000	-2.155625000
N	-0.927830000	-0.961801000	-2.605577000
O	-0.932747000	-1.071288000	-3.817291000

CuPorph (m2)

C	2.670409000	-1.441372000	0.019733000
C	4.092061000	-1.224785000	-0.057036000
C	4.276183000	0.118361000	-0.211282000
C	2.965273000	0.716262000	-0.229524000
N	2.006139000	-0.249754000	-0.089108000
C	2.066480000	-2.686309000	0.184430000
C	2.718193000	2.079706000	-0.371887000
C	1.462361000	2.683352000	-0.389603000
C	1.238243000	4.097723000	-0.539256000
C	-0.112684000	4.286547000	-0.503896000
C	-0.708151000	2.986108000	-0.332060000
N	0.266954000	2.027199000	-0.266044000
C	-2.078395000	2.749250000	-0.247626000
C	-2.682301000	1.504315000	-0.082830000
C	-4.103978000	1.287685000	-0.006549000
C	-4.288073000	-0.055357000	0.148649000
C	-2.977130000	-0.653147000	0.167784000
N	-2.018001000	0.312792000	0.026895000
C	-2.730017000	-2.016500000	0.311015000
C	-1.474189000	-2.620154000	0.328600000
C	-1.250064000	-4.034504000	0.478374000
C	0.100822000	-4.223434000	0.442049000
C	0.696269000	-2.923062000	0.269667000
N	-0.278826000	-1.964076000	0.204231000
H	4.842979000	-2.011684000	0.004519000
H	5.210364000	0.671134000	-0.305028000
H	2.726570000	-3.553639000	0.253321000
H	3.585715000	2.734898000	-0.476704000
H	2.025929000	4.841193000	-0.655568000
H	-0.672182000	5.217885000	-0.584931000
Cu	-0.005818000	0.031489000	-0.031013000
H	-2.738531000	3.616500000	-0.317085000
H	-4.854924000	2.074487000	-0.069008000
H	-5.222258000	-0.608110000	0.242473000
H	-3.597503000	-2.671635000	0.416494000
H	-2.037725000	-4.777905000	0.595281000
H	0.660293000	-5.154818000	0.522732000

CuPorph-OH (m3)

C	2.684584000	-1.434473000	0.091045000
C	4.104810000	-1.221148000	-0.015769000
C	4.286159000	0.116981000	-0.208948000
C	2.975286000	0.715179000	-0.218280000
N	2.021404000	-0.243086000	-0.013404000
C	2.078025000	-2.677926000	0.256860000
C	2.726042000	2.072279000	-0.410804000
C	1.474883000	2.684854000	-0.433342000
C	1.255063000	4.096987000	-0.605796000
C	-0.093735000	4.295562000	-0.546361000
C	-0.694596000	3.002837000	-0.342556000
N	0.278068000	2.042717000	-0.267514000
C	-2.063358000	2.766069000	-0.239877000
C	-2.667982000	1.522895000	-0.064771000
C	-4.089469000	1.304225000	0.000828000
C	-4.273532000	-0.040194000	0.141169000
C	-2.962780000	-0.637320000	0.167134000
N	-2.004068000	0.332462000	0.057167000
C	-2.716002000	-2.001754000	0.298410000
C	-1.462955000	-2.610218000	0.339414000
C	-1.244398000	-4.025901000	0.484184000
C	0.106567000	-4.216489000	0.477886000
C	0.707206000	-2.915973000	0.327429000
N	-0.264675000	-1.955640000	0.258219000
H	4.855473000	-2.007749000	0.048753000
H	5.217922000	0.666500000	-0.337339000
H	2.736847000	-3.546492000	0.320494000
H	3.594160000	2.718255000	-0.558695000
H	2.045016000	4.833791000	-0.744722000
H	-0.648713000	5.229249000	-0.628258000
Cu	0.001360000	0.016807000	-0.186846000
H	-2.724304000	3.631974000	-0.315802000
H	-4.839823000	2.091521000	-0.058536000
H	-5.207285000	-0.594937000	0.224297000
H	-3.586299000	-2.657100000	0.374415000
H	-2.035763000	-4.768206000	0.579237000
H	0.663336000	-5.148619000	0.565499000
O	-0.088562000	-0.078101000	-2.189420000
H	-0.531234000	0.747940000	-2.439202000

CuPorph-Cl (m3)

C	2.681825000	-1.434359000	0.068559000
C	4.102098000	-1.221651000	-0.029625000
C	4.284906000	0.120442000	-0.190779000
C	2.973928000	0.716825000	-0.193352000
N	2.017125000	-0.243272000	-0.016544000
C	2.073036000	-2.680093000	0.223383000
C	2.720437000	2.078104000	-0.358245000
C	1.467403000	2.690874000	-0.379752000
C	1.246162000	4.104502000	-0.538373000
C	-0.103991000	4.294873000	-0.495023000
C	-0.696949000	2.995487000	-0.310244000
N	0.275668000	2.038483000	-0.235089000
C	-2.066252000	2.750406000	-0.211461000
C	-2.673735000	1.508471000	-0.026930000
C	-4.095700000	1.291919000	0.037058000
C	-4.278910000	-0.050674000	0.192224000
C	-2.966652000	-0.643806000	0.226113000
N	-2.007679000	0.323238000	0.110425000
C	-2.714209000	-2.009026000	0.353137000
C	-1.461401000	-2.622054000	0.374427000
C	-1.242620000	-4.041143000	0.475474000
C	0.107858000	-4.230893000	0.439008000
C	0.703718000	-2.925426000	0.316050000
N	-0.266726000	-1.962957000	0.297181000
H	4.851443000	-2.010104000	0.022251000
H	5.216564000	0.673449000	-0.301281000
H	2.733556000	-3.549193000	0.260088000
H	3.588390000	2.728931000	-0.485632000
H	2.034764000	4.845424000	-0.660020000
H	-0.664295000	5.225381000	-0.573496000
Cu	-0.010498000	0.007985000	-0.187964000
H	-2.729293000	3.614479000	-0.293607000
H	-4.845621000	2.078237000	-0.034469000
H	-5.211525000	-0.606587000	0.276946000

H	-3.583832000	-2.665911000	0.426455000
H	-2.032843000	-4.785595000	0.559926000
H	0.667126000	-5.164113000	0.486463000
Cl	-0.251526000	-0.327320000	-2.547426000

CuPorph-F (m3)

C	2.673674000	-1.441714000	0.071521000
C	4.093477000	-1.228897000	-0.032946000
C	4.276261000	0.113995000	-0.186663000
C	2.965832000	0.711683000	-0.178078000
N	2.009721000	-0.248741000	0.001973000
C	2.065965000	-2.688360000	0.218648000
C	2.714498000	2.073967000	-0.335451000
C	1.462095000	2.687338000	-0.353631000
C	1.240742000	4.101692000	-0.505058000
C	-0.109640000	4.291550000	-0.464291000
C	-0.703132000	2.990897000	-0.289782000
N	0.269889000	2.034735000	-0.213375000
C	-2.072449000	2.744886000	-0.199365000
C	-2.678589000	1.500576000	-0.028329000
C	-4.099736000	1.285155000	0.048554000
C	-4.283465000	-0.058228000	0.197046000
C	-2.972239000	-0.653685000	0.213855000
N	-2.012740000	0.312642000	0.092776000
C	-2.721913000	-2.019221000	0.342440000
C	-1.469216000	-2.631598000	0.365264000
C	-1.249273000	-4.049826000	0.474476000
C	0.101390000	-4.239005000	0.438384000
C	0.696704000	-2.934080000	0.308824000
N	-0.274513000	-1.972475000	0.286273000
H	4.842608000	-2.018243000	0.008446000
H	5.207723000	0.666811000	-0.300067000
H	2.726675000	-3.557422000	0.250841000
H	3.583018000	2.723911000	-0.462987000
H	2.029335000	4.843580000	-0.621169000
H	-0.669811000	5.222424000	-0.539802000
Cu	-0.014032000	-0.002270000	-0.212897000
H	-2.735874000	3.609263000	-0.273918000
H	-4.849199000	2.073214000	-0.007819000
H	-5.216051000	-0.612835000	0.290511000
H	-3.591938000	-2.674876000	0.420639000
H	-2.038894000	-4.794339000	0.564109000
H	0.661025000	-5.171727000	0.491585000
F	-0.072385000	-0.244358000	-2.119723000

CuPorph-N3 (m3)

C	2.708764000	-1.259732000	0.208125000
C	4.124674000	-1.092618000	0.010089000
C	4.318463000	0.210923000	-0.342891000
C	3.019325000	0.831296000	-0.356467000
N	2.058310000	-0.077721000	-0.010230000
C	2.094186000	-2.461496000	0.562244000
C	2.777817000	2.166082000	-0.679351000
C	1.534415000	2.795967000	-0.700868000
C	1.320199000	4.177007000	-1.044880000
C	-0.023148000	4.398197000	-0.953869000
C	-0.619875000	3.149965000	-0.556044000
N	0.343424000	2.194085000	-0.398812000
C	-1.988825000	2.949490000	-0.369777000
C	-2.603741000	1.756086000	-0.002472000
C	-4.024877000	1.569915000	0.130807000
C	-4.218472000	0.261158000	0.462670000
C	-2.913200000	-0.342923000	0.533600000
N	-1.947456000	0.584200000	0.266579000
C	-2.674774000	-1.692420000	0.803117000
C	-1.427912000	-2.317893000	0.862931000
C	-1.221865000	-3.715699000	1.138355000
C	0.123415000	-3.931882000	1.071752000
C	0.729620000	-2.665594000	0.751287000
N	-0.231404000	-1.696756000	0.645271000
H	4.863826000	-1.883437000	0.128781000
H	5.250937000	0.723213000	-0.575738000
H	2.745721000	-3.328656000	0.689810000
H	3.645098000	2.775539000	-0.942547000
H	2.109346000	4.875411000	-1.319555000
H	-0.576794000	5.317483000	-1.139635000
Cu	0.045424000	0.226598000	0.013289000
H	-2.641341000	3.806982000	-0.547677000
H	-4.768117000	2.351204000	-0.020558000
H	-5.154666000	-0.265604000	0.641881000
H	-3.549893000	-2.319340000	0.987910000
H	-2.017295000	-4.428503000	1.350274000
H	0.672077000	-4.861036000	1.219283000
N	-0.357765000	-0.448689000	-2.077030000
N	-1.321249000	-1.143067000	-2.174450000
N	-2.266646000	-1.825602000	-2.284101000

CuPorph-SCN (m3)

C	2.755510000	-1.221661000	0.261587000
C	4.170549000	-1.043396000	0.070587000
C	4.353879000	0.258145000	-0.295159000
C	3.049148000	0.865611000	-0.322256000
N	2.094498000	-0.047504000	0.028862000
C	2.145751000	-2.424526000	0.618254000
C	2.795815000	2.194648000	-0.664759000
C	1.548600000	2.814903000	-0.706368000
C	1.324554000	4.182827000	-1.093596000
C	-0.020970000	4.394737000	-1.017026000
C	-0.607653000	3.154859000	-0.581627000
N	0.360785000	2.212224000	-0.389556000
C	-1.975597000	2.942457000	-0.403900000
C	-2.582220000	1.749998000	-0.005040000
C	-4.001889000	1.558594000	0.130534000
C	-4.186822000	0.253493000	0.481411000
C	-2.878495000	-0.342438000	0.555317000
N	-1.917396000	0.590672000	0.270582000
C	-2.629141000	-1.679484000	0.856196000
C	-1.379334000	-2.300330000	0.901768000
C	-1.164818000	-3.689723000	1.208751000
C	0.181730000	-3.900328000	1.143187000
C	0.778892000	-2.637527000	0.795539000
N	-0.186319000	-1.678605000	0.661769000
H	4.916053000	-1.826217000	0.200886000
H	5.282492000	0.776725000	-0.528891000
H	2.802244000	-3.284940000	0.765182000
H	3.659318000	2.806314000	-0.935381000

H	2.109253000	4.878422000	-1.387051000
H	-0.582226000	5.302006000	-1.235104000
Cu	0.075833000	0.246772000	0.045888000
H	-2.636082000	3.788305000	-0.606538000
H	-4.749259000	2.333736000	-0.030780000
H	-5.119273000	-0.276378000	0.670234000
H	-3.498105000	-2.307164000	1.065920000
H	-1.955768000	-4.400989000	1.441455000
H	0.736609000	-4.821939000	1.312454000
S	-0.185861000	-0.419680000	-2.520897000
C	-1.228062000	0.775144000	-2.948982000
N	-1.979320000	1.633666000	-3.264862000

CuPorph-ONO2 (m3)

C	2.661243000	-1.473573000	0.074536000
C	4.080169000	-1.260530000	-0.038418000
C	4.259345000	0.081354000	-0.207806000
C	2.947208000	0.674992000	-0.199313000
N	1.993963000	-0.285007000	-0.009330000
C	2.053378000	-2.719257000	0.241868000
C	2.690509000	2.035596000	-0.372303000
C	1.437681000	2.651691000	-0.384631000
C	1.218787000	4.062862000	-0.567293000
C	-0.131569000	4.254829000	-0.527952000
C	-0.723982000	2.958427000	-0.321971000
N	0.246898000	2.003575000	-0.224896000
C	-2.094856000	2.712085000	-0.233460000
C	-2.699971000	1.470201000	-0.036108000
C	-4.120310000	1.254927000	0.055611000
C	-4.298755000	-0.084567000	0.244075000
C	-2.985530000	-0.674855000	0.264908000
N	-2.031117000	0.287226000	0.104640000
C	-2.729861000	-2.036994000	0.423282000
C	-1.476787000	-2.650813000	0.441824000
C	-1.259777000	-4.066331000	0.583674000
C	0.089612000	-4.261055000	0.525181000
C	0.684056000	-2.961003000	0.352038000
N	-0.284998000	-1.998263000	0.316971000
H	4.830796000	-2.048023000	0.008836000
H	5.188898000	0.635128000	-0.331226000
H	2.713653000	-3.588311000	0.283757000
H	3.557566000	2.684305000	-0.516487000
H	2.007963000	4.800424000	-0.704424000
H	-0.691486000	5.183661000	-0.626327000
Cu	-0.038194000	-0.034476000	-0.167170000
H	-2.759224000	3.573830000	-0.328541000
H	-4.871783000	2.039615000	-0.016844000
H	-5.228925000	-0.638710000	0.361082000
H	-3.598084000	-2.690428000	0.536451000
H	-2.049576000	-4.805438000	0.708442000
H	0.648201000	-5.193481000	0.591676000
O	0.081704000	-0.056956000	-2.295733000
N	-0.773646000	-0.703256000	-3.002771000
O	-1.613012000	-1.414234000	-2.456130000
O	-0.698278000	-0.568591000	-4.224198000

CuPorph-Me (m3)

C	2.670699000	-1.454021000	0.036544000
C	4.091716000	-1.238144000	-0.056001000
C	4.274840000	0.104007000	-0.220011000
C	2.964160000	0.703051000	-0.227656000
N	2.007353000	-0.261971000	-0.067798000
C	2.066588000	-2.698419000	0.206809000
C	2.715326000	2.065097000	-0.382284000
C	1.460638000	2.672179000	-0.387330000
C	1.235327000	4.085135000	-0.550766000
C	-0.115099000	4.275471000	-0.501716000
C	-0.709790000	2.977420000	-0.308450000
N	0.266467000	2.020637000	-0.237698000
C	-2.079664000	2.738174000	-0.219056000
C	-2.683498000	1.494080000	-0.045030000
C	-4.105196000	1.276258000	0.029353000
C	-4.288873000	-0.066925000	0.184532000
C	-2.977763000	-0.664435000	0.206317000
N	-2.019673000	0.302918000	0.069956000
C	-2.729608000	-2.028217000	0.347325000
C	-1.474170000	-2.633073000	0.369936000
C	-1.249136000	-4.047541000	0.519000000
C	0.101761000	-4.236319000	0.478893000
C	0.697019000	-2.935913000	0.304291000
N	-0.278667000	-1.977763000	0.246924000
H	4.842935000	-2.024950000	0.002457000
H	5.208339000	0.655546000	-0.326619000
H	2.726906000	-3.566011000	0.269346000
H	3.582161000	2.717519000	-0.507865000
H	2.021872000	4.826760000	-0.684805000
H	-0.674681000	5.206301000	-0.587535000
Cu	-0.008950000	0.013602000	-0.051513000
H	-2.741123000	3.603903000	-0.294619000
H	-4.856574000	2.062473000	-0.034666000
H	-5.222980000	-0.619971000	0.277042000
H	-3.597489000	-2.683417000	0.448889000
H	-2.036271000	-4.791052000	0.638307000
H	0.661423000	-5.167672000	0.558176000
C	-0.178608000	-0.213633000	-2.527049000
H	0.885341000	-0.253776000	-2.774847000
H	-0.742085000	-1.148817000	-2.582765000
H	-0.713456000	0.704942000	-2.782188000

CuPorph-NO2 (m3)

C	2.673584000	-1.458133000	0.061537000
C	4.092863000	-1.247284000	-0.051369000
C	4.278340000	0.093420000	-0.223501000
C	2.970932000	0.696963000	-0.214660000
N	2.012037000	-0.263977000	-0.039095000
C	2.067992000	-2.699019000	0.244950000
C	2.724658000	2.059199000	-0.364631000
C	1.471276000	2.666982000	-0.356833000
C	1.245219000	4.076019000	-0.544674000
C	-0.104938000	4.265992000	-0.497255000
C	-0.697856000	2.971475000	-0.281266000
N	0.278308000	2.017726000	-0.185103000
C	-2.068242000	2.733275000	-0.207836000
C	-2.674348000	1.489884000	-0.044871000
C	-4.095992000	1.272912000	0.012205000
C	-4.282429000	-0.069618000	0.168282000

C	-2.973043000	-0.667552000	0.207784000
N	-2.011759000	0.298476000	0.078407000
C	-2.727023000	-2.029427000	0.360360000
C	-1.471415000	-2.631258000	0.400804000
C	-1.248015000	-4.046578000	0.537086000
C	0.102888000	-4.234881000	0.506470000
C	0.698886000	-2.933280000	0.350461000
N	-0.275197000	-1.973261000	0.302403000
H	4.841321000	-2.037065000	-0.000924000
H	5.211549000	0.641748000	-0.346390000
H	2.726373000	-3.568331000	0.301930000
H	3.590738000	2.708748000	-0.507709000
H	2.031609000	4.814013000	-0.696989000
H	-0.666522000	5.193392000	-0.603093000
Cu	0.001913000	0.014994000	0.016341000
H	-2.728403000	3.598539000	-0.296905000
H	-4.845275000	2.059998000	-0.061946000
H	-5.217192000	-0.622734000	0.251275000
H	-3.595414000	-2.685577000	0.448606000
H	-2.037017000	-4.790616000	0.638179000
H	0.662886000	-5.166573000	0.576695000
N	-0.113548000	-0.241172000	-2.380805000
O	0.286574000	-1.297773000	-2.796966000
O	-0.563427000	0.695731000	-2.988449000

ZnPorph (m1)

C	2.697791000	-1.447972000	0.021639000
C	4.122715000	-1.230307000	-0.058457000
C	4.307725000	0.115259000	-0.212040000
C	2.994550000	0.716859000	-0.225515000
N	2.044873000	-0.253423000	-0.082674000
C	2.072431000	-2.691280000	0.183202000
C	2.726435000	2.084193000	-0.367457000
C	1.469558000	2.703763000	-0.385329000
C	1.242377000	4.119805000	-0.544557000
C	-0.110814000	4.308770000	-0.509360000
C	-0.708883000	3.007175000	-0.328623000
N	0.271815000	2.057780000	-0.256046000
C	-2.084343000	2.754373000	-0.245435000
C	-2.709643000	1.511042000	-0.083785000
C	-4.134565000	1.293406000	-0.003152000
C	-4.319529000	-0.052174000	0.150231000
C	-3.006314000	-0.653766000	0.163331000
N	-2.056706000	0.316504000	0.020079000
C	-2.738321000	-2.021121000	0.305185000
C	-1.481508000	-2.640808000	0.322875000
C	-1.254272000	-4.056832000	0.482125000
C	0.098949000	-4.245709000	0.447153000
C	0.696951000	-2.944097000	0.266168000
N	-0.283809000	-1.994814000	0.193456000
H	4.879379000	-2.012531000	-0.001855000
H	5.246542000	0.660336000	-0.308199000
H	2.730898000	-3.560782000	0.253998000
H	3.592555000	2.741733000	-0.478039000
H	2.024444000	4.868406000	-0.668439000
H	-0.663831000	5.243561000	-0.598880000
Zn	-0.005806000	0.031473000	-0.031439000
H	-2.742817000	3.623884000	-0.316088000
H	-4.891217000	2.075682000	-0.059235000
H	-5.258307000	-0.597259000	0.246738000
H	-3.604528000	-2.678547000	0.415784000
H	-2.036293000	-4.805482000	0.605997000
H	0.652009000	-5.180458000	0.536849000

ZnPorph-OH (m2)

C	2.697183000	-1.440787000	0.066200000
C	4.122933000	-1.226780000	-0.001376000
C	4.306013000	0.115763000	-0.171021000
C	2.989705000	0.709238000	-0.203545000
N	2.035978000	-0.252678000	-0.046896000
C	2.072341000	-2.685449000	0.226150000
C	2.725481000	2.076771000	-0.368111000
C	1.475307000	2.709197000	-0.402915000
C	1.257133000	4.125188000	-0.581460000
C	-0.093954000	4.319359000	-0.538071000
C	-0.689807000	3.019468000	-0.333965000
N	0.282726000	2.065627000	-0.247358000
C	-2.062286000	2.758401000	-0.231936000
C	-2.686440000	1.513165000	-0.058713000
C	-4.111629000	1.304660000	0.034821000
C	-4.298283000	-0.039902000	0.184757000
C	-2.984359000	-0.638921000	0.181788000
N	-2.027792000	0.323774000	0.042834000
C	-2.720521000	-2.009110000	0.315272000
C	-1.468555000	-2.642263000	0.331918000
C	-1.250498000	-4.054007000	0.535074000
C	0.102118000	-4.245210000	0.511791000
C	0.695928000	-2.947811000	0.293046000
N	-0.275899000	-1.998251000	0.185255000
H	4.877505000	-2.008881000	0.073141000
H	5.242066000	0.665269000	-0.264326000
H	2.734946000	-3.550393000	0.315212000
H	3.597620000	2.724785000	-0.489802000
H	2.042438000	4.867619000	-0.717968000
H	-0.646612000	5.253392000	-0.633060000
Zn	-0.009198000	-0.020336000	-0.469491000
H	-2.726477000	3.623760000	-0.304146000
H	-4.863579000	2.091666000	-0.009476000
H	-5.234946000	-0.586279000	0.289542000
H	-3.591397000	-2.659695000	0.432231000
H	-2.035695000	-4.794079000	0.683941000
H	0.656559000	-5.174355000	0.638669000
O	-0.009765000	0.017100000	-2.409681000
H	-0.513611000	0.798739000	-2.672126000

ZnPorph-Cl (m2)

C	2.697142000	-1.438052000	0.039826000
C	4.122806000	-1.224960000	-0.014022000
C	4.307712000	0.119484000	-0.169923000
C	2.991848000	0.710853000	-0.210100000
N	2.037044000	-0.252150000	-0.076180000
C	2.071143000	-2.684468000	0.204509000
C	2.723444000	2.080763000	-0.355686000
C	1.470090000	2.713329000	-0.375850000
C	1.251896000	4.132980000	-0.509946000
C	-0.100558000	4.321880000	-0.468025000

C	-0.691120000	3.014609000	-0.307379000
N	0.280786000	2.061898000	-0.251342000
C	-2.066102000	2.749804000	-0.208172000
C	-2.692679000	1.505555000	-0.033152000
C	-4.117862000	1.295956000	0.053566000
C	-4.302017000	-0.048254000	0.209438000
C	-2.986204000	-0.642742000	0.216896000
N	-2.032202000	0.319619000	0.078934000
C	-2.717325000	-2.013634000	0.350872000
C	-1.464239000	-2.647567000	0.359807000
C	-1.245910000	-4.064732000	0.522402000
C	0.106155000	-4.254741000	0.481596000
C	0.696212000	-2.950638000	0.292356000
N	-0.275011000	-1.998174000	0.226782000
H	4.876169000	-2.007448000	0.065097000
H	5.243848000	0.670860000	-0.246777000
H	2.735956000	-3.549292000	0.278235000
H	3.594100000	2.734220000	-0.457598000
H	2.036671000	4.880348000	-0.617578000
H	-0.656685000	5.256060000	-0.533529000
Zn	-0.017815000	-0.012454000	-0.451460000
H	-2.729531000	3.616323000	-0.274667000
H	-4.870837000	2.080993000	-0.000947000
H	-5.237317000	-0.597098000	0.311115000
H	-3.587405000	-2.666839000	0.459669000
H	-2.030242000	-4.808623000	0.654470000
H	0.662512000	-5.186639000	0.573014000
Cl	-0.242720000	-0.303699000	-2.740395000

ZnPorph-F (m2)

C	2.685957000	-1.437482000	0.059150000
C	4.112517000	-1.227894000	-0.004366000
C	4.298802000	0.116753000	-0.154143000
C	2.982527000	0.709509000	-0.182487000
N	2.026328000	-0.250328000	-0.039510000
C	2.059920000	-2.686044000	0.208036000
C	2.715662000	2.079197000	-0.333526000
C	1.461695000	2.710959000	-0.369567000
C	1.245471000	4.134144000	-0.463409000
C	-0.107449000	4.322433000	-0.426955000
C	-0.699034000	3.010502000	-0.315076000
N	0.271593000	2.055703000	-0.284526000
C	-2.073702000	2.743924000	-0.215568000
C	-2.699177000	1.496816000	-0.053941000
C	-4.120384000	1.293864000	0.089440000
C	-4.304820000	-0.050967000	0.241474000
C	-2.992974000	-0.651098000	0.191056000
N	-2.040417000	0.306081000	0.013790000
C	-2.726680000	-2.022566000	0.331323000
C	-1.474264000	-2.657193000	0.339917000
C	-1.254944000	-4.073171000	0.516355000
C	0.097429000	-4.260869000	0.478427000
C	0.684359000	-2.955731000	0.281949000
N	-0.287247000	-2.006092000	0.203770000
H	4.864728000	-2.012903000	0.060866000
H	5.235179000	0.666600000	-0.239599000
H	2.725515000	-3.550760000	0.277771000
H	3.587063000	2.733536000	-0.423451000
H	2.031627000	4.884457000	-0.535740000
H	-0.663000000	5.258566000	-0.463657000
Zn	-0.022207000	-0.015812000	-0.548182000
H	-2.736650000	3.612866000	-0.247352000
H	-4.870131000	2.083912000	0.080105000
H	-5.237222000	-0.595558000	0.384652000
H	-3.597191000	-2.671175000	0.462430000
H	-2.038141000	-4.817277000	0.654313000
H	0.655656000	-5.190675000	0.580189000
F	-0.010853000	-0.339809000	-2.393145000

ZnPorph-N3 (m2)

C	2.736460000	-1.260715000	0.181332000
C	4.159053000	-1.085407000	0.023445000
C	4.353901000	0.220973000	-0.327934000
C	3.047977000	0.831097000	-0.381772000
N	2.090025000	-0.088381000	-0.070577000
C	2.100785000	-2.456918000	0.554083000
C	2.788084000	2.176072000	-0.685085000
C	1.544345000	2.825548000	-0.710976000
C	1.339474000	4.224514000	-0.992511000
C	-0.006210000	4.447144000	-0.902733000
C	-0.610447000	3.181230000	-0.567700000
N	0.348278000	2.220359000	-0.454591000
C	-1.985124000	2.961790000	-0.376539000
C	-2.620008000	1.764650000	-0.023223000
C	-4.043164000	1.588394000	0.137785000
C	-4.237462000	0.278656000	0.472326000
C	-2.929790000	-0.331316000	0.516957000
N	-1.970606000	0.588516000	0.223325000
C	-2.671404000	-1.682030000	0.806574000
C	-1.423495000	-2.323782000	0.874034000
C	-1.218691000	-3.721616000	1.168892000
C	0.127503000	-3.939816000	1.097932000
C	0.731714000	-2.673963000	0.756218000
N	-0.229458000	-1.710546000	0.643424000
H	4.905499000	-1.864696000	0.170414000
H	5.293097000	0.735160000	-0.527855000
H	2.752899000	-3.321976000	0.700551000
H	3.659430000	2.795237000	-0.914325000
H	2.129653000	4.937955000	-1.222319000
H	-0.548361000	5.380953000	-1.045720000
Zn	0.035747000	0.178640000	-0.251175000
H	-2.635613000	3.827868000	-0.523907000
H	-4.789057000	2.371122000	0.006867000
H	-5.175454000	-0.237630000	0.672314000
H	-3.545824000	-2.306439000	1.008812000
H	-2.009791000	-4.435144000	1.395088000
H	0.671476000	-4.870172000	1.256135000
N	-0.444195000	-0.606378000	-2.155551000
N	-1.417964000	-1.291597000	-2.229779000
N	-2.367559000	-1.965221000	-2.321128000

ZnPorph-SCN (m2)

C	2.778073000	-1.219153000	0.260817000
C	4.198882000	-1.031864000	0.100709000
C	4.381826000	0.275224000	-0.253131000
C	3.070772000	0.874629000	-0.302919000

N	2.121156000	-0.049518000	0.017480000
C	2.150563000	-2.424890000	0.613564000
C	2.797808000	2.213100000	-0.626634000
C	1.547832000	2.848260000	-0.676988000
C	1.327719000	4.231231000	-1.021411000
C	-0.020859000	4.439627000	-0.952901000
C	-0.610587000	3.181106000	-0.567683000
N	0.358375000	2.238619000	-0.400564000
C	-1.983767000	2.944461000	-0.398216000
C	-2.609032000	1.741047000	-0.030794000
C	-4.030153000	1.552775000	0.122567000
C	-4.213679000	0.241969000	0.460846000
C	-2.902557000	-0.357633000	0.508789000
N	-1.949972000	0.575970000	0.213922000
C	-2.634025000	-1.698791000	0.815295000
C	-1.382996000	-2.336095000	0.873151000
C	-1.163445000	-3.720905000	1.210757000
C	0.186585000	-3.925812000	1.154498000
C	0.778241000	-2.663555000	0.783326000
N	-0.193566000	-1.720649000	0.624779000
H	4.951454000	-1.806133000	0.242148000
H	5.315471000	0.796243000	-0.460616000
H	2.811460000	-3.280271000	0.776728000
H	3.663361000	2.834822000	-0.870482000
H	2.110823000	4.942516000	-1.279912000
H	-0.575291000	5.356927000	-1.145844000
Zn	0.056837000	0.192082000	-0.167999000
H	-2.645295000	3.796470000	-0.575552000
H	-4.781889000	2.328874000	-0.012729000
H	-5.147502000	-0.281669000	0.660751000
H	-3.502947000	-2.324320000	1.036340000
H	-1.946937000	-4.435438000	1.458907000
H	0.740623000	-4.843116000	1.348946000
S	-0.145091000	-0.426401000	-2.636852000
C	-1.227526000	0.755778000	-3.029309000
N	-2.003839000	1.595918000	-3.325194000

ZnPorph-ONO2 (m2)

C	2.678392000	-1.474807000	0.055439000
C	4.102915000	-1.259116000	-0.010511000
C	4.283521000	0.084646000	-0.178861000
C	2.966243000	0.673040000	-0.213241000
N	2.015145000	-0.291181000	-0.066396000
C	2.055707000	-2.721364000	0.236444000
C	2.695780000	2.042410000	-0.368064000
C	1.443303000	2.678379000	-0.382544000
C	1.226561000	4.095897000	-0.542600000
C	-0.125818000	4.286870000	-0.502351000
C	-0.716841000	2.983029000	-0.316123000
N	0.254124000	2.031833000	-0.241264000
C	-2.092470000	2.717487000	-0.219609000
C	-2.716284000	1.473319000	-0.029574000
C	-4.139341000	1.262160000	0.074842000
C	-4.318691000	-0.080124000	0.257709000
C	-3.002971000	-0.672378000	0.259744000
N	-2.054494000	0.289267000	0.091326000
C	-2.731339000	-2.040985000	0.415084000
C	-1.479351000	-2.677928000	0.422744000
C	-1.260936000	-4.089876000	0.619795000
C	0.090923000	-4.284492000	0.566645000
C	0.681028000	-2.987600000	0.337785000
N	-0.290888000	-2.037916000	0.255615000
H	4.858513000	-2.039377000	0.068806000
H	5.217890000	0.637277000	-0.267178000
H	2.722350000	-3.583936000	0.318898000
H	3.566471000	2.693455000	-0.484843000
H	2.011677000	4.840175000	-0.667524000
H	-0.681033000	5.220019000	-0.587408000
Zn	-0.052021000	-0.061052000	-0.366634000
H	-2.757953000	3.581245000	-0.299512000
H	-4.894345000	2.044824000	0.015796000
H	-5.251846000	-0.628105000	0.381124000
H	-3.600546000	-2.690067000	0.553108000
H	-2.044588000	-4.828388000	0.782144000
H	0.646189000	-5.214957000	0.676751000
O	0.077162000	-0.037285000	-2.426041000
N	-0.790079000	-0.722322000	-3.093532000
O	-1.627231000	-1.389284000	-2.481993000
O	-0.732916000	-0.672219000	-4.318477000

ZnPorph-Me (m2)

C	2.724485000	-1.461346000	0.142588000
C	4.148233000	-1.246155000	0.038970000
C	4.333212000	0.099127000	-0.116796000
C	3.021167000	0.703079000	-0.108227000
N	2.071885000	-0.265587000	0.048353000
C	2.100716000	-2.704358000	0.312137000
C	2.753417000	2.070764000	-0.246242000
C	1.497368000	2.692165000	-0.248575000
C	1.268505000	4.104497000	-0.436060000
C	-0.084894000	4.292408000	-0.404160000
C	-0.681153000	2.994099000	-0.195864000
N	0.301076000	2.048758000	-0.095630000
C	-2.056985000	2.737268000	-0.137297000
C	-2.681625000	1.492596000	0.016654000
C	-4.107626000	1.268415000	0.041613000
C	-4.291835000	-0.077326000	0.194291000
C	-2.976954000	-0.671894000	0.262509000
N	-2.027067000	0.302477000	0.154051000
C	-2.709281000	-2.038490000	0.412905000
C	-1.452011000	-2.655689000	0.459532000
C	-1.225846000	-4.075261000	0.587442000
C	0.127641000	-4.263097000	0.557909000
C	0.726247000	-2.957103000	0.411387000
N	-0.253501000	-2.005826000	0.364185000
H	4.904055000	-2.030070000	0.082124000
H	5.271246000	0.642537000	-0.228653000
H	2.759366000	-3.574971000	0.365229000
H	3.618708000	2.726111000	-0.374669000
H	2.049509000	4.850411000	-0.580886000
H	-0.639360000	5.223653000	-0.518375000
Zn	0.032024000	0.031073000	0.184757000
H	-2.716868000	3.602855000	-0.236961000
H	-4.865200000	2.046147000	-0.051933000
H	-5.230712000	-0.627667000	0.252393000
H	-3.576756000	-2.698913000	0.490795000

H	-2.008988000	-4.827124000	0.681613000
H	0.680417000	-5.200067000	0.622627000
C	-0.196307000	-0.269992000	-2.899011000
H	0.888007000	-0.320257000	-3.016269000
H	-0.774027000	-1.195044000	-2.850474000
H	-0.711716000	0.671169000	-3.100569000

ZnPorph-NO2 (m2)

C	2.689466000	-1.444759000	0.064950000
C	4.109701000	-1.240840000	-0.087243000
C	4.300173000	0.104131000	-0.234759000
C	2.994572000	0.716767000	-0.175530000
N	2.042591000	-0.243785000	0.016116000
C	2.060855000	-2.687488000	0.218440000
C	2.731478000	2.083991000	-0.320242000
C	1.477595000	2.709254000	-0.326175000
C	1.250169000	4.117733000	-0.537132000
C	-0.102851000	4.306471000	-0.506804000
C	-0.697653000	3.012241000	-0.276184000
N	0.282479000	2.067245000	-0.160678000
C	-2.072545000	2.755478000	-0.206663000
C	-2.698660000	1.514812000	-0.029966000
C	-4.124551000	1.297214000	0.003590000
C	-4.312542000	-0.045999000	0.168609000
C	-2.999864000	-0.643042000	0.234963000
N	-2.044809000	0.325830000	0.117815000
C	-2.739286000	-2.011240000	0.380822000
C	-1.486404000	-2.635948000	0.410406000
C	-1.264091000	-4.058501000	0.494193000
C	0.088210000	-4.248768000	0.444088000
C	0.688119000	-2.940674000	0.333404000
N	-0.287671000	-1.984828000	0.328556000
H	4.858272000	-2.032548000	-0.085768000
H	5.236365000	0.642026000	-0.381018000
H	2.716478000	-3.561885000	0.233744000
H	3.597693000	2.733066000	-0.471392000
H	2.031912000	4.859735000	-0.695491000
H	-0.658361000	5.234835000	-0.636200000
Zn	0.008257000	0.049035000	0.151860000
H	-2.732795000	3.619556000	-0.315905000
H	-4.878445000	2.077611000	-0.094086000
H	-5.251717000	-0.594371000	0.235053000
H	-3.610306000	-2.667207000	0.454495000
H	-2.049207000	-4.810045000	0.570079000
H	0.640185000	-5.187764000	0.471288000
N	-0.038460000	-0.380554000	-2.346558000
O	0.675982000	-1.261023000	-2.754205000
O	-0.791033000	0.332985000	-2.962445000

1.3.2. BOX complexes

ScBOX-Cl2 (m2)

C	-0.166815000	-0.490075000	0.445201000
O	0.563629000	-1.669129000	0.383298000
C	-1.686266000	-2.103385000	0.003574000
C	-0.280501000	-2.639185000	-0.236491000
H	-0.090891000	-3.622524000	0.215962000
C	-0.192909000	1.979373000	0.373750000
O	0.436607000	3.067089000	0.803142000
C	-1.835917000	3.509697000	0.377935000
C	-0.505258000	4.163939000	0.762049000
H	-0.505561000	4.646463000	1.746267000
C	-2.526350000	4.150407000	-0.802638000
H	-1.886464000	4.121006000	-1.696555000
H	-3.470106000	3.638970000	-1.040586000
H	-2.766461000	5.200272000	-0.585952000
C	-2.283817000	-2.567204000	1.319866000
H	-1.595484000	-2.361082000	2.153764000
H	-3.229012000	-2.048241000	1.532071000
H	-2.484832000	-3.648412000	1.308606000
N	-1.444294000	2.114207000	0.092955000
N	-1.436061000	-0.650616000	-0.010022000
Sc	-2.891318000	0.622009000	-0.691522000
Cl	-4.444721000	1.026817000	1.172724000
H	-2.358965000	-2.372884000	-0.828346000
H	-0.043645000	-2.694465000	-1.314715000
H	-2.526790000	3.486214000	1.238059000
H	-0.145328000	4.885191000	0.013716000
C	0.656257000	0.749703000	0.200391000
C	1.847646000	0.780224000	1.158290000
H	1.522766000	0.834035000	2.206839000
H	2.434419000	-0.138147000	1.033697000
H	2.504897000	1.634934000	0.954953000
C	1.165121000	0.763645000	-1.259764000
H	1.809636000	-0.111158000	-1.423143000
H	0.328142000	0.719989000	-1.972457000
H	1.756941000	1.666753000	-1.471165000
Cl	-2.326959000	0.874517000	-3.068716000

ScBOX-Cl2-OH (m1)

C	-0.072626000	-0.499303000	0.043929000
O	0.704738000	-1.581859000	0.068839000
C	-1.564331000	-2.157648000	-0.135287000
C	-0.162683000	-2.737778000	0.070547000
H	-0.044338000	-3.243987000	1.039835000
C	-0.171265000	2.011649000	0.227876000
O	0.502263000	3.122003000	0.545693000
C	-1.803072000	3.524354000	0.378402000
C	-0.444616000	4.213656000	0.503440000
H	-0.319791000	4.809672000	1.414445000
C	-2.726551000	4.102340000	-0.666813000
H	-2.271960000	4.063837000	-1.667338000
H	-3.674209000	3.542841000	-0.699917000
H	-2.969274000	5.148772000	-0.437666000
C	-2.597835000	-2.630107000	0.858663000
H	-2.293354000	-2.394486000	1.888032000
H	-3.573885000	-2.158420000	0.673728000
H	-2.735166000	-3.717269000	0.780433000
N	-1.435022000	2.129714000	0.070266000
N	-1.336237000	-0.701414000	-0.043891000
Sc	-3.098154000	0.648845000	-0.359019000
Cl	-5.451184000	0.072882000	-0.726374000
Cl	-3.395757000	0.892798000	2.136596000
H	-1.923299000	-2.361473000	-1.159844000
H	0.169256000	-3.414499000	-0.725057000
H	-2.324783000	3.497900000	1.351348000

H	-0.198761000	4.839734000	-0.367973000
C	0.700853000	0.795123000	0.059439000
C	1.736685000	0.749408000	1.190713000
H	1.253421000	0.669789000	2.174028000
H	2.401998000	-0.111700000	1.062256000
H	2.350624000	1.656664000	1.180945000
C	1.407627000	0.924061000	-1.304978000
H	2.102224000	0.088060000	-1.455093000
H	0.678676000	0.927145000	-2.127160000
H	1.981532000	1.858835000	-1.343140000
O	-2.676188000	0.873021000	-2.214585000
H	-1.874852000	1.256999000	-2.582720000

ScBOX-Cl3 (m1)

C	-0.069056000	-0.501157000	0.051294000
O	0.702851000	-1.582954000	0.031128000
C	-1.571275000	-2.157467000	-0.113195000
C	-0.165062000	-2.739791000	0.041683000
H	-0.017682000	-3.258680000	0.999928000
C	-0.164766000	2.006790000	0.241126000
O	0.510815000	3.121553000	0.517573000
C	-1.800121000	3.528274000	0.357568000
C	-0.438830000	4.212620000	0.507752000
H	-0.316542000	4.776675000	1.439381000
C	-2.666909000	4.075225000	-0.752730000
H	-2.157605000	3.999049000	-1.723545000
H	-3.615745000	3.521801000	-0.822670000
H	-2.911569000	5.129929000	-0.568075000
C	-2.574231000	-2.655756000	0.898626000
H	-2.245542000	-2.435600000	1.923962000
H	-3.560533000	-2.194404000	0.746261000
H	-2.701374000	-3.742686000	0.803651000
N	-1.433779000	2.120057000	0.111657000
N	-1.337317000	-0.701940000	0.004854000
Sc	-3.075771000	0.638394000	-0.250005000
Cl	-5.388531000	0.042825000	-0.665170000
Cl	-3.372880000	0.806030000	2.163816000
H	-1.956998000	-2.334703000	-1.132715000
H	0.142793000	-3.403866000	-0.773556000
H	-2.360494000	3.550388000	1.308446000
H	-0.188503000	4.867400000	-0.339777000
C	0.706152000	0.790102000	0.078484000
C	1.723702000	0.733919000	1.227014000
H	1.223920000	0.645526000	2.201238000
H	2.389855000	-0.126942000	1.100188000
H	2.338134000	1.640954000	1.234377000
C	1.435622000	0.925173000	-1.273615000
H	2.119334000	0.080045000	-1.420872000
H	0.721530000	0.951473000	-2.107867000
H	2.024800000	1.850663000	-1.287766000
Cl	-2.525723000	0.815101000	-2.627875000

ScBOX-Cl2-F (m1)

C	-0.024726000	-0.516320000	0.117607000
O	0.734731000	-1.575581000	0.372192000
C	-1.490988000	-2.204155000	-0.054944000
C	-0.132607000	-2.733779000	0.410262000
H	-0.145640000	-3.103786000	1.445858000
C	-0.145821000	1.981710000	0.293722000
O	0.505118000	3.086594000	0.660630000
C	-1.812002000	3.454354000	0.492155000
C	-0.466944000	4.159346000	0.682553000
H	-0.363768000	4.692000000	1.634727000
C	-2.701271000	4.054949000	-0.572036000
H	-2.198854000	4.063636000	-1.550158000
H	-3.637150000	3.482927000	-0.670359000
H	-2.973151000	5.087788000	-0.315841000
C	-2.650563000	-2.584569000	0.834591000
H	-2.499133000	-2.219384000	1.859528000
H	-3.596755000	-2.166834000	0.460613000
H	-2.760043000	-3.677234000	0.865878000
N	-1.412752000	2.077547000	0.149346000
N	-1.270957000	-0.742260000	-0.105690000
Sc	-2.982209000	0.610947000	-0.574273000
Cl	-5.227336000	0.039887000	-1.397697000
Cl	-3.690108000	0.778702000	1.800692000
H	-1.700900000	-2.531988000	-1.087965000
H	0.301627000	-3.503498000	-0.237285000
H	-2.370544000	3.392573000	1.441832000
H	-0.221803000	4.849444000	-0.138767000
C	0.732792000	0.787032000	0.025193000
C	1.925159000	0.781752000	0.983768000
H	1.605420000	0.683662000	2.029829000
H	2.599276000	-0.048874000	0.749007000
H	2.490532000	1.714528000	0.883402000
C	1.220275000	0.912079000	-1.435162000
H	1.870332000	0.065200000	-1.691222000
H	0.370893000	0.933842000	-2.130730000
H	1.799709000	1.836972000	-1.554604000
F	-2.317197000	1.055529000	-2.286577000

ScBOX-Cl2-N3 (m1)

C	-0.010261000	-0.547253000	0.169696000
O	0.767603000	-1.613474000	0.314742000
C	-1.478773000	-2.234915000	0.023508000
C	-0.095995000	-2.773460000	0.391231000
H	-0.044280000	-3.157742000	1.420643000
C	-0.143744000	1.957359000	0.274556000
O	0.505103000	3.082762000	0.557793000
C	-1.813028000	3.447844000	0.353416000
C	-0.470619000	4.153121000	0.573102000
H	-0.384864000	4.672728000	1.534458000
C	-2.620417000	3.971398000	-0.812527000
H	-2.036790000	3.944170000	-1.744136000
H	-3.532258000	3.372981000	-0.962055000
H	-2.932360000	5.008938000	-0.632279000
C	-2.592369000	-2.668935000	0.945207000
H	-2.394577000	-2.355969000	1.979544000
H	-3.558677000	-2.243553000	0.636196000
H	-2.693910000	-3.762571000	0.927932000
N	-1.415156000	2.042589000	0.138136000
N	-1.270141000	-0.770169000	0.040565000
Sc	-3.014445000	0.544673000	-0.342063000
Cl	-5.244650000	-0.009328000	-1.163365000
Cl	-3.630821000	0.697506000	2.014839000
H	-1.736551000	-2.505205000	-1.016320000

H	0.295415000	-3.532824000	-0.294257000
H	-2.430383000	3.473159000	1.267376000
H	-0.211027000	4.853040000	-0.233776000
C	0.743135000	0.755954000	0.082356000
C	1.882554000	0.774775000	1.105977000
H	1.504096000	0.710599000	2.134968000
H	2.561326000	-0.067205000	0.932520000
H	2.459520000	1.700712000	1.007197000
C	1.316566000	0.846369000	-1.349215000
H	1.992529000	0.002556000	-1.537824000
H	0.516000000	0.834581000	-2.101642000
H	1.889512000	1.775993000	-1.460332000
N	-2.358018000	0.742046000	-2.373055000
N	-2.852099000	0.628005000	-3.455613000
N	-3.305369000	0.524185000	-4.518640000

ScBOX-Cl2-SCN (m1)

C	-0.185029000	-0.569514000	-0.183896000
O	0.485810000	-1.629338000	-0.601565000
C	-1.760536000	-2.167268000	-0.174193000
C	-0.482195000	-2.685309000	-0.831990000
H	-0.089374000	-3.607026000	-0.386176000
C	-0.169489000	1.912357000	-0.098537000
O	0.543808000	3.002529000	-0.339755000
C	-1.712605000	3.531109000	0.001067000
C	-0.393792000	4.092068000	-0.520158000
H	-0.015871000	4.962501000	0.025978000
C	-2.923871000	3.883240000	-0.826385000
H	-2.778673000	3.618603000	-1.883150000
H	-3.819728000	3.356586000	-0.463262000
H	-3.128867000	4.960446000	-0.765753000
C	-2.107306000	-2.817006000	1.147342000
H	-1.271205000	-2.737861000	1.858919000
H	-2.984675000	-2.335135000	1.601915000
H	-2.340249000	-3.880984000	1.006058000
N	-1.439574000	2.074906000	0.050816000
N	-1.444344000	-0.735771000	0.036772000
Sc	-3.100352000	0.649738000	0.414316000
Cl	-5.435182000	0.119090000	0.210600000
Cl	-2.836064000	0.743810000	2.783261000
H	-2.614963000	-2.239533000	-0.866360000
H	-0.566267000	-2.817292000	-1.919260000
H	-1.882028000	3.847008000	1.046654000
H	-0.424377000	4.323207000	-1.596191000
C	0.636936000	0.656328000	0.113827000
C	0.969090000	0.600306000	1.623391000
H	0.055387000	0.600436000	2.233541000
H	1.543074000	-0.309821000	1.843251000
H	1.582908000	1.466655000	1.902774000
C	1.924406000	0.675388000	-0.705426000
H	2.514755000	-0.223646000	-0.495390000
H	1.710562000	0.713662000	-1.780476000
H	2.531331000	1.546552000	-0.435339000
S	-3.249217000	0.493952000	-2.362135000
C	-1.645045000	0.655149000	-2.716673000
N	-0.491541000	0.763627000	-2.946412000

ScBOX-Cl2-ONO2 (m1)

C	-0.089354000	-0.511222000	-0.128504000
O	0.640736000	-1.573300000	-0.432996000
C	-1.643237000	-2.121759000	-0.270251000
C	-0.249072000	-2.713625000	-0.495365000
H	0.056109000	-3.418627000	0.290708000
C	-0.148822000	1.999497000	-0.020913000
O	0.547069000	3.128327000	-0.114941000
C	-1.742799000	3.563070000	0.115987000
C	-0.411137000	4.204093000	-0.261540000
H	-0.100128000	5.029647000	0.387344000
C	-2.914998000	3.939032000	-0.759361000
H	-2.720339000	3.682736000	-1.809514000
H	-3.823814000	3.406328000	-0.435675000
H	-3.122352000	5.014682000	-0.686412000
C	-2.439850000	-2.762726000	0.841126000
H	-1.904696000	-2.703642000	1.799714000
H	-3.417042000	-2.272310000	0.962266000
H	-2.628536000	-3.821219000	0.616649000
N	-1.424777000	2.117955000	0.059814000
N	-1.351392000	-0.703339000	0.019268000
Sc	-3.088240000	0.631016000	0.108076000
Cl	-5.394960000	0.065659000	-0.291406000
Cl	-3.053982000	0.750736000	2.512987000
H	-2.226776000	-2.142152000	-1.207378000
H	-0.115184000	-3.188492000	-1.474130000
H	-1.990300000	3.779254000	1.170971000
H	-0.378195000	4.535695000	-1.310572000
C	0.701839000	0.757149000	0.057709000
C	1.295077000	0.708561000	1.482234000
H	0.498965000	0.675078000	2.238137000
H	1.924932000	-0.183255000	1.594215000
H	1.916465000	1.594722000	1.662189000
C	1.818658000	0.830098000	-0.989368000
H	2.485188000	-0.034381000	-0.897700000
H	1.408106000	0.846915000	-2.008458000
H	2.414430000	1.736951000	-0.838918000
O	-1.043477000	-0.258340000	-2.893388000
N	-1.660242000	0.771780000	-2.641862000
O	-2.780771000	0.666594000	-1.983241000
O	-1.289909000	1.887488000	-2.985700000

ScBOX-Cl2-Me (m1)

C	-0.050231000	-0.489600000	-0.068909000
O	0.717526000	-1.572863000	-0.170506000
C	-1.569470000	-2.126574000	-0.249173000
C	-0.155975000	-2.719352000	-0.288297000
H	0.058491000	-3.392804000	0.553092000
C	-0.120697000	2.023182000	0.113718000
O	0.580760000	3.150387000	0.273032000
C	-1.721770000	3.568116000	0.255934000
C	-0.356840000	4.245306000	0.165484000
H	-0.146354000	4.957493000	0.970820000
C	-2.765432000	4.078570000	-0.707104000
H	-2.423696000	3.994037000	-1.748932000
H	-3.705471000	3.514307000	-0.603095000
H	-2.994048000	5.133853000	-0.505966000
C	-2.440858000	-2.640612000	0.875803000
H	-1.959394000	-2.483297000	1.851903000

H	-3.414253000	-2.127421000	0.890173000
H	-2.630814000	-3.715574000	0.753222000
N	-1.392423000	2.145271000	0.030959000
N	-1.318214000	-0.677773000	-0.108208000
Sc	-3.102863000	0.672453000	-0.142495000
Cl	-5.480913000	0.178925000	-0.369527000
Cl	-3.063582000	0.864417000	2.354895000
H	-2.086358000	-2.283248000	-1.211370000
H	0.089446000	-3.234342000	-1.225050000
H	-2.117421000	3.629399000	1.286305000
H	-0.188622000	4.740099000	-0.803747000
C	0.732074000	0.782871000	0.122140000
C	1.404624000	0.697756000	1.507579000
H	0.652735000	0.633349000	2.306527000
H	2.048117000	-0.188963000	1.559930000
H	2.024581000	1.585355000	1.681487000
C	1.796711000	0.894595000	-0.978499000
H	2.467429000	0.028100000	-0.948551000
H	1.340264000	0.946151000	-1.976545000
H	2.397917000	1.798663000	-0.826883000
C	-2.805078000	0.720468000	-2.381359000
H	-1.776283000	0.918975000	-2.738291000
H	-3.101708000	-0.264188000	-2.796221000
H	-3.466227000	1.459678000	-2.875590000

ScBOX-Cl2-ONO (m1)

C	-0.123341000	-0.478287000	-0.150602000
O	0.596921000	-1.532608000	-0.516876000
C	-1.663744000	-2.102596000	-0.224021000
C	-0.273529000	-2.687778000	-0.478406000
H	0.073979000	-3.341268000	0.335146000
C	-0.162337000	2.031342000	0.036300000
O	0.540490000	3.160899000	0.014226000
C	-1.770122000	3.584449000	0.160599000
C	-0.403602000	4.257887000	-0.001183000
H	-0.139942000	4.943381000	0.812275000
C	-2.792101000	3.945410000	-0.892787000
H	-2.433466000	3.681640000	-1.898071000
H	-3.739295000	3.412349000	-0.722447000
H	-3.011063000	5.021329000	-0.869625000
C	-2.443278000	-2.761891000	0.888698000
H	-1.893425000	-2.712752000	1.839576000
H	-3.418712000	-2.274269000	1.031344000
H	-2.632361000	-3.818251000	0.654522000
N	-1.436863000	2.148772000	0.108170000
N	-1.369374000	-0.688641000	0.082397000
Sc	-3.109101000	0.651179000	0.155753000
Cl	-5.425451000	0.076346000	-0.179905000
Cl	-3.053140000	0.775557000	2.577841000
H	-2.264765000	-2.113500000	-1.151994000
H	-0.172199000	-3.220188000	-1.430630000
H	-2.186477000	3.781853000	1.163423000
H	-0.289218000	4.782568000	-0.960660000
C	0.680568000	0.783044000	0.036846000
C	1.336868000	0.681084000	1.430307000
H	0.572352000	0.631078000	2.217664000
H	1.960706000	-0.220045000	1.487633000
H	1.975324000	1.554497000	1.612833000
C	1.749698000	0.892502000	-1.056053000
H	2.409862000	0.018638000	-1.034434000
H	1.295538000	0.962578000	-2.054146000
H	2.363939000	1.784775000	-0.891817000
N	-1.648571000	0.589603000	-2.524159000
O	-2.797468000	0.661844000	-1.896140000
O	-1.505959000	1.390980000	-3.416329000

TiBOX-Cl3 (m2)

C	-0.049649000	-0.490511000	0.042874000
O	0.693706000	-1.590887000	-0.019291000
C	-1.600688000	-2.105515000	-0.150196000
C	-0.207400000	-2.723211000	-0.012725000
H	-0.069742000	-3.254164000	0.940400000
C	-0.133329000	2.007358000	0.205589000
O	0.530116000	3.141884000	0.412554000
C	-1.790218000	3.509681000	0.354341000
C	-0.436460000	4.219535000	0.344341000
H	-0.264208000	4.883333000	1.198439000
C	-2.790501000	3.987851000	-0.670372000
H	-2.379701000	3.921390000	-1.687328000
H	-3.704252000	3.371908000	-0.631601000
H	-3.082860000	5.028181000	-0.475618000
C	-2.615591000	-2.619523000	0.841602000
H	-2.278030000	-2.457286000	1.874701000
H	-3.587594000	-2.121040000	0.713322000
H	-2.774875000	-3.696337000	0.694862000
N	-1.409257000	2.104010000	0.115735000
N	-1.323110000	-0.662186000	0.019992000
Ti	-2.961659000	0.661338000	-0.178207000
Cl	-5.238077000	0.151921000	-0.527520000
Cl	-3.223552000	0.860133000	2.215472000
H	-1.987684000	-2.229828000	-1.177384000
H	0.074922000	-3.388469000	-0.836020000
H	-2.246000000	3.540694000	1.359634000
H	-0.250562000	4.775472000	-0.586734000
C	0.737946000	0.788107000	0.090144000
C	1.693038000	0.735046000	1.292436000
H	1.139733000	0.653181000	2.238069000
H	2.359697000	-0.130817000	1.203768000
H	2.309232000	1.640959000	1.327015000
C	1.532554000	0.902314000	-1.224533000
H	2.203223000	0.041488000	-1.334752000
H	0.857748000	0.937097000	-2.090562000
H	2.141305000	1.814741000	-1.216040000
Cl	-2.445548000	0.786169000	-2.535830000

TiBOX-Cl3-OH (m1)

C	-0.242452000	-0.427966000	-0.165969000
O	0.513782000	-1.462969000	-0.526163000
C	-1.709275000	-2.099889000	-0.298810000
C	-0.396126000	-2.506904000	-0.948027000
H	0.000093000	-3.468419000	-0.603306000
C	-0.282095000	2.051016000	0.165597000
O	0.449020000	3.159559000	0.227486000
C	-1.821453000	3.657046000	-0.059412000
C	-0.467274000	4.275056000	0.282254000
H	-0.417170000	4.697218000	1.295060000

C	-2.317155000	3.992493000	-1.448831000
H	-1.579952000	3.698921000	-2.211997000
H	-3.266948000	3.488052000	-1.666559000
H	-2.480275000	5.075583000	-1.536953000
C	-1.933749000	-2.769655000	1.041542000
H	-1.052248000	-2.645759000	1.689699000
H	-2.800041000	-2.345495000	1.561666000
H	-2.104022000	-3.846107000	0.901593000
N	-1.555966000	2.199922000	0.071218000
N	-1.509956000	-0.636928000	-0.104184000
Ti	-3.230896000	0.727989000	0.149293000
Cl	-4.751600000	-0.994237000	0.258247000
Cl	-2.617412000	0.587735000	2.460510000
H	-2.563267000	-2.263394000	-0.970317000
H	-0.424132000	-2.496903000	-2.047150000
H	-2.578482000	3.936072000	0.685861000
H	-0.125598000	5.029031000	-0.437419000
C	0.540832000	0.794071000	0.223020000
C	1.007219000	0.583078000	1.679585000
H	0.151371000	0.476859000	2.359423000
H	1.627154000	-0.320781000	1.744158000
H	1.614326000	1.438807000	2.002055000
C	1.758463000	0.954476000	-0.696579000
H	2.393125000	0.063384000	-0.643092000
H	1.458441000	1.102011000	-1.743094000
H	2.357425000	1.816951000	-0.384992000
Cl	-4.752749000	2.416393000	0.557634000
O	-3.199171000	0.856056000	-1.648397000
H	-2.436692000	1.042171000	-2.214127000

TiBOX-Cl4 (m1)

C	-0.212871000	-0.500459000	0.000799000
O	0.587201000	-1.557404000	-0.034090000
C	-1.655529000	-2.211121000	0.024705000
C	-0.245097000	-2.717725000	-0.255838000
H	0.089707000	-3.510657000	0.423170000
C	-0.353020000	1.978822000	0.308347000
O	0.290981000	3.057839000	0.734807000
C	-1.913703000	3.570064000	0.206074000
C	-0.717882000	4.062391000	1.003755000
H	-0.886069000	4.075653000	2.089797000
C	-2.003579000	4.193594000	-1.170921000
H	-1.049487000	4.084190000	-1.709672000
H	-2.790578000	3.725754000	-1.773266000
H	-2.224760000	5.266281000	-1.085019000
C	-2.202893000	-2.651892000	1.363752000
H	-1.535414000	-2.347010000	2.183660000
H	-3.196319000	-2.228885000	1.553026000
H	-2.287698000	-3.747159000	1.383051000
N	-1.613360000	2.114420000	0.079073000
N	-1.480930000	-0.729443000	-0.028122000
Ti	-3.179190000	0.647828000	-0.301036000
Cl	-4.580103000	-1.118606000	-0.651793000
Cl	-3.375426000	0.625916000	2.016688000
H	-2.340506000	-2.505070000	-0.781301000
H	-0.089563000	-3.042123000	-1.293877000
H	-2.850888000	3.698875000	0.763100000
H	-0.327878000	5.033930000	0.681616000
C	0.537176000	0.799883000	0.048018000
C	1.626900000	0.711685000	1.124133000
H	1.195936000	0.561544000	2.123160000
H	2.305048000	-0.120743000	0.908586000
H	2.216692000	1.634491000	1.137474000
C	1.179112000	1.030298000	-1.338104000
H	1.878609000	0.215381000	-1.562977000
H	0.418517000	1.072694000	-2.129316000
H	1.739795000	1.973920000	-1.337449000
Cl	-2.477098000	0.753262000	-2.524502000
Cl	-4.775252000	2.261684000	-0.554374000

TiBOX-Cl3-F (m1)

C	-0.169142000	-0.522653000	0.014838000
O	0.629003000	-1.563773000	0.188517000
C	-1.625796000	-2.213170000	0.203685000
C	-0.196094000	-2.750582000	0.158078000
H	0.075462000	-3.370964000	1.020611000
C	-0.338483000	1.931944000	0.266640000
O	0.237866000	2.928646000	0.921414000
C	-1.932254000	3.491875000	0.297299000
C	-0.826805000	3.852967000	1.275851000
H	-1.084701000	3.661658000	2.327253000
C	-1.901621000	4.336316000	-0.959608000
H	-0.881414000	4.381390000	-1.370575000
H	-2.562972000	3.927268000	-1.732419000
H	-2.223438000	5.363188000	-0.738858000
C	-2.331208000	-2.469532000	1.515899000
H	-1.781975000	-2.016932000	2.354910000
H	-3.349998000	-2.063899000	1.511264000
H	-2.392732000	-3.552291000	1.693356000
N	-1.578734000	2.087519000	-0.044535000
N	-1.436112000	-0.756291000	-0.047429000
Ti	-3.074333000	0.619560000	-0.643109000
Cl	-4.377691000	-1.207760000	-1.079016000
Cl	-3.659076000	0.762472000	1.630714000
H	-2.217473000	-2.626304000	-0.624210000
H	0.044271000	-3.296765000	-0.763613000
H	-2.921186000	3.508694000	0.776337000
H	-0.441946000	4.873142000	1.171219000
C	0.559010000	0.789559000	-0.120828000
C	1.827590000	0.781303000	0.731828000
H	1.606848000	0.633817000	1.796862000
H	2.500046000	-0.018376000	0.404106000
H	2.357882000	1.733279000	0.619126000
C	0.927567000	0.978825000	-1.608444000
H	1.590132000	0.165430000	-1.932184000
H	0.034160000	0.986059000	-2.243771000
H	1.463116000	1.929140000	-1.736940000
Cl	-4.610246000	2.215991000	-1.232448000
F	-2.274256000	0.645747000	-2.258500000

TiBOX-Cl3-N3 (m1)

C	-0.274446000	-0.479744000	-0.174124000
O	0.465132000	-1.493395000	-0.602457000
C	-1.732578000	-2.168495000	-0.254731000
C	-0.456841000	-2.540415000	-0.992999000
H	-0.027437000	-3.503777000	-0.697741000

C	-0.333876000	1.995235000	0.109012000
O	0.378515000	3.112011000	0.121895000
C	-1.896144000	3.574525000	-0.159793000
C	-0.553912000	4.216071000	0.176428000
H	-0.506260000	4.638395000	1.189238000
C	-2.362859000	3.846052000	-1.573333000
H	-1.605125000	3.529416000	-2.306611000
H	-3.303386000	3.325621000	-1.795374000
H	-2.534226000	4.922896000	-1.708359000
C	-1.876237000	-2.873383000	1.077539000
H	-0.967183000	-2.742796000	1.684754000
H	-2.727522000	-2.484520000	1.648255000
H	-2.029725000	-3.949782000	0.921383000
N	-1.615866000	2.122690000	0.036186000
N	-1.536573000	-0.706128000	-0.029674000
Ti	-3.237736000	0.639254000	0.278104000
Cl	-4.742369000	-1.075982000	0.389841000
Cl	-2.670106000	0.606260000	2.528688000
H	-2.617755000	-2.323649000	-0.886431000
H	-0.551957000	-2.499897000	-2.087689000
H	-2.664480000	3.880013000	0.562148000
H	-0.225003000	4.972012000	-0.546136000
C	0.506331000	0.751210000	0.188086000
C	0.985599000	0.570424000	1.644492000
H	0.141275000	0.461975000	2.337626000
H	1.617840000	-0.324218000	1.716687000
H	1.584386000	1.438724000	1.947852000
C	1.714328000	0.907546000	-0.744100000
H	2.359082000	0.024693000	-0.678192000
H	1.402679000	1.031981000	-1.790233000
H	2.306285000	1.781435000	-0.452287000
Cl	-4.785344000	2.311062000	0.435697000
N	-3.215884000	0.628442000	-1.730722000
N	-2.261529000	0.598422000	-2.456697000
N	-1.341333000	0.567186000	-3.153470000

TiBOX-Cl3-SCN (m1)

C	-0.255503000	-0.519651000	-0.071931000
O	0.528753000	-1.575775000	-0.107440000
C	-1.711941000	-2.223342000	0.070583000
C	-0.309260000	-2.750746000	-0.210009000
H	0.049149000	-3.479617000	0.526634000
C	-0.381225000	1.943454000	0.265831000
O	0.250049000	2.994575000	0.758183000
C	-1.942136000	3.537943000	0.224153000
C	-0.770820000	3.974044000	1.085002000
H	-0.960904000	3.897360000	2.164684000
C	-1.967506000	4.236759000	-1.119410000
H	-0.989096000	4.155179000	-1.617544000
H	-2.726436000	3.815625000	-1.788853000
H	-2.188509000	5.303880000	-0.981127000
C	-2.242824000	-2.612140000	1.431713000
H	-1.570578000	-2.268252000	2.231903000
H	-3.241867000	-2.199146000	1.615550000
H	-2.314140000	-3.706515000	1.495415000
N	-1.644098000	2.084841000	0.030465000
N	-1.533168000	-0.739070000	-0.038926000
Ti	-3.208645000	0.613175000	-0.225346000
Cl	-4.598723000	-1.136334000	-0.616236000
Cl	-3.507372000	0.739072000	2.023379000
H	-2.404396000	-2.543493000	-0.719245000
H	-0.182459000	-3.154355000	-1.222376000
H	-2.895291000	3.649108000	0.757739000
H	-0.377987000	4.968404000	0.848671000
C	0.501209000	0.778393000	-0.065534000
C	1.656318000	0.673015000	0.938133000
H	1.292414000	0.469563000	1.954096000
H	2.339516000	-0.130528000	0.644275000
H	2.222892000	1.609996000	0.955000000
C	1.060240000	1.040167000	-1.482031000
H	1.663974000	0.183779000	-1.808783000
H	0.257770000	1.208420000	-2.212473000
H	1.703268000	1.929512000	-1.462414000
Cl	-4.674473000	2.224809000	-0.859196000
S	-2.564364000	0.710174000	-2.768320000
C	-1.615937000	-0.628970000	-3.000727000
N	-0.925606000	-1.568782000	-3.170191000

TiBOX-Cl3-ONO2 (m1)

C	-0.224141000	-0.452001000	-0.175485000
O	0.550056000	-1.491857000	-0.441276000
C	-1.672464000	-2.154262000	-0.215271000
C	-0.321385000	-2.605936000	-0.750560000
H	0.080175000	-3.497559000	-0.255863000
C	-0.322783000	2.027636000	0.155674000
O	0.357569000	3.138075000	0.400181000
C	-1.887597000	3.616740000	-0.007952000
C	-0.612221000	4.195543000	0.589144000
H	-0.677651000	4.391095000	1.668317000
C	-2.176196000	4.104399000	-1.410504000
H	-1.291093000	3.997464000	-2.053861000
H	-2.997981000	3.541929000	-1.869762000
H	-2.454841000	5.166811000	-1.385049000
C	-1.995526000	-2.726583000	1.147695000
H	-1.182943000	-2.516165000	1.859917000
H	-2.922781000	-2.304866000	1.552606000
H	-2.115123000	-3.816370000	1.077607000
N	-1.595257000	2.151794000	-0.013256000
N	-1.493820000	-0.674129000	-0.115762000
Ti	-3.209473000	0.663649000	-0.039082000
Cl	-4.695575000	-1.067349000	-0.134345000
Cl	-2.983917000	0.635955000	2.252072000
H	-2.471113000	-2.364896000	-0.937650000
H	-0.299108000	-2.749984000	-1.838970000
H	-2.746253000	3.798439000	0.650811000
H	-0.243019000	5.092266000	0.078972000
C	0.545590000	0.807334000	0.088634000
C	1.249780000	0.625710000	1.448027000
H	0.523146000	0.478615000	2.258538000
H	1.914848000	-0.245472000	1.412972000
H	1.854387000	1.511180000	1.676760000
C	1.583393000	1.020378000	-1.026128000
H	2.274915000	0.171022000	-1.058829000
H	1.102455000	1.116747000	-2.009313000
H	2.164758000	1.928558000	-0.830848000
Cl	-4.800494000	2.296509000	-0.100730000

O	-1.572334000	-0.635833000	-2.960984000
N	-1.950917000	0.508392000	-2.804979000
O	-2.982840000	0.699537000	-1.984846000
O	-1.496199000	1.492690000	-3.346422000

TiBOX-Cl3-Me (m1)

C	-0.207639000	-0.443412000	-0.170272000
O	0.567907000	-1.483214000	-0.473303000
C	-1.654970000	-2.134054000	-0.271817000
C	-0.320343000	-2.556005000	-0.865107000
H	0.076896000	-3.495600000	-0.464675000
C	-0.284692000	2.040694000	0.168104000
O	0.433763000	3.148077000	0.334043000
C	-1.807837000	3.657473000	-0.056731000
C	-0.500516000	4.241388000	0.463567000
H	-0.535952000	4.523097000	1.525134000
C	-2.134703000	4.073675000	-1.474891000
H	-1.300125000	3.847192000	-2.155666000
H	-3.034403000	3.568012000	-1.846863000
H	-2.317174000	5.156585000	-1.512953000
C	-1.932378000	-2.750106000	1.083342000
H	-1.080979000	-2.594462000	1.763895000
H	-2.820882000	-2.306793000	1.548658000
H	-2.094586000	-3.832074000	0.982252000
N	-1.554602000	2.191022000	0.016572000
N	-1.473256000	-0.663144000	-0.126144000
Ti	-3.193683000	0.693232000	0.058914000
Cl	-4.712939000	-1.012416000	-0.040162000
Cl	-2.687006000	0.581607000	2.389967000
H	-2.482756000	-2.328578000	-0.966822000
H	-0.322569000	-2.598837000	-1.963889000
H	-2.640292000	3.904758000	0.614402000
H	-0.120403000	5.087341000	-0.120833000
C	0.557163000	0.795811000	0.192516000
C	1.070053000	0.587835000	1.633416000
H	0.233973000	0.461370000	2.334408000
H	1.706173000	-0.305431000	1.679130000
H	1.668420000	1.452565000	1.946594000
C	1.739240000	0.985693000	-0.768743000
H	2.395205000	0.108706000	-0.740788000
H	1.396487000	1.127673000	-1.803225000
H	2.330581000	1.860500000	-0.476831000
Cl	-4.742080000	2.374071000	0.098046000
C	-2.927800000	0.637403000	-2.063227000
H	-2.042376000	1.188266000	-2.413690000
H	-2.856109000	-0.407579000	-2.404053000
H	-3.840526000	1.082047000	-2.496874000

TiBOX-Cl3-NO2 (m1)

C	-0.202071000	-0.504489000	-0.041361000
O	0.588220000	-1.564591000	-0.195985000
C	-1.666698000	-2.188434000	-0.115559000
C	-0.270092000	-2.691202000	-0.475240000
H	0.070417000	-3.541039000	0.128441000
C	-0.293861000	1.999069000	0.207643000
O	0.392945000	3.077058000	0.538996000
C	-1.838296000	3.630300000	0.066901000
C	-0.522341000	4.192323000	0.603402000
H	-0.569396000	4.520244000	1.649481000
C	-2.245761000	4.225454000	-1.260754000
H	-1.490188000	4.021911000	-2.034304000
H	-3.213276000	3.839605000	-1.600193000
H	-2.336285000	5.315346000	-1.155313000
C	-2.212444000	-2.749882000	1.177631000
H	-1.523755000	-2.556112000	2.013467000
H	-3.184075000	-2.304575000	1.423009000
H	-2.343557000	-3.836988000	1.086767000
N	-1.551999000	2.166875000	-0.039564000
N	-1.467063000	-0.717366000	-0.019547000
Ti	-3.173098000	0.739819000	-0.147693000
Cl	-4.579876000	-0.989834000	-0.660979000
Cl	-3.070458000	0.528313000	2.170190000
H	-2.367250000	-2.385700000	-0.938588000
H	-0.155938000	-2.937573000	-1.540299000
H	-2.645008000	3.756590000	0.802641000
H	-0.105544000	5.002329000	-0.007620000
C	0.568100000	0.772868000	0.117929000
C	1.411298000	0.650238000	1.399216000
H	0.773008000	0.549699000	2.288070000
H	2.059138000	-0.231590000	1.334851000
H	2.045817000	1.535186000	1.522613000
C	1.480849000	0.961339000	-1.109808000
H	2.175223000	0.117138000	-1.192319000
H	0.893361000	1.024257000	-2.036473000
H	2.067541000	1.881605000	-1.004264000
Cl	-4.842816000	2.332974000	0.141908000
N	-2.489650000	0.544673000	-2.115890000
O	-1.807364000	0.075569000	-2.983506000
O	-3.343255000	1.447483000	-2.270860000

TiBOX-Cl3-ONO (m1)

C	-0.255872000	-0.457486000	-0.182119000
O	0.523590000	-1.497475000	-0.445490000
C	-1.692641000	-2.171130000	-0.171300000
C	-0.342051000	-2.624237000	-0.709692000
H	0.073651000	-3.500377000	-0.199015000
C	-0.336278000	2.026484000	0.159218000
O	0.364536000	3.140282000	0.334816000
C	-1.888821000	3.624160000	0.024541000
C	-0.590318000	4.202943000	0.563675000
H	-0.611303000	4.400621000	1.644474000
C	-2.189310000	4.049896000	-1.397476000
H	-1.325898000	3.860616000	-2.053974000
H	-3.057490000	3.516764000	-1.804556000
H	-2.407063000	5.126245000	-1.427818000
C	-2.024913000	-2.739398000	1.190942000
H	-1.236025000	-2.496734000	1.919331000
H	-2.975464000	-2.345114000	1.569805000
H	-2.108335000	-3.833117000	1.128870000
N	-1.615010000	2.157786000	0.073260000
N	-1.520459000	-0.690681000	-0.086463000
Ti	-3.223133000	0.672712000	0.007156000
Cl	-4.690521000	-1.056501000	-0.255033000
Cl	-3.012517000	0.579460000	2.315027000
H	-2.492057000	-2.395622000	-0.888962000
H	-0.335022000	-2.799566000	-1.794345000

H	-2.732798000	3.850988000	0.687605000
H	-0.240417000	5.097109000	0.036117000
C	0.520955000	0.800654000	0.073246000
C	1.236357000	0.612768000	1.426984000
H	0.515073000	0.453421000	2.240117000
H	1.908788000	-0.252260000	1.380677000
H	1.834797000	1.501649000	1.659566000
C	1.551815000	1.013692000	-1.047029000
H	2.222716000	0.149534000	-1.107205000
H	1.062499000	1.145743000	-2.021683000
H	2.158066000	1.901705000	-0.836569000
Cl	-4.815901000	2.317695000	0.012136000
N	-1.757171000	0.867431000	-2.553237000
O	-2.930519000	0.799867000	-1.909281000
O	-1.855926000	1.101826000	-3.717509000

TiBOX-Cl2 (m3)

C	-0.102155000	-0.513387000	0.107535000
O	0.631315000	-1.631594000	0.292464000
C	-1.637150000	-2.134922000	-0.041759000
C	-0.233355000	-2.744422000	0.002829000
H	-0.102840000	-3.498461000	0.790290000
C	-0.164972000	1.979330000	0.293560000
O	0.498898000	3.079964000	0.645744000
C	-1.808239000	3.493990000	0.457390000
C	-0.451992000	4.171837000	0.654454000
H	-0.346328000	4.705858000	1.605616000
C	-2.674619000	4.092620000	-0.623202000
H	-2.162639000	4.066694000	-1.596256000
H	-3.614057000	3.526114000	-0.714743000
H	-2.931041000	5.135964000	-0.393607000
C	-2.482732000	-2.447308000	1.174223000
H	-1.959181000	-2.155350000	2.097842000
H	-3.435923000	-1.901547000	1.145857000
H	-2.699066000	-3.523452000	1.231979000
N	-1.435435000	2.104013000	0.125694000
N	-1.367030000	-0.683508000	-0.137658000
Ti	-2.849538000	0.711595000	-0.672233000
Cl	-4.304455000	0.898822000	1.294593000
H	-2.169182000	-2.447762000	-0.954912000
H	0.082141000	-3.180363000	-0.956796000
H	-2.374902000	3.459632000	1.404073000
H	-0.184139000	4.853873000	-0.166471000
C	0.695650000	0.762096000	0.077948000
C	1.800776000	0.720515000	1.139046000
H	1.385908000	0.653232000	2.154294000
H	2.445053000	-0.150309000	0.974705000
H	2.424859000	1.619640000	1.081495000
C	1.321457000	0.892268000	-1.328779000
H	1.989017000	0.041270000	-1.519565000
H	0.538881000	0.906642000	-2.100846000
H	1.913249000	1.815043000	-1.405271000
Cl	-2.334895000	0.840317000	-3.044495000

TiBOX-Cl2-OH (m2)

C	-0.096613000	-0.529349000	0.059229000
O	0.634361000	-1.599446000	0.337457000
C	-1.632458000	-2.168995000	0.048941000
C	-0.238918000	-2.753775000	0.293687000
H	-0.141357000	-3.280724000	1.251801000
C	-0.173019000	1.955827000	0.269324000
O	0.480112000	3.024333000	0.724576000
C	-1.828270000	3.435607000	0.516518000
C	-0.481287000	4.105702000	0.795931000
H	-0.398217000	4.566899000	1.786619000
C	-2.673555000	4.123857000	-0.527856000
H	-2.128184000	4.228746000	-1.477733000
H	-3.595786000	3.553930000	-0.715236000
H	-2.964960000	5.128703000	-0.192131000
C	-2.605709000	-2.374796000	1.186388000
H	-2.222399000	-1.935782000	2.119856000
H	-3.572720000	-1.908858000	0.955159000
H	-2.773194000	-3.447689000	1.355233000
N	-1.436429000	2.078226000	0.090866000
N	-1.353696000	-0.727694000	-0.159563000
Ti	-2.819282000	0.649996000	-0.859419000
Cl	-4.285773000	-1.016327000	-1.781349000
Cl	-4.157795000	1.045146000	1.086328000
H	-2.072437000	-2.559469000	-0.881374000
H	0.120151000	-3.410393000	-0.508986000
H	-2.419169000	3.320183000	1.441256000
H	-0.202508000	4.848480000	0.033596000
C	0.689206000	0.756901000	-0.037713000
C	1.889499000	0.717143000	0.910851000
H	1.579597000	0.607331000	1.958508000
H	2.548945000	-0.119110000	0.655650000
H	2.466995000	1.643365000	0.818938000
C	1.177004000	0.890032000	-1.496069000
H	1.831955000	0.046213000	-1.750548000
H	0.331045000	0.905331000	-2.196042000
H	1.753232000	1.817671000	-1.612457000
O	-2.122137000	1.214962000	-2.468838000
H	-1.687370000	2.062811000	-2.619537000

TiBOX-Cl2-F (m2)

C	-0.095069000	-0.519159000	0.071497000
O	0.635152000	-1.590777000	0.328898000
C	-1.631450000	-2.162536000	0.037902000
C	-0.237034000	-2.747275000	0.276412000
H	-0.136589000	-3.280530000	1.230573000
C	-0.164071000	1.966178000	0.300056000
O	0.493075000	3.041703000	0.722638000
C	-1.819418000	3.453214000	0.525726000
C	-0.471005000	4.121548000	0.809287000
H	-0.386384000	4.567835000	1.806707000
C	-2.637677000	4.108266000	-0.561810000
H	-2.080779000	4.143248000	-1.509787000
H	-3.572577000	3.552568000	-0.728650000
H	-2.906242000	5.136498000	-0.283413000
C	-2.608463000	-2.393960000	1.166693000
H	-2.229773000	-1.974353000	2.110780000
H	-3.575982000	-1.926120000	0.941963000
H	-2.773873000	-3.470543000	1.311639000
N	-1.431157000	2.083145000	0.143623000
N	-1.355015000	-0.716296000	-0.141746000
Ti	-2.798005000	0.682070000	-0.809305000

Cl	-4.250491000	-0.969723000	-1.786655000
Cl	-4.158350000	1.024466000	1.110319000
H	-2.064951000	-2.534708000	-0.902637000
H	0.123820000	-3.395601000	-0.531754000
H	-2.427905000	3.371672000	1.442410000
H	-0.193084000	4.874238000	0.057359000
C	0.692116000	0.766885000	-0.015712000
C	1.897587000	0.715773000	0.924981000
H	1.593217000	0.589540000	1.972413000
H	2.558016000	-0.114805000	0.654176000
H	2.472167000	1.644768000	0.843559000
C	1.166033000	0.914283000	-1.478457000
H	1.800538000	0.061943000	-1.754762000
H	0.312263000	0.962955000	-2.166679000
H	1.760106000	1.831768000	-1.583225000
F	-2.076077000	1.175846000	-2.404447000

TiBOX-Cl2-N3 (m2)

C	-0.127320000	-0.511856000	0.091634000
O	0.624351000	-1.598064000	0.157430000
C	-1.650652000	-2.166329000	0.025031000
C	-0.238369000	-2.747715000	-0.024294000
H	-0.017285000	-3.461657000	0.778500000
C	-0.170009000	1.999131000	0.281514000
O	0.515797000	3.104471000	0.558845000
C	-1.792952000	3.535860000	0.393368000
C	-0.426460000	4.205882000	0.558007000
H	-0.302507000	4.755001000	1.498256000
C	-2.648371000	4.083079000	-0.724771000
H	-2.114113000	4.052829000	-1.685371000
H	-3.573506000	3.495057000	-0.825538000
H	-2.933890000	5.124224000	-0.522919000
C	-2.446352000	-2.541401000	1.254075000
H	-1.925836000	-2.236370000	2.174369000
H	-3.436900000	-2.067229000	1.235318000
H	-2.593760000	-3.629577000	1.288955000
N	-1.438799000	2.124619000	0.150932000
N	-1.401632000	-0.701468000	-0.020023000
Ti	-2.944841000	0.686033000	-0.429003000
Cl	-4.540149000	-0.901533000	-1.244385000
Cl	-3.861780000	1.051035000	1.704449000
H	-2.218962000	-2.435544000	-0.878191000
H	0.021871000	-3.200463000	-0.990215000
H	-2.363346000	3.559832000	1.336965000
H	-0.162845000	4.869084000	-0.278475000
C	0.674309000	0.765950000	0.116769000
C	1.690647000	0.692555000	1.266093000
H	1.190828000	0.603118000	2.240005000
H	2.352906000	-0.170201000	1.134866000
H	2.309621000	1.596304000	1.276643000
C	1.413915000	0.881352000	-1.231934000
H	2.074161000	0.016913000	-1.374998000
H	0.711245000	0.931184000	-2.074485000
H	2.030105000	1.789182000	-1.239245000
N	-2.226965000	0.916909000	-2.341996000
N	-1.878484000	0.016649000	-3.053116000
N	-1.515111000	-0.834383000	-3.749222000

TiBOX-Cl2-SCN (m2)

C	-0.131989000	-0.507755000	0.008935000
O	0.612688000	-1.597046000	0.032259000
C	-1.668178000	-2.154147000	-0.021873000
C	-0.261774000	-2.741066000	-0.130372000
H	-0.013638000	-3.462902000	0.657195000
C	-0.173084000	1.994110000	0.233197000
O	0.505052000	3.100323000	0.506379000
C	-1.806169000	3.524986000	0.363977000
C	-0.440626000	4.199694000	0.510376000
H	-0.308138000	4.752443000	1.446976000
C	-2.683187000	4.074356000	-0.735715000
H	-2.158446000	4.077823000	-1.702220000
H	-3.601232000	3.473407000	-0.836267000
H	-2.987053000	5.105299000	-0.510024000
C	-2.418506000	-2.540175000	1.231583000
H	-1.863391000	-2.248219000	2.135630000
H	-3.408864000	-2.066453000	1.260801000
H	-2.566421000	-3.628371000	1.257838000
N	-1.445722000	2.114340000	0.109619000
N	-1.412875000	-0.688046000	-0.062188000
Ti	-2.965471000	0.686428000	-0.392240000
Cl	-4.504855000	-0.835176000	-1.277289000
Cl	-3.826489000	1.055438000	1.735617000
H	-2.262168000	-2.412806000	-0.910895000
H	-0.042811000	-3.182261000	-1.110847000
H	-2.358954000	3.540641000	1.318145000
H	-0.187054000	4.858358000	-0.332418000
C	0.673589000	0.764491000	0.071864000
C	1.645604000	0.663379000	1.259270000
H	1.109769000	0.541567000	2.210149000
H	2.317246000	-0.191731000	1.124449000
H	2.256876000	1.570751000	1.317778000
C	1.469010000	0.907516000	-1.241077000
H	2.083812000	0.014399000	-1.407093000
H	0.804031000	1.045590000	-2.103717000
H	2.135564000	1.776258000	-1.172861000
S	-2.007609000	1.087933000	-2.852231000
C	-1.497340000	-0.466595000	-3.110405000
N	-1.102843000	-1.564145000	-3.287232000

TiBOX-Cl2-ONO2 (m2)

C	-0.038834000	-0.531849000	0.151418000
O	0.712155000	-1.607419000	0.294081000
C	-1.556276000	-2.194886000	0.068178000
C	-0.154083000	-2.769431000	0.276087000
H	-0.027518000	-3.287560000	1.236202000
C	-0.121548000	1.972121000	0.305229000
O	0.537175000	3.087227000	0.597787000
C	-1.777570000	3.476701000	0.405066000
C	-0.425291000	4.171473000	0.584107000
H	-0.321877000	4.724245000	1.524527000
C	-2.630890000	4.018063000	-0.717281000
H	-2.103595000	3.962346000	-1.680128000
H	-3.568639000	3.447578000	-0.801401000
H	-2.897542000	5.066723000	-0.528741000
C	-2.543952000	-2.544137000	1.155504000
H	-2.197870000	-2.187364000	2.136874000

H	-3.527173000	-2.100913000	0.948850000
H	-2.671537000	-3.633837000	1.212227000
N	-1.392432000	2.072156000	0.158123000
N	-1.308096000	-0.732735000	0.006013000
Ti	-2.794738000	0.613932000	-0.594880000
Cl	-4.161782000	-0.990669000	-1.692644000
Cl	-4.065612000	0.962053000	1.319798000
H	-1.963457000	-2.492096000	-0.910494000
H	0.189688000	-3.426807000	-0.531056000
H	-2.355379000	3.490177000	1.344944000
H	-0.163268000	4.836999000	-0.251016000
C	0.746854000	0.755312000	0.139968000
C	1.787366000	0.717277000	1.268103000
H	1.310711000	0.625187000	2.253271000
H	2.466895000	-0.130757000	1.130712000
H	2.384878000	1.635204000	1.257547000
C	1.455405000	0.864942000	-1.225843000
H	2.111673000	-0.001139000	-1.379305000
H	0.732996000	0.908140000	-2.050432000
H	2.072202000	1.772198000	-1.248980000
O	-2.027010000	1.132804000	-2.373554000
N	-1.789217000	0.275021000	-3.359345000
O	-2.267618000	0.537671000	-4.443047000
O	-1.083215000	-0.689295000	-3.111245000

TiBOX-Cl2-Me (m2)

C	-0.016402000	-0.605896000	0.153212000
O	0.695255000	-1.647857000	0.554009000
C	-1.557834000	-2.237949000	0.185535000
C	-0.187943000	-2.797964000	0.578909000
H	-0.157024000	-3.216487000	1.593604000
C	-0.121946000	1.875918000	0.268589000
O	0.494831000	2.917527000	0.818628000
C	-1.794005000	3.342012000	0.481495000
C	-0.467275000	3.999890000	0.863100000
H	-0.442694000	4.432142000	1.869651000
C	-2.602110000	4.112239000	-0.532444000
H	-2.023835000	4.305117000	-1.447340000
H	-3.514198000	3.562581000	-0.803174000
H	-2.906775000	5.082137000	-0.114796000
C	-2.616355000	-2.358702000	1.256836000
H	-2.297049000	-1.863339000	2.186182000
H	-3.556520000	-1.897028000	0.925657000
H	-2.814355000	-3.415906000	1.481835000
N	-1.370500000	2.010386000	-0.003529000
N	-1.260978000	-0.817914000	-0.112264000
Ti	-2.678195000	0.537401000	-0.972851000
Cl	-3.905264000	-1.229172000	-2.079538000
Cl	-4.254583000	0.978663000	0.743228000
H	-1.926526000	-2.696841000	-0.744404000
H	0.215126000	-3.538323000	-0.123205000
H	-2.419344000	3.162400000	1.372731000
H	-0.140610000	4.761565000	0.138986000
C	0.756968000	0.680049000	-0.015895000
C	1.984472000	0.690248000	0.894267000
H	1.710059000	0.629086000	1.955325000
H	2.638552000	-0.155693000	0.657392000
H	2.555083000	1.612483000	0.740225000
C	1.200629000	0.760887000	-1.491627000
H	1.854199000	-0.087828000	-1.733519000
H	0.339186000	0.744140000	-2.172881000
H	1.765379000	1.687386000	-1.661330000
C	-2.396074000	1.547837000	-2.835651000
H	-1.661604000	2.370207000	-2.832456000
H	-2.110227000	0.818182000	-3.614217000
H	-3.383908000	1.960423000	-3.115655000

TiBOX-Cl2-NO2 (m2)

C	-0.073372000	-0.557163000	0.032749000
O	0.662473000	-1.641571000	0.225826000
C	-1.614996000	-2.194132000	0.013244000
C	-0.213429000	-2.791804000	0.158298000
H	-0.072212000	-3.371756000	1.079350000
C	-0.137857000	1.936705000	0.215644000
O	0.508542000	3.013486000	0.651369000
C	-1.788761000	3.432835000	0.424667000
C	-0.438160000	4.108883000	0.641906000
H	-0.344727000	4.646494000	1.591743000
C	-2.687047000	4.125549000	-0.568568000
H	-2.198199000	4.231654000	-1.547462000
H	-3.626146000	3.570359000	-0.705171000
H	-2.945682000	5.130289000	-0.206750000
C	-2.532147000	-2.456238000	1.185067000
H	-2.096118000	-2.075173000	2.120568000
H	-3.507958000	-1.975323000	1.034981000
H	-2.700383000	-3.535846000	1.300756000
N	-1.398000000	2.068049000	0.000005000
N	-1.340022000	-0.743354000	-0.131076000
Ti	-2.823776000	0.638925000	-0.796459000
Cl	-4.300966000	-0.990373000	-1.671236000
Cl	-4.077994000	1.052485000	1.136838000
H	-2.095242000	-2.538029000	-0.914883000
H	0.104095000	-3.400925000	-0.697249000
H	-2.328773000	3.311610000	1.380227000
H	-0.157622000	4.783572000	-0.180946000
C	0.727609000	0.722184000	0.011837000
C	1.788998000	0.664510000	1.119725000
H	1.334715000	0.566508000	2.114756000
H	2.457197000	-0.187817000	0.957656000
H	2.395329000	1.576355000	1.105025000
C	1.425004000	0.848462000	-1.358376000
H	2.081885000	-0.015072000	-1.524933000
H	0.697376000	0.905203000	-2.177119000
H	2.044510000	1.754762000	-1.373092000
N	-2.624716000	1.517923000	-2.588080000
O	-1.632228000	0.689111000	-2.520728000
O	-2.827834000	2.214848000	-3.551870000

TiBOX-Cl2-ONO (m2)

C	-0.142156000	-0.546362000	-0.129850000
O	0.599915000	-1.632676000	-0.251621000
C	-1.671418000	-2.196899000	-0.061631000
C	-0.287209000	-2.774846000	-0.349960000
H	0.047958000	-3.520047000	0.382035000
C	-0.155405000	1.961925000	0.051115000
O	0.557659000	3.083008000	0.111463000

C	-1.752843000	3.520651000	0.219694000
C	-0.378545000	4.188095000	0.118881000
H	-0.127426000	4.837208000	0.965322000
C	-2.755098000	3.964812000	-0.819457000
H	-2.354639000	3.844282000	-1.836645000
H	-3.681774000	3.375026000	-0.742830000
H	-3.019083000	5.021232000	-0.676096000
C	-2.272218000	-2.621324000	1.258266000
H	-1.600193000	-2.380179000	2.095608000
H	-3.234474000	-2.117954000	1.425739000
H	-2.451912000	-3.705179000	1.264436000
N	-1.432190000	2.085714000	0.085593000
N	-1.422150000	-0.731738000	-0.063458000
Ti	-2.997606000	0.633502000	-0.286160000
Cl	-4.697589000	-0.920573000	-0.919715000
Cl	-3.776935000	1.113318000	1.872246000
H	-2.372719000	-2.424556000	-0.877241000
H	-0.177804000	-3.188879000	-1.360381000
H	-2.184295000	3.655762000	1.226130000
H	-0.236658000	4.750309000	-0.815429000
C	0.672923000	0.709383000	0.033323000
C	1.353516000	0.602020000	1.417378000
H	0.608818000	0.532751000	2.222369000
H	1.994956000	-0.286836000	1.453781000
H	1.978331000	1.485793000	1.593657000
C	1.741149000	0.800469000	-1.064035000
H	2.379927000	-0.090001000	-1.045569000
H	1.290574000	0.888785000	-2.060962000
H	2.376427000	1.676524000	-0.889313000
O	-2.506587000	0.932845000	-2.189844000
N	-1.253584000	0.928829000	-2.619865000
O	-1.116139000	1.302082000	-3.754183000

VBOX-Cl3 (m3)

C	-0.151613000	-0.507342000	0.018611000
O	0.577938000	-1.615187000	0.080584000
C	-1.708326000	-2.123849000	-0.065763000
C	-0.309853000	-2.732424000	-0.162333000
H	-0.103134000	-3.499284000	0.593693000
C	-0.174579000	1.986505000	0.256075000
O	0.498697000	3.085214000	0.579005000
C	-1.807759000	3.517182000	0.386946000
C	-0.443715000	4.187265000	0.558564000
H	-0.327697000	4.746796000	1.493268000
C	-2.680876000	4.099109000	-0.698726000
H	-2.170973000	4.078483000	-1.671622000
H	-3.617746000	3.528738000	-0.791649000
H	-2.945648000	5.138865000	-0.464163000
C	-2.458646000	-2.489682000	1.195245000
H	-1.875690000	-2.228635000	2.091342000
H	-3.421528000	-1.965844000	1.244551000
H	-2.651729000	-3.571106000	1.215704000
N	-1.439872000	2.118470000	0.098417000
N	-1.423590000	-0.668150000	-0.111995000
V	-2.909000000	0.724454000	-0.462695000
Cl	-4.616065000	-0.723862000	-1.080629000
Cl	-3.627971000	0.959349000	1.758489000
H	-2.315176000	-2.374056000	-0.947940000
H	-0.069157000	-3.134223000	-1.155766000
H	-2.366509000	3.501053000	1.338428000
H	-0.172739000	4.839677000	-0.284296000
C	0.667305000	0.756368000	0.073874000
C	1.672779000	0.652519000	1.229174000
H	1.164428000	0.550171000	2.197466000
H	2.325846000	-0.215490000	1.086506000
H	2.302124000	1.548754000	1.261276000
C	1.410468000	0.898679000	-1.269489000
H	2.057444000	0.028146000	-1.435291000
H	0.700980000	0.979222000	-2.104355000
H	2.040448000	1.797126000	-1.253460000
Cl	-2.206193000	1.121009000	-2.666467000

VBOX-Cl3-OH (m2)

C	-0.242567000	-0.436711000	-0.171522000
O	0.519933000	-1.474989000	-0.505025000
C	-1.703969000	-2.120213000	-0.276761000
C	-0.382418000	-2.536488000	-0.903810000
H	0.018299000	-3.485080000	-0.529453000
C	-0.293827000	2.052592000	0.151241000
O	0.427351000	3.165569000	0.229373000
C	-1.852321000	3.651510000	-0.026077000
C	-0.499702000	4.273155000	0.315160000
H	-0.441748000	4.674764000	1.335761000
C	-2.366879000	4.010285000	-1.402684000
H	-1.632521000	3.748466000	-2.180148000
H	-3.309589000	3.492254000	-1.619742000
H	-2.551872000	5.091639000	-1.464149000
C	-1.948206000	-2.764619000	1.072033000
H	-1.083316000	-2.616635000	1.737201000
H	-2.833498000	-2.339356000	1.558101000
H	-2.102352000	-3.845790000	0.950406000
N	-1.568906000	2.198877000	0.068063000
N	-1.508991000	-0.656581000	-0.109510000
V	-3.140436000	0.722668000	0.118053000
Cl	-4.721896000	-0.954938000	0.220907000
Cl	-2.573929000	0.595975000	2.393434000
H	-2.552037000	-2.295157000	-0.952890000
H	-0.400612000	-2.556203000	-2.002907000
H	-2.607647000	3.901024000	0.731731000
H	-0.170698000	5.043681000	-0.392649000
C	0.536925000	0.799379000	0.184969000
C	1.056109000	0.600693000	1.625225000
H	0.227031000	0.486886000	2.335785000
H	1.688435000	-0.295306000	1.672576000
H	1.663131000	1.464968000	1.923456000
C	1.721771000	0.967636000	-0.776447000
H	2.370538000	0.085969000	-0.737178000
H	1.384519000	1.102637000	-1.813279000
H	2.319036000	1.840418000	-0.491004000
Cl	-4.762303000	2.323393000	0.544895000
O	-3.105098000	0.856332000	-1.638794000
H	-2.320648000	1.061842000	-2.173814000

VBOX-Cl4 (m2)

C	-0.238064000	-0.486260000	-0.071762000
O	0.550100000	-1.538551000	-0.246882000

C	-1.685215000	-2.193901000	-0.092862000
C	-0.307977000	-2.667177000	-0.539919000
H	0.070408000	-3.534523000	0.012504000
C	-0.336674000	2.012960000	0.235972000
O	0.353985000	3.115620000	0.494661000
C	-1.891075000	3.617447000	0.101325000
C	-0.608021000	4.178474000	0.699529000
H	-0.666679000	4.362584000	1.781162000
C	-2.177941000	4.124699000	-1.295687000
H	-1.321268000	3.944464000	-1.962894000
H	-3.058005000	3.633625000	-1.727327000
H	-2.365787000	5.206854000	-1.266450000
C	-2.088454000	-2.727149000	1.265090000
H	-1.333645000	-2.475037000	2.025388000
H	-3.053788000	-2.320510000	1.587890000
H	-2.175014000	-3.821496000	1.220748000
N	-1.607067000	2.157807000	0.078552000
N	-1.505612000	-0.717227000	-0.048110000
V	-3.113200000	0.670635000	-0.062671000
Cl	-4.663886000	-1.008251000	-0.251350000
Cl	-2.966489000	0.604891000	2.220132000
H	-2.451475000	-2.429387000	-0.843422000
H	-0.234956000	-2.863872000	-1.618458000
H	-2.751790000	3.794699000	0.759459000
H	-0.233104000	5.077743000	0.198119000
C	0.527759000	0.791880000	0.114464000
C	1.367309000	0.640888000	1.396314000
H	0.727189000	0.489869000	2.276602000
H	2.043766000	-0.216962000	1.304948000
H	1.972218000	1.541053000	1.555266000
C	1.442220000	1.006368000	-1.106270000
H	2.127749000	0.158193000	-1.216524000
H	0.853546000	1.104882000	-2.028833000
H	2.038333000	1.916650000	-0.972616000
Cl	-2.749218000	0.771504000	-2.321898000
Cl	-4.789595000	2.238618000	-0.029054000

VBOX-Cl3-F (m2)

C	-0.190243000	-0.512153000	0.040220000
O	0.615305000	-1.560137000	0.110805000
C	-1.633601000	-2.230946000	0.072564000
C	-0.199804000	-2.745955000	-0.041596000
H	0.085493000	-3.452039000	0.747301000
C	-0.355009000	1.969030000	0.321776000
O	0.257263000	3.017485000	0.852198000
C	-1.928508000	3.552548000	0.249900000
C	-0.775844000	3.999188000	1.135275000
H	-0.993582000	3.940557000	2.210872000
C	-1.956320000	4.265057000	-1.087141000
H	-0.970141000	4.214653000	-1.574081000
H	-2.698548000	3.821423000	-1.761822000
H	-2.209188000	5.325042000	-0.946964000
C	-2.319261000	-2.622782000	1.361627000
H	-1.755890000	-2.266514000	2.236803000
H	-3.336620000	-2.217508000	1.412401000
H	-2.383974000	-3.718081000	1.421719000
N	-1.603419000	2.119096000	0.040606000
N	-1.454178000	-0.757896000	-0.034168000
V	-3.024308000	0.626518000	-0.460778000
Cl	-4.481446000	-1.090622000	-0.914276000
Cl	-3.442508000	0.645284000	1.804592000
H	-2.235524000	-2.561069000	-0.785058000
H	0.039314000	-3.184314000	-1.019604000
H	-2.896077000	3.631871000	0.764482000
H	-0.376611000	4.990785000	0.896090000
C	0.539495000	0.804283000	0.000587000
C	1.733996000	0.764773000	0.956896000
H	1.416504000	0.612963000	1.997010000
H	2.414847000	-0.046513000	0.679495000
H	2.289434000	1.706964000	0.900105000
C	1.027376000	1.018315000	-1.450256000
H	1.718049000	0.213462000	-1.732577000
H	0.185882000	1.029990000	-2.154852000
H	1.564602000	1.973179000	-1.524925000
Cl	-4.663325000	2.183425000	-0.894543000
F	-2.340368000	0.697137000	-2.087365000

VBOX-Cl3-N3 (m2)

C	-0.279602000	-0.488189000	-0.171256000
O	0.463960000	-1.511622000	-0.567045000
C	-1.742940000	-2.176255000	-0.243849000
C	-0.455880000	-2.564646000	-0.953631000
H	-0.031947000	-3.522885000	-0.635142000
C	-0.338987000	1.996258000	0.095312000
O	0.369923000	3.113961000	0.113486000
C	-1.909386000	3.574416000	-0.163238000
C	-0.566285000	4.217792000	0.167457000
H	-0.514490000	4.642361000	1.179000000
C	-2.389371000	3.850220000	-1.571662000
H	-1.641506000	3.531300000	-2.314252000
H	-3.335047000	3.333637000	-1.780429000
H	-2.558569000	4.927739000	-1.703613000
C	-1.916890000	-2.854458000	1.098719000
H	-1.024132000	-2.711067000	1.726957000
H	-2.784367000	-2.451055000	1.633589000
H	-2.066341000	-3.933905000	0.960275000
N	-1.621037000	2.127327000	0.022001000
N	-1.544291000	-0.715047000	-0.044508000
V	-3.153188000	0.650976000	0.240993000
Cl	-4.734527000	-1.018399000	0.356503000
Cl	-2.614027000	0.601406000	2.459577000
H	-2.621595000	-2.336499000	-0.883627000
H	-0.531765000	-2.541292000	-2.050423000
H	-2.677950000	3.867947000	0.563794000
H	-0.239187000	4.971599000	-0.558066000
C	0.500213000	0.750513000	0.172593000
C	1.001777000	0.582138000	1.623097000
H	0.169358000	0.477881000	2.330826000
H	1.637019000	-0.310460000	1.692126000
H	1.603380000	1.454010000	1.909891000
C	1.696497000	0.901940000	-0.776487000
H	2.345349000	0.022125000	-0.709909000
H	1.372096000	1.016047000	-1.819869000
H	2.288414000	1.780674000	-0.499173000
Cl	-4.783879000	2.254827000	0.439847000
N	-3.162186000	0.644768000	-1.713030000

N	-2.228507000	0.587513000	-2.465411000
N	-1.334799000	0.528304000	-3.191738000

VBOX-Cl3-SCN (m2)

C	-0.249978000	-0.523801000	-0.065604000
O	0.539314000	-1.574020000	-0.152719000
C	-1.694723000	-2.240738000	0.033723000
C	-0.295834000	-2.749402000	-0.290013000
H	0.080046000	-3.507064000	0.407710000
C	-0.365456000	1.965369000	0.235799000
O	0.295738000	3.057703000	0.577799000
C	-1.933474000	3.558907000	0.141314000
C	-0.698486000	4.076641000	0.859374000
H	-0.809251000	4.128358000	1.951227000
C	-2.088543000	4.140352000	-1.247472000
H	-1.173827000	3.986816000	-1.840235000
H	-2.933037000	3.692545000	-1.783841000
H	-2.270292000	5.221491000	-1.176597000
C	-2.207726000	-2.672399000	1.388330000
H	-1.520666000	-2.366096000	2.191148000
H	-3.196553000	-2.246705000	1.595842000
H	-2.296130000	-3.766941000	1.413680000
N	-1.638487000	2.097618000	0.061814000
N	-1.525106000	-0.758639000	-0.029750000
V	-3.115446000	0.613987000	-0.102639000
Cl	-4.517568000	-1.078295000	-0.668333000
Cl	-3.259327000	0.648511000	2.134535000
H	-2.400637000	-2.536685000	-0.753611000
H	-0.182584000	-3.110496000	-1.320528000
H	-2.841342000	3.709634000	0.739548000
H	-0.321670000	5.033153000	0.482093000
C	0.509903000	0.769814000	0.011483000
C	1.523754000	0.650693000	1.161703000
H	1.017510000	0.488355000	2.123029000
H	2.206175000	-0.186183000	0.979011000
H	2.119180000	1.567136000	1.231847000
C	1.243206000	0.997412000	-1.327296000
H	1.893172000	0.141758000	-1.547086000
H	0.536203000	1.127133000	-2.158309000
H	1.867673000	1.896562000	-1.258464000
Cl	-4.764031000	2.153457000	-0.480702000
S	-2.548814000	0.878660000	-2.596522000
C	-1.917128000	-0.608171000	-2.963872000
N	-1.450217000	-1.656813000	-3.228926000

VBOX-Cl3-ONO2 (m2)

C	-0.218291000	-0.460419000	-0.172325000
O	0.519880000	-1.461770000	-0.611993000
C	-1.684026000	-2.149475000	-0.278811000
C	-0.392815000	-2.523708000	-0.991041000
H	0.042855000	-3.476540000	-0.671720000
C	-0.263848000	2.018366000	0.108361000
O	0.446361000	3.128725000	0.128496000
C	-1.826311000	3.607867000	-0.165636000
C	-0.482370000	4.241356000	0.176080000
H	-0.433487000	4.661475000	1.189264000
C	-2.285504000	3.884433000	-1.580953000
H	-1.513414000	3.600061000	-2.312379000
H	-3.215941000	3.351403000	-1.814568000
H	-2.478111000	4.958900000	-1.704029000
C	-1.893145000	-2.879141000	1.030674000
H	-1.025928000	-2.749533000	1.696478000
H	-2.785971000	-2.509147000	1.548694000
H	-2.024044000	-3.954226000	0.846976000
N	-1.548298000	2.158750000	0.031761000
N	-1.480329000	-0.698618000	-0.024256000
V	-3.032534000	0.686713000	0.210901000
Cl	-4.625844000	-0.960638000	0.118141000
Cl	-2.672803000	0.601005000	2.442598000
H	-2.551821000	-2.278588000	-0.938773000
H	-0.470068000	-2.504089000	-2.087171000
H	-2.600177000	3.910704000	0.551273000
H	-0.141607000	4.992429000	-0.545575000
C	0.559108000	0.766373000	0.211473000
C	0.977811000	0.586038000	1.688185000
H	0.106514000	0.488045000	2.348774000
H	1.596383000	-0.315643000	1.785773000
H	1.574087000	1.448583000	2.011890000
C	1.806337000	0.909156000	-0.667797000
H	2.442475000	0.024287000	-0.560863000
H	1.545617000	1.024133000	-1.727695000
H	2.389635000	1.782024000	-0.356306000
Cl	-4.673126000	2.284839000	0.375088000
O	-0.991041000	0.779174000	-2.492641000
N	-2.180981000	0.930204000	-2.718609000
O	-3.049303000	0.715772000	-1.744847000
O	-2.659902000	1.255922000	-3.782692000

VBOX-Cl3-Me (m2)

C	-0.214880000	-0.442092000	-0.161266000
O	0.552461000	-1.492884000	-0.449048000
C	-1.677810000	-2.120887000	-0.272009000
C	-0.344568000	-2.551546000	-0.859575000
H	0.037948000	-3.499936000	-0.465954000
C	-0.292984000	2.034011000	0.173325000
O	0.416701000	3.148682000	0.340336000
C	-1.829057000	3.637446000	-0.054558000
C	-0.530261000	4.229586000	0.477206000
H	-0.575340000	4.499243000	1.541736000
C	-2.146981000	4.052943000	-1.474920000
H	-1.302740000	3.838672000	-2.147777000
H	-3.036437000	3.537591000	-1.857921000
H	-2.342182000	5.133675000	-1.512276000
C	-1.955810000	-2.729477000	1.086384000
H	-1.094414000	-2.591118000	1.757976000
H	-2.829042000	-2.266925000	1.561059000
H	-2.139763000	-3.808114000	0.986402000
N	-1.562520000	2.173945000	0.020219000
N	-1.482912000	-0.649683000	-0.134360000
V	-3.155521000	0.688725000	0.046126000
Cl	-4.688811000	-0.972194000	-0.001104000
Cl	-2.604632000	0.584324000	2.366142000
H	-2.505830000	-2.312534000	-0.967085000
H	-0.339065000	-2.582221000	-1.958903000
H	-2.667925000	3.875793000	0.611164000
H	-0.158207000	5.086622000	-0.096077000

C	0.555787000	0.795624000	0.186709000
C	1.103867000	0.593312000	1.614732000
H	0.285246000	0.466773000	2.335755000
H	1.745078000	-0.296794000	1.647819000
H	1.706232000	1.461551000	1.909922000
C	1.714898000	0.985952000	-0.802878000
H	2.373625000	0.110544000	-0.786259000
H	1.348557000	1.123253000	-1.829935000
H	2.309826000	1.863661000	-0.527082000
Cl	-4.711146000	2.327062000	0.075341000
C	-2.855348000	0.636764000	-2.039303000
H	-1.955426000	1.165964000	-2.380241000
H	-2.837128000	-0.407660000	-2.383537000
H	-3.761203000	1.136733000	-2.420885000

VBOX-Cl3-NO2 (m2)

C	-0.249006000	-0.431690000	-0.211563000
O	0.497186000	-1.451807000	-0.590305000
C	-1.677806000	-2.153254000	-0.185853000
C	-0.414666000	-2.533500000	-0.933868000
H	0.040402000	-3.475302000	-0.610987000
C	-0.356491000	2.030711000	0.175215000
O	0.301047000	3.140195000	0.500791000
C	-1.919399000	3.606809000	-0.017010000
C	-0.709421000	4.156477000	0.722040000
H	-0.858419000	4.239499000	1.808437000
C	-2.050389000	4.146143000	-1.425507000
H	-1.109074000	4.018510000	-1.981296000
H	-2.848504000	3.637492000	-1.978005000
H	-2.285579000	5.219005000	-1.396220000
C	-1.747956000	-2.758609000	1.201025000
H	-0.804840000	-2.597816000	1.745513000
H	-2.564607000	-2.320542000	1.786359000
H	-1.917921000	-3.841477000	1.130313000
N	-1.614781000	2.151592000	-0.051534000
N	-1.514274000	-0.673764000	-0.067270000
V	-3.156412000	0.615784000	-0.003390000
Cl	-4.649812000	-1.021833000	0.619275000
Cl	-2.974140000	1.152800000	2.240810000
H	-2.583568000	-2.374854000	-0.765211000
H	-0.522812000	-2.528048000	-2.027586000
H	-2.844745000	3.761971000	0.553129000
H	-0.331243000	5.107008000	0.329801000
C	0.516253000	0.812580000	0.119651000
C	1.117626000	0.573432000	1.520400000
H	0.325621000	0.424288000	2.267074000
H	1.763343000	-0.313520000	1.509742000
H	1.723726000	1.438350000	1.815756000
C	1.621249000	1.054872000	-0.916636000
H	2.311230000	0.203649000	-0.940007000
H	1.198877000	1.191760000	-1.921715000
H	2.193073000	1.952012000	-0.653662000
Cl	-4.729179000	2.148151000	-0.666960000
N	-2.673494000	0.626383000	-2.001732000
O	-3.481787000	-0.309961000	-2.089663000
O	-2.004807000	1.085392000	-2.882309000

VBOX-Cl3-ONO (m2)

C	-0.251486000	-0.455236000	-0.202256000
O	0.517642000	-1.491988000	-0.494121000
C	-1.699574000	-2.163596000	-0.203063000
C	-0.358054000	-2.613041000	-0.765594000
H	0.062688000	-3.497774000	-0.274304000
C	-0.322316000	2.033671000	0.128849000
O	0.379924000	3.149047000	0.261264000
C	-1.889088000	3.627937000	0.009229000
C	-0.569103000	4.222295000	0.479560000
H	-0.550420000	4.461399000	1.551657000
C	-2.267850000	4.037895000	-1.398333000
H	-1.446344000	3.834469000	-2.102191000
H	-3.165703000	3.509441000	-1.742712000
H	-2.476787000	5.115998000	-1.427406000
C	-2.017983000	-2.749710000	1.155309000
H	-1.216560000	-2.528062000	1.876717000
H	-2.958775000	-2.348448000	1.550689000
H	-2.115291000	-3.841254000	1.077393000
N	-1.604140000	2.167979000	0.070273000
N	-1.517073000	-0.689991000	-0.096388000
V	-3.113149000	0.683573000	0.057749000
Cl	-4.656582000	-0.989683000	-0.250286000
Cl	-2.888606000	0.556362000	2.321665000
H	-2.514373000	-2.368701000	-0.909295000
H	-0.362223000	-2.769351000	-1.853069000
H	-2.703092000	3.854592000	0.709171000
H	-0.233932000	5.093761000	-0.093676000
C	0.526510000	0.797874000	0.080295000
C	1.169007000	0.610387000	1.472181000
H	0.403787000	0.485710000	2.250450000
H	1.816133000	-0.275254000	1.470775000
H	1.782874000	1.484980000	1.719242000
C	1.615015000	0.990343000	-0.985599000
H	2.283164000	0.122427000	-1.000867000
H	1.178636000	1.113160000	-1.986180000
H	2.216072000	1.876547000	-0.754746000
Cl	-4.793309000	2.254497000	0.170317000
N	-1.768250000	0.863831000	-2.513996000
O	-2.920877000	0.832159000	-1.843302000
O	-1.873032000	1.110038000	-3.673457000

VBOX-Cl2 (m4)

C	-0.039167000	-0.492829000	0.135728000
O	0.707929000	-1.595204000	0.269373000
C	-1.556250000	-2.123892000	-0.056845000
C	-0.204634000	-2.712769000	0.345913000
H	-0.195263000	-3.085239000	1.382051000
C	-0.123101000	1.995840000	0.308803000
O	0.530262000	3.105231000	0.671520000
C	-1.786304000	3.480826000	0.490724000
C	-0.438360000	4.180445000	0.678064000
H	-0.337708000	4.723459000	1.625019000
C	-2.674398000	4.061160000	-0.583476000
H	-2.173718000	4.039907000	-1.562316000
H	-3.606513000	3.480316000	-0.662268000
H	-2.946600000	5.100429000	-0.353690000
C	-2.717867000	-2.536944000	0.812296000
H	-2.541835000	-2.261593000	1.862287000

H	-3.646645000	-2.046294000	0.487492000
H	-2.874723000	-3.623200000	0.762559000
N	-1.394028000	2.101367000	0.155922000
N	-1.306830000	-0.670047000	-0.004583000
V	-2.826347000	0.702763000	-0.413444000
Cl	-4.095237000	0.968421000	1.633110000
H	-1.783868000	-2.364446000	-1.111808000
H	0.164812000	-3.502910000	-0.317623000
H	-2.347683000	3.428813000	1.439368000
H	-0.188689000	4.862800000	-0.148783000
C	0.742756000	0.792815000	0.043914000
C	1.929738000	0.768341000	1.008836000
H	1.602810000	0.678441000	2.053789000
H	2.586661000	-0.078081000	0.779353000
H	2.515652000	1.689297000	0.910805000
C	1.240989000	0.909856000	-1.413823000
H	1.902121000	0.067455000	-1.657508000
H	0.391558000	0.910583000	-2.112401000
H	1.810605000	1.840300000	-1.542298000
Cl	-2.418789000	0.723525000	-2.821022000

VBOX-Cl2-OH (m3)

C	-0.113297000	-0.514895000	0.043661000
O	0.609908000	-1.604590000	0.282874000
C	-1.661092000	-2.138986000	-0.004435000
C	-0.261883000	-2.746997000	0.107422000
H	-0.129436000	-3.407874000	0.973046000
C	-0.187677000	1.961300000	0.313964000
O	0.461783000	3.048432000	0.725669000
C	-1.853816000	3.439308000	0.530054000
C	-0.509787000	4.120858000	0.801360000
H	-0.427228000	4.580807000	1.792856000
C	-2.676443000	4.066140000	-0.570379000
H	-2.113907000	4.103128000	-1.514969000
H	-3.599711000	3.489002000	-0.735086000
H	-2.970225000	5.091205000	-0.306273000
C	-2.541016000	-2.362779000	1.204445000
H	-2.054255000	-1.990886000	2.118989000
H	-3.498009000	-1.837263000	1.089285000
H	-2.743821000	-3.434732000	1.336120000
N	-1.455573000	2.069525000	0.167155000
N	-1.369834000	-0.697385000	-0.177328000
V	-2.799996000	0.692165000	-0.760943000
Cl	-4.413591000	-0.803643000	-1.593006000
Cl	-4.062357000	1.045716000	1.269922000
H	-2.177975000	-2.492150000	-0.909128000
H	0.065135000	-3.276039000	-0.797927000
H	-2.463190000	3.365071000	1.446081000
H	-0.241322000	4.867626000	0.039920000
C	0.679648000	0.768317000	0.012570000
C	1.836905000	0.697224000	1.012878000
H	1.480912000	0.555100000	2.042157000
H	2.504634000	-0.133474000	0.760195000
H	2.420552000	1.623736000	0.975719000
C	1.231073000	0.945650000	-1.418469000
H	1.880323000	0.097734000	-1.674769000
H	0.409192000	1.006201000	-2.144200000
H	1.832146000	1.863332000	-1.473387000
O	-1.900074000	1.003093000	-2.344109000
H	-2.335991000	1.651181000	-2.919337000

VBOX-Cl2-F (m3)

C	-0.104688000	-0.517455000	0.056656000
O	0.615477000	-1.598201000	0.324154000
C	-1.654499000	-2.143997000	0.022865000
C	-0.260027000	-2.745566000	0.212837000
H	-0.152409000	-3.338437000	1.129872000
C	-0.171004000	1.958441000	0.295905000
O	0.476624000	3.040186000	0.714329000
C	-1.838977000	3.435762000	0.523599000
C	-0.493290000	4.115723000	0.789213000
H	-0.406018000	4.578412000	1.778758000
C	-2.673859000	4.075325000	-0.560002000
H	-2.125023000	4.112981000	-1.512191000
H	-3.602748000	3.506286000	-0.715769000
H	-2.954211000	5.100589000	-0.283029000
C	-2.592752000	-2.363694000	1.186770000
H	-2.171955000	-1.952310000	2.116595000
H	-3.560790000	-1.880147000	1.004759000
H	-2.765662000	-3.438747000	1.334485000
N	-1.439698000	2.068639000	0.146109000
N	-1.362067000	-0.702300000	-0.166137000
V	-2.780896000	0.690284000	-0.767275000
Cl	-4.228177000	-0.868877000	-1.706776000
Cl	-4.050979000	1.008606000	1.208920000
H	-2.120370000	-2.514835000	-0.901578000
H	0.086086000	-3.344259000	-0.639672000
H	-2.436120000	3.351787000	1.447473000
H	-0.224080000	4.857688000	0.023594000
C	0.690459000	0.762914000	-0.010477000
C	1.872847000	0.704739000	0.959531000
H	1.543242000	0.573742000	1.998774000
H	2.535772000	-0.127288000	0.699284000
H	2.453025000	1.631853000	0.896472000
C	1.200977000	0.917830000	-1.459255000
H	1.841178000	0.065339000	-1.721946000
H	0.362063000	0.968745000	-2.164361000
H	1.799269000	1.834566000	-1.546199000
F	-1.944075000	1.150411000	-2.326216000

VBOX-Cl2-N3 (m3)

C	-0.224103000	-0.501620000	-0.057720000
O	0.504189000	-1.611390000	-0.071014000
C	-1.788105000	-2.113652000	-0.067898000
C	-0.402073000	-2.721431000	-0.278703000
H	-0.144242000	-3.507748000	0.440636000
C	-0.223938000	1.993415000	0.198169000
O	0.464953000	3.088970000	0.491450000
C	-1.845261000	3.531088000	0.387304000
C	-0.473196000	4.196342000	0.507563000
H	-0.319067000	4.753348000	1.438107000
C	-2.756089000	4.101001000	-0.672946000
H	-2.269515000	4.099563000	-1.658939000
H	-3.681924000	3.508790000	-0.745301000
H	-3.040002000	5.133604000	-0.430346000
C	-2.453719000	-2.496266000	1.234395000

H	-1.814444000	-2.246169000	2.094482000
H	-3.411745000	-1.973060000	1.349842000
H	-2.646606000	-3.577662000	1.254847000
N	-1.495231000	2.130740000	0.090230000
N	-1.504029000	-0.657518000	-0.103058000
V	-2.998029000	0.750612000	-0.391076000
Cl	-4.735642000	-0.698299000	-0.978561000
Cl	-3.687805000	1.008684000	1.842326000
H	-2.456140000	-2.347982000	-0.909665000
H	-0.234817000	-3.096939000	-1.297783000
H	-2.370526000	3.520941000	1.357654000
H	-0.229960000	4.847972000	-0.344143000
C	0.603699000	0.758173000	-0.013279000
C	1.627800000	0.631048000	1.124793000
H	1.135066000	0.506545000	2.098331000
H	2.278456000	-0.233007000	0.950327000
H	2.256546000	1.527419000	1.165093000
C	1.335970000	0.912755000	-1.361559000
H	1.922390000	0.010941000	-1.577457000
H	0.633909000	1.080516000	-2.188897000
H	2.023808000	1.766343000	-1.314457000
N	-2.408827000	0.906572000	-2.329804000
N	-1.800174000	0.050742000	-2.909971000
N	-1.209799000	-0.757941000	-3.494108000

VBOX-Cl2-SCN (m3)

C	-0.190033000	-0.520130000	-0.031808000
O	0.538287000	-1.625821000	-0.034317000
C	-1.756868000	-2.134363000	-0.036248000
C	-0.364562000	-2.748616000	-0.183231000
H	-0.116832000	-3.481535000	0.594379000
C	-0.195520000	1.975789000	0.189635000
O	0.484400000	3.076932000	0.468030000
C	-1.824828000	3.511143000	0.362968000
C	-0.455799000	4.183719000	0.451786000
H	-0.296301000	4.772923000	1.360842000
C	-2.761849000	4.070109000	-0.678784000
H	-2.290135000	4.100845000	-1.671821000
H	-3.676806000	3.458288000	-0.741647000
H	-3.069390000	5.091341000	-0.418002000
C	-2.475160000	-2.511306000	1.239133000
H	-1.879586000	-2.242663000	2.124400000
H	-3.447387000	-2.006837000	1.309760000
H	-2.651141000	-3.595467000	1.261528000
N	-1.469055000	2.107757000	0.073081000
N	-1.470648000	-0.677548000	-0.078058000
V	-2.963169000	0.721700000	-0.334469000
Cl	-4.623115000	-0.689125000	-1.083914000
Cl	-3.620813000	0.948753000	1.864417000
H	-2.382617000	-2.375892000	-0.907873000
H	-0.180008000	-3.190796000	-1.170649000
H	-2.329681000	3.505846000	1.344468000
H	-0.219466000	4.802924000	-0.425531000
C	0.636392000	0.739723000	0.012389000
C	1.628718000	0.631102000	1.181292000
H	1.108406000	0.525532000	2.142696000
H	2.281379000	-0.237515000	1.040166000
H	2.257774000	1.527322000	1.221946000
C	1.404275000	0.874951000	-1.317147000
H	2.010871000	-0.022075000	-1.492084000
H	0.720811000	1.010827000	-2.165945000
H	2.076465000	1.740797000	-1.269514000
S	-2.205839000	1.122328000	-2.789099000
C	-1.704496000	-0.426908000	-3.096474000
N	-1.321029000	-1.520305000	-3.314300000

VBOX-Cl2-ONO2 (m3)

C	-0.135381000	-0.480023000	-0.032865000
O	0.602976000	-1.582438000	-0.036366000
C	-1.687602000	-2.104432000	-0.104976000
C	-0.288996000	-2.702255000	-0.256799000
H	-0.050088000	-3.473621000	0.485365000
C	-0.172440000	2.013153000	0.213308000
O	0.493661000	3.121493000	0.505560000
C	-1.819134000	3.530100000	0.358768000
C	-0.458376000	4.216788000	0.474724000
H	-0.323072000	4.805967000	1.387949000
C	-2.737176000	4.087000000	-0.702340000
H	-2.272920000	4.037484000	-1.697264000
H	-3.682958000	3.523926000	-0.733952000
H	-2.985775000	5.134499000	-0.486502000
C	-2.414470000	-2.512429000	1.156084000
H	-1.835065000	-2.252518000	2.054751000
H	-3.392829000	-2.018618000	1.219741000
H	-2.579697000	-3.598604000	1.157987000
N	-1.443599000	2.130712000	0.077471000
N	-1.410537000	-0.648103000	-0.111380000
V	-2.903724000	0.724153000	-0.445635000
Cl	-4.576845000	-0.670879000	-1.214147000
Cl	-3.646917000	0.928494000	1.754742000
H	-2.308888000	-2.324211000	-0.983773000
H	-0.081605000	-3.094543000	-1.261619000
H	-2.343663000	3.520646000	1.329745000
H	-0.213758000	4.842233000	-0.396116000
C	0.677057000	0.786352000	0.051686000
C	1.631077000	0.675075000	1.251899000
H	1.079656000	0.556746000	2.194358000
H	2.295324000	-0.187231000	1.125436000
H	2.250491000	1.576427000	1.321994000
C	1.489130000	0.943322000	-1.248278000
H	2.114311000	0.056833000	-1.409913000
H	0.839412000	1.079283000	-2.123459000
H	2.147282000	1.817664000	-1.170569000
O	-1.702681000	-0.854978000	-2.928229000
N	-1.807969000	0.330601000	-3.207551000
O	-2.313655000	1.154289000	-2.315855000
O	-1.477366000	0.826318000	-4.272178000

VBOX-Cl2-Me (m3)

C	-0.167771000	-0.536803000	0.011680000
O	0.562916000	-1.645706000	0.101440000
C	-1.723774000	-2.150809000	-0.042428000
C	-0.326278000	-2.767224000	-0.103608000
H	-0.128609000	-3.504389000	0.684396000
C	-0.187308000	1.960860000	0.216206000
O	0.502030000	3.063163000	0.517259000

C	-1.804477000	3.491016000	0.410754000
C	-0.435858000	4.165252000	0.487535000
H	-0.276862000	4.772337000	1.385508000
C	-2.758898000	4.085436000	-0.595209000
H	-2.328871000	4.094581000	-1.606934000
H	-3.700201000	3.515660000	-0.622774000
H	-3.008140000	5.120074000	-0.322451000
C	-2.494229000	-2.477278000	1.217551000
H	-1.925528000	-2.187309000	2.114105000
H	-3.456188000	-1.948619000	1.234836000
H	-2.691318000	-3.556943000	1.271286000
N	-1.454988000	2.093996000	0.093658000
N	-1.439484000	-0.697156000	-0.122877000
V	-2.955165000	0.688873000	-0.445437000
Cl	-4.628755000	-0.759837000	-1.180572000
Cl	-3.668996000	1.015242000	1.832942000
H	-2.321858000	-2.425427000	-0.924005000
H	-0.080329000	-3.211555000	-1.077620000
H	-2.292981000	3.464736000	1.400142000
H	-0.208067000	4.774632000	-0.400470000
C	0.653094000	0.726821000	0.037421000
C	1.668665000	0.644285000	1.186777000
H	1.168493000	0.571954000	2.162101000
H	2.313626000	-0.232562000	1.062249000
H	2.305882000	1.535578000	1.190375000
C	1.396239000	0.837279000	-1.308547000
H	2.074211000	-0.016553000	-1.434156000
H	0.696634000	0.853523000	-2.154882000
H	1.995293000	1.756652000	-1.332107000
C	-2.370425000	1.167358000	-2.430706000
H	-1.466883000	1.786117000	-2.549959000
H	-2.197127000	0.203288000	-2.944023000
H	-3.206946000	1.672241000	-2.949823000

VBOX-Cl2-NO2 (m3)

C	-0.158326000	-0.495664000	-0.018952000
O	0.582027000	-1.594805000	-0.076524000
C	-1.713931000	-2.116310000	-0.028862000
C	-0.312578000	-2.731610000	-0.046673000
H	-0.081504000	-3.312835000	0.856836000
C	-0.189777000	2.001298000	0.183900000
O	0.479821000	3.103856000	0.483653000
C	-1.833331000	3.512475000	0.418084000
C	-0.472084000	4.200690000	0.478221000
H	-0.302446000	4.797525000	1.380378000
C	-2.818875000	4.105727000	-0.556617000
H	-2.401185000	4.153092000	-1.570254000
H	-3.739400000	3.502827000	-0.595553000
H	-3.101199000	5.119642000	-0.242432000
C	-2.577289000	-2.553767000	1.129930000
H	-2.086916000	-2.338920000	2.091001000
H	-3.545002000	-2.035119000	1.112348000
H	-2.766807000	-3.634605000	1.077159000
N	-1.465484000	2.123006000	0.072715000
N	-1.434343000	-0.664754000	0.019533000
V	-2.935630000	0.708519000	-0.335799000
Cl	-4.517266000	-0.668503000	-1.311461000
Cl	-3.767476000	0.980354000	1.824028000
H	-2.239461000	-2.304287000	-0.979146000
H	-0.101908000	-3.347779000	-0.928707000
H	-2.293674000	3.460792000	1.420139000
H	-0.259182000	4.816508000	-0.407944000
C	0.654533000	0.774447000	-0.009589000
C	1.674368000	0.683811000	1.137789000
H	1.176680000	0.590912000	2.112407000
H	2.322158000	-0.187943000	0.993128000
H	2.306263000	1.578765000	1.152822000
C	1.396317000	0.913313000	-1.353630000
H	2.043271000	0.041901000	-1.513168000
H	0.702298000	0.990157000	-2.200562000
H	2.028757000	1.809731000	-1.334107000
N	-2.074411000	0.852898000	-2.298843000
O	-1.448880000	-0.083629000	-2.797702000
O	-2.252972000	1.905897000	-2.912022000

VBOX-Cl2-ONO (m3)

C	-0.198438000	-0.507613000	-0.176290000
O	0.533438000	-1.608492000	-0.303000000
C	-1.751578000	-2.130035000	-0.173797000
C	-0.376994000	-2.717343000	-0.491478000
H	-0.071142000	-3.524660000	0.185200000
C	-0.189264000	1.996290000	0.019485000
O	0.521979000	3.116699000	0.052949000
C	-1.781545000	3.560285000	0.253452000
C	-0.414644000	4.223090000	0.069547000
H	-0.125137000	4.898263000	0.882198000
C	-2.847934000	4.007354000	-0.718776000
H	-2.509364000	3.903262000	-1.759347000
H	-3.763978000	3.409250000	-0.592242000
H	-3.112565000	5.058701000	-0.544994000
C	-2.334275000	-2.583746000	1.145486000
H	-1.648252000	-2.360019000	1.976503000
H	-3.290198000	-2.081257000	1.340318000
H	-2.510411000	-3.668159000	1.127861000
N	-1.463626000	2.128457000	0.101008000
N	-1.476593000	-0.672660000	-0.144280000
V	-2.991888000	0.727945000	-0.150401000
Cl	-4.779614000	-0.679327000	-0.638503000
Cl	-3.288224000	0.843697000	2.165864000
H	-2.464828000	-2.328089000	-0.987297000
H	-0.270991000	-3.059992000	-1.529399000
H	-2.149729000	3.690259000	1.286022000
H	-0.316407000	4.753608000	-0.888867000
C	0.629371000	0.738155000	-0.005291000
C	1.315357000	0.628805000	1.374840000
H	0.571623000	0.579748000	2.182759000
H	1.937411000	-0.273623000	1.415105000
H	1.959663000	1.500167000	1.544023000
C	1.683890000	0.820119000	-1.115820000
H	2.327400000	-0.066731000	-1.091734000
H	1.216978000	0.887951000	-2.107366000
H	2.316300000	1.703121000	-0.967375000
N	-1.440493000	1.155815000	-2.582035000
O	-2.648273000	1.032076000	-2.073027000
O	-1.399684000	1.152137000	-3.786034000

CrBOX-Cl3 (m4)

C	-0.139904000	-0.485585000	0.021677000
O	0.578210000	-1.593600000	0.083376000
C	-1.714984000	-2.102248000	-0.055964000
C	-0.315740000	-2.714596000	-0.131896000
H	-0.112968000	-3.458560000	0.647523000
C	-0.163049000	2.000207000	0.244263000
O	0.492769000	3.107665000	0.568799000
C	-1.825174000	3.500190000	0.394261000
C	-0.469846000	4.194298000	0.543567000
H	-0.354162000	4.767631000	1.469633000
C	-2.725944000	4.061388000	-0.678091000
H	-2.242519000	4.017123000	-1.663704000
H	-3.666561000	3.491969000	-0.730740000
H	-2.983889000	5.106184000	-0.459575000
C	-2.489092000	-2.473448000	1.187381000
H	-1.925059000	-2.214531000	2.095854000
H	-3.454221000	-1.952740000	1.216167000
H	-2.675235000	-3.556222000	1.196513000
N	-1.429577000	2.112633000	0.093906000
N	-1.415457000	-0.648148000	-0.095462000
Cr	-2.823606000	0.717377000	-0.465835000
Cl	-4.546793000	-0.700615000	-1.096085000
Cl	-3.615735000	0.888739000	1.737384000
H	-2.303769000	-2.341101000	-0.952987000
H	-0.070863000	-3.143570000	-1.112152000
H	-2.367842000	3.468368000	1.354239000
H	-0.219105000	4.838978000	-0.311466000
C	0.683199000	0.774912000	0.065281000
C	1.689223000	0.676291000	1.220332000
H	1.183015000	0.579986000	2.190149000
H	2.342601000	-0.191964000	1.080250000
H	2.317969000	1.573280000	1.244094000
C	1.426593000	0.904927000	-1.278903000
H	2.082294000	0.038543000	-1.431566000
H	0.716945000	0.969124000	-2.114254000
H	2.048093000	1.809314000	-1.271175000
Cl	-2.087146000	0.971819000	-2.674217000

CrBOX-Cl3-OH (m3)

C	-0.176072000	-0.517573000	-0.013875000
O	0.615920000	-1.572068000	0.107414000
C	-1.640804000	-2.210495000	0.113905000
C	-0.218100000	-2.749881000	-0.012996000
H	0.068816000	-3.447325000	0.782642000
C	-0.350717000	1.938449000	0.287465000
O	0.221134000	2.949537000	0.928405000
C	-1.948942000	3.499673000	0.286863000
C	-0.853425000	3.868676000	1.272510000
H	-1.119641000	3.675816000	2.321661000
C	-1.900562000	4.331344000	-0.978324000
H	-0.879116000	4.356477000	-1.388428000
H	-2.574141000	3.928704000	-1.743239000
H	-2.202692000	5.365848000	-0.764530000
C	-2.286770000	-2.495751000	1.451136000
H	-1.678591000	-2.095431000	2.276169000
H	-3.286136000	-2.048572000	1.512475000
H	-2.382514000	-3.581192000	1.593262000
N	-1.587746000	2.095654000	-0.027696000
N	-1.441824000	-0.750822000	-0.084426000
Cr	-2.962497000	0.608401000	-0.579942000
Cl	-4.461483000	-1.076553000	-0.865217000
Cl	-3.515461000	0.856137000	1.692433000
H	-2.273668000	-2.587415000	-0.700369000
H	-0.002064000	-3.206541000	-0.988271000
H	-2.944720000	3.514891000	0.750540000
H	-0.473477000	4.890765000	1.169256000
C	0.556061000	0.796392000	-0.077314000
C	1.772305000	0.759080000	0.849840000
H	1.484809000	0.596038000	1.896761000
H	2.453889000	-0.043324000	0.548636000
H	2.317430000	1.707102000	0.786113000
C	1.011277000	1.024125000	-1.534914000
H	1.700994000	0.225123000	-1.837035000
H	0.155366000	1.036229000	-2.221914000
H	1.543892000	1.981905000	-1.610158000
Cl	-4.548626000	2.190386000	-1.237257000
O	-2.239849000	0.668547000	-2.216918000
H	-2.697162000	1.352191000	-2.744139000

CrBOX-Cl4 (m3)

C	-0.244488000	-0.472748000	-0.069475000
O	0.530753000	-1.529563000	-0.281852000
C	-1.709343000	-2.167874000	-0.107297000
C	-0.348073000	-2.634573000	-0.606295000
H	0.030449000	-3.530582000	-0.101971000
C	-0.338814000	2.018245000	0.242217000
O	0.353720000	3.127077000	0.471287000
C	-1.892990000	3.620656000	0.084122000
C	-0.608598000	4.190268000	0.669888000
H	-0.664372000	4.385184000	1.749840000
C	-2.175370000	4.090788000	-1.326598000
H	-1.314357000	3.896365000	-1.984106000
H	-3.049721000	3.584279000	-1.753030000
H	-2.369684000	5.172172000	-1.327360000
C	-2.075524000	-2.732681000	1.247931000
H	-1.283567000	-2.525893000	1.983875000
H	-3.013271000	-2.305014000	1.619402000
H	-2.195603000	-3.822371000	1.174066000
N	-1.610124000	2.160661000	0.095946000
N	-1.510418000	-0.696142000	-0.028932000
Cr	-3.089925000	0.693701000	-0.013122000
Cl	-4.675016000	-0.974768000	-0.142142000
Cl	-2.862882000	0.533646000	2.267681000
H	-2.498065000	-2.375541000	-0.842199000
H	-0.302814000	-2.781732000	-1.694468000
H	-2.750452000	3.818953000	0.739182000
H	-0.238948000	5.085313000	0.157285000
C	0.526352000	0.797283000	0.134988000
C	1.338542000	0.639467000	1.433262000
H	0.679530000	0.484618000	2.298696000
H	2.016479000	-0.218487000	1.352091000
H	1.941316000	1.538113000	1.609074000
C	1.464695000	1.011113000	-1.066930000
H	2.144134000	0.157136000	-1.168845000
H	0.892880000	1.118902000	-1.999195000

H	2.066614000	1.915058000	-0.918686000
Cl	-2.720505000	0.651106000	-2.282250000
Cl	-4.741959000	2.239968000	0.009051000

CrBOX-Cl3-F (m3)

C	-0.193243000	-0.499153000	0.036092000
O	0.601710000	-1.555497000	0.072377000
C	-1.657473000	-2.206703000	0.078592000
C	-0.229344000	-2.732809000	-0.063916000
H	0.063716000	-3.440797000	0.720536000
C	-0.349573000	1.973831000	0.315700000
O	0.262225000	3.026531000	0.837923000
C	-1.927204000	3.553562000	0.245580000
C	-0.776017000	4.000043000	1.131785000
H	-0.992247000	3.929889000	2.207061000
C	-1.945833000	4.259222000	-1.095000000
H	-0.951890000	4.219248000	-1.566680000
H	-2.668753000	3.801863000	-1.781286000
H	-2.215379000	5.316308000	-0.964964000
C	-2.331737000	-2.623181000	1.365264000
H	-1.756379000	-2.290961000	2.242009000
H	-3.344723000	-2.211287000	1.433268000
H	-2.401183000	-3.719368000	1.398604000
N	-1.599713000	2.118779000	0.043155000
N	-1.460173000	-0.735247000	-0.000897000
Cr	-2.988558000	0.637674000	-0.415871000
Cl	-4.485557000	-1.064798000	-0.857889000
Cl	-3.395503000	0.593763000	1.852398000
H	-2.273796000	-2.506919000	-0.779909000
H	-0.013087000	-3.174365000	-1.045603000
H	-2.893694000	3.638503000	0.760341000
H	-0.384354000	4.996231000	0.899762000
C	0.543235000	0.810551000	-0.006719000
C	1.737771000	0.765292000	0.948957000
H	1.419538000	0.614084000	1.988948000
H	2.415256000	-0.048643000	0.670563000
H	2.296608000	1.705580000	0.892660000
C	1.026050000	1.023273000	-1.459311000
H	1.713969000	0.217191000	-1.744584000
H	0.179524000	1.036551000	-2.157946000
H	1.564558000	1.977228000	-1.535658000
Cl	-4.601385000	2.162909000	-0.851088000
F	-2.324100000	0.588121000	-2.070936000

CrBOX-Cl3-N3 (m3)

C	-0.242790000	-0.489723000	-0.052355000
O	0.546453000	-1.553622000	-0.118571000
C	-1.698010000	-2.194983000	-0.080486000
C	-0.301360000	-2.683819000	-0.435650000
H	0.040842000	-3.543771000	0.150676000
C	-0.359389000	1.999829000	0.269428000
O	0.308626000	3.090195000	0.626418000
C	-1.918749000	3.598267000	0.172889000
C	-0.682499000	4.114643000	0.892148000
H	-0.800648000	4.179508000	1.982804000
C	-2.082170000	4.174569000	-1.217613000
H	-1.160002000	4.040002000	-1.804033000
H	-2.908213000	3.693446000	-1.754328000
H	-2.292875000	5.251158000	-1.157693000
C	-2.196384000	-2.664476000	1.266830000
H	-1.489846000	-2.399071000	2.067701000
H	-3.168265000	-2.214761000	1.500768000
H	-2.309104000	-3.757344000	1.257004000
N	-1.624471000	2.144683000	0.082433000
N	-1.511271000	-0.714935000	-0.097527000
Cr	-3.083660000	0.684823000	-0.239464000
Cl	-4.576427000	-1.015321000	-0.700451000
Cl	-3.198207000	0.560051000	2.058586000
H	-2.419212000	-2.465765000	-0.861904000
H	-0.172935000	-2.897869000	-1.506834000
H	-2.827098000	3.742677000	0.771891000
H	-0.300543000	5.067156000	0.508548000
C	0.516565000	0.801583000	0.047289000
C	1.531257000	0.682027000	1.193601000
H	1.025469000	0.539671000	2.158380000
H	2.199848000	-0.168354000	1.021087000
H	2.142506000	1.589022000	1.251541000
C	1.242806000	1.025596000	-1.296283000
H	1.934288000	0.196665000	-1.490962000
H	0.528402000	1.092272000	-2.129203000
H	1.822831000	1.956144000	-1.258831000
Cl	-4.764866000	2.229558000	-0.409730000
N	-2.518310000	0.800026000	-2.129283000
N	-2.321948000	-0.157668000	-2.830242000
N	-2.102875000	-1.044719000	-3.535820000

CrBOX-Cl3-SCN (m3)

C	-0.295791000	-0.550337000	-0.191458000
O	0.406466000	-1.576933000	-0.633811000
C	-1.762200000	-2.237035000	-0.110628000
C	-0.545649000	-2.634134000	-0.928335000
H	-0.094900000	-3.588640000	-0.636784000
C	-0.327539000	1.922248000	0.030664000
O	0.375329000	3.038602000	0.109999000
C	-1.897237000	3.510874000	-0.185390000
C	-0.570332000	4.128993000	0.240596000
H	-0.549058000	4.453290000	1.289779000
C	-2.310277000	3.887844000	-1.590748000
H	-1.529374000	3.618101000	-2.318094000
H	-3.248971000	3.400601000	-1.880997000
H	-2.466832000	4.973749000	-1.648872000
C	-1.795330000	-2.878476000	1.260097000
H	-0.838555000	-2.731531000	1.784823000
H	-2.596456000	-2.456643000	1.878540000
H	-1.967524000	-3.959464000	1.166529000
N	-1.608503000	2.052668000	-0.079621000
N	-1.552568000	-0.766666000	0.031407000
Cr	-3.069528000	0.601112000	0.316735000
Cl	-4.612819000	-1.052751000	0.630672000
Cl	-2.634877000	1.009818000	2.515549000
H	-2.695298000	-2.422786000	-0.657798000
H	-0.716134000	-2.623323000	-2.014345000
H	-2.692416000	3.756618000	0.530846000
H	-0.229950000	4.950294000	-0.400032000
C	0.503420000	0.676552000	0.130639000
C	0.960783000	0.519996000	1.600047000

H	0.106349000	0.458815000	2.286896000
H	1.561998000	-0.393118000	1.700423000
H	1.586346000	1.375397000	1.884595000
C	1.728532000	0.797446000	-0.780546000
H	2.374710000	-0.078391000	-0.655817000
H	1.441067000	0.871261000	-1.836806000
H	2.309482000	1.686013000	-0.510288000
Cl	-4.811520000	2.154030000	0.126226000
S	-3.451813000	0.544863000	-2.180559000
C	-1.891616000	0.535602000	-2.717170000
N	-0.771127000	0.529973000	-3.083668000

CrBOX-Cl3-ONO2 (m3)

C	-0.208189000	-0.436739000	-0.181289000
O	0.516279000	-1.437387000	-0.633280000
C	-1.687373000	-2.119070000	-0.295740000
C	-0.402644000	-2.494196000	-1.016605000
H	0.031870000	-3.449666000	-0.704194000
C	-0.261507000	2.028585000	0.122832000
O	0.441658000	3.146206000	0.154183000
C	-1.833227000	3.601646000	-0.160981000
C	-0.502606000	4.245107000	0.212689000
H	-0.475194000	4.644257000	1.235281000
C	-2.262887000	3.870274000	-1.586646000
H	-1.467412000	3.599075000	-2.297815000
H	-3.174570000	3.314958000	-1.844286000
H	-2.477147000	4.939649000	-1.718530000
C	-1.879613000	-2.851642000	1.016702000
H	-0.991046000	-2.747467000	1.658056000
H	-2.748881000	-2.456749000	1.554236000
H	-2.041739000	-3.921112000	0.826595000
N	-1.542467000	2.154518000	0.030354000
N	-1.475436000	-0.674016000	-0.036733000
Cr	-2.991396000	0.665453000	0.199862000
Cl	-4.605958000	-0.984420000	0.231577000
Cl	-2.651048000	0.574036000	2.447770000
H	-2.566561000	-2.238965000	-0.942526000
H	-0.481319000	-2.467913000	-2.112397000
H	-2.622212000	3.897270000	0.541369000
H	-0.164619000	5.016919000	-0.488148000
C	0.568566000	0.781544000	0.219364000
C	0.976637000	0.581347000	1.696744000
H	0.098885000	0.479266000	2.348479000
H	1.594395000	-0.321458000	1.789386000
H	1.570500000	1.441028000	2.032403000
C	1.819469000	0.934058000	-0.652180000
H	2.456937000	0.049039000	-0.553017000
H	1.565645000	1.063659000	-1.712401000
H	2.399187000	1.803817000	-0.325714000
Cl	-4.638023000	2.233628000	0.307884000
O	-1.045222000	0.672725000	-2.526098000
N	-2.238150000	0.867025000	-2.725494000
O	-3.101090000	0.584111000	-1.791641000
O	-2.696465000	1.286804000	-3.769849000

CrBOX-Cl3-Me (m3)

C	-0.219108000	-0.450355000	-0.180405000
O	0.546533000	-1.497764000	-0.469189000
C	-1.678494000	-2.135595000	-0.236135000
C	-0.351048000	-2.576649000	-0.829467000
H	0.047476000	-3.507655000	-0.410845000
C	-0.284325000	2.031545000	0.159202000
O	0.424572000	3.141035000	0.334696000
C	-1.827148000	3.639263000	-0.023219000
C	-0.523107000	4.220922000	0.505905000
H	-0.551232000	4.465918000	1.576841000
C	-2.159763000	4.080971000	-1.432053000
H	-1.326719000	3.871767000	-2.120434000
H	-3.060742000	3.577466000	-1.802955000
H	-2.347792000	5.163487000	-1.449475000
C	-1.946891000	-2.708262000	1.139188000
H	-1.092409000	-2.530622000	1.810697000
H	-2.834075000	-2.245964000	1.587770000
H	-2.109809000	-3.792984000	1.073232000
N	-1.556662000	2.176842000	0.017000000
N	-1.488743000	-0.663225000	-0.133503000
Cr	-3.048834000	0.706494000	0.139220000
Cl	-4.638299000	-0.956633000	-0.123025000
Cl	-2.681498000	0.615623000	2.414887000
H	-2.517245000	-2.345112000	-0.911686000
H	-0.359877000	-2.643406000	-1.926996000
H	-2.665442000	3.855701000	0.650757000
H	-0.156000000	5.088784000	-0.053728000
C	0.555709000	0.787466000	0.164639000
C	1.092177000	0.579494000	1.597275000
H	0.266230000	0.460214000	2.311646000
H	1.724574000	-0.316592000	1.635007000
H	1.699604000	1.442386000	1.897432000
C	1.719524000	0.974421000	-0.818702000
H	2.376685000	0.098015000	-0.798376000
H	1.358051000	1.112047000	-1.847315000
H	2.314680000	1.851147000	-0.540218000
Cl	-4.718237000	2.307912000	0.070161000
C	-2.856159000	0.644236000	-2.312399000
H	-1.928090000	1.193317000	-2.489879000
H	-2.848256000	-0.424774000	-2.536127000
H	-3.797526000	1.173505000	-2.484026000

CrBOX-Cl3-NO2 (m3)

C	-0.244304000	-0.470644000	-0.156727000
O	0.516509000	-1.513697000	-0.457115000
C	-1.709506000	-2.159640000	-0.194942000
C	-0.377265000	-2.607459000	-0.776866000
H	0.027856000	-3.521840000	-0.328687000
C	-0.298080000	2.018897000	0.147411000
O	0.415098000	3.127588000	0.278724000
C	-1.852119000	3.629073000	0.013391000
C	-0.521227000	4.215447000	0.467155000
H	-0.497933000	4.486120000	1.531523000
C	-2.260026000	4.058214000	-1.379070000
H	-1.480620000	3.810389000	-2.116115000
H	-3.202709000	3.586867000	-1.682959000
H	-2.411875000	5.146030000	-1.402068000
C	-2.015445000	-2.753445000	1.162610000
H	-1.186706000	-2.574138000	1.864764000
H	-2.925940000	-2.316459000	1.590331000

H	-2.160058000	-3.839107000	1.075155000
N	-1.576329000	2.165941000	0.057428000
N	-1.511527000	-0.688797000	-0.075230000
Cr	-3.051629000	0.692065000	0.183015000
Cl	-4.605969000	-0.930320000	-0.221961000
Cl	-2.675008000	0.603868000	2.422504000
H	-2.531079000	-2.352125000	-0.895845000
H	-0.387736000	-2.710801000	-1.871063000
H	-2.650374000	3.853237000	0.732195000
H	-0.176869000	5.067152000	-0.130344000
C	0.539173000	0.773577000	0.143669000
C	1.124137000	0.590493000	1.560832000
H	0.326200000	0.489634000	2.308738000
H	1.752419000	-0.308461000	1.592582000
H	1.747305000	1.454677000	1.821532000
C	1.672358000	0.942094000	-0.878799000
H	2.333498000	0.069051000	-0.856023000
H	1.282381000	1.054956000	-1.899377000
H	2.271371000	1.826466000	-0.635917000
Cl	-4.715071000	2.266099000	0.225786000
N	-2.689749000	0.814552000	-2.154091000
O	-1.577210000	0.644311000	-2.583875000
O	-3.701443000	1.069026000	-2.755234000

CrBOX-Cl3-ONO (m3)

C	-0.254077000	-0.444615000	-0.216242000
O	0.498008000	-1.483115000	-0.546759000
C	-1.715981000	-2.132498000	-0.249506000
C	-0.409263000	-2.560357000	-0.894872000
H	0.006789000	-3.496122000	-0.505790000
C	-0.318833000	2.030478000	0.133123000
O	0.382016000	3.145208000	0.269681000
C	-1.884466000	3.621190000	-0.030286000
C	-0.568457000	4.225049000	0.441385000
H	-0.564149000	4.497581000	1.505514000
C	-2.267480000	4.021990000	-1.438856000
H	-1.455841000	3.798317000	-2.148732000
H	-3.181115000	3.506692000	-1.762206000
H	-2.461022000	5.102601000	-1.481488000
C	-1.936651000	-2.724583000	1.126289000
H	-1.056654000	-2.560976000	1.767761000
H	-2.804602000	-2.262353000	1.611280000
H	-2.108205000	-3.807106000	1.049756000
N	-1.599205000	2.161648000	0.034936000
N	-1.524273000	-0.661545000	-0.124543000
Cr	-3.084744000	0.685739000	0.195731000
Cl	-4.668180000	-0.994555000	0.011979000
Cl	-2.779166000	0.600171000	2.458470000
H	-2.576270000	-2.327872000	-0.901242000
H	-0.452312000	-2.606184000	-1.992555000
H	-2.701925000	3.846666000	0.665858000
H	-0.222440000	5.078398000	-0.152734000
C	0.526493000	0.791340000	0.118438000
C	1.088422000	0.578157000	1.540293000
H	0.275974000	0.452688000	2.268908000
H	1.723488000	-0.316373000	1.562171000
H	1.698478000	1.441531000	1.833219000
C	1.663971000	0.984358000	-0.893645000
H	2.323428000	0.109656000	-0.892839000
H	1.271216000	1.123417000	-1.911161000
H	2.263645000	1.861800000	-0.628298000
Cl	-4.764737000	2.274347000	0.192439000
N	-1.937385000	0.839226000	-2.520846000
O	-3.031869000	0.779120000	-1.979009000
O	-1.683016000	0.933240000	-3.676816000

CrBOX-Cl2 (m5)

C	0.038999000	-0.507945000	0.057264000
O	0.764745000	-1.516064000	0.525650000
C	-1.452378000	-2.174961000	0.033428000
C	-0.091429000	-2.689261000	0.520559000
H	-0.109711000	-3.091586000	1.542206000
C	-0.200453000	1.916236000	0.391831000
O	0.379149000	2.926349000	1.029832000
C	-1.871608000	3.376720000	0.583055000
C	-0.694683000	3.809054000	1.450947000
H	-0.852937000	3.639710000	2.525572000
C	-2.070580000	4.242136000	-0.643450000
H	-1.136216000	4.329191000	-1.219871000
H	-2.845869000	3.814362000	-1.292576000
H	-2.384855000	5.254905000	-0.354516000
C	-2.577797000	-2.290054000	1.036681000
H	-2.322457000	-1.784719000	1.980189000
H	-3.492783000	-1.832640000	0.630440000
H	-2.797490000	-3.343343000	1.260374000
N	-1.465877000	2.012768000	0.168532000
N	-1.175703000	-0.760124000	-0.270223000
Cr	-2.693436000	0.583115000	-0.790558000
Cl	-4.665942000	1.859424000	-0.494829000
H	-1.754327000	-2.662039000	-0.906315000
H	0.368688000	-3.431312000	-0.143744000
H	-2.806052000	3.314100000	1.157602000
H	-0.366265000	4.842801000	1.293150000
C	0.720861000	0.825998000	-0.105846000
C	2.063302000	0.855602000	0.613631000
H	1.954350000	0.691597000	1.693274000
H	2.722503000	0.075834000	0.214648000
H	2.554585000	1.823820000	0.463257000
C	0.907497000	1.066991000	-1.618347000
H	1.559338000	0.294016000	-2.047259000
H	-0.060610000	1.041619000	-2.140847000
H	1.373612000	2.046937000	-1.790559000
Cl	-3.616835000	-1.040292000	-2.258141000

CrBOX-Cl2-OH (m4)

C	-0.116290000	-0.493136000	0.070039000
O	0.605365000	-1.582513000	0.276253000
C	-1.672005000	-2.124134000	-0.026626000
C	-0.276067000	-2.726461000	0.165787000
H	-0.174558000	-3.312614000	1.088224000
C	-0.163199000	1.993574000	0.254849000
O	0.474257000	3.082062000	0.667877000
C	-1.839168000	3.473410000	0.435654000
C	-0.494231000	4.164050000	0.670685000
H	-0.412406000	4.686552000	1.629924000
C	-2.707168000	4.100359000	-0.627798000

H	-2.178345000	4.164170000	-1.589964000
H	-3.625182000	3.511580000	-0.773937000
H	-3.007334000	5.115202000	-0.334587000
C	-2.633947000	-2.447768000	1.090932000
H	-2.243753000	-2.106702000	2.060699000
H	-3.607187000	-1.972931000	0.919517000
H	-2.777975000	-3.536303000	1.141840000
N	-1.425618000	2.108010000	0.064826000
N	-1.378551000	-0.672307000	-0.126876000
Cr	-2.757812000	0.703041000	-0.636341000
Cl	-4.366024000	-0.760154000	-1.499168000
Cl	-3.741295000	0.813134000	1.558906000
H	-2.108848000	-2.431826000	-0.988274000
H	0.071924000	-3.331910000	-0.680270000
H	-2.414029000	3.378840000	1.372287000
H	-0.218242000	4.855911000	-0.138693000
C	0.689941000	0.780205000	0.008709000
C	1.836189000	0.733400000	1.021922000
H	1.465064000	0.647057000	2.051780000
H	2.490235000	-0.120057000	0.814656000
H	2.437170000	1.646379000	0.947437000
C	1.249407000	0.882151000	-1.426551000
H	1.925444000	0.040436000	-1.625080000
H	0.438019000	0.863627000	-2.166573000
H	1.818550000	1.813912000	-1.541515000
O	-2.194947000	1.119076000	-2.366881000
H	-1.548522000	1.837666000	-2.351811000

CrBOX-Cl2-F (m4)

C	-0.110469000	-0.485311000	0.076313000
O	0.613290000	-1.577455000	0.246756000
C	-1.670654000	-2.118310000	-0.020318000
C	-0.276057000	-2.719474000	0.183010000
H	-0.171688000	-3.270746000	1.126778000
C	-0.152648000	1.999308000	0.295354000
O	0.501483000	3.101949000	0.637484000
C	-1.824217000	3.487078000	0.473628000
C	-0.471997000	4.179109000	0.680068000
H	-0.367710000	4.686886000	1.645363000
C	-2.669909000	4.047081000	-0.646333000
H	-2.135216000	3.998593000	-1.605605000
H	-3.605887000	3.476455000	-0.744557000
H	-2.937247000	5.093443000	-0.447954000
C	-2.651461000	-2.471039000	1.070797000
H	-2.282851000	-2.148103000	2.055007000
H	-3.625676000	-2.001586000	0.889825000
H	-2.788483000	-3.561260000	1.096261000
N	-1.423231000	2.102297000	0.179312000
N	-1.378571000	-0.664374000	-0.088168000
Cr	-2.740218000	0.717961000	-0.576822000
Cl	-4.312655000	-0.739726000	-1.519519000
Cl	-3.786474000	0.781852000	1.539959000
H	-2.088204000	-2.405391000	-0.997089000
H	0.061907000	-3.358591000	-0.641370000
H	-2.410553000	3.465616000	1.407463000
H	-0.218898000	4.884776000	-0.124038000
C	0.694679000	0.788679000	0.024404000
C	1.850144000	0.722731000	1.026379000
H	1.488157000	0.606917000	2.056757000
H	2.508042000	-0.120908000	0.792123000
H	2.444869000	1.641141000	0.970661000
C	1.235145000	0.921607000	-1.415847000
H	1.874967000	0.062864000	-1.656629000
H	0.406960000	0.968759000	-2.134598000
H	1.839291000	1.834116000	-1.501866000
F	-1.995294000	1.067079000	-2.222417000

CrBOX-Cl2-N3 (m4)

C	-0.132219000	-0.488850000	0.065273000
O	0.591280000	-1.591990000	0.138296000
C	-1.697459000	-2.116090000	-0.005861000
C	-0.294801000	-2.717824000	-0.084959000
H	-0.085451000	-3.467459000	0.687064000
C	-0.160388000	2.003454000	0.271079000
O	0.494419000	3.112119000	0.589676000
C	-1.823149000	3.508370000	0.385911000
C	-0.468841000	4.199180000	0.569493000
H	-0.367822000	4.753486000	1.508941000
C	-2.687560000	4.055278000	-0.724332000
H	-2.158417000	4.037059000	-1.687891000
H	-3.610888000	3.464107000	-0.821887000
H	-2.980628000	5.092010000	-0.512261000
C	-2.472156000	-2.479717000	1.238872000
H	-1.921249000	-2.196088000	2.147939000
H	-3.447419000	-1.977010000	1.249102000
H	-2.641036000	-3.565004000	1.263906000
N	-1.426542000	2.115933000	0.114906000
N	-1.407608000	-0.658736000	-0.057814000
Cr	-2.817615000	0.714078000	-0.435215000
Cl	-4.488619000	-0.745869000	-1.135138000
Cl	-3.655272000	0.914282000	1.745188000
H	-2.284704000	-2.370867000	-0.899753000
H	-0.048297000	-3.134462000	-1.070721000
H	-2.397877000	3.493234000	1.327159000
H	-0.203429000	4.860982000	-0.267361000
C	0.686102000	0.776099000	0.100040000
C	1.694734000	0.686600000	1.253662000
H	1.189581000	0.592190000	2.224170000
H	2.351717000	-0.179394000	1.116958000
H	2.319516000	1.586356000	1.273370000
C	1.428574000	0.897951000	-1.245916000
H	2.071218000	0.022497000	-1.402061000
H	0.725919000	0.976910000	-2.086350000
H	2.063768000	1.792683000	-1.237470000
N	-2.132310000	0.861898000	-2.341993000
N	-1.765281000	-0.123191000	-2.920165000
N	-1.381231000	-1.051740000	-3.500021000

CrBOX-Cl2-SCN (m4)

C	-0.106678000	-0.479910000	0.007513000
O	0.618816000	-1.580337000	0.057109000
C	-1.669537000	-2.113383000	-0.058033000
C	-0.266901000	-2.708434000	-0.166100000
H	-0.042132000	-3.464183000	0.595283000
C	-0.151071000	2.008950000	0.235476000
O	0.490949000	3.118647000	0.565116000

C	-1.825149000	3.504959000	0.346540000
C	-0.476438000	4.202511000	0.537351000
H	-0.382896000	4.757907000	1.476682000
C	-2.693473000	4.055262000	-0.757009000
H	-2.156657000	4.071515000	-1.716790000
H	-3.602676000	3.445244000	-0.873492000
H	-3.013639000	5.079910000	-0.526772000
C	-2.413010000	-2.483579000	1.203482000
H	-1.846101000	-2.195220000	2.101136000
H	-3.394870000	-1.995073000	1.237047000
H	-2.568094000	-3.570811000	1.230367000
N	-1.417692000	2.116363000	0.060026000
N	-1.383581000	-0.653339000	-0.111343000
Cr	-2.804603000	0.706405000	-0.439252000
Cl	-4.474608000	-0.711454000	-1.143833000
Cl	-3.592379000	0.883587000	1.737025000
H	-2.270096000	-2.367961000	-0.942387000
H	-0.034527000	-3.113102000	-1.159843000
H	-2.398107000	3.474899000	1.288626000
H	-0.208402000	4.863097000	-0.299405000
C	0.706129000	0.785711000	0.093976000
C	1.637774000	0.682727000	1.312725000
H	1.071115000	0.573098000	2.246973000
H	2.305452000	-0.179115000	1.203545000
H	2.255270000	1.585234000	1.383009000
C	1.544626000	0.926006000	-1.190993000
H	2.204241000	0.056783000	-1.303506000
H	0.909360000	1.007119000	-2.082940000
H	2.170149000	1.824874000	-1.123886000
S	-1.933680000	0.955892000	-2.810529000
C	-1.731622000	-0.648792000	-3.171492000
N	-1.546910000	-1.779369000	-3.446329000

CrBOX-Cl2-ONO2 (m4)

C	-0.056020000	-0.506529000	0.112406000
O	0.670571000	-1.605561000	0.193298000
C	-1.619844000	-2.137077000	-0.007477000
C	-0.214351000	-2.741581000	0.016669000
H	-0.042882000	-3.429026000	0.854452000
C	-0.108246000	1.988255000	0.271659000
O	0.533875000	3.111630000	0.555658000
C	-1.786181000	3.482724000	0.329805000
C	-0.437531000	4.189822000	0.491811000
H	-0.343979000	4.779861000	1.409827000
C	-2.650748000	3.996179000	-0.796184000
H	-2.132484000	3.921557000	-1.762432000
H	-3.590275000	3.426462000	-0.860141000
H	-2.917049000	5.047752000	-0.626195000
C	-2.491095000	-2.519761000	1.164857000
H	-2.025473000	-2.239234000	2.121080000
H	-3.471017000	-2.030747000	1.095158000
H	-2.647797000	-3.607259000	1.165832000
N	-1.375427000	2.084259000	0.104868000
N	-1.326944000	-0.682373000	-0.025834000
Cr	-2.716918000	0.656302000	-0.467035000
Cl	-4.361103000	-0.728573000	-1.317215000
Cl	-3.642269000	0.816693000	1.658866000
H	-2.133051000	-2.371830000	-0.950543000
H	0.073290000	-3.235298000	-0.920226000
H	-2.360040000	3.495183000	1.271547000
H	-0.174487000	4.820676000	-0.369221000
C	0.751779000	0.763398000	0.160687000
C	1.700586000	0.704688000	1.367230000
H	1.144909000	0.630662000	2.311646000
H	2.365265000	-0.162392000	1.284087000
H	2.320246000	1.607997000	1.398464000
C	1.561090000	0.862627000	-1.146950000
H	2.238567000	0.004218000	-1.233756000
H	0.905413000	0.882599000	-2.028061000
H	2.165521000	1.778082000	-1.140427000
O	-2.074255000	1.048935000	-2.311226000
N	-1.805941000	0.150182000	-3.231045000
O	-1.922805000	0.511262000	-4.389230000
O	-1.430923000	-0.960114000	-2.881485000

CrBOX-Cl2-Me (m4)

C	-0.115688000	-0.492027000	-0.040282000
O	0.596780000	-1.609893000	0.023070000
C	-1.691655000	-2.091590000	-0.143841000
C	-0.301240000	-2.711515000	-0.258383000
H	-0.103063000	-3.504682000	0.471907000
C	-0.123857000	1.997180000	0.182302000
O	0.545208000	3.107513000	0.487996000
C	-1.764233000	3.511354000	0.333333000
C	-0.404021000	4.201394000	0.416949000
H	-0.265003000	4.829460000	1.303405000
C	-2.725053000	4.077874000	-0.681671000
H	-2.282949000	4.103800000	-1.688129000
H	-3.643933000	3.472561000	-0.720281000
H	-3.016529000	5.101534000	-0.411189000
C	-2.423266000	-2.430904000	1.135215000
H	-1.808598000	-2.189798000	2.015700000
H	-3.360050000	-1.863098000	1.202211000
H	-2.656079000	-3.504388000	1.162564000
N	-1.388065000	2.119654000	0.023178000
N	-1.387135000	-0.639759000	-0.195724000
Cr	-2.834340000	0.738573000	-0.476589000
Cl	-4.579468000	-0.710961000	-1.085024000
Cl	-3.585255000	0.989716000	1.828989000
H	-2.318165000	-2.338401000	-1.012257000
H	-0.062434000	-3.080957000	-1.264984000
H	-2.257749000	3.473992000	1.320012000
H	-0.167233000	4.790785000	-0.481902000
C	0.715676000	0.757543000	0.081866000
C	1.570887000	0.645731000	1.355692000
H	0.943999000	0.556614000	2.253501000
H	2.224306000	-0.232077000	1.296769000
H	2.201112000	1.536582000	1.459693000
C	1.626994000	0.869237000	-1.153071000
H	2.292422000	-0.001108000	-1.208772000
H	1.044730000	0.924458000	-2.082390000
H	2.248338000	1.770232000	-1.077094000
C	-2.328452000	0.934942000	-2.483313000
H	-1.389652000	1.489063000	-2.648567000
H	-2.247259000	-0.060987000	-2.948390000
H	-3.154426000	1.476363000	-2.979885000

CrBOX-Cl2-NO2 (m4)

C	-0.123575000	-0.479274000	-0.040090000
O	0.600743000	-1.582622000	-0.073958000
C	-1.700350000	-2.094061000	-0.070076000
C	-0.303139000	-2.711214000	-0.174490000
H	-0.062182000	-3.402661000	0.642717000
C	-0.144814000	2.008967000	0.186244000
O	0.515200000	3.104790000	0.529332000
C	-1.792213000	3.519162000	0.379735000
C	-0.432725000	4.203476000	0.486471000
H	-0.295564000	4.802882000	1.392447000
C	-2.750634000	4.132383000	-0.607027000
H	-2.320436000	4.170779000	-1.616988000
H	-3.684411000	3.552371000	-0.648601000
H	-3.009029000	5.154805000	-0.300163000
C	-2.479942000	-2.506894000	1.155707000
H	-1.918268000	-2.280331000	2.074026000
H	-3.445698000	-1.986636000	1.199060000
H	-2.673619000	-3.587938000	1.131042000
N	-1.412483000	2.134506000	0.017739000
N	-1.405034000	-0.641022000	-0.051261000
Cr	-2.823582000	0.723240000	-0.437917000
Cl	-4.493821000	-0.697060000	-1.197769000
Cl	-3.815243000	1.132996000	1.644444000
H	-2.287675000	-2.296065000	-0.978032000
H	-0.105140000	-3.206661000	-1.133078000
H	-2.278145000	3.449786000	1.367315000
H	-0.188371000	4.816564000	-0.393584000
C	0.700072000	0.780173000	0.022830000
C	1.658773000	0.665547000	1.219844000
H	1.111500000	0.562202000	2.166311000
H	2.310990000	-0.205991000	1.095142000
H	2.290452000	1.558947000	1.277683000
C	1.507549000	0.923903000	-1.280741000
H	2.163270000	0.054701000	-1.413509000
H	0.853322000	1.007066000	-2.158016000
H	2.135944000	1.821531000	-1.225676000
N	-2.148298000	0.738991000	-2.358191000
O	-1.380327000	0.004100000	-2.956846000
O	-2.729732000	1.690743000	-2.883841000

CrBOX-Cl2-ONO (m4)

C	-0.083957000	-0.499199000	0.046926000
O	0.639609000	-1.599020000	0.145447000
C	-1.649119000	-2.127781000	-0.071959000
C	-0.245411000	-2.736351000	-0.017499000
H	-0.088660000	-3.403182000	0.839604000
C	-0.129334000	1.992897000	0.247590000
O	0.522591000	3.111939000	0.531178000
C	-1.800742000	3.489077000	0.360395000
C	-0.447534000	4.192409000	0.499213000
H	-0.332306000	4.774044000	1.420106000
C	-2.685945000	4.007593000	-0.747006000
H	-2.183295000	3.939038000	-1.722051000
H	-3.623080000	3.432125000	-0.798451000
H	-2.954365000	5.057055000	-0.567435000
C	-2.527028000	-2.498478000	1.099897000
H	-2.052219000	-2.221859000	2.052696000
H	-3.500498000	-1.996849000	1.037570000
H	-2.694662000	-3.584371000	1.103016000
N	-1.399684000	2.092251000	0.115048000
N	-1.354850000	-0.672697000	-0.105419000
Cr	-2.760847000	0.685417000	-0.478733000
Cl	-4.414092000	-0.704631000	-1.315402000
Cl	-3.650871000	0.808844000	1.689618000
H	-2.155892000	-2.375339000	-1.016520000
H	0.053445000	-3.255735000	-0.936355000
H	-2.356309000	3.496301000	1.313080000
H	-0.202265000	4.830703000	-0.361811000
C	0.726693000	0.769987000	0.092470000
C	1.711661000	0.692095000	1.267901000
H	1.185530000	0.596814000	2.227188000
H	2.378057000	-0.169339000	1.148706000
H	2.328029000	1.597457000	1.298251000
C	1.496448000	0.892339000	-1.237491000
H	2.148702000	0.020939000	-1.375036000
H	0.815155000	0.960986000	-2.096068000
H	2.124722000	1.791508000	-1.218436000
O	-2.092862000	1.051689000	-2.292784000
N	-1.768468000	0.010116000	-3.037652000
O	-1.432844000	0.307484000	-4.157001000

MnBOX-Cl3 (m5)

C	-0.120630000	-0.551788000	0.024338000
O	0.608604000	-1.660196000	0.099005000
C	-1.692047000	-2.140863000	-0.046683000
C	-0.299006000	-2.780934000	-0.035709000
H	-0.127758000	-3.454510000	0.814040000
C	-0.160624000	1.940986000	0.262555000
O	0.511270000	3.017411000	0.647718000
C	-1.782904000	3.491236000	0.459262000
C	-0.410998000	4.136701000	0.626499000
H	-0.283783000	4.696795000	1.558790000
C	-2.681160000	4.147070000	-0.558693000
H	-2.197314000	4.204069000	-1.543288000
H	-3.619565000	3.582079000	-0.665206000
H	-2.939724000	5.164702000	-0.236723000
C	-2.544667000	-2.476995000	1.155987000
H	-2.031325000	-2.203989000	2.090338000
H	-3.501972000	-1.940687000	1.117085000
H	-2.756194000	-3.554817000	1.184781000
N	-1.424500000	2.104017000	0.089063000
N	-1.390887000	-0.696008000	-0.089809000
Mn	-2.872939000	0.821743000	-0.553187000
Cl	-4.490251000	-0.605961000	-1.271000000
Cl	-3.914107000	1.176307000	1.482129000
H	-2.238483000	-2.393099000	-0.968691000
H	-0.041080000	-3.306923000	-0.963986000
H	-2.312433000	3.421117000	1.423713000
H	-0.130971000	4.778919000	-0.221413000
C	0.687995000	0.719141000	0.038373000
C	1.759271000	0.630908000	1.132184000
H	1.312426000	0.531339000	2.130493000
H	2.404676000	-0.235943000	0.953639000
H	2.388076000	1.527789000	1.121798000

C	1.353476000	0.873826000	-1.344918000
H	1.965171000	1.784931000	-1.366428000
H	2.008768000	0.015835000	-1.541528000
H	0.598467000	0.936244000	-2.140016000
Cl	-2.210203000	1.293563000	-2.733937000

MnBOX-Cl3-OH (m4)

C	-0.170617000	-0.491155000	0.019298000
O	0.616095000	-1.549853000	0.090566000
C	-1.647560000	-2.187959000	0.091514000
C	-0.222448000	-2.724808000	-0.037858000
H	0.061623000	-3.425715000	0.755872000
C	-0.340554000	1.960506000	0.326228000
O	0.236764000	2.992665000	0.921758000
C	-1.933498000	3.527435000	0.274749000
C	-0.835343000	3.924832000	1.244136000
H	-1.097530000	3.762211000	2.299247000
C	-1.868860000	4.294188000	-1.030598000
H	-0.840731000	4.305997000	-1.424033000
H	-2.522401000	3.847033000	-1.788134000
H	-2.182089000	5.335803000	-0.876093000
C	-2.322342000	-2.554811000	1.392510000
H	-1.735371000	-2.206852000	2.255439000
H	-3.326133000	-2.119502000	1.454870000
H	-2.412861000	-3.647877000	1.460180000
N	-1.583873000	2.105083000	0.025713000
N	-1.439044000	-0.720489000	-0.028378000
Mn	-2.929219000	0.636340000	-0.484730000
Cl	-4.440591000	-1.037344000	-0.871902000
Cl	-3.518329000	0.755229000	1.773985000
H	-2.263967000	-2.510362000	-0.758319000
H	-0.003572000	-3.176966000	-1.013999000
H	-2.929339000	3.581974000	0.732346000
H	-0.453963000	4.942286000	1.108002000
C	0.562442000	0.819064000	-0.035623000
C	1.782590000	0.777717000	0.884521000
H	1.498920000	0.611700000	1.932073000
H	2.462266000	-0.024227000	0.577590000
H	2.327461000	1.725953000	0.820887000
C	0.997495000	1.052377000	-1.499882000
H	1.683310000	0.255083000	-1.814585000
H	0.124847000	1.065739000	-2.166527000
H	1.527710000	2.011127000	-1.578362000
Cl	-4.508246000	2.178014000	-1.088734000
O	-2.261440000	0.597241000	-2.193308000
H	-2.836510000	1.146868000	-2.749892000

MnBOX-Cl4 (m4)

C	-0.213613000	-0.492081000	-0.009528000
O	0.566055000	-1.557732000	-0.060368000
C	-1.685977000	-2.191190000	0.013360000
C	-0.287865000	-2.702051000	-0.311589000
H	0.048936000	-3.523176000	0.331073000
C	-0.348964000	1.977344000	0.300063000
O	0.282859000	3.066796000	0.703060000
C	-1.935224000	3.557131000	0.191374000
C	-0.738946000	4.062982000	0.978162000
H	-0.895671000	4.074048000	2.065585000
C	-2.035519000	4.158983000	-1.193347000
H	-1.079300000	4.060498000	-1.730102000
H	-2.813676000	3.662775000	-1.783167000
H	-2.278097000	5.227941000	-1.121156000
C	-2.199021000	-2.633631000	1.364417000
H	-1.492285000	-2.364062000	2.163469000
H	-3.167805000	-2.174937000	1.592210000
H	-2.320763000	-3.725467000	1.369442000
N	-1.614096000	2.106212000	0.085216000
N	-1.484463000	-0.714561000	-0.029679000
Mn	-3.016750000	0.650537000	-0.246139000
Cl	-4.533579000	-1.004171000	-0.580529000
Cl	-3.282615000	0.634103000	2.042002000
H	-2.395084000	-2.464723000	-0.777508000
H	-0.152171000	-2.983999000	-1.364458000
H	-2.870808000	3.676672000	0.751375000
H	-0.361821000	5.036865000	0.648966000
C	0.542903000	0.800299000	0.052556000
C	1.603784000	0.702170000	1.156275000
H	1.143937000	0.555024000	2.142713000
H	2.279537000	-0.136234000	0.956297000
H	2.200619000	1.620142000	1.183790000
C	1.214424000	1.031169000	-1.319087000
H	1.912741000	0.212209000	-1.531542000
H	0.468904000	1.080795000	-2.124055000
H	1.780871000	1.970883000	-1.302556000
Cl	-2.459076000	0.728047000	-2.485817000
Cl	-4.681800000	2.176491000	-0.495648000

MnBOX-Cl3-F (m4)

C	-0.175507000	-0.503674000	0.030166000
O	0.613329000	-1.557541000	0.115412000
C	-1.649629000	-2.205973000	0.109276000
C	-0.219853000	-2.738088000	0.008032000
H	0.058574000	-3.417310000	0.822387000
C	-0.344306000	1.950681000	0.319914000
O	0.238296000	2.989672000	0.890125000
C	-1.944514000	3.516521000	0.271699000
C	-0.826511000	3.932040000	1.210889000
H	-1.067075000	3.794521000	2.274343000
C	-1.916777000	4.262458000	-1.046394000
H	-0.899243000	4.269513000	-1.466301000
H	-2.589744000	3.802639000	-1.779406000
H	-2.227743000	5.305607000	-0.898219000
C	-2.346804000	-2.576855000	1.396854000
H	-1.790841000	-2.207475000	2.271024000
H	-3.364814000	-2.172849000	1.432638000
H	-2.408609000	-3.671551000	1.469935000
N	-1.594286000	2.090505000	0.035980000
N	-1.443911000	-0.736694000	-0.019214000
Mn	-2.935710000	0.630529000	-0.464952000
Cl	-4.397813000	-1.043664000	-0.932185000
Cl	-3.518155000	0.684563000	1.757634000
H	-2.245518000	-2.537436000	-0.751439000
H	0.011160000	-3.214477000	-0.953405000
H	-2.928331000	3.573578000	0.754340000
H	-0.444947000	4.944663000	1.043671000
C	0.555444000	0.806981000	-0.041200000

C	1.786199000	0.775854000	0.864525000
H	1.514956000	0.617975000	1.916503000
H	2.463708000	-0.027181000	0.556045000
H	2.328400000	1.724395000	0.785737000
C	0.971814000	1.028563000	-1.513131000
H	1.652042000	0.227851000	-1.830524000
H	0.092220000	1.038065000	-2.169166000
H	1.500429000	1.986521000	-1.606946000
Cl	-4.549971000	2.155735000	-0.967931000
F	-2.292698000	0.657839000	-2.153398000

MnBOX-Cl3-N3 (m4)

C	-0.231167000	-0.491591000	-0.040405000
O	0.547527000	-1.555828000	-0.026717000
C	-1.709697000	-2.190542000	-0.012361000
C	-0.296459000	-2.714595000	-0.241513000
H	0.011455000	-3.491391000	0.467330000
C	-0.368172000	1.971399000	0.294479000
O	0.254598000	3.031563000	0.778152000
C	-1.946566000	3.559417000	0.235988000
C	-0.775619000	4.010112000	1.090899000
H	-0.964030000	3.946459000	2.171520000
C	-1.984101000	4.240443000	-1.116378000
H	-1.005857000	4.166205000	-1.616158000
H	-2.740999000	3.793183000	-1.770879000
H	-2.220357000	5.306098000	-0.992313000
C	-2.311547000	-2.597395000	1.311291000
H	-1.674106000	-2.285768000	2.151720000
H	-3.305599000	-2.156495000	1.444525000
H	-2.410115000	-3.691131000	1.343096000
N	-1.628710000	2.115101000	0.059026000
N	-1.503990000	-0.713651000	-0.093924000
Mn	-3.023692000	0.667960000	-0.344333000
Cl	-4.491707000	-0.995084000	-0.842772000
Cl	-3.429324000	0.638940000	1.926729000
H	-2.369587000	-2.491747000	-0.836496000
H	-0.113803000	-3.063483000	-1.266880000
H	-2.902179000	3.657417000	0.766035000
H	-0.387744000	5.002920000	0.840458000
C	0.519993000	0.808000000	-0.030498000
C	1.680962000	0.719664000	0.964845000
H	1.324494000	0.535259000	1.986843000
H	2.360992000	-0.090073000	0.680512000
H	2.248893000	1.656035000	0.960175000
C	1.061384000	1.055919000	-1.457210000
H	1.709183000	0.222799000	-1.758276000
H	0.246957000	1.157522000	-2.186724000
H	1.658203000	1.977122000	-1.470666000
Cl	-4.653347000	2.208838000	-0.713695000
N	-2.472116000	0.778676000	-2.269943000
N	-2.125485000	-0.235361000	-2.820330000
N	-1.770807000	-1.175548000	-3.392394000

MnBOX-Cl3-SCN (m4)

C	-0.251862000	-0.521198000	-0.074568000
O	0.518875000	-1.585420000	-0.130090000
C	-1.729647000	-2.217645000	0.039072000
C	-0.339076000	-2.744175000	-0.293661000
H	0.019421000	-3.522150000	0.389997000
C	-0.378262000	1.941259000	0.256495000
O	0.246699000	3.012399000	0.705416000
C	-1.956228000	3.530614000	0.167535000
C	-0.779837000	3.999190000	1.003351000
H	-0.959796000	3.959302000	2.086493000
C	-2.008014000	4.183109000	-1.197041000
H	-1.042416000	4.082231000	-1.715975000
H	-2.785789000	3.737249000	-1.826811000
H	-2.225854000	5.254372000	-1.089334000
C	-2.214869000	-2.612841000	1.414545000
H	-1.493845000	-2.313097000	2.189573000
H	-3.183318000	-2.156038000	1.647617000
H	-2.329016000	-3.704565000	1.458387000
N	-1.641720000	2.078191000	0.019748000
N	-1.529066000	-0.738429000	-0.058556000
Mn	-3.062837000	0.616850000	-0.196445000
Cl	-4.567856000	-1.034687000	-0.536448000
Cl	-3.314991000	0.693069000	2.080447000
H	-2.451746000	-2.519982000	-0.729643000
H	-0.230654000	-3.083260000	-1.331936000
H	-2.905719000	3.643999000	0.704607000
H	-0.392937000	4.985349000	0.727338000
C	0.510098000	0.769942000	-0.026880000
C	1.598743000	0.652225000	1.048135000
H	1.165961000	0.456756000	2.038341000
H	2.286464000	-0.162620000	0.798587000
H	2.176007000	1.581558000	1.097108000
C	1.153285000	1.027159000	-1.407577000
H	1.767944000	0.166218000	-1.698983000
H	0.396756000	1.200883000	-2.184268000
H	1.801100000	1.911010000	-1.352091000
Cl	-4.671798000	2.153534000	-0.643069000
S	-2.685544000	0.761085000	-2.686916000
C	-1.701592000	-0.533871000	-2.996792000
N	-0.986773000	-1.443042000	-3.222410000

MnBOX-Cl3-ONO2 (m4)

C	-0.188678000	-0.441793000	-0.063220000
O	0.589311000	-1.504290000	-0.088611000
C	-1.669432000	-2.141089000	-0.008440000
C	-0.261628000	-2.665681000	-0.268976000
H	0.067243000	-3.432851000	0.441319000
C	-0.339853000	2.006406000	0.321436000
O	0.259527000	3.055161000	0.851926000
C	-1.933271000	3.580314000	0.272224000
C	-0.789087000	4.013905000	1.170998000
H	-1.002749000	3.911576000	2.243882000
C	-1.938946000	4.300926000	-1.059924000
H	-0.942447000	4.259616000	-1.526138000
H	-2.663032000	3.859179000	-1.753762000
H	-2.199059000	5.357997000	-0.912525000
C	-2.252924000	-2.560386000	1.319876000
H	-1.625488000	-2.228990000	2.160336000
H	-3.263571000	-2.157884000	1.456237000
H	-2.316128000	-3.656745000	1.353657000
N	-1.596439000	2.144460000	0.059663000
N	-1.461675000	-0.665530000	-0.080436000

Mn	-2.959645000	0.686292000	-0.404842000
Cl	-4.414665000	-0.940302000	-0.962014000
Cl	-3.483423000	0.677004000	1.816494000
H	-2.340956000	-2.425963000	-0.826018000
H	-0.104233000	-3.025816000	-1.294155000
H	-2.901378000	3.653961000	0.783387000
H	-0.405072000	5.017929000	0.962893000
C	0.560108000	0.859141000	-0.023005000
C	1.705138000	0.745672000	0.989250000
H	1.332769000	0.520506000	1.997336000
H	2.398153000	-0.046494000	0.687174000
H	2.262967000	1.687385000	1.028343000
C	1.127576000	1.155053000	-1.429513000
H	1.750429000	0.316267000	-1.765135000
H	0.332668000	1.329164000	-2.166370000
H	1.755728000	2.054104000	-1.388675000
Cl	-4.565120000	2.231893000	-0.850232000
O	-1.796084000	-1.123661000	-2.816150000
N	-1.918345000	0.039208000	-3.148803000
O	-2.327474000	0.941172000	-2.274718000
O	-1.674791000	0.488388000	-4.254567000

MnBOX-Cl3-Me (m4)

C	-0.204555000	-0.457276000	-0.231879000
O	0.538389000	-1.514374000	-0.528167000
C	-1.685315000	-2.131649000	-0.248021000
C	-0.381910000	-2.579869000	-0.883116000
H	0.015119000	-3.520396000	-0.486128000
C	-0.251186000	2.016265000	0.109614000
O	0.459200000	3.122814000	0.270782000
C	-1.803689000	3.629473000	-0.033256000
C	-0.481169000	4.208343000	0.453963000
H	-0.479218000	4.464948000	1.521979000
C	-2.190139000	4.084100000	-1.423056000
H	-1.383374000	3.882826000	-2.143772000
H	-3.104870000	3.587174000	-1.767958000
H	-2.376532000	5.166864000	-1.418652000
C	-1.896476000	-2.684388000	1.145061000
H	-0.994237000	-2.540665000	1.759796000
H	-2.728101000	-2.174892000	1.645038000
H	-2.110132000	-3.761010000	1.097658000
N	-1.526181000	2.166851000	-0.007615000
N	-1.476394000	-0.659109000	-0.166486000
Mn	-2.998332000	0.704582000	0.078083000
Cl	-4.598852000	-0.924463000	0.032058000
Cl	-2.586691000	0.629122000	2.460132000
H	-2.546972000	-2.341714000	-0.893312000
H	-0.417271000	-2.628103000	-1.980654000
H	-2.610610000	3.839945000	0.679374000
H	-0.125798000	5.067613000	-0.125988000
C	0.583776000	0.770608000	0.107092000
C	1.121312000	0.556879000	1.539598000
H	0.294957000	0.449890000	2.255795000
H	1.743238000	-0.346544000	1.574551000
H	1.741106000	1.412802000	1.834119000
C	1.744620000	0.947936000	-0.879993000
H	2.392736000	0.064766000	-0.862024000
H	1.381237000	1.090813000	-1.906890000
H	2.349657000	1.817607000	-0.601092000
Cl	-4.647238000	2.279013000	0.189134000
C	-3.044622000	0.696773000	-2.016403000
H	-2.171895000	1.263450000	-2.363930000
H	-2.996729000	-0.354074000	-2.324995000
H	-3.994572000	1.161454000	-2.305039000

MnBOX-Cl3-NO2 (m4)

C	-0.210951000	-0.483203000	-0.038159000
O	0.565313000	-1.548579000	-0.112416000
C	-1.687197000	-2.176427000	-0.003300000
C	-0.296565000	-2.690743000	-0.351978000
H	0.050369000	-3.514727000	0.281624000
C	-0.356750000	1.979560000	0.281454000
O	0.247660000	3.042347000	0.777808000
C	-1.940804000	3.562146000	0.171376000
C	-0.788408000	4.025708000	1.045396000
H	-1.001227000	3.982749000	2.122552000
C	-1.989633000	4.259679000	-1.170052000
H	-1.019233000	4.184716000	-1.685007000
H	-2.756817000	3.821027000	-1.816204000
H	-2.215426000	5.325224000	-1.025955000
C	-2.188599000	-2.609537000	1.354659000
H	-1.467277000	-2.353451000	2.145113000
H	-3.143298000	-2.125484000	1.592356000
H	-2.336733000	-3.698025000	1.364590000
N	-1.606725000	2.118372000	-0.015262000
N	-1.483345000	-0.700465000	-0.043050000
Mn	-3.028606000	0.648282000	-0.200757000
Cl	-4.531244000	-0.992697000	-0.633940000
Cl	-3.286251000	0.729925000	2.115396000
H	-2.406807000	-2.445024000	-0.785491000
H	-0.179406000	-2.969102000	-1.408376000
H	-2.900869000	3.642900000	0.695754000
H	-0.393842000	5.013840000	0.786549000
C	0.544430000	0.809374000	0.032927000
C	1.596444000	0.701587000	1.143722000
H	1.127466000	0.542572000	2.124077000
H	2.274352000	-0.134261000	0.940319000
H	2.191264000	1.620151000	1.187811000
C	1.224052000	1.057751000	-1.330690000
H	1.914577000	0.236252000	-1.557962000
H	0.485973000	1.135648000	-2.140729000
H	1.799960000	1.991179000	-1.294043000
Cl	-4.632516000	2.170561000	-0.699388000
N	-2.469323000	0.541565000	-2.352920000
O	-2.141032000	-0.549274000	-2.765837000
O	-2.491966000	1.569960000	-2.987975000

MnBOX-Cl3-ONO (m4)

C	-0.122274000	-0.443230000	-0.137524000
O	0.643192000	-1.484816000	-0.417390000
C	-1.597554000	-2.115630000	-0.337825000
C	-0.241373000	-2.543399000	-0.871524000
H	0.126393000	-3.492103000	-0.465980000
C	-0.190182000	2.030204000	0.153528000
O	0.518300000	3.140471000	0.257345000
C	-1.728987000	3.630569000	-0.192894000

C	-0.413718000	4.248883000	0.271197000
H	-0.444547000	4.636592000	1.298091000
C	-2.090047000	3.970366000	-1.621767000
H	-1.310335000	3.634831000	-2.321558000
H	-3.047502000	3.522840000	-1.913083000
H	-2.190576000	5.059606000	-1.723454000
C	-1.944423000	-2.739732000	0.996386000
H	-1.113878000	-2.622449000	1.709878000
H	-2.834915000	-2.273637000	1.433999000
H	-2.134973000	-3.814362000	0.870202000
N	-1.459631000	2.173842000	-0.031863000
N	-1.396032000	-0.648458000	-0.170956000
Mn	-2.930375000	0.710527000	0.032046000
Cl	-4.496742000	-0.908185000	-0.337190000
Cl	-2.761668000	0.552051000	2.386362000
H	-2.392527000	-2.286420000	-1.073733000
H	-0.185264000	-2.564342000	-1.968503000
H	-2.549349000	3.905519000	0.481676000
H	-0.022419000	5.024669000	-0.396950000
C	0.627753000	0.780170000	0.296147000
C	0.947759000	0.591399000	1.795873000
H	0.026328000	0.490007000	2.385540000
H	1.561627000	-0.308341000	1.931742000
H	1.516601000	1.455136000	2.163009000
C	1.923325000	0.926127000	-0.510280000
H	2.550725000	0.038880000	-0.374203000
H	1.720501000	1.048163000	-1.582669000
H	2.489458000	1.796471000	-0.162164000
Cl	-4.574015000	2.276588000	0.033287000
N	-3.475132000	0.899132000	-3.028256000
O	-2.641382000	0.778672000	-2.119373000
O	-4.652534000	1.035743000	-2.898361000

MnBOX-Cl2 (m6)

C	-0.037581000	-0.512354000	0.150134000
O	0.722587000	-1.590901000	0.349015000
C	-1.532537000	-2.163037000	-0.000860000
C	-0.163831000	-2.734543000	0.383668000
H	-0.138962000	-3.145524000	1.403791000
C	-0.127066000	1.988219000	0.345970000
O	0.542902000	3.078527000	0.723908000
C	-1.769272000	3.489642000	0.535565000
C	-0.414487000	4.164469000	0.777115000
H	-0.318562000	4.652826000	1.753857000
C	-2.601120000	4.101497000	-0.568315000
H	-2.072630000	4.051818000	-1.531571000
H	-3.557253000	3.566457000	-0.673853000
H	-2.829643000	5.154021000	-0.351543000
C	-2.660783000	-2.527115000	0.933527000
H	-2.433052000	-2.216915000	1.964087000
H	-3.592727000	-2.029401000	0.631160000
H	-2.836619000	-3.611457000	0.931853000
N	-1.393624000	2.107011000	0.203504000
N	-1.291954000	-0.711868000	-0.025404000
Mn	-2.865106000	0.710052000	-0.605379000
Cl	-4.566688000	0.944845000	1.038327000
H	-1.803214000	-2.457484000	-1.030990000
H	0.227421000	-3.487384000	-0.310453000
H	-2.367464000	3.453608000	1.462301000
H	-0.143848000	4.886151000	-0.007759000
C	0.729540000	0.784749000	0.037273000
C	1.965409000	0.763562000	0.936551000
H	1.697170000	0.661992000	1.996719000
H	2.619675000	-0.072392000	0.666191000
H	2.534723000	1.691671000	0.814748000
C	1.145803000	0.914756000	-1.445144000
H	1.790264000	0.072854000	-1.731182000
H	0.260819000	0.923632000	-2.098540000
H	1.710310000	1.844714000	-1.595709000
Cl	-2.527263000	0.772246000	-2.967522000

MnBOX-Cl2-OH (m5)

C	-0.032737000	-0.478172000	0.138372000
O	0.727182000	-1.572356000	0.230965000
C	-1.528976000	-2.117488000	-0.103189000
C	-0.174173000	-2.703272000	0.290045000
H	-0.161299000	-3.087520000	1.321741000
C	-0.115071000	2.023341000	0.321550000
O	0.555820000	3.126942000	0.659948000
C	-1.757909000	3.529484000	0.493195000
C	-0.399904000	4.215463000	0.666518000
H	-0.284656000	4.762140000	1.609303000
C	-2.638591000	4.103956000	-0.590962000
H	-2.132304000	4.074405000	-1.567105000
H	-3.572396000	3.526546000	-0.673766000
H	-2.905879000	5.146398000	-0.371443000
C	-2.697322000	-2.594326000	0.721313000
H	-2.550944000	-2.368301000	1.786470000
H	-3.629581000	-2.111573000	0.395825000
H	-2.824921000	-3.679904000	0.611524000
N	-1.380732000	2.142410000	0.185018000
N	-1.293429000	-0.668445000	0.014020000
Mn	-2.881495000	0.718551000	-0.427582000
Cl	-5.054137000	0.825618000	-1.261272000
Cl	-3.593103000	0.729655000	1.788487000
H	-1.737624000	-2.308584000	-1.172300000
H	0.204761000	-3.478628000	-0.384637000
H	-2.316791000	3.508298000	1.445110000
H	-0.146338000	4.887828000	-0.166782000
C	0.744819000	0.812138000	0.072302000
C	1.913169000	0.780300000	1.060612000
H	1.563792000	0.693013000	2.098362000
H	2.568238000	-0.070510000	0.843877000
H	2.507511000	1.696530000	0.973520000
C	1.268277000	0.932037000	-1.375742000
H	1.926311000	0.085574000	-1.611256000
H	0.433034000	0.944685000	-2.089296000
H	1.847555000	1.857968000	-1.488079000
O	-2.252757000	0.746474000	-2.158682000
H	-3.011997000	0.875116000	-2.746761000

MnBOX-Cl2-F (m5)

C	-0.040331000	-0.491913000	0.140193000
O	0.717118000	-1.582122000	0.267351000
C	-1.535469000	-2.136889000	-0.089410000
C	-0.183323000	-2.714671000	0.326202000

H	-0.180658000	-3.089180000	1.361308000
C	-0.127446000	2.009312000	0.304124000
O	0.534717000	3.121290000	0.619456000
C	-1.784962000	3.504544000	0.490527000
C	-0.429817000	4.204883000	0.613384000
H	-0.297445000	4.780071000	1.536229000
C	-2.708531000	4.050519000	-0.572296000
H	-2.236289000	4.006547000	-1.564826000
H	-3.639808000	3.463910000	-0.611707000
H	-2.979110000	5.094063000	-0.363194000
C	-2.708522000	-2.599403000	0.736863000
H	-2.566019000	-2.360146000	1.799656000
H	-3.639427000	-2.122214000	0.400236000
H	-2.834809000	-3.686511000	0.641750000
N	-1.397058000	2.119076000	0.185048000
N	-1.299474000	-0.684121000	-0.002449000
Mn	-2.877426000	0.719751000	-0.443426000
Cl	-5.047232000	0.572062000	-1.247722000
Cl	-3.590896000	0.700880000	1.739819000
H	-1.736987000	-2.352006000	-1.154980000
H	0.205605000	-3.495021000	-0.336871000
H	-2.311438000	3.486985000	1.460973000
H	-0.198234000	4.850755000	-0.246700000
C	0.736054000	0.799208000	0.063901000
C	1.902951000	0.779995000	1.054132000
H	1.553126000	0.699348000	2.092115000
H	2.561612000	-0.069564000	0.843753000
H	2.493529000	1.697856000	0.959235000
C	1.263085000	0.908638000	-1.383617000
H	1.919014000	0.058701000	-1.611752000
H	0.430500000	0.918168000	-2.099043000
H	1.844349000	1.832410000	-1.501490000
F	-2.217380000	0.832533000	-2.124759000

MnBOX-Cl2-N3 (m5)

C	-0.081821000	-0.528628000	-0.143862000
O	0.655454000	-1.614592000	-0.364317000
C	-1.639108000	-2.123939000	-0.361052000
C	-0.245072000	-2.750701000	-0.344508000
H	-0.042194000	-3.319984000	0.574886000
C	-0.153135000	1.973776000	-0.000859000
O	0.537312000	3.111316000	-0.040695000
C	-1.776522000	3.512210000	0.113473000
C	-0.421549000	4.194659000	-0.094580000
H	-0.155838000	4.922244000	0.680522000
C	-2.836209000	3.883095000	-0.895655000
H	-2.503860000	3.655804000	-1.919461000
H	-3.770762000	3.336592000	-0.703586000
H	-3.058755000	4.957327000	-0.840259000
C	-2.616724000	-2.691332000	0.638087000
H	-2.228608000	-2.604553000	1.662586000
H	-3.574172000	-2.149651000	0.587413000
H	-2.819591000	-3.750116000	0.428878000
N	-1.430211000	2.080222000	0.055285000
N	-1.350482000	-0.704920000	-0.086937000
Mn	-2.988894000	0.602604000	0.293036000
Cl	-5.273699000	0.618392000	0.564435000
Cl	-2.561340000	0.535144000	2.555495000
H	-2.079281000	-2.172901000	-1.373646000
H	-0.017654000	-3.379604000	-1.211740000
H	-2.158921000	3.702918000	1.131937000
H	-0.327556000	4.673163000	-1.080660000
C	0.707081000	0.737136000	0.052648000
C	1.330926000	0.673483000	1.462550000
H	0.552972000	0.618568000	2.236035000
H	1.973957000	-0.211936000	1.545910000
H	1.946191000	1.563967000	1.642157000
C	1.808694000	0.825906000	-1.010963000
H	2.469644000	-0.045711000	-0.946155000
H	1.387420000	0.868108000	-2.024529000
H	2.414818000	1.724462000	-0.850434000
N	-3.254804000	0.618532000	-1.696038000
N	-2.287918000	0.745968000	-2.401380000
N	-1.391724000	0.871584000	-3.122564000

MnBOX-Cl2-SCN (m5)

C	-0.073054000	-0.560213000	-0.114983000
O	0.658889000	-1.631376000	-0.397531000
C	-1.623444000	-2.169341000	-0.284343000
C	-0.224900000	-2.779522000	-0.337882000
H	0.030966000	-3.347569000	0.569054000
C	-0.145375000	1.942139000	-0.065724000
O	0.545429000	3.069618000	-0.205454000
C	-1.745160000	3.503811000	0.051108000
C	-0.417423000	4.140963000	-0.350534000
H	-0.097901000	4.975800000	0.282271000
C	-2.913112000	3.874947000	-0.827407000
H	-2.716886000	3.625630000	-1.880145000
H	-3.830013000	3.357618000	-0.510004000
H	-3.105052000	4.954713000	-0.764041000
C	-2.562169000	-2.784359000	0.723068000
H	-2.150648000	-2.726317000	1.740968000
H	-3.534646000	-2.269125000	0.712817000
H	-2.745654000	-3.840716000	0.485245000
N	-1.422010000	2.060920000	0.023546000
N	-1.336378000	-0.752854000	0.016500000
Mn	-2.939063000	0.587972000	0.389489000
Cl	-5.231560000	0.604605000	0.517310000
Cl	-2.570408000	0.621540000	2.639071000
H	-2.095425000	-2.192631000	-1.283504000
H	-0.031729000	-3.402163000	-1.217713000
H	-1.989571000	3.743308000	1.102198000
H	-0.401271000	4.459598000	-1.403922000
C	0.709358000	0.709410000	0.085267000
C	1.216655000	0.695063000	1.545356000
H	0.380955000	0.664132000	2.257604000
H	1.853008000	-0.184598000	1.707666000
H	1.816238000	1.593346000	1.740552000
C	1.898364000	0.762492000	-0.878323000
H	2.546690000	-0.107361000	-0.725710000
H	1.568132000	0.774832000	-1.925149000
H	2.492336000	1.662866000	-0.687108000
S	-3.412402000	0.427486000	-2.016162000
C	-1.871826000	0.521963000	-2.634406000
N	-0.779347000	0.589148000	-3.067055000

MnBOX-Cl2-ONO2 (m5)

C	-0.067446000	-0.494790000	-0.117915000
O	0.665421000	-1.575238000	-0.361950000
C	-1.635809000	-2.082228000	-0.298952000
C	-0.241029000	-2.703862000	-0.428477000
H	0.010839000	-3.382948000	0.398649000
C	-0.129867000	2.009769000	0.005658000
O	0.553742000	3.147714000	-0.040946000
C	-1.744142000	3.559271000	0.161360000
C	-0.415493000	4.217231000	-0.191937000
H	-0.121001000	5.039809000	0.467788000
C	-2.919778000	3.946072000	-0.699985000
H	-2.728068000	3.719560000	-1.757487000
H	-3.822270000	3.398832000	-0.385996000
H	-3.134402000	5.018202000	-0.600181000
C	-2.502774000	-2.672050000	0.788038000
H	-2.019939000	-2.582882000	1.771839000
H	-3.471271000	-2.154393000	0.833489000
H	-2.697780000	-3.735101000	0.592795000
N	-1.408603000	2.123677000	0.054432000
N	-1.334766000	-0.664882000	-0.039528000
Mn	-2.975586000	0.726389000	-0.037443000
Cl	-5.231856000	0.432132000	-0.344872000
Cl	-3.142976000	0.780882000	2.211542000
H	-2.172167000	-2.122591000	-1.262906000
H	-0.064250000	-3.220246000	-1.379172000
H	-1.994902000	3.733115000	1.223249000
H	-0.372083000	4.555683000	-1.238526000
C	0.725378000	0.771432000	0.067382000
C	1.340389000	0.722059000	1.482017000
H	0.555698000	0.685921000	2.250132000
H	1.972220000	-0.169566000	1.581753000
H	1.963777000	1.608168000	1.654628000
C	1.825791000	0.851988000	-0.997563000
H	2.490901000	-0.015349000	-0.921888000
H	1.400431000	0.875789000	-2.010416000
H	2.426765000	1.756426000	-0.851828000
O	-1.075865000	-0.177360000	-2.876901000
N	-1.664713000	0.864317000	-2.635227000
O	-2.827845000	0.783303000	-2.004782000
O	-1.278339000	1.972194000	-2.970979000

MnBOX-Cl2-Me (m5)

C	0.008882000	-0.469551000	-0.096535000
O	0.770285000	-1.564588000	-0.189134000
C	-1.509588000	-2.075245000	-0.419886000
C	-0.128683000	-2.698665000	-0.213936000
H	-0.034411000	-3.222159000	0.749869000
C	-0.067521000	2.039835000	0.068416000
O	0.631304000	3.175928000	0.167350000
C	-1.677602000	3.579426000	0.190143000
C	-0.317317000	4.262406000	0.058513000
H	-0.094136000	4.992066000	0.844685000
C	-2.735148000	4.043071000	-0.780107000
H	-2.400343000	3.922842000	-1.820917000
H	-3.663901000	3.468718000	-0.644133000
H	-2.972237000	5.103286000	-0.617722000
C	-2.600778000	-2.604199000	0.478798000
H	-2.336770000	-2.471759000	1.537157000
H	-3.545453000	-2.068335000	0.294882000
H	-2.778480000	-3.672014000	0.292401000
N	-1.341159000	2.155852000	0.015214000
N	-1.255336000	-0.645490000	-0.178129000
Mn	-2.928615000	0.693609000	0.052674000
Cl	-5.253760000	0.729379000	-0.008048000
Cl	-2.684354000	0.631545000	2.427433000
H	-1.826530000	-2.171000000	-1.474205000
H	0.191265000	-3.372116000	-1.016663000
H	-2.063512000	3.675577000	1.221564000
H	-0.171585000	4.739013000	-0.923314000
C	0.786963000	0.800157000	0.119348000
C	1.399628000	0.730194000	1.532652000
H	0.611426000	0.673628000	2.296059000
H	2.039184000	-0.156881000	1.620872000
H	2.013387000	1.618745000	1.724839000
C	1.894396000	0.895641000	-0.938935000
H	2.563644000	0.030360000	-0.867208000
H	1.477651000	0.926315000	-1.955433000
H	2.489725000	1.802433000	-0.781640000
C	-2.986349000	0.669928000	-2.013678000
H	-1.962222000	0.759781000	-2.404420000
H	-3.438774000	-0.291462000	-2.300141000
H	-3.620586000	1.499362000	-2.354590000

MnBOX-Cl2-NO2 (m5)

C	-0.061046000	-0.496789000	0.065591000
O	0.689556000	-1.594424000	0.130501000
C	-1.587673000	-2.123808000	-0.102679000
C	-0.213871000	-2.724320000	0.197093000
H	-0.143187000	-3.146950000	1.210682000
C	-0.135197000	2.004818000	0.233423000
O	0.536613000	3.103065000	0.570051000
C	-1.773907000	3.515641000	0.448997000
C	-0.412442000	4.198088000	0.577809000
H	-0.270317000	4.762714000	1.505659000
C	-2.706299000	4.131623000	-0.564067000
H	-2.233263000	4.199829000	-1.552926000
H	-3.623283000	3.531192000	-0.664334000
H	-3.002595000	5.141474000	-0.249459000
C	-2.692014000	-2.568618000	0.823624000
H	-2.459782000	-2.319735000	1.868411000
H	-3.644452000	-2.087018000	0.560388000
H	-2.835308000	-3.655388000	0.751044000
N	-1.404336000	2.131581000	0.096426000
N	-1.328180000	-0.673268000	-0.023074000
Mn	-2.954606000	0.714029000	-0.281483000
Cl	-5.065168000	0.546311000	-1.226690000
Cl	-3.608382000	0.742313000	1.940743000
H	-1.883182000	-2.326905000	-1.146822000
H	0.125092000	-3.473548000	-0.526749000
H	-2.284394000	3.459387000	1.426472000
H	-0.175568000	4.848993000	-0.277110000
C	0.726350000	0.787581000	0.017681000
C	1.841132000	0.750035000	1.069790000
H	1.435201000	0.686026000	2.088282000
H	2.487643000	-0.117795000	0.899812000

H	2.459261000	1.651665000	0.999745000
C	1.344571000	0.896366000	-1.392264000
H	2.017393000	0.048583000	-1.573425000
H	0.575618000	0.903363000	-2.176232000
H	1.930125000	1.821262000	-1.472507000
N	-2.345018000	0.710078000	-2.301201000
O	-2.164095000	-0.377773000	-2.819691000
O	-2.216190000	1.780452000	-2.865778000

MnBOX-Cl2-ONO (m5)

C	-0.048061000	-0.452904000	-0.126424000
O	0.700402000	-1.528814000	-0.361006000
C	-1.587226000	-2.059505000	-0.383850000
C	-0.188571000	-2.673565000	-0.349835000
H	0.007509000	-3.237976000	0.574286000
C	-0.122135000	2.054522000	0.057761000
O	0.571264000	3.190906000	0.080429000
C	-1.744825000	3.594272000	0.191838000
C	-0.383733000	4.279255000	0.042692000
H	-0.135528000	4.975923000	0.851192000
C	-2.785173000	4.004279000	-0.822636000
H	-2.426159000	3.845876000	-1.850149000
H	-3.713302000	3.430294000	-0.685583000
H	-3.033227000	5.068163000	-0.709286000
C	-2.585944000	-2.672463000	0.567063000
H	-2.233546000	-2.616540000	1.606882000
H	-3.554770000	-2.155029000	0.502424000
H	-2.757130000	-3.728171000	0.317125000
N	-1.398493000	2.166114000	0.083522000
N	-1.313592000	-0.645462000	-0.071372000
Mn	-2.940329000	0.695986000	0.141186000
Cl	-5.237906000	0.714638000	0.155358000
Cl	-2.798515000	0.648505000	2.422217000
H	-1.997105000	-2.083319000	-1.410498000
H	0.055348000	-3.302986000	-1.212048000
H	-2.149769000	3.741836000	1.208623000
H	-0.263643000	4.794166000	-0.921756000
C	0.733259000	0.814821000	0.089407000
C	1.348616000	0.729564000	1.502090000
H	0.561933000	0.671467000	2.267005000
H	1.984062000	-0.161565000	1.581316000
H	1.968061000	1.613324000	1.698869000
C	1.833919000	0.923725000	-0.973050000
H	2.502262000	0.057048000	-0.919257000
H	1.409549000	0.973169000	-1.985555000
H	2.432487000	1.825745000	-0.803572000
N	-1.913996000	0.785072000	-2.493616000
O	-3.051990000	0.696014000	-1.804784000
O	-2.104088000	0.807212000	-3.679506000

FeBOX-Cl3 (m6)

C	-0.128559000	-0.541540000	0.025644000
O	0.577329000	-1.639778000	0.236863000
C	-1.700698000	-2.144528000	-0.006748000
C	-0.310792000	-2.772557000	0.066446000
H	-0.164909000	-3.444668000	0.920588000
C	-0.150493000	1.936877000	0.290957000
O	0.518293000	2.998258000	0.731590000
C	-1.782869000	3.448125000	0.521689000
C	-0.422537000	4.098867000	0.780876000
H	-0.327847000	4.580442000	1.760609000
C	-2.627343000	4.148214000	-0.514782000
H	-2.096781000	4.230929000	-1.473794000
H	-3.569425000	3.605959000	-0.682835000
H	-2.883605000	5.160740000	-0.173374000
C	-2.553531000	-2.352362000	1.223230000
H	-2.026417000	-2.018884000	2.129781000
H	-3.492184000	-1.788661000	1.139726000
H	-2.800085000	-3.416289000	1.341928000
N	-1.411577000	2.079163000	0.113698000
N	-1.393906000	-0.703558000	-0.180103000
Fe	-2.856496000	0.701352000	-0.721467000
Cl	-4.321329000	-0.913058000	-1.496735000
Cl	-4.176501000	1.211183000	1.139500000
H	-2.242477000	-2.484439000	-0.900066000
H	-0.007611000	-3.289598000	-0.853503000
H	-2.362208000	3.350677000	1.455423000
H	-0.132856000	4.817687000	0.000226000
C	0.690776000	0.723664000	0.003472000
C	1.834581000	0.622166000	1.016947000
H	1.462149000	0.487240000	2.041129000
H	2.483715000	-0.224432000	0.769479000
H	2.441558000	1.533480000	0.987731000
C	1.264237000	0.882666000	-1.420738000
H	1.880677000	1.789888000	-1.469762000
H	1.900555000	0.022646000	-1.666602000
H	0.461868000	0.960016000	-2.166387000
Cl	-2.150028000	1.338132000	-2.840061000

FeBOX-Cl3-OH (m5)

C	-0.154095000	-0.509509000	0.017595000
O	0.646703000	-1.561360000	0.130983000
C	-1.612898000	-2.200584000	0.144816000
C	-0.186728000	-2.742092000	0.040921000
H	0.095744000	-3.416119000	0.858633000
C	-0.343040000	1.942285000	0.322086000
O	0.220091000	2.927899000	1.012571000
C	-1.921020000	3.513917000	0.312218000
C	-0.856405000	3.848961000	1.343997000
H	-1.159576000	3.635503000	2.379312000
C	-1.819532000	4.371811000	-0.933182000
H	-0.781426000	4.404218000	-1.298212000
H	-2.454491000	3.979418000	-1.736264000
H	-2.133663000	5.402189000	-0.716681000
C	-2.287645000	-2.496187000	1.465562000
H	-1.689091000	-2.114291000	2.306462000
H	-3.281192000	-2.034082000	1.512665000
H	-2.403289000	-3.581298000	1.594365000
N	-1.567917000	2.115134000	-0.028227000
N	-1.417797000	-0.743167000	-0.038440000
Fe	-3.025623000	0.618717000	-0.607621000
Cl	-4.481719000	-1.162429000	-0.965494000
Cl	-3.709248000	0.797694000	1.553140000
H	-2.232633000	-2.568521000	-0.684777000
H	0.035132000	-3.229790000	-0.917823000
H	-2.933538000	3.541506000	0.738348000

H	-0.463931000	4.869547000	1.278772000
C	0.564269000	0.810247000	-0.075688000
C	1.824788000	0.788774000	0.787707000
H	1.593899000	0.622680000	1.848054000
H	2.500527000	-0.005600000	0.453147000
H	2.355548000	1.742999000	0.697330000
C	0.937062000	1.035661000	-1.556987000
H	1.612977000	0.240086000	-1.897454000
H	0.040645000	1.041257000	-2.190976000
H	1.457959000	1.996847000	-1.665677000
Cl	-4.514119000	2.279398000	-1.348816000
O	-2.336734000	0.540222000	-2.284187000
H	-2.862899000	1.132605000	-2.851652000

FeBOX-Cl4 (m5)

C	-0.132806000	-0.400451000	-0.160726000
O	0.629964000	-1.386266000	-0.616930000
C	-1.604621000	-2.055228000	-0.437304000
C	-0.264815000	-2.434248000	-1.061667000
H	0.137127000	-3.394019000	-0.717068000
C	-0.196322000	2.067143000	0.181065000
O	0.515142000	3.180335000	0.143281000
C	-1.773097000	3.643578000	-0.055512000
C	-0.413283000	4.291594000	0.189513000
H	-0.313803000	4.759902000	1.177806000
C	-2.328794000	3.889815000	-1.440203000
H	-1.627797000	3.541763000	-2.213930000
H	-3.286259000	3.372389000	-1.575850000
H	-2.497239000	4.965181000	-1.590453000
C	-1.942902000	-2.864453000	0.796387000
H	-1.137670000	-2.797405000	1.544196000
H	-2.873248000	-2.518088000	1.262729000
H	-2.069439000	-3.922337000	0.527457000
N	-1.481059000	2.200721000	0.148342000
N	-1.396480000	-0.629205000	-0.085252000
Fe	-2.975864000	0.746076000	0.474310000
Cl	-4.627383000	2.324855000	0.674828000
Cl	-2.769398000	0.193381000	2.674042000
H	-2.416361000	-2.120553000	-1.174982000
H	-0.268030000	-2.418043000	-2.160251000
H	-2.502727000	3.954668000	0.703767000
H	-0.113884000	5.010805000	-0.581625000
C	0.625992000	0.813102000	0.307689000
C	0.936578000	0.606900000	1.806705000
H	0.016866000	0.498910000	2.397227000
H	1.546213000	-0.297342000	1.934879000
H	1.507503000	1.462936000	2.189584000
C	1.930868000	0.969083000	-0.478124000
H	2.498640000	1.826724000	-0.102360000
H	2.551342000	0.075280000	-0.354863000
H	1.746073000	1.117938000	-1.550207000
Cl	-4.670514000	-0.960873000	-0.225055000
Cl	-3.459016000	0.542005000	-2.117219000

FeBOX-Cl3-F (m5)

C	-0.159774000	-0.528966000	0.049770000
O	0.636636000	-1.577631000	0.208257000
C	-1.625279000	-2.215023000	0.162875000
C	-0.195865000	-2.759424000	0.131837000
H	0.054548000	-3.402673000	0.984221000
C	-0.347224000	1.927230000	0.312774000
O	0.206832000	2.925241000	0.984198000
C	-1.933196000	3.499975000	0.263768000
C	-0.866133000	3.862676000	1.283496000
H	-1.166556000	3.686618000	2.326052000
C	-1.852111000	4.331113000	-1.000118000
H	-0.816233000	4.373400000	-1.369770000
H	-2.481828000	3.906485000	-1.790783000
H	-2.185750000	5.359597000	-0.805888000
C	-2.362688000	-2.499374000	1.452001000
H	-1.825818000	-2.079623000	2.315963000
H	-3.373714000	-2.074885000	1.432492000
H	-2.448978000	-3.584963000	1.598935000
N	-1.571238000	2.097352000	-0.053091000
N	-1.421502000	-0.760337000	-0.038656000
Fe	-3.007426000	0.640013000	-0.663755000
Cl	-4.386054000	-1.155710000	-1.107067000
Cl	-3.720938000	0.752169000	1.473355000
H	-2.205014000	-2.598230000	-0.688349000
H	0.060286000	-3.283997000	-0.798296000
H	-2.941825000	3.525656000	0.699810000
H	-0.468278000	4.878125000	1.181408000
C	0.563584000	0.787770000	-0.050843000
C	1.807512000	0.780530000	0.836148000
H	1.556443000	0.633322000	1.894614000
H	2.487017000	-0.021046000	0.528005000
H	2.341956000	1.731872000	0.738274000
C	0.963356000	0.990782000	-1.528993000
H	1.642366000	0.187446000	-1.843380000
H	0.079650000	0.989546000	-2.179495000
H	1.488708000	1.948704000	-1.643040000
Cl	-4.574865000	2.264347000	-1.257294000
F	-2.287343000	0.618412000	-2.296119000

FeBOX-Cl3-N3 (m5)

C	-0.219109000	-0.498012000	-0.042027000
O	0.580698000	-1.556290000	-0.039648000
C	-1.673743000	-2.198744000	-0.010672000
C	-0.253692000	-2.725509000	-0.208001000
H	0.053181000	-3.469250000	0.537181000
C	-0.362344000	1.975691000	0.290977000
O	0.271612000	3.020621000	0.808646000
C	-1.919284000	3.571918000	0.251358000
C	-0.745979000	4.009497000	1.113370000
H	-0.942317000	3.953984000	2.193414000
C	-1.968993000	4.289899000	-1.082266000
H	-0.997008000	4.220711000	-1.595148000
H	-2.739146000	3.867103000	-1.738609000
H	-2.195179000	5.354437000	-0.930812000
C	-2.313456000	-2.611901000	1.294706000
H	-1.705693000	-2.289974000	2.153476000
H	-3.315743000	-2.178637000	1.394538000
H	-2.407159000	-3.705936000	1.332273000
N	-1.613869000	2.135159000	0.031664000
N	-1.486592000	-0.726025000	-0.077653000
Fe	-3.165286000	0.687540000	-0.306970000

Cl	-4.531729000	-1.083483000	-0.920964000
Cl	-3.569113000	0.677932000	1.918714000
H	-2.318428000	-2.505296000	-0.848359000
H	-0.063587000	-3.123517000	-1.214208000
H	-2.874630000	3.657685000	0.786327000
H	-0.345027000	4.998386000	0.865491000
C	0.524277000	0.809585000	-0.042894000
C	1.699446000	0.729351000	0.936844000
H	1.357644000	0.552206000	1.965343000
H	2.375698000	-0.082988000	0.651011000
H	2.267851000	1.665318000	0.919757000
C	1.049865000	1.055232000	-1.474808000
H	1.711390000	0.232689000	-1.775703000
H	0.228677000	1.134082000	-2.199521000
H	1.628602000	1.987954000	-1.504149000
Cl	-4.731517000	2.316560000	-0.795619000
N	-2.550997000	0.707749000	-2.306331000
N	-2.164616000	-0.309444000	-2.812724000
N	-1.766949000	-1.258912000	-3.345247000

FeBOX-Cl3-SCN (m5)

C	-0.235267000	-0.500396000	-0.056737000
O	0.556610000	-1.548893000	-0.221091000
C	-1.690110000	-2.202758000	-0.033360000
C	-0.276998000	-2.725177000	-0.295879000
H	0.077962000	-3.435242000	0.462776000
C	-0.335535000	1.992093000	0.247343000
O	0.347246000	3.092330000	0.526440000
C	-1.894778000	3.588708000	0.165513000
C	-0.627743000	4.128756000	0.806707000
H	-0.691540000	4.229301000	1.899110000
C	-2.116656000	4.106662000	-1.239481000
H	-1.225422000	3.934619000	-1.862889000
H	-2.974513000	3.613409000	-1.712412000
H	-2.313454000	5.187337000	-1.217158000
C	-2.316246000	-2.734935000	1.234192000
H	-1.697572000	-2.498473000	2.112347000
H	-3.318311000	-2.317116000	1.393364000
H	-2.413664000	-3.827392000	1.169789000
N	-1.613644000	2.125295000	0.120672000
N	-1.501491000	-0.727634000	0.025311000
Fe	-3.135842000	0.674623000	0.237791000
Cl	-4.522729000	-1.006996000	-0.833216000
Cl	-3.312825000	0.511851000	2.469037000
H	-2.345479000	-2.425463000	-0.887928000
H	-0.142486000	-3.167237000	-1.291168000
H	-2.775777000	3.766358000	0.796109000
H	-0.262436000	5.066011000	0.373176000
C	0.530575000	0.789478000	0.024764000
C	1.548739000	0.665176000	1.169876000
H	1.045795000	0.504857000	2.133338000
H	2.223286000	-0.177779000	0.983742000
H	2.152086000	1.576781000	1.239802000
C	1.257101000	1.016239000	-1.317406000
H	1.890688000	0.152099000	-1.551100000
H	0.542755000	1.160298000	-2.140038000
H	1.895879000	1.905304000	-1.250562000
Cl	-4.772078000	2.209897000	-0.286756000
S	-3.017453000	0.560439000	-2.712263000
C	-1.804294000	-0.501604000	-3.010143000
N	-0.914095000	-1.256294000	-3.198313000

FeBOX-Cl3-ONO2 (m5)

C	-0.187130000	-0.456482000	-0.160095000
O	0.545405000	-1.446737000	-0.638782000
C	-1.661002000	-2.125663000	-0.285887000
C	-0.372057000	-2.509628000	-1.001797000
H	0.061121000	-3.459973000	-0.669997000
C	-0.260979000	2.014207000	0.125527000
O	0.435830000	3.133259000	0.152072000
C	-1.834108000	3.593101000	-0.177360000
C	-0.508317000	4.233584000	0.211292000
H	-0.483490000	4.627274000	1.236009000
C	-2.245720000	3.859511000	-1.609230000
H	-1.449647000	3.565592000	-2.310638000
H	-3.166846000	3.322387000	-1.867341000
H	-2.433192000	4.933155000	-1.746596000
C	-1.886778000	-2.884357000	1.006310000
H	-0.995934000	-2.828046000	1.650594000
H	-2.736456000	-2.467639000	1.559597000
H	-2.092371000	-3.942961000	0.797454000
N	-1.545171000	2.147534000	0.020590000
N	-1.441601000	-0.693987000	0.016761000
Fe	-3.023214000	0.722717000	0.337030000
Cl	-4.583509000	-0.911106000	0.205717000
Cl	-2.702400000	0.685880000	2.602998000
H	-2.531402000	-2.223869000	-0.950997000
H	-0.449461000	-2.511064000	-2.097761000
H	-2.634015000	3.890901000	0.513899000
H	-0.165683000	5.007167000	-0.485147000
C	0.578216000	0.772832000	0.233528000
C	0.984765000	0.592756000	1.713510000
H	0.106871000	0.476841000	2.362693000
H	1.616352000	-0.299738000	1.812705000
H	1.563754000	1.462904000	2.048329000
C	1.829517000	0.936447000	-0.635958000
H	2.467523000	0.051393000	-0.541210000
H	1.575911000	1.070348000	-1.695919000
H	2.408567000	1.805308000	-0.305940000
Cl	-4.707456000	2.318707000	0.264784000
O	-1.120414000	0.597709000	-2.546761000
N	-2.301828000	0.852738000	-2.791329000
O	-3.187219000	0.754823000	-1.892313000
O	-2.660410000	1.207379000	-3.910141000

FeBOX-Cl3-Me (m5)

C	-0.186001000	-0.443872000	-0.219313000
O	0.587887000	-1.485034000	-0.517592000
C	-1.631485000	-2.134894000	-0.276872000
C	-0.303921000	-2.569577000	-0.875062000
H	0.100926000	-3.497976000	-0.456372000
C	-0.257632000	2.036083000	0.130734000
O	0.450635000	3.142491000	0.348166000
C	-1.791464000	3.642382000	-0.073290000
C	-0.498509000	4.223579000	0.488841000
H	-0.555816000	4.475135000	1.557204000

C	-2.104974000	4.110436000	-1.478349000
H	-1.263058000	3.910968000	-2.158859000
H	-3.001582000	3.615390000	-1.871270000
H	-2.291075000	5.193495000	-1.480798000
C	-1.892671000	-2.704439000	1.101700000
H	-1.029394000	-2.533770000	1.764028000
H	-2.769100000	-2.228074000	1.557689000
H	-2.070436000	-3.787215000	1.043865000
N	-1.521694000	2.181761000	-0.053886000
N	-1.452468000	-0.664062000	-0.167250000
Fe	-3.123654000	0.685159000	0.125103000
Cl	-4.657582000	-1.041883000	-0.115958000
Cl	-2.808455000	0.675439000	2.423649000
H	-2.472132000	-2.350502000	-0.949512000
H	-0.316009000	-2.638428000	-1.972369000
H	-2.641587000	3.846154000	0.591059000
H	-0.115363000	5.089055000	-0.064297000
C	0.579181000	0.789606000	0.161309000
C	1.040423000	0.566802000	1.618584000
H	0.176601000	0.453400000	2.288263000
H	1.658010000	-0.338281000	1.683272000
H	1.641841000	1.420271000	1.955713000
C	1.790923000	0.978560000	-0.759269000
H	2.444711000	0.100516000	-0.713270000
H	1.483544000	1.125432000	-1.803817000
H	2.373642000	1.851603000	-0.444840000
Cl	-4.731468000	2.344644000	-0.075181000
C	-3.076419000	0.609596000	-2.115656000
H	-2.288382000	1.334253000	-2.335527000
H	-2.829939000	-0.432147000	-2.332926000
H	-4.093773000	0.912329000	-2.372244000

FeBOX-Cl3-NO2 (m5)

C	-0.224605000	-0.481154000	-0.043938000
O	0.569648000	-1.537909000	-0.162424000
C	-1.672662000	-2.180607000	0.001472000
C	-0.281992000	-2.690095000	-0.360931000
H	0.085183000	-3.498402000	0.282793000
C	-0.356290000	1.994450000	0.284349000
O	0.287925000	3.065385000	0.729633000
C	-1.908339000	3.589024000	0.177525000
C	-0.721578000	4.071280000	0.994350000
H	-0.901490000	4.075187000	2.078852000
C	-1.991997000	4.240645000	-1.186167000
H	-1.043373000	4.123608000	-1.733525000
H	-2.795627000	3.798756000	-1.784936000
H	-2.189875000	5.316134000	-1.078183000
C	-2.174401000	-2.638109000	1.352540000
H	-1.466839000	-2.371546000	2.152343000
H	-3.143679000	-2.177850000	1.582670000
H	-2.302001000	-3.729357000	1.357544000
N	-1.612288000	2.134949000	0.026621000
N	-1.492729000	-0.703859000	-0.011862000
Fe	-3.184703000	0.658075000	0.118883000
Cl	-4.545547000	-1.023822000	-0.690103000
Cl	-3.351485000	0.677971000	2.367341000
H	-2.400445000	-2.446795000	-0.775248000
H	-0.182879000	-2.995705000	-1.412067000
H	-2.852631000	3.708049000	0.724290000
H	-0.323332000	5.044255000	0.686709000
C	0.532893000	0.811944000	0.042724000
C	1.568590000	0.690388000	1.169481000
H	1.082521000	0.537351000	2.142783000
H	2.238475000	-0.154809000	0.977667000
H	2.175374000	1.600575000	1.224593000
C	1.238410000	1.063569000	-1.306298000
H	1.927623000	0.240161000	-1.530331000
H	0.513836000	1.151429000	-2.128051000
H	1.818480000	1.993828000	-1.256829000
Cl	-4.697286000	2.255257000	-0.574762000
N	-2.477350000	0.502616000	-2.601146000
O	-2.072695000	-0.555086000	-2.971772000
O	-2.656341000	1.549110000	-3.139551000

FeBOX-Cl3-ONO (m5)

C	-0.275564000	-0.449652000	-0.210764000
O	0.484762000	-1.483404000	-0.551518000
C	-1.719868000	-2.144049000	-0.202408000
C	-0.413497000	-2.580087000	-0.847014000
H	0.017140000	-3.497143000	-0.428921000
C	-0.345502000	2.028212000	0.133208000
O	0.359420000	3.141223000	0.298382000
C	-1.898502000	3.619569000	-0.010690000
C	-0.593835000	4.210815000	0.503127000
H	-0.605086000	4.438797000	1.578201000
C	-2.238603000	4.046215000	-1.422884000
H	-1.400749000	3.845030000	-2.109006000
H	-3.131822000	3.525834000	-1.789614000
H	-2.443090000	5.125413000	-1.452025000
C	-1.947602000	-2.732725000	1.173710000
H	-1.080519000	-2.548270000	1.827222000
H	-2.832342000	-2.289127000	1.647446000
H	-2.099106000	-3.818810000	1.105122000
N	-1.624814000	2.156521000	0.030286000
N	-1.539164000	-0.672193000	-0.084273000
Fe	-3.176774000	0.667369000	0.430504000
Cl	-4.673470000	-1.009754000	-0.081972000
Cl	-2.884950000	0.617721000	2.672525000
H	-2.577686000	-2.347586000	-0.855055000
H	-0.470005000	-2.671044000	-1.941250000
H	-2.732723000	3.840637000	0.666492000
H	-0.244529000	5.089936000	-0.050047000
C	0.507302000	0.794901000	0.087051000
C	1.137599000	0.591394000	1.480893000
H	0.363901000	0.461483000	2.250237000
H	1.780237000	-0.297868000	1.476533000
H	1.753664000	1.460088000	1.743367000
C	1.601176000	0.996827000	-0.971490000
H	2.271863000	0.130796000	-0.990395000
H	1.171174000	1.124420000	-1.974600000
H	2.197970000	1.884295000	-0.734101000
Cl	-4.780411000	2.290191000	0.090227000
N	-1.871728000	0.860254000	-2.641641000
O	-3.015091000	0.740253000	-2.315499000
O	-1.319568000	0.965871000	-3.690558000

FeBOX-Cl2 (m5)

C	-0.040196000	-0.506458000	0.123452000
O	0.717907000	-1.593713000	0.253639000
C	-1.553207000	-2.145346000	-0.030857000
C	-0.169956000	-2.738496000	0.255682000
H	-0.097969000	-3.213107000	1.245055000
C	-0.127437000	1.997776000	0.330906000
O	0.553566000	3.103506000	0.633245000
C	-1.763607000	3.511259000	0.516959000
C	-0.401750000	4.190462000	0.693201000
H	-0.272650000	4.701401000	1.654464000
C	-2.623846000	4.080543000	-0.586504000
H	-2.117713000	4.000498000	-1.559605000
H	-3.578914000	3.539379000	-0.652306000
H	-2.848124000	5.139821000	-0.399945000
C	-2.626049000	-2.528499000	0.959314000
H	-2.327265000	-2.263916000	1.984449000
H	-3.564099000	-2.001952000	0.735905000
H	-2.821520000	-3.608899000	0.927137000
N	-1.401443000	2.111969000	0.231608000
N	-1.301772000	-0.694681000	-0.022325000
Fe	-2.785410000	0.715208000	-0.476243000
Cl	-4.570341000	0.882699000	0.948349000
H	-1.887850000	-2.403436000	-1.051081000
H	0.188671000	-3.445011000	-0.501905000
H	-2.332640000	3.517854000	1.462076000
H	-0.157449000	4.893700000	-0.116473000
C	0.732661000	0.788514000	0.058622000
C	1.909769000	0.751415000	1.037041000
H	1.569372000	0.647457000	2.076233000
H	2.569505000	-0.091420000	0.804081000
H	2.496514000	1.673130000	0.957486000
C	1.249461000	0.924678000	-1.390599000
H	1.904805000	0.079721000	-1.638756000
H	0.416162000	0.946228000	-2.106738000
H	1.829317000	1.850878000	-1.496028000
Cl	-2.865167000	0.705781000	-2.764502000

FeBOX-Cl2-OH (m6)

C	-0.034662000	-0.554239000	0.076979000
O	0.667013000	-1.565258000	0.561125000
C	-1.573525000	-2.182160000	0.163402000
C	-0.206601000	-2.723753000	0.590842000
H	-0.186914000	-3.127554000	1.611178000
C	-0.165893000	1.890457000	0.288443000
O	0.434685000	2.911964000	0.887241000
C	-1.871473000	3.307166000	0.554607000
C	-0.585698000	3.912716000	1.137080000
H	-0.620804000	4.087323000	2.219463000
C	-2.499057000	4.128808000	-0.548510000
H	-1.775365000	4.331928000	-1.353208000
H	-3.369004000	3.610653000	-0.975103000
H	-2.841914000	5.097076000	-0.158015000
C	-2.634498000	-2.225507000	1.239468000
H	-2.306951000	-1.687692000	2.141787000
H	-3.567055000	-1.767872000	0.881033000
H	-2.853932000	-3.264788000	1.520290000
N	-1.423737000	1.991374000	0.064852000
N	-1.267683000	-0.786061000	-0.216474000
Fe	-2.730245000	0.537099000	-1.017416000
Cl	-3.782298000	-1.259612000	-2.060205000
Cl	-4.468229000	1.223008000	0.311694000
H	-1.941585000	-2.696431000	-0.735833000
H	0.214105000	-3.469102000	-0.095670000
H	-2.622274000	3.125973000	1.339431000
H	-0.271856000	4.839986000	0.638610000
C	0.716462000	0.739132000	-0.129029000
C	2.030080000	0.741666000	0.644728000
H	1.872116000	0.628940000	1.724820000
H	2.673182000	-0.077761000	0.304436000
H	2.562459000	1.684265000	0.474232000
C	0.982422000	0.884724000	-1.641478000
H	1.593657000	0.045072000	-1.998330000
H	0.040102000	0.905440000	-2.206710000
H	1.529892000	1.816882000	-1.835343000
O	-2.383173000	1.503945000	-2.565376000
H	-1.912884000	2.337183000	-2.427179000

FeBOX-Cl2-F (m6)

C	-0.070172000	-0.522233000	0.087811000
O	0.652568000	-1.570242000	0.440118000
C	-1.600399000	-2.169650000	0.087048000
C	-0.220741000	-2.727956000	0.446996000
H	-0.172034000	-3.171461000	1.450134000
C	-0.165330000	1.942406000	0.327414000
O	0.474193000	3.014731000	0.780051000
C	-1.845468000	3.399413000	0.548340000
C	-0.529801000	4.034498000	1.020782000
H	-0.509149000	4.271175000	2.091868000
C	-2.494195000	4.091282000	-0.629642000
H	-1.811164000	4.134541000	-1.490128000
H	-3.404123000	3.558547000	-0.940251000
H	-2.779368000	5.117994000	-0.362279000
C	-2.647536000	-2.350276000	1.160523000
H	-2.332810000	-1.878737000	2.103362000
H	-3.601039000	-1.905302000	0.847481000
H	-2.815847000	-3.419252000	1.350542000
N	-1.434748000	2.033228000	0.190106000
N	-1.319634000	-0.736760000	-0.155831000
Fe	-2.774674000	0.633321000	-0.805380000
Cl	-4.005618000	-1.036375000	-1.857346000
Cl	-4.281587000	1.051388000	0.884412000
H	-1.966573000	-2.597509000	-0.858898000
H	0.178014000	-3.446052000	-0.279711000
H	-2.571019000	3.326690000	1.373748000
H	-0.240029000	4.929351000	0.454082000
C	0.696162000	0.769349000	-0.061548000
C	1.980502000	0.739812000	0.762989000
H	1.777317000	0.612214000	1.834307000
H	2.628260000	-0.080726000	0.435697000
H	2.529302000	1.678298000	0.627586000
C	1.019740000	0.929825000	-1.563191000
H	1.620155000	0.078875000	-1.911718000
H	0.096769000	0.989248000	-2.154883000
H	1.601331000	1.848193000	-1.718690000
F	-2.238588000	1.422174000	-2.385329000

FeBOX-Cl2-N3 (m6)

C	-0.016687000	-0.591364000	0.223862000
O	0.698874000	-1.639652000	0.596398000
C	-1.551112000	-2.231347000	0.243405000
C	-0.165112000	-2.802044000	0.549711000
H	-0.095464000	-3.307343000	1.521295000
C	-0.120336000	1.890630000	0.326502000
O	0.498055000	2.993705000	0.732583000
C	-1.817429000	3.344514000	0.456339000
C	-0.490958000	4.054145000	0.747326000
H	-0.442475000	4.545097000	1.725856000
C	-2.630295000	3.969362000	-0.653498000
H	-2.054828000	4.017671000	-1.589062000
H	-3.550127000	3.395749000	-0.838446000
H	-2.925890000	4.991425000	-0.379957000
C	-2.547775000	-2.363690000	1.371611000
H	-2.166060000	-1.897416000	2.292399000
H	-3.498887000	-1.881279000	1.110271000
H	-2.746173000	-3.423792000	1.581016000
N	-1.383917000	1.973337000	0.128086000
N	-1.261349000	-0.807068000	-0.040215000
Fe	-2.714979000	0.533353000	-0.766664000
Cl	-3.930461000	-1.167084000	-1.787668000
Cl	-4.222315000	0.978361000	0.911843000
H	-1.967740000	-2.674723000	-0.672659000
H	0.224761000	-3.470904000	-0.228032000
H	-2.440034000	3.271952000	1.364279000
H	-0.210062000	4.779683000	-0.029841000
C	0.765613000	0.693286000	0.103952000
C	1.927137000	0.703456000	1.100468000
H	1.577379000	0.636959000	2.139007000
H	2.600509000	-0.138020000	0.905792000
H	2.502690000	1.628758000	0.988999000
C	1.313662000	0.770708000	-1.336798000
H	1.974768000	-0.084423000	-1.529993000
H	0.502023000	0.768781000	-2.076517000
H	1.900075000	1.691025000	-1.458115000
N	-1.970133000	1.134186000	-2.532955000
N	-2.306643000	0.824499000	-3.641367000
N	-2.599748000	0.541975000	-4.725378000

FeBOX-Cl2-SCN (m6)

C	-0.113042000	-0.509538000	0.019691000
O	0.633942000	-1.597448000	0.015927000
C	-1.649429000	-2.156250000	-0.025803000
C	-0.241759000	-2.744265000	-0.124153000
H	0.005749000	-3.449588000	0.678731000
C	-0.161088000	1.990866000	0.243702000
O	0.503549000	3.098927000	0.546274000
C	-1.807884000	3.508998000	0.343743000
C	-0.450326000	4.192279000	0.525989000
H	-0.345696000	4.746330000	1.465323000
C	-2.665637000	4.059625000	-0.770952000
H	-2.123282000	4.073609000	-1.727604000
H	-3.579472000	3.456308000	-0.889817000
H	-2.979945000	5.087345000	-0.545222000
C	-2.416796000	-2.555924000	1.213297000
H	-1.866514000	-2.284916000	2.126513000
H	-3.400311000	-2.068325000	1.240539000
H	-2.578789000	-3.642249000	1.220294000
N	-1.428066000	2.107118000	0.090582000
N	-1.391890000	-0.695519000	-0.041214000
Fe	-2.907308000	0.668329000	-0.384666000
Cl	-4.475709000	-0.775023000	-1.198895000
Cl	-3.887271000	1.089878000	1.648126000
H	-2.231306000	-2.397831000	-0.928407000
H	-0.022236000	-3.206334000	-1.094748000
H	-2.383966000	3.513116000	1.284232000
H	-0.180723000	4.852431000	-0.310724000
C	0.690458000	0.764780000	0.084757000
C	1.660369000	0.665783000	1.273240000
H	1.121576000	0.551940000	2.223499000
H	2.329080000	-0.192628000	1.145310000
H	2.274681000	1.571306000	1.328662000
C	1.480102000	0.910541000	-1.230928000
H	2.122272000	0.034610000	-1.383814000
H	0.808985000	1.013666000	-2.094764000
H	2.119014000	1.800873000	-1.178605000
S	-2.109597000	1.127780000	-2.852160000
C	-1.617518000	-0.433408000	-3.089237000
N	-1.225863000	-1.534558000	-3.251641000

FeBOX-Cl2-ONO2 (m6)

C	0.042093000	-0.685108000	0.178280000
O	0.765975000	-1.719562000	0.570661000
C	-1.481148000	-2.336821000	0.238816000
C	-0.100742000	-2.879879000	0.612770000
H	-0.048690000	-3.294047000	1.628173000
C	-0.098123000	1.804228000	0.141871000
O	0.484025000	2.891328000	0.633061000
C	-1.793197000	3.258828000	0.235791000
C	-0.476185000	3.966469000	0.531533000
H	-0.459152000	4.529670000	1.470890000
C	-2.660685000	3.996047000	-0.750838000
H	-2.175620000	4.083676000	-1.733564000
H	-3.632456000	3.506285000	-0.881480000
H	-2.844769000	5.011896000	-0.372897000
C	-2.526528000	-2.465643000	1.321801000
H	-2.207169000	-1.957270000	2.243684000
H	-3.480733000	-2.029339000	0.996713000
H	-2.702406000	-3.524295000	1.556538000
N	-1.337783000	1.908696000	-0.191665000
N	-1.204311000	-0.913748000	-0.063170000
Fe	-2.665604000	0.339074000	-0.891815000
Cl	-3.740740000	-1.390640000	-1.963475000
Cl	-4.209428000	0.911728000	0.662723000
H	-1.849911000	-2.802008000	-0.687004000
H	0.298390000	-3.617035000	-0.094424000
H	-2.367935000	3.098968000	1.165393000
H	-0.153298000	4.625893000	-0.288709000
C	0.812955000	0.604379000	0.050612000
C	1.866621000	0.672880000	1.163693000
H	1.411082000	0.657827000	2.162473000
H	2.553408000	-0.176227000	1.083333000
H	2.454540000	1.591151000	1.065271000

C	1.525368000	0.623981000	-1.317477000
H	2.212060000	-0.229666000	-1.388252000
H	0.814187000	0.580252000	-2.149733000
H	2.116754000	1.544879000	-1.405939000
O	-3.157463000	1.677825000	-2.487321000
N	-2.129460000	1.419226000	-3.201345000
O	-1.911916000	1.993753000	-4.241118000
O	-1.370029000	0.527005000	-2.728630000

FeBOX-Cl2-Me (m6)

C	0.042523000	-0.603777000	0.172182000
O	0.709121000	-1.561961000	0.796185000
C	-1.499955000	-2.223483000	0.281171000
C	-0.193753000	-2.689656000	0.936604000
H	-0.291625000	-2.901388000	2.010138000
C	-0.088043000	1.833979000	0.289009000
O	0.483194000	2.816464000	0.978289000
C	-1.809600000	3.223804000	0.569232000
C	-0.534055000	3.832284000	1.171705000
H	-0.596011000	4.043156000	2.245989000
C	-2.490278000	4.103153000	-0.452790000
H	-1.802688000	4.372569000	-1.267849000
H	-3.365544000	3.595079000	-0.880453000
H	-2.836089000	5.033916000	0.018688000
C	-2.697069000	-2.224358000	1.201915000
H	-2.526201000	-1.575664000	2.074101000
H	-3.587190000	-1.864684000	0.669515000
H	-2.900903000	-3.241431000	1.564580000
N	-1.328177000	1.956594000	-0.004701000
N	-1.170698000	-0.857603000	-0.170698000
Fe	-2.550117000	0.456865000	-1.159905000
Cl	-3.540357000	-1.417991000	-2.187341000
Cl	-4.334022000	1.061444000	0.159594000
H	-1.731115000	-2.820198000	-0.615228000
H	0.274786000	-3.551908000	0.446749000
H	-2.536735000	2.963388000	1.356304000
H	-0.199364000	4.740970000	0.650902000
C	0.792435000	0.675130000	-0.118745000
C	2.132995000	0.711133000	0.603190000
H	2.016573000	0.666823000	1.693165000
H	2.753167000	-0.137297000	0.291539000
H	2.671028000	1.633391000	0.354433000
C	0.995464000	0.756112000	-1.643333000
H	1.625015000	-0.076679000	-1.984426000
H	0.033472000	0.707254000	-2.173302000
H	1.494598000	1.698253000	-1.907548000
C	-2.440968000	1.583619000	-2.920881000
H	-1.861018000	2.514343000	-2.823202000
H	-2.004771000	0.958265000	-3.716684000
H	-3.482423000	1.824098000	-3.195478000

FeBOX-Cl2-NO2 (m6)

C	-0.078046000	-0.554050000	0.031322000
O	0.663247000	-1.648169000	0.131506000
C	-1.626316000	-2.182286000	0.019692000
C	-0.225304000	-2.789836000	0.128966000
H	-0.057214000	-3.343000000	1.062741000
C	-0.116485000	1.947327000	0.187682000
O	0.549932000	3.035582000	0.558301000
C	-1.751495000	3.461983000	0.385348000
C	-0.390994000	4.137285000	0.536244000
H	-0.265544000	4.708214000	1.462529000
C	-2.680128000	4.115787000	-0.605443000
H	-2.213445000	4.206178000	-1.596248000
H	-3.611064000	3.540055000	-0.708935000
H	-2.949696000	5.124014000	-0.262222000
C	-2.545568000	-2.509600000	1.172519000
H	-2.115198000	-2.176084000	2.128193000
H	-3.523895000	-2.027980000	1.041159000
H	-2.709302000	-3.594383000	1.230221000
N	-1.380305000	2.081793000	0.006964000
N	-1.352089000	-0.729241000	-0.053771000
Fe	-2.865887000	0.643648000	-0.535479000
Cl	-4.291575000	-0.860201000	-1.540828000
Cl	-4.115335000	1.225971000	1.303063000
H	-2.106789000	-2.471345000	-0.927710000
H	0.053500000	-3.429226000	-0.717185000
H	-2.263521000	3.380304000	1.359591000
H	-0.131729000	4.779012000	-0.319091000
C	0.732386000	0.718135000	0.017562000
C	1.763951000	0.657691000	1.154472000
H	1.279475000	0.584307000	2.137388000
H	2.418801000	-0.210617000	1.024499000
H	2.388976000	1.556995000	1.142689000
C	1.459353000	0.823790000	-1.337780000
H	2.121653000	-0.040073000	-1.476095000
H	0.748974000	0.863614000	-2.172278000
H	2.074584000	1.732559000	-1.355341000
N	-2.573095000	1.414645000	-2.522849000
O	-1.573832000	0.721561000	-2.763648000
O	-3.097613000	2.141826000	-3.339512000

FeBOX-Cl2-ONO (m6)

C	-0.081117000	-0.521786000	0.059381000
O	0.667097000	-1.608734000	0.152642000
C	-1.612188000	-2.169461000	-0.027155000
C	-0.202550000	-2.762046000	0.048418000
H	-0.032642000	-3.386614000	0.934873000
C	-0.140091000	1.980913000	0.257072000
O	0.524643000	3.088086000	0.574208000
C	-1.787457000	3.490762000	0.375077000
C	-0.432244000	4.177364000	0.563323000
H	-0.329912000	4.724965000	1.506930000
C	-2.643462000	4.047788000	-0.736276000
H	-2.126786000	3.989840000	-1.704546000
H	-3.585185000	3.483515000	-0.811250000
H	-2.901205000	5.097043000	-0.539745000
C	-2.505466000	-2.533728000	1.135843000
H	-2.054484000	-2.226549000	2.091029000
H	-3.488225000	-2.053412000	1.042564000
H	-2.660456000	-3.620934000	1.163904000
N	-1.406075000	2.094503000	0.105359000
N	-1.351554000	-0.712098000	-0.068669000
Fe	-2.861795000	0.655299000	-0.507674000
Cl	-4.412472000	-0.801577000	-1.388613000
Cl	-3.948950000	1.044069000	1.495129000

H	-2.103154000	-2.441371000	-0.974499000
H	0.097005000	-3.322042000	-0.845879000
H	-2.366147000	3.483047000	1.314010000
H	-0.166078000	4.846214000	-0.267931000
C	0.716554000	0.758727000	0.080943000
C	1.741281000	0.688782000	1.222070000
H	1.249346000	0.588289000	2.198878000
H	2.411349000	-0.166186000	1.080880000
H	2.351154000	1.598585000	1.234144000
C	1.442788000	0.889739000	-1.273372000
H	2.104549000	0.029095000	-1.431878000
H	0.733045000	0.947034000	-2.109388000
H	2.056255000	1.799549000	-1.275797000
O	-2.146657000	1.082013000	-2.349397000
N	-1.823262000	0.082023000	-3.135159000
O	-1.381947000	0.415569000	-4.206658000

CoBOX-Cl2 (m4)

C	0.019671000	-0.505726000	0.189169000
O	0.775739000	-1.593303000	0.312646000
C	-1.493135000	-2.149462000	0.034971000
C	-0.121828000	-2.731130000	0.382182000
H	-0.070128000	-3.131147000	1.405710000
C	-0.062186000	2.004189000	0.375047000
O	0.615904000	3.105441000	0.690078000
C	-1.702866000	3.514268000	0.579226000
C	-0.340349000	4.195555000	0.736436000
H	-0.203533000	4.723304000	1.687076000
C	-2.592891000	4.087471000	-0.497296000
H	-2.097290000	4.056783000	-1.478834000
H	-3.525150000	3.508948000	-0.567453000
H	-2.855176000	5.130684000	-0.275123000
C	-2.618867000	-2.539107000	0.959287000
H	-2.392353000	-2.257151000	1.998178000
H	-3.547311000	-2.029554000	0.666390000
H	-2.797045000	-3.622366000	0.926875000
N	-1.335412000	2.123644000	0.271228000
N	-1.245125000	-0.699282000	0.066034000
Co	-2.687487000	0.687447000	-0.181816000
Cl	-4.344531000	0.870122000	1.369888000
H	-1.772851000	-2.397070000	-1.004522000
H	0.243297000	-3.491553000	-0.316861000
H	-2.253185000	3.499664000	1.535605000
H	-0.100459000	4.882267000	-0.088626000
C	0.785668000	0.791087000	0.081953000
C	2.013150000	0.766291000	0.993515000
H	1.731520000	0.671743000	2.050787000
H	2.663034000	-0.076136000	0.732831000
H	2.590397000	1.689519000	0.872476000
C	1.215320000	0.913541000	-1.397865000
H	1.851793000	0.064447000	-1.678648000
H	0.336960000	0.931726000	-2.058068000
H	1.789140000	1.837624000	-1.545990000
Cl	-3.346922000	0.694923000	-2.360952000

CoBOX-Cl2-OH (m3)

C	-0.042722000	-0.402670000	-0.109669000
O	0.693365000	-1.455302000	-0.455182000
C	-1.602358000	-1.988965000	-0.437901000
C	-0.201971000	-2.594477000	-0.535625000
H	0.036139000	-3.263128000	0.304866000
C	-0.139107000	2.079945000	0.135786000
O	0.528709000	3.228110000	0.178689000
C	-1.793999000	3.583122000	0.019772000
C	-0.453991000	4.293660000	0.235287000
H	-0.358475000	4.778210000	1.215850000
C	-2.466699000	3.885282000	-1.300338000
H	-1.773254000	3.727979000	-2.139888000
H	-3.335661000	3.228677000	-1.438657000
H	-2.812647000	4.927445000	-1.332377000
C	-2.529445000	-2.675544000	0.533136000
H	-2.110754000	-2.676648000	1.548919000
H	-3.503666000	-2.167040000	0.559901000
H	-2.703181000	-3.715827000	0.226148000
N	-1.419609000	2.159722000	0.084193000
N	-1.306920000	-0.603908000	-0.035426000
Co	-2.758647000	0.625750000	0.399280000
Cl	-4.626654000	1.888211000	0.770798000
Cl	-2.307097000	0.391928000	2.634569000
H	-2.086126000	-1.931892000	-1.426634000
H	0.001048000	-3.117809000	-1.476378000
H	-2.491998000	3.784113000	0.846458000
H	-0.204258000	5.021468000	-0.546961000
C	0.733218000	0.853017000	0.180004000
C	1.324888000	0.726877000	1.597727000
H	0.531707000	0.638287000	2.352282000
H	1.967107000	-0.161632000	1.654786000
H	1.938244000	1.607503000	1.826810000
C	1.861733000	1.006355000	-0.848730000
H	2.531310000	0.139767000	-0.809289000
H	1.468957000	1.094079000	-1.870852000
H	2.451758000	1.902681000	-0.626840000
O	-3.501501000	0.110567000	-1.166015000
H	-4.352041000	0.577878000	-1.209277000

CoBOX-Cl3 (m3)

C	-0.078996000	-0.420885000	-0.107552000
O	0.655374000	-1.472657000	-0.445559000
C	-1.634562000	-2.025543000	-0.388742000
C	-0.231113000	-2.621547000	-0.482232000
H	0.022789000	-3.261650000	0.375470000
C	-0.189754000	2.057005000	0.121205000
O	0.461657000	3.205485000	0.046601000
C	-1.870759000	3.548259000	0.091584000
C	-0.521217000	4.271848000	0.105930000
H	-0.329013000	4.844344000	1.021967000
C	-2.701045000	3.793362000	-1.146891000
H	-2.148349000	3.511008000	-2.055511000
H	-3.630994000	3.212217000	-1.107480000
H	-2.962424000	4.857850000	-1.222198000
C	-2.553371000	-2.711271000	0.590170000
H	-2.120525000	-2.735681000	1.599323000
H	-3.522379000	-2.194228000	0.640766000
H	-2.742749000	-3.743569000	0.265868000
N	-1.474406000	2.127301000	0.189769000
N	-1.343377000	-0.625534000	-0.017793000

Co	-2.744460000	0.598744000	0.565270000
Cl	-4.446729000	1.917331000	1.284595000
Cl	-2.205383000	0.078738000	2.714556000
H	-2.118510000	-1.986867000	-1.377948000
H	-0.034373000	-3.170098000	-1.409423000
H	-2.462938000	3.787859000	0.986566000
H	-0.357073000	4.921912000	-0.762854000
C	0.693878000	0.839589000	0.161017000
C	1.298170000	0.730973000	1.575915000
H	0.513832000	0.615073000	2.335540000
H	1.967981000	-0.137157000	1.625815000
H	1.885044000	1.630670000	1.800553000
C	1.812381000	0.989752000	-0.877526000
H	2.481852000	0.123352000	-0.838774000
H	1.411609000	1.074577000	-1.896478000
H	2.403889000	1.886444000	-0.661930000
Cl	-3.832118000	0.181741000	-1.377866000

CoBOX-Cl2-F (m3)

C	-0.065644000	-0.399625000	-0.099191000
O	0.633973000	-1.440841000	-0.528876000
C	-1.664564000	-1.955978000	-0.411120000
C	-0.277040000	-2.572815000	-0.578660000
H	-0.007189000	-3.248116000	0.246698000
C	-0.168479000	2.067257000	0.131103000
O	0.468928000	3.216729000	-0.013486000
C	-1.863344000	3.539608000	0.069047000
C	-0.522533000	4.276759000	0.060958000
H	-0.321462000	4.847275000	0.976661000
C	-2.681091000	3.691386000	-1.193362000
H	-2.089302000	3.424703000	-2.081627000
H	-3.564341000	3.040058000	-1.153404000
H	-3.024190000	4.728649000	-1.308004000
C	-2.574539000	-2.682861000	0.546184000
H	-2.119471000	-2.771228000	1.542115000
H	-3.532053000	-2.153066000	0.642201000
H	-2.786439000	-3.693036000	0.170433000
N	-1.450938000	2.131814000	0.240506000
N	-1.325800000	-0.595237000	0.045506000
Co	-2.711186000	0.608236000	0.676633000
Cl	-4.417522000	1.936553000	1.363536000
Cl	-2.162228000	0.109845000	2.826316000
H	-2.172762000	-1.839705000	-1.382531000
H	-0.121022000	-3.088729000	-1.531773000
H	-2.470376000	3.819878000	0.941197000
H	-0.375579000	4.930228000	-0.807800000
C	0.718001000	0.850605000	0.189225000
C	1.289822000	0.737838000	1.616887000
H	0.487581000	0.629756000	2.358429000
H	1.950203000	-0.136943000	1.680705000
H	1.881145000	1.631769000	1.853507000
C	1.858805000	0.996280000	-0.822867000
H	2.524076000	0.127372000	-0.768692000
H	1.483089000	1.082551000	-1.851101000
H	2.448770000	1.890460000	-0.593388000
F	-3.681731000	0.090314000	-0.769951000

CoBOX-Cl2-N3 (m3)

C	0.095820000	-0.445451000	-0.075682000
O	0.898017000	-1.460584000	-0.371197000
C	-1.368123000	-2.098531000	-0.510559000
C	0.062726000	-2.638324000	-0.524121000
H	0.277394000	-3.306952000	0.322333000
C	-0.125445000	2.028403000	0.117278000
O	0.496849000	3.197374000	0.088584000
C	-1.831332000	3.461555000	-0.139767000
C	-0.522819000	4.229345000	0.064089000
H	-0.466009000	4.768389000	1.018831000
C	-2.464471000	3.656349000	-1.499579000
H	-1.774138000	3.360857000	-2.303930000
H	-3.385684000	3.066256000	-1.588390000
H	-2.726906000	4.712989000	-1.647582000
C	-2.319603000	-2.836713000	0.396715000
H	-1.955179000	-2.845317000	1.433036000
H	-3.312369000	-2.364604000	0.385866000
H	-2.438596000	-3.874650000	0.057989000
N	-1.410464000	2.057360000	0.045034000
N	-1.161361000	-0.701176000	-0.088651000
Co	-2.687598000	0.479935000	0.257911000
Cl	-4.560136000	1.714015000	0.613925000
Cl	-2.503322000	0.049819000	2.480096000
H	-1.788678000	-2.059803000	-1.529695000
H	0.351782000	-3.136451000	-1.455928000
H	-2.560539000	3.702940000	0.647201000
H	-0.277895000	4.920847000	-0.751439000
C	0.785048000	0.840718000	0.285185000
C	1.180604000	0.755068000	1.773871000
H	0.297167000	0.609445000	2.409468000
H	1.868929000	-0.086398000	1.927102000
H	1.693409000	1.676815000	2.077907000
C	2.036134000	1.022770000	-0.580852000
H	2.720348000	0.179889000	-0.433498000
H	1.787227000	1.084070000	-1.648679000
H	2.563130000	1.939615000	-0.294652000
N	-3.255879000	0.064430000	-1.538733000
N	-4.268887000	0.449592000	-2.065123000
N	-5.211081000	0.784723000	-2.642909000

CoBOX-Cl2-SCN (m3)

C	-0.113580000	-0.515898000	-0.102526000
O	0.628553000	-1.576998000	-0.375175000
C	-1.660095000	-2.153657000	-0.255437000
C	-0.252114000	-2.728043000	-0.437333000
H	0.044745000	-3.416127000	0.366117000
C	-0.217326000	1.980478000	0.074442000
O	0.448184000	3.123107000	0.128116000
C	-1.878752000	3.492579000	0.025251000
C	-0.526948000	4.195244000	0.191643000
H	-0.398690000	4.700274000	1.157316000
C	-2.611413000	3.869314000	-1.241829000
H	-2.003984000	3.642796000	-2.130856000
H	-3.565917000	3.334291000	-1.317219000
H	-2.824412000	4.947212000	-1.241156000
C	-2.472567000	-2.780224000	0.849875000
H	-1.964161000	-2.694394000	1.819373000
H	-3.458547000	-2.300906000	0.932237000

H	-2.636216000	-3.844539000	0.633688000
N	-1.501418000	2.064253000	0.037816000
N	-1.378600000	-0.723989000	-0.008480000
Co	-2.805588000	0.544802000	0.341815000
Cl	-4.715517000	1.729964000	0.674957000
Cl	-2.463581000	0.264464000	2.564740000
H	-2.226622000	-2.218281000	-1.199387000
H	-0.093660000	-3.215272000	-1.406240000
H	-2.529854000	3.665174000	0.895964000
H	-0.295046000	4.902388000	-0.614668000
C	0.658266000	0.757176000	0.104356000
C	1.314020000	0.673933000	1.498216000
H	0.556320000	0.585906000	2.288445000
H	1.975054000	-0.200413000	1.546263000
H	1.916798000	1.571706000	1.682352000
C	1.734034000	0.887317000	-0.983076000
H	2.420527000	0.034486000	-0.937208000
H	1.290554000	0.922865000	-1.987364000
H	2.316301000	1.802029000	-0.824774000
S	-3.583857000	0.322560000	-1.858096000
C	-2.183707000	-0.095371000	-2.657361000
N	-1.190590000	-0.374428000	-3.222801000

CoBOX-Cl2-ONO2 (m1)

C	-0.004066000	-0.451776000	-0.127262000
O	0.720907000	-1.525589000	-0.398445000
C	-1.587182000	-2.024923000	-0.430238000
C	-0.193656000	-2.656609000	-0.429116000
H	0.000194000	-3.260163000	0.469431000
C	-0.170981000	2.009621000	0.116795000
O	0.444887000	3.178620000	0.079456000
C	-1.886149000	3.485791000	0.062267000
C	-0.561517000	4.204750000	0.295751000
H	-0.436879000	4.570053000	1.323788000
C	-2.513288000	3.758458000	-1.285877000
H	-1.795976000	3.584448000	-2.100913000
H	-3.391839000	3.120329000	-1.440284000
H	-2.839821000	4.806153000	-1.334559000
C	-2.579616000	-2.638462000	0.525068000
H	-2.205909000	-2.604124000	1.557142000
H	-3.537804000	-2.099487000	0.488105000
H	-2.774499000	-3.684343000	0.253778000
N	-1.457527000	2.068365000	0.178014000
N	-1.271783000	-0.627920000	-0.082559000
Co	-2.657444000	0.637855000	0.291289000
Cl	-4.555630000	1.809822000	0.680162000
Cl	-2.263663000	0.344787000	2.505925000
H	-2.011296000	-2.020470000	-1.450351000
H	0.034491000	-3.250353000	-1.319878000
H	-2.601562000	3.690300000	0.868957000
H	-0.366578000	5.021407000	-0.407607000
C	0.746966000	0.819711000	0.115929000
C	1.408101000	0.729237000	1.506079000
H	0.653692000	0.601585000	2.294157000
H	2.098180000	-0.123498000	1.532790000
H	1.982384000	1.642555000	1.706011000
C	1.813143000	1.001729000	-0.972338000
H	2.500314000	0.147861000	-0.962966000
H	1.364722000	1.079102000	-1.971361000
H	2.395860000	1.909197000	-0.778122000
O	-1.239288000	0.926013000	-2.465023000
N	-2.403221000	0.572357000	-2.596548000
O	-3.207444000	0.557192000	-1.556503000
O	-2.912892000	0.236870000	-3.652046000

CoBOX-Cl2-Me (m3)

C	0.004898000	-0.447857000	-0.114464000
O	0.770855000	-1.533364000	-0.221355000
C	-1.507580000	-2.069876000	-0.460548000
C	-0.117498000	-2.678424000	-0.274729000
H	-0.011529000	-3.225172000	0.674229000
C	-0.115023000	2.055264000	0.086201000
O	0.564406000	3.197438000	0.242730000
C	-1.755493000	3.566844000	0.094414000
C	-0.397490000	4.273429000	0.175266000
H	-0.266321000	4.904252000	1.062595000
C	-2.639170000	4.038853000	-1.035089000
H	-2.146994000	3.896147000	-2.008649000
H	-3.590847000	3.490908000	-1.036068000
H	-2.864088000	5.108495000	-0.922400000
C	-2.584017000	-2.612256000	0.447102000
H	-2.304254000	-2.494156000	1.502827000
H	-3.529756000	-2.071582000	0.287606000
H	-2.765021000	-3.676552000	0.244953000
N	-1.385307000	2.149020000	-0.035469000
N	-1.258186000	-0.642733000	-0.204677000
Co	-2.780662000	0.612983000	0.134901000
Cl	-4.910397000	1.367115000	0.318718000
Cl	-2.339269000	0.443271000	2.440694000
H	-1.835746000	-2.157861000	-1.511640000
H	0.209574000	-3.324641000	-1.096432000
H	-2.305996000	3.658044000	1.047900000
H	-0.171930000	4.871442000	-0.720106000
C	0.760014000	0.828759000	0.131720000
C	1.373027000	0.740387000	1.543125000
H	0.583655000	0.660544000	2.303439000
H	2.027009000	-0.137775000	1.615526000
H	1.974117000	1.634759000	1.747825000
C	1.870096000	0.960806000	-0.921418000
H	2.547161000	0.099973000	-0.870245000
H	1.457362000	1.016997000	-1.938306000
H	2.456281000	1.868255000	-0.735878000
C	-3.085909000	0.456892000	-1.834722000
H	-2.117961000	0.358141000	-2.341771000
H	-3.726163000	-0.424478000	-1.984614000
H	-3.603259000	1.373341000	-2.139099000

CoBOX-Cl2-NO2 (m1)

C	-0.010231000	-0.457645000	-0.004728000
O	0.749080000	-1.542129000	-0.058670000
C	-1.532877000	-2.093120000	-0.272077000
C	-0.145962000	-2.686490000	-0.031038000
H	-0.049297000	-3.156008000	0.958981000
C	-0.225863000	2.005603000	0.223611000
O	0.370948000	3.161749000	0.464032000
C	-1.939553000	3.474879000	0.190872000

C	-0.672427000	4.138646000	0.720567000
H	-0.686937000	4.320582000	1.803566000
C	-2.378579000	3.998034000	-1.157005000
H	-1.555062000	3.952377000	-1.884585000
H	-3.224576000	3.420889000	-1.545444000
H	-2.691659000	5.046741000	-1.059440000
C	-2.625152000	-2.633765000	0.614546000
H	-2.385710000	-2.486262000	1.675517000
H	-3.582456000	-2.134190000	0.407525000
H	-2.761696000	-3.707159000	0.426678000
N	-1.508777000	2.054641000	0.102995000
N	-1.276081000	-0.651139000	-0.064249000
Co	-2.721179000	0.609878000	0.057101000
Cl	-4.672689000	1.765579000	0.051335000
Cl	-2.677128000	0.396094000	2.361994000
H	-1.825727000	-2.209752000	-1.329962000
H	0.183018000	-3.392911000	-0.799964000
H	-2.759967000	3.534008000	0.918377000
H	-0.402881000	5.064196000	0.199552000
C	0.716948000	0.847357000	0.067967000
C	1.700820000	0.815380000	1.243640000
H	1.172088000	0.691137000	2.198739000
H	2.402383000	-0.018301000	1.122704000
H	2.279063000	1.745458000	1.282852000
C	1.458802000	1.045885000	-1.269262000
H	2.165850000	0.223445000	-1.433174000
H	0.748866000	1.076110000	-2.107709000
H	2.022588000	1.986636000	-1.249701000
N	-2.849264000	0.486307000	-1.825376000
O	-3.583661000	-0.467866000	-2.073686000
O	-2.280257000	1.155258000	-2.661347000

CoBOX-Cl2-ONO (m1)

C	-0.020321000	-0.446159000	-0.113536000
O	0.707069000	-1.526608000	-0.356403000
C	-1.601118000	-2.021947000	-0.405465000
C	-0.209341000	-2.656259000	-0.370483000
H	-0.029271000	-3.236813000	0.546159000
C	-0.197570000	2.013856000	0.130039000
O	0.412420000	3.185847000	0.119652000
C	-1.925416000	3.479663000	0.112091000
C	-0.599360000	4.208340000	0.321286000
H	-0.467870000	4.600688000	1.338061000
C	-2.602461000	3.795207000	-1.202080000
H	-1.934803000	3.593552000	-2.053206000
H	-3.520130000	3.207088000	-1.321320000
H	-2.870979000	4.860294000	-1.224997000
C	-2.607847000	-2.614800000	0.547668000
H	-2.243011000	-2.574998000	1.582739000
H	-3.559740000	-2.064754000	0.499732000
H	-2.813098000	-3.660972000	0.285140000
N	-1.485968000	2.061834000	0.179207000
N	-1.288336000	-0.618986000	-0.081257000
Co	-2.682254000	0.626913000	0.306106000
Cl	-4.596308000	1.756088000	0.745549000
Cl	-2.257502000	0.312980000	2.528040000
H	-2.010907000	-2.030975000	-1.431152000
H	0.029489000	-3.274037000	-1.241881000
H	-2.615765000	3.654325000	0.947919000
H	-0.414809000	5.009638000	-0.402852000
C	0.729819000	0.831013000	0.099279000
C	1.457729000	0.751375000	1.453782000
H	0.742696000	0.610422000	2.275567000
H	2.161689000	-0.090096000	1.447247000
H	2.026264000	1.673276000	1.628775000
C	1.735001000	1.017267000	-1.046345000
H	2.427552000	0.168115000	-1.072424000
H	1.226258000	1.085364000	-2.017587000
H	2.318805000	1.931529000	-0.891092000
N	-2.140318000	0.764717000	-2.392202000
O	-3.147219000	0.678164000	-1.545718000
O	-2.493151000	0.723446000	-3.546534000

NiBOX-Cl3 (m2)

C	-0.119332000	-0.516644000	0.009024000
O	0.615444000	-1.623586000	0.016774000
C	-1.690697000	-2.115803000	-0.045281000
C	-0.294109000	-2.742045000	-0.141751000
H	-0.077464000	-3.464735000	0.654931000
C	-0.190016000	1.970801000	0.236538000
O	0.461523000	3.081657000	0.550600000
C	-1.857667000	3.489508000	0.374063000
C	-0.498404000	4.171115000	0.538421000
H	-0.385265000	4.728073000	1.474747000
C	-2.730748000	4.061840000	-0.714479000
H	-2.223844000	4.029186000	-1.688169000
H	-3.670830000	3.495766000	-0.794750000
H	-2.986887000	5.104576000	-0.484669000
C	-2.464812000	-2.505599000	1.193573000
H	-1.891293000	-2.274040000	2.103732000
H	-3.419732000	-1.967113000	1.241021000
H	-2.672962000	-3.584406000	1.186089000
N	-1.459871000	2.095645000	0.096219000
N	-1.390376000	-0.673246000	-0.052446000
Ni	-2.766881000	0.767981000	-0.404137000
Cl	-4.490344000	-0.508783000	-1.037782000
Cl	-3.640083000	0.975207000	1.674148000
H	-2.289998000	-2.333360000	-0.942754000
H	-0.080498000	-3.206772000	-1.113059000
H	-2.414960000	3.471277000	1.324502000
H	-0.240981000	4.828284000	-0.304396000
C	0.678441000	0.757571000	0.058458000
C	1.673079000	0.673499000	1.225664000
H	1.155845000	0.572026000	2.189221000
H	2.332307000	-0.191703000	1.092490000
H	2.296184000	1.574207000	1.257954000
C	1.435694000	0.914570000	-1.274230000
H	2.103979000	0.057896000	-1.426284000
H	0.738947000	0.976904000	-2.120678000
H	2.046070000	1.826172000	-1.251764000
Cl	-2.166784000	1.078089000	-2.570965000

NiBOX-Cl3-OH (m1)

C	-0.172370000	-0.476930000	0.017463000
O	0.602670000	-1.546210000	0.052753000
C	-1.673131000	-2.160085000	0.069790000

C	-0.253833000	-2.708659000	-0.082510000
H	0.027343000	-3.422805000	0.700449000
C	-0.331371000	1.964826000	0.328109000
O	0.251050000	3.028075000	0.859584000
C	-1.944943000	3.516992000	0.260360000
C	-0.825418000	3.956147000	1.185623000
H	-1.055775000	3.820879000	2.251856000
C	-1.910810000	4.229686000	-1.077112000
H	-0.886318000	4.246358000	-1.480040000
H	-2.560334000	3.736909000	-1.808751000
H	-2.243086000	5.270429000	-0.961096000
C	-2.348459000	-2.562975000	1.359133000
H	-1.750710000	-2.259298000	2.231159000
H	-3.342434000	-2.110134000	1.443414000
H	-2.458559000	-3.656112000	1.384598000
N	-1.585206000	2.089369000	0.068809000
N	-1.441560000	-0.696036000	-0.006163000
Ni	-2.838544000	0.652911000	-0.392172000
Cl	-4.369175000	-0.938303000	-0.885508000
Cl	-3.428412000	0.711239000	1.838698000
H	-2.293418000	-2.447032000	-0.788920000
H	-0.049918000	-3.151441000	-1.065957000
H	-2.927059000	3.582863000	0.743817000
H	-0.462922000	4.973806000	1.007464000
C	0.572096000	0.823861000	-0.018321000
C	1.767969000	0.768446000	0.933507000
H	1.454115000	0.594846000	1.971329000
H	2.450790000	-0.034961000	0.637258000
H	2.318972000	1.714518000	0.893389000
C	1.041883000	1.064195000	-1.470517000
H	1.721423000	0.258814000	-1.777765000
H	0.181427000	1.097548000	-2.152156000
H	1.588935000	2.014725000	-1.529318000
Cl	-4.466908000	2.111020000	-0.961497000
O	-2.200783000	0.656572000	-2.147323000
H	-2.902123000	1.120492000	-2.632912000

NiBOX-Cl4 (m1)

C	-0.226383000	-0.464855000	-0.092806000
O	0.528329000	-1.532805000	-0.291527000
C	-1.721104000	-2.145371000	-0.110367000
C	-0.371456000	-2.624618000	-0.622471000
H	-0.003586000	-3.529618000	-0.127397000
C	-0.326208000	2.000431000	0.231431000
O	0.331790000	3.119058000	0.486433000
C	-1.923490000	3.575865000	0.068604000
C	-0.667192000	4.150742000	0.705436000
H	-0.748462000	4.298169000	1.790944000
C	-2.169554000	4.068467000	-1.340174000
H	-1.281069000	3.912046000	-1.970528000
H	-3.014028000	3.545579000	-1.802374000
H	-2.390561000	5.144540000	-1.320408000
C	-2.070792000	-2.677932000	1.261159000
H	-1.263387000	-2.469050000	1.979215000
H	-2.994389000	-2.225654000	1.637849000
H	-2.209054000	-3.766606000	1.208860000
N	-1.596940000	2.124563000	0.065469000
N	-1.496431000	-0.675586000	-0.059313000
Ni	-2.929419000	0.678565000	-0.058504000
Cl	-4.550740000	-0.887266000	-0.186685000
Cl	-2.926125000	0.638867000	2.219847000
H	-2.518270000	-2.358434000	-0.832460000
H	-0.333192000	-2.757753000	-1.712271000
H	-2.801584000	3.740941000	0.704003000
H	-0.311728000	5.073439000	0.234327000
C	0.550840000	0.794979000	0.118415000
C	1.355119000	0.630259000	1.420259000
H	0.689500000	0.464410000	2.278606000
H	2.039751000	-0.221800000	1.335354000
H	1.950179000	1.531937000	1.606309000
C	1.487155000	1.020804000	-1.081496000
H	2.170833000	0.170617000	-1.187790000
H	0.913502000	1.131749000	-2.012023000
H	2.083995000	1.926870000	-0.925443000
Cl	-4.651684000	2.136393000	-0.075670000
Cl	-2.718232000	0.723889000	-2.329393000

NiBOX-Cl3-F (m1)

C	-0.176780000	-0.492558000	0.027255000
O	0.599910000	-1.557233000	0.072914000
C	-1.675675000	-2.181233000	0.103603000
C	-0.253177000	-2.725699000	-0.038713000
H	0.031327000	-3.422196000	0.758717000
C	-0.340240000	1.951286000	0.323105000
O	0.238555000	3.011483000	0.856943000
C	-1.961401000	3.501997000	0.261912000
C	-0.838602000	3.941428000	1.183713000
H	-1.063540000	3.808804000	2.251164000
C	-1.941924000	4.219892000	-1.072312000
H	-0.922761000	4.236062000	-1.488030000
H	-2.603803000	3.732854000	-1.797027000
H	-2.270398000	5.260586000	-0.945867000
C	-2.354792000	-2.577609000	1.392840000
H	-1.777367000	-2.241296000	2.266354000
H	-3.364937000	-2.158552000	1.457650000
H	-2.431927000	-3.672897000	1.436014000
N	-1.595234000	2.076659000	0.062093000
N	-1.446317000	-0.716431000	0.011592000
Ni	-2.842023000	0.635722000	-0.398056000
Cl	-4.358729000	-0.955697000	-0.897727000
Cl	-3.469711000	0.688382000	1.772845000
H	-2.289512000	-2.480729000	-0.755480000
H	-0.047411000	-3.186883000	-1.012972000
H	-2.941235000	3.560490000	0.751548000
H	-0.475593000	4.958009000	1.001162000
C	0.561843000	0.811097000	-0.030699000
C	1.776742000	0.769487000	0.896146000
H	1.485942000	0.605080000	1.941952000
H	2.456559000	-0.033844000	0.593133000
H	2.322582000	1.717283000	0.833508000
C	0.999033000	1.040342000	-1.494964000
H	1.681913000	0.239895000	-1.806973000
H	0.124892000	1.053931000	-2.157849000
H	1.531047000	1.997447000	-1.576576000
Cl	-4.490748000	2.086902000	-0.924896000
F	-2.213247000	0.654233000	-2.114704000

NiBOX-Cl3-N3 (m1)

C	-0.227999000	-0.483040000	-0.040237000
O	0.536909000	-1.557635000	-0.042049000
C	-1.729280000	-2.166204000	-0.032876000
C	-0.326296000	-2.695639000	-0.304416000
H	-0.022740000	-3.510983000	0.361365000
C	-0.365494000	1.968647000	0.293339000
O	0.246319000	3.044350000	0.757734000
C	-1.963936000	3.540311000	0.211180000
C	-0.800212000	4.009958000	1.064859000
H	-0.989254000	3.947478000	2.145562000
C	-2.001903000	4.200973000	-1.150818000
H	-1.024091000	4.121917000	-1.650689000
H	-2.758968000	3.740871000	-1.794712000
H	-2.240803000	5.267725000	-1.041724000
C	-2.301251000	-2.584139000	1.300351000
H	-1.632547000	-2.302695000	2.127036000
H	-3.278804000	-2.120383000	1.470274000
H	-2.423953000	-3.675847000	1.314130000
N	-1.625868000	2.100091000	0.060156000
N	-1.500737000	-0.694494000	-0.098859000
Ni	-2.921633000	0.669079000	-0.319316000
Cl	-4.460926000	-0.908558000	-0.795456000
Cl	-3.319015000	0.645915000	1.942982000
H	-2.410634000	-2.450772000	-0.844885000
H	-0.160649000	-2.992826000	-1.349101000
H	-2.922407000	3.637494000	0.734743000
H	-0.427398000	5.007190000	0.809553000
C	0.529816000	0.808722000	-0.012548000
C	1.665740000	0.716461000	1.010513000
H	1.283536000	0.539905000	2.024546000
H	2.345708000	-0.100187000	0.745632000
H	2.240215000	1.649060000	1.013941000
C	1.102751000	1.052877000	-1.427141000
H	1.771098000	0.227384000	-1.702882000
H	0.304661000	1.132985000	-2.177646000
H	1.685211000	1.983177000	-1.435379000
Cl	-4.605630000	2.142515000	-0.640419000
N	-2.457845000	0.809963000	-2.250622000
N	-2.157729000	-0.234495000	-2.772298000
N	-1.846197000	-1.199781000	-3.331066000

NiBOX-Cl3-SCN (m1)

C	-0.221655000	-0.502519000	-0.092952000
O	0.543713000	-1.573595000	-0.171107000
C	-1.708741000	-2.194152000	-0.047360000
C	-0.329650000	-2.703922000	-0.439323000
H	0.021054000	-3.550219000	0.161184000
C	-0.344343000	1.962631000	0.217112000
O	0.290870000	3.065587000	0.568380000
C	-1.951138000	3.534093000	0.111348000
C	-0.727570000	4.066586000	0.838871000
H	-0.847187000	4.116884000	1.929653000
C	-2.131456000	4.136377000	-1.265030000
H	-1.227010000	3.997342000	-1.876317000
H	-2.986100000	3.699586000	-1.794429000
H	-2.314309000	5.215562000	-1.170324000
C	-2.140924000	-2.621616000	1.336250000
H	-1.379891000	-2.361955000	2.087258000
H	-3.084903000	-2.147058000	1.625669000
H	-2.277529000	-3.711716000	1.349399000
N	-1.611565000	2.085711000	0.011969000
N	-1.495174000	-0.716797000	-0.116808000
Ni	-2.927589000	0.632334000	-0.180480000
Cl	-4.495197000	-0.957801000	-0.486030000
Cl	-3.042562000	0.624674000	2.110656000
H	-2.457340000	-2.482002000	-0.795483000
H	-0.235685000	-2.941380000	-1.507649000
H	-2.856036000	3.647094000	0.719926000
H	-0.366814000	5.029423000	0.462007000
C	0.546012000	0.774277000	0.041496000
C	1.468856000	0.639553000	1.263745000
H	0.887101000	0.482904000	2.182804000
H	2.150136000	-0.207879000	1.128978000
H	2.069775000	1.548222000	1.379567000
C	1.369718000	1.001044000	-1.240752000
H	2.048566000	0.154848000	-1.401050000
H	0.720975000	1.105387000	-2.121479000
H	1.972194000	1.911761000	-1.138532000
Cl	-4.642985000	2.082783000	-0.348544000
S	-2.749918000	0.862824000	-2.541188000
C	-2.093554000	-0.588781000	-2.987187000
N	-1.621857000	-1.611561000	-3.330952000

NiBOX-Cl3-ONO2 (m1)

C	-0.178982000	-0.438791000	-0.067216000
O	0.587887000	-1.511117000	-0.136389000
C	-1.674928000	-2.125394000	-0.086489000
C	-0.281896000	-2.638591000	-0.425001000
H	0.043713000	-3.482046000	0.193916000
C	-0.318259000	2.009305000	0.302594000
O	0.291129000	3.100757000	0.729246000
C	-1.926256000	3.571309000	0.180102000
C	-0.753209000	4.078221000	1.000491000
H	-0.930698000	4.067766000	2.084609000
C	-2.002128000	4.192604000	-1.198051000
H	-1.033260000	4.111255000	-1.714546000
H	-2.763242000	3.701469000	-1.813199000
H	-2.253891000	5.258580000	-1.114039000
C	-2.192799000	-2.586695000	1.255404000
H	-1.504552000	-2.314410000	2.069277000
H	-3.174754000	-2.150437000	1.469093000
H	-2.293904000	-3.680708000	1.244743000
N	-1.578986000	2.129759000	0.064170000
N	-1.449662000	-0.652906000	-0.110148000
Ni	-2.874689000	0.689753000	-0.304282000
Cl	-4.422429000	-0.871335000	-0.785182000
Cl	-3.223798000	0.614538000	1.934189000
H	-2.384133000	-2.379333000	-0.880927000
H	-0.146599000	-2.888955000	-1.485655000
H	-2.873399000	3.672066000	0.722958000
H	-0.384280000	5.062645000	0.694538000
C	0.583679000	0.843385000	0.055705000
C	1.596002000	0.705238000	1.200286000
H	1.092946000	0.525206000	2.159807000

H	2.278835000	-0.127567000	1.000522000
H	2.190868000	1.621233000	1.284339000
C	1.310450000	1.115069000	-1.278758000
H	1.985761000	0.282566000	-1.510821000
H	0.598886000	1.236884000	-2.107603000
H	1.907804000	2.031594000	-1.196391000
Cl	-4.585358000	2.150569000	-0.520915000
O	-1.860006000	-0.964836000	-2.822522000
N	-2.097999000	0.189940000	-3.109836000
O	-2.528329000	1.041664000	-2.208493000
O	-1.969514000	0.684947000	-4.219323000

NiBOX-Cl3-Me (m1)

C	-0.194307000	-0.444320000	-0.201880000
O	0.553914000	-1.507803000	-0.460094000
C	-1.683195000	-2.113376000	-0.268922000
C	-0.361156000	-2.562986000	-0.861067000
H	0.013830000	-3.510929000	-0.460744000
C	-0.274289000	2.011477000	0.138812000
O	0.404983000	3.132120000	0.332839000
C	-1.854246000	3.593651000	-0.070300000
C	-0.569088000	4.193654000	0.485079000
H	-0.622844000	4.434678000	1.555538000
C	-2.181631000	4.046732000	-1.475913000
H	-1.338184000	3.865747000	-2.159218000
H	-3.068919000	3.533129000	-1.864315000
H	-2.390275000	5.125482000	-1.476110000
C	-1.935839000	-2.662323000	1.118569000
H	-1.049676000	-2.524227000	1.757450000
H	-2.776654000	-2.146608000	1.594905000
H	-2.152968000	-3.738042000	1.064229000
N	-1.543809000	2.139540000	-0.035101000
N	-1.467502000	-0.642328000	-0.192196000
Ni	-2.888957000	0.693851000	-0.033602000
Cl	-4.511865000	-0.871440000	-0.098836000
Cl	-2.603229000	0.631890000	2.368701000
H	-2.523484000	-2.324499000	-0.940486000
H	-0.357795000	-2.597128000	-1.959823000
H	-2.698223000	3.784418000	0.602110000
H	-0.208658000	5.068682000	-0.067264000
C	0.578576000	0.782448000	0.160751000
C	1.080636000	0.571305000	1.605793000
H	0.233135000	0.449341000	2.294495000
H	1.715933000	-0.322180000	1.653964000
H	1.677947000	1.436341000	1.919807000
C	1.759337000	0.974878000	-0.798743000
H	2.413933000	0.096463000	-0.770646000
H	1.417993000	1.120119000	-1.832906000
H	2.349885000	1.848697000	-0.501573000
Cl	-4.590186000	2.177405000	0.053747000
C	-2.946228000	0.684677000	-2.029764000
H	-2.062616000	1.247829000	-2.351114000
H	-2.900270000	-0.367867000	-2.326702000
H	-3.891726000	1.155890000	-2.314068000

NiBOX-Cl3-NO2 (m1)

C	-0.207093000	-0.482759000	-0.059300000
O	0.553019000	-1.562326000	-0.118624000
C	-1.706824000	-2.156243000	-0.012928000
C	-0.332720000	-2.679083000	-0.403197000
H	0.004981000	-3.539537000	0.184339000
C	-0.350772000	1.971200000	0.243174000
O	0.238019000	3.040053000	0.748006000
C	-1.960146000	3.535138000	0.139422000
C	-0.816937000	4.002187000	1.023985000
H	-1.032690000	3.939605000	2.099725000
C	-2.026497000	4.267069000	-1.182775000
H	-1.061684000	4.213504000	-1.710230000
H	-2.800025000	3.844404000	-1.830948000
H	-2.255918000	5.326806000	-1.004727000
C	-2.150442000	-2.565467000	1.373294000
H	-1.379044000	-2.326529000	2.120814000
H	-3.074362000	-2.050296000	1.660502000
H	-2.327111000	-3.649578000	1.398589000
N	-1.594317000	2.105008000	-0.071957000
N	-1.479854000	-0.686438000	-0.079061000
Ni	-2.909650000	0.652159000	-0.270636000
Cl	-4.505154000	-0.917635000	-0.485680000
Cl	-3.135541000	0.797338000	2.053231000
H	-2.456639000	-2.429461000	-0.764212000
H	-0.234703000	-2.903666000	-1.474677000
H	-2.920296000	3.580024000	0.667296000
H	-0.438525000	5.000620000	0.780635000
C	0.555968000	0.801211000	0.023617000
C	1.564456000	0.690945000	1.174184000
H	1.053872000	0.549722000	2.136558000
H	2.237044000	-0.157277000	1.006340000
H	2.170613000	1.601426000	1.231018000
C	1.280302000	1.034456000	-1.317353000
H	1.979761000	0.212105000	-1.511798000
H	0.568017000	1.098807000	-2.151246000
H	1.852219000	1.970063000	-1.274157000
Cl	-4.575779000	2.103292000	-0.699069000
N	-2.519702000	0.518382000	-2.294446000
O	-2.403002000	-0.597468000	-2.733653000
O	-2.388346000	1.557128000	-2.887018000

NiBOX-Cl3-ONO (m1)

C	-0.128214000	-0.444242000	-0.134197000
O	0.627419000	-1.503835000	-0.374870000
C	-1.622043000	-2.104397000	-0.319838000
C	-0.267267000	-2.550815000	-0.838792000
H	0.081509000	-3.504256000	-0.428259000
C	-0.201306000	2.016265000	0.165238000
O	0.488510000	3.134606000	0.310653000
C	-1.756231000	3.605084000	-0.180039000
C	-0.465191000	4.225336000	0.343464000
H	-0.529655000	4.571269000	1.383733000
C	-2.071666000	3.976084000	-1.611988000
H	-1.259757000	3.676085000	-2.291120000
H	-3.007567000	3.516589000	-1.949306000
H	-2.191477000	5.065492000	-1.688044000
C	-1.970298000	-2.695870000	1.028660000
H	-1.136209000	-2.571276000	1.736641000
H	-2.854134000	-2.210648000	1.456141000
H	-2.169553000	-3.771375000	0.924368000

N	-1.465500000	2.151629000	-0.047193000
N	-1.401494000	-0.636851000	-0.191692000
Ni	-2.828073000	0.715882000	-0.083480000
Cl	-4.438476000	-0.847277000	-0.319502000
Cl	-2.721792000	0.594798000	2.259965000
H	-2.415687000	-2.287766000	-1.052855000
H	-0.200212000	-2.572534000	-1.935318000
H	-2.601231000	3.850237000	0.473423000
H	-0.073477000	5.034046000	-0.283961000
C	0.630584000	0.776826000	0.278209000
C	1.014787000	0.582653000	1.760891000
H	0.118597000	0.467885000	2.385313000
H	1.642954000	-0.311050000	1.866555000
H	1.587856000	1.450508000	2.110890000
C	1.889498000	0.938279000	-0.583171000
H	2.530494000	0.055920000	-0.480423000
H	1.638706000	1.064592000	-1.644969000
H	2.460911000	1.813335000	-0.254963000
Cl	-4.512387000	2.211263000	-0.039930000
N	-3.463005000	0.877616000	-2.983520000
O	-2.611874000	0.775374000	-2.080707000
O	-4.638434000	0.976893000	-2.847203000

NiBOX-Cl2 (m3)

C	-0.027652000	-0.498484000	0.148812000
O	0.711966000	-1.591504000	0.324476000
C	-1.553451000	-2.126870000	-0.027391000
C	-0.201919000	-2.715086000	0.383475000
H	-0.196478000	-3.093536000	1.416527000
C	-0.110331000	1.993868000	0.331285000
O	0.541921000	3.092771000	0.703173000
C	-1.778752000	3.476632000	0.522459000
C	-0.429852000	4.170075000	0.737483000
H	-0.331278000	4.682657000	1.700963000
C	-2.643373000	4.070514000	-0.563568000
H	-2.120967000	4.062107000	-1.531056000
H	-3.573035000	3.492119000	-0.668331000
H	-2.915725000	5.107380000	-0.324779000
C	-2.713927000	-2.507014000	0.857289000
H	-2.527381000	-2.214394000	1.901025000
H	-3.632870000	-2.005073000	0.526873000
H	-2.883464000	-3.591867000	0.828908000
N	-1.378764000	2.102553000	0.186301000
N	-1.288206000	-0.679329000	-0.010547000
Ni	-2.658419000	0.712586000	-0.450239000
Cl	-4.326656000	0.815485000	1.082361000
H	-1.791574000	-2.388296000	-1.074347000
H	0.178213000	-3.496428000	-0.283928000
H	-2.355728000	3.426336000	1.461502000
H	-0.173047000	4.873817000	-0.067934000
C	0.749146000	0.790777000	0.038188000
C	1.969755000	0.767512000	0.957442000
H	1.683148000	0.673482000	2.013411000
H	2.621523000	-0.074803000	0.700648000
H	2.546951000	1.691282000	0.838963000
C	1.184828000	0.912358000	-1.439073000
H	1.825533000	0.064424000	-1.714768000
H	0.306332000	0.926406000	-2.100397000
H	1.757801000	1.837594000	-1.585305000
Cl	-2.562930000	0.758893000	-2.724864000

NiBOX-Cl2-OH (m2)

C	-0.045028000	-0.409246000	-0.097230000
O	0.688817000	-1.467890000	-0.426513000
C	-1.610781000	-1.995096000	-0.421640000
C	-0.210501000	-2.605456000	-0.504575000
H	0.019045000	-3.267192000	0.343558000
C	-0.145659000	2.073432000	0.149975000
O	0.514462000	3.230308000	0.149783000
C	-1.816603000	3.560618000	0.050841000
C	-0.479020000	4.283566000	0.241862000
H	-0.368478000	4.757655000	1.226241000
C	-2.499350000	3.829916000	-1.271813000
H	-1.807742000	3.662653000	-2.111145000
H	-3.359834000	3.160312000	-1.396716000
H	-2.854873000	4.868108000	-1.321998000
C	-2.544873000	-2.664434000	0.555138000
H	-2.129613000	-2.651701000	1.572089000
H	-3.516743000	-2.149420000	0.573564000
H	-2.724797000	-3.707374000	0.261867000
N	-1.424092000	2.146089000	0.141682000
N	-1.309331000	-0.610190000	-0.033171000
Ni	-2.745677000	0.624794000	0.387588000
Cl	-4.651276000	1.750657000	0.796437000
Cl	-2.254947000	0.399260000	2.614092000
H	-2.089381000	-1.946239000	-1.413763000
H	-0.002096000	-3.136782000	-1.439449000
H	-2.511647000	3.771850000	0.877793000
H	-0.257508000	5.023676000	-0.537130000
C	0.732052000	0.849629000	0.180175000
C	1.352412000	0.728263000	1.585543000
H	0.573578000	0.636093000	2.354365000
H	2.003641000	-0.154230000	1.632005000
H	1.962494000	1.614788000	1.800644000
C	1.839049000	1.002114000	-0.872001000
H	2.511045000	0.136946000	-0.843151000
H	1.425665000	1.086308000	-1.886267000
H	2.431356000	1.900345000	-0.664414000
O	-3.330476000	0.351791000	-1.339634000
H	-4.177894000	0.821868000	-1.375413000

NiBOX-Cl2-F (m2)

C	-0.069737000	-0.429872000	-0.072933000
O	0.642012000	-1.486133000	-0.442884000
C	-1.655693000	-2.010652000	-0.352309000
C	-0.261948000	-2.625773000	-0.466970000
H	-0.006140000	-3.267054000	0.389078000
C	-0.182171000	2.049239000	0.154595000
O	0.464030000	3.209721000	0.122861000
C	-1.877188000	3.519499000	0.122633000
C	-0.535494000	4.260016000	0.191510000
H	-0.376019000	4.807673000	1.129026000
C	-2.681735000	3.783445000	-1.129740000
H	-2.088465000	3.567021000	-2.030526000
H	-3.583332000	3.157799000	-1.146441000
H	-2.993122000	4.836218000	-1.170167000

C	-2.585384000	-2.684083000	0.623931000
H	-2.151778000	-2.707803000	1.632823000
H	-3.543682000	-2.144764000	0.670797000
H	-2.796654000	-3.713823000	0.307179000
N	-1.462140000	2.108735000	0.187317000
N	-1.329488000	-0.635305000	0.055438000
Ni	-2.747575000	0.576367000	0.520264000
Cl	-4.617652000	1.681492000	1.049325000
Cl	-2.209711000	0.349294000	2.702396000
H	-2.143928000	-1.936329000	-1.338187000
H	-0.084802000	-3.176326000	-1.396488000
H	-2.495138000	3.725242000	1.010876000
H	-0.362876000	4.940153000	-0.652635000
C	0.709201000	0.834054000	0.163861000
C	1.386246000	0.728342000	1.543956000
H	0.641908000	0.628880000	2.345206000
H	2.050410000	-0.145253000	1.567418000
H	1.992570000	1.623749000	1.730166000
C	1.769849000	0.984581000	-0.935195000
H	2.447046000	0.123144000	-0.927260000
H	1.313047000	1.058733000	-1.931265000
H	2.364834000	1.887461000	-0.758389000
F	-3.345731000	0.350051000	-1.187255000

NiBOX-Cl2-N3 (m2)

C	0.104271000	-0.470947000	-0.039096000
O	0.917177000	-1.497060000	-0.256533000
C	-1.338460000	-2.141307000	-0.497594000
C	0.086535000	-2.682825000	-0.385608000
H	0.242139000	-3.294186000	0.515397000
C	-0.153923000	2.006773000	0.146326000
O	0.445389000	3.191293000	0.198639000
C	-1.888219000	3.412390000	-0.078323000
C	-0.592471000	4.202219000	0.146018000
H	-0.565781000	4.755466000	1.093474000
C	-2.564596000	3.683226000	-1.403619000
H	-1.903267000	3.432943000	-2.246560000
H	-3.493143000	3.105634000	-1.496843000
H	-2.826814000	4.747294000	-1.481794000
C	-2.364254000	-2.838021000	0.359654000
H	-2.086975000	-2.801300000	1.421824000
H	-3.351515000	-2.365555000	0.242895000
H	-2.463670000	-3.889472000	0.059315000
N	-1.430168000	2.015600000	0.016784000
N	-1.149214000	-0.734375000	-0.105935000
Ni	-2.672979000	0.426308000	0.223045000
Cl	-4.617294000	1.462404000	0.590510000
Cl	-2.300897000	0.202671000	2.455694000
H	-1.680782000	-2.132799000	-1.547003000
H	0.430792000	-3.240240000	-1.262927000
H	-2.605412000	3.581982000	0.739740000
H	-0.347486000	4.889215000	-0.674201000
C	0.784168000	0.834299000	0.266855000
C	1.315766000	0.775745000	1.711368000
H	0.496130000	0.643245000	2.428872000
H	2.017614000	-0.061449000	1.816956000
H	1.851081000	1.703616000	1.949353000
C	1.951051000	1.030003000	-0.712541000
H	2.663611000	0.202286000	-0.620663000
H	1.603406000	1.074765000	-1.753447000
H	2.479546000	1.962214000	-0.483062000
N	-3.128560000	0.139471000	-1.667309000
N	-4.104348000	0.606922000	-2.191660000
N	-5.020380000	1.020164000	-2.766543000

NiBOX-Cl2-SCN (m2)

C	-0.105300000	-0.529549000	-0.091737000
O	0.625702000	-1.600169000	-0.352582000
C	-1.671071000	-2.152318000	-0.266013000
C	-0.266254000	-2.745495000	-0.400991000
H	0.006312000	-3.411489000	0.429328000
C	-0.204179000	1.964947000	0.064300000
O	0.462400000	3.111951000	0.093872000
C	-1.871969000	3.468418000	0.021018000
C	-0.516204000	4.181341000	0.114452000
H	-0.369833000	4.748231000	1.042049000
C	-2.673483000	3.830777000	-1.207265000
H	-2.115757000	3.592187000	-2.125470000
H	-3.629003000	3.291927000	-1.225201000
H	-2.888430000	4.908311000	-1.208572000
C	-2.525946000	-2.756160000	0.820115000
H	-2.036150000	-2.681919000	1.800255000
H	-3.497896000	-2.243330000	0.879154000
H	-2.719508000	-3.815074000	0.603721000
N	-1.484360000	2.045689000	0.028675000
N	-1.373046000	-0.729659000	-0.012542000
Ni	-2.775727000	0.531064000	0.371222000
Cl	-4.716075000	1.571028000	0.755344000
Cl	-2.313934000	0.361493000	2.579769000
H	-2.209738000	-2.203192000	-1.227952000
H	-0.091271000	-3.262982000	-1.350700000
H	-2.478039000	3.644111000	0.924508000
H	-0.309162000	4.837699000	-0.740981000
C	0.671875000	0.741464000	0.107369000
C	1.334171000	0.669166000	1.498182000
H	0.579972000	0.601990000	2.293624000
H	1.987327000	-0.210787000	1.554690000
H	1.947500000	1.563407000	1.664155000
C	1.744785000	0.856949000	-0.984765000
H	2.423753000	-0.002068000	-0.939498000
H	1.296572000	0.895376000	-1.986644000
H	2.335822000	1.766702000	-0.830624000
S	-3.562308000	0.344639000	-1.826630000
C	-2.135792000	0.086165000	-2.640073000
N	-1.125165000	-0.095066000	-3.215572000

NiBOX-Cl2-ONO2 (m2)

C	-0.033000000	-0.422528000	-0.127333000
O	0.701675000	-1.471618000	-0.462012000
C	-1.589295000	-2.029878000	-0.441328000
C	-0.183113000	-2.623978000	-0.510476000
H	0.055393000	-3.258205000	0.355420000
C	-0.142236000	2.064868000	0.124545000
O	0.518089000	3.218368000	0.121637000
C	-1.810968000	3.559770000	0.035641000
C	-0.472610000	4.271628000	0.251399000

H	-0.361133000	4.706025000	1.253859000
C	-2.464304000	3.839003000	-1.300388000
H	-1.769520000	3.640730000	-2.129766000
H	-3.356429000	3.212233000	-1.430180000
H	-2.776420000	4.890695000	-1.358082000
C	-2.532807000	-2.718945000	0.511259000
H	-2.123235000	-2.737165000	1.530014000
H	-3.504067000	-2.203218000	0.538224000
H	-2.712455000	-3.751929000	0.185852000
N	-1.420793000	2.143114000	0.134304000
N	-1.295685000	-0.640879000	-0.043839000
Ni	-2.710553000	0.612421000	0.301454000
Cl	-4.622710000	1.710329000	0.669107000
Cl	-2.378780000	0.344762000	2.513667000
H	-2.045347000	-1.991033000	-1.446568000
H	0.030082000	-3.174405000	-1.432363000
H	-2.517972000	3.776395000	0.850243000
H	-0.248332000	5.038647000	-0.499337000
C	0.733553000	0.840454000	0.150897000
C	1.332012000	0.712461000	1.566894000
H	0.541528000	0.613832000	2.323029000
H	1.984610000	-0.168469000	1.619562000
H	1.934904000	1.599997000	1.796492000
C	1.853319000	0.995585000	-0.885986000
H	2.527789000	0.132848000	-0.846833000
H	1.453900000	1.078143000	-1.905522000
H	2.439881000	1.895182000	-0.669020000
O	-1.368994000	0.806233000	-2.483874000
N	-2.534370000	0.463819000	-2.593554000
O	-3.313220000	0.401508000	-1.535189000
O	-3.082056000	0.169650000	-3.642953000

NiBOX-Cl2-Me (m2)

C	-0.003172000	-0.453639000	-0.119575000
O	0.764285000	-1.535218000	-0.251687000
C	-1.520000000	-2.075109000	-0.459128000
C	-0.121784000	-2.680198000	-0.316422000
H	0.003337000	-3.250756000	0.615885000
C	-0.126145000	2.046568000	0.093502000
O	0.539774000	3.188044000	0.271658000
C	-1.781826000	3.554224000	0.074171000
C	-0.428724000	4.261540000	0.219400000
H	-0.325340000	4.855015000	1.135666000
C	-2.612152000	4.036118000	-1.091375000
H	-2.083099000	3.885430000	-2.044046000
H	-3.571738000	3.504519000	-1.127832000
H	-2.823078000	5.109406000	-0.986668000
C	-2.559473000	-2.637363000	0.479377000
H	-2.254368000	-2.504349000	1.526210000
H	-3.525395000	-2.127330000	0.342781000
H	-2.715008000	-3.707396000	0.286627000
N	-1.395300000	2.140066000	-0.052892000
N	-1.266289000	-0.649512000	-0.198406000
Ni	-2.719620000	0.623336000	0.112889000
Cl	-4.791406000	1.477682000	0.381456000
Cl	-2.300523000	0.440178000	2.433290000
H	-1.879754000	-2.156866000	-1.500041000
H	0.190051000	-3.304924000	-1.160421000
H	-2.374011000	3.634103000	1.002333000
H	-0.179124000	4.895713000	-0.643214000
C	0.751041000	0.822415000	0.132768000
C	1.369945000	0.729316000	1.541058000
H	0.583149000	0.647349000	2.303455000
H	2.022868000	-0.150102000	1.606413000
H	1.973995000	1.621742000	1.745488000
C	1.856575000	0.965805000	-0.923955000
H	2.542025000	0.111749000	-0.871752000
H	1.441088000	1.014531000	-1.939994000
H	2.434066000	1.879314000	-0.740778000
C	-3.103673000	0.510138000	-1.806311000
H	-2.147598000	0.420106000	-2.338779000
H	-3.713802000	-0.399286000	-1.912062000
H	-3.667208000	1.393274000	-2.124995000

NiBOX-Cl2-NO2 (m2)

C	-0.025961000	-0.444638000	-0.133407000
O	0.743470000	-1.508989000	-0.313749000
C	-1.541488000	-2.084313000	-0.450259000
C	-0.125907000	-2.666160000	-0.415926000
H	0.059485000	-3.297438000	0.464686000
C	-0.156266000	2.051825000	0.112029000
O	0.507139000	3.191239000	0.283372000
C	-1.814722000	3.561136000	0.069561000
C	-0.467285000	4.262147000	0.287856000
H	-0.382218000	4.788661000	1.246493000
C	-2.561358000	4.047371000	-1.150201000
H	-1.956747000	3.918971000	-2.059313000
H	-3.506766000	3.506718000	-1.277632000
H	-2.791067000	5.116630000	-1.041396000
C	-2.499343000	-2.683556000	0.550524000
H	-2.115778000	-2.579748000	1.574522000
H	-3.479984000	-2.188010000	0.498209000
H	-2.652730000	-3.750116000	0.338384000
N	-1.427652000	2.141347000	-0.028800000
N	-1.292593000	-0.653884000	-0.183169000
Ni	-2.729476000	0.612268000	0.241980000
Cl	-4.691383000	1.684532000	0.515829000
Cl	-2.295301000	0.422591000	2.482534000
H	-1.971721000	-2.159856000	-1.463348000
H	0.155785000	-3.219169000	-1.318501000
H	-2.460796000	3.652759000	0.958402000
H	-0.203786000	4.955565000	-0.522235000
C	0.723758000	0.830289000	0.136898000
C	1.350363000	0.717018000	1.540567000
H	0.573832000	0.616027000	2.310598000
H	2.012181000	-0.156797000	1.586402000
H	1.948163000	1.611355000	1.754177000
C	1.826560000	0.995168000	-0.920278000
H	2.511329000	0.139950000	-0.888529000
H	1.408101000	1.069516000	-1.933120000
H	2.405136000	1.903127000	-0.715481000
N	-3.270465000	0.402822000	-1.620081000
O	-3.958688000	-0.588022000	-1.770735000
O	-2.901018000	1.161796000	-2.481130000

CuBOX-Cl2 (m2)

C	-0.086305000	-0.509921000	0.109792000
O	0.693080000	-1.596341000	0.193394000
C	-1.581461000	-2.159286000	-0.017372000
C	-0.184980000	-2.743875000	0.225592000
H	-0.082942000	-3.217414000	1.213593000
C	-0.166275000	2.008043000	0.295409000
O	0.519324000	3.099261000	0.652671000
C	-1.785808000	3.541043000	0.466587000
C	-0.418769000	4.202391000	0.665269000
H	-0.305682000	4.732975000	1.617634000
C	-2.642387000	4.143030000	-0.620902000
H	-2.121259000	4.122987000	-1.589079000
H	-3.578876000	3.575458000	-0.723853000
H	-2.900970000	5.184978000	-0.388330000
C	-2.629469000	-2.584705000	0.982572000
H	-2.328363000	-2.316318000	2.006179000
H	-3.587576000	-2.089880000	0.775971000
H	-2.784857000	-3.671737000	0.945274000
N	-1.428620000	2.154116000	0.148272000
N	-1.346099000	-0.710654000	0.015583000
Cu	-2.822051000	0.750248000	-0.487788000
Cl	-4.164977000	0.777461000	1.317024000
H	-1.933335000	-2.401837000	-1.036402000
H	0.150827000	-3.452675000	-0.540665000
H	-2.358382000	3.520466000	1.410319000
H	-0.145443000	4.883708000	-0.154451000
C	0.686763000	0.787702000	0.044427000
C	1.848715000	0.751351000	1.042681000
H	1.492371000	0.664057000	2.078136000
H	2.501236000	-0.102092000	0.829384000
H	2.448133000	1.664699000	0.961034000
C	1.231419000	0.912528000	-1.394191000
H	1.895669000	0.068411000	-1.620793000
H	0.408525000	0.922346000	-2.122360000
H	1.809382000	1.840414000	-1.498139000
Cl	-2.399220000	0.768011000	-2.707432000

CuBOX-Cl2-OH (m1)

C	-0.102443000	-0.525689000	0.069641000
O	0.677014000	-1.619972000	0.064626000
C	-1.617572000	-2.143258000	0.002092000
C	-0.214476000	-2.755590000	0.122399000
H	-0.049268000	-3.266677000	1.083152000
C	-0.198140000	1.979279000	0.298843000
O	0.477639000	3.063558000	0.667648000
C	-1.829099000	3.533114000	0.517972000
C	-0.454426000	4.175317000	0.704525000
H	-0.325184000	4.690656000	1.662458000
C	-2.690566000	4.152699000	-0.555231000
H	-2.182569000	4.135629000	-1.529418000
H	-3.636095000	3.598199000	-0.649176000
H	-2.933195000	5.193565000	-0.303063000
C	-2.592847000	-2.580100000	1.069947000
H	-2.219031000	-2.321973000	2.072065000
H	-3.564306000	-2.086960000	0.932706000
H	-2.751324000	-3.667007000	1.035031000
N	-1.466767000	2.147170000	0.182628000
N	-1.364883000	-0.700075000	0.054033000
Cu	-2.814717000	0.947604000	-0.553650000
Cl	-4.402196000	-0.223885000	-1.583297000
Cl	-3.950491000	1.082810000	1.399594000
H	-2.052950000	-2.360078000	-0.990072000
H	0.052470000	-3.442594000	-0.689998000
H	-2.390594000	3.494668000	1.466243000
H	-0.183179000	4.861023000	-0.111122000
C	0.664509000	0.772298000	0.024892000
C	1.815227000	0.727063000	1.037498000
H	1.446594000	0.628280000	2.067644000
H	2.464408000	-0.128244000	0.820680000
H	2.421052000	1.637688000	0.973228000
C	1.225172000	0.937219000	-1.403367000
H	1.905150000	0.106458000	-1.632096000
H	0.408146000	0.949980000	-2.136886000
H	1.792977000	1.873984000	-1.481402000
O	-2.002257000	1.209922000	-2.207392000
H	-2.511076000	0.634077000	-2.800776000

CuBOX-Cl3 (m1)

C	-0.119723000	-0.556447000	-0.005683000
O	0.629045000	-1.662562000	-0.080739000
C	-1.681241000	-2.138528000	-0.071803000
C	-0.286523000	-2.781974000	-0.120809000
H	-0.072685000	-3.422396000	0.746737000
C	-0.172707000	1.944181000	0.237077000
O	0.513375000	3.037384000	0.541805000
C	-1.787740000	3.525536000	0.426395000
C	-0.403717000	4.162935000	0.505731000
H	-0.236472000	4.762793000	1.406168000
C	-2.719351000	4.126542000	-0.592975000
H	-2.277599000	4.113515000	-1.598194000
H	-3.665726000	3.566543000	-0.620890000
H	-2.952513000	5.165640000	-0.325074000
C	-2.533628000	-2.585507000	1.093870000
H	-2.025025000	-2.390396000	2.049986000
H	-3.493272000	-2.052705000	1.098175000
H	-2.738933000	-3.663338000	1.032625000
N	-1.441753000	2.122589000	0.119895000
N	-1.387534000	-0.701611000	0.001766000
Cu	-2.842385000	0.901620000	-0.401482000
Cl	-4.566905000	-0.338688000	-1.024962000
Cl	-3.791871000	1.208404000	1.645559000
H	-2.232414000	-2.313161000	-1.011815000
H	-0.084343000	-3.351751000	-1.036387000
H	-2.278587000	3.514105000	1.412791000
H	-0.154333000	4.762229000	-0.381792000
C	0.679403000	0.717855000	0.048774000
C	1.675562000	0.622946000	1.215244000
H	1.157293000	0.523133000	2.178575000
H	2.322005000	-0.251238000	1.077930000
H	2.311452000	1.514483000	1.252016000
C	1.437532000	0.887200000	-1.281703000
H	2.060678000	1.789715000	-1.248829000
H	2.093276000	0.023578000	-1.446937000
H	0.742500000	0.970499000	-2.128076000
Cl	-2.252471000	1.240896000	-2.582392000

CuBOX-Cl2-F (m1)

C	-0.071769000	-0.557155000	0.093132000
O	0.698849000	-1.640407000	0.247668000
C	-1.588126000	-2.180016000	0.023654000
C	-0.193440000	-2.779516000	0.266531000
H	-0.094968000	-3.265892000	1.248038000
C	-0.198236000	1.933589000	0.311512000
O	0.448636000	3.021745000	0.699722000
C	-1.867838000	3.460428000	0.522668000
C	-0.506404000	4.116217000	0.760575000
H	-0.404373000	4.593567000	1.741017000
C	-2.688212000	4.075675000	-0.584201000
H	-2.141984000	4.059766000	-1.537239000
H	-3.631145000	3.524502000	-0.714648000
H	-2.939040000	5.116566000	-0.341031000
C	-2.610769000	-2.524043000	1.082546000
H	-2.288972000	-2.165295000	2.071851000
H	-3.581532000	-2.065933000	0.851963000
H	-2.751470000	-3.612291000	1.143091000
N	-1.471863000	2.080228000	0.193940000
N	-1.327859000	-0.737097000	-0.036199000
Cu	-2.755883000	0.896108000	-0.626414000
Cl	-4.265606000	-0.387935000	-1.619328000
Cl	-4.009690000	0.989256000	1.240526000
H	-1.977879000	-2.484731000	-0.962861000
H	0.137410000	-3.482516000	-0.507949000
H	-2.462794000	3.412742000	1.449274000
H	-0.229511000	4.835695000	-0.022453000
C	0.680523000	0.747041000	0.006936000
C	1.882765000	0.734149000	0.953466000
H	1.576257000	0.621026000	2.001933000
H	2.546433000	-0.098659000	0.696978000
H	2.454493000	1.663679000	0.857749000
C	1.151615000	0.915104000	-1.453978000
H	1.799053000	0.073299000	-1.731899000
H	0.287693000	0.950230000	-2.129326000
H	1.730285000	1.842298000	-1.560275000
F	-1.972005000	1.293309000	-2.246894000

CuBOX-Cl2-N3 (m1)

C	-0.168565000	-0.551521000	-0.049766000
O	0.592884000	-1.650552000	-0.111060000
C	-1.708686000	-2.153269000	-0.080077000
C	-0.311562000	-2.773899000	-0.229152000
H	-0.059847000	-3.495413000	0.559659000
C	-0.241210000	1.955449000	0.201710000
O	0.450104000	3.052765000	0.487965000
C	-1.854534000	3.529377000	0.400894000
C	-0.471995000	4.173579000	0.479670000
H	-0.298907000	4.758480000	1.389091000
C	-2.782741000	4.102930000	-0.639015000
H	-2.336631000	4.059688000	-1.642677000
H	-3.728196000	3.540748000	-0.654133000
H	-3.018238000	5.150251000	-0.408273000
C	-2.461772000	-2.572935000	1.162762000
H	-1.875120000	-2.360321000	2.069208000
H	-3.414303000	-2.030818000	1.234022000
H	-2.679187000	-3.649847000	1.142177000
N	-1.512712000	2.125865000	0.109232000
N	-1.435260000	-0.710027000	-0.055144000
Cu	-2.939708000	0.897720000	-0.413138000
Cl	-4.585295000	-0.428685000	-1.121923000
Cl	-3.902955000	1.200907000	1.610815000
H	-2.330312000	-2.365238000	-0.965652000
H	-0.136542000	-3.243694000	-1.206574000
H	-2.354480000	3.533228000	1.383083000
H	-0.232941000	4.791172000	-0.398105000
C	0.616905000	0.732650000	-0.001281000
C	1.630652000	0.642266000	1.150364000
H	1.127175000	0.533908000	2.120577000
H	2.283448000	-0.225234000	1.001209000
H	2.258850000	1.539300000	1.182098000
C	1.358190000	0.907907000	-1.340724000
H	2.011383000	0.044861000	-1.519251000
H	0.657695000	0.996156000	-2.182519000
H	1.982036000	1.810064000	-1.313106000
N	-2.362542000	1.133139000	-2.315816000
N	-1.993739000	0.112024000	-2.832495000
N	-1.609563000	-0.837542000	-3.375796000

CuBOX-Cl2-SCN (m1)

C	-0.111587000	-0.501062000	-0.136275000
O	0.614164000	-1.605626000	-0.226005000
C	-1.680720000	-2.117537000	-0.191514000
C	-0.305834000	-2.696720000	-0.514328000
H	-0.020318000	-3.552150000	0.108196000
C	-0.147812000	2.003194000	0.076689000
O	0.540870000	3.131731000	0.192357000
C	-1.777260000	3.540475000	0.261293000
C	-0.415562000	4.224951000	0.139681000
H	-0.178979000	4.917753000	0.953923000
C	-2.838161000	4.008972000	-0.701447000
H	-2.488668000	3.956399000	-1.742750000
H	-3.741990000	3.388659000	-0.602249000
H	-3.122803000	5.048257000	-0.490967000
C	-2.262355000	-2.533597000	1.139720000
H	-1.557548000	-2.335200000	1.961378000
H	-3.191062000	-1.980619000	1.337296000
H	-2.493871000	-3.607242000	1.134782000
N	-1.424480000	2.127238000	0.043926000
N	-1.386271000	-0.670499000	-0.176233000
Cu	-2.830241000	0.714795000	-0.074923000
Cl	-4.645756000	-0.520252000	-0.621522000
Cl	-3.368247000	1.042309000	2.171156000
H	-2.397757000	-2.324026000	-0.999452000
H	-0.179864000	-2.954881000	-1.574763000
H	-2.162832000	3.604974000	1.293622000
H	-0.277197000	4.737511000	-0.823595000
C	0.688604000	0.753412000	0.087823000
C	1.289725000	0.638685000	1.506212000
H	0.496762000	0.580072000	2.265157000
H	1.915135000	-0.259377000	1.577439000
H	1.916167000	1.513418000	1.718227000
C	1.802262000	0.856001000	-0.960671000
H	2.453011000	-0.024200000	-0.905342000
H	1.394766000	0.923625000	-1.978436000

H	2.413892000	1.745421000	-0.771035000
S	-2.259439000	0.979992000	-2.710526000
C	-2.466876000	-0.571448000	-3.200043000
N	-2.586223000	-1.689660000	-3.564802000

CuBOX-Cl2-ONO2 (m1)

C	-0.110877000	-0.621075000	-0.166761000
O	0.647513000	-1.712147000	-0.296632000
C	-1.637476000	-2.239099000	-0.154701000
C	-0.263535000	-2.821235000	-0.500224000
H	0.051762000	-3.643781000	0.153799000
C	-0.169236000	1.894597000	-0.070545000
O	0.528483000	3.019589000	-0.052346000
C	-1.766490000	3.492785000	0.104012000
C	-0.411165000	4.120481000	-0.192417000
H	-0.112129000	4.911772000	0.501835000
C	-2.905602000	3.974152000	-0.754364000
H	-2.686575000	3.864711000	-1.825426000
H	-3.824709000	3.416362000	-0.523136000
H	-3.103100000	5.035049000	-0.552351000
C	-2.179903000	-2.678730000	1.188846000
H	-1.442283000	-2.496994000	1.986019000
H	-3.094228000	-2.123328000	1.436268000
H	-2.415648000	-3.751948000	1.182863000
N	-1.447855000	2.060759000	-0.081250000
N	-1.376612000	-0.791942000	-0.132041000
Cu	-2.855142000	0.768444000	-0.217559000
Cl	-4.636981000	-0.517059000	-0.470331000
Cl	-3.331416000	1.153642000	1.974343000
H	-2.373824000	-2.454141000	-0.944100000
H	-0.167930000	-3.140307000	-1.547225000
H	-2.033317000	3.607638000	1.169177000
H	-0.333633000	4.486981000	-1.227026000
C	0.665926000	0.649055000	0.049774000
C	1.149834000	0.616256000	1.519381000
H	0.299531000	0.596068000	2.215822000
H	1.762520000	-0.278556000	1.685488000
H	1.764212000	1.498623000	1.737454000
C	1.865945000	0.721757000	-0.898221000
H	2.512423000	-0.149310000	-0.743409000
H	1.551479000	0.739057000	-1.949002000
H	2.458170000	1.621162000	-0.695319000
O	-0.871957000	0.849869000	-2.652796000
N	-1.966621000	1.271670000	-2.994410000
O	-2.991848000	1.165987000	-2.206632000
O	-2.194515000	1.797859000	-4.075057000

CuBOX-Cl2-Me (m1)

C	-0.081801000	-0.536758000	-0.116321000
O	0.700866000	-1.626409000	-0.168459000
C	-1.589405000	-2.162594000	-0.274887000
C	-0.176156000	-2.757368000	-0.374122000
H	0.044570000	-3.504207000	0.400362000
C	-0.165140000	1.987993000	0.063901000
O	0.536184000	3.110051000	0.257787000
C	-1.760815000	3.557996000	0.184002000
C	-0.387223000	4.216461000	0.120249000
H	-0.185566000	4.932229000	0.924313000
C	-2.789246000	4.090475000	-0.779208000
H	-2.440305000	4.020779000	-1.819744000
H	-3.728370000	3.525124000	-0.687540000
H	-3.011130000	5.144117000	-0.562793000
C	-2.386165000	-2.619812000	0.927738000
H	-1.834996000	-2.423434000	1.859404000
H	-3.339936000	-2.077267000	0.977907000
H	-2.595594000	-3.697166000	0.871901000
N	-1.431412000	2.143674000	-0.060877000
N	-1.340821000	-0.720113000	-0.198213000
Cu	-2.914348000	0.819879000	-0.150731000
Cl	-4.948665000	-0.044186000	-0.487751000
Cl	-3.049254000	0.927810000	2.166729000
H	-2.169347000	-2.366634000	-1.191032000
H	0.056973000	-3.189911000	-1.356566000
H	-2.168043000	3.594017000	1.210895000
H	-0.190627000	4.699446000	-0.849712000
C	0.686240000	0.743504000	0.089932000
C	1.328447000	0.667281000	1.490631000
H	0.559109000	0.609838000	2.273391000
H	1.964492000	-0.223461000	1.560988000
H	1.950168000	1.551456000	1.675329000
C	1.775867000	0.862786000	-0.985348000
H	2.445339000	-0.004214000	-0.941409000
H	1.342719000	0.913570000	-1.994082000
H	2.374303000	1.766551000	-0.820681000
C	-2.794130000	0.872919000	-2.137443000
H	-1.751929000	1.137560000	-2.343053000
H	-3.065094000	-0.131990000	-2.472528000
H	-3.514081000	1.635173000	-2.454276000

CuBOX-Cl2-NO2 (m1)

C	-0.107882000	-0.548076000	-0.024721000
O	0.660478000	-1.639953000	-0.139933000
C	-1.638538000	-2.156640000	-0.076055000
C	-0.234099000	-2.776428000	-0.137481000
H	0.006032000	-3.392480000	0.741355000
C	-0.194275000	1.967652000	0.155113000
O	0.484077000	3.063773000	0.477661000
C	-1.805280000	3.534743000	0.313780000
C	-0.430225000	4.185288000	0.388174000
H	-0.273179000	4.822482000	1.264556000
C	-2.766636000	4.162454000	-0.659891000
H	-2.350786000	4.195769000	-1.676451000
H	-3.711236000	3.600747000	-0.686460000
H	-2.997836000	5.190637000	-0.350838000
C	-2.487232000	-2.636135000	1.079636000
H	-1.984781000	-2.448621000	2.040668000
H	-3.453022000	-2.113917000	1.089652000
H	-2.678927000	-3.715395000	1.001320000
N	-1.457398000	2.139392000	-0.031273000
N	-1.370472000	-0.718347000	0.021924000
Cu	-2.909968000	0.825137000	-0.277678000
Cl	-4.639628000	-0.400624000	-0.999577000
Cl	-3.630873000	1.099338000	1.859757000
H	-2.186327000	-2.321119000	-1.020968000
H	-0.037952000	-3.364886000	-1.042284000
H	-2.275750000	3.485844000	1.310746000

H	-0.174212000	4.749922000	-0.521105000
C	0.672436000	0.738018000	0.041029000
C	1.593212000	0.672676000	1.272233000
H	1.013223000	0.612733000	2.203293000
H	2.233717000	-0.214363000	1.206109000
H	2.237688000	1.557827000	1.319002000
C	1.521245000	0.877564000	-1.235359000
H	2.217781000	0.033701000	-1.310358000
H	0.897670000	0.893453000	-2.139176000
H	2.107863000	1.803935000	-1.200898000
N	-2.525595000	0.936315000	-2.271751000
O	-1.773389000	0.111360000	-2.717697000
O	-3.074767000	1.843833000	-2.840530000

CuBOX-Cl (m1)

C	-0.084748000	-0.511835000	0.029715000
O	0.636344000	-1.614890000	0.290171000
C	-1.605049000	-2.118664000	-0.203743000
C	-0.223889000	-2.744640000	0.026142000
H	-0.176306000	-3.420351000	0.890513000
C	-0.145067000	2.001438000	0.223506000
O	0.566214000	3.138862000	0.273776000
C	-1.742020000	3.540397000	0.464186000
C	-0.380984000	4.228051000	0.313211000
H	-0.111160000	4.886742000	1.147381000
C	-2.790406000	3.969569000	-0.533361000
H	-2.422231000	3.852960000	-1.563834000
H	-3.691429000	3.347701000	-0.422657000
H	-3.071360000	5.022006000	-0.387644000
C	-2.608614000	-2.370351000	0.900790000
H	-2.192883000	-2.084286000	1.879169000
H	-3.514658000	-1.770433000	0.726385000
H	-2.893007000	-3.430916000	0.946787000
N	-1.420123000	2.110504000	0.307133000
N	-1.312873000	-0.678421000	-0.293983000
Cu	-2.825919000	0.750962000	-0.131919000
Cl	-4.997174000	0.463390000	-0.451037000
H	-2.030156000	-2.449130000	-1.164958000
H	0.172993000	-3.271378000	-0.853112000
H	-2.137759000	3.676335000	1.485648000
H	-0.284589000	4.793928000	-0.626150000
C	0.712122000	0.767889000	0.078527000
C	1.698764000	0.724387000	1.254824000
H	1.175729000	0.615886000	2.215035000
H	2.390592000	-0.118810000	1.144387000
H	2.288680000	1.648040000	1.286862000
C	1.488237000	0.869405000	-1.248983000
H	2.115236000	-0.021151000	-1.387036000
H	0.803531000	0.946341000	-2.104938000
H	2.141974000	1.750617000	-1.245480000

CuBOX-Cl -OH (m2)

C	-0.111751000	-0.578563000	0.088483000
O	0.627720000	-1.610611000	0.485475000
C	-1.646303000	-2.187450000	0.260901000
C	-0.243168000	-2.772989000	0.456538000
H	-0.110304000	-3.316007000	1.400362000
C	-0.287918000	1.865963000	0.249649000
O	0.214274000	2.813317000	1.033971000
C	-2.036733000	3.247315000	0.442149000
C	-0.825818000	3.813641000	1.200556000
H	-0.989748000	3.947944000	2.276287000
C	-2.606509000	4.160694000	-0.616840000
H	-1.822832000	4.481117000	-1.319847000
H	-3.384748000	3.639957000	-1.190966000
H	-3.046175000	5.060084000	-0.164353000
C	-2.517254000	-2.232202000	1.497475000
H	-2.009360000	-1.765200000	2.354878000
H	-3.459487000	-1.700217000	1.310979000
H	-2.754172000	-3.271100000	1.766420000
N	-1.498997000	2.009919000	-0.141618000
N	-1.359638000	-0.794782000	-0.115632000
Cu	-2.562921000	0.548246000	-1.128343000
Cl	-4.410399000	-0.842764000	-1.213494000
H	-2.175767000	-2.665148000	-0.576756000
H	0.091291000	-3.412870000	-0.371426000
H	-2.838775000	2.962514000	1.144113000
H	-0.449873000	4.756628000	0.778664000
C	0.616008000	0.725881000	-0.154868000
C	1.940759000	0.769740000	0.595727000
H	1.802419000	0.664840000	1.679402000
H	2.597197000	-0.039385000	0.255486000
H	2.451017000	1.722158000	0.408573000
C	0.842792000	0.836412000	-1.675556000
H	1.449242000	-0.007366000	-2.031385000
H	-0.116843000	0.836416000	-2.214631000
H	1.375016000	1.768429000	-1.910276000
O	-2.912416000	1.592253000	-2.665351000
H	-2.301011000	2.337096000	-2.601358000

CuBOX-Cl -F (m2)

C	-0.111366000	-0.502488000	-0.041324000
O	0.663949000	-1.585291000	-0.054909000
C	-1.618738000	-2.144919000	-0.231030000
C	-0.200866000	-2.722785000	-0.301618000
H	0.011313000	-3.478988000	0.464935000
C	-0.269456000	1.979401000	0.272591000
O	0.388918000	3.127769000	0.424813000
C	-1.925672000	3.479357000	0.326136000
C	-0.602524000	4.151881000	0.683399000
H	-0.521295000	4.430024000	1.743139000
C	-2.506099000	3.903610000	-1.005033000
H	-1.769075000	3.787788000	-1.814497000
H	-3.388735000	3.294855000	-1.244053000
H	-2.816025000	4.957405000	-0.975442000
C	-2.427773000	-2.593231000	0.965437000
H	-1.898555000	-2.377609000	1.905980000
H	-3.392165000	-2.069338000	0.967485000
H	-2.619176000	-3.674395000	0.923173000
N	-1.552218000	2.050038000	0.271146000
N	-1.371689000	-0.695001000	-0.167888000
Cu	-2.931458000	0.608091000	-0.077995000
Cl	-4.695316000	1.797967000	0.818312000
H	-2.186116000	-2.349180000	-1.150145000
H	0.063652000	-3.131723000	-1.285780000
H	-2.679166000	3.604577000	1.115826000

H	-0.359357000	5.024463000	0.064877000
C	0.637850000	0.793323000	0.085369000
C	1.604291000	0.706185000	1.275971000
H	1.065552000	0.550880000	2.220603000
H	2.303889000	-0.125999000	1.134887000
H	2.185174000	1.632396000	1.356893000
C	1.427288000	1.009269000	-1.220214000
H	2.110094000	0.167711000	-1.390071000
H	0.753798000	1.091419000	-2.084496000
H	2.022264000	1.928129000	-1.153168000
F	-4.032815000	-0.673689000	-0.866830000

CuBOX-Cl -N3 (m2)

C	0.013943000	-0.506846000	-0.049363000
O	0.773927000	-1.586396000	0.119453000
C	-1.447027000	-2.161459000	-0.430512000
C	-0.047906000	-2.740961000	-0.188421000
H	0.006335000	-3.428959000	0.665316000
C	-0.210903000	1.970549000	0.281998000
O	0.400435000	3.121373000	0.552361000
C	-1.922617000	3.416483000	0.298262000
C	-0.628439000	4.131121000	0.701038000
H	-0.612761000	4.468127000	1.745500000
C	-2.533073000	3.899760000	-0.998377000
H	-1.806332000	3.839231000	-1.822589000
H	-3.409991000	3.289430000	-1.256866000
H	-2.860500000	4.944704000	-0.907924000
C	-2.484996000	-2.547794000	0.599708000
H	-2.141976000	-2.301811000	1.615864000
H	-3.428260000	-2.010428000	0.419472000
H	-2.694132000	-3.625536000	0.560965000
N	-1.488198000	2.014180000	0.162521000
N	-1.204333000	-0.711267000	-0.390405000
Cu	-2.760038000	0.577306000	-0.474842000
Cl	-4.658954000	1.383901000	0.523659000
H	-1.815549000	-2.416076000	-1.436389000
H	0.387389000	-3.232520000	-1.067877000
H	-2.676817000	3.461472000	1.098753000
H	-0.367301000	4.976209000	0.050941000
C	0.731771000	0.805104000	0.119443000
C	1.663610000	0.735302000	1.336293000
H	1.102202000	0.565801000	2.265105000
H	2.385504000	-0.080436000	1.215219000
H	2.222743000	1.672548000	1.436853000
C	1.554356000	1.043606000	-1.162644000
H	2.256813000	0.214675000	-1.317094000
H	0.904336000	1.119148000	-2.045064000
H	2.132450000	1.971583000	-1.071980000
N	-3.666184000	-0.449127000	-1.922423000
N	-4.821195000	-0.762630000	-1.901022000
N	-5.936582000	-1.092876000	-1.923849000

CuBOX-Cl -SCN (m2)

C	-0.312776000	-0.589236000	0.005490000
O	0.417957000	-1.697778000	-0.087653000
C	-1.867025000	-2.181936000	0.225436000
C	-0.503895000	-2.816546000	-0.074128000
H	-0.164525000	-3.521226000	0.696187000
C	-0.343886000	1.909605000	0.202015000
O	0.353120000	2.989949000	0.534985000
C	-1.956432000	3.457383000	0.442644000
C	-0.576023000	4.100851000	0.602758000
H	-0.423439000	4.610783000	1.560393000
C	-2.834985000	4.099682000	-0.602592000
H	-2.330004000	4.138982000	-1.578540000
H	-3.777517000	3.545661000	-0.716690000
H	-3.085771000	5.127974000	-0.308695000
C	-2.453694000	-2.542063000	1.571213000
H	-1.748767000	-2.317165000	2.385511000
H	-3.380544000	-1.977962000	1.741197000
H	-2.692586000	-3.613670000	1.614100000
N	-1.615379000	2.063859000	0.104448000
N	-1.576786000	-0.740233000	0.156384000
Cu	-2.980822000	0.701387000	-0.330088000
Cl	-4.812469000	-0.585662000	-0.374077000
H	-2.595751000	-2.414124000	-0.566721000
H	-0.447152000	-3.304355000	-1.056073000
H	-2.492693000	3.436476000	1.406484000
H	-0.324221000	4.792656000	-0.214050000
C	0.482166000	0.687495000	-0.104395000
C	1.683820000	0.609138000	0.844350000
H	1.367222000	0.500600000	1.890540000
H	2.311807000	-0.249772000	0.582530000
H	2.294822000	1.514714000	0.760061000
C	0.964495000	0.837480000	-1.561667000
H	1.539881000	-0.046979000	-1.863610000
H	0.115908000	0.959172000	-2.248260000
H	1.613270000	1.718658000	-1.650166000
S	-2.920328000	1.169200000	-2.824417000
C	-2.198362000	-0.310920000	-2.953049000
N	-1.659884000	-1.359585000	-3.040055000

CuBOX-Cl -ONO2 (m2)

C	-0.187045000	-0.514182000	-0.122307000
O	0.572486000	-1.605170000	-0.138757000
C	-1.713850000	-2.141624000	-0.325429000
C	-0.301707000	-2.734805000	-0.382961000
H	-0.103127000	-3.487733000	0.390307000
C	-0.290632000	1.971405000	0.226028000
O	0.386808000	3.101587000	0.407484000
C	-1.921869000	3.503472000	0.302451000
C	-0.587584000	4.139448000	0.685691000
H	-0.508324000	4.386360000	1.753110000
C	-2.482466000	3.961634000	-1.026091000
H	-1.745910000	3.827283000	-1.833460000
H	-3.382962000	3.384834000	-1.277902000
H	-2.754576000	5.025307000	-0.985097000
C	-2.541211000	-2.590777000	0.857642000
H	-2.008956000	-2.408364000	1.802957000
H	-3.497915000	-2.051593000	0.890945000
H	-2.759961000	-3.665197000	0.791791000
N	-1.571505000	2.070655000	0.223087000
N	-1.451113000	-0.692033000	-0.251856000
Cu	-2.945750000	0.669632000	-0.164832000
Cl	-4.735516000	1.795706000	0.681334000
H	-2.262996000	-2.346578000	-1.257246000

H	-0.036126000	-3.152468000	-1.362647000
H	-2.677801000	3.628764000	1.089820000
H	-0.321084000	5.022324000	0.092338000
C	0.585791000	0.770484000	-0.005253000
C	1.600244000	0.654537000	1.140611000
H	1.099615000	0.495471000	2.105273000
H	2.280474000	-0.185612000	0.960272000
H	2.198418000	1.570401000	1.207738000
C	1.315580000	0.988638000	-1.346317000
H	1.977967000	0.139823000	-1.556054000
H	0.599650000	1.087530000	-2.174465000
H	1.925572000	1.898917000	-1.300362000
O	-3.105196000	0.687483000	-2.583869000
N	-3.864011000	-0.287327000	-2.427711000
O	-4.138274000	-0.629383000	-1.214890000
O	-4.347381000	-0.923156000	-3.347508000

CuBOX-Cl -Me (m2)

C	-0.078908000	-0.478458000	0.000964000
O	0.646734000	-1.562965000	0.280529000
C	-1.587645000	-2.106982000	-0.242868000
C	-0.214958000	-2.711099000	0.085295000
H	-0.201718000	-3.301908000	1.011202000
C	-0.228586000	1.992671000	0.346892000
O	0.413200000	3.095327000	0.742205000
C	-1.891161000	3.472703000	0.465092000
C	-0.598375000	4.099760000	0.996484000
H	-0.610228000	4.292493000	2.077944000
C	-2.415526000	4.098690000	-0.809138000
H	-1.648139000	4.089378000	-1.598404000
H	-3.293969000	3.541449000	-1.163802000
H	-2.716115000	5.141833000	-0.637434000
C	-2.659465000	-2.374065000	0.790953000
H	-2.326927000	-2.059359000	1.791526000
H	-3.577183000	-1.818379000	0.550038000
H	-2.908012000	-3.443345000	0.833235000
N	-1.499678000	2.076436000	0.209410000
N	-1.301922000	-0.668046000	-0.328508000
Cu	-2.806066000	0.715063000	-0.656671000
Cl	-4.734595000	1.571976000	0.381562000
H	-1.941142000	-2.445241000	-1.230740000
H	0.213432000	-3.315377000	-0.724778000
H	-2.688158000	3.473971000	1.223674000
H	-0.299955000	5.019791000	0.477159000
C	0.675387000	0.827202000	0.022613000
C	1.836205000	0.762288000	1.016479000
H	1.483593000	0.592964000	2.042871000
H	2.519067000	-0.052168000	0.750258000
H	2.402231000	1.700207000	0.998855000
C	1.213292000	1.061121000	-1.405236000
H	1.879914000	0.237972000	-1.695234000
H	0.392074000	1.118620000	-2.133066000
H	1.786840000	1.996517000	-1.446341000
C	-3.726572000	-0.215138000	-2.163891000
H	-2.992425000	-0.556091000	-2.915048000
H	-4.291490000	-1.087628000	-1.795761000
H	-4.437263000	0.483579000	-2.635329000

CuBOX-Cl -NO2 (m2)

C	-0.091553000	-0.471456000	-0.068126000
O	0.649925000	-1.570278000	0.054111000
C	-1.613191000	-2.093290000	-0.338241000
C	-0.214904000	-2.705177000	-0.202155000
H	-0.113033000	-3.402760000	0.639333000
C	-0.208094000	2.016563000	0.267159000
O	0.455030000	3.170269000	0.360071000
C	-1.871496000	3.498723000	0.430686000
C	-0.526777000	4.197422000	0.647693000
H	-0.363736000	4.531897000	1.681206000
C	-2.593768000	3.899542000	-0.838245000
H	-1.934158000	3.795032000	-1.713648000
H	-3.475305000	3.259814000	-0.990452000
H	-2.929290000	4.944471000	-0.786286000
C	-2.579886000	-2.429578000	0.775314000
H	-2.140395000	-2.207923000	1.759563000
H	-3.498722000	-1.835359000	0.661594000
H	-2.855288000	-3.492872000	0.753493000
N	-1.485412000	2.079088000	0.351252000
N	-1.335626000	-0.646612000	-0.330558000
Cu	-2.862501000	0.658281000	-0.155206000
Cl	-4.715613000	1.461603000	0.912913000
H	-2.067915000	-2.351732000	-1.306809000
H	0.144749000	-3.195922000	-1.115691000
H	-2.541181000	3.627297000	1.294684000
H	-0.344645000	5.039110000	-0.031808000
C	0.679632000	0.816285000	0.063143000
C	1.649969000	0.719886000	1.249493000
H	1.114600000	0.558821000	2.195076000
H	2.351409000	-0.109640000	1.103165000
H	2.228697000	1.647296000	1.333582000
C	1.466216000	1.014251000	-1.246679000
H	2.121712000	0.152584000	-1.425175000
H	0.790136000	1.121219000	-2.106092000
H	2.091052000	1.913197000	-1.179924000
N	-3.997265000	-0.132202000	-1.657768000
O	-4.452801000	-1.268070000	-1.700145000
O	-4.195349000	0.697766000	-2.543311000

CuBOX-Cl -ONO (m2)

C	-0.099264000	-0.415106000	-0.242428000
O	0.645029000	-1.517432000	-0.293947000
C	-1.623404000	-1.999210000	-0.643779000
C	-0.216872000	-2.586076000	-0.760842000
H	-0.041646000	-3.463510000	-0.127175000
C	-0.197981000	2.050221000	0.220968000
O	0.485601000	3.174035000	0.421757000
C	-1.827725000	3.587934000	0.259094000
C	-0.478155000	4.246959000	0.559832000
H	-0.392777000	4.641178000	1.580647000
C	-2.491330000	4.059375000	-1.014859000
H	-1.828393000	3.917758000	-1.881671000
H	-3.422652000	3.501960000	-1.188071000
H	-2.741715000	5.127134000	-0.948065000
C	-2.406608000	-2.494289000	0.553304000
H	-1.825297000	-2.370599000	1.479531000
H	-3.348249000	-1.936891000	0.662925000

H	-2.653316000	-3.559340000	0.444335000
N	-1.475063000	2.159914000	0.156047000
N	-1.350389000	-0.557111000	-0.491067000
Cu	-2.866265000	0.768577000	-0.290332000
Cl	-4.577801000	1.771433000	0.845179000
H	-2.200898000	-2.156832000	-1.566438000
H	0.078337000	-2.822414000	-1.792164000
H	-2.531022000	3.695285000	1.098014000
H	-0.204091000	5.037628000	-0.150632000
C	0.668645000	0.823564000	0.126464000
C	1.317117000	0.594300000	1.504210000
H	0.559829000	0.394478000	2.274668000
H	2.005743000	-0.257973000	1.461711000
H	1.888741000	1.482301000	1.800507000
C	1.755557000	1.058379000	-0.934724000
H	2.402516000	0.175961000	-1.005196000
H	1.316599000	1.247600000	-1.923760000
H	2.377215000	1.917957000	-0.658977000
N	-4.914444000	-1.039998000	-1.065921000
O	-3.974303000	-0.279936000	-1.540909000
O	-5.514284000	-1.675201000	-1.909664000

ZnBOX-Cl (m2)

C	0.004019000	-0.538608000	0.104111000
O	0.700690000	-1.579744000	0.580732000
C	-1.479660000	-2.191341000	-0.087564000
C	-0.197395000	-2.712862000	0.578925000
H	-0.351393000	-3.022618000	1.623590000
C	-0.186364000	1.904099000	0.280280000
O	0.264799000	2.883040000	1.075458000
C	-1.946138000	3.266549000	0.338373000
C	-0.772787000	3.892992000	1.107337000
H	-0.990169000	4.124052000	2.157364000
C	-2.526357000	4.132061000	-0.754706000
H	-1.757100000	4.409472000	-1.490699000
H	-3.328088000	3.590278000	-1.276679000
H	-2.954350000	5.056928000	-0.342696000
C	-2.737782000	-2.383490000	0.726759000
H	-2.631025000	-1.928531000	1.722982000
H	-3.592023000	-1.901258000	0.232308000
H	-2.962941000	-3.450774000	0.860605000
N	-1.366198000	2.033410000	-0.198536000
N	-1.195267000	-0.766055000	-0.297116000
Zn	-2.358861000	0.619357000	-1.497448000
Cl	-4.448395000	0.824658000	-0.361867000
H	-1.617052000	-2.656226000	-1.079339000
H	0.293006000	-3.532889000	0.039703000
H	-2.759623000	2.969301000	1.023142000
H	-0.374786000	4.798114000	0.623447000
C	0.754707000	0.766314000	-0.033125000
C	1.987128000	0.799130000	0.861377000
H	1.727021000	0.700868000	1.923572000
H	2.667977000	-0.019582000	0.600669000
H	2.524527000	1.746137000	0.730122000
C	1.153046000	0.894399000	-1.517505000
H	1.823059000	0.072785000	-1.805220000
H	0.251923000	0.858352000	-2.157367000
H	1.672858000	1.845928000	-1.694294000

ZnBOX-Cl -OH (m1)

C	-0.075320000	-0.549687000	0.115197000
O	0.677842000	-1.540161000	0.585778000
C	-1.508607000	-2.262740000	0.081250000
C	-0.186094000	-2.696079000	0.725032000
H	-0.279495000	-2.911568000	1.799796000
C	-0.258650000	1.912885000	0.328231000
O	0.344496000	2.966347000	0.872301000
C	-1.977689000	3.311329000	0.589105000
C	-0.685397000	3.963119000	1.106426000
H	-0.694403000	4.185275000	2.180568000
C	-2.652689000	4.040703000	-0.550965000
H	-1.962935000	4.172157000	-1.398369000
H	-3.525143000	3.470153000	-0.900653000
H	-3.001673000	5.033393000	-0.235071000
C	-2.734548000	-2.508931000	0.926156000
H	-2.647984000	-2.005238000	1.900368000
H	-3.633650000	-2.121659000	0.428637000
H	-2.874153000	-3.583646000	1.106061000
N	-1.522550000	1.990108000	0.146085000
N	-1.293329000	-0.829657000	-0.174391000
Zn	-2.690866000	0.570788000	-0.885246000
Cl	-4.665807000	0.628701000	0.262099000
H	-1.640456000	-2.751951000	-0.899633000
H	0.304680000	-3.544812000	0.235101000
H	-2.703053000	3.165812000	1.406352000
H	-0.399258000	4.872377000	0.559865000
C	0.624182000	0.772007000	-0.120599000
C	1.979031000	0.815006000	0.575358000
H	1.887255000	0.708052000	1.663961000
H	2.622110000	0.008061000	0.205692000
H	2.478970000	1.768329000	0.368021000
C	0.788012000	0.925641000	-1.648807000
H	1.412956000	0.111554000	-2.041714000
H	-0.192878000	0.902803000	-2.153080000
H	1.286679000	1.878533000	-1.875225000
O	-2.511791000	0.774672000	-2.759174000
H	-2.558980000	1.722236000	-2.931124000

ZnBOX-Cl2 (m1)

C	0.004019000	-0.538608000	0.104111000
O	0.700690000	-1.579744000	0.580732000
C	-1.479660000	-2.191341000	-0.087564000
C	-0.197395000	-2.712862000	0.578925000
H	-0.351393000	-3.022618000	1.623590000
C	-0.186364000	1.904099000	0.280280000
O	0.264799000	2.883040000	1.075458000
C	-1.946138000	3.266549000	0.338373000
C	-0.772787000	3.892992000	1.107337000
H	-0.990169000	4.124052000	2.157364000
C	-2.526357000	4.132061000	-0.754706000
H	-1.757100000	4.409472000	-1.490699000
H	-3.328088000	3.590278000	-1.276679000
H	-2.954350000	5.056928000	-0.342696000
C	-2.737782000	-2.383490000	0.726759000
H	-2.631025000	-1.928531000	1.722982000
H	-3.592023000	-1.901258000	0.232308000

H	-2.962941000	-3.450774000	0.860605000
N	-1.366198000	2.033410000	-0.198536000
N	-1.195267000	-0.766055000	-0.297116000
Zn	-2.358861000	0.619357000	-1.497448000
Cl	-4.448395000	0.824658000	-0.361867000
H	-1.617052000	-2.656226000	-1.079339000
H	0.293006000	-3.532889000	0.039703000
H	-2.759623000	2.969301000	1.023142000
H	-0.374786000	4.798114000	0.623447000
C	0.754707000	0.766314000	-0.033125000
C	1.987128000	0.799130000	0.861377000
H	1.727021000	0.700868000	1.923572000
H	2.667977000	-0.019582000	0.600669000
H	2.524527000	1.746137000	0.730122000
C	1.153046000	0.894399000	-1.517505000
H	1.823059000	0.072785000	-1.805220000
H	0.251923000	0.858352000	-2.157367000
H	1.672858000	1.845928000	-1.694294000

ZnBOX-Cl -F (m1)

C	-0.096151000	-0.502469000	0.029625000
O	0.679828000	-1.587704000	0.004665000
C	-1.592598000	-2.141696000	-0.233243000
C	-0.199450000	-2.738459000	-0.021028000
H	-0.096077000	-3.259158000	0.942714000
C	-0.207092000	2.012002000	0.234565000
O	0.471500000	3.151220000	0.376826000
C	-1.854730000	3.524329000	0.297496000
C	-0.496484000	4.228820000	0.409233000
H	-0.352589000	4.780816000	1.345878000
C	-2.697958000	3.928995000	-0.890026000
H	-2.132137000	3.822984000	-1.827412000
H	-3.590246000	3.290017000	-0.962026000
H	-3.029412000	4.972590000	-0.804216000
C	-2.661899000	-2.646521000	0.703720000
H	-2.384965000	-2.466990000	1.752727000
H	-3.613766000	-2.130678000	0.514938000
H	-2.823346000	-3.724417000	0.567796000
N	-1.483242000	2.106798000	0.193923000
N	-1.358468000	-0.699881000	-0.062109000
Zn	-2.936174000	0.642912000	-0.091050000
Cl	-4.230670000	0.628892000	1.778947000
H	-1.928943000	-2.285317000	-1.275342000
H	0.136366000	-3.406182000	-0.822341000
H	-2.442189000	3.650663000	1.222507000
H	-0.278653000	4.899734000	-0.433597000
C	0.671606000	0.792536000	0.111915000
C	1.621682000	0.737668000	1.317854000
H	1.068620000	0.628313000	2.260676000
H	2.308641000	-0.110953000	1.218440000
H	2.217703000	1.656308000	1.370599000
C	1.483223000	0.931025000	-1.190643000
H	2.156235000	0.072952000	-1.307788000
H	0.823944000	0.979381000	-2.068186000
H	2.090839000	1.843600000	-1.161018000
F	-3.812069000	0.514606000	-1.720771000

ZnBOX-Cl -N3 (m1)

C	-0.093315000	-0.525385000	0.025908000
O	0.677787000	-1.611518000	0.073198000
C	-1.571282000	-2.175528000	-0.274931000
C	-0.209882000	-2.757510000	0.099086000
H	-0.186260000	-3.172977000	1.118253000
C	-0.198975000	1.996641000	0.184533000
O	0.487214000	3.132464000	0.307307000
C	-1.829839000	3.518436000	0.359619000
C	-0.472613000	4.216720000	0.261110000
H	-0.250980000	4.901483000	1.086955000
C	-2.865457000	3.966093000	-0.642177000
H	-2.517442000	3.801217000	-1.672445000
H	-3.803142000	3.409757000	-0.500611000
H	-3.089612000	5.034421000	-0.520940000
C	-2.741370000	-2.661508000	0.541560000
H	-2.585400000	-2.473095000	1.613524000
H	-3.661756000	-2.141848000	0.236945000
H	-2.897269000	-3.739062000	0.398338000
N	-1.476742000	2.101879000	0.170187000
N	-1.351910000	-0.726856000	-0.116209000
Zn	-2.904026000	0.633024000	-0.114790000
Cl	-4.256647000	0.629283000	1.699336000
H	-1.782762000	-2.347706000	-1.345647000
H	0.177574000	-3.504449000	-0.601737000
H	-2.245843000	3.613376000	1.378457000
H	-0.329008000	4.748047000	-0.691815000
C	0.674050000	0.771523000	0.075124000
C	1.634580000	0.746036000	1.274338000
H	1.088751000	0.661549000	2.223887000
H	2.318383000	-0.106609000	1.191398000
H	2.233635000	1.663564000	1.299221000
C	1.472979000	0.876042000	-1.238811000
H	2.145006000	0.015119000	-1.339923000
H	0.804704000	0.901831000	-2.110341000
H	2.081249000	1.788649000	-1.238100000
N	-3.710758000	0.572577000	-1.948758000
N	-3.848436000	-0.424798000	-2.595300000
N	-4.003434000	-1.364390000	-3.264667000

ZnBOX-Cl -SCN (m1)

C	-0.079228000	-0.544647000	0.078478000
O	0.696025000	-1.608971000	0.256649000
C	-1.529943000	-2.235855000	-0.166673000
C	-0.169355000	-2.772140000	0.279196000
H	-0.174043000	-3.159916000	1.308677000
C	-0.218945000	1.968176000	0.275105000
O	0.433532000	3.084674000	0.578134000
C	-1.888939000	3.457401000	0.424179000
C	-0.540265000	4.159410000	0.611946000
H	-0.430673000	4.681731000	1.569014000
C	-2.744550000	3.999979000	-0.695804000
H	-2.213853000	3.948785000	-1.657840000
H	-3.677956000	3.424872000	-0.781392000
H	-3.013468000	5.047938000	-0.507048000
C	-2.700696000	-2.665514000	0.681448000
H	-2.568304000	-2.355051000	1.728493000
H	-3.635751000	-2.223078000	0.310031000
H	-2.812745000	-3.757909000	0.659158000

N	-1.492953000	2.064681000	0.158196000
N	-1.325795000	-0.775597000	-0.131199000
Zn	-2.826232000	0.597567000	-0.345370000
Cl	-4.720667000	0.608268000	0.855224000
H	-1.728969000	-2.503904000	-1.219584000
H	0.262537000	-3.528258000	-0.385099000
H	-2.471656000	3.460426000	1.361011000
H	-0.294393000	4.854925000	-0.203250000
C	0.665663000	0.769067000	0.037987000
C	1.811135000	0.759891000	1.054620000
H	1.439608000	0.659483000	2.083192000
H	2.491392000	-0.073392000	0.849151000
H	2.384724000	1.690291000	0.983472000
C	1.229741000	0.908769000	-1.393279000
H	1.906269000	0.073124000	-1.613589000
H	0.423490000	0.914879000	-2.139136000
H	1.799017000	1.842988000	-1.481325000
S	-3.243799000	0.725713000	-2.757753000
C	-3.791575000	-0.841293000	-2.744982000
N	-4.179631000	-1.955020000	-2.748237000

ZnBOX-Cl -ONO2 (m1)

C	-0.227819000	-0.581649000	-0.089306000
O	0.545303000	-1.660749000	-0.195555000
C	-1.731073000	-2.239316000	-0.157364000
C	-0.318391000	-2.819157000	-0.083162000
H	-0.102001000	-3.302597000	0.881302000
C	-0.313195000	1.945911000	0.076074000
O	0.378532000	3.082314000	0.046517000
C	-1.926219000	3.479819000	0.336864000
C	-0.560911000	4.169811000	0.226580000
H	-0.264683000	4.721562000	1.127000000
C	-2.916869000	3.844084000	-0.746047000
H	-2.493027000	3.666288000	-1.745069000
H	-3.834166000	3.244355000	-0.645026000
H	-3.200744000	4.902746000	-0.675536000
C	-2.697259000	-2.742502000	0.885448000
H	-2.305340000	-2.579376000	1.900148000
H	-3.657578000	-2.213796000	0.801681000
H	-2.887268000	-3.816539000	0.758499000
N	-1.577518000	2.052629000	0.256414000
N	-1.491027000	-0.793995000	-0.020940000
Zn	-2.983930000	0.594107000	0.028504000
Cl	-4.620789000	0.704087000	1.568507000
H	-2.165873000	-2.394888000	-1.161670000
H	-0.065564000	-3.511013000	-0.893853000
H	-2.383120000	3.659172000	1.324188000
H	-0.473219000	4.836000000	-0.642447000
C	0.541855000	0.715046000	-0.091451000
C	1.552880000	0.682079000	1.068741000
H	1.046004000	0.597812000	2.039703000
H	2.229735000	-0.172886000	0.955053000
H	2.154126000	1.598931000	1.069400000
C	1.288857000	0.818879000	-1.434101000
H	1.966788000	-0.034974000	-1.551426000
H	0.590722000	0.831310000	-2.280912000
H	1.886841000	1.737663000	-1.459459000
O	-1.977476000	0.898909000	-2.539390000
N	-3.153202000	0.588970000	-2.767283000
O	-3.946934000	0.414264000	-1.755799000
O	-3.608691000	0.442387000	-3.890959000

ZnBOX-Cl -Me (m1)

C	-0.085847000	-0.520212000	0.099558000
O	0.678964000	-1.604728000	0.263417000
C	-1.576499000	-2.166046000	-0.090570000
C	-0.212758000	-2.743814000	0.299811000
H	-0.195955000	-3.149564000	1.322708000
C	-0.206448000	1.976386000	0.274469000
O	0.420437000	3.067270000	0.725631000
C	-1.880636000	3.439777000	0.430526000
C	-0.549114000	4.143941000	0.697060000
H	-0.495578000	4.674929000	1.654195000
C	-2.755818000	4.066886000	-0.626289000
H	-2.228201000	4.137814000	-1.589108000
H	-3.659508000	3.456645000	-0.770913000
H	-3.072893000	5.076629000	-0.332099000
C	-2.723505000	-2.564957000	0.805255000
H	-2.523210000	-2.288731000	1.851067000
H	-3.646829000	-2.055633000	0.497342000
H	-2.894147000	-3.649421000	0.762357000
N	-1.458790000	2.087748000	0.038911000
N	-1.339628000	-0.715422000	-0.069497000
Zn	-2.865407000	0.664057000	-0.634905000
Cl	-4.306194000	0.817294000	1.203709000
H	-1.827256000	-2.432677000	-1.133666000
H	0.176655000	-3.503156000	-0.388145000
H	-2.463947000	3.334169000	1.362592000
H	-0.257939000	4.834530000	-0.109357000
C	0.680518000	0.780165000	0.032094000
C	1.839789000	0.764196000	1.030917000
H	1.483684000	0.665686000	2.065295000
H	2.510778000	-0.074776000	0.816417000
H	2.419476000	1.690705000	0.954434000
C	1.223964000	0.908438000	-1.406633000
H	1.895191000	0.070384000	-1.635598000
H	0.405601000	0.914678000	-2.139988000
H	1.794215000	1.841254000	-1.510307000
C	-3.525096000	0.383141000	-2.511921000
H	-2.710806000	0.407963000	-3.258931000
H	-4.013706000	-0.601927000	-2.626469000
H	-4.267771000	1.132140000	-2.840382000

ZnBOX-Cl -NO2 (m1)

C	-0.079563000	-0.473628000	-0.101262000
O	0.683493000	-1.559821000	-0.216715000
C	-1.600140000	-2.088555000	-0.406169000
C	-0.206091000	-2.702996000	-0.266812000
H	-0.072997000	-3.268315000	0.667753000
C	-0.148696000	2.041492000	0.128881000
O	0.540942000	3.179991000	0.117270000
C	-1.779535000	3.573792000	0.264401000
C	-0.418898000	4.267758000	0.133343000
H	-0.165730000	4.926307000	0.972123000
C	-2.793991000	3.904543000	-0.804514000
H	-2.375641000	3.739853000	-1.808853000

H	-3.681704000	3.260711000	-0.704026000
H	-3.117876000	4.951629000	-0.734124000
C	-2.643192000	-2.622685000	0.543782000
H	-2.336661000	-2.485718000	1.591447000
H	-3.595031000	-2.094900000	0.389397000
H	-2.817015000	-3.693563000	0.371267000
N	-1.423173000	2.149581000	0.207805000
N	-1.346974000	-0.657162000	-0.169842000
Zn	-2.877297000	0.702910000	0.101096000
Cl	-4.142626000	0.729471000	1.976592000
H	-1.978639000	-2.179144000	-1.437965000
H	0.098684000	-3.333680000	-1.109216000
H	-2.223527000	3.761187000	1.257332000
H	-0.302068000	4.827392000	-0.805574000
C	0.704734000	0.798425000	0.111038000
C	1.400952000	0.698632000	1.482709000
H	0.667611000	0.599993000	2.294683000
H	2.065479000	-0.173791000	1.504042000
H	2.005467000	1.595579000	1.665407000
C	1.753973000	0.920543000	-1.003875000
H	2.406035000	0.039740000	-1.005871000
H	1.282914000	1.006535000	-1.992436000
H	2.377643000	1.806509000	-0.839309000
N	-4.118188000	0.343048000	-1.547219000
O	-4.185231000	-0.814943000	-1.987342000
O	-4.822222000	1.230828000	-2.042034000

ZnBOX-Cl -ONO (m1)

C	-0.181784000	-0.551728000	-0.036753000
O	0.586083000	-1.635310000	-0.134445000
C	-1.697829000	-2.195644000	-0.147917000
C	-0.289339000	-2.789284000	-0.077443000
H	-0.088802000	-3.313371000	0.868571000
C	-0.268152000	1.969684000	0.141590000
O	0.425097000	3.104625000	0.211603000
C	-1.889748000	3.494876000	0.382631000
C	-0.532532000	4.191518000	0.248771000
H	-0.272200000	4.843724000	1.089816000
C	-2.937140000	3.926081000	-0.615608000
H	-2.580116000	3.789112000	-1.646794000
H	-3.857493000	3.338057000	-0.485840000
H	-3.193699000	4.985159000	-0.478382000
C	-2.666973000	-2.687767000	0.898097000
H	-2.260219000	-2.549733000	1.910690000
H	-3.611354000	-2.127747000	0.832848000
H	-2.890820000	-3.753685000	0.758743000
N	-1.543791000	2.075095000	0.209190000
N	-1.448110000	-0.753535000	-0.004818000
Zn	-2.976790000	0.609054000	0.073596000
Cl	-4.554774000	0.831572000	1.655252000
H	-2.137483000	-2.344194000	-1.150693000
H	-0.031894000	-3.450910000	-0.911840000
H	-2.293010000	3.608278000	1.404073000
H	-0.427826000	4.758798000	-0.687710000
C	0.592330000	0.742195000	-0.020524000
C	1.604959000	0.697217000	1.135110000
H	1.100387000	0.606919000	2.106653000
H	2.277605000	-0.159827000	1.012739000
H	2.211052000	1.610639000	1.142562000
C	1.330002000	0.860458000	-1.367679000
H	2.001437000	0.004789000	-1.507135000
H	0.620278000	0.889034000	-2.205581000
H	1.930571000	1.777809000	-1.388270000
N	-2.805688000	0.688316000	-2.642825000
O	-3.761014000	0.547254000	-1.759052000
O	-3.184240000	0.664481000	-3.794656000

1.4. References

(1) Frisch, M. J.; Trucks, G. W.; Schlegel, H. B.; Scuseria, G. E.; Robb, M. A.; Cheeseman, J. R.; Scalmani, G.; Barone, V.; Mennucci, B.; Petersson, G. A.; Nakatsuji, H.; Caricato, M.; Li, X.; Hratchian, H. P.; Izmaylov, A. F.; Bloino, J.; Zheng, G.; Sonnenberg, J. L.; Hada, M.; Ehara, M.; Toyota, K.; Fukuda, R.; Hasegawa, J.; Ishida, M.; Nakajima, T.; Honda, Y.; Kitao, O.; Nakai, H.; Vreven, T.; J. A. Montgomery, J.; Peralta, J. E.; Ogliaro, F.; Bearpark, M.; Heyd, J. J.; Brothers, E.; Kudin, K. N.; Staroverov, V. N.; Keith, T.; Kobayashi, R.; J. Normand; Raghavachari, K.; Rendell, A.; Burant, J. C.; Iyengar, S. S.; Tomasi, J.; Cossi, M.; Rega, N.; Millam, J. M.; Klene, M.; Knox, J. E.; J. B. Cross; Bakken, V.; Adamo, C.; Jaramillo, J.; Gomperts, R.; Stratmann, R. E.; Yazyev, O.; Austin, A. J.; Cammi, R.; Pomelli, C.; J. W. Ochterski; Martin, R. L.; Morokuma, K.; Zakrzewski, V. G.; Voth, G. A.; Salvador, P.; Dannenberg, J. J.; Dapprich, S.; Daniels, A. D.; Farkas, O.; Foresman, J. B.; Ortiz, J. V.; Cioslowski, J.; Fox, D. J. Gaussian 9 Rev. D.01. **2013**, *Wallingford, CT.*

(2) Pracht, P.; Bohle, F.; Grimme, S. Automated exploration of the low-energy chemical space with fast quantum chemical methods. *Phys. Chem. Chem. Phys.* **2020**, *22* (14), 7169–7192. DOI: 10.1039/C9CP06869D.

(3) Zhao, Y.; Truhlar, D. G. The M06 suite of density functionals for main group thermochemistry, thermochemical kinetics, noncovalent interactions, excited states, and transition elements: two new functionals and systematic testing of four M06-class functionals and 12 other function. *Theor. Chem. Account.* **2008**, *120* (1-3), 215–241. DOI: 10.1007/s00214-007-0310-x.

(4) Weigend, F.; Ahlrichs, R. Balanced basis sets of split valence, triple zeta valence and quadruple zeta valence quality for H to Rn: Design and assessment of accuracy. *Phys. Chem. Chem. Phys.* **2005**, *7* (18), 3297–3305. DOI: 10.1039/B508541A.

(5) Rappoport, D.; Furche, F. Property-optimized Gaussian basis sets for molecular response calculations. *J. Chem. Phys.* **2010**, *133* (13), 134105. DOI: 10.1063/1.3484283.

(6) Grimme, S.; Hansen, A.; Brandenburg, J. G.; Bannwarth, C. Dispersion-Corrected Mean-Field Electronic Structure Methods. *Chem. Rev.* **2016**, *116* (9), 5105–5154. DOI: 10.1021/acs.chemrev.5b00533.

(7) Cancès, E.; Mennucci, B.; Tomasi, J. A new integral equation formalism for the polarizable continuum model: Theoretical background and applications to isotropic and anisotropic dielectrics. *J. Chem. Phys.* **1997**, *107* (8), 3032–3041. DOI: 10.1063/1.474659.

(8) Marenich, A. V.; Cramer, C. J.; Truhlar, D. G. Universal Solvation Model Based on Solute Electron Density and on a Continuum Model of the Solvent Defined by the Bulk Dielectric Constant and Atomic Surface Tensions. *J. Phys. Chem. B* **2009**, *113* (18), 6378–6396. DOI: 10.1021/jp810292n.

(9) Morgante, P.; Peverati, R. Comparison of the Performance of Density Functional Methods for the Description of Spin States and Binding Energies of Porphyrins. *Molecules* **2023**, *28* (8), 3487. DOI: 10.3390/molecules28083487.
